# Weak base pairing in both seed and 3′ regions reduces RNAi off-targets and enhances si/shRNA designs

**DOI:** 10.1093/nar/gku854

**Published:** 2014-09-30

**Authors:** Shuo Gu, Yue Zhang, Lan Jin, Yong Huang, Feijie Zhang, Michael C. Bassik, Martin Kampmann, Mark A. Kay

**Affiliations:** 1 Departments of Pediatrics and Genetics, Stanford University, Stanford, CA 94305, USA; 2 Department of Genetics, Stanford University, Stanford, CA 94305, USA; 3 Department of Cellular and Molecular Pharmacology, Howard Hughes Medical Institute, University of California at San Francisco, San Francisco, CA 94158, USA

## Abstract

The use of RNA interference is becoming routine in scientific discovery and treatment of human disease. However, its applications are hampered by unwanted effects, particularly off-targeting through miRNA-like pathways. Recent studies suggest that the efficacy of such off-targeting might be dependent on binding stability. Here, by testing shRNAs and siRNAs of various GC content in different guide strand segments with reporter assays, we establish that weak base pairing in both seed and 3′ regions is required to achieve minimal off-targeting while maintaining the intended on-target activity. The reduced off-targeting was confirmed by RNA-Seq analyses from mouse liver RNAs expressing various anti-HCV shRNAs. Finally, our protocol was validated on a large scale by analyzing results of a genome-wide shRNA screen. Compared with previously established work, the new algorithm was more effective in reducing off-targeting without jeopardizing on-target potency. These studies provide new rules that should significantly improve on siRNA/shRNA design.

## INTRODUCTION

RNA interference (RNAi) is a cross-kingdom mechanism in which RNAs mediate sequence-specific gene inhibition (1,2). RNase III enzymes Drosha and Dicer process long double-stranded RNA, a common inducer of the RNAi pathway, into small interfering RNAs (siRNAs) (3). Only one strand of the resulting duplex—the guide, will associate with Argonaute protein and direct the RNA-induced silencing complex (RISC) to its targets. The other is discarded and therefore referred to as the passenger strand (4). Asymmetry in the thermodynamic stability of si/shRNA ends determines strand selection—the strand with a less stable 5′ end will be preferably loaded as guide (5,6). The ∼21-nt-long guide strand can be divided into multiple domains based on their roles in Argonaute association, target recognition and repression function (7). In mammals, extensive base pairing between the guide strand and target mRNA enables Argonaute 2-mediated cleavage, resulting in the destruction of target mRNA and robust inhibition (8). In light of these mechanistic insights, it is possible to piggyback onto the RNAi machinery to suppress the expression of almost any desired target gene by applying an siRNA with a complementary sequence (9).

In nature, endogenous microRNAs (miRNAs) regulate gene expression using the same RISC complex pathway (10). In contrast to artificially designed siRNAs, most mammalian miRNAs are partially complementary to their targets, inducing non-cleavage repression, which usually leads to mild inhibition (11). Base paring as short as 6 bp in the seed region is sufficient for many miRNAs to function (12). As a result, each miRNA has hundreds, if not thousands, potential targets (13).

One of the major hurdles preventing RNAi from achieving its full potential is the lack of specificity (2,14,15). Robust knock-down of intended targets is often accompanied by undesired off-target effects (16). The factors that may contribute to these side effects include: (i) an innate immune response, which can be alleviated by the inclusion of chemical modifications of the siRNA backbone (17,18) and avoidance of specific sequence motifs (19); (ii) saturation of endogenous miRNA machinery with the addition of large amounts of siRNA/shRNAs (20); (iii) unintentional loading of the passenger strand into RISC. Recent developments in siRNA/shRNA design have improved upon strand-specific loading of the guide RNA into RISC (21); (iv) the RISC-associated guide strands can still downregulate endogenous mRNAs containing partial complementary target sequences through miRNA-like pathways. These off-target effects not only generate false signals in RNAi-based screens (22) but can also cause substantial toxicity and fatality in a therapeutic setting (23). Despite some efforts (24,25), reduction in miRNA-induced off-targeting remains a challenge.

DNA-encoded RNAi species (shRNA expressed from plasmids) (26) are preferred or in some cases required in genetic screens and specific RNAi therapeutic approaches (27). Since chemical modification is unavailable for expressed shRNAs, the performance of an expressed shRNA will solely depend on its sequence design. Currently, major shRNA/siRNA design algorithms have focused on knock-down efficacy over specificity. Here, we present a novel approach to design potent shRNA/siRNA with minimal off-target effects.

## MATERIALS AND METHODS

### shRNA design and Plasmid construction

To take into account the loop-counting rule we previously established (21), each shRNA was designed as 21-mer stem–loop with the guide strand in the 3p arm. A guide strand always starts with U and contains a C in the 19th position. These are required to promote incorporation into RISC. Therefore, nucleotides 2–18 represent the seed, central and 3′ regions and are complementary to the target sequence. In our study, the shRNAs were named after the sequence motifs used in the seed and 3′ regions. In addition, we included in the name a reference to their GC content—S stands for GC-rich sequences (strong binding to targets), W for AU-rich sequence (weak binding to targets) and N for random sequences of either high or low GC content. For example, the guide sequences of sh-S1_N and sh-W1_N are UUGACCAGCCG*GAGCUUA*CUU and UUAUAAUACCG*GAGCUUA*CUU, respectively. The two shRNAs have the same sequence in the 3′ region (italic font)—motif N. The seed sequences (underlined) are different. The seed of sh-S1_N has high GC content (motif S1) while the seed of sh-W1_N is AU-rich (motif W1). Motif sequences can be found in Supplementary Table S1. All shRNAs were directly cloned downstream of U6 Pol III promoter between BglII and KpnI. For cloning of target sequences into the psi-CHECK2 reporter system, both strands of the insert were chemically synthesized, annealed, purified and inserted between the XhoI and SpeI sites of a modified psi-CHECK2 vector (Promega). The modification contained additional cloning sites in its 3′ untranslated region (3′ UTR). Of note, the target flanking sequences were maintained for all the psi-CHECK2 reporters. No stable local structure was formed (checked by mfold). All target sites therefore have similar accessibility. The sequences of oligos used in the cloning are listed in Supplementary Table S2.

### Cell culture and transfection

HEK293 and mouse embryonic fibroblast (MEF) cells were grown in Dulbecco's modified Eagle's medium (Gibco-BRL) with L-glutamine, non-essential amino acids, sodium pyruvate and 10% heat-inactivated fetal bovine serum with antibiotics. All cells were tested to be free of mycoplasma contamination. All transfection assays were done using Lipofectamine 2000 (Invitrogen) following the manufacturer's protocol.

### Dual-luciferase reporter assay

A psi-CHECK2 reporter containing a perfectly matched or mismatched target in its 3′ UTR was used to measure on-target or off-target effects, respectively. Hundred nanograms of reporter plasmids were co-transfected with either 100 ng of shRNA-plasmid or a specified amount of synthetic siRNA into HEK293 or MEF cells in a 24-well plate. Twenty-four hours post-transfection, FF-luciferase and RL-luciferase activities were measured using Promega's dual-luciferase kit (cat E1980) protocol and detected by a Modulus Microplate Luminometer (Turner BioSystems).

### Northern blots

HEK293 cells in 6-well dishes were transfected with 1 ug of an shRNA expression plasmid. Twenty-four hours post-transfection, total RNA was isolated using Trizol (Invitrogen) and then electrophoresed on 15% (w/v) acrylamide/7-M urea gel. After transfer onto a Hybond-N1 membrane (Amersham Pharmacia Biotech), small RNAs were detected using P32-labeled probes of complementary sequences (Supplementary Table S2) (http://bartellab.wi.mit.edu/protocols/smallRNA_northern.pdf).

### Mouse studies

All animal studies were done in concordance with the US National Institutes of Health guidelines and the Stanford Animal Care Committee. Ten female BALB/c mice, 6–8 weeks of age (Jackson Laboratory), were randomly selected and hydrodynamically infused with a mixture of 6 mg of the appropriate shRNA plasmid and 4 mg of the pBluescript plasmid DNA (Stratagene) included as a carrier. Blood was collected via retro-orbital bleeding, and serum alanine aminotransferase (ALT) levels were determined as previously described (23). The liver was harvested 7 days post-injection, frozen in liquid nitrogen and then ground into a powder. Liver total RNA was isolated using Trizol (Invitrogen). The investigators who handled the mouse injections, RNA extraction, library construction and data analysis were blinded to the various experimental groups.

### RNA-Seq and gene expression profiling

An RNA-Seq library was created by using a TruSeq kit (illumina). All samples were barcoded and then mixed into one library. Sequencing reads (101 bp, paired-end) were generated using the Illumina Hi-Seq (Stanford Cancer Institute Core Facility). An average of 18.1 million paired-end reads per sample were mapped to a known transcriptome sequence from UCSC mm9 RefSeq using Bowtie 2 (average 82.67% alignment rate). Singleton reads were discarded. For genes with alternative isoforms, the multi-reads were distributed randomly among isoforms. To determine the mRNA expression as a measure of gene expression, we used the abundance–variance among isoforms to estimate the effective length for each gene. Firstly, abundance in isoform level was obtained by counting the number of reads mapping to individual isoform for each gene. Secondly, a formula was used to calculate the effective length for each gene, i.e. ${\rm l}_{{\rm eff}} = \sum\nolimits_{{\it i} = 1}^{\it g} {{\rm l}_{\it i} \frac{{{\it F}_{\it i} }}{{\sum\nolimits_{{\it i} = 1}^{\it g} {{\it F}_{\it i} } }}}$, while *g* is the total number of isoforms in one particular gene, *l_i_* is the length for isoform *i*, *F_i_* is the number of fragments (a fragment is defined as a pair of read) mapped to isoform *i*. Then, the effective gene length is involved to calculate the fragments per kilobase per million total mappable reads (FPKM), to represent the abundance of each gene expression. This method can capture gene expression profile by considering both relative structure change and absolute abundance fluctuation among the different conditions.

The Jensen–Shannon divergence (JSD; also known as information radius) was used to measure the similarity between two transcriptomes and is based on the Kullback–Leibler (K–L) divergence. In detail, for each sample, the gene FPKM value and the normalized gene expression were obtained using Equations (1) and (2), respectively. Then K–L divergence between two samples *P* and *Q* was determined using Equation (3). Finally, the JSD was calculated using Equations (4) and (5). JSD is a symmetric matrix, and each value is bounded by 0 and 1. JSD is equal to 0 when the two transcriptomes are the same. The JSD value is 1 when the two transcriptomes are totally independent. The scatter plots using the gene FPKM value for a direct comparison between control and individual samples can be found in Supplementary Figure S3H:

(1) \begin{equation*} p_i = \frac{{{\rm FPKM}_i }}{{\sum\nolimits_{i = 1}^g {{\rm FPKM}_i } }} \end{equation*}

(2) \begin{equation*} \sum\nolimits_{i = 1}^g {p_i = 1} \end{equation*}

(3) \begin{equation*} D(p||Q) = \sum\nolimits_{i = 1}^g {\log _2 \left( {\frac{{p_i }}{{q_i }}} \right)p_i } \end{equation*}

(4) \begin{equation*} M = \frac{1}{2}(P + Q) \end{equation*}

(5) \begin{equation*} {\rm JSD}(P||Q) = \frac{1}{2}D(P||M) + \frac{1}{2}D(Q||M). \end{equation*}

### Calculating optimal design score

The optimal design (OD) score was calculated by taking the sum of the values assigned to each position in the guide strand according to its nucleotide composition. The positional weight values were derived from an *in vitro* biochemical binding assay (7). Mutations at each position have different impacts on the binding constant (*K*_M_) between RISC and target. Weight values were determined to reflect such a relationship where higher values were assigned to positions contributing more to the overall binding affinity. The ends were fixed and therefore do not contribute to the score. AU nucleotides were scored if they were in the seed or 3′ region while GC nucleotides were scored for being in the central region. The average score for AU was higher in the seed versus the 3′ region to reflect the superior role of seed in target recognition. The score matrix is shown in Figure 4A.

**Figure 1.**
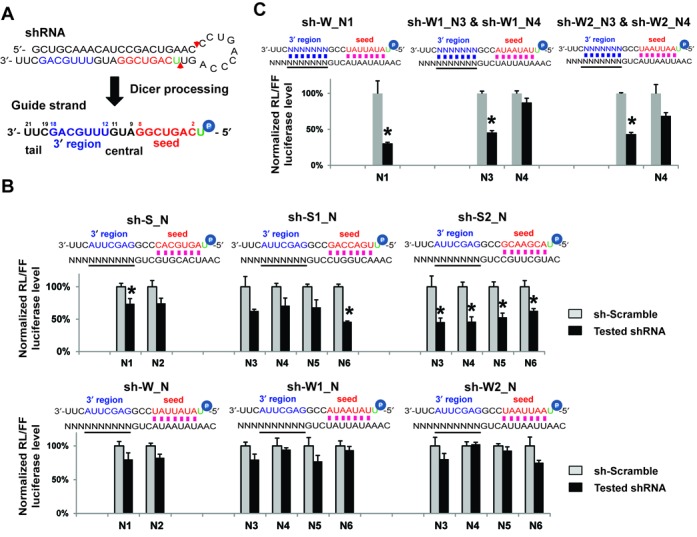
Base pairing beyond the seed region contributes to off-target effects. (**A**) Diagram depicting the processing of an shRNA (sh-miR30-21) by Dicer and the resulting guide strand with different domains: seed (bases 2–8), central (bases 9–11), 3′ region (bases 12–18) and tail (bases 19–21) (7). (**B**) Measuring miRNA-like off-target effects by dual-luciferase reporter assay. The psi-CHECK2 vector with one target site in the 3′ UTR and DNA plasmids expressing shRNA were co-transfected into HEK293 cells. Base pairing between guide strand and target is illustrated. shRNAs were named by symbols representing the seed and 3′ region sequences, with a underline in between. S stands for GC-balanced motif and therefore generates relatively Strong binding; W stands for AU-rich motif and Weak binding; N stands for random sequences (Supplementary Table S1). Sequence motifs used in the underlined region of target are labeled in the x-axis. In each combination, RL-luciferase activities were normalized with FF-luciferase, and the percentage of relative enzyme activity (dark bar) compared to the negative control (treated with sh-scramble, gray bar) was plotted. Error bars represent the SD from two independent experiments, each performed in triplicate transfections. *P (*t*-test, two tailed) < 0.0001 compared with sh-scramble control treatment. (**C**) Dual-luciferase assay shows that base pairing in the 3′ region contributes to off-target effects. Different from experiments in (B), each shRNA–target combination tested had sequence complementarity in the 3′ region in addition to the seed. Results were plotted as described above. Different sequence motifs used in the 3′ region of guide strand were indicated by the name of the shRNA tested.

**Figure 2.**
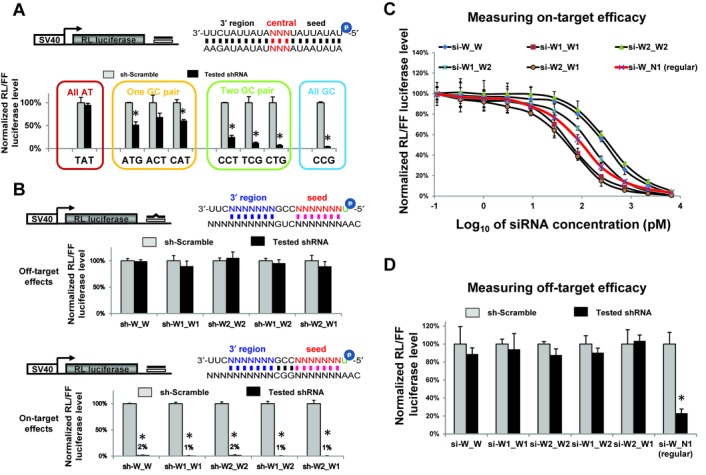
AT-enrichment in both seed and 3′ regions is an optimal design to reduce miRNA-like off-target effect. (**A**) On-target efficacy of shRNAs with no, one, two or three GC pair(s) in the central region was measured by dual-luciferase reporter assay in HEK293 cells. shRNAs tested had all-AU seed and 3′ region sequences. The guide strand and target were perfectly matched in all cases. Sequences used in the central region of guide strand are labeled on the x-axis. RL-luciferase activities were normalized with FF-luciferase, and the percentage of relative enzyme activity (dark bar) compared to the negative control (treated with sh-scramble, gray bar) was plotted. Error bars represent the SD from two independent experiments, each performed in triplicate transfections. *P (*t*-test, two tailed) < 0.0001 compared with sh-scramble control treatment. (**B**) Validation of the new design by dual-luciferase assay in HEK293 cells. As illustrated in the figure, a perfectly matched target was used to measure the on-target effect while a central-mismatched target was used to capture the miRNA-like off-target effects. All tested shRNAs have a GC-enriched central region and an AU-enriched seed and 3′ region. Sequences used in the seed and 3′ regions were indicated in the shRNA name. Symbol before the underline represents the seed sequence. Results were plotted as described above. (**C**) AU-enriched siRNAs are as potent as regular siRNAs (si-W_N1) with respect to on-target knock-down. Each siRNA was transfected with psi-CHECK2 vector containing one perfectly matched target site in the 3′ UTR. Every siRNA was tested at various concentrations in HEK293 cells. RL-luciferase activities were normalized with FF-luciferase, and the percentage of relative enzyme activity compared to the si-scramble negative control was plotted against the final concentration of siRNAs on a log scale. Error bars represent the SD from two independent experiments, each performed in triplicate transfections. (**D**) The off-target effects of siRNAs tested in (C) were measured by being co-transfected with psi-CHECK2 vector containing one central-mismatched target in the 3′ UTR in HEK293 cells. All siRNAs were tested at a final concentration of 30 nM, which is five times of the highest concentration used in (C). The result was plotted as described above.

**Figure 3.**
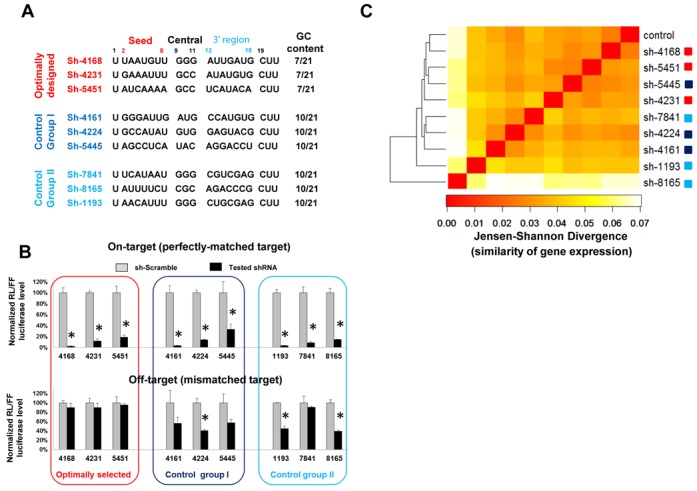
Silencing efficacy and off-targeting of anti-HCV shRNAs. (**A**) Design of shRNAs against HCV genome. Guide strand sequences (5′ to 3′) are listed. (**B**) On-target silencing efficacy and off-target effects were measured by dual-luciferase reporter assays in HEK293 cells as described earlier. *P (*t*-test, two tailed) < 0.0001 compared with sh-scramble control treatment. (**C**) The off-target spectrum of anti-HCV shRNAs was evaluated *in vivo*. shRNAs were expressed in mice and the resulting mRNA expression profiles in mouse livers were determined by RNA-Seq. All mRNAs with a value of FPKM higher than 2 were considered (*n* = 9000). Clustering of mRNA expression signature figures is shown. Average linkage hierarchical clustering was used with distance between samples measured by the square root of the Jensen–Shannon divergence (JSD). A low Jensen–Shannon distance corresponds to similar gene expression profiles (red shades of colors in the heat map). Scatter plots-based RNA expressions between individual samples and control can be found in Supplementary Figure S3H. Also see the Materials and Methods section for details.

**Figure 4.**
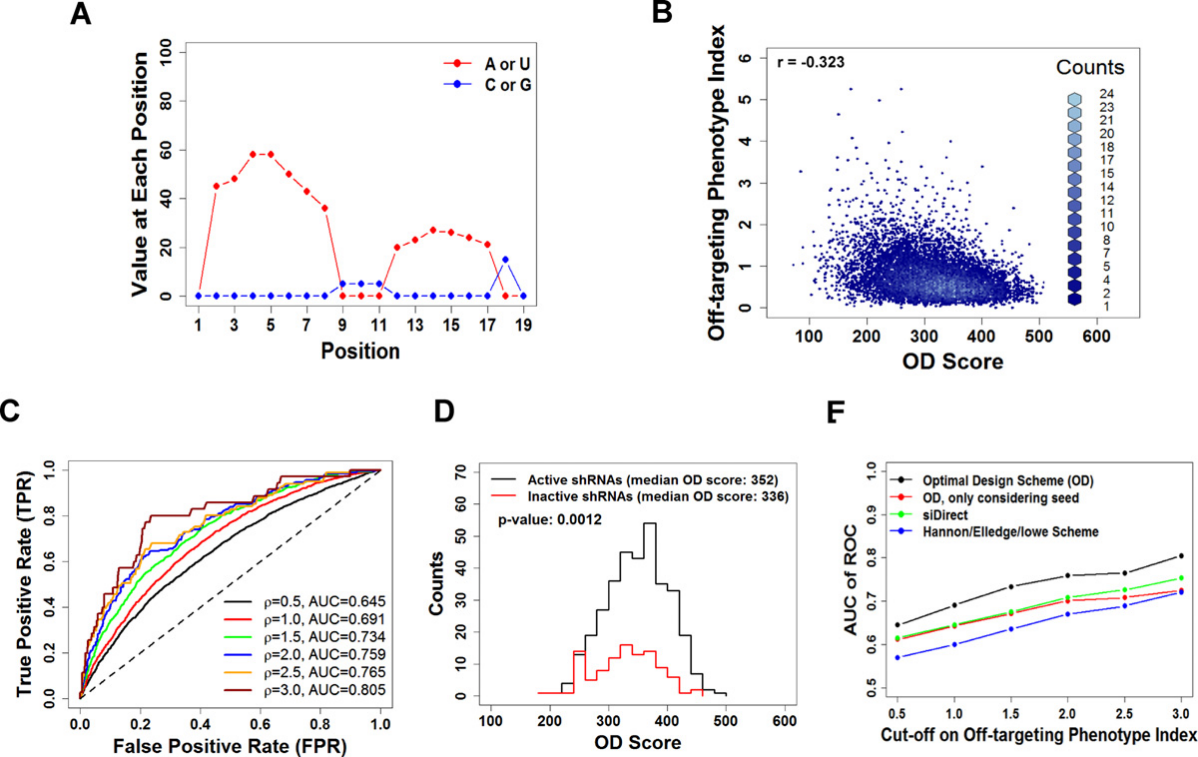
Evaluation of the new design by a large data set from shRNA library screen. (**A**) Scoring matrix used to calculate the optimal design (OD) score. X-axis is the relative position counted from the 5′ end of guide strand. See the Materials and Methods section for details. (**B**) Off-targeting phenotype index was plotted against OD score for over 10 000 shRNAs. (**C**) Based on the phenotype index, shRNAs were classified as strong off-target effect (positive) or no strong off-target effect (negative). Receiver operating characteristic (ROC) curve was created by plotting true positive rate (TPR) against false positive rate (FPR) at various threshold settings of OD score. Different value of phenotype index was used as cutoff (labeled as ρ in the figure) to distinguish positive (strong off-targeting) from negative (no strong off-target). Area under the curve (AUC) presenting the prediction power of the algorithm was calculated and indicated in the figure at various ρ values. (**D**) OD score distributions are plotted for a high-confidence set of active and inactive shRNAs. (**E**) Power to predict low off-targeting was measured as AUC and compared between algorithms. The Design scheme presented in this study is more predictive compared to the Hannon/Elledge/Lowe (40,41) and the siDirect scheme (42).

### Comparing siRNA/shRNA design tools

To directly compare different algorithms, scores were generated for any given shRNA sequence to quantify each design scheme by applying the corresponding algorithm. We were able to obtain the score matrix of the Hannon/Elledge/Lowe scheme but not the siDirect scheme. Nonetheless, the siDirect scheme ranked the capability of an siRNA to induce off-target effects based on the melting temperature (standard free-energy change) of its seed sequence. We therefore calculated the seed melting temperature and used it as the score for siDirect design. The formula used for the melting temperature calculation is as follows: *T*_m_ = {(1000 x Δ*H*)/[*A* + Δ*S* + ln(*C*_t_/4)]}*273.15 + 16.6log[Na_+_]. Δ*H* (kcal/mol), sum of nearest-neighbor enthalpy change. *A*, helix initiation constant (–10.8). Δ*S*, sum of nearest-neighbor entropy change. *R*, gas constant (1.987 cal/deg/mol). *C*_t_, total molecular concentration of strand (100 nM). [Na_+_] was fixed at 100 mM (28).

## RESULTS

### Base pairing beyond the seed region contributes to off-target effects

We began the study by applying the ‘loop-counting rule’ (21) and designed shRNAs with a 21-mer stem–loop. This allowed us to precisely determine the Dicer cleavage site and the exact sequences of the guide strand domains processed from the 3p arm of the shRNA (Figure 1A). Because base pairing in the ends of the guide strand contributes little to on-target cleavage (29), we fixed the first position as a uracil (U) and the 19th position as a cytosine (C) regardless of the target sequences. This ensured the thermodynamic difference between the 5′ ends, which in turn favored loading guide over passenger strand during RISC assembly. Indeed, only the guide, but not the passenger strand of sh-miR30-21 could inhibit the reporter containing a perfectly complementary target in its 3′ UTR (Supplementary Figure S1A). While passenger-strand-mediated off-target effects were successfully eliminated by this design, off-targeting originating from the guide strand was not abrogated—sh-miR30-21 induced notable repression of a reporter gene with a central-mismatched target to the guide strand (Supplementary Figure S1A).

Because the guide strand inhibits undesired targets through partial base pairing just like an miRNA (30) and given the critical role of the seed sequence in miRNA target recognition, such off-targeting events are often reported to be seed related (25,31,32). miRNA function can be disrupted by ongoing translation (33,34), suggesting miRNA activity is dependent on a relatively stable association between guide strand (RISC) and target mRNA. Therefore, guide strands with AU-rich seeds should form weak base pairing with undesired targets and exert less repression. To validate this assumption experimentally, we designed six shRNAs that varied only in their seed sequences. Each shRNA was co-transfected with multiple dual-luciferase reporters in HEK293 cells (Figure 1B). All reporters contained a seed-matched target in their 3′ UTR to capture the miRNA-like off-targeting from the corresponding shRNA. While all expressed well (Supplementary Figure S1B), only shRNAs containing a GC-balanced seed, but not an AU-rich seed, mediated notable repression of their targets (Figure 1B). A similar result was obtained in MEF cells (Supplementary Figure S1C) and reported previously (25,35), suggesting reducing the binding stability between guide strand and target mRNA could offer an effective way to eliminate miRNA-like off-targeting.

Recent findings that non-canonical miRNA target sites do not have perfect seed match indicate that the guide strand sequence outside of the seed region also contributes to miRNA target recognition (36). Therefore, base pairing outside the seed region should affect off-targeting as well. To test this, we designed five shRNAs with AU seed sequences and measured their off-target efficacies using a luciferase reporter assay. Unlike the previous experiment, the 3′ regions of the guide strand were also base-paired to the targets (Figure 1C). Despite a similar expression level (Supplementary Figure S1D), off-targeting was observed with some, but not all, of the shRNAs tested in both HEK293 (Figure 1C) and MEF cells (Supplementary Figure S1E). Nonetheless, these results demonstrate that designing shRNAs with an AU-rich seed is inadequate to avoid off-target effects.

### AT-enrichment in both seed and 3′ regions is an OD to reduce miRNA-like off-target effect

We next sought to reduce binding stability by maximizing AU content beyond the seed region. Indeed, off-target effects were almost undetectable when five shRNAs with extensive AU sequences were tested (Supplementary Figure S2A). However, the on-target knock-down efficiencies were correspondingly reduced (Supplementary Figure S2A). *In vitro* biochemical studies indicated that base paring in the central region was critical for on-target cleavage but less important for RISC–target association (7). Consistent with this idea, we found that on-target knock-down efficacy was positively correlated with the GC content in the central region when the rest of the guide strand was AU-rich (Figure 2A and Supplementary Figure S2B). Taken together, these results indicate that AU enrichment in both the seed and 3′ regions, but not the central region, could be an OD scheme. Indeed, five shRNAs designed using these criteria were shown to have potent on-target activity while having minimal off-target effects in both HEK293 cells (Figure 2B) and MEF cells (Supplementary Figure S2C).

We noticed that the processing of AU-rich shRNAs was relatively inefficient, resulting in a lower level of mature guide strand RNAs (Supplementary Figure S2D). This raised the concern that the reduced off-targeting was merely due to a dose effect. To address this possibility directly, we chemically synthesized siRNAs with the same sequences as those produced from the AU-rich shRNAs. The half maximal inhibitory concentration (IC_50_) was measured by co-transfecting different amounts of each siRNA together with a luciferase reporter containing the perfectly matched target in HEK293 cells. The IC_50_ values of five AU-rich siRNAs (60–300 pM) were comparable to that of the control siRNA (GC balanced, IC_50_ ∼ 100 pM), demonstrating the AU-rich design did not reduce the on-target potency (Figure 2C). We then measured their off-target effects at a concentration of 30 nM, at which the maximal on-target knock-down could be achieved for all tested siRNAs. Consistent with shRNAs, siRNAs with a GC-balanced sequence, but not the AU-rich siRNAs, induced miRNA-like off-targeting (Figure 2D). These results demonstrate that our general algorithm is effective in designing both shRNAs and siRNAs.

### Silencing efficacy and off-targeting of anti-hepatitis C virus shRNAs

To validate our findings in a relevant preclinical setting, we created nine shRNAs against the hepatitis C virus (HCV) genome. All were designed as a 21-mer stem–loop structure with fixed ends as described previously, leaving only positions 2–18 as guide strand variables. Three were designed by the new scheme: GC-rich sequences in the central and AU-rich sequences in both seed and 3′ regions. Three shRNAs with balanced GC content (group I) and another three containing an AU-rich seed but overall balanced GC content (group II) were selected as controls (Figure 3A). As expected, all anti-HCV shRNAs had marginal passenger strand-mediated off-target effects (Supplementary Figure S3A) and relatively potent on-target activity (Figure 3B). In contrast, the guide-strand-mediated miRNA-like repression (off-targeting parameter) varied (Figure 3B). The degree of off-targeting did not correlate with the amount of guide strand (Supplementary Figure S3B), but rather with the GC content distribution profile. Off-targeting was observed in both control groups, but not with shRNAs containing AU-rich sequences in both seed and 3′ regions (Figure 3B). Similar observations were made in MEF cells (Supplementary Figure S3C). Together, our results demonstrate that potent anti-HCV shRNAs with reduced off-targeting could be achieved by following the new design scheme.

To further validate the low off-targeting potential of these anti-HCV shRNAs *in vivo*, we transfected shRNA-expressing plasmids into the mouse liver via a hydrodynamic tail vein infusion, a method known to transfect up to 30% of mouse hepatocytes *in vivo*. A plasmid backbone lacking the shRNA sequence was used as a negative control. After 7 days, we evaluated the potential toxicity by measuring the serum alanine transaminase (ALT) levels. No ALT elevation was observed with any of these shRNA treatments (Supplementary Figure S3D). This was most likely due to the modest shRNA expression level (Supplementary Figure S3E), which was not sufficient to cause liver injury as we had observed in the past (23). To look at the off-targeting in detail, mouse livers were harvested, and RNA was isolated from these and subjected to RNA-Seq analysis. Most miRNA-like repression leads to a reduction in the corresponding mRNA (37). As expected, transcripts containing seed binding sites for the guide strand of sh-4168 (optimally designed) were much less downregulated relative to negative controls than those targeted by sh-8165 and sh-1193 (group I and II controls) (Supplementary Figure S3F). However, this pattern was not observed when all nine shRNAs were taken into consideration (Supplementary Figure S3G). This indicates that the observed gene expression changes could not simply be explained by seed-mediated gene silencing. The very nature of ‘off’-targeting made it difficult to predict which subgroup of genes was directly affected by transfected shRNAs—some mRNAs might be repressed through seedless non-canonical target sites. Nonetheless, because there were no endogenous targets of the anti-HCV shRNAs, all off-targeting effects, direct or indirect, would eventually be reflected in the changes in the overall gene expression signature. Therefore, we used mRNA expression profiling to measure the off-targeting of these anti-HCV shRNAs.

RNA-Seq detected more than 9000 genes highly expressed in the liver. To quantify the expression difference between the samples, we used hierarchical clustering based on JSD distances to compare the relative expression of each gene. Results were concordant with the data obtained from the reporter assay in tissue-culture cells. Despite any differences in the shRNA expression level (Supplementary Figure S3E), samples treated with optimally designed shRNAs clustered closely to the negative control (Figure 3C and Supplementary Figure S3H), indicating these shRNAs had minimal off-target effects. A biological repeat was performed with a new set of mice. Barcode/adaptor sequences were switched during library construction to eliminate potential sequencing bias (38). Again, three optimally designed shRNAs induced the least alterations in the gene expression pattern (Supplementary Figure S3I). These results demonstrate that the new design scheme is effective in reducing shRNA-mediated off-target effects in *in-vivo* applications.

### Evaluation of the new design by a large data set from shRNA library screen

Based on these findings, we formulated an OD score for each guide strand sequence to quantify our design scheme (Figure 4A). A high score predicts low off-targeting potential. To assess our algorithm on a larger scale, we took advantage of a recently published genome-wide screen for ricin resistance using a Pol II-transcribed ultra-complex shRNA library (39). This library contains a large set (over 10 000) of negative control shRNAs designed to not target any human gene within three base substitutions. Phenotypes observed for these negative-control shRNAs reflect stochastic noise inherent to pooled screening approaches, as well as off-target effects on genes involved in ricin susceptibility. Ricin resistance phenotypes for negative-control shRNAs measured in six independent experiments (39) were averaged to reduce stochastic noise. The absolute value of the average phenotype was used as an off-targeting phenotype index (Supplementary Table S4). This screen study therefore provided an extensive list of shRNAs with experimentally measured off-target effects to validate the design scheme.

After analyzing over 10 000 shRNAs, we observed a negative correlation between phenotype index and OD score, demonstrating that the OD score predicted a lower likelihood of off-target effects as expected (Figure 4B). The correlation was relatively weak (*R* = −0.32), most likely because only a fraction of off-target effects, namely those resulting in a ricin phenotype, would be detected in this particular screen. Using a specific cutoff value of the off-targeting phenotype index, we classified tested shRNAs into ‘strong off-target effects’ and ‘no strong off-target effects’. At the same time, a prediction was made by applying a specific OD score as a discriminate threshold. With a binary classifier system, the sensitivity and specificity of our algorithm could be measured by calculating the true positive rate (TPR; the fraction of true positives out of the total actual positives) and the false positive rate (FPR; the fraction of false positives out of the total actual negatives), respectively. To illustrate the performance of our algorithm, we generated a receiver operating characteristic (ROC) curve by plotting TPR over FPR at various settings where different OD score was used as the threshold (Figure 4C). The prediction power was evaluated by calculating the area under the curve (AUC) of the ROC curve. Interestingly, the prediction power increased when more stringent conditions (higher phenotype index value) were used to classify the positive group, demonstrating our algorithm was very effective (AUC > 0.8) in designing shRNAs free of strong off-target effects (phenotype index > 3) (Figure 4C).

We also checked the correlation between OD score and on-target efficacy. For shRNAs targeting genes with known function in ricin susceptibility, their on-target efficiencies could be easily measured by the corresponding phenotypes (39) (Supplementary Table S5). Of note, shRNAs in this library were designed following a well-developed protocol (40). It was not surprising that we identified more active shRNAs than inactive shRNAs of high confidence. Interestingly, the median OD score of active shRNAs was slightly higher than that of inactive shRNAs (Figure 4D), indicating our algorithm generates shRNAs with similar, if not higher, on-target potency than those designed by established protocol.

To further evaluate the ability to predict off-targeting, we compared our design strategy to other published algorithms. One of the most popular design schemes is the Hannon/Elledge/Lowe method, which is an empirical algorithm trained by results from more than 20 000 shRNAs (40,41). Another is siDirect, which correlates the siRNA/shRNA off-targeting potential to the melting temperature (*T*_m_) of its seed sequences (42). Using the ricin shRNA screen data as input, we plotted the predictions from all algorithms against the measured off-targeting phenotype index and created the respective ROC curves (Figure 4). Our design scheme had the highest AUC, highlighting its ability to predict low off-target potential (Figure 4E).

## DISCUSSION

Previously published efforts to reduce off-targeting were centered on properties of the seed region (24,25). Here, multiple lines of evidence suggest that base pairing beyond the seed region should also be considered. First, dual-luciferase reporter assays showed that a relatively stable binding between the target and the 3′ region of the guide strand enhances off-targeting. Second, shRNAs without an AU-rich 3′ region (control group II) disturbed the mRNA expression profile more vigorously *in vivo* when introduced into the mouse liver. Finally, when the OD score was modified to consider only the seed region, its ability to predict off-targeting fell to the level of previously established design schemes (Figure 4E).

Considered together, our results provide new rules for shRNA design, enabling the creation of potent RNAi triggers with substantially reduced off-target effects. These rules were established by reporter assays in cell lines, and further validated both *in vivo* and when tested at large scale. These improved design rules have wide application—we have shown that they were effective in designing either Pol II or Pol III promoter-driven shRNAs, as well as synthetic siRNAs. Applying these rules, we have developed a freely available algorithm to facilitate the design of optimal sh/siRNAs (http://web.stanford.edu/group/markkaylab/cgi-bin/). Finally, our results suggest that guide sequences in addition to the seed sequence, particularly the 3′ region, might have more important roles in miRNA target recognition and function than previously believed. Future improvement in si/shRNA design will rely on additional insights gained through mechanistic study of the RNAi pathway.

## ACCESSION NUMBERS

Raw data and processed FPKM table of the nine anti-HCV shRNAs RNA-seq experiments can be accessed by GSE55131 from the NCBI Gene Expression Omnibus. The library sizes can be found in Supplementary Table S3.

## SUPPLEMENTARY DATA

Supplementary Data are available at NAR Online.

SUPPLEMENTARY DATA
