# Supplementary material for: Weak base pairing in both seed and 3′ regions reduces RNAi off-targets and enhances si/shRNA designs
Source: Nucleic Acids Res. 2014 Sep 30;42(19):12169–76. doi: 10.1093/nar/gku854 (PMC4231738; doi:10.1093/nar/gku854)
Supplement: SUPPLEMENTARY DATA [file supp_42_19_12169__index.html]

Weak base pairing in both seed and 3′ regions reduces RNAi off-targets and enhances si/shRNA designs — Weak base pairing in both seed and 3′ regions reduces RNAi off-targets and enhances si/shRNA designs — SUPPLEMENTARY DATA 

# Weak base pairing in both seed and 3′ regions reduces RNAi off-targets and enhances si/shRNA designs

## SUPPLEMENTARY DATA

**Files in this Data Supplement:**

- SUPPLEMENTARY DATA
- SUPPLEMENTARY DATA
- SUPPLEMENTARY DATA
- SUPPLEMENTARY DATA
