# Supplementary material for: Weak base pairing in both seed and 3′ regions reduces RNAi off-targets and enhances si/shRNA designs
Source: Nucleic Acids Res. 2014 Sep 30;42(19):12169–76. doi: 10.1093/nar/gku854 (PMC4231738; doi:10.1093/nar/gku854)
Supplement: SUPPLEMENTARY DATA [file supp_gku854_nar-00955-y-2014-File006.pdf]

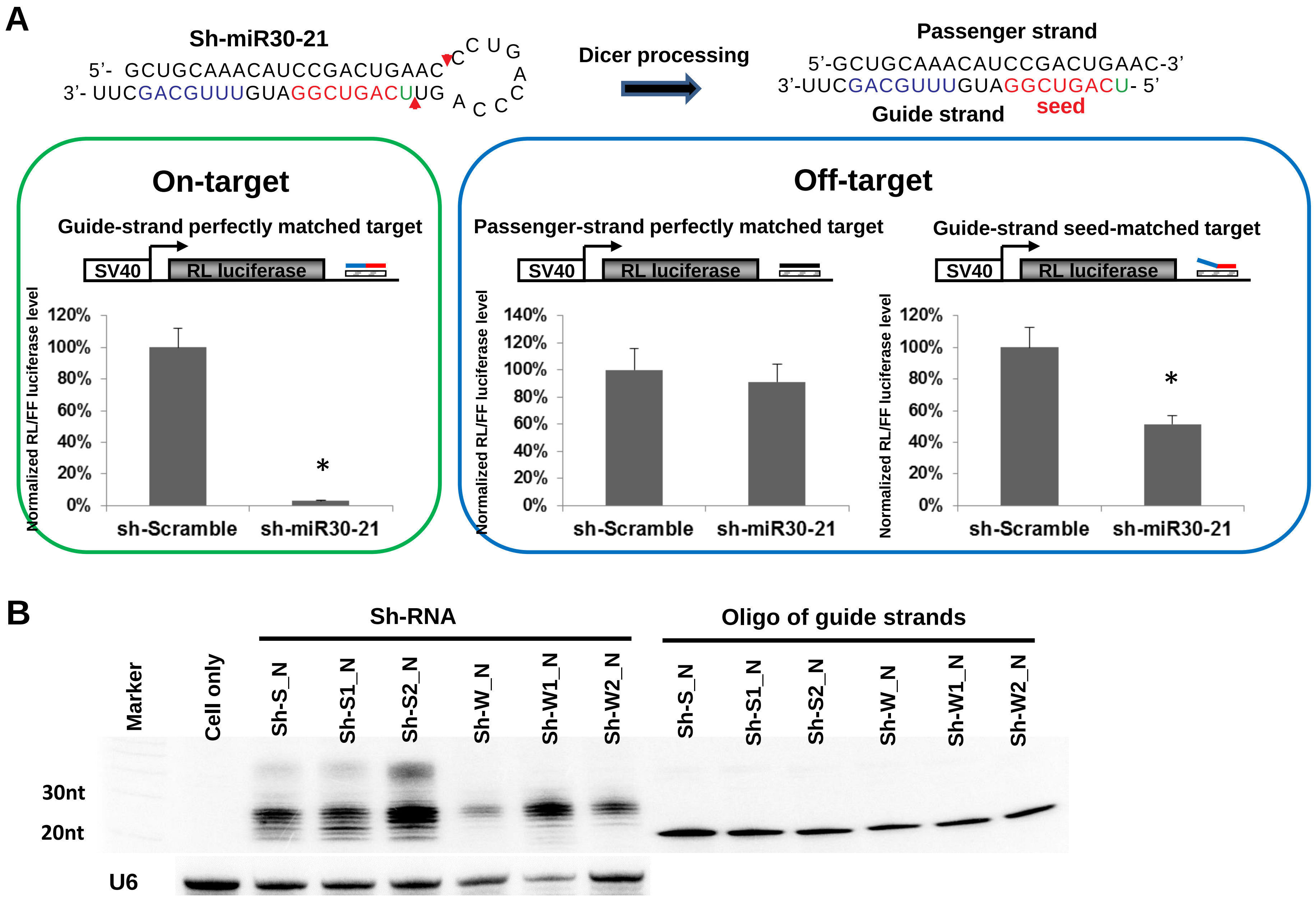

A
Sh-miR30-21
5'- GCUGCAAACAUCCGACUGAAC
3'- UUCGACGUUUGUAGGCUGACUUG
Dicer processing
Passenger strand
5'-GCUGCAAACAUCCGACUGAAC-3'
3'-UUCGACGUUUGUAGGCUGACU- 5'
Guide strand
seed
On-target
Guide-strand perfectly matched target
SV40
RL luciferase
Normalized RL/FF luciferase level
sh-Scramble
sh-miR30-21
Off-target
Passenger-strand perfectly matched target
Guide-strand seed-matched target
B
Sh-RNA
Oligo of guide strands
Marker
Cell only
Sh-S_N
Sh-S1_N
Sh-S2_N
Sh-W_N
Sh-W1_N
Sh-W2_N
30nt
20nt
U6

Figure S1 (A-B)

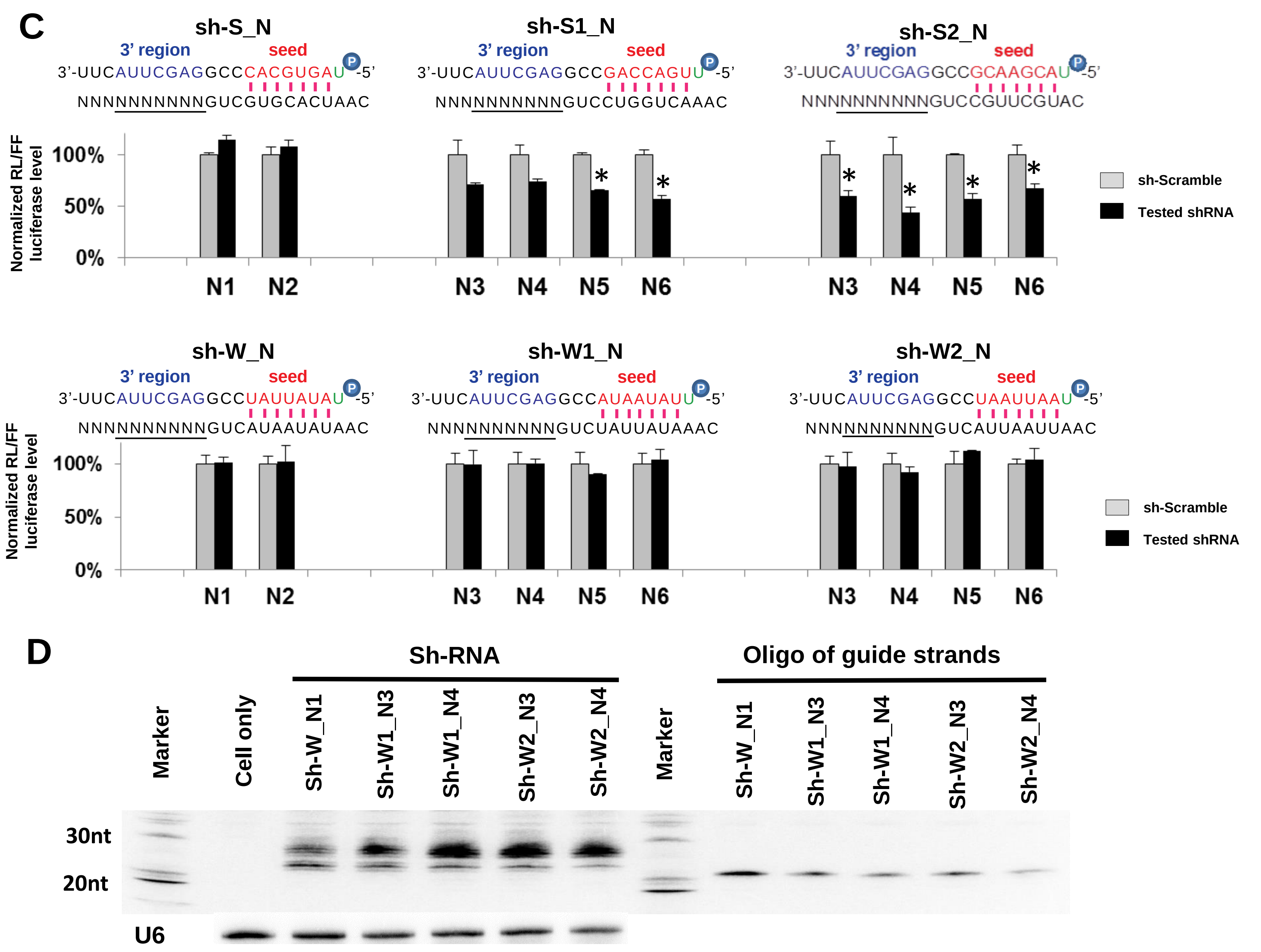
C
sh-S_N
3' region
seed
3'-UUCAUUCGAGGCCCACGUGAU-5'
NNNNNNNNNNGUCGUGCACUAAC
sh-S1_N
3'-UUCAUUCGAGGCCGACCAGUU-5'
NNNNNNNNNNGUCCUGGUCAAAC
sh-S2_N
3'-UUCAUUCGAGGCCGCAAGCAU-5'
NNNNNNNNNNGUCCGUUCGUAC
Normalized RL/FF luciferase level
100%
50%
0%
N1
N2
N3
N4
N5
N6
sh-Scramble
Tested shRNA
sh-W_N
3'-UUCAUUCGAGGCCUAUUAUAU-5'
NNNNNNNNNNGUCAUAAUAUAAC
sh-W1_N
3'-UUCAUUCGAGGCCAUAAUAUU-5'
NNNNNNNNNNGUCUAUUAUAAAC
sh-W2_N
3'-UUCAUUCGAGGCCUAAUUAAU-5'
NNNNNNNNNNGUCAUUAAUUAAC
D
Sh-RNA
Oligo of guide strands
Marker
Cell only
Sh-W_N1
Sh-W1_N3
Sh-W1_N4
Sh-W2_N3
Sh-W2_N4
30nt
20nt
U6

Figure S1 (C-D)

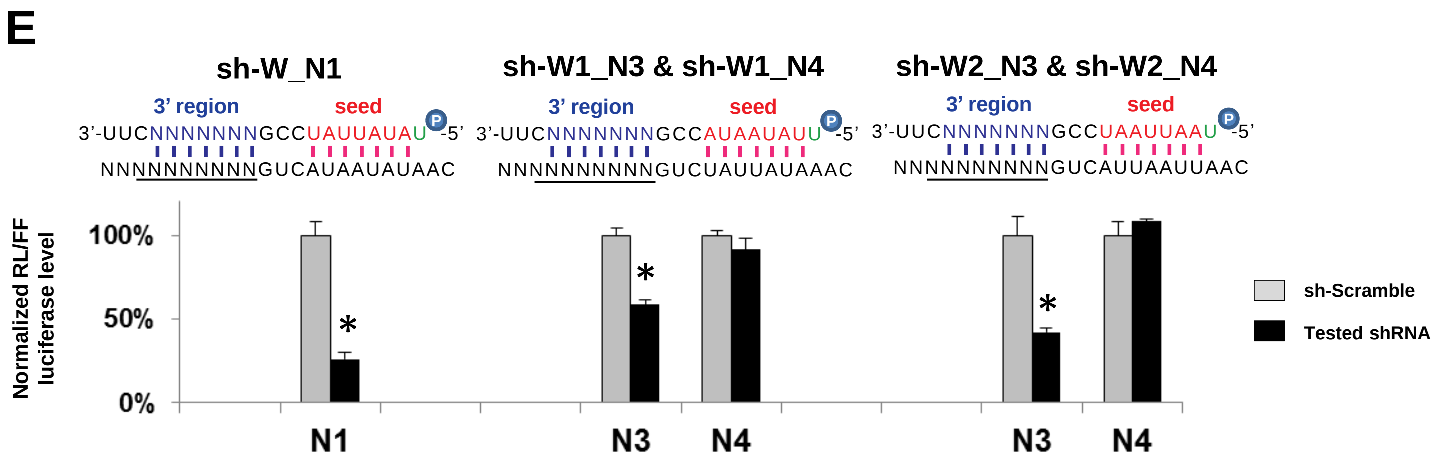

Supplementary Figure S1 Base Pairing Beyond the Seed region contributes to off-target effects. Related to Figure 1.

(A) On-target and off-target effects of sh-miR30-21 were measured by dual-luciferase reporter assay. PsiCHECK vector with one target site in the 3'UTR and DNA plasmids expressing sh-miR30-21 were co-transfected into HEK293 cells. shRNAs were named by symbols representing the seed and 3'region sequences, with a underline in between. S stands for GC-balanced motif; W stands for AU-rich motif; N stands for random sequences (Supplementary Table 1). As illustrated in the figure, perfectly matched targets was used to measure the on-target effect from guide strand and detect off-target effect from passenger strand. A seed-matched target was used to capture the guide strand mediated miRNA-like off-target effects. RL-luciferase activities were normalized with FF-luciferase, and the percentage of relative enzyme activity compared to the negative control (treated with sh-scramble) was plotted. Error bars represent the SD from two independent experiments, each performed in triplicate transfections. (B) Expression of shRNAs used in Fig1B was analyzed by Northern blotting 24 hr post-transfection in 293 cells. Extracted RNAs (10ug each) were run on 15% polyacrylamide 7 M urea denaturing gels. Guide strands were identified with a mixture of corresponding probes. Synthetic oligos (20 fmol each) with same sequences as guide strands were also loaded on the gel as controls for probe strength and hybridization efficiency. Endogenous U6 snRNA was detected as an internal control. (C) The experiment as described in Fig1B was repeated in MEF cells. (D) Expression of shRNAs used in Fig1C was analyzed by Northern blotting 24 hr post-transfection in HEK293 cells. The label is the same as described above. (E) The experiment as described in Fig1C was repeated in MEF cells. * p (t-test, two tailed) < 0.0001 compared with sh-scramble control treatment.

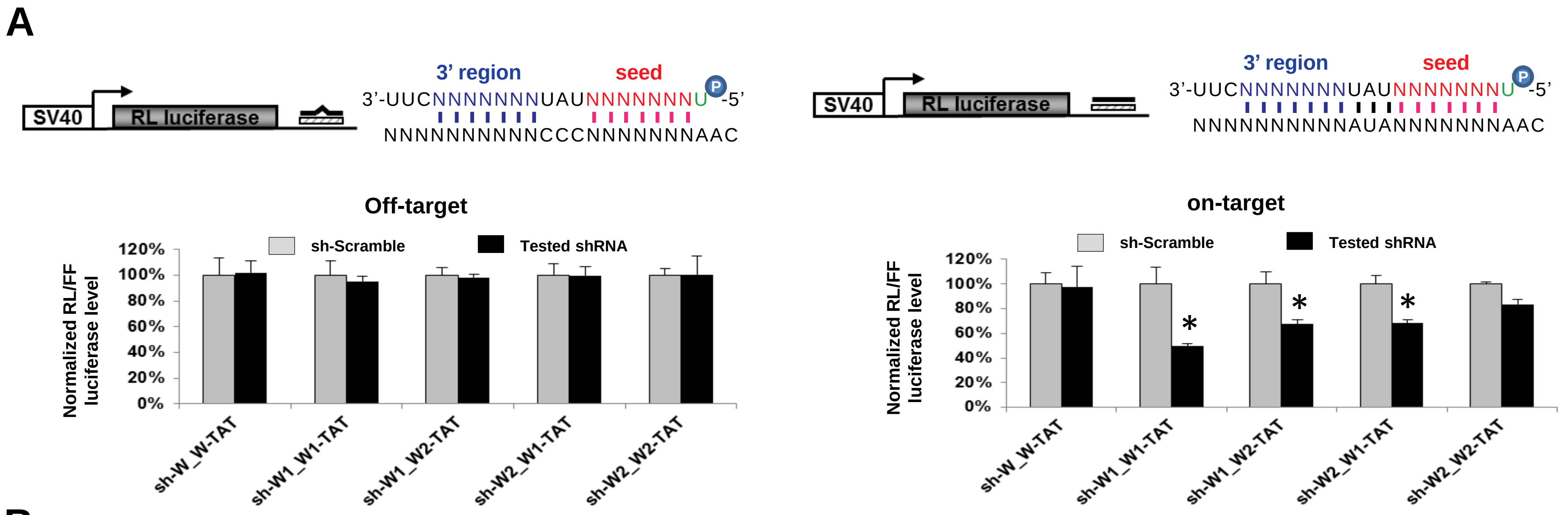
A
SV40
RL luciferase
3' region
seed
3'-UUCNNNNNNNUAUNNNNNNNU-5'
NNNNNNNNNNCCNNNNNNNAAC
P
3'-UUCNNNNNNNUAUNNNNNNNU-5'
NNNNNNNNNNAUANNNNNNNAAC
Off-target
on-target
sh-Scramble
Tested shRNA
Normalized RL/FF luciferase level
120%
100%
80%
60%
40%
20%
0%
sh-W_W-TAT
sh-W1_W1-TAT
sh-W1_W2-TAT
sh-W2_W1-TAT
sh-W2_W2-TAT
*

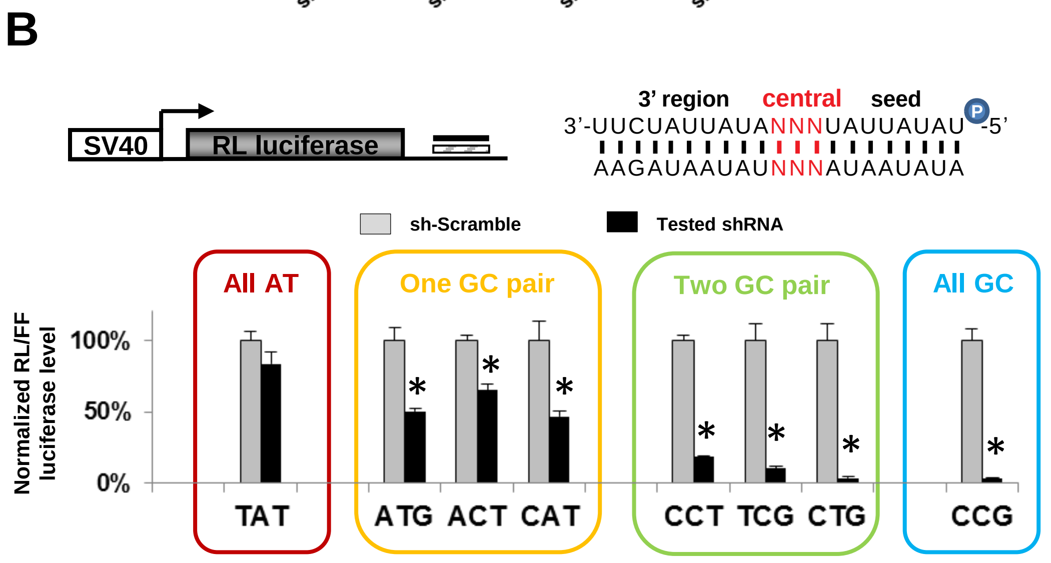
B
SV40
RL luciferase
3' region
central
seed
P
3'-UUCUAUUAUANNNUAUUAUAU-5'
AAGAUAAUAUNNNAUAAUAUA
sh-Scramble
Tested shRNA
All AT
One GC pair
Two GC pair
All GC
Normalized RL/FF luciferase level
100%
50%
0%
*
TAT
ATG
ACT
CAT
CCT
TCG
CTG
CCG

Figure S2 (A-B)

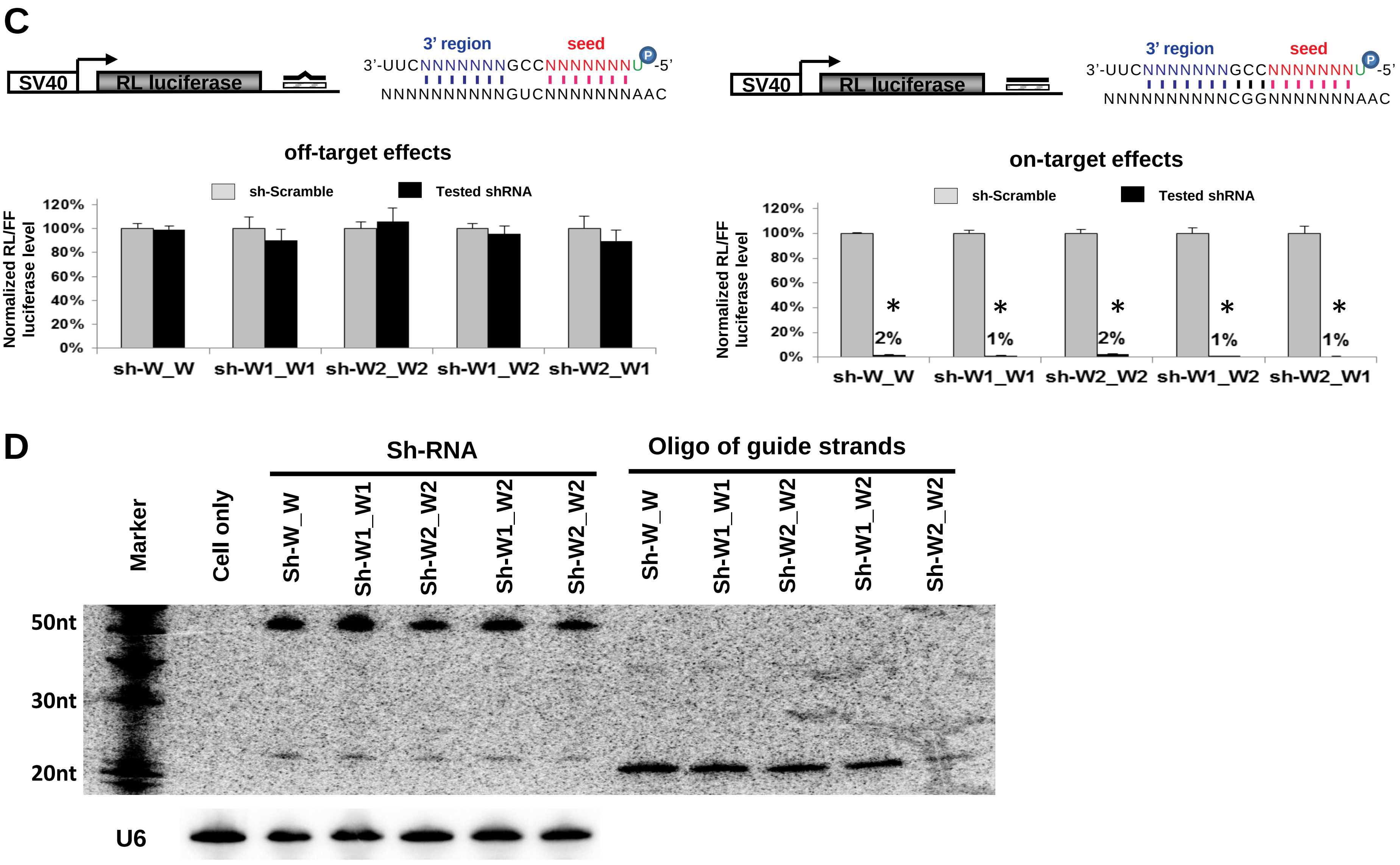
C
SV40
RL luciferase
3' region
seed
3'-UUCNNNNNNNGCCNNNNNNNU-5'
NNNNNNNNNNGUCNNNNNNNAAC
3'-UUCNNNNNNNGCCNNNNNNNU-5'
NNNNNNNNNNCGGNNNNNNNAAC
P
off-target effects
on-target effects
sh-Scramble
Tested shRNA
Normalized RL/FF luciferase level
120%
100%
80%
60%
40%
20%
0%
sh-W_W
sh-W1_W1
sh-W2_W2
sh-W1_W2
sh-W2_W1
*
2%
1%
2%
1%
1%
D
Sh-RNA
Oligo of guide strands
Marker
Cell only
Sh-W_W
Sh-W1_W1
Sh-W2_W2
Sh-W1_W2
Sh-W2_W2
Sh-W_W
Sh-W1_W1
Sh-W2_W2
Sh-W1_W2
Sh-W2_W2
50nt
30nt
20nt
U6

Figure S2 (C-D)

Supplementary Figure S2 AT-enrichment in both seed and 3’region is an optimal design to reduce miRNA-like off-target effect. Related to Figure 2.

(A) Extensive AU reduced the efficacies of both on-targeting and off-targeting. DNA plasmids expressing shRNAs with extensive AU sequences were co-transfected with psi-CHECK vector in HEK293 cells. As illustrated in the figure, a perfectly matched target was used to measure the on-target effect while a central-mismatched target was used to capture the miRNA-like off-target effects. Sequences used in the seed and 3’region were indicated in the shRNA name. Symbol before the underline represents the seed sequence. Results were plotted as described in Fig 2B. (B) The experiment as described in Fig 2A was repeated in MEF cells. (C) The experiment as described in Fig2B was repeated in MEF cells. (D) Expression of shRNAs used in Fig2B was analyzed by Northern blotting 24 hr post-transfection in HEK293 cells. Extracted RNAs (10ug each) were run on 15% polyacrylamide 7 M urea denaturing gels. Guide strands were identified with a mixture of corresponding probes. Synthetic oligos (20 fmol each) with same sequences as the guide strands being detected were also loaded on the gel as controls for probe strength and hybridization efficiency. Endogenous U6 snRNA was detected as an internal control. * p (t-test, two tailed) < 0.0001 compared with sh-scramble control treatment.

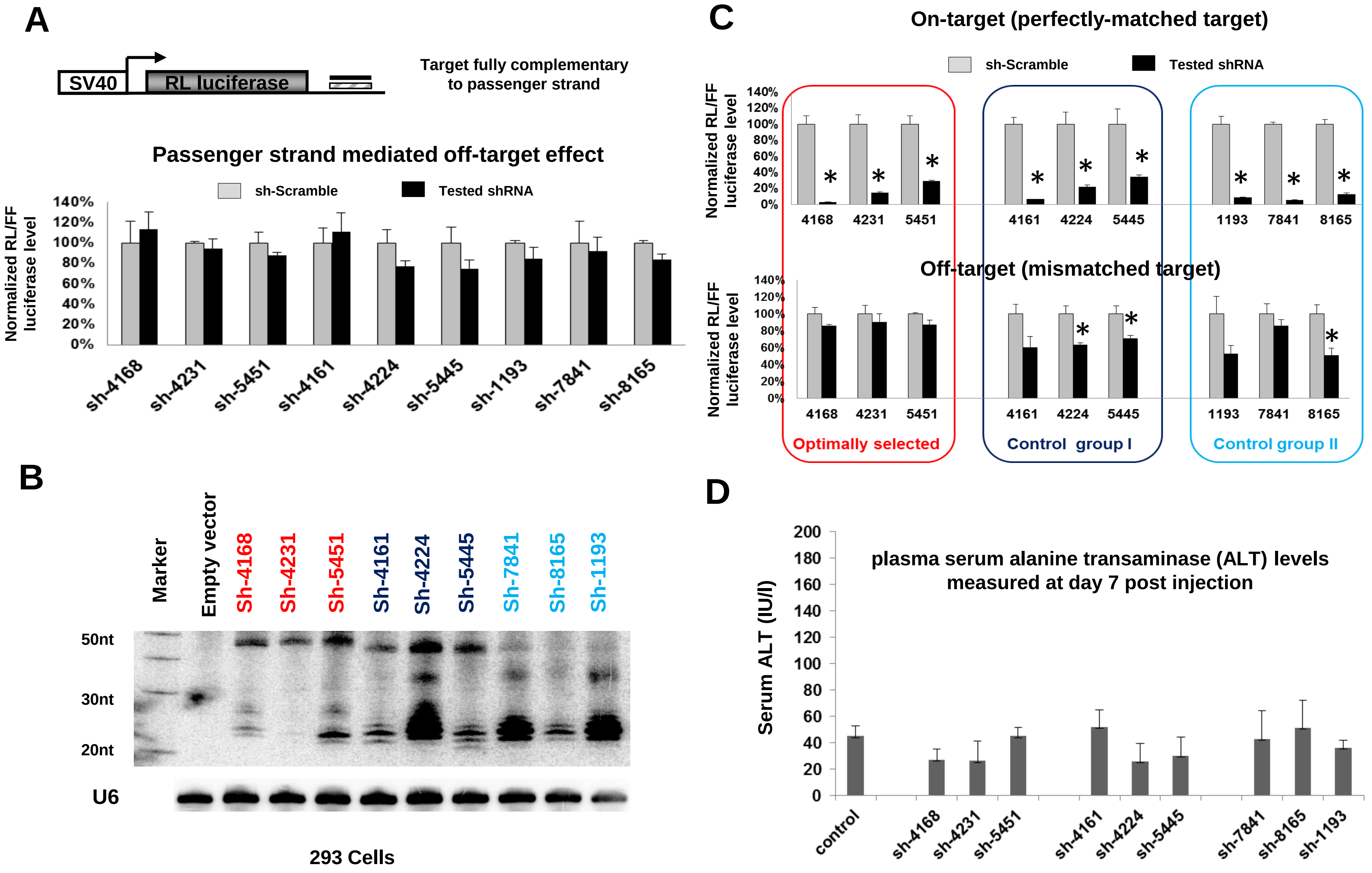
A
SV40
RL luciferase
Target fully complementary to passenger strand
Passenger strand mediated off-target effect
sh-Scramble
Tested shRNA
Normalized RL/FF luciferase level
140%
120%
100%
80%
60%
40%
20%
0%
sh-4168
sh-4231
sh-5451
sh-4161
sh-4224
sh-5445
sh-1193
sh-7841
sh-8165
B
Marker
Empty vector
Sh-4168
Sh-4231
Sh-5451
Sh-4161
Sh-4224
Sh-5445
Sh-7841
Sh-8165
Sh-1193
50nt
30nt
20nt
U6
293 Cells
C
On-target (perfectly-matched target)
sh-Scramble
Tested shRNA
Normalized RL/FF luciferase level
4168 4231 5451
4161 4224 5445
1193 7841 8165
Off-target (mismatched target)
Optimally selected
Control group I
Control group II
D
plasma serum alanine transaminase (ALT) levels measured at day 7 post injection
Serum ALT (IU/l)
200
180
160
140
120
100
80
60
40
20
0
control
sh-4168
sh-4231
sh-5451
sh-4161
sh-4224
sh-5445
sh-7841
sh-8165
sh-1193

Figure S3 (A-D)

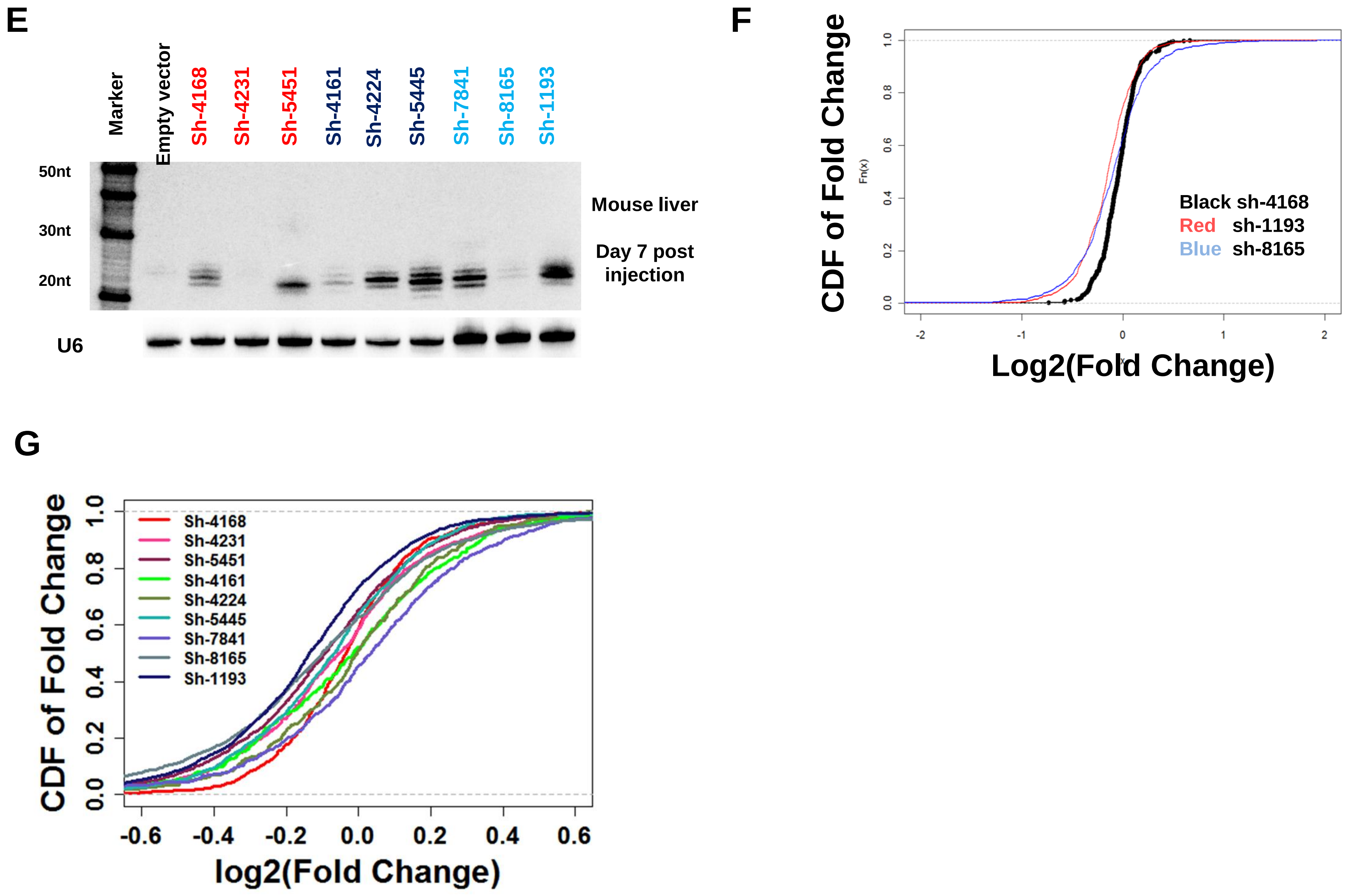
E
Marker
Empty vector
Sh-4168
Sh-4231
Sh-5451
Sh-4161
Sh-4224
Sh-5445
Sh-7841
Sh-8165
Sh-1193
50nt
30nt
20nt
U6
Mouse liver
Day 7 post injection
F
CDF of Fold Change
Fn(x)
Black sh-4168
Red sh-1193
Blue sh-8165
Log2(Fold Change)
G
CDF of Fold Change
Sh-4168
Sh-4231
Sh-5451
Sh-4161
Sh-4224
Sh-5445
Sh-7841
Sh-8165
Sh-1193
log2(Fold Change)

Figure S3 (E-G)

H

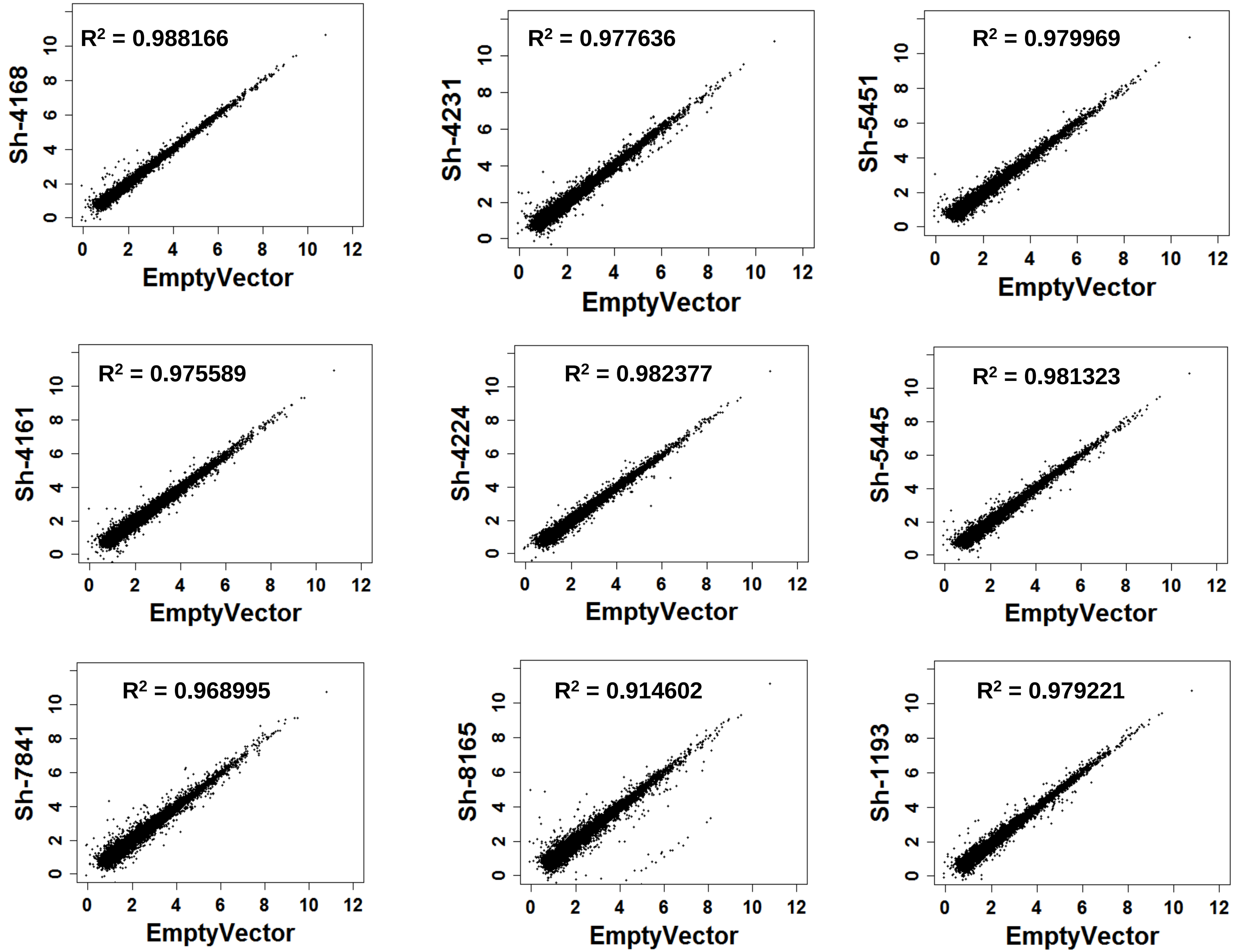

R2 = 0.988166
Sh-4168
EmptyVector
R2 = 0.977636
Sh-4231
EmptyVector
R2 = 0.979969
Sh-5451
EmptyVector
R2 = 0.975589
Sh-4161
EmptyVector
R2 = 0.982377
Sh-4224
EmptyVector
R2 = 0.981323
Sh-5445
EmptyVector
R2 = 0.968995
Sh-7841
EmptyVector
R2 = 0.914602
Sh-8165
EmptyVector
R2 = 0.979221
Sh-1193
EmptyVector

I

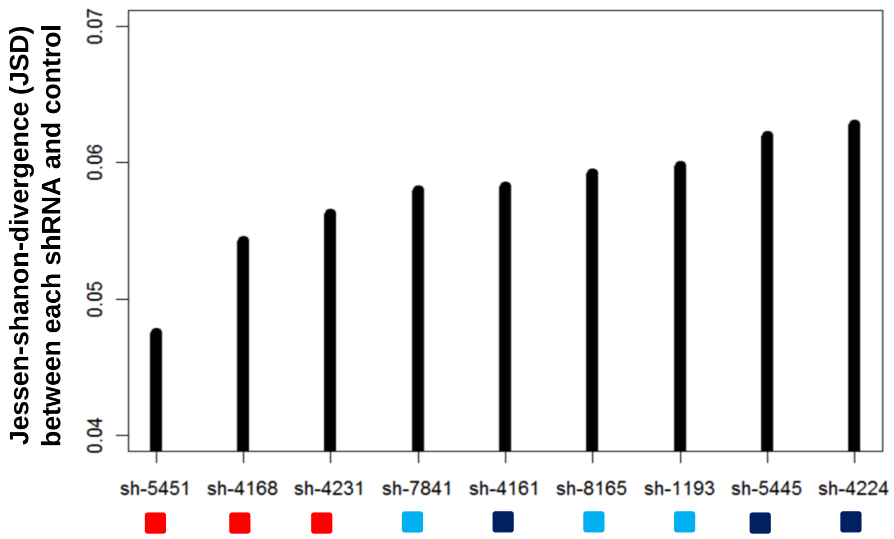

Supplementary Figure S3 Silencing efficacy and off-targeting of anti-HCV shRNAs. Related to Figure 3.

(A) PsiCHECK vectors with a target perfectly matched to the passenger strand in its 3'UTR were used to monitor the off-target effects originated the passenger strand of anti-HCV shRNAs. Result was plotted as described previously. Error bars represent the SD from two independent experiments, each performed in triplicate transfections. (B) Expression of anti-HCV shRNAs in HEK293 cells was analyzed by Northern blotting 24hr post-transfection (C) The experiment described in Fig 3B was repeated in MEF cells. * p (t-test, two tailed) < 0.0001 compared with sh-scramble control treatment. (D) Measurements of serum ALT levels in mice 7 days after injection. Each group contains three mice. Mean ALT value was plotted in figure with SD as error bar. (E) Expression of anti-HCV shRNAs in mouse liver were measured by Northern blot seven 7 days post-injection. (F) and (G) Liver RNAs were subject to RNA-Seq. Cumulative distributions of fold changes (CDF) in mRNA levels following shRNA treatments for messages containing the seed binding sites were shown here. (H) Scatter plots of gene expressions (based on FMPK value) between individual shRNA treated sample and control were shown in the figure. (I) Result of a biological repeat of experiment performed in Fig 3C. Liver mRNA expression of mice treated with anti-HCV shRNA were compared to that of mouse treated with empty vector. Jensen-Shannon Divergence (JSD) was calculated and shown in the figure.

A

Hannon/Elledge/Lowe design

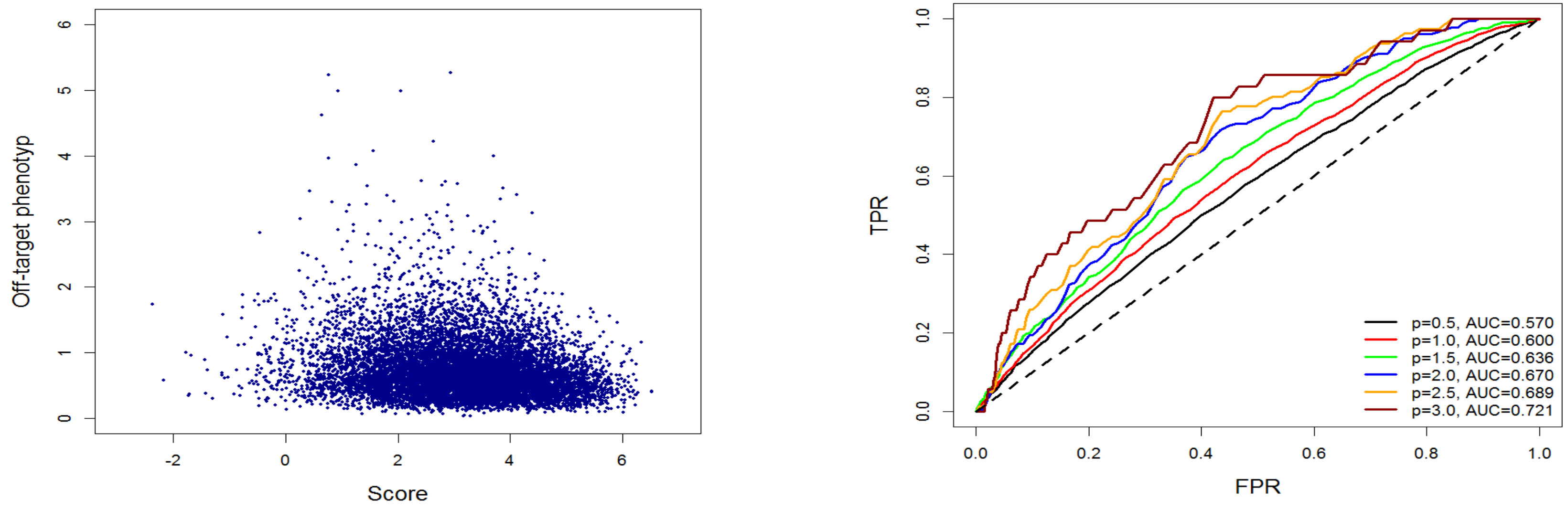

B

siDirect 2.0 design

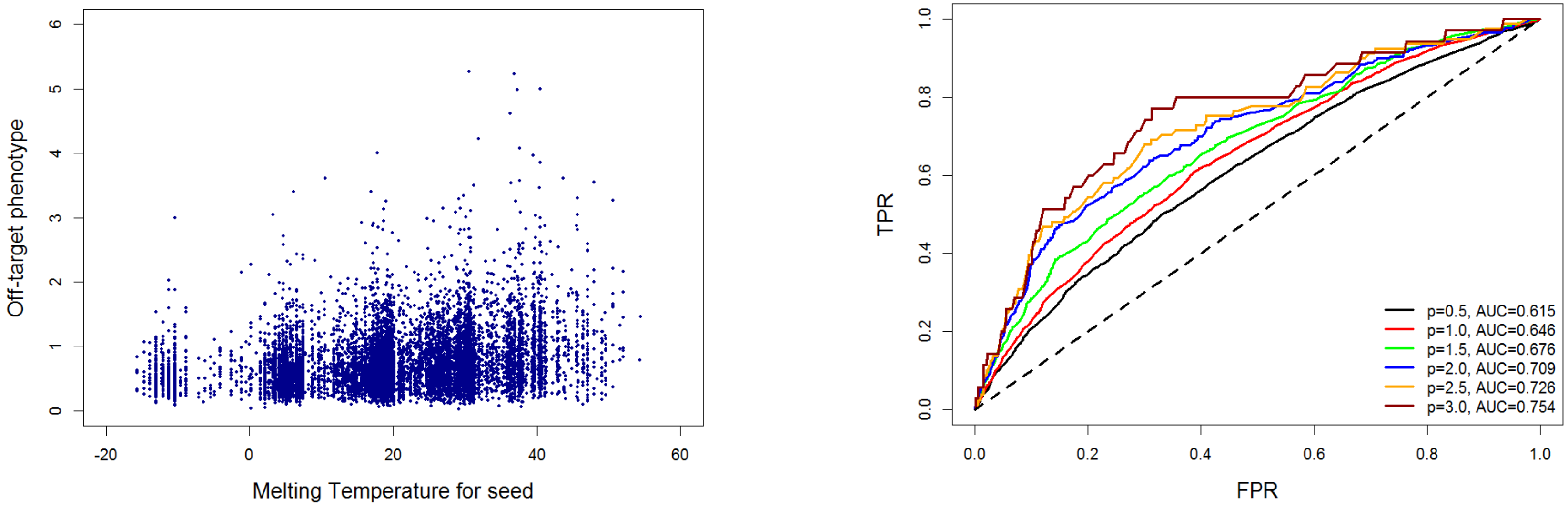

Supplementary Figure S4 Evaluation of the two established design schemes by large data set from shRNA library screen. Related to Figure 4.

Off-targeting phenotype index was plotted against OD score for over 10, 000 shRNAs. Based on the phenotype index, shRNAs were classified as strong off-target effect (positive) or no strong off-target effect (negative). Receiver operating characteristic (ROC) curve was created by plotting true positive rate (TPR) against false positive rate (FPR) at various threshold settings of OD score. Different value of phenotype index was used as cut-off (labeled as ρ in the figure) to distinguish positive (strong off-targeting) from negative (no strong off-target). Area under the curve (AUC) presenting the prediction power of the algorithm was calculated and indicated in the figure at various ρ values. (A) Hannon/Elledge/lowe scheme and (B) siDirect 2.0 Also see method for details.
