# Supplementary material for: Weak base pairing in both seed and 3′ regions reduces RNAi off-targets and enhances si/shRNA designs
Source: Nucleic Acids Res. 2014 Sep 30;42(19):12169–76. doi: 10.1093/nar/gku854 (PMC4231738; doi:10.1093/nar/gku854)
Supplement: SUPPLEMENTARY DATA [file supp_gku854_nar-00955-y-2014-File007.docx]

| Motif Name | Motif Sequences (5 to 3) | GC content |
| --- | --- | --- |
| N | GAGCTTA | 3/7 |
| N1 | AGTGCAC | 4/7 |
| N2 | ATATTAT | 0/7 |
| N3 | TGACCAG | 4/7 |
| N4 | ACGAACG | 4/7 |
| N5 | TATAATA | 0/7 |
| N6 | AATTAAT | 0/7 |
| W | ATATTAT | 0/7 |
| W1 | TATAATA | 0/7 |
| W2 | AATTAAT | 0/7 |
| S | AGTGCAC | 4/7 |
| S1 | TGACCAG | 4/7 |
| S2 | ACGAACG | 4/7 |

**Supplementary Table 1** Motif sequences used in this study

| Sh-W_N1-Sense | GATC GGTGCACTCGGATAATATA CT CCTGACCCA AG T ATATTAT CCG AGTGCAC C TTTTT GTAC |
| --- | --- |
| Sh-W_N1-Antisense | AAAAAGGTGCACTCGGATAATATACTTGGGTCAGGAGTATATTATCCGAGTGCACC |
| Sh-W1_N3-Sense | GATC GCTGGTCACGGTATTATAACT CCTGACCCA AG T TATAATA CCG TGACCAG C TTTTT GTAC |
| Sh-W1_N3-Antisense | AAAAAGCTGGTCACGGTATTATAACTTGGGTCAGGAGTTATAATACCGTGACCAGC |
| Sh-W1_N4-Sense | GATC GCGTTCGTCGGTATTATAA CT CCTGACCCA AG T TATAATA CCG ACGAACG C TTTTT GTAC |
| Sh-W1_N4-Antisense | AAAAAGCGTTCGTCGGTATTATAACTTGGGTCAGGAGTTATAATACCGACGAACGC |
| Sh-W2_N3-Sense | GATC GCTGGTCACGGATTAATTA CT CCTGACCCA AG T AATTAAT CCG TGACCAG C TTTTT GTAC |
| Sh-W2_N3-Antisense | AAAAAGCTGGTCACGGATTAATTACTTGGGTCAGGAGTAATTAATCCGTGACCAGC |
| Sh-W2_N4-Sense | GATC GCGTTCGTCGGATTAATTACT CCTGACCCA AG T AATTAAT CCG ACGAACG C TTTTT GTAC |
| Sh-W2_N4-Antisense | AAAAAGCGTTCGTCGGATTAATTACTTGGGTCAGGAGTAATTAATCCGACGAACGC |
| Sh-S_N-Sense | GATC GTAAGCTCCGGGTGCACTA CT CCTGACCCA AG T AGTGCAC CCG gagctta C TTTTT GTAC |
| Sh-S_N-Antisense | AAAAAGTAAGCTCCGGGTGCACTACTTGGGTCAGGAGTAGTGCACCCGGAGCTTAC |
| Sh-S1_N-Sense | GATC GTAAGCTCCGGCTGGTCAA CT CCTGACCCA AG T TGACCAG CCG gagctta C TTTTT GTAC |
| Sh-S1_N-Antisense | AAAAAGTAAGCTCCGGCTGGTCAACTTGGGTCAGGAGTTGACCAGCCGGAGCTTAC |
| Sh-S2_N-Sense | GATC GTAAGCTCCGGCGTTCGTA CT CCTGACCCA AG T ACGAACG CCG gagctta C TTTTT GTAC |
| Sh-S2_N-Antisense | AAAAAGTAAGCTCCGGCGTTCGTACTTGGGTCAGGAGTACGAACGCCGGAGCTTAC |
| Sh-W_N-Sense | GATC GTAAGCTCCGGATAATATA CT CCTGACCCA AG T ATATTAT CCG gagctta C TTTTT GTAC |
| Sh-W_N-Antisense | AAAAAGTAAGCTCCGGATAATATACTTGGGTCAGGAGTATATTATCCGGAGCTTAC |
| Sh-W1_N-Sense | GATC GTAAGCTCCGGTATTATAA CT CCTGACCCA AG T TATAATA CCG gagctta C TTTTT GTAC |
| Sh-W1_N-Antisense | AAAAAGTAAGCTCCGGTATTATAACTTGGGTCAGGAGTTATAATACCGGAGCTTAC |
| Sh-W2_N-Sense | GATC GTAAGCTCCGGATTAATTA CT CCTGACCCA AG T AATTAAT CCG gagctta C TTTTT GTAC |
| Sh-W2_N-Antisense | AAAAAGTAAGCTCCGGATTAATTACTTGGGTCAGGAGTAATTAATCCGGAGCTTAC |
| Sh-W_W_TAT-Sense | GATC GATAATATATA ATAATATA CT CCTGACCCA AG T ATATTAT TATATATTAT C TTTTT GTAC |
| Sh-W_W_TAT-Antisense | AAAAAGATAATATATAATAATATACTTGGGTCAGGAGTATATTATTATATATTATC |
| Sh-W_W_ATG-Sense | GATC GATAATATCAT ATAATATA CT CCTGACCCA AG T ATATTAT ATGATATTAT C TTTTT GTAC |
| Sh-W_W_ATG-Antisense | AAAAAGATAATATCATATAATATACTTGGGTCAGGAGTATATTATATGATATTATC |
| Sh-W_W_ACT-Sense | GATC GATAATATAGT ATAATATA CT CCTGACCCA AG T ATATTAT ACT ATATTAT C TTTTT GTAC |
| Sh-W_W_ACT-Antisense | AAAAAGATAATATAGTATAATATACTTGGGTCAGGAGTATATTATACTATATTATC |
| Sh-W_W_CAT-Sense | GATC GATAATATATG ATAATATA CT CCTGACCCA AG T ATATTAT CAT ATATTAT C TTTTT GTAC |
| Sh-W_W_CAT-Antisense | AAAAAGATAATATATGATAATATACTTGGGTCAGGAGTATATTATCATATATTATC |
| Sh-W_W_CCT-Sense | GATC GATAATATAGGATAATATA CT CCTGACCCA AG T ATATTAT CCT ATATTAT C TTTTT GTAC |
| Sh-W_W_CCT-Antisense | AAAAAGATAATATAGGATAATATACTTGGGTCAGGAGTATATTATCCTATATTATC |
| Sh-W_W_TCG-Sense | GATC GATAATATCGAATAATATA CT CCTGACCCA AG T ATATTAT TCGATATTAT C TTTTT GTAC |
| Sh-W_W_TCG-Antisense | AAAAAGATAATATCGAATAATATACTTGGGTCAGGAGTATATTATTCGATATTATC |
| Sh-W_W_CTG-Sense | GATC GATAATATCAGATAATATA CT CCTGACCCA AG T ATATTAT CTG ATATTAT C TTTTT GTAC |
| Sh-W_W_CTG-Antisense | AAAAAGATAATATCAGATAATATACTTGGGTCAGGAGTATATTATCTGATATTATC |
| Sh-W_W_CCG-Sense | GATC GATAATATCGGATAATATA CT CCTGACCCA AG T ATATTAT CCG ATATTAT C TTTTT GTAC |
| Sh-W_W_CCG-Antisense | AAAAAGATAATATCGGATAATATACTTGGGTCAGGAGTATATTATCCGATATTATC |
| Sh-W_W-Sense | GATC GATAATATCGGATAATATA CT CCTGACCCA AG T ATATTAT CCG ATATTAT C TTTTT GTAC |
| Sh-W_W-Antisense | AAAAAGATAATATCGGATAATATACTTGGGTCAGGAGTATATTATCCGATATTATC |
| Sh-W1_W1-Sense | GATC GTATTATACGGTATTATAA CT CCTGACCCA AG T TATAATA CCG TATAATA C TTTTT GTAC |
| Sh-W1_W1-Antisense | AAAAAGTATTATACGGTATTATAACTTGGGTCAGGAGTTATAATACCGTATAATAC |
| Sh-W2_W2-Sense | GATC GATTAATTCGGATTAATTA CT CCTGACCCA AG T AATTAAT CCG AATTAAT C TTTTT GTAC |
| Sh-W2_W2-Antisense | AAAAAGATTAATTCGGATTAATTACTTGGGTCAGGAGTAATTAATCCGAATTAATC |
| Sh-W1_W2-Sense | GATC GATTAATTCGGTATTATAA CT CCTGACCCA AG T TATAATA CCG AATTAAT C TTTTT GTAC |
| Sh-W1_W2-Antisense | AAAAAGATTAATTCGGTATTATAACTTGGGTCAGGAGTTATAATACCGAATTAATC |
| Sh-W2_W1-Sense | GATC GTATTATACGGATTAATTA CT CCTGACCCA AG T AATTAAT CCG TATAATA C TTTTT GTAC |
| Sh-W2_W1-Antisense | AAAAAGCTGGTCACGGATTAATTACTTGGGTCAGGAGTAATTAATCCGTGACCAGC |
| PsiCheck-W_N1-Sense | TCGAGGAGGTGCACTGTCATAATATAA |
| PsiCheck-W_N1-Antisense | CTAGTTATATTATGACAGTGCACCTCC |
| PsiCheck-W1_N3-Sense | TCGAGAAGCTGGTCAGTCTATTATAAA |
| PsiCheck-W1_N3-Antisense | CTAGTTTATAATAGACTGACCAGCTTC |
| PsiCheck-W1_N4-Sense | TCGAGAA GCGTTCGTGTCTATTATAAA |
| PsiCheck-W1_N4-Antisense | CTAGTTTATAATAGACACGAACGCTTC |
| PsiCheck-W2_N3-Sense | TCGAGAAGCTGGTCAGTCATTAATTAA |
| PsiCheck-W2_N3-Antisense | CTAGTTAATTAATGACTGACCAGCTTC |
| PsiCheck-W2_N4-Sense | TCGAGAA GCGTTCGTGTCATTAATTAA |
| PsiCheck-W2_N4-Antisense | CTAGTTAATTAATGACACGAACGCTTC |
| PsiCheck-W_W-off_target -Sense | TCGAGGAGATAATATGTCATAATATAA |
| PsiCheck-W_W-off_target -Antisense | CTAGTTATATTATGACATATTATCTCC |
| PsiCheck-W1_W1-off_target -Sense | TCGAGAA GTATTATAGTCTATTATAAA |
| PsiCheck-W1_W1-off_target- Antisense | CTAGTTTATAATAGACTATAATACTTC |
| PsiCheck-W2_W2-off_target -Sense | TCGAGAA GATTAATTGTCATTAATTAA |
| PsiCheck-W2_W2-off_target -Antisense | CTAGTTAATTAATGACAATTAATCTTC |
| PsiCheck-W1_W2-off_target -Sense | TCGAG AA GATTAATTGTCTATTATAA A |
| PsiCheck-W1_W2-off_target -Antisense | CTAGTTTATAATAGACAATTAATCTTC |
| PsiCheck-W2_W1-off_target -Sense | TCGAGAA GTATTATAGTCATTAATTAA |
| PsiCheck-W2_W1-off_target -Antisense | CTAGTTAATTAATGACTATAATACTTC |
| Sh-4168-Sense | GATC G CATCAATCCCAACATTAA CTCCTGACCCAAG T TAATGTTGGGATTGATG C TTTTT GTAC |
| Sh-4168-Antisense | AAAAAGCATCAATCCCAACATTAACTTGGGTCAGGAGTTAATGTTGGGATTGATGC |
| Sh-4231-Sense | GATC G CACATATGGCAAATTTCA CTCCTGACCCAAG T GAAATTTGCCATATGTG C TTTTT GTAC |
| Sh-4231-Antisense | AAAAAGCACATATGGCAAATTTCACTTGGGTCAGGAGTGAAATTTGCCATATGTGC |
| Sh-5451-Sense | GATC G TGTATGAGGCTTTTGATA CTCCTGACCCAAG T ATCAAAAGCCTCATACA C TTTTT GTAC |
| Sh-5451-Antisense | AAAAAGTGTATGAGGCTTTTGATACTTGGGTCAGGAGTATCAAAAGCCTCATACAC |
| Sh-4161-Sense | GATC G CACATGGCATCAATCCCA CTCCTGACCCAAG T GGGATTGATGCCATGTG C TTTTT GTAC |
| Sh-4161-Antisense | AAAAAGCACATGGCATCAATCCCACTTGGGTCAGGAGTGGGATTGATGCCATGTGC |
| Sh-4224-Sense | GATC G CGTACTCCACATATGGCA CTCCTGACCCAAG T GCCATATGTGGAGTACG C TTTTT GTAC |
| Sh-4224-Antisense | AAAAAGCGTACTCCACATATGGCACTTGGGTCAGGAGTGCCATATGTGGAGTACGC |
| Sh-5445-Sense | GATC G AGGTCCTGTATGAGGCTA CTCCTGACCCAAG T AGCCTCATACAGGACCT C TTTTT GTAC |
| Sh-5445-Antisense | AAAAAGAGGTCCTGTATGAGGCTACTTGGGTCAGGAGTAGCCTCATACAGGACCTC |
| Sh-7841-Sense | GATC G CTCGACGCCCATTATGAA CTCCTGACCCAAG T TCATAATGGGCGTCGAG C TTTTT GTAC |
| Sh-7841-Antisense | AAAAAGCTCGACGCCCATTATGAACTTGGGTCAGGAGTTCATAATGGGCGTCGAGC |
| Sh-8165-Sense | GATC G CGGGTCTGCGAGAAAATA CTCCTGACCCAAG TATTTTCTCGCAGACCCG C TTTTT GTAC |
| Sh-8165-Antisense | AAAAAGCGGGTCTGCGAGAAAATACTTGGGTCAGGAGTATTTTCTCGCAGACCCGC |
| Sh-1193-Sense | GATC G CTCGCAGCCCAAATGTTA CTCCTGACCCAAG T AACATTTGGGCTGCGAG C TTTTT GTAC |
| Sh-1193-Antisense | AAAAAGCTCGCAGCCCAAATGTTACTTGGGTCAGGAGTAACATTTGGGCTGCGAGC |
| PsiCheck-4168-shRNA-on-target-Sense | TCGAG TGGCATCAATCCCAACATTAG A |
| PsiCheck-4168-shRNA-on-target-Antisense | CTAGT CTAATGTTGGGATTGATGCCA C |
| PsiCheck-4168-shRNA-off-target-Sense | TCGAG TGGCATCAATGGGAACATTAG A |
| PsiCheck-4231-shRNA-off-target-Antisense | CTAGT CTAATGTTCCCATTGATGCCA C |
| PsiCheck-4231-shRNA-on-target-Sense | TCGAG CT C CACATATGGCAAATTTC T A |
| PsiCheck-4231-shRNA-on-target-Antisense | CTAGT AGAAATTTGCCATATGTGGAG C |
| PsiCheck-4231-shRNA-off-target-Sense | TCGAG CT C CACATATCCGAAATTTC T A |
| PsiCheck-4231-shRNA-off-target-Antisense | CTAGT AGAAATTTCGGATATGTGGAG C |
| PsiCheck-5451-shRNA-on-target-Sense | TCGAG TC C TGTATGAGGCTTTTGAT GA |
| PsiCheck-5451-shRNA-on-target-Antisense | CTAGT CATCAAAAGCCTCATACAGGA C |
| PsiCheck-5451-shRNA-off-target-Sense | TCGAG TC C TGTATGACCGTTTTGAT GA |
| PsiCheck-5451-shRNA-off-target-Antisense | CTAGT CATCAAAACGGTCATACAGGA C |
| PsiCheck-4161-shRNA-on-target-Sense | TCGAG AGG CACATGGCATCAATCCC AA |
| PsiCheck-4161-shRNA-on-target-Antisense | CTAGT TGGGATTGATGCCATGTGCCT C |
| PsiCheck-4161-shRNA-off-target-Sense | TCGAG AGG CACATGGGTACAATCCC AA |
| PsiCheck-4161-shRNA-off-target-Antisense | CTAGT TGGGATTGTACCCATGTGCCT C |
| PsiCheck-4224-shRNA-on-target-Sense | TCGAG TCACGTACTCCACATATGGCAA |
| PsiCheck-4224-shRNA-on-target-Antisense | CTAGT TGCCATATGTGGAGTACGTGA C |
| PsiCheck-4224-shRNA-off-target-Sense | TCGAG TCACGTACTCGTGATATGGCAA |
| PsiCheck-4224-shRNA-off-target-Antisense | CTAGT TGCCATATCACGAGTACGTGA C |
| PsiCheck-5445-shRNA-on-target-Sense | TCGAG AGGAGGTCCTGTATGAGGCTTA |
| PsiCheck-5445-shRNA-on-target-Antisense | CTAGT AAGCCTCATACAGGACCTCCT C |
| PsiCheck-5445-shRNA-off-target-Sense | TCGAG AGGAGGTCCTCATTGAGGCTTA |
| PsiCheck-5445-shRNA-off-target-Antisense | CTAGT AAGCCTCAATGAGGACCTCCT C |
| PsiCheck-1193-shRNA-on-target-Sense | TCGAG ATGCTCGCAGCCCAAATGTTCA |
| PsiCheck-1193-shRNA-on-target-Antisense | CTAGT GAACATTTGGGCTGCGAGCAT C |
| PsiCheck-1193-shRNA-off-target-Sense | TCGAG ATGCTCGCAGGGGAAATGTTCA |
| PsiCheck-1193-shRNA-off-target-Antisense | CTAGT GAACATTTCCCCTGCGAGCAT C |
| PsiCheck-7841-shRNA-on-target-Sense | TCGAG GTGCTCGACGCCCATTATGACA |
| PsiCheck-7841-shRNA-on-target-Antisense | CTAGT GTCATAATGGGCGTCGAGCAC C |
| PsiCheck-7841-shRNA-off-target-Sense | TCGAG GTGCTCGACGGGGATTATGACA |
| PsiCheck-7841-shRNA-off-target-Antisense | CTAGT GTCATAATCCCCGTCGAGCAC C |
| PsiCheck-8165-shRNA-on-target-Sense | TCGAG GTCCGGGTCTGCGAGAAAATGA |
| PsiCheck-8165-shRNA-on-target-Antisense | CTAGT CATTTTCTCGCAGACCCGGAC C |
| PsiCheck-8165-shRNA-off-target-Sense | CTAGT CATTTTCTCGCAGACCCGGAC C |
| PsiCheck-8165-shRNA-off-target-Antisense | CTAGT CATTTTCTGCGAGACCCGGAC C |
| Probe detecting guide strand of sh-S-N | GTAAGCTCCGGGTGCACTA |
| Probe detecting guide strand of sh-S1-N | GTAAGCTCCGGCTGGTCAA |
| Probe detecting guide strand of sh-S2-N | GTAAGCTCCGGCGTTCGTA |
| Probe detecting guide strand of sh-W-N | GTAAGCTCCGGATAATATA |
| Probe detecting guide strand of sh-W1-N | GTAAGCTCCGGTATTATAA |
| Probe detecting guide strand of sh-W2-N | GTAAGCTCCGGATTAATTA |
| Probe detecting guide strand of sh-W-N1 | GGTGCACTCGGATAATATA |
| Probe detecting guide strand of sh-W1-N3 | GCTGGTCACGGTATTATAA |
| Probe detecting guide strand of sh-W1-N4 | GCGTTCGTCGGTATTATAA |
| Probe detecting guide strand of sh-W2-N3 | GCTGGTCACGGATTAATTA |
| Probe detecting guide strand of sh-W2-N4 | GCGTTCGTCGGATTAATTA |
| Probe detecting guide strand of sh-W_W | GATAATATCGGATAATATA |
| Probe detecting guide strand of sh-W1_W1 | GTATTATACGGTATTATAA |
| Probe detecting guide strand of sh-W2_W2 | GATTAATTCGGATTAATTA |
| Probe detecting guide strand of sh-W1_W2 | GATTAATTCGGTATTATAA |
| Probe detecting guide strand of sh-W2_W1 | GTATTATACGGATTAATTA |
| Probe detecting guide strand of sh-4168 | G CATCAATCCCAACATTA A CT |
| Probe detecting guide strand of sh-4231 | G CACATATGGCAAATTTC A CT |
| Probe detecting guide strand of sh-5451 | G TGTATGAGGCTTTTGAT A CT |
| Probe detecting guide strand of sh-4161 | G CACATGGCATCAATCCC A CT |
| Probe detecting guide strand of sh-4224 | G CGTACTCCACATATGGC A CT |
| Probe detecting guide strand of sh-5445 | G AGGTCCTGTATGAGGCT A CT |
| Probe detecting guide strand of sh-1193 | G CTCGCAGCCCAAATGTT A CT |
| Probe detecting guide strand of sh-7841 | G CTCGACGCCCATTATGA A CT |
| Probe detecting guide strand of sh-8165 | G CGGGTCTGCGAGAAAAT A CT |
| Probe detecting U6 | gccatgctaatcttctctgtat |

**Supplementary Table 2** Oligo sequences used in this study


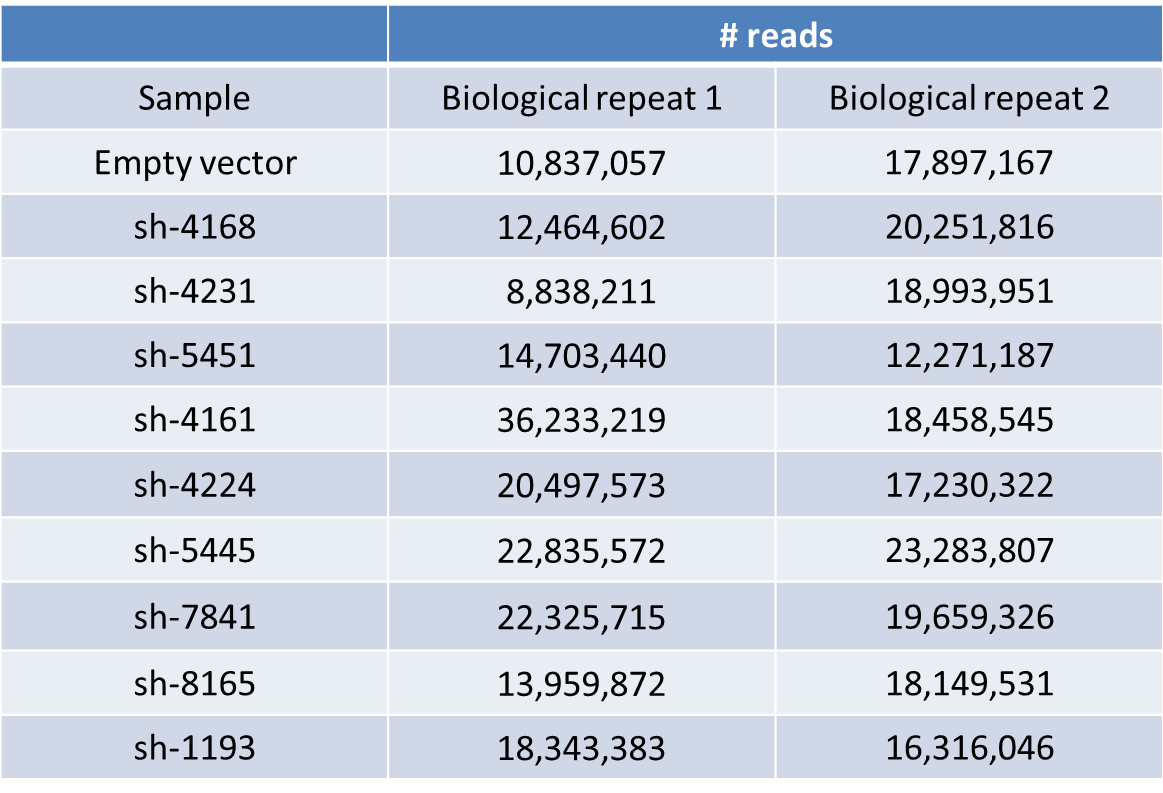


**Supplementary Table 3** Library size of RNA-Seq
