# Supplementary material for: Weak base pairing in both seed and 3′ regions reduces RNAi off-targets and enhances si/shRNA designs
Source: Nucleic Acids Res. 2014 Sep 30;42(19):12169–76. doi: 10.1093/nar/gku854 (PMC4231738; doi:10.1093/nar/gku854)
Supplement: SUPPLEMENTARY DATA [file supp_gku854_nar-00955-y-2014-File008.docx]

**Supplementary Table 4** Target sequences of control shRNAs and their off-target phenotype indexes

**Target site Sequence** **Off-target phenotype index**

(Complementary to the G-strand)

AAAAAATTCTATAGTAACTAAC 0.321511165649

AAAACACAAGCTGAAATAAATT 0.218480978415

AAAACACAGATTGCGTATGCAT 0.252705463673

AAAACATAATCCCTTTAGCCAG 0.66940529137

AAAACATGATCTCTGAAATATT 0.442891633079

AAAAGATACTATGTAACAGATC 0.337733908728

AAAAGATCATGTCGGGGTGAAC 0.885212614126

AAACCAGGATGTCATGCTTCTA 1.25261852286

AAACCTTGATCTCGACGAGAAA 0.543798025625

AAAGAAGGCGGTGCTTACTGAG 0.59895721131

AAAGCACAGAGGCCGGGTTGAG 0.710598277458

AAAGGAGTCTCTACTACATAAA 0.406617150086

AAAGGATGCACTCGGTATTAAG 0.331759661467

AAAGTACTTGCTCAAGTGGGAG 0.448244109344

AAATAATCAGTACATACTAAAC 0.592823788126

AAATCACAATCCGATCTGCAAT 0.445699160263

AAATCCCAGGTACATACCAGAC 0.557358012403

AAATGAGTACATCCATTCGTAG 0.154413573312

AAATGTGCGCCTCGATACAGAT 0.415108660726

AACAAAGTAGGCCCTCTCCGTG 1.59646805889

AACAAATAGGAGCATTTCTTAT 0.291462010281

AACAAATTAACCGCTAAAGAAA 0.299521817226

AACAACCAATGCTGTTAGAAAT 0.244745459547

AACAACCCACGTGAGAGACAAT 0.650968605132

AACAACGCGGGTGATCGAGAAC 1.29810655845

AACAATCAATGGGCTAATACAA 0.385888368242

AACAATCACAATGTACATGAAT 0.712067052743

AACAATCCATGTGATCTGTAAA 0.241528358591

AACAATCCATGTTTTGGCGAAC 0.292007405244

AACAATGAGCGTCAACCTGGAA 0.47271498496

AACAATGTATCTGGTTGCAAAA 0.590867344186

AACAATTAATGGGCTTTAAAAA 0.296428147281

AACACAAAATTTAAACCGGCTA 0.462983745601

AACACAAACAGTGATCGTTGAT 0.24414905092

AACACACCACCTGCTAGCTGAG 1.16865993159

AACACACGATATGATGCTGTAA 0.403985399416

AACACACTATGACATCGACAAG 0.331201969321

AACACACTGCTTGCTGCCGGAG 0.144237482308

AACACAGAAACACCATAATAAC 0.534789935943

AACACAGACTGTCCTGGCGATG 0.386459086112

AACACAGAGTGTCCTTCCCAAC 0.308366172397

AACACAGCACCTGATGGATAAA 0.142594036682

AACACAGCACTTCTCCAGTTAC 0.300117029732

AACACAGCGGATGTTTTCTAAT 0.45956089773

AACACAGGAAATGGTGTCTATA 0.590774288922

AACACAGTGGCTGATGGCCAAC 0.792680696214

AACACATACTCTGGTATCTCAA 0.43594192562

AACACATCATGTCACTGTTTAC 0.878650990458

AACACATGATATGTTCGAAGTA 0.376573815539

AACACATGATTTGCTTGATCTT 0.462421581418

AACACATTTTGTGCTGAAGAAA 0.616073796642

AACACCCAATTTGTTGGAATAA 0.617148050681

AACACCCGATCTTCGCCCATAA 0.915758815554

AACACCCTCTGTTGACAAAGAT 0.389164706074

AACACCGAAACTGAGTTCAGTG 0.536023270026

AACACCGAGGCCGATCAAGGAG 0.837617016243

AACACCGGAAGGCACTGAGAAT 0.809570561579

AACACCGGCTCTTAGTACACAT 0.445694231229

AACACCTCATGTGATTCGACAG 0.624275276369

AACACCTCCCCGGTTAGATGAA 0.884878223774

AACACGCTATCTCATACATAAT 0.391793554685

AACACGCTATGTTGTTCATTAT 0.527652294137

AACACGTATTCCTGCGGGGTAT 0.460783236953

AACACGTGATGTCCACAAAAAG 0.589425379822

AACACTCAATGTCCTTGGACTG 0.900522939289

AACACTCAGGGTCCAACAGCAT 0.590563392481

AACACTCCTACTGGCCGACTAT 0.868499470679

AACACTGAACCTTGACGGCAAG 0.93361959173

AACACTGCACCTGATCCCGTAG 1.1716836642

AACACTGCATTGCAAAAGTGTC 0.393232622043

AACACTGGAGATGTTGCATGAG 0.548892420738

AACACTGGTGGTTCATCCGGAG 0.785857857479

AACACTGTAGGTCAGAGCGAAG 0.267128369796

AACACTGTATGTCTAAATCAAT 0.459505205394

AACACTGTGACTCGTGCGTATG 0.648447603751

AACACTTAAAGTCCAACTCTTC 0.403571256723

AACACTTACTCTGCACATATAA 0.439971338493

AACACTTGAACTGCTCACGGAG 0.517430902771

AACACTTGAGGTTAATAGCAAA 0.138525855261

AACACTTGTTCTCGTTGAGAAC 0.57511168513

AACACTTTTTTTCCTAAGCAAA 0.140884767617

AACAGAAAAACTCGTGCTAAAA 0.471486564303

AACAGAACAGCACTTAGAGGTT 0.91986307719

AACAGACACGGTGGTTAACAAG 0.839859531613

AACAGACATAGTGCTGACTCAA 0.788052432348

AACAGACTCTCTGAAGGACAAT 0.58755090319

AACAGACTTTCGTATATACGAT 0.238894599363

AACAGAGAACTTGCTGGCTGAG 0.652141780292

AACAGAGAATCTCCTACGTTAC 0.350191366675

AACAGAGACTCTCAATAAGTAT 0.205970367535

AACAGAGATCCTGAAAACAGAG 0.425940729529

AACAGAGCAGCTCCACATTCAA 0.608284265233

AACAGAGCGACTTCAGTGCCAC 0.690540868929

AACAGAGCGTTTCGTTTCACAG 0.778201575999

AACAGAGCTTTTATATGAGCAC 0.529526745213

AACAGAGGCTCGCAGATTCCAA 0.703968167378

AACAGAGGGGTTCCTATTTAAT 0.560793547677

AACAGAGTAACTGAGTAGACAG 0.499495660669

AACAGAGTAGCTCCACATGAAC 0.513396385957

AACAGAGTATGTGAATTAGGAC 0.198348954151

AACAGAGTATTTGCGGTATAAT 0.465100411155

AACAGAGTTTCTCCATCGTTAT 0.473456349745

AACAGATATTATCCTCGGCAAA 0.50650221153

AACAGATGAGGCCCAAGCTAAG 0.668995529807

AACAGATGTTGTGAAGGTTAAG 0.598100196152

AACAGATTGGCTGATTTGTCAC 0.454736730712

AACAGATTGTCTGGGCAGTCTT 0.854594315628

AACAGATTTACTCCTTGTACAC 0.363091019083

AACAGCAGGTCTGATACATAAC 0.263561946549

AACAGCCCTTGGGATCCGAGAG 1.73088217706

AACAGCCTCTCTGAATGGAAAA 0.622850829796

AACAGCCTGTCTGATGTGTAAG 0.965600286208

AACAGCGAATGTGTGCCATGAT 0.709451170914

AACAGCGGGCGTGCTAGGCGAC 0.428049605965

AACAGCGTATGTTCTGGATTAG 0.335256871555

AACAGCTATATTCCTAATACTG 0.576366969042

AACAGGCTATCTCATAGGACAT 0.313519023065

AACAGGGAAAGTCACTCTTGAG 0.443675134445

AACAGGGCATTTCATTAATAAC 0.903508600629

AACAGTACATCTGATTAAAGAT 0.547784619076

AACAGTCAACCTGAAAATGTAA 0.451957438306

AACAGTCCCCAATACCGACAAA 0.666679452929

AACAGTCCCTTTGCGAACAGAG 0.421159887315

AACAGTCGATCTCAATTCCTAC 0.312455746889

AACAGTCTATTTGCTGGTTAAG 0.676664515529

AACAGTGAAGCTCATGTTGAAT 0.3046732972

AACAGTGACAGTGTTGGTTAAG 0.627762694537

AACAGTGACTCTCCGTTGGGAA 0.864094422413

AACAGTGGAGGTTTTTTTAAAC 0.452373741024

AACAGTGGCTGTCCGATACGTG 1.77939299305

AACAGTTAAACTGACGACTAAG 0.606009894078

AACAGTTAATTTGATTATTTAG 0.386530502099

AACAGTTAATTTGCTCGGTATC 0.615976410564

AACAGTTAGTGGTTTCAAGAAC 0.586751158873

AACAGTTATGATCTATTTATAC 0.241236144568

AACAGTTTAGGTGTTTAACGAT 0.391700824977

AACAGTTTATCCGCCTGGAAAC 0.247314581007

AACAGTTTCGTTCGAATGGTAA 0.527390394665

AACAGTTTCTGTCATAGCGTAC 0.521567083396

AACATAGAATGTCATTCTGAAA 0.163795653089

AACATAGGGAGTGTTAGCAGAG 0.593581126706

AACATATCGACTCCAGTTGTAC 0.765798555543

AACATTCTATCGCGTCTCTTAC 0.375583023628

AACATTGTAAATGACCCCTAAA 0.689999006221

AACATTTGCTTTTAAAAGAAAG 0.335987012327

AACATTTTCATTCATGTGCCAT 0.4803475916

AACATTTTCTATGCAGTGAAAA 0.431618299746

AACCAACTAATTGGACAACTAC 0.468173696893

AACCAAGAATCTGCCCTTGGAT 0.723629879729

AACCAAGGCTCTCAAGAGTGAA 0.473416404871

AACCACCTCGGGGCAGGTAATG 0.460995727248

AACCACGTATGTGCTAAAAAAG 0.503915813982

AACCATGAAGCTCTTATACTAA 0.638849147127

AACCATGTAACTGATCTGAAAC 0.254302829603

AACCCACAATGTCGAAGTTAAA 0.528165478846

AACCCACACACTGATGTTGGAT 0.456417750629

AACCCACATTAGTATTGTAGAT 0.24569958482

AACCCACCGTCTCTTTAGTAAG 0.500521303774

AACCCACTCTGTGGCACGCAAG 0.672058705501

AACCCAGAAAGTCGCAACTAAA 0.49131273014

AACCCAGATTCGGGTGCGGTAG 0.455675134564

AACCCAGCATCTTAAGCCAATT 0.383163681221

AACCCAGTGGGTTCAGTCTCAG 0.448024631136

AACCCAGTTTCTAACACCAAAT 0.604610860921

AACCCATGCTCTGCATGGTAAT 0.276570677918

AACCCCTATTTTGATGGGTGAT 0.949779712774

AACCCGTAGCGTTATGAAACAG 0.39317110646

AACCCGTGATCGGATTCAAAAA 0.722420746759

AACCCTCTAGGTGGTTAAATAC 0.565141720497

AACCCTTGACCTGCATAACTTC 0.40563315767

AACCCTTGATCTGTATATCTAA 0.400660489931

AACCCTTTATCTGAATGGATAC 0.224896272283

AACCCTTTATGGGAGATCTAAA 0.891574088011

AACCGAACATGACCAGTTAGAG 0.910064422737

AACCGACAAGCTGCGGTATCTG 1.12483017708

AACCGACAATCAGAAAGTAGAG 0.239813497419

AACCGACAATCTGATAAAGGAT 0.401082325968

AACCGACAGATGCTAGGAATAT 0.292430434583

AACCGACTTCCTGGAAGTCGTC 0.178628093391

AACCGAGAAATTGAGAGATTTA 0.565433832931

AACCGAGGGCGTCACTGGCGTC 0.999717276166

AACCGAGTATATCCTAAGACAA 0.679791185804

AACCGATACTATGCTGGCATTG 1.13283160488

AACCGATAGCATGCCTCCGGTG 1.10677974899

AACCGATCATGGGGTCACCGAT 1.11516501531

AACCGATGCACTACTATTAGTT 0.761226553304

AACCGATTAGTTCCAAATCTTT 0.237782147252

AACCGCCAACCTGTTATTTAAA 0.43521987232

AACCGCCCACATCCTTAACTAA 0.513571591125

AACCGCTAAAAGGCACAATGTC 0.667622578485

AACCGCTTGACTCCAATGTCTG 0.283958008928

AACCGCTTGGGTCATCGGTGAA 1.38238764873

AACCGGCTATATCAATCCGATC 1.26671153916

AACCGGGAATTTGGGGCAACAA 0.61571231933

AACCGGTTCTGTGGTACCATAG 1.48922486368

AACCGTCAAGCTCAAGACGGAC 0.313371298303

AACCGTCAATCGTCTGTCGCAG 1.47996757982

AACCGTCATCGGGCGTGGTGAT 1.81663524277

AACCGTGCCTACCATGATGCAA 0.921593548632

AACCGTGGAAGTGCAGGAAGAT 0.462062886402

AACCGTGTACCTCTACATTTAT 0.385463187486

AACCGTTAGACTGTACAGGGTC 1.10255179946

AACCGTTATTCTGATCCTTAAG 0.470715164363

AACCGTTCACGTGTCGTTACAC 0.376867390803

AACCGTTTGCGTGAACTGTTAC 1.57716889877

AACCTACAGGATCCTAAATGAG 0.279741583489

AACCTACCCTCTGATACAGGAA 0.459108045288

AACCTAGAGTTTCCGTAGGGAT 0.84271433758

AACCTCTTCTCTCATGTAACAC 0.835700126103

AACCTGAACTGTGCAATCTCTT 0.873516681116

AACCTTGGAGCTCATCCTGCTC 0.465371857246

AACCTTTAGTATCCTTCGGAAA 0.351848102108

AACGAAGACTGTCCTTGGTGAT 0.690494176909

AACGAAGAGTGTGGATTGTAAC 0.601906168284

AACGAATAAATACTTCCCCCAA 1.10936125182

AACGAATACGCGGATTCGCAAG 0.439788169489

AACGAATGAAGAGTTCGATTTG 0.252279651085

AACGACCCGTGTGCATCAAAAT 0.578773692532

AACGAGTCCTCAGCTACTATAC 0.638613011195

AACGCACACGCTCCGTCACAAA 0.285180386477

AACGCACGCTCTTGAAAGACAA 0.622510893064

AACGCAGAATATGTAAACACAC 0.949487176128

AACGCAGAATGTGCTCTCTTAT 0.530654025624

AACGCAGAATTTCCAAGCTCAT 0.400200576536

AACGCATAAAGTCCGGCCGTAG 1.54084330451

AACGCATTGAGTGCTCCTATAG 0.844392176313

AACGCATTGGTCGGAAAGAGAG 0.644133004901

AACGCCCACAGTGACAAGATAG 0.317132439898

AACGCCTACCCTGCTCCACAAT 0.619503702507

AACGCGTTTGGTGTACGTTAAG 0.372999471666

AACGCTAACCTTGCTCTGTTAC 0.490256579818

AACGCTCCATATACTGATATTC 0.690187527954

AACGCTCTAATTACTTAAAGAC 0.190061833988

AACGCTGACTTTCGAAAATTAC 1.01489455123

AACGCTGCCATTGACTGATCAC 0.701853979246

AACGCTTCGTGTGCTATAAATC 0.9017130333

AACGGACATTCGGCGGAGGAAA 0.546594836708

AACGGACATTCTGCAGACCAAA 0.76428590761

AACGGACGGCTTGCACTCGAAG 0.560433626713

AACGGACTAAGGGATTGCCATG 0.763110298911

AACGGAGCATTTCTGGCCTAAC 0.493019340243

AACGGAGGGCCTGAAGGGAGAA 0.759118616337

AACGGCCAAGATGCGAAATAAC 0.331004872307

AACGGCCAATATGAGGTATTTC 0.60051707951

AACGGCGTATCGCCCGCCCCAA 1.62306203738

AACGGCGTATGTCCCGCCTAAG 0.549414045229

AACGGGGACTGTCCTAATTTTA 0.692845369766

AACGGGGCATTTCCTCAGTCAT 1.00934694679

AACGGGTCAACTCCGGTACAAG 1.00677033093

AACGGTAAATCTGCCGCCGGAC 0.666626154419

AACGGTCTGGATGGCGGTGCAG 0.749039412193

AACGGTGACATTCCATACGTAA 0.384977363628

AACGTACAATCTGAACCCTAAA 0.568611451857

AACGTACTCTATGATAAATGAG 0.379054127808

AACGTAGCAGCTGGATCGCTAA 2.29218245894

AACGTATGCACGGATGGCCAAC 1.01137276545

AACGTTCTGACTCCCCCTTTAA 0.680029198356

AACGTTTATTGTGGGTCCTTAG 0.309632871104

AACTAACCAACTGCTGGACAAG 0.443150121377

AACTACGAGTGTGTGATCATAT 0.680828718409

AACTACGTCGGCGCTACCGAAA 0.648656135048

AACTAGAATTCTCCATATTAAT 0.544951399174

AACTAGGCTGATCATTCGTCAG 0.519710868546

AACTAGGGACTTGCTGTAAAAA 0.698079650195

AACTATGAGTCTGATATTTTAT 0.0837797555436

AACTATTGAATTGACTATCAAT 0.231677309541

AACTCAATAGCAGAAGGATGAG 0.953823627365

AACTCACAAACTGCTAAAAGAA 0.372852785629

AACTCACAAACTTCAAGTCGAC 0.439192466193

AACTCACAGTCTGCTCCCCAAT 1.25639572457

AACTCACCAGGTCCAAGCCTAT 0.817985528098

AACTCACTAACTCAAGCAGCAC 0.492528963684

AACTCAGAGTATCCAGAAGTTG 0.423708771938

AACTCAGGGTGTCATTTTGTAT 0.176717908143

AACTCAGGTCATGATAGGTGAC 0.59951996044

AACTCAGTAAGTGCATACACAG 0.452974293229

AACTCATAAACGCATATTTGAT 0.26365465558

AACTCATACACTTTTAGGTTTT 0.458161810874

AACTCATACGCTGTTGACTTAT 0.197532183946

AACTCATAGACTCCTATAAAAC 0.578836755436

AACTCATCAGCTCCAACACGAG 0.439457103098

AACTCATCATGGTCTTGGGGAA 0.864613823751

AACTCATCATTTCAAGGCCAAG 0.621693030244

AACTCATCGTTTTCTGTCACAT 0.71372723632

AACTCATGAGCTTCCAGGAAAC 0.662731264123

AACTCATGCTGTCAGTGAAGAC 1.339015768

AACTCATGGGGTTATTCTAAAG 0.519289388777

AACTCATGTTGGGTTGTAGCAG 0.463980890174

AACTCCCCGGGTCTTTCGGTAC 1.6764655196

AACTCCGCCTCTGCTGAGATAG 1.01277918646

AACTCCTAGGGAGTATAAGGAG 0.410025658124

AACTCCTGGTCGTCTAATAAAC 0.209279081238

AACTCGCCCGGTGATTTAATAC 0.714967516803

AACTCGGCCTTTCATGTTAGTC 1.25988466315

AACTCGGTGACGGCAATCTTAA 0.708238630289

AACTCTCAATGTGTTTTAGTAG 0.541730521139

AACTCTCAGTGTCATCGTGAAC 0.222826618462

AACTCTCTACGGCAAACTATAA 0.541613406226

AACTCTCTAGCTGAGCCCCTTC 0.389172718854

AACTCTCTAGCTGCACGAGGTG 0.720003033352

AACTCTCTTGGTCACGTTGCTG 0.595229061658

AACTCTGAACCTGTTTGGCCAT 0.668632612824

AACTCTGAATATCATACGTTAT 0.37212767188

AACTCTGACTCTCATGTCTTTT 0.32838230953

AACTCTGATTGTCATCATAAAC 0.242583481951

AACTCTGGAAGTCAAGCACTAC 0.77004203163

AACTCTGTAGGCGCATGTTGAG 0.77676027391

AACTCTTAAAGTCCTACCATAA 0.314921656372

AACTCTTGGAATCTTTACCAAT 0.395830136151

AACTCTTTACCTGAATACTCAT 0.2917375118

AACTCTTTCTCTGTGTTGGTAG 0.438751312782

AACTGACACCGTGTTACCTGAC 0.401827039859

AACTGACATGGTTATGGTCCAT 0.364178389754

AACTGACTAGACCAAAGTGAAT 0.628997573792

AACTGAGACCGCCATATGTAAT 0.549791815596

AACTGAGAGCATGATGCCTAAC 0.926586186513

AACTGAGATGTTGTATCTCTAG 0.955600147902

AACTGAGCACCTGAAACCAAAT 0.81011817117

AACTGAGGGAGTTATAGGTGAA 0.741508211113

AACTGAGTAGCATATGCTTAAT 0.392447360194

AACTGAGTAGGTGTTTGTCATG 0.904555063361

AACTGAGTGAGTTATTTGAAAA 0.503168087383

AACTGATAATTTCAATGATGTC 0.477425275771

AACTGATTATGTCAACGATAAC 0.341712152883

AACTGATTGGTGCATTCTGGTC 0.715385228338

AACTGATTTGAGGGTGGTGTAG 1.1983675522

AACTGCCACCTTGATGACAAAT 0.878761591218

AACTGCCAGCCTCGAACACGAG 0.658231696911

AACTGCCGCTCTGGAAGAGAAC 0.234252122734

AACTGCGATCGTGCTTGTAGAA 0.139554481852

AACTGCGCATCTGATGTTTGAA 0.750128242368

AACTGGGCTAATGATGTGCCAC 0.944217971216

AACTGGGTATGTGCTAAGGGAC 1.47783434575

AACTGTAGCTCTGCACGCGGAG 0.859196799417

AACTGTCAACGGTCTCTTAAAC 0.475020544086

AACTGTCATTGGGCTAAAAAAC 0.546250699588

AACTGTCCATGTGTTTTATAAT 0.430629360745

AACTGTCGAGGTCATCTAAAAC 0.607437141542

AACTGTCGTTCTGCTTCTCAAG 0.559393960556

AACTGTGATCGTGCTTGGGAAT 0.541657214417

AACTGTTAAAGTGTGAGGTCAC 0.477781970823

AACTGTTCATGACAAAAGTTAC 0.403918516649

AACTGTTCCGGTGATTAGTATT 1.29151353447

AACTGTTGCGGTGACTCGTTAA 0.785878189338

AACTGTTTATTTGATCCAATAC 0.519894498864

AACTGTTTGCCTCATCGACTAG 0.452593691409

AACTTAACATCTCACCCATTAG 0.243344149527

AACTTACTAGCTGCTTCTACTG 0.671823500755

AACTTACTATGAGAGTTGGAAG 0.180802488149

AACTTATTATTTCACGCTTAAC 0.744842815391

AACTTCCACTCGCATGTTGCAG 0.213864609102

AAGAAACACATCCCTCTCAAAC 0.396176178824

AAGAAAGACCTTGTCAATTGTG 0.356503189916

AAGAAAGTATCACATCTCAAAT 0.256522012633

AAGAAAGTATTTGAATCAAAAG 0.275245293332

AAGAAAGTTTCACAAATTCTAT 0.433432130525

AAGAACGTATCTGATTCGTCAC 0.200072974336

AAGAATCGTTCTTATAGGATAT 0.146165543957

AAGAATGGTTTTGCACCATCAC 0.675882164384

AAGAATTCGTCTTAGGACGAAG 1.02296257595

AAGAATTGAGATGTATAATAAT 0.195751088849

AAGACACAACGGCATGTCTGAG 0.910839020231

AAGACACACCCTCCTAATTTAC 0.546780926744

AAGACACTACATGCATGTAGAT 0.414779769641

AAGACACTAGCTTCTTCCAAAA 0.324185475301

AAGACAGAACGTCTAGAGTCAA 0.276510134192

AAGACAGAATGATCTTCTAAAC 0.322994386683

AAGACAGACGGTCGTATCCCAC 1.18594284293

AAGACAGAGGGTCCTACCTAAG 0.295401923071

AAGACAGGCGCGTCTACTCAAG 0.799516224524

AAGACAGTATGTGATAAGAAAG 0.403035091288

AAGACAGTATTTTATGGGTGAG 0.374865062373

AAGACATAATGTGCAAGTACAT 0.256971262582

AAGACATAGACTGCTCGCTGTT 0.320828024336

AAGACCGGAAATCTTAAATGAC 0.280421781463

AAGACCGTCTCTGATAACAAAG 0.541603603165

AAGACCTACACTCGTGATAAAC 0.774292339186

AAGACCTACGTTCCCACAACTC 0.869570840654

AAGACCTTCTGTGCCATATAAC 0.78677026116

AAGACGCGCTGTCACCGCTGAT 2.04077028417

AAGACGCGGTCTGAACCCCCAC 1.21888905671

AAGACGTGATTTGTAGGCTAAC 0.416233201631

AAGACGTTGATTCGTTCATAAC 0.362273352157

AAGACTACAGGAGACGACAGAT 0.260509253735

AAGACTCAGTCTGCACACCTAT 0.385973375166

AAGACTCGATTTCCAAGTTGAG 0.803177726403

AAGACTCGTGGTGGTTGGATTA 1.6054824988

AAGACTCTGGGTCAGGCCAAAG 1.16607716228

AAGACTCTTACTCATGCCATAT 0.719401210557

AAGACTGGTTCTGATTAGCCAC 1.22620580243

AAGACTGTCACAGGTTCATAAT 0.542625638661

AAGACTGTGAATTAATAAGAAT 0.866114564942

AAGACTTAATCTGCATTGAAAT 0.357528577728

AAGACTTCATCTCGCTAAGAAC 0.438033608783

AAGACTTTCGTCCCGCGTGGAG 1.11016837886

AAGAGACATTCTTATATAGAAA 0.236227067979

AAGAGACCCAGTGCTGAGGTAC 0.411258244232

AAGAGAGAAAATGCATGTGAAG 0.276281075339

AAGAGAGACTGAGGTCTAATAG 1.24441075759

AAGAGAGAGCCGGCTAAATTAC 0.793690635797

AAGAGAGTAACTCATAGTAAAA 0.45882144643

AAGAGAGTGCCTCCTGGGCAAA 0.842147426695

AAGAGATAATTTCTAGAAGTAA 0.441655828967

AAGAGATACTCTCCAAGTAGAT 0.635795335017

AAGAGATGCTGTGCCTAGTTAT 0.255793154898

AAGAGCGGCACTTATGGCCTAT 0.56321570516

AAGAGCTAATCTAATGAAACAT 0.598086882433

AAGAGGCTTTCTGCCCAGTATG 0.964527959662

AAGAGGGATAATCATAGCGCAG 0.934113740938

AAGAGTAAGTCTGCACGATATA 0.505690989356

AAGAGTCAAACTCCAGCAATTG 0.606338459376

AAGAGTCCGCGTGATAATTGAT 0.975033704135

AAGAGTCTGAGTCGAAGAGTAA 0.462573531871

AAGAGTGAAACCCAAGAAGGAG 0.465603462787

AAGAGTGAACGTGCCAAACGAG 0.883968582174

AAGAGTGGATAACCTGCATCAG 0.432045119414

AAGAGTGGTTTTGATATTGAAT 0.454438047738

AAGAGTTACTCTGACGGCTTTT 0.459368069738

AAGAGTTATAGTTCAAAGTTAC 0.515134738879

AAGAGTTCGGCACTAGGTAATC 0.833498258827

AAGAGTTGTTGTGAGAGGCCAG 0.767261487129

AAGAGTTTATCTGCTGTAAAAT 0.315841460006

AAGATAGGAGGCCATGCCAAAT 0.489233302284

AAGATAGTGTCTATTGAAAGAC 0.362175753243

AAGATGCTATCTGGTTAGCAAA 0.465302647988

AAGATTCAACGTCTTACAGGAC 0.633913143486

AAGATTCAATGTCATGGCAAAG 0.546175577367

AAGATTCTATGGTTAAGAGGAG 0.317598669202

AAGATTCTGAATGTTCCACCAA 0.765157250778

AAGATTGTCAGAGTTCCCTCAA 0.420947000345

AAGCAAGAGTGTCCTAGAACTT 0.61040277344

AAGCACGAATTTGGTTGGCATC 0.831764600521

AAGCACGGAAGTCCTCATATAT 0.562851319731

AAGCCACAATGTGATCGTCCAG 0.449758041216

AAGCCACACTCTGAATGCGGAC 0.920450619884

AAGCCAGATTGCCTAACTAAAT 0.862444775061

AAGCCAGTCCTGCATGGTAAAC 0.639192894184

AAGCCATACTCCGAGCGCCCTT 0.778144151977

AAGCCATATGTTCGCATTGCTA 0.502151762325

AAGCCATGAGCTGTACGAGGAA 0.960189075755

AAGCCATTCGGTGATCGTTCAG 0.312911742049

AAGCCCGAATCTCCTGACCCAG 0.617115093033

AAGCCCTACGGTCTGTTGTAAG 0.600778168149

AAGCCGCGAGTTCAATCACTTG 0.413700195788

AAGCCGGAATTTGCTTATAAAA 0.42765498627

AAGCCTCGAGTTGTTTGCCAAG 1.55205004492

AAGCCTCTAATACTACCTTAAC 0.408439643011

AAGCCTGACCCTGCCTGGGATT 2.11005909474

AAGCCTGAGAACCCAGGAGAAA 0.396823543508

AAGCCTTAGGCTGAGAAGTCAG 0.806478876952

AAGCCTTCAGGTGAAGTCTTAA 1.24751016333

AAGCCTTGACATGCTGGCCAAG 1.22263555789

AAGCCTTGATGTGCTACGACTC 0.738308896365

AAGCCTTTATATGCACTAGGAA 0.365588996324

AAGCGAACCACTGGAAATACAC 0.293852914774

AAGCGACAGTCTGTTCGCGCAA 0.931243411457

AAGCGAGAATCGGCGGGACAAG 1.3601202446

AAGCGAGATTAGGTTCTTGGAT 0.286931705438

AAGCGAGGAAGTACGCAAACAT 0.457025619227

AAGCGATTCACTCAATATGCAG 0.70296691259

AAGCGATTGACTCATACATCTG 0.22586894219

AAGCGCCAACGCGCGAGAGAAG 0.545921378183

AAGCGCGCAGTTCGCAAATTAT 0.402030366082

AAGCGCTCGCGTGTTTTTAAAA 1.03374755894

AAGCGTCAATGTCCTATCCTAT 0.794443518418

AAGCGTCAATGTGATTGCCCTG 0.640066199615

AAGCGTCTAAGTCGTGTCTTAT 0.682247887274

AAGCGTGAGTGTGCTTGCAGTC 1.76213924223

AAGCGTGCCTATCCGCATGAAT 0.918444722861

AAGCGTGTATCTGCTATGCTTT 0.874196066105

AAGCGTTTGTGTGCTGATCTAG 0.971511006404

AAGCTACAATGAGCTCACTAAC 0.977122539929

AAGCTAGGGCCTGTTAATTGTA 1.49706030627

AAGCTATAGCGCCCTTCTGTAT 1.28180907307

AAGCTATTATGTGCGCCGTGTG 1.53241612015

AAGCTCGGGGAAGATTTTTGTT 0.271801258989

AAGCTTACCTCTCTTAATGAAT 0.455064487466

AAGCTTGCTTCTCAATGTAGAT 0.423707605696

AAGCTTGTATGTTGTCAGCGAA 0.512766952893

AAGCTTTCATTTGAATTAGCAC 0.491619037202

AAGGAATGCACAGATATGGAAC 1.22529151615

AAGGATCTGGCCGATTTCTATC 1.00436722163

AAGGCACGGGTTTACTAGGCAA 1.48565535297

AAGGCAGAGATTCATAACCTAT 0.408738847993

AAGGCCCGACTCCCTTTTGTAA 1.00659751308

AAGGCCGAAAGTCCTGAGAAAC 1.14113562961

AAGGCCTCAACTGATTTTGGTG 0.268206244629

AAGGCGGCACGAGCCGGACGAG 2.821813924

AAGGCGGTAGCTCAGTTAGAAT 0.482702517091

AAGGCTACGTGTGCTACTCGAT 1.2444426604

AAGGCTTGATCTGGGCGGCGTC 0.813700520741

AAGGGAGAACGTCAATAGACAG 0.499239073943

AAGGGAGCATTAGCTGACTCTA 0.982004855693

AAGGGAGCTGCGCCTTAGGTAA 1.03527949963

AAGGGAGGATGTAACGGATATT 0.502760738638

AAGGGATTAGCGCAAGGTTCAG 1.13102681322

AAGGGCGTATTTGATGACGCAA 0.885817156118

AAGGGCTTGGGTCAGGTCATAG 0.655808926231

AAGGGGCAGATGGCGGGTCTAT 2.24753223762

AAGGGGGAATGTGTAACGGGAG 0.134376462076

AAGGGTCCCTTTTAATGGGAAG 1.30666713166

AAGGGTCCGCGTGCAGAAATTG 0.87251193413

AAGGGTTCCTCTGCAACGTTAG 0.744049924846

AAGGGTTTTTTTGATTGCGATC 0.404918399913

AAGGTACTTGATCAAATACAAT 0.269621833694

AAGGTGGCCTCTCTAAGGAGAG 1.20279188019

AAGGTTGTTTAGGAAGATTAAT 0.361811091274

AAGTAACTACGGCCGAATTCAC 1.20703377156

AAGTAAGTAACTGCAAATCCTG 0.425736186894

AAGTAATTACGGTGGAAGCAAC 0.628114883242

AAGTACTACCCTGAACGTTATG 0.517476612345

AAGTAGGGATTTCTTCGCTTTC 0.215944292016

AAGTATAGGTGTCCATGTAAAT 0.504975156247

AAGTATGAAGGTCCTAAAATAC 0.139567681144

AAGTATGATTCTCCACATGGAG 0.43266367864

AAGTATGTTGCTGATTTGGGAT 0.720863051575

AAGTATTTGCACGCTAATCGAA 0.222453358265

AAGTCAAAAAATGATAAGCCAG 0.108704216915

AAGTCACAAGACTAGAATTGAG 0.463761712437

AAGTCACATATTGATGCTACAG 0.607413051337

AAGTCACCATAGGCCTCTGAAA 0.533141409402

AAGTCACCTCGCCATATCGTAA 0.181179797194

AAGTCAGAAGCTCTTTAGCAAC 0.422725800844

AAGTCAGGATGTCCTGCCCAAC 0.595983091937

AAGTCAGGCTATCTCCAAATCG 0.987236416315

AAGTCAGTATGTGGCGAAAGAC 0.830733601096

AAGTCAGTCACTGTGGTACCAT 1.14988992791

AAGTCAGTGGGTGGTTGATGTC 1.12033400526

AAGTCATCCAGTCTCATCGGAG 0.171185079943

AAGTCCCTAGGGCCTTACTATA 0.390971129082

AAGTCCCTTTTTGGACAGATTG 0.787316177336

AAGTCCGACACTCATACTGCAA 0.525684475342

AAGTCCGTCCGACTTAATATTC 0.810053443379

AAGTCCTATTTACCATAGGTAG 0.349753099441

AAGTCCTTATGTCGTAAAGTAC 0.361168813335

AAGTCGGAAGGTCATCAGACAA 0.706003562444

AAGTCTCCACGTGTTGGCGAAG 0.749432926684

AAGTCTCTCTGTGAGGAGCGAT 0.723152275334

AAGTCTGCATCTTAAACAACTT 0.61283273586

AAGTCTGCCGTTGCATTACAAC 0.916685399923

AAGTCTGCGTGTGTAGATAAAA 0.503611073995

AAGTCTGGGCTTCTTAAGTAAT 0.58859624481

AAGTCTTATTCACTAAATTCAA 0.390936078728

AAGTCTTGTGATCTACACACAC 0.61964106706

AAGTCTTTTTATGCATTTTAAG 0.511284646284

AAGTGACAAGCCCCTCTCGAAG 0.597127800807

AAGTGACTCTGTCCTCAGTGTT 0.948581900466

AAGTGACTTGCTGCGAAGAGAG 1.5170837083

AAGTGAGAAGCTGCTATCCTTT 0.782162294491

AAGTGAGACGCTCACAGGATAC 0.806711278963

AAGTGAGCGCATGGTGCGTGAG 0.575421306234

AAGTGAGGCTCTCAGACGTAAA 0.971183326724

AAGTGAGGGTGTGATGCTGGAA 0.651576637842

AAGTGAGTCACACATGACGCTC 0.598395909409

AAGTGAGTTTACGCTTATCTAC 0.325972589353

AAGTGCCGAGATCCTAGAAAAC 0.576358403669

AAGTGCGAGTGTGTTTTGGGAG 0.966295546035

AAGTGCGATTGCGAGTCCGCAG 0.450815188996

AAGTGCGCATGTCCTAAAGTAA 0.423830567996

AAGTGCGCCACTCTTGGTCTAT 1.0779850466

AAGTGCGGATATGATTTTTCAC 0.591329201145

AAGTGCGGTTCTTCATTGTGAA 0.719535722102

AAGTGGGGCCTTTCCTTTGTAC 0.93839026515

AAGTGGTAATATGAACACTTAG 0.62538413969

AAGTGGTCAAGTGCTGTAATAA 0.127020212088

AAGTGTGAATCTTTTGTTCCAG 0.503001981369

AAGTGTGAATGTGCATGGTAAA 1.6645063352

AAGTGTGAGCCGGATAGCGCAT 1.55384660995

AAGTGTGGCTGTCATGAAGGAC 1.07943249486

AAGTGTGTATCCGACTGGCTAG 1.12344228861

AAGTGTTGATCTCATGCGTCAA 0.710352387886

AAGTTATAAGCTGCCGACCCTG 0.512255292239

AAGTTCCCAAGTGAGGCCTAAC 0.683837187545

AAGTTCCTCCGGGTTTAGTTAG 0.772002621642

AAGTTTGAAAGAGATGATAAAC 0.273645149291

AATAAAGAATTTTCTGACCTAA 0.405314239822

AATAAATCGTGTGTTCTTTATG 0.315535006544

AATAACTTATATTAAAGGGAAG 0.370797368944

AATAATGTGTCTGAGTCTTAAA 1.07490983095

AATACAATATCCCCTCAGAGTA 0.512594691335

AATACACAGTCTGCGTCGGAAT 1.31106283452

AATACACTTTGTGATATAGAAG 0.301825724361

AATACAGAAAGTGCGCGTCAAC 0.350657964806

AATACAGGATATCTGTTAGAAC 0.284964530914

AATACAGTCACACCCGCATAAT 0.529762255968

AATACAGTGACTCATAGTGGAC 0.263842248573

AATACATAATTTGCTCGCTATC 0.356979438296

AATACATCGTCTGAGCGGTAAA 0.400968538323

AATACCCTCTATGACGTAAGTT 0.583331824872

AATACCGTAGTTTTAGTAAGAG 0.716790074191

AATACCTACTCCGGAAAGCAAG 0.375678770716

AATACGGTCTCGGTTTGCTAAA 0.357290043864

AATACTCACGCTTGTAAATTAA 0.390051904272

AATACTGACTTGCGTCAAAGAA 0.22142682451

AATACTGTCTGTGGACTCGATA 0.680891430951

AATACTTAAATTCATTGACCAG 0.548622717967

AATACTTGCCGACAATATAGAT 0.475232287483

AATACTTTGATTGGAACTCAAG 0.580927583435

AATACTTTTTCGCAATATTGAG 0.697842909176

AATAGACGAACTGACGAGTAAA 0.477455032997

AATAGACTGCGTGCCTTATCAT 0.721957415593

AATAGAGAGTGTCGTGCTGGAC 0.819640334809

AATAGAGCATTTCGCGGTTCAG 0.744035993292

AATAGAGGATGTCCACGTGCAA 0.680059654404

AATAGAGGCTCAGCTCGGAATT 0.547329139067

AATAGATGAAAGAATGGAAAAT 0.787599387573

AATAGATTATCTGATGTATGAA 0.322376476845

AATAGCCATAGTACTAGTTTAC 0.365334567223

AATAGCTTCCCATCACGATAAC 0.393267779348

AATAGGTCCAGTGTTCTCTCTC 0.594095000552

AATAGTCACTGTTTAAGTCTAT 0.313826577318

AATAGTGAACGTCGTTATGTAG 0.948218955501

AATAGTGATTCGGTTGCAGAAA 0.399863837976

AATAGTGTGTATGATCGTGAAA 0.924878517326

AATATATATTGGCCTGGATAAC 0.289238616929

AATATATCCTCTCCCACTCAAT 0.514506211381

AATATCCCAGAACCATTGGAAA 0.402077916168

AATCATCTTTCTCATGGGCGAT 0.951414561355

AATCCATAAACTGCTGTCTTTG 0.55086177817

AATCCATAATGTCTACCTTTAC 0.528042053443

AATCCATGATCTGGGACTGAAT 0.583683617068

AATCCGGTTTCTGTAAATCTAG 0.663766302813

AATCCGTAATCTTTTACAGAAC 0.498041126362

AATCCTATGTCTCGTCGGAGAT 0.706898753205

AATCCTCCACGTGGAAGCCCAA 1.05774936675

AATCCTGAAACTGCTCAGAAAC 0.4797362612

AATCCTGACCGTGCTGCATTAA 0.760982356508

AATCCTGTATGTTCTCAGTCAA 0.407115753669

AATCCTTAAGCCCCTCTTATAG 0.81984385793

AATCGACAAACTTATCTTTAAG 0.70790485149

AATCGACAGTGAGTAAGTAGAA 0.587907692988

AATCGAGACCATGCTGATGCAT 0.401042426643

AATCGAGATTCAGCGGGAGATG 0.797379766418

AATCGAGTAACTACTTCAAAAC 0.221328676238

AATCGATTTACTGGTGGGGGAC 0.457571755676

AATCGATTTTCTGATTCTTGTG 0.368478435957

AATCGGAACCGTGATGCAATAA 0.594687729327

AATCGGCATATTGTATATATAG 0.250003608441

AATCGTCACACTCGCTTCGAAG 0.850324057768

AATCGTCTAAATCTAGGCGAAG 0.607897604258

AATCGTGAGTGTCAAACCAGAG 0.504387771141

AATCGTTACCGTGCAACGCAAC 0.280363446899

AATCTACAAATTCTTATGATAC 0.458010323146

AATCTATCTTCTGTATAAGAAG 0.254989318641

AATCTTGAATGCGACCGTCCAT 0.927111394387

AATGAGGCCGGTCCTCGCTGAT 1.04037891568

AATGATCTGTGTTCTGCCAAAT 0.514774101425

AATGCAAGCTCTCACGTATTTT 0.385134212641

AATGCAGCGTGTCTCTCACGAG 0.563385955806

AATGCATACTCTCTAAGTGTAG 0.77315053688

AATGCCCCGCCTCATCGGTCAG 0.585142537975

AATGCCCGAACTGATGAAAGTC 0.509287386397

AATGCTGCGTCTCATAATGATC 0.541719632013

AATGGATGTAGTGATACCAAAC 0.307597423876

AATGGATTGTCGGCTAACATAG 0.60955560568

AATGGCGTCAATGACCGACCAG 1.88621459072

AATGGGCACGCTCGAGAATGAC 1.0550835485

AATGGTGTCGGTGATAGCGGAG 1.70262101911

AATGGTGTCTGTCCTATGGAAG 0.552191088758

AATGTAGAGTCCGTGACCGTAT 0.9346084282

AATGTATGTCCTATATATTTAT 0.222685095565

AATTAACGCTCTCATGTAAAAC 0.511375777241

AATTATTAGCTTGCGAATGTAC 0.389938234363

AATTCAAAATGTCCTCGGGAAT 0.254289688769

AATTCACAGCCTACAAAACTAA 0.323053415054

AATTCACTCTAGCACCTCAAAT 0.464702694268

AATTCAGCCCCTGCTTGACTTA 0.593656848107

AATTCAGGCTCTGCTTGCTAAT 1.41225630798

AATTCATAAAATCATCGACGAG 0.396713458998

AATTCATCATCCGCATCTATAG 0.327695966864

AATTCATGGGCTGGAATAGAAG 1.41623091891

AATTCATTCACTGCACTGCATG 0.184823746563

AATTCCGTAGCACACGTATGAT 0.33691886101

AATTCGCGTTGTTCATGTTAAC 0.331118079861

AATTCGTCGTCTCATCTTCTTT 0.104976344549

AATTCTGAAGGTGCAACATAAC 0.877500121225

AATTCTGAGGTTGATCATGCAA 0.50826676831

AATTCTGTGTCTGTAGGTATAG 0.455692416174

AATTCTTACGCTCAATTACGAG 0.441015517568

AATTCTTTATATGTACATGGTG 0.310880729841

AATTGAGCCTGTCCTATGTGAT 0.300563518269

AATTGAGCCTGTGCTAGGACAG 0.711759637509

AATTGAGGATGGGGTAGCTATC 0.111574088027

AATTGAGGGTGTCATAGCTCAC 0.842963067831

AATTGAGTCGCTCATGAGTTTG 0.766753479581

AATTGATCAGATCCGCGGCAAG 0.597096341303

AATTGATGTGCTGGAGTAACAC 0.648296954273

AATTGATTACCTGAACCAGAAA 0.675406100428

AATTGCAAGCATTACGTTCCAT 0.345883540269

AATTGTCAATAATACTTGCCTC 0.689420792883

AATTGTCTCTGTCTCACAACTT 0.488351005243

AATTGTCTGGGTGTTTTGAATG 0.74601359427

AATTGTGGAACGGCGTACGATT 1.28447910509

AATTGTGGAACTGGAGGCCGAT 0.964572519588

AATTGTTAAAGTCGTCTGTAAT 0.249507631545

AATTTAGGAGAGCTTCATAAAC 0.568608112464

ACAACACACCCTGGATTTGAAT 1.27438435735

ACAACATACTGTGATACACCAT 0.363835064018

ACAACATTATGTGGTAAATATC 0.354152264775

ACAACGGTATTTGTTACGGCAT 0.231728035921

ACAACTGAATCTCAAGGAAGAA 0.445939931225

ACAACTTACAGTGTCTGGTAAT 0.612393130614

ACACCCTTTTCTGATTTATAAC 0.551597761427

ACAGAGGGAAATGTTATGTAAG 0.369333432948

ACATCCTAATTGGATCGCATTA 0.581444250649

ACATCGCGTACTCATCCGTAAC 0.523248134126

ACATGAGAGCCGGGTGACTGAA 1.71063648319

ACCAAAGTCTTTGATCAACCAG 0.519527492821

ACCAACGTAACTCGTTTATGAG 0.76921543818

ACCAACTACTCTCCTAGTGAAA 0.565026263005

ACCAAGAGGCCTGGTCGTTAAG 0.890420243998

ACCAAGCTAGCACCCATTGAAT 0.413249392349

ACCAAGGACTCTCCAACTTGAC 0.808985864699

ACCAAGTCATCTGTTTTTTAAG 0.251870357082

ACCAATCAGTTTGAACACATTA 0.334186947736

ACCAATCCTAGTTTTCAATATT 0.206588029452

ACCAATGCGTCGGTTAGCAAAC 0.639282438796

ACCAATTAATATTTTGTCTTAG 0.64730609959

ACCACAACGCCGGCACGCTAAC 1.35852428727

ACCACACCATCTGACAGTATAC 0.264259652336

ACCACACGGTGTGCTGAAATAT 0.531301064976

ACCACACGTTGGGCTGGATATA 0.310009265884

ACCACAGAGGGTCGTGACAAAT 0.562584963563

ACCACAGAGTGGCCTAGCTATC 1.87030040323

ACCACAGAGTTTTCTAGTCGAT 0.807030228049

ACCACAGTATGTCCGGGTAGAG 1.4881917769

ACCACATCCAGAGTAAGAGAAG 0.556880875648

ACCACATCGTCTTCTAGAGGTG 1.18077353012

ACCACATTGGCTCAGATGCCAC 0.927668576497

ACCACATTGTCTTGAACTAAAT 0.750481204796

ACCACCCTTAGTCATACACGTC 0.51693827339

ACCACCGCATCTCCACCGAATG 1.21709410054

ACCACCTCAACTGCTGATTCAC 0.987354103867

ACCACCTGCTCTGAGTAGATAA 0.588777222893

ACCACGGAAAGTCATTAAACAT 0.417330364301

ACCACGGCTCTTCATCGTATAA 0.270846372898

ACCACTATTCCTGAGTCTGGAG 1.06597477036

ACCACTCACTGTTGGTATGTAT 0.215358811338

ACCACTCTGTTCTATTCATATA 0.396481190994

ACCACTGAAAGCTCTTACAATA 0.417379693683

ACCACTGAACCTCAAAGGTGAT 0.412415146491

ACCACTGATCGTGTTAGGGAAC 1.12007705664

ACCACTGATTTTGGGAATGAAA 0.571848031714

ACCACTGCACTTTAATTATAAA 0.503197822577

ACCACTGCAGATGCAGGATAAG 0.454650901937

ACCACTGCTACTCGAACTTCAG 1.44654271753

ACCACTGTACTTTCTCGAGGAC 2.03525796836

ACCACTTTATTCCATCATTTAT 0.188337948763

ACCAGACAAATTTATTAGTGAT 0.29317993151

ACCAGACAACCTGAAACAGATG 0.689343775749

ACCAGACAATCTTAAACAGAAA 0.442283453182

ACCAGACGCAGCGGTCGGTTAG 0.243669967988

ACCAGACTACGTGTAACGTAAA 0.384236325847

ACCAGAGACCACGAATAGTCAC 0.410455226181

ACCAGAGCATTTGTTCATTGAC 0.930120975334

ACCAGAGGCTGTCCAGTGAATG 1.11033259515

ACCAGATAACGTCACGCCATAC 0.893290167329

ACCAGATAGCGTCCTAGGCAAC 0.748424345523

ACCAGATGATATCGCAACACAG 0.431785293807

ACCAGATGATCTCATAGTGGAT 0.222533602524

ACCAGATGATCTCGATGCATTG 0.88435146567

ACCAGATGATCTTGTAAATGAA 0.760042927452

ACCAGATTAACGGAGGTCGTAG 0.819457905928

ACCAGATTAAGTCATGAGGAAG 0.531316734716

ACCAGCCGTTCTCAATAGGAAA 0.260248006668

ACCAGCGTATCTGTCCGTATAT 0.534718607762

ACCAGCGTGACAGCTCAATGTG 0.0675381022141

ACCAGCGTGACTCCATAACTAT 0.538053399013

ACCAGCTAGTTAGAACCTGAAG 0.642568255301

ACCAGCTTAGGTGTCTTGACAC 0.454795754253

ACCAGGGAACATGCTTAACAAC 0.493498237386

ACCAGGGAAGCTGGAGAACAAA 1.03812650528

ACCAGGGCCTATCCAAGCTTAT 0.802805909658

ACCAGGTAGTCTCGAGAAAGAG 3.99763191775

ACCAGGTTGTGTCGCCGCATAC 0.342727555178

ACCAGTAATTATGCTTCACTAT 0.347109193842

ACCAGTCAAGATCAAGCGACAA 0.593990978044

ACCAGTCCAATTGCTGTTCGTA 0.806847956936

ACCAGTCGAGGGCATTGTACTG 1.37569542391

ACCAGTGCAAGTCAAAACTATA 0.399461210213

ACCAGTGCCTCTGCAACCTCAC 0.567327718286

ACCAGTTAATTTGATTCTGTAG 0.47256756611

ACCAGTTACGCCCCAACAATAA 0.554496388552

ACCAGTTAGCCCGCATTGAATA 1.14657432601

ACCAGTTCACCTCCCTCGAAAC 0.591809373189

ACCAGTTCGTTTCTAGTCGTAA 0.584111536623

ACCAGTTTGGCTCATTGACTTT 1.04344777833

ACCATAGAGGCGGCAAATGAAC 1.14628252403

ACCATATTATCTCATCTACAAG 0.479433788545

ACCATGTAGACTCAATACACTC 0.247183941875

ACCATTAGGTCTCCTCTGGAAC 0.447376250464

ACCATTCCAGGTGCTGGTAGTG 1.02141102962

ACCATTGTATCTCAGTCTCCAA 0.502652508506

ACCATTTCCAGTCAGCGTGGAC 0.633143304421

ACCATTTGATCTCCCGCAGAAT 0.174647928478

ACCATTTTGTAAGCAGGAGAAA 0.195790232592

ACCCAACTCACTGGTACTAAAC 0.243811259986

ACCCACGCGACTTGAGGCGCAA 0.637695491702

ACCCATCGATCTCCCGTGAGTG 0.649281478328

ACCCATGTGTGTCCTTAGAATC 0.289631445694

ACCCATGTGTGTGAAAGCTAAA 0.596437332665

ACCCATTGTTCTCCAACGTTAT 0.389463841021

ACCCCACAGCCTCCATTTTATA 0.698822738375

ACCCCACTCGCTGCTCGTTAAC 1.29333435422

ACCCCAGACAGTTGAAAGTAAT 0.55882050569

ACCCCAGGATCTGTTGACACAG 1.80010495404

ACCCCAGGGTGTGATGCCTTAT 1.90200130463

ACCCCATAATCTGCTGGATAAT 0.855532371411

ACCCCATACTCACTATGGTGAT 0.161246929936

ACCCCATATACTGCTTAGGCAT 0.354867223712

ACCCCCCTAATTCCCGGTGCAA 0.634644539812

ACCCCCTAGCCTCATGATAAAT 0.632934673266

ACCCCCTGACGTGCAAGAATAC 0.667266974123

ACCCCGCGAAGTCATGAATAAC 0.728032798851

ACCCCGCGATGTGCCTACAATC 1.33674688392

ACCCCGCTATCTGAATGACGAG 0.581804056744

ACCCCGGTATCAGCCCCCGGAA 1.65557859148

ACCCCGGTATGTCCTCCTGTAC 0.974752188672

ACCCCTCAATGTGGTAAGGAAG 0.891519720398

ACCCCTCTTATTGATTGTCAAT 0.43086403772

ACCCCTGTAGCAACTAACTAAG 0.692816145329

ACCCCTTAATCTCAATCAAAAC 0.61603775996

ACCCCTTACACTCTTGCAACTT 0.438605094208

ACCCGACACAATCCACGTGTAC 0.513560186735

ACCCGACTAGGTTCTGCGCAAT 1.13129290878

ACCCGACTATCTGTTTTAATAC 0.435633853008

ACCCGACTTTGTGGAGGCCGAT 0.433594289848

ACCCGAGACCCTCCGGGCTGAG 1.06586184521

ACCCGAGCATATTGTCAGTCAC 0.264029089794

ACCCGAGTCTTTCCACGGACAG 1.16925337166

ACCCGAGTGCCTCAGACAACTA 0.548240368354

ACCCGATACGCTGCTAGGTGAC 1.1994936418

ACCCGATATGATTCTCAGACAC 0.486307938906

ACCCGCCAAGATGCCGCAAGTG 0.785744172262

ACCCGCGAGGGTCACCACGAAG 1.03294652478

ACCCGGGTATGTCGTCTCCTAG 1.40894049883

ACCCGTGCAACTGATGCCTGAT 1.36341478118

ACCCGTGGGAGTGGTGTAATAC 1.08247635722

ACCCGTGGTTCTTAATGTCAAA 1.17956021855

ACCCGTTACTGTGTAGATGAAG 0.292310849814

ACCCGTTTATTTCAAAGAGCTG 0.219134077761

ACCCTACAGTCTAGATTCGTAA 0.392825628069

ACCCTACTGCTTGCTGGTCCTT 0.753917506632

ACCCTAGCGCGAGAAGAAGGAG 0.792221723801

ACCCTCGTGTATTCGCTTAGAC 0.777906750489

ACCCTGGCCTCTCCTTGTAAAC 1.0097644218

ACCCTTGCTTGTCCATTGCCAG 0.632059407188

ACCGATTAATCTGCACATAAAC 0.456232236562

ACCGCAAAATCTCAAGCGACAG 0.867768099805

ACCGCACAAAATTGTCCATGTG 0.395147787388

ACCGCACAGACTGCTGAGGAAA 0.798586251958

ACCGCACAGTGGCCTTGATGAG 1.49766893958

ACCGCACGACCTCTGAGATTAT 0.682805772969

ACCGCACTCGATCCAGCGTTAA 0.747298029324

ACCGCAGAAGATGATAAATCAA 0.614609902681

ACCGCAGTATCTGTTCCATCAT 0.807497748366

ACCGCAGTGGTTCGTGATGCAT 0.308326694584

ACCGCATCACCTCATCGGGTAT 0.918030659137

ACCGCATTTCGTGCTATCAGAA 0.531667075582

ACCGCCGTGGCTGCTATAACAG 0.86974595563

ACCGCCTCTTCTTCGTTTTTTT 0.737316890277

ACCGCGCTGCGTGTTAAAAGAC 1.46002581824

ACCGCGTTATCACGTGAGGTAG 1.30719166359

ACCGCTCACTCTGGTAGTCAAC 0.727563201278

ACCGCTCACTGTCATTGATTAA 0.539442432052

ACCGCTGCCCGTGCTATCAAAT 0.466422763517

ACCGCTGTTGCTCATAGCAGTT 0.191890987863

ACCGCTTGATATCAAAGGGAAT 0.476761386679

ACCGCTTTCACTGTAATCAAAG 0.37993332267

ACCGGACTGAATCCTGTCGAAA 0.321434242817

ACCGGATACAGTCCTCGTACAT 0.596936684156

ACCGGATATTATGGTACATCTA 0.534824043695

ACCGGCCTATGTCCCATTCGTG 1.01567407446

ACCGGCGTTGGTCCTCTTAAAC 1.56747924099

ACCGGCTCAGGTCCTTCCATAG 0.81060853291

ACCGGGACCAGTCTTAAAACAC 0.718340047695

ACCGGGTAAGGGCGGCGTAAAG 1.21931280826

ACCGGGTTCACCCATATCTGTT 0.580200025916

ACCGGTCAGACCGCTAGATATC 0.681498424201

ACCGGTCCCTCTGCTGCCGAAA 1.88711435629

ACCGGTTTATCTCCTTCCTGAA 0.184398519901

ACCGGTTTCACTTAAAGTCGAC 0.826803588743

ACCGTACTGCCTCCTTAGCAAG 0.372114372828

ACCGTTGGATGAGCATCGTAAG 0.492077892129

ACCGTTTCCAATGATTGGTATC 0.979627035507

ACCTAACAATATGCCAATGAAA 0.44729819088

ACCTAAGGACCTTAATGAATAC 0.495330302688

ACCTAATAGTGTTATTTGACGG 1.08028312863

ACCTAATGTCTTGAAGGTTAAA 0.583933450821

ACCTAATTGCATCATGTTTGAC 0.332451361604

ACCTACGACTGTGATAATTGTC 0.503309705398

ACCTACTTGTGCGATCGATTAC 0.383838876066

ACCTATGAAGCTTTAGGAGTAC 0.274106358589

ACCTATGGACCGGCATGTAATC 0.301757317712

ACCTATGTCTCTGCACCTAGAT 0.62937936514

ACCTCAACCACTGCTCTAGAAA 0.469926980135

ACCTCACAATGACGTACAATAG 0.759054820202

ACCTCACAGTGTCAGGGGAAAA 0.216626795714

ACCTCACAGTGTGATCATAAAG 0.446708360323

ACCTCACGATGTTTTTTTAGAG 0.718387216566

ACCTCACTCGATTCAAATCGTT 0.203848975209

ACCTCACTGGGTGAATAATTAG 0.30419118652

ACCTCAGCGTGGGCAGTGTAAC 1.03127493189

ACCTCAGTCGCTGCATTAGAAC 0.481340130032

ACCTCAGTTTGTGTTAGTCAAA 0.836974204368

ACCTCATAGCGTCACGTTGAAG 0.671284425893

ACCTCATGAGATCCTAGGTTTA 0.620534674288

ACCTCATTGAGTCGTGAAGTAC 0.391651314228

ACCTCCATACGTCTTACTTATC 0.75451166927

ACCTCCCAGGGTCAGCACCTAT 0.634619878426

ACCTCCGACTATCGATATACAA 0.749014982341

ACCTCCGCCTAGGCGGCCAGAG 0.992113999421

ACCTCCTCGTCTGCTTCTATAG 0.336808084688

ACCTCGCAAGGTGTCCCTTGAT 0.979602363334

ACCTCGCTATCTGATGGAGTAG 0.931144810911

ACCTCGTCGAGTCAAAGCTCAC 1.01694310987

ACCTCTCACACTTTTTATAGAC 0.257835271771

ACCTCTCACAGTATTTCATATG 0.477655154472

ACCTCTCCATCTCCTGCAGTAT 0.81084938096

ACCTCTCTATGTGGGAAGTAAC 0.324897689314

ACCTCTGAATCTCCGCCGCAAT 1.69133457773

ACCTCTGAATGTGGATGCATAT 0.815785891761

ACCTCTGCATGCGTATAGAGTA 1.01915909335

ACCTCTGCATTTCCCTAGCCAG 1.61244612799

ACCTCTGCCTATGATACTCAAC 0.571965671575

ACCTCTGTAACTTATTACTTAA 0.638435001271

ACCTCTTAAGGTCGGCTGTCAC 0.93442486193

ACCTCTTAGTATCTTAATTAAT 0.446837632081

ACCTCTTAGTGTGATCGGTTAC 0.469063687853

ACCTCTTCGGTGGGTAATGGAT 0.534129496985

ACCTGAGCGCCTCAGAGGGAAT 0.568571062113

ACCTGAGCGCGTCATGTACTAC 0.773238455383

ACCTGAGCGGGTGCATAATAAA 0.274268063487

ACCTGAGGCCGTCAAAAATGAG 0.65537051922

ACCTGAGTGTCTGCAGTGGGAG 1.14643073588

ACCTGATAGGGGGCTTGTATAA 0.735827654656

ACCTGATCAAGTCCCGCCAGAT 1.10807074878

ACCTGATTATCTCCTATCTTAG 0.349898871489

ACCTGCCTGTATGCGCTAGAAG 1.46002340807

ACCTGCCTGTCTTACTTCTTAG 0.30310214844

ACCTGCGAACGTGTCTAGATAG 0.223859874209

ACCTGCGCAGGTGAAACTTGAG 0.717333168088

ACCTGCTAATCTCATGACGAAT 0.250683345712

ACCTGCTTTTGTGCAGTAAGAC 0.876126268959

ACCTGGGCATCTCCAGGCAAAG 0.501063706452

ACCTGGGGGAGTCCTATACCTC 1.18970026637

ACCTGGGTACAGTCTGTAGATG 0.448599264112

ACCTGGTACTCTGATAAAGTAT 0.773380031709

ACCTGGTCGTCTAGCTACTTAC 1.55508300212

ACCTGGTGATGATTTGAAGAAT 0.672821311355

ACCTGTCAATTTGCTGCTTAAC 1.00901327196

ACCTGTCACATTCAAAGCGAAC 0.585923520864

ACCTGTCCATCACCACAAATAA 1.05762432639

ACCTGTGAAACTCTATATTCAC 0.20719874318

ACCTGTGAACCTCTAGTAAAAT 0.447997578631

ACCTGTGAGTGCTCTTTGTCAG 0.725851481988

ACCTGTGTATCTTAGACATGTC 0.521375257055

ACCTGTTCGCTTCGACATATTC 0.485460377961

ACCTGTTTAACGTCTGGCTAAT 0.795518159612

ACCTGTTTCACGGGGCGATAAG 0.79250828946

ACCTTACAATCTCAATCTGTTG 0.338270440147

ACCTTACAGGCTCACGAAGAAG 0.669151389499

ACCTTGTAATCTCCTTAGTAAT 0.637831169445

ACCTTGTTGAGTGGTGGTGAAG 1.04063744275

ACGAAAGGATGACACATAGAAG 0.403539278938

ACGAAAGGATGTCCATCAGCTC 0.570443889486

ACGAACTTAACTGTGGTTAATT 0.231188972688

ACGAAGGCAGTTCCTACTCGAG 1.13369787803

ACGAATCGTTATCACTGAGAAA 0.44106942348

ACGACACACTATCTCCTTTGAG 0.416879099768

ACGACACAGTCAGGTCCTTATA 0.989761057565

ACGACAGAATCTCCAGGAGAAA 0.527949503519

ACGACAGAATGTCATGCATTAA 0.438111834336

ACGACAGAATGTCCTTGTTAAC 0.87968717458

ACGACAGACGGTGCTTATAATC 0.441050304877

ACGACAGTGTTTGCGAGAGTTT 0.41687866986

ACGACATACTGTGTGCGGGAAA 1.54038629368

ACGACATTACTGGAATTCACAG 1.04375110657

ACGACCGCATGTGCAAAAAAAG 0.596848260335

ACGACGTAAGGGCCTAATGGAG 1.13497235497

ACGACGTAATCTGCATCTAATC 0.481987301792

ACGACTACTTCTGTGTGGATAT 1.27526963164

ACGACTATCACTCCTAGGACAA 0.327092067037

ACGACTCAGTTTGAACTATGTA 0.364601784066

ACGACTCGTCGTCAAACCACTG 0.496290659475

ACGACTCTCCACCGTTAATCAA 0.502827734312

ACGACTGAATTGGATGACAAAA 0.622968047121

ACGACTGTAAGTGATGTCATAA 0.767525167975

ACGACTTACGGTCTCTTTTCAT 0.322510110833

ACGACTTGCACTCGGTGCTTAT 0.870120512118

ACGAGACGGTCTGGTACTTAAT 0.255741999132

ACGAGAGAACGTCCTAATTCAC 0.662262342297

ACGAGAGATCATGCTCCTCAAG 0.324565609865

ACGAGAGATCGTGCTCCCCAAA 0.523823661532

ACGAGAGCAGTTGCGTTGTAAC 0.615291388529

ACGAGAGCTTCATCGAGTTGAA 0.734432614777

ACGAGAGGCTGGCATTGCGAAG 0.321701646183

ACGAGAGTAGATCATACTAAAG 0.308731615098

ACGAGAGTATGCGCTAAGGTTT 0.950890355034

ACGAGATTACCTGATCGAACAG 0.419998281972

ACGAGCAGCTCTCTAGATACAG 0.573513535651

ACGAGCGTATATGCCACATTAT 1.24359457968

ACGAGCTCATTTCATATGGAAT 0.388424219619

ACGAGGCACAACGTTCGAGCAA 0.70871795965

ACGAGGCCCACTCAACGACCAC 1.08083005514

ACGAGGTAGCCTGATGGCTTAC 0.847140166591

ACGAGTCGATTAGTGTTCTTAC 0.960340153412

ACGAGTGAAGGTCGCCAATTAT 0.936279302762

ACGAGTGGCTGAGAGAATGCTT 1.51941110963

ACGAGTTCAGCTGTATCCCAAG 1.56279743076

ACGAGTTTTTTACATGACATTG 0.504922473822

ACGATCTACTCTCCTTTGGTAG 0.652404641144

ACGATTCCCTCAGGTTAGGCAT 0.595504840137

ACGATTGATACTTATGTGCAAC 0.513534648812

ACGATTGGATGAGTTAGTGAAG 0.707056369199

ACGATTGGGGCTCCATGCGAAA 0.818948730511

ACGATTGTATCTCTTACGAGTC 1.13383016752

ACGATTTACGCTGAAAAATCAA 1.21148468039

ACGCAACAATCGCAACATCCTG 0.393460427092

ACGCAACCGATTCCTAGATTAA 0.468919835117

ACGCATTACGATCCTGCGTGAG 1.35250724446

ACGCCACCTACTGAATATTAAG 0.269104672301

ACGCCACGATGTCAATGTACAC 0.750409135913

ACGCCACGGTGACCTACGCGAT 1.73250436913

ACGCCAGAAAGTGAATAACTAA 0.5896522603

ACGCCAGACTCTGGATCGTATT 0.289245215232

ACGCCAGCATTTGACTGATTAA 0.521720415935

ACGCCAGTAACTTAACGCCTAA 0.622004314486

ACGCCAGTTAATGATGCTCATC 0.474786575944

ACGCCATCATCTCAAAAAGATT 0.262795143936

ACGCCATCCTCTGCTTTACCAG 0.980035750394

ACGCCATGAAGTGATCAAGCAC 0.925443763062

ACGCCATTTTGAGTTAGCTCAT 1.10031276642

ACGCCCCGATGAGTACCCCTAG 1.96661185161

ACGCCCTTTTGTCATAGGCATT 1.06510261372

ACGCCGGTCGATCACAAACTTA 1.52592660026

ACGCCTCAATCTGTTAAAGCAG 1.0514888323

ACGCCTCAATGTGCTAGCTAAC 1.55496306739

ACGCCTCCCTATGAGGAGATTT 0.504411154916

ACGCCTGCCACGGTCGACTTAA 1.06485742096

ACGCCTTGATGTGTAGGCGGTT 0.439563252207

ACGCGAGGACCTCTTTCATCAA 0.757172704514

ACGCGATACTTTGATAGTAAAT 0.505508854768

ACGCGCGAATGGCCACTTAGAC 1.04272466601

ACGCGGTGTTGTGCCTTTGCAC 1.33572699307

ACGCGTGCGTGTCTAAATGGAG 1.13035180144

ACGCGTTGCTTTCGTTCCAAAT 1.66300557739

ACGCGTTGTGCACCAAGCCAAC 0.574023200287

ACGCGTTTATGTGCTTGCAAAG 0.985971114944

ACGCTAGGATCAGCAGGGACAG 1.04427452473

ACGCTAGTCACTTCACGTGATG 0.978404024616

ACGCTATCTTGTGTTAGCTTTT 0.481836667555

ACGGATCAAAGTGATTGTCCAC 0.568641006687

ACGGATGTCGCTGAGTAGTTTC 1.69080340025

ACGGATTTATGTCAGGGTGTAT 0.590328977786

ACGGCACTAGTTCTTGCATAAA 0.338686099396

ACGGCACTGTTTGCATCCCAAG 0.725641524345

ACGGCAGAAGGTGACGTGCGAT 0.909073632855

ACGGCAGGCTCTGATTGTGAAG 1.51780440352

ACGGCATGATCTCAGCCTGAAG 0.521999622773

ACGGCATTAAGTCATATCCAAC 0.393278239445

ACGGCCCAGTGTCCAATACTTA 1.45613883516

ACGGCTGAACTTGATGCGCAAA 0.880479380979

ACGGCTGAATGTGACTACTTAA 0.85836876522

ACGGCTGGATCTGCGGAACGCC 1.89337732996

ACGGGACTGTATGATATGGTAT 0.683265634712

ACGGGAGCGTTTGCTACTCTAC 0.84981318972

ACGGGATACTGTGCATAGGGTG 0.236799065952

ACGGGATTCCTCCATCTGTGAA 1.3490592999

ACGGGCGAATCCGATCCGTTAC 0.802314124818

ACGGGTCCATATCAGGTGCTAC 0.811135686396

ACGGGTCTTCGTCATGCGCCAC 0.799953744716

ACGGGTGACTGGCCTCCGAATG 1.29582686926

ACGGGTGTCACTGCCCCCCATA 0.533225278688

ACGGGTTGCTAACCTGTGTGAC 0.940037965172

ACGGGTTTATTTGTTTTCGTAC 0.841597900241

ACGGTATACTCGCATAACATAC 0.718785920422

ACGTAACAACCTCAAACTTTAC 0.345255239543

ACGTAACTCTCGTCTTTATGAG 0.284943072554

ACGTAAGACTCCTATCATGGAG 0.525625461463

ACGTAAGGCTCTGCTAAATAAT 0.2544752863

ACGTAATTAGGTCCATCCGGAG 0.455634921238

ACGTAATTGTATCATCTTTGAG 0.792995974487

ACGTACGTGAGTTCATGGTTAA 0.564843881243

ACGTACTAATCTTCTAGATAAT 0.50835697293

ACGTATCTCGATGTTAGGCTAT 0.486841310394

ACGTATGATTGTCATAACACAG 0.708776187619

ACGTATGCGACACACGAATGTG 0.979418633203

ACGTATTACTCTGAAGGCGAAC 0.532442587291

ACGTATTCGTGTCCTACGGTTG 0.535026788967

ACGTCACGCTCTGATAGGGTAA 0.854320126981

ACGTCAGAAAGTGATATGAATA 0.407795086369

ACGTCAGAATATAATAAGTGAC 0.521666536617

ACGTCAGCCATTCGTTGCTAAA 1.03105594751

ACGTCAGGAGCTGAAGACCTAA 0.215377945696

ACGTCAGTAAATGGTTACATTC 0.924410604313

ACGTCATAATCTGAGCAGTGAT 0.404319682837

ACGTCATAGTATCCTAGATATA 0.620295593674

ACGTCATAGTCTGCATCCCAAC 0.545577322401

ACGTCATCAACTGCAACAGAAC 0.440262506497

ACGTCATGGTAGGCTTTATAAG 1.33874064429

ACGTCCCTATTTCATAAGAAAC 0.255093985428

ACGTCCGAACGTTAAGCATTAG 0.513205455182

ACGTCCGACGGTGTGGCACTAT 0.636083570431

ACGTCCGACTTGGCTATAAAAT 0.703476546655

ACGTCCGAGTGTGGTGGGTTTA 1.8543181587

ACGTCCTAGACTCCAGATCTTT 0.637304684827

ACGTCGCTAACTCATGTTCCAG 0.721915810568

ACGTCGCTATGCCATTAGTAAT 0.733246735928

ACGTCTCCATATTCAGGTTAAG 0.957728245484

ACGTCTCCCTCTGCACTAACTC 1.61686875864

ACGTCTGAAAGCCCTTCCATAC 1.04505446468

ACGTCTGATTCTCAAACACAAC 0.69654592631

ACGTCTGCGGTTGCATCCTAAC 0.832289704758

ACGTCTGTGAATGCTCGTGTAG 1.50700559191

ACGTCTTAAAGTCCTACCCGAT 0.30612688707

ACGTCTTCATGTCTTACCCAAG 0.884607034891

ACGTGACCATATTGAGTTCAAA 0.486711847053

ACGTGACTCTCTCCAAAGTGAT 1.13307877316

ACGTGACTTGCTGCTACCGAAC 0.99817538913

ACGTGACTTTGTGCTATTGAAA 0.266150027504

ACGTGAGAAATTGTTAGCGCAG 1.69611036904

ACGTGAGAACGTCTAATAGAAG 1.09507013702

ACGTGAGCGTGTCTTTGGCGAT 1.26483424848

ACGTGAGTCGCTGCAGGAGAAG 1.01893601056

ACGTGATCAGGTTAAGGTGGTA 0.901793756854

ACGTGATGATTTGTTCACATAG 0.810946931893

ACGTGATTATCTCCTACTGAAC 0.573956594991

ACGTGATTTTTTGATAAGAATC 0.347588123874

ACGTGCTAATGTGATATAAGAG 0.53265312069

ACGTGGGATCGTCATGAATAAG 0.773997497311

ACGTGGGTAGGTCAATCCAAAG 1.43096724795

ACGTGGTAAGGTGCGAGTGATG 1.77577861565

ACGTGGTTGCGTCTTCCTGCTC 1.76922650542

ACGTGTGCCATCCCTAAGCAAC 0.663985418446

ACGTGTGTTTTAAGAGGCTGTA 0.650291461314

ACGTGTTATGGTGATTCGCCAC 1.81562890848

ACGTGTTCATGTGCTACAAAAG 0.302644159101

ACGTTAGTATGTCCAGACAAAG 0.290650171339

ACGTTAGTTTCAGACGGGCGAT 1.18677612893

ACGTTATCATTTCACGACTCTA 0.536523686365

ACGTTTTAAGCTGTGTGTAGAG 0.608649869832

ACGTTTTTCTGTGGGGACGATT 2.13842987679

ACTAAAGGATTCCGTACAGTAA 0.361271240757

ACTAAGCTGTCTCAATAGAGAA 0.250390356325

ACTAATCAATCTGCTTAGGCAA 0.378194741167

ACTACACAATAGCTTCATTGTC 0.463195605498

ACTACACACCATCGCTGCGCTT 0.639409850185

ACTACAGCATATGATGCGTTAG 0.743255599639

ACTACAGCGGCTGCCTGAATAT 0.603652876345

ACTACAGTCTCGGCTATCAAAA 0.798186798434

ACTACCATCCCTGTAAGAGTTT 0.374275717873

ACTACCCAGACAGCTAGATATA 0.467541849805

ACTACCCTCTTTGTGCTAACAA 0.526967298837

ACTACCGAAAGTCACCTGGGAA 0.474688039661

ACTACCGTATGTTTAAAATGAA 0.551223857489

ACTACGGTAAGTGGTGCTCCAG 1.29023235713

ACTACTCAGGTTGATCTTCATC 0.431022076945

ACTACTCTACGTGATTCAAAAG 0.635937488025

ACTACTCTTAGTGGTACATTAC 0.901728693371

ACTACTGTGAGTGCCAATTAAC 0.849446206933

ACTAGAGAACCTTAAATGTAAG 0.658396842267

ACTAGCCCGTATGCGGGCTAAA 0.580877162067

ACTAGCTTGTCTGAAGGAACAG 0.633114654567

ACTAGTCATTTTGATTCTCAAT 0.328662310769

ACTAGTGTAGGAGACTGCGAAC 0.643360217712

ACTATATAGCATGGATAAGGAG 0.788252093897

ACTATCTAAGCTGAAGCATAAC 0.274212822341

ACTATTCCGTTGCTAATAACAC 0.598392558522

ACTCAAAAATGTCATACCACAA 0.794751423463

ACTCACTTCTGTCCTCCATATA 0.584248804944

ACTCCAGTCTCTGTGAACTCAG 0.668143884434

ACTCCAGTTAGTGGTGGAAGAA 0.418956895262

ACTCCATCCTTTCAAGACTAAT 0.453768788117

ACTCCCGCATATGCAGGTCAAC 0.985628402843

ACTCCCTTAAAGCAGACGTTAC 0.687590255517

ACTCCTCTGAGTGCTCAGACAG 0.590157901885

ACTCCTGGCTCTGCAAAGTTAC 0.576927185172

ACTCCTGTATCACCGAATTCAT 0.401145726274

ACTCCTGTCCATTTAACGGAAG 0.902958610594

ACTCCTTAATCTAACATCTTAA 0.253872741168

ACTCGACGAAGTTCAGACAGAG 0.960686966181

ACTCGACGTTGACATGAAGAAT 0.8137712372

ACTCGCCGAGGTTGTGCCGAAG 4.07718604578

ACTCGGATAATTTCTTTTAAAG 0.628173771638

ACTCGTGAAAGTTTTCGAGAAG 0.70171856482

ACTCGTGAGTGAGATAATGCAC 0.303918829586

ACTCGTTACACTGCTTCGAATT 0.415504031625

ACTCGTTACTCCGTAGAGTTAG 0.260399477816

ACTCTACCAAATGTCCGTTAAC 0.526824074988

ACTCTACGGCCTCAACCTTTAC 0.66483233437

ACTCTATATTTTGTATGTGAAC 0.432992153677

ACTGAACACGCTGGAGCTTAAC 0.674548149203

ACTGAAGAATATGATGGGTCAA 0.327939005408

ACTGATTGTTTGTCGAGCTAAG 0.882589473608

ACTGCAACATCTGATCGGGAAG 0.310898116155

ACTGCTCAGTCCGAAGCATTTG 0.249117826432

ACTGCTGTAACTCCTGGGAGTC 0.752082740547

ACTGCTTAAAGTCGAAGCTCTA 1.18601243098

ACTGGAACACGATCATAACAAC 0.5397396404

ACTGGGGAATACCTTTTGGAAA 0.584421804646

ACTGGTGTGGCTTCACTCGAAC 1.37635030749

ACTGGTTGTACTCTTCCCTTAC 0.847301948715

ACTGTTGACTCTGTGGATTATA 0.877338161665

ACTTCACAAAGTCCTATTCGAA 0.297802717587

ACTTCACGTAATGATGGGTGAT 1.08566860469

ACTTCATCTTGTCGGAAACAAC 0.478365103919

ACTTCCGGAAGTGTTTGATGTG 1.1107916594

ACTTCGTAAATTGGACTTTAAT 0.165932811798

ACTTCTCAAGCTGCTCATACAG 0.399806866559

ACTTCTCGATGTCCATGGTAAG 0.589766934355

ACTTCTTAGTCTGTTTCGCGAC 0.869786845422

ACTTGACAATCTCATCATCCTG 0.189263330256

ACTTGACAGCGTGCTCCACCTA 0.774805399586

ACTTGAGATTCTTTTGAGAGTA 0.414836623543

ACTTGATAAGCGCCTTAACCAG 0.32352905635

ACTTGATTAGCTCGTTGATCAG 0.226982529031

ACTTGCTTGTTACTAGGGAGAT 0.758556871113

ACTTGGTATGACCGTTTCGTAA 0.589168742011

ACTTGTTCCAATTATGAGGTAC 0.295720212588

ACTTTAGACTGTGAAGAGCTAT 1.82990145886

ACTTTGGAATATCTTTTAAGAC 0.346378244221

AGAAAGATACATCATGATTAAC 0.595118058639

AGAAAGGATACGCGTATGCAAT 0.777829901217

AGAACTAAAATTGATGGTCCAA 0.6426126078

AGAAGAGTCTGTGCAAATAGTG 0.333949368004

AGAAGTTGGGGTGATATCCTTA 0.471549820787

AGACCATACTCTCCTGCCGGAG 1.31088111741

AGAGCAAAGGATTCTGTCGGAC 0.530066473899

AGAGCACTAGTTCTTCGTAAAG 0.16297966183

AGAGCTCAATCTCCATCCGGTA 0.896590457848

AGATCAGCCTGTGCAAACCTAC 0.539673649875

AGATGGCAGAGTCATTAGTTAG 0.276139525589

AGATGTGTAGCGCATCAGCTAC 0.661641578017

AGATGTGTTCCGGATTATTCAT 0.633054073669

AGCAAACAAGCTCCTAACATAA 0.164563422087

AGCAAACTATTGTCTATCGAAG 0.124973194231

AGCAAAGACTCTGAAAGAAAAG 0.279279837576

AGCAAAGATCCTTCTATCTGAG 0.47426883518

AGCAAAGGCTGTGCTGATTAAT 0.522064267185

AGCAAAGTTACTGCAGGTGTTA 1.08501090942

AGCAACCAATAAGTCGTAGCAC 0.38159450119

AGCAACTGAGCTGCCTCGTAAG 0.870664999299

AGCAAGGACTCTTTTTGGCGAG 0.244602005771

AGCAAGTAGACTGCTCGACTTA 0.36939402942

AGCAAGTTCGATCTTCCTTGAT 0.590522945083

AGCAATCACCCTGATCCTCAAG 0.991884264502

AGCAATCATTCACAATCCCAAT 1.07671354941

AGCAATCTGCGTCGTGATTATC 0.355358282612

AGCAATTAGACTGATAGAAAAA 0.6004897157

AGCACAAGGACTCTTTACTATG 0.270350548382

AGCACACAATGACATGCTGGAA 0.802203343007

AGCACACACTCTCCTAGAATAC 0.428112404633

AGCACACGAAGTGTAATAAATC 0.41255121028

AGCACACGATCCCATACATTAC 1.02735777316

AGCACAGAACTTCCCGCGGTAC 0.68190687833

AGCACAGAATCATGAGTGTCAA 0.55819804915

AGCACAGAATGTGAGGCTGTAG 0.501973266311

AGCACAGAATGTGCTAAGGAAC 0.470343433045

AGCACAGACTGTCAGAACATAA 0.520875462495

AGCACAGACTTTCCGCAATGAT 0.770370847653

AGCACAGCCGTTGAATTACTAG 0.751592249709

AGCACAGCCTATGAATCGCCAG 0.257729608065

AGCACAGCTTCTGCTCCCGGAC 0.205252222567

AGCACAGGAGTGCGGAGTTTAT 0.342136835023

AGCACAGGATCTGGGGGCGAAG 1.58188670297

AGCACATACAGTCTCGAGCGAT 1.20285739597

AGCACATCATGTGCTCCGATAT 0.917154288226

AGCACATGGTGCGTTGGCCTAG 0.432957571242

AGCACATTCTTTCATGACGTTT 0.281942728958

AGCACCCCGTTTGCAACTGGAA 0.210292656007

AGCACCCGAAACGGTAACATAT 0.751634508479

AGCACCCGGCAAGCTTTTCCAG 0.788624832728

AGCACCCTCTGTTAAGCGGCTG 1.60132571838

AGCACCGACTGTCCCAAATTAT 0.915435864061

AGCACCTGACATCCACTTAGAG 2.86611503926

AGCACCTGCTGTCTTCTCAGAA 0.715424034179

AGCACGCACGCTGATCTACCAG 1.95289690138

AGCACGGAAAGTCAGACAGATC 1.01019246115

AGCACGGCGACCCTTGGTTAAT 1.02221591208

AGCACGGTCACTGATGCTCTAG 0.848479637118

AGCACGTTTTCTGCATTGGATG 0.357158572097

AGCACTCAAAATGATGGGTCAC 0.369421292774

AGCACTCACTGTGATTAGATAG 1.45623567217

AGCACTCAGAGTCGCCAGATTA 0.641432595735

AGCACTCCAATTGTACAAGGAC 0.841055258543

AGCACTCTAAGTGGTCTTGCAT 0.155794200823

AGCACTCTATGTCCAAAATGAT 0.193102584309

AGCACTCTTAAACATATGTAAT 0.206064415796

AGCACTGAATCCGATGAGGTTT 0.315120167139

AGCACTGACAGTGCTGAGACAT 0.511540852746

AGCACTGTATTATCGGACGGAC 0.290398147978

AGCACTGTCTATCAAAGGCAAG 0.544969057465

AGCACTTCAGCTCTTTCCCTAG 0.776305512155

AGCACTTCCCTTCTAACTGTAT 0.379995283464

AGCACTTCTCGTGGAGCATAAG 0.805432874056

AGCACTTGATAGGCTTGCTGAC 1.08503850829

AGCACTTGGTATGTTATGCAAC 0.385138227068

AGCACTTTCTATGATTCCCTAT 0.091710314378

AGCAGACACTTTGCACAATAAC 0.844172456899

AGCAGACTCAATGCTTAAAGTG 0.874660014988

AGCAGAGAATGTCTTGTAGTAG 0.918426117941

AGCAGAGACGGTGAGAGACTAT 0.960688512419

AGCAGAGGAGCTGATATTGAAG 0.646557736793

AGCAGAGGCTCTGTTGCGTAAA 0.739181329136

AGCAGAGTACAATTGCTATAAA 0.543204426885

AGCAGAGTAGCTCGACTGTCAA 0.276886317984

AGCAGAGTGTGAGCTCGAGCAG 1.80029967415

AGCAGAGTTACTGTTAGATTAG 0.573031246663

AGCAGATAAACTCCTAAAAAAG 0.394565916865

AGCAGATAAGGACTGTCAATTC 0.493270139181

AGCAGATAATATGTGTATGTAG 0.784026273787

AGCAGATAATCTTATGGCTAAG 0.258389632214

AGCAGATACTCTGGACTAGAAG 0.670821307598

AGCAGATCATATCATGCATGAA 0.679167994532

AGCAGATGGCATGCCGAAAGAT 0.445100787561

AGCAGATTGTACGCTGGTTGAG 0.724116994226

AGCAGCCAAAGTGTTTATCAAC 0.556677693473

AGCAGCGATGGTCTTCAAGCAC 1.91150049939

AGCAGCGTATGTCGTGCATATG 0.562832925225

AGCAGCTCGTTTCATTCTGTAT 0.912652800567

AGCAGGATCACTGCAGAAGGAT 0.883108286472

AGCAGGCTGTCTGCTCGCTTTT 0.875361304597

AGCAGGGCAGGTCGCTAGGTTC 1.39332540652

AGCAGTAAATTTGTATAATCAC 0.506060026101

AGCAGTAGATGTGAACAAACAG 0.450090113452

AGCAGTCCGAATCTTTTTTAAG 0.3310202571

AGCAGTCGGTTTTACGCAAATT 0.579947410208

AGCAGTCTCGCTGAAGGCATTA 0.968644929301

AGCAGTGAACCACTCTATAGAT 0.928070205376

AGCAGTGACTTTGAACGGTAAA 0.2625694051

AGCAGTGTGTTCCATAACTTAT 0.223360907151

AGCAGTGTTCCTGCTGCTGTAT 1.10874128643

AGCAGTTAAGCACGAACAGAAA 0.723619164712

AGCAGTTAGTCTCCCTCCTAAC 0.984829989859

AGCAGTTCACCACGTCGATCTG 0.350913363404

AGCAGTTCATCTTACAGGTTAC 0.644614426418

AGCAGTTGGTGTCAATGCAGAC 1.34947694974

AGCAGTTTGCTTGAGCTAACTA 0.568910959098

AGCATACAGTTTGGTATCTAAA 0.586645535741

AGCATAGAAACTTCCTTTGAAC 0.579208014192

AGCATAGGCTGTCCGGATACAG 1.84538752925

AGCATATGATCTCCTAAGTCAG 0.652737307505

AGCATCTAGTCTCGCCAATGAT 0.640380314014

AGCATCTTCCGTCCACGAGATG 1.44558309695

AGCATTCAAACTGCGTTACTAT 0.3984980097

AGCATTCCCTATGTTCCGTAAC 0.630629115172

AGCATTGAGTCTCCCGGACGAT 1.21467347636

AGCATTGTCTGTTGATTTAGAT 0.273876564667

AGCCAATACACTGATATTGGAT 0.46496034132

AGCCAGTCCCCTCCAGGTTGAA 0.953426059036

AGCCATGAATATGCTGGTGAAA 0.433761890563

AGCCCAAATTCAGATTCCTTAA 0.656295474994

AGCCCACACTGTGAGTACTTAG 1.3057103878

AGCCCACGTAGTGTTTCTCTAA 0.641629092091

AGCCCACTTGTTCCTTGGAGAG 0.350031894891

AGCCCAGAACCTGTATATGAAT 0.401497028632

AGCCCAGAATCTCATGTATGAA 0.574165561225

AGCCCAGACCCCTATATTAAAT 1.13898836689

AGCCCAGACGTTCCAATGTCAA 0.6279515629

AGCCCAGACTGTCATCGGATAG 0.823019199399

AGCCCAGAGTATCATGACAGTT 1.20526365412

AGCCCAGTATATGCTTATGAAA 0.506572428443

AGCCCCGACCCTGATTACGATA 1.15325655668

AGCCCCGCGTTTAAACAACAAC 0.767858324483

AGCCCCTTAACTGGAGATCATG 1.02264630663

AGCCCTGAATATGCTTGTTGAA 1.48448579557

AGCCCTGACACTTTAGGATAAC 0.587699932854

AGCCCTGTGGCTGGAAGGATAC 0.860305688189

AGCCCTTACACTCTTGATTAAA 0.813748704565

AGCCCTTCACGTCATCCGTTTG 0.610788810763

AGCCCTTCATGTCGTACTGCAA 0.861158077519

AGCCCTTTTCGTGAACGTAAAA 0.293619744224

AGCCGACAATGTGATTGGCTTG 0.166601565644

AGCCGACGATGGGAACGGGAAC 1.37746458078

AGCCGACTCAGTCACGTTCGAT 0.563495827862

AGCCGACTGTCTTCTGAGAAAC 0.359923568128

AGCCGAGAATGTCCAGAAATAG 0.371176251507

AGCCGAGAGGGTGCAACGTAAA 0.441336255411

AGCCGAGAGTTCCACTAGCTAC 0.399638104775

AGCCGAGGATGGGCACAAGCAT 1.57383397947

AGCCGAGGCGCCGTTGCTCCAG 3.86037925449

AGCCGAGGCGCTCGTTACCATA 1.46218581423

AGCCGAGTAGCTGCAATCACAT 0.87835966358

AGCCGAGTGAGTGCAGTCTAAG 1.78743094153

AGCCGATAGCCTGCTATCTCAT 0.4939375031

AGCCGATATTGTCAATTGCCAA 0.601299460823

AGCCGATTAGACCTTTAATCAA 0.564950202038

AGCCGATTTGTTGATGACTGAA 0.790590684322

AGCCGCATCGCTGATGGTAGAT 1.33641770692

AGCCGCTGGTCTTATAAGGCAC 1.88759942497

AGCCGCTTAACTGTTGGGGAAC 2.09068955856

AGCCGCTTGACTGATCTGGAAC 1.47046589238

AGCCGTACGGTTCACGCGTTTC 0.979995795192

AGCCGTCACGCCCAATCGTTAG 0.930920686867

AGCCGTGCATGTGGTGCCTAAG 0.53181646264

AGCCGTGGGAGTCTACGCTGAA 1.75103093581

AGCCGTGTAGTTGCTAGGGAAA 0.168367206422

AGCCGTTAGTTTCCAACCGGAA 0.58036474032

AGCCGTTCCGATCATGCTGGTA 0.705900024344

AGCCGTTTAACTGTGCACATAC 1.4448851099

AGCCGTTTCCCTGATCACAGAT 1.15861907185

AGCCTAATATATCAAGAGAAAC 0.58106448406

AGCCTAGAATCTCCTGGTCGTA 1.36221327073

AGCCTAGGAGGCGTTTCGTAAG 0.967993122091

AGCCTAGTATGTCATGATTTAA 0.419259526134

AGCCTAGTGTCTGCAGAAGAAG 0.911404783919

AGCCTATAGGCTGGAAGTTAAT 0.521700894627

AGCCTGGAGATTGATATGTGAC 0.556142668995

AGCCTTTTATTATATCGGTAAC 0.961868649816

AGCGAAGGCTTTTATGAGTGAG 1.89552311058

AGCGACGGGTCTGTGAGGTTAA 0.591612814054

AGCGATCTTTGTCGTACCATAT 0.635068369653

AGCGATGAATCTCTATAATCAG 0.397098154546

AGCGCACCATGTGCTGCCACTC 1.22399479466

AGCGCACTAAGTGCTGGAAAAA 1.55228821653

AGCGCAGAGGGTGATATTCAAT 0.63523247529

AGCGCAGTAACTCCGTAAACAC 0.230005925653

AGCGCAGTATCCGCATGATAAG 1.38583055414

AGCGCAGTATGATATAAAAAAC 0.210458540441

AGCGCATAGTGTGCAGGTAGAT 0.544203167465

AGCGCATATTTGCTTGGCTCAG 1.1338875888

AGCGCATCAGGCGATGCCATTC 1.22902105165

AGCGCCACAGTTGGACATCAAG 0.461440457719

AGCGCCCGACGTCCCCAGAGAG 1.32745364199

AGCGCCTGCTCTGCTTTGATTT 1.21898305562

AGCGCGCACTCTTCAAAGGAAG 1.64962885801

AGCGCGGACAGTCATGAACGTC 1.69543699814

AGCGCGGGTGCTGGAACTAGAA 1.98125786589

AGCGCTCACCGTCATAGTCGAA 0.806708850875

AGCGCTCACTCTTATTACTGAG 0.824003665213

AGCGGACACTCTGCAATCATAA 0.693359000662

AGCGGACGTAGTCTAGAAATAG 0.752846583254

AGCGGACTATCTGCTCCGGCAG 1.2350275602

AGCGGAGAAAATGTTACTCAAA 0.588178203245

AGCGGATAGACAGGAGGTGTAT 0.879043004098

AGCGGATAGGGTGCAAACTATC 0.581544025532

AGCGGATGTCGTGGTAAATCAC 0.907011000694

AGCGGCAACTCTGATACTATTC 0.466390760788

AGCGGCGCATGTTCTAAAGTAG 0.347555783029

AGCGGGCTAATTGTAGATGAAG 1.27177543727

AGCGGGTCAGGTCAAAGTAAAG 0.76233152197

AGCGGTAAATCGTCTCCATCAA 0.710471439615

AGCGGTACAAGTCAGGGGGGAG 0.786756112226

AGCGGTCACTGTCTACGGTATT 0.599422461973

AGCGGTGTACGTCTACTACTAC 0.853528693507

AGCGGTTAGTCTCATGCAGAAT 1.30032251556

AGCGTACCTCTTCGTTAATCAT 0.815188496475

AGCGTGTAAGGTCAAAGATCAG 0.393803354241

AGCTAAACGTGAGTATTCAGTC 0.551554738825

AGCTAAGAACTTCAAGGAACAC 0.547672544988

AGCTAAGAATATCAATATAGAA 0.271805390066

AGCTAAGAATCTGCGCGAGTAG 0.69283888675

AGCTAAGAATCTGTTAATTGAG 0.215639483503

AGCTACGACACTGCAGTGTTAC 0.345942799372

AGCTACTCATTTGGTAAGCGAG 0.928114247852

AGCTATACATGTCATCTCGAAA 0.420977399362

AGCTATCTGTAAGCTTAAAGAT 0.390345245353

AGCTATGACCGTGCCCTCACTA 0.156595981987

AGCTATGACTGTCCATAAACAG 0.323088827551

AGCTATGAGCGTCTTTGGATAG 0.173952880363

AGCTATGGGTATGTTTTGAAAG 0.57101839456

AGCTATGTATCACTTGGTCGAC 1.06382375274

AGCTCACATTCTCTTCCATTAT 0.419361323821

AGCTCACTTTGTCGGATGCGAC 0.650609249083

AGCTCAGAACGTCCATCCTTAG 0.289491343054

AGCTCAGACGCTGTACGCCTAC 0.182539356661

AGCTCAGACTCTGAGCGATAAG 0.659648358687

AGCTCAGAGCGCGAAGGTCAAT 0.459497395995

AGCTCAGAGGCTGAACCCTAAA 0.306433590664

AGCTCAGAGTCTTAAAAAGATG 0.860788203421

AGCTCAGCCCATTCTAACCGAA 0.242614311623

AGCTCAGGTGGTCATTCACAAG 0.821945800434

AGCTCAGTGAAGGCTTCAGAAT 0.658294600704

AGCTCATAATTTGGTATGGTTG 0.226629376702

AGCTCATACTATGATCACGCTC 0.582482230395

AGCTCATGAATTGTTATTAATG 0.459307394707

AGCTCATGACGTGATTTGCGAG 0.956787407186

AGCTCATGCTGCGAAGGCCTAA 0.595011763573

AGCTCATGGGGTCGATATGTTT 0.419876819379

AGCTCATTAGATTCATGTCCTA 0.870518444934

AGCTCATTATATCGTCTTGAAA 0.308030832527

AGCTCATTCCGTCTTAAGTAAT 0.62191143287

AGCTCCCAATCTGACGGATCAG 0.917202211992

AGCTCCTCCTGTCAATGGAGAG 0.780417871954

AGCTCCTGCTATCCTTAACCAT 0.514117406808

AGCTCGCACTATTCAGAATAAG 0.67006519446

AGCTCGTACTCTGATAAAAAAC 0.531850119534

AGCTCTCAAAGTGAACGGTGTA 0.540329345263

AGCTCTCAGTATGAGTTGTGAT 0.692566333781

AGCTCTCGATGGGTAAGGCCAT 0.965888184749

AGCTCTCTCGTACGTTCCGTAT 0.686780357011

AGCTCTGAAGATGATCGGGGAA 0.625534042705

AGCTCTGAATGTTGTGGCCTAG 1.98881242208

AGCTCTGCATCTGGTCGAATAG 1.14790717576

AGCTCTGGCGGTGGTTGAGAAC 0.568516949477

AGCTCTGTAATTGCCAATCCAA 0.742670641924

AGCTCTGTAGATGCTCAATATA 0.335048835343

AGCTCTTAGGGTGAATAGAGAG 1.58183398585

AGCTCTTAGGTTCCTAATAAAA 0.406421349407

AGCTCTTATTATGCCGACTAAC 0.698273256104

AGCTCTTCATCCCATTCTCTAC 0.730221332376

AGCTCTTCCCTTGTATCAAAAG 1.0245614187

AGCTCTTGAGGTGGTCCGCAAA 0.435318874583

AGCTCTTTACATGCTTCCCCAA 0.518152807852

AGCTCTTTGTGTGAGTCCTAAG 0.767706960784

AGCTGAAGGGCTCTATCGATAT 0.360835864655

AGCTGAATATAGCAATGTCAAC 0.437727250263

AGCTGACACATTCTATTACATT 0.555426702511

AGCTGACAGTTTTCATTCTAAG 0.245362296093

AGCTGACGAGGTGTAACGTAAT 0.728016996017

AGCTGACTCGGTTAACCACAAT 0.660441924544

AGCTGAGAATCAGATTGCAGAA 0.588231944465

AGCTGAGAGTTGAGAAACAAAC 1.02965038083

AGCTGAGATTCTGACTGATTAC 0.329860447046

AGCTGAGCAGGTCTAATGTTAA 1.33777887437

AGCTGAGCATCTCCCCCTGTTC 0.325512675738

AGCTGAGCGCATTAATCGGTAG 0.774202613259

AGCTGAGGCTCTGCATCAAAAC 0.42925988046

AGCTGAGGCTGTGTTGGTTCAG 2.52190477216

AGCTGATAATGGGGTTAGGCAA 0.835093272744

AGCTGATACACTGAATACGAAC 0.225408481942

AGCTGATACGGTCGTTTCTAAC 0.943657770912

AGCTGATAGTCTCAAAAGTCAA 0.38115224141

AGCTGATGACGTTCTTATGTAT 0.341916706074

AGCTGATGCGGTGACCGGCAAG 1.69015003395

AGCTGATGCGTTTTTTGAAATT 0.713043047663

AGCTGCCACTGTGCAGGGGTAT 1.90415138121

AGCTGCCGGAATCTGTTATAAC 0.975257672042

AGCTGCCTGTATCCATGCTCAA 0.65601540027

AGCTGCCTTCCTCTTCTCGAAA 1.59583634807

AGCTGCGTATGAGAAACTACTG 0.731481908149

AGCTGCTAGTCTCCGTTCGCAG 1.73306286025

AGCTGCTCGTATGATGCTGCAG 1.2114369826

AGCTGCTCGTCTCATTCCAAAT 0.670725553389

AGCTGGGCGTGTCATATGGAAC 1.3277053133

AGCTGGGTCTCTCATATCCTAA 0.617167208521

AGCTGGGTTAGTTATGCGAAAT 1.14998294251

AGCTGTAAAAGTGGTATACTTT 0.32222718041

AGCTGTCCGTATCCCTGCTTAG 1.64489664891

AGCTGTCGAACTCATTGGAGAC 0.924978776194

AGCTGTCGGACACTAAACCATA 0.876262503754

AGCTGTCGTTCTGATACGTGAA 0.430283052846

AGCTGTCTCCGGGACGAATCAG 0.223182083808

AGCTGTCTCGGTGCTCGACGAA 1.56853230618

AGCTGTGAGTGTGTTTTCGAAT 0.771925205362

AGCTGTGCGTCTGCTGGATCAG 1.32581714689

AGCTGTGGAAACGCAGGTTCAC 0.949760001287

AGCTGTGTATATGATGACACAA 1.46408724293

AGCTGTTAATGTCGTGATGTTT 0.913885110497

AGCTGTTGATTGGATAGATGTA 0.470318050545

AGCTGTTTCACTGGTAGAGAAG 0.566823510098

AGCTGTTTCTCTCATAGACAAG 0.187787559412

AGCTTACGACTTGCTACATTAG 0.436891932018

AGCTTACTAAATCTCTAAGCAC 0.641128877138

AGCTTAGCGTGACATTGCCCTG 1.57355804191

AGCTTATAAGATGGATCCTGAG 0.350871283074

AGCTTATTTTACTTTTAGCAAG 0.557198540181

AGCTTCAGGAGTGCATGTGCAG 0.89487862982

AGCTTCTGCGCTCACCTATAAG 0.628710726205

AGCTTGTACCATTCAACTTGAC 0.841037989188

AGCTTTCAATCTGATCCCAAAT 0.545733599749

AGCTTTCTAGGTGTTCAGAATC 1.54604616873

AGCTTTGTGCCTTCTTCTGCAA 0.808454704931

AGCTTTGTTTCTGCTTACTCAT 0.327878682328

AGGAAACGGCGACCTGATTCTT 0.437820738455

AGGAACCATTCCGACTTCAAAG 0.533482883907

AGGAATCTTACTCATTCTAATG 0.655640397456

AGGAATGGTCCTGAGATGTAAA 0.798397848182

AGGACACCCTCTCCTTAAACAC 0.578561642404

AGGACACTCCGTGCTGGAAAAT 0.764499396793

AGGACACTTGCTGCATAACTAC 0.535394546578

AGGACAGAAAGTCATTTCATAG 0.411425612605

AGGACAGAAGATCAGATCACTT 0.68145675038

AGGACAGTACCTGATGTAGAAC 0.243264164579

AGGACATCATGTGACACGTCAC 0.51206691734

AGGACCATGTGTCCAACACAAA 0.554870515277

AGGACCTAGGCCCGTGTGAAAG 1.31614345832

AGGACCTCATGGGCTTAAGTAA 0.821935380897

AGGACCTCATTGCGTCTTGTAG 0.903442304175

AGGACGCGTTCTCTTCACAAAG 0.525454767728

AGGACGGCTTATGAGAACTATT 0.882620855684

AGGACGTAACCTGTAAGCTAAC 0.604591877261

AGGACTAGCGGTGGTTGGGAAA 0.896296286986

AGGACTCCATGTGTTTCGAAAA 0.742055412786

AGGACTCGATCTGAACTGCCAG 1.41245690575

AGGACTCTTGCGCTATTCGGAT 1.75581187929

AGGACTGAATATCGATACGGAC 0.393419049731

AGGACTGCCCGTCATCGAGAAT 1.08487999595

AGGACTGGAAGTGCAAGCGATA 0.995738347443

AGGACTGGATGTGGTGAATATT 0.629194830344

AGGACTGTCTATTCTTAGTAAG 0.347020235602

AGGACTTTACACGATAAACTAA 0.556383893173

AGGACTTTATGTGCTTATACAG 0.450688187628

AGGAGACGTGGCATATCGTAAG 0.404185507691

AGGAGACTACGACCTAATCAAC 0.646168214689

AGGAGACTATGTCATCTAAGTA 0.689878024871

AGGAGAGCACTTGACGGGGAAG 1.06036387743

AGGAGAGCATCCCTTGTGGATC 0.311540277745

AGGAGAGTTCAGTGTAACAGAT 0.451738419464

AGGAGATCCTCTCATGTTACAC 0.864815413943

AGGAGATTATCTGAATCTAATC 0.540492321653

AGGAGATTGACTGATCATAAAT 0.374568111266

AGGAGCCAATCCGAAGATTAAA 0.459635214243

AGGAGCCTATCGCTAGAGAGAA 0.583683051085

AGGAGCTAGGCTGTATGATTAA 0.552194728165

AGGAGCTATAATTATGGACAAG 0.270546611806

AGGAGGGGACCTCTCAGGGGTC 1.36702795341

AGGAGGTTCCGTACTGGCAAAT 1.24676591798

AGGAGTGAAAGGCTTTGTCGAT 1.30031575779

AGGAGTGAATATCGTGCTTAAA 0.734000302444

AGGAGTGCCCGTGAAACCTGAA 0.911929809386

AGGAGTGCGCTTCCAGTTTAAG 1.6632920353

AGGAGTGTATTGCGCTGATGAA 1.50840170743

AGGAGTTAGGCGCCTTTATAAT 0.205662295735

AGGAGTTCATCTTCAGCTCTAC 0.715100916174

AGGAGTTCATGTACATCCGTTT 0.887982475291

AGGAGTTTATCTTCTGCCTTAA 0.526159678148

AGGAGTTTATGCTATCGAAAAG 0.353588032158

AGGAGTTTATGTTATGTGTAAC 0.850414901288

AGGATACGGACCTCAGCCATTG 1.04844243639

AGGATAGGGGGTCAAGTGGGAC 1.5836859168

AGGATATACTGTCAACTGAAAG 1.03081746275

AGGATATCTTCTCCTCGCTGAT 0.636535272788

AGGATCGCGTCGTGTTATTTAC 1.21823629451

AGGATTGAGAGTCAAGGTGTAT 0.02533158493

AGGATTGCGTCTCGCAGTGAAA 0.598296353065

AGGATTTTAGGACCTGACGAAC 0.37225107727

AGGCAACGATGTGTGGCTGAAG 0.357426182968

AGGCAACTCTCTGATGTAAGAC 0.630303673931

AGGCAAGAATAAGACGGAGTAC 0.822775880705

AGGCAAGGCGGTGTTACTTTAC 1.16057901021

AGGCAATGCTCGCTATTACAAA 0.562544217739

AGGCACAGAACTCGGTGTCCAC 0.483534631663

AGGCATCCTCCTGGAAGAGGAA 0.467562521312

AGGCCACGATCTGAAATGGTAC 0.530960258748

AGGCCACGCGTTGCAAGAAGAG 0.383423397466

AGGCCACGGTTTTGTAGGATAG 0.306058167057

AGGCCACTGGTTGATTATAAAA 0.260760278353

AGGCCAGAATTTTCATTTTAAA 0.460618967431

AGGCCAGGATCTCAAGGGAAAT 1.27226317284

AGGCCAGTGTGTGTTAGAAAGA 0.849457764885

AGGCCATAAAGGCAAAGTTAAT 0.467264777617

AGGCCATAACGTGATGAATCAC 0.481656851461

AGGCCCCGTCTTGCGAGGTAAG 1.92148931855

AGGCCGCGATGTGGACCTAATA 0.631613590827

AGGCCGGGGTTTGTTGGAAGAG 3.10006037712

AGGCCTCCAGGTCAAGGTTTAC 1.68237490136

AGGCCTCTTTGTGGATGGAAAG 0.525813336784

AGGCCTGAATTTGATCAACTAT 0.631714137181

AGGCCTGACGCTCAATCGACAG 1.24492980606

AGGCCTGTAAGCCAGTAAGAAA 0.448255837326

AGGCCTGTCAGTCTTGAGTGAG 0.378111140217

AGGCCTTACCCTGCGAGATGAA 0.939428290279

AGGCCTTAGGGAGCTAGTGGAG 1.77924073985

AGGCCTTATTCTCAATTGAAAA 0.3035345194

AGGCGAAAACTTCAAAAGCAAT 0.427789205682

AGGCGACAATGTTATTTTTAAG 0.854425797769

AGGCGACAGTGTTAACCTAAAG 1.08576806437

AGGCGACCATCTGTTCCAGTAC 1.19272819401

AGGCGACCCGATCCATATATAA 0.421687432019

AGGCGACCCTCTGCTGGTAGAT 0.680435175253

AGGCGAGAATCTCATCCCGATC 0.822131094977

AGGCGATAATATGATCTTTCAT 0.331265082009

AGGCGATGTTCTCGTGGAAGAA 0.706991021397

AGGCGGCCATATGCCTTTCAAA 1.57361668145

AGGCGGGGTCCTCAGGCAGGAG 0.774962656254

AGGCGTAAGTCCCATTGGGTTG 2.19752775086

AGGCGTGCGTCTGACTGAAGAA 1.58351859741

AGGCGTTGGTGGTATTCTAATC 2.70738190117

AGGCTACTTCCTGATAGCAAAT 0.867956051565

AGGCTTCAAAATGAATTGTAAC 0.838898975889

AGGGAATAGCATGCAGATGAAG 1.74520682826

AGGGAATCACGTCCTGCGCGAC 1.03000576699

AGGGAGCACCCTGTTGCACAAT 0.527720125046

AGGGAGTACACTTAAGCCTCAT 1.00666070965

AGGGCACAGTCACACTCCTCAT 0.784754916842

AGGGCAGGAGCCGGGCCCTATG 1.11492210334

AGGGCATACTGCCGTACAAAAA 0.365675905407

AGGGCTGAACATGATAGACAAC 0.589959857119

AGGGCTGAATGTTATTCACCAA 0.76322924803

AGGGCTTTTGATCCCGATCAAT 0.638527125184

AGGGGACCCAATTTGGTGAAAT 1.24270451542

AGGGGAGAACCTGCGTATCTAG 0.748015403195

AGGGGAGCTGCTTGCGACCGAT 1.48966202155

AGGGGCCATGTTCAAACGTGAA 0.255351059997

AGGGGCCCAAGTCATTCTCCAT 0.733321410077

AGGGGGGGAAGTGTATATGAAA 0.952074167758

AGGGGTAACTTTCAAATGTCTA 0.682290396131

AGGGGTCTGTTTCAACTACCAA 0.51351688266

AGGGGTGATTGTGAACCGGCAG 0.640277413715

AGGGGTGTGTCTCCTTGTCGAA 0.753374347517

AGGGGTTAGTCTTAAGGTTCAC 0.68175207182

AGGGGTTATGCTGACCGGAAAC 0.93011967883

AGGGTACTTACTCATTTCTTAG 0.661180160465

AGGGTAGAATTCTAGGGATCTA 1.01393073335

AGGGTATTGCATGCAATCAAAC 0.295749652665

AGGGTTTAAACTGATAGTTAAT 0.509056183528

AGGTAAAGAACTCGGTTCGAAA 0.479035785167

AGGTAACACTCTGAGTGTCTAA 0.40020546703

AGGTAATCTGTTGATATGGAAT 0.343863954518

AGGTACTTAGCTCCCTCAATAA 1.27756011565

AGGTAGAGAATTCATAGCGCAG 4.98466597527

AGGTATGATTGTGACTTTCGAG 0.9520953574

AGGTATGTACTGGATCATAGAA 0.451453753843

AGGTATGTATCTGGAACCGCAT 1.09706162384

AGGTATTGCTTTGCCTCTCGAG 0.611587665933

AGGTCACAACTTGCAGTTTCTA 0.431562578569

AGGTCACAATATCATCGAGAAC 0.366866116944

AGGTCACATAGTCGACTAGAAG 1.05029801948

AGGTCACCAGTTGATTAACAAG 0.244465596067

AGGTCACTCTGTGCTAATTGTA 0.595864289057

AGGTCACTTACTCTAGGGGCAG 0.161579783366

AGGTCAGGGAGTCCTACCTCTT 0.902261086831

AGGTCAGTAAACCCTGAGTAAG 0.0752548861274

AGGTCAGTATATCAACAAAAAA 0.230216094161

AGGTCAGTGGCTCGTCCCTAAA 1.48050017779

AGGTCATAAAATCTACTGGATG 0.173556531176

AGGTCATACTGTGATCAGGAAC 0.913203878263

AGGTCATTGTATGCGTAGTGAT 0.605687727287

AGGTCCGTGGTTGATTGTCTAT 0.784698070063

AGGTCCGTGTTTGCCAGCTGTC 0.315905489365

AGGTCGCTCCCTGGTCTGTTAG 2.07882367424

AGGTCGGCCTCTGATGACCAAG 0.839830607792

AGGTCGGGTGCTGTAGGGTTTA 0.969608615257

AGGTCGGTCGGTGCCAAGACAA 1.85308436635

AGGTCGTAACGTCCCCGGCAAG 0.678364339872

AGGTCGTACATTGACAGTTTTA 0.280867578801

AGGTCTCTAGACCATGGCTAAA 0.507724650746

AGGTCTCTGCGCCATACAAGAC 0.846454871502

AGGTCTGCATCTGATCATGAAT 0.649386233107

AGGTCTGCTCCTGCTGAGATTG 1.0839465183

AGGTCTTAAGCTCCTCCCTATG 1.02966221773

AGGTCTTAAGTTCAATTTCAAA 0.32925529847

AGGTCTTAGTCTGCCATCAATC 0.472144000715

AGGTCTTTCTCCGGTCAACAAT 1.01607484896

AGGTGACAGCGTTTTTCATAAA 0.773240286806

AGGTGACGAGCTGATACAGTAA 0.953794608613

AGGTGAGACCCACCAAGGGATT 1.44353326432

AGGTGAGGAGGCGCACCTATAA 0.579802324475

AGGTGAGGCTGTCAAGCACAAC 1.08932308257

AGGTGAGGGTATTAGTCGACAT 0.300184824745

AGGTGAGTTGCTGTTTCAAATA 0.711379218888

AGGTGATAATCTGGAACTAAAT 0.100132848006

AGGTGATGAGCAGACGAGTTAG 0.833135303952

AGGTGCCAATCTGGTTAACCAA 0.285706288829

AGGTGCGAGTCTCCGTAATGAT 0.503984848085

AGGTGCGGGGATCGTAAATTTC 1.1388389785

AGGTGCTACTACCACCTCAATC 0.627775250935

AGGTGCTGAACTCGATCACATA 0.634754570513

AGGTGCTTGGCTGAAAACTAAG 0.775981676491

AGGTGTAGCTCTCTAGCCCAAA 0.672252687481

AGGTGTAGGAATCGTACTGAAC 0.215743586137

AGGTGTCAATCTCGTTCGGAAA 0.703715914286

AGGTGTCGTTTTCAAGGCTGAT 0.836096130754

AGGTGTCTATGTCCATATTAAG 0.473616090197

AGGTGTGACCTTTGAAAGGAAT 0.499718617257

AGGTGTGACGTCTAAGAAACAC 0.450902595081

AGGTGTGTAAGTGCTTACAGAC 0.763676669966

AGGTGTGTCTGTTACCAAAAAC 0.487198587756

AGGTGTTAGGCTGCTCGTACTT 0.666617247597

AGGTGTTGCTAAGATACTGCAA 0.604213209515

AGGTGTTGCTGTGAGTGTATAT 0.303033465766

AGGTTACATACGGTTTCCATAG 0.570367288557

AGGTTAGACTGTGTACTTAGAT 0.889911721811

AGGTTAGCTTATTTTACCCTAC 0.80523317672

AGGTTATACGCTGAGGGTCAAA 0.650173483921

AGGTTGCACGCTCATCTCAGAA 0.869562194933

AGGTTTCAACATGTTGGTGTAA 0.622508804247

AGGTTTCCTTATGACTCTACAT 0.959822732813

AGGTTTGGATGTGTTTTCTATA 0.452353430212

AGTAAAACATGTGCTATGTCAA 0.621883607463

AGTAAACGAGGGCCTTTTAAAT 0.564185336816

AGTAAAGAAGGTCATACATAAT 0.735063150343

AGTAAGTAATGTCCTAATAGAA 0.668499466685

AGTAAGTACCTAGCTTACCCAT 0.428404188422

AGTAAGTAGTTGGCTACTGAAG 0.625373228987

AGTAATGCGCCGCTCAATAAAA 0.524280517106

AGTACACAATCTCCTGACCAAC 0.356844744607

AGTACACAGTCTGTTGGTCTAT 1.01451983559

AGTACAGCATGTCATTTTAAAC 0.639147966439

AGTACAGCTAATGATTAGCAAC 0.694884272449

AGTACAGCTACTGCTAGTGAAC 0.834385782945

AGTACAGGATCTCCTTGCTAAG 1.02991834583

AGTACAGTGCGTCATATTAAAT 0.164066321944

AGTACCCACGGTGAGTGAGGAA 0.619689282587

AGTACCGAAGGTTATGCATAAG 0.687925078405

AGTACCGTATGTGTAAGAGGAT 0.406299956348

AGTACCGTCTCTCCTAACGTAG 0.282171891714

AGTACCTTATGTCGTGTTATAA 0.424184993576

AGTACCTTATGTTATTCCATTG 0.112299956809

AGTACCTTGGGTCAAGCGCAAA 0.914113761797

AGTACGACAGCTCCGCGGCATA 0.247400423072

AGTACGCAATGTTCATCCTGAA 0.50414581798

AGTACGGTACATGATTACCAAT 0.523856684109

AGTACGTTAGCTCACGTTAAAC 0.271351179063

AGTACTCAAGCTGACAAAACAG 0.577776793063

AGTACTCTCTCTGTTGAGGCAA 0.502663320619

AGTACTCTTTCTGATAATTTAA 0.401114838062

AGTACTGAATCTGAAAAAAATT 0.326997888964

AGTACTGCTACTCCACTATAAT 0.228558371978

AGTACTGGCTATTACGAAGCAG 0.686020358576

AGTACTGTATGTGCTTATGAAT 0.606957464359

AGTACTGTCTGACCCATATTAG 0.285485796341

AGTACTTAAGGTCCTCATCGTA 0.492714768877

AGTACTTATAGTCAAAAAGTAC 0.578908381716

AGTACTTGTAAAGGAATCCCAT 0.566138644675

AGTAGACGCTGTGCCTCTTTAC 0.604477793095

AGTAGAGATCGTGAGTACAAAG 0.432756021021

AGTAGATAATCTGTTCTTCCTA 0.268945543234

AGTAGATACGCGGACAAACAAG 0.657702487644

AGTAGATCGAGTCTAAGGGAAC 0.358733106328

AGTAGATGCGGTCACAGATGAC 0.71021136337

AGTAGATGGACTCCAGGTATAT 0.482087631881

AGTAGATTCTCTGATTGTTAAA 0.655892617732

AGTAGATTGATACCTTAGATAG 1.16692103664

AGTAGCCCCTGTGATGAGCTAG 0.692915307132

AGTAGGAACTTTGCCAATTTAA 0.554871877082

AGTAGGGGTTGTTAATGCTAAG 1.07203385462

AGTAGTCAGTGTTTTGCTATTA 0.737756823635

AGTAGTCCACGTTATTTACAAG 0.474191162778

AGTAGTGAAGCTGATCTGAGTT 0.790651382738

AGTAGTGGCACTGAATAGTAAT 0.907874422798

AGTATACAATCTCTATACAGAT 0.137054686931

AGTATATTGTGTCTACAAATAG 0.30306804761

AGTATGTACTATCTTCATGAAT 0.583722166854

AGTATTCATGTAGCTCATAAAG 0.677231961171

AGTCAACTGACTGTTGAATAAC 0.35526673911

AGTCATCGTAGTGATGTTGGAC 0.520551353138

AGTCATCTTTGCGAAAACCGAA 0.531695243686

AGTCCACACACTTACCTCTGTT 0.507336841181

AGTCCACTCGATTTCAAGTAAG 0.81269877241

AGTCCACTCGGTGATAGAGAAA 0.520483186835

AGTCCAGAACTTCCTTAAGTAA 0.455118873918

AGTCCAGTAACTGGTAGAATAC 0.477968086587

AGTCCAGTCGCTCCTAGCTAAA 0.678702091073

AGTCCATTAGGTTCCGTTTCAC 1.10267208977

AGTCCATTCTCATTTCTCCAAT 0.675369723555

AGTCCCTTAATTTGTCAGGTTC 0.547525740527

AGTCCGCTATCCGACCCCGATT 1.00597429249

AGTCCTAAATGTGAAGCCGGAC 1.14026583105

AGTCCTACACGGGACAGATTAG 0.770398230064

AGTCCTCTGTATGGAGCCAAAT 1.12457980845

AGTCCTGAGTGTCTTTTCTATA 0.30181442836

AGTCGACAGTATGAAGTCAAAC 0.248396437828

AGTCGAGGAGGTCTTAAAACAA 0.647660850853

AGTCGCGAATCTCCTCGGTTAT 0.784758559232

AGTCGCTAATCTCCTGAACAAT 0.284508213867

AGTCGGGAATCTCCGGTAGTAT 0.547119108755

AGTCGGTATACCCCTTGATTAA 0.596329276531

AGTCGGTCGGATGCTTGTCGAC 0.956085070775

AGTCGTGACTGTGCTTGCAATA 0.742419648092

AGTCGTGGGCTTGATGAATCAC 1.50156963298

AGTCGTTAACGCGTATCGGAAC 0.786657063757

AGTCGTTAATGTCGATTCTTAC 0.645326659765

AGTCGTTTGTGTCCTAGCTTTT 0.579567295805

AGTCTGGCCGCTGCTCAAAGAC 1.52162596836

AGTGACGTCCGTCCGCGCTTAA 1.61951841093

AGTGAGTCGTGTTTTTTGTGTG 1.50876990396

AGTGCAAACTGTCGTCCAGCAG 1.47663785091

AGTGCAAACTGTCGTGGCGTAG 2.50533350097

AGTGCACCAATTGTTAAAAAAG 0.319067259169

AGTGCCCGGTGTGCTTATTTAA 0.296637529417

AGTGCCTAGTGTGCTTCGTTAG 1.69784359409

AGTGCTGGATGTGAACGCGAAG 1.51459261433

AGTGGAACCGTTGATTGCATTA 0.532145836469

AGTGGACAATTTCCTCGCTTAG 0.840003105587

AGTGGACAGGGTCGCGCTGTAA 1.1466660917

AGTGGACGCCCTGCTGAAGTAA 1.22011580726

AGTGGAGAACGCGATACCCTTT 0.706668261862

AGTGGAGGCTCTCTCTAGATAG 0.573753254981

AGTGGATAATCGGCACAATATG 1.29811706869

AGTGGATGAAGTGCAAGGCTAG 0.651223589561

AGTGGGAAATCTGCGTTTGAAG 0.733153420088

AGTGGTCACAGTGAATTTAAAG 0.552327443582

AGTGGTGGATTTGCTGTATAAT 0.581322135791

AGTGTATAATACCACAAGTCAG 0.806774442202

AGTGTATACCTTCCTGATGCAG 0.834370545927

AGTGTTCTTATTGCCAAGACTC 0.688145271806

AGTTAATTCGGTCATTACGATC 0.477894004352

AGTTAGCCCTGTCCCTAGTAAC 1.2630299884

AGTTATCTAACTGAAAATATAG 0.325551812784

AGTTCAATCTCTGGAAGCGTAG 0.426913167624

AGTTCACAATGTGGCCCGTCAA 1.31755388666

AGTTCACATTGTTCTCGTCATA 0.361186296231

AGTTCAGGTTCTCGTCTAATAA 0.221655017232

AGTTCAGTGTCCGCCTCGACAC 1.0378434546

AGTTCAGTTATTTCTGAGAGAC 0.316772409381

AGTTCATTTCGCCCGGCCTTAG 0.823463634926

AGTTCCGAAGGTCCTTACCCTG 0.563911064246

AGTTCGCTGTGCGCTGCAGAAC 1.08610036223

AGTTCGGCACCTCCTAAGACTC 1.13147780576

AGTTCGGGACCTCATTTAGCAG 1.56933848465

AGTTCTCCGTCTGCATTACTAG 1.0216991023

AGTTCTCGGAAAGTTATGCGAG 1.28482039001

AGTTCTGTATCTGATCGCAGAG 0.699258941717

AGTTCTGTTTGTCAAGCCGGAC 0.384139380937

AGTTCTTAAGGAGTATATTGTC 0.467041949588

AGTTCTTAGGGTGAGTAAGAAA 0.136321811032

AGTTGAATAGCAGCAATTCTAT 0.390190733981

AGTTGACCAGGTGGTCCAATTG 0.997854926758

AGTTGAGAATCTCTCCAGAAAA 0.347112553689

AGTTGAGAATGTCACCGTTCAA 0.551589054502

AGTTGAGCGGCTGATTCAGAAC 1.67312802019

AGTTGATAGGTTGCTACGAGAG 0.292901320551

AGTTGATAGTGTGGATTCGGAA 0.796185715144

AGTTGATGCTCTGAAGTCCATT 0.419965716531

AGTTGCGACTTTGAAAGCCCAT 0.71846747024

AGTTGGCATTCAGCGGCAAAAC 0.700124440759

AGTTGGGAAGTTCGGTAGTGAG 0.819523529555

AGTTGGTTCTCCCCACTGGGAG 0.549220478879

AGTTGTAGAACGTGCTTGACAC 1.11461043326

AGTTGTCAGTCATGCTTCTAAA 1.44287503858

AGTTGTCTAACTCGGAAAACAC 0.637315284493

AGTTGTGCACCGTTCGAGGTAG 0.893234802894

AGTTGTGCATGAGCAGCTCCAG 0.587227189771

AGTTGTGGACCTTATATCCTTC 0.541726760924

AGTTGTTAAGGTCATCGCATAG 0.720874457219

AGTTGTTTAAGTCGAGTCACAT 0.687491691406

AGTTGTTTGTGTCTGGATGTAC 0.22234573949

AGTTTATCAAGTGCACTAGTTA 0.45051892494

AGTTTTTAATTTCATTCCAAAC 0.333204781297

ATAAAACAAGGTGTTCGTCGAT 0.646720051166

ATAAAATCCGGTGGGATAAAAA 0.400011166443

ATAACTGCTTCGCTTATGTAAT 0.367184297065

ATAACTGTATCTGCAGAACATG 0.242822045497

ATAAGAGAAACTGAATAAGAAT 0.412370063011

ATAAGAGACGCGCGTAGTGCTG 0.740074575765

ATAAGGCACTCTGATTTATAAA 0.246668516625

ATAAGTGCCAACCATTCGTAAC 0.457192210952

ATAAGTTTTTATCTATGAGTAC 0.42771323975

ATAATCCCGTCTGACTGGAAAC 0.391054719366

ATAATCGTCTGTCGTTGTGTAG 0.722602638392

ATACATCCTTGTCCCAGCGTAA 0.572219365161

ATACATGAATCTGAAAAATGAG 0.531426547118

ATACCTGCCTCTGTTAAGGTAG 0.768235783713

ATACGTGAATGCGCACATTGAT 0.414986053469

ATACTTGGCTCTCTTTGATAAC 0.741514129724

ATAGCTGAGTGTCCTGTAGAAG 0.79638179779

ATAGCTGCATGTCTTGCTAGAG 0.505387685829

ATATAAGAATGACATAGGCAAT 0.339036666802

ATATACCATTGTCGATAAACAA 0.499074502482

ATATCAGTATATCCTAAATAAA 0.405547254608

ATATCATAGGGTTAGTAAAAAC 0.486371276766

ATATCCGTCTCTCCTGTACAAC 0.248058561686

ATATGATTCTTTTCCAGTAAAA 0.43384906939

ATCAAACCCGCCCCTGATTAAA 0.513597567303

ATCAAACTATCTGTCCATAGAC 0.710395455088

ATCAAACTGTCTCCGGGAACAG 0.214568879741

ATCAAAGAAGGCGAGCTGATAT 0.430370274367

ATCAAAGCATATCATCAGACAT 0.118566489136

ATCAAATCATGTGCTCACAATC 0.621281971674

ATCAAATTCAAGCCTAGGATAA 0.550047770878

ATCAACAATGCTCCAGGCAGTA 0.548681303286

ATCAACCTAAATCATGATCCAG 0.458658945264

ATCAACGAATCTGTAGTACTTG 0.364339997333

ATCAAGATATTTGCATAAACAC 0.52951712304

ATCAAGTAACATGCTTACAATC 0.251810694677

ATCAATCAATCGTTAGGGTAAG 0.24632300328

ATCAATCAGACAGCTCCCTATG 0.143678858539

ATCAATCTTGCCGATTTGTGAA 0.400285192112

ATCAATGCAGGTTCGGAGTTAT 0.254639355905

ATCAATGCATTCCAACCTAGTG 0.478453459715

ATCAATTAATATCTTGCGAGAG 0.473352446237

ATCAATTAATCTCAACGTTCAT 0.480052199702

ATCAATTAGTGTTGAAAGAAAC 0.363222725847

ATCACAAAGTCTGCTGATCCAC 0.388065127302

ATCACAATTTGTGTTAGACAAA 0.419786098116

ATCACACAGCCCGCTACAGAAC 1.0240771894

ATCACACAGGCGCCTAGGATTC 0.540642695512

ATCACACCCTTTGCGGAAAAAG 0.731234037276

ATCACACCTTAGGCTTCAATAC 0.271758018429

ATCACACGGGCTCCGGGGTCAA 0.691098660385

ATCACAGAAATTGATCGGTTAA 0.639106176119

ATCACAGAATCTGGAGTAAAAC 0.519297599055

ATCACAGACCAGCGAAGAAGTG 0.379314591793

ATCACAGACGCTCATCTTCAAG 0.262801570136

ATCACAGCCTCTGTACCCTCAG 0.635825764088

ATCACATACTATGTAACTGGAA 0.37289490147

ATCACATAGAATGTAAGTATAA 0.271837074997

ATCACATCAAGGGCTGTCGGAG 0.126483968633

ATCACATCGTTTGCTCCCAAAC 0.417108886307

ATCACATGAGGTGTTACGTATT 0.559382084487

ATCACCCCAACTGGGGGAAATG 1.05241369856

ATCACCCTCGTACCTCAATGAA 0.508511811645

ATCACCGGAACGGCTCATTAAT 0.293382119192

ATCACCGTGACTGAAAAAGGTG 0.819962675764

ATCACCTAATGTCGTTTGACAC 0.736807914197

ATCACCTACCGTGGATTCCATG 0.340215323592

ATCACCTTATTTGCACTAGATT 0.314012729997

ATCACCTTCTATCGTCATACAT 0.418258176293

ATCACCTTTGCACTTCAGGAAG 0.596650763711

ATCACGCACTCTGATGCTAAAA 0.631728236431

ATCACGGTATCTGTTCATATAA 0.91715833778

ATCACGTGCTCAGGTTTCTATG 0.446062363474

ATCACTCACTCTGACGAAAAAA 0.625107426426

ATCACTGAAGCTCATGCTTAAT 0.239266270855

ATCACTGACTCAGATAGATTAC 0.624441145758

ATCACTGAGGTTGTTGGCTCTG 1.42068717361

ATCACTGATTCTCCGTGAATAC 0.269874468192

ATCACTGCCACTGAAGGCGGAT 0.781598797369

ATCACTGGACGTCGATGGGTAG 0.887905381337

ATCACTGGGATTGGACATTTAA 0.431680479607

ATCACTGGTGCTGATGTTATAA 0.336372243598

ATCACTGTAGTTGCTTGGGGAG 0.356002416501

ATCACTGTGTGTCAAACTGAAT 0.480737360893

ATCACTGTTAGTGCATCCAGAT 0.471619577558

ATCACTTAGTATCGACTAACAG 0.664463499901

ATCACTTAGTGTCGTGGTTGAC 0.962613939588

ATCACTTGCTTAGAACTCAAAG 0.819231960998

ATCACTTTGCAGGCATCGTTAC 1.15357409762

ATCACTTTGTTTTCTCACTTAG 0.872754781742

ATCAGAAAGTCTGCTACGATAC 0.412196729378

ATCAGACAAAGTCCGTCAAAAA 0.316042791325

ATCAGACAAGATCCTAATATAA 0.314275749689

ATCAGACACACTGCTACGTTAT 0.357346660597

ATCAGACCAATTCCTGATAAAT 0.278793703521

ATCAGACGCCCTCGATCACAAC 1.21177676754

ATCAGAGACTCTCATAGAAATC 0.429314154783

ATCAGAGAGAATCGTAACTAAA 0.240911533531

ATCAGAGAGCTTCGTCTTTGAG 0.761813569586

ATCAGAGGGCCTGCTAGGAAAC 1.20989563181

ATCAGATAATGTCCATAACTTT 0.249608914278

ATCAGATAATGTCTCTGAAAAA 0.451339527619

ATCAGATAATGTGTGAGCTAAT 1.29686433246

ATCAGATACACTTATTGGAAAC 0.645831077754

ATCAGATATTTTCAAGTTATAC 0.250856680262

ATCAGATCATCTCTATTCTCAC 0.448281854568

ATCAGATCGTCTCAATGGGCTG 1.60788699442

ATCAGATGATCTGCTGGCTATC 0.890509845162

ATCAGATTATGCGATTACGAAG 0.508343857912

ATCAGATTCGGTGATTTGTTAG 0.424176378158

ATCAGCAGCCTTGCATACGAAG 1.18870511234

ATCAGCCTCAGGGTATTTTTTA 0.579347327014

ATCAGCTACCCGTGTAAGTAAA 0.747881157605

ATCAGCTTTGGTCCTCGGAGAT 0.430072988621

ATCAGGCAACCTGCTCAGTGAG 0.374051204831

ATCAGGCAATCTGATCCCCTAG 0.383987374127

ATCAGGGCGAACCATTATGAAT 0.468305248619

ATCAGGTACTCACATAATACAT 0.531997065368

ATCAGGTGAAGTCCTACTTATA 0.750795907662

ATCAGTATGTATGAAGTCAAAG 0.469204354175

ATCAGTCAGTATCCTTGTATAT 0.192988183347

ATCAGTCAGTGCGATAGGGATA 0.769900418199

ATCAGTCCTTGTGCGGTCGGAA 0.988584909082

ATCAGTCTACATCCCCGCTAAT 0.643907384788

ATCAGTCTCATTCGCATATGTG 0.723559163574

ATCAGTCTCCGTAGTAGCTCAA 1.124476782

ATCAGTGAATCTGTCGTTGCAC 0.443882416799

ATCAGTGAATGTCCATCCGAAA 0.617401248024

ATCAGTGAGGGTCCAAGTAAAT 0.706265900015

ATCAGTGGAATTCAATTCGAAA 0.89944455091

ATCAGTGGCACTGCAGATACAT 0.964347588862

ATCAGTGGCCTGCATGGTCTAC 2.68537637775

ATCAGTGTCACTGTAGGAGAAC 0.481539574867

ATCAGTGTCTCTGCTGGACTTT 0.480747301392

ATCAGTGTGTCGGTTGGGTAAG 0.555566577163

ATCAGTTAATATGAACAAGAAG 0.438023408556

ATCAGTTACGGGGAACGTGTAA 0.761582283396

ATCAGTTATTCACCACCTGAAC 1.64346614464

ATCAGTTTCTGTGCATATTAAT 0.760604661011

ATCATAGTCGGTCATAGACGAT 0.485370688749

ATCATATATTCTCCTCGGTTAG 0.367903678708

ATCATATGATCTCATAAACATA 0.561210213562

ATCATCCTCGATGACAAACGAC 0.799788517673

ATCATCGACCGTCTAGGCGCAG 1.48584595346

ATCATCTCGGTTGTGAAGCCTC 0.981105053722

ATCATGGCAGGTCGTAATTAAG 2.998330805

ATCATTCAATGTCTTGAGCATG 0.37843151853

ATCATTGAACATGCTTCGTAAC 0.54718596233

ATCATTGAATGTCACTACTGAA 0.726837320037

ATCCAAAGAAGTGTAAGCGAAA 0.528282234392

ATCCAACTGACTGTTTCTAAAG 0.754929444168

ATCCAAGGCGATGAAGCCTGAT 0.586819806005

ATCCAATTAGCTCAAACTGTAT 0.315318343093

ATCCAATTATGAGTTGAGAATC 0.513655591519

ATCCACCGTGTTGTGTGTGAAG 0.9733457982

ATCCATTACCATCATGATAAAT 0.361221121279

ATCCATTACTCTGACTGGAGAT 0.653958950091

ATCCATTAGTGACAAAGATGAG 0.216398434163

ATCCATTCATTACATTCAGATT 0.494409979163

ATCCCAATATGTCTACGTAAAC 0.810778275636

ATCCCACAAAGTTGAAAGATAC 0.18868543041

ATCCCACAGTCTCGCCACTGAA 0.849189103298

ATCCCACTAGGTGCATTAAGAT 0.709527537981

ATCCCACTCTATTCTATGACAA 0.550875935548

ATCCCAGGAACTGATAGATGAA 0.282538190738

ATCCCATAGTCTGCTGCAAAAC 0.437404094684

ATCCCCGAATCTCCTTGCACAC 1.24355513231

ATCCCCGCCATTCGTGGATCAA 1.02147090316

ATCCCCTACACATGAGACGGTC 0.521247267098

ATCCCGCGGCCCGAAGTGAAAT 0.8642865928

ATCCCGGAGAATCCAATATGAT 0.267474419426

ATCCCGGTAGATGTTCCTTTTA 0.638110061209

ATCCCGTCAGCTACTTGGATAT 1.64788547723

ATCCCGTCATATGAACACAGAT 0.627502160161

ATCCCGTGAGGTGAAAGAGGAG 0.344070396606

ATCCCTCAAGGTCAAGCGTAAA 0.170916861866

ATCCCTGGGTGTCTTGCACATA 0.608111528229

ATCCCTTACTCGCGTAGAAGAC 0.744440445651

ATCCCTTGATCCCATCATCCTG 0.692585874364

ATCCGAGACGCACGATTGGGAG 0.582705802205

ATCCGAGGGAGTGAAGAAATTA 0.658958499606

ATCCGAGTAAATGAGATGTGAG 0.318800704409

ATCCGATAGTCTGCGACAGAAG 1.27925644683

ATCCGATCTTACGCAGGAAAAC 0.521155267048

ATCCGATGGCATGGTATAATAG 1.12260781107

ATCCGATTCTCCCATGCTTAAA 0.751575110374

ATCCGCAAGACTCACTTTCTAG 0.443461255448

ATCCGCCAGTCTGAAAAGTAAC 0.361806596986

ATCCGCCCCGGTGGTATAGTAC 1.66698811925

ATCCGCCGCTCTCAATCCATTC 0.486647397688

ATCCGCGAATCTCCTGGGTCAA 0.412008740927

ATCCGCGCGAGTGGTTTGGTAG 0.730082841965

ATCCGCTAAAGCTCACTAGAAG 1.07896880555

ATCCGGCTATTTGATGCTACAA 0.216089391455

ATCCGGTTCACTCGCATGTTTT 0.81711090292

ATCCGTAAGTCTATTCCTTCAG 0.219320897559

ATCCGTCGAACTCGCGCCTCTC 1.29309700741

ATCCGTCGAGCGGCCTTAACAG 0.526628727745

ATCCGTCGAGCTGCCAAGTAAA 1.18975892912

ATCCGTGAATTTCTTGGATCAA 0.249785837158

ATCCGTGAGGTTGCGTCGTAAA 1.00289428012

ATCCGTGGATCTCAAGGCTGAT 1.68931776323

ATCCGTGTATCGCTGAAGGTAT 0.765591392988

ATCCGTGTATCTGGCATGAGAA 0.230418688659

ATCCGTGTGGGTGATCACGAAC 0.851631707042

ATCCGTTGATGTGTTAATCGAA 0.958831712798

ATCCGTTGCTCCTCTTGTTGTA 0.291947241168

ATCCTACTTTATTATGCGGCAC 0.460145438745

ATCCTCCCTTTTGCTTATTCAT 0.203366008606

ATCCTCCTGCCGCCATATTATA 0.568324948553

ATCCTCGCCTGTGAGACAAAAG 1.03258416101

ATCCTCTGGTGTCCCCGGACAG 0.789581242157

ATCCTTTAGTCTGAAACGGTAG 0.470458122722

ATCCTTTTACATCTGGATTAAG 0.284649900623

ATCCTTTTTCCTGGAAATATAT 0.413361564082

ATCGAGTACTGTCTATATCTAT 0.460569900849

ATCGATGGACGTGATGTAGGTC 0.959944515857

ATCGATTCCTTTCGTGCTATAC 0.480341647946

ATCGCAATAAGTGATTCCATTG 0.649663188745

ATCGCACAACGACTATGAAGAA 0.300707491855

ATCGCAGAATTTGAGCCTTAAG 1.44535840438

ATCGCAGCATATCCTTACGAAG 0.323470679166

ATCGCATAGCCTGATGTACTAC 0.510673197781

ATCGCCCCAGCTGAGTCCCCAT 2.80968937217

ATCGCCGAGTGTCCATATATAT 0.457873374334

ATCGCCGTCTCTGAAAAAGGTT 0.399401312689

ATCGCTCTAGCTCCTGAGCTAG 0.808588175305

ATCGCTGTGCCTCCTCGGATAG 1.25718803897

ATCGCTTAATCTCCTGGAATTG 0.519634676927

ATCGCTTTATAAGAAGACGAAC 0.616621583321

ATCGGACACCCTCATATCGAAA 0.655376456965

ATCGGACCAAAGGGTTCGGCAA 1.52459416297

ATCGGCGTGTCGCATCTGCGAC 0.059915387884

ATCGGCTGATGTCCTGTAGGAG 0.678530410858

ATCGGGGGATCTGGATAGCAAA 0.937874897944

ATCGGGTTCGGTCCACAGAAAT 0.615794930453

ATCGGTGATTCTCATGGGGGAG 0.73727975984

ATCGGTGTAAGTCCAAACACAC 0.351082484619

ATCGTACTGTTTGCTCGCAGAA 0.66588055855

ATCGTGCGGGGAGTACATGCTT 1.00219396912

ATCGTGTAATTTGCTATATCAG 1.10788316994

ATCGTGTTCTGCCCAACAGTAC 0.166755782658

ATCGTTCATGATGGTACCTTAG 0.78001419834

ATCGTTGCAACTTTTAGGGTAA 0.867233940091

ATCGTTTAGTGTGATGAACAAA 0.272638659725

ATCTAAGCTGCTCCGATTCTAT 0.277008792647

ATCTAAGTATCTGAAAAGAATT 0.224129481933

ATCTAATAGACTGCTTAAAATG 0.232988309318

ATCTAATAGTGTGACATTAGTA 0.542405915029

ATCTAATCAAGTGATCGATGTC 0.621413752089

ATCTACTAAGCTGATCCCATTA 0.614580726784

ATCTACTAATGTCATGATTAAG 0.19710498655

ATCTACTAATTTACTTTCGAAG 0.66434701864

ATCTAGGAAGGGGATGGAACAA 0.859781866758

ATCTATCACACTTCAATACATA 0.315010047334

ATCTATCCATTTCGACCGTGTA 0.504672972438

ATCTATCCCACTCCTACAATAA 0.128967702614

ATCTATCGAGGCCCTCAACAAG 0.969254028564

ATCTATCTACCTGGAAGGACAA 0.596371304332

ATCTATGCATGTCATTCTCAAA 0.439412167356

ATCTATGTGACTGAAATGTCAG 0.475087322102

ATCTCACGGCTGGTTGAAGAAT 0.587527953816

ATCTCACTGTCTGCTTTGAAAT 0.316422962949

ATCTCAGACACTGTTGGATGAT 0.510745409253

ATCTCAGACCGTCGACCATTAC 0.474610718742

ATCTCAGCGGACCGTCATCTAG 0.444207445286

ATCTCAGTAACTGAAGAATTAA 0.273433669682

ATCTCAGTGAGTCATACTTAAG 0.545821872069

ATCTCATAAGCACGCCCGTAAA 0.457283715168

ATCTCATAATGTGGCATAATAC 0.633841447858

ATCTCATAGTATGCCGAAAGAA 0.749079333998

ATCTCATGAACTCCATGATGTA 0.803175802629

ATCTCATTATCTCACAAACTTC 0.885959383456

ATCTCATTTTATGTTGACTAAC 0.42969536469

ATCTCCGAATGTCATGCGTTAG 0.893535905999

ATCTCCGTCTTCGCGACTGATG 1.68807497553

ATCTCCTGAACGCAAGGGGCAG 0.552008073956

ATCTCGCAACGTGCTAGCTGTC 1.13915205534

ATCTCGCATTGTTCTAACTTAC 0.71247127043

ATCTCGGACGATGTTGGAAGTC 0.885924450681

ATCTCGGGATGTGAGGAACTAC 0.977571505491

ATCTCGTACTGTCCAGCCGAAT 0.332974358285

ATCTCTCATTCTGTTGTTTATT 0.371587730314

ATCTCTCCCTCGGAGTAATTAA 0.816235756402

ATCTCTCTAACTGTTTACTGAG 0.687699793438

ATCTCTCTAGTTCGTAGACTAG 0.623029843099

ATCTCTGAACCTTACTGAGGAA 0.472550137054

ATCTCTGAGTGTCAACTCCCAA 0.626431429976

ATCTCTGATTGTGTTTCGGAAA 0.486015005791

ATCTCTGTTACTTCATAGAAAT 0.333700510351

ATCTCTTAACGACCTTCGAAAC 0.421673000859

ATCTCTTACGGTTTTTCTGTAG 0.507218522919

ATCTCTTCAGTACCAAATTGAC 0.138668461232

ATCTCTTTATTCCCTTGGCCAT 0.412998591769

ATCTGACAGTCTCGACGAAAAC 0.56141201459

ATCTGACTAGCTGCCACGGAAC 0.393352483759

ATCTGACTAGGTCAGGATATAA 0.671576678927

ATCTGACTGTATCATATCGTAA 0.303468532184

ATCTGAGAATCTCACATCTATC 0.654779488539

ATCTGAGAGTGTGAACCATTTG 0.531184953759

ATCTGAGCATCTTCTAGACGAA 0.531897728418

ATCTGAGGAGGCGCTCTGGGAT 1.12249944816

ATCTGAGGCTCTCCATTAGGAT 0.466154104774

ATCTGAGTAGGTGTATAAAAAC 0.562639419921

ATCTGAGTATGTCATGAGTCAA 0.618025278822

ATCTGATAATATGAATGAGATC 0.609959116336

ATCTGATCACTTGGTTTTAGAG 0.611802271102

ATCTGCCAGTGTCTAGGTATAG 1.54139446075

ATCTGCCTGCATGTATGTATAA 0.531527819425

ATCTGCGAAAGTGAAAAGGATT 0.578378506941

ATCTGCGCGTGTGATTCGTCAT 0.310327731573

ATCTGCGGTGCTCTGAGCCCAA 2.87518492558

ATCTGCTAGTATTATGTCATAA 0.653720387701

ATCTGCTTCGGTGCTCTTAGTG 0.967557765135

ATCTGCTTGGTTCAGCAAGCTC 0.825787675013

ATCTGGGAATGTCTTTATAAAC 0.330614255684

ATCTGGGACTGTGAATACCATA 0.949821213041

ATCTGGGATCGCCCTAAGCCAA 0.19432644529

ATCTGTCAACCTCCAAGGTAAG 1.37103481967

ATCTGTCAATTTGGACTCGGAT 0.617990084397

ATCTGTCACGCTCCTTGTCGAT 0.260702347778

ATCTGTGAATGAGATTAATAAT 0.182043020744

ATCTGTGACGCTGCTGCTAAAA 0.555707544316

ATCTGTGCATGTGGAAAACATC 0.757256945533

ATCTGTGGATAGAATGAGACAG 0.826874605687

ATCTGTGTACTTTCTTACTCAG 0.469664871612

ATCTGTTCATTGGTGAAAGGAA 0.882722343539

ATCTGTTTATCAGAACTACCTG 0.550148624948

ATCTGTTTCGATGGTAAGCCAC 0.354144167774

ATCTGTTTGCGTGATAAAAGAG 0.579272350386

ATCTGTTTGCTTGCTACGATAG 0.378893066556

ATCTGTTTTGATCACAGACCTG 0.583000385371

ATCTTACGGGGTTACGGCCTAC 0.680784950567

ATCTTATCATCTCCTGGTGAAT 0.739258565927

ATCTTCCAATATCCTGATCGAT 0.640384208858

ATCTTTATATGTGCTGTACGAA 0.92604507886

ATCTTTCGAGGTGTTGTATAAC 0.32501589463

ATCTTTCGTAGACCTTGGTGAG 0.696044406646

ATCTTTGAAGGTGAACACCGAA 0.297523433204

ATCTTTGTGTCACACTCGATAA 1.05356758205

ATGAAAATGTCCGCATGCTCAA 0.164059937037

ATGAAAGACAATGAAGACGATA 0.284550603215

ATGAAAGCCTTTGCCTGTTTAC 0.659381249258

ATGAAAGTCAATCAACTGCTTG 0.399666786785

ATGAACGAAGGTCCAAGTTTAG 0.295949029993

ATGAACTAGTCTCTACAATAAG 0.356720720525

ATGAAGGCGCGTGGAATCGCAT 0.76660241605

ATGAAGTTCGGTCATCGCACTC 0.554434061699

ATGAATGCATATCTTTCGAAAA 0.318993135558

ATGAATGCCTGTCTTGACCAAC 0.554082970908

ATGAATGCTTTTGACCTCCCAT 1.098340836

ATGAATTACTGTCCGACGATAC 0.369040180541

ATGAATTTGTGCCGTCGACAAG 0.820458341452

ATGACACAGAATCCATGGCAAC 0.453252188988

ATGACACCGGCTGAATAGGAAA 0.861855020978

ATGACACGATGTGTCCGGGGAG 0.362841660263

ATGACACTCTTTACATTCTTAT 0.217783687221

ATGACAGCATCTGCTAGCAGTG 0.699597209126

ATGACAGTAAGTGATTGAGTAG 0.627657080029

ATGACAGTAATTGAGTCGGCAC 0.928874417175

ATGACAGTATGTGGCTCAAAAC 0.708324126861

ATGACAGTGTGTCATTAGTAAT 0.647711897602

ATGACAGTGTGTCCTTAAGAAC 0.211982077268

ATGACCCAATTTCCAACCGGAC 0.388814899622

ATGACCCAGTATTTTGGGGAAA 0.853722181054

ATGACCCTTGCTGCAGAAGGAA 0.73928859616

ATGACCCTTTATCCTTTCAAAA 0.539471889495

ATGACCGAATCTCGTGGTTCAG 1.23653372313

ATGACCTAATCTGATGTTGTAC 0.390746315452

ATGACGCAAGATCCCGGCTAAG 0.50333664498

ATGACGCAGTTTCTGACAGCAC 0.308424290853

ATGACGGAGTGTGATTCAGAAT 0.740633225174

ATGACGGGTAGTGAACATAATA 0.447220241079

ATGACTCTATGTCATATGTTAG 0.432486423788

ATGACTCTCTGTGAGACCGTAC 0.649621683275

ATGACTGCACCTACCAATCATA 1.25371793796

ATGACTGCATCTCATGCCGCAA 0.895427223547

ATGACTGGCCTGCTTTAATTAT 0.550323884209

ATGACTGTAGCTCGTATCTAAA 0.569606908504

ATGACTGTGACTGAAGGGCGAG 1.51754675823

ATGACTTATTGTGTTTCTAGAG 0.416392543732

ATGACTTTGGCCAATCGCCATC 0.672079638074

ATGAGAATGGGTCTTCGGTCAG 1.1298184589

ATGAGACAACGTGGATCGCAAG 0.658087745545

ATGAGACAATATCCTACCGAAC 0.364528647625

ATGAGACAGACACCAGATTAAT 0.273243501296

ATGAGACGAGGTGCTTGCCAAT 0.732349477421

ATGAGACGATTCCTTATAGAAC 0.591825632119

ATGAGAGAATCTGCTACAAGAT 0.204898627827

ATGAGAGAGTATGTTAGGTGAG 0.680088162367

ATGAGAGTATGCTCTTAGCCAT 0.605473725634

ATGAGATACACTGATAGCAAAA 0.423404068866

ATGAGATACTGTCCAAATGCAC 0.410510750429

ATGAGCCGATATGCTTGAGAAC 0.773067120981

ATGAGCGAAGATGTTCAGAAAA 0.387212715131

ATGAGCGCAGCTCGTCGCATAA 1.00156962596

ATGAGCTCCGATCAAGAGAAAG 0.294001793894

ATGAGCTTCCCTCAGATTCGAG 0.551457260951

ATGAGGCATTCTCCGTTACATC 0.178376658627

ATGAGGGGATTTGCTGTAACAA 0.31267155208

ATGAGGTAGGGTGATCTCATAG 1.46761115323

ATGAGGTTCTGCGCTCCACGAC 0.754623004135

ATGAGTAAAACTCATAGAGAAC 0.103114933125

ATGAGTCACAATCGACAAATTT 0.293464685763

ATGAGTCACACTTATTTGTTAA 0.175147442572

ATGAGTCATCCTGTGGCAACAG 0.569001007612

ATGAGTCCACCTGATAGCCAAG 0.858542947915

ATGAGTCTAACTCCTAGTTGAT 0.589855286253

ATGAGTGAATCCGCATCTTGTC 1.25780141422

ATGAGTGAGCGTGACGATGTAA 0.954150006243

ATGAGTGAGGGAGCTAAGTTAG 0.364228691835

ATGAGTGAGTTTCTAGCTTTAT 0.511450673769

ATGAGTGCACGAGCGCCCTTAT 0.97809185865

ATGAGTGCGTATGCTGTGAGTC 0.601617107624

ATGAGTGTGCCTTAATCGCGAA 0.705750199761

ATGAGTTCGGCTCGCTATGGAA 0.754377554922

ATGATACTATACGCCCTACAAT 0.538682716982

ATGATATAAACACCGGCTGAAT 0.483137281012

ATGATATCAGGTCGAATTGAAC 0.400939629621

ATGATATTAACTTGGCCATAAC 0.259682844139

ATGATGCAATGTAAAAGAGCAT 0.268157841145

ATGATTGTACGTCATTGGCGAG 0.285365786122

ATGATTGTCTGTTCTAGATCTT 0.372264397515

ATGATTTTCGCTGTTCCGATAA 0.70167787075

ATGCAACCCTCGCCTACGTCAT 1.0588698212

ATGCAAGTGTCTGCTCACTAAG 0.692168711475

ATGCAGTTAATTGAATTGAGTT 0.367381192189

ATGCCAAGCTCCGCGCAGTAAT 0.798840393703

ATGCCACCTTGTCCTCAAAGAG 0.32351204921

ATGCCACTATGTTGCAGGCAAG 0.794680467735

ATGCCACTCGGCCCATCTTTAT 0.891355846394

ATGCCAGAGAGCCACCGTATAT 0.935303486807

ATGCCAGCTTATCCTTGGTGTG 0.642134315376

ATGCCATCAGCTCTAAGCCATT 0.664485146235

ATGCCCAGCTGTCCATCCAAAG 2.94450913156

ATGCCCCAATCATGAATACGAG 0.448855530729

ATGCCCGTATCTCCGTTAGAAT 0.430172335255

ATGCCGGGATGTGCAGAGAGTG 0.342364954225

ATGCCGGGCTCTGAGTAGTAAG 1.69922822891

ATGCCTCGCGGTGCACCGCATA 0.886311638797

ATGCCTGAAGCTTGTTCTTCAT 0.555487077133

ATGCCTTACTGTGCGGATCTAA 0.485502752098

ATGCCTTTATCCTCTTGTACTA 0.478805829434

ATGCGACAATCTGCTGACTTAG 1.24603584058

ATGCGACACTCTGAGCCATGTT 0.727777626915

ATGCGACGCTGTGCTATGGTAG 0.578657400577

ATGCGAGACTCTGCTAATAGAA 0.800940517657

ATGCGAGCATGTGAATACTCAG 0.664857270768

ATGCGAGCCTGTGCAAGGATTG 1.02730826005

ATGCGAGGCTGTGTCAATCATA 0.707871811365

ATGCGAGTCCGTACTCTGTAAT 1.33719155999

ATGCGATAGTGTCAAGGTCCAT 0.982584318233

ATGCGATCATGGCCTGGGAGAC 0.847744937777

ATGCGATGGCGTCGTGGAAATG 0.17264605867

ATGCGATTACCTGCAATTCTAA 0.594926156333

ATGCGCCCCGGTCCATTCGAAG 0.645322939279

ATGCGCCTAAGTGCGAAACAAT 0.570168316727

ATGCGCGCAGGTGCTGCTAAAC 1.90590773102

ATGCGGCCCTGTGATTAGTATT 0.945144613583

ATGCGGGGATTTGAGCAACTTT 0.68228111567

ATGCGGGTAAGTCAAGATTAAC 0.596001116088

ATGCGTAAGTCTCATGGAAGAC 0.77843759293

ATGCGTCCCTGTGATCTATTTT 0.836408816797

ATGCGTCGCTCTTATACCGAAG 0.509485653568

ATGCGTCTACCTTGTCGCCTAG 2.0255804496

ATGCGTGAAGCTTTAGGGATAA 0.624532270204

ATGCGTGAATGTAGATCAACTA 0.762046565782

ATGCGTGAATGTCCATCAGGAG 0.51105352507

ATGCGTGGTTCTGATGGTTGAT 1.52423972014

ATGCGTGTTTTTCGAGATCAAG 0.330830084757

ATGCGTTAATGTGCAACCTCAC 0.768598837014

ATGCGTTACACTGGTCTGGAAT 2.68919198559

ATGCGTTTATCTCGAGGCAGAT 1.32016554027

ATGCTACAATGTCAACCGGGAC 1.38636617288

ATGCTACTGCGTCCTACATTAA 0.916456351816

ATGCTATAGACTGAACAAAGAC 0.199842674451

ATGGAACACTCTGGTAGTTAAG 0.332258803948

ATGGAAGAACGCCTTGTATCAC 0.301579133096

ATGGATGTATATCATGAATTTG 0.587496833335

ATGGCAACCATTCCTGAAATAG 0.354464725515

ATGGCAATTTGTCATCCCACAG 0.99476327923

ATGGCACAATGACCTTGACAAG 0.77909661329

ATGGCACCCCGTCGTGACTGAA 0.543917862216

ATGGCACTATCTCCGTGTAAAA 0.655298811167

ATGGCACTCTAACCTGGAGAAG 0.676850972408

ATGGCCAGACGTCCGAACCAAT 1.20772071753

ATGGCCGACGATGGATACCCAG 0.922362478295

ATGGCCGCAACTCAAGGGTCTG 1.14896163631

ATGGCGCATGATATATGTTAAT 0.458888195382

ATGGCGTCCACTGTAGTAGTAG 0.82032572669

ATGGCTCAATGGCAACAAGTAA 0.297706836426

ATGGCTCCATCTGCTCGCCTAA 0.995886094465

ATGGCTCGAACTCCACATCATG 0.389792636279

ATGGCTTAAACTCATACGAGTG 0.297925327593

ATGGCTTATACTTATGGGGGAA 0.406038047659

ATGGCTTGGTGTCCGACACGAC 0.315905489365

ATGGGACGTTTCGAAGATCGTA 0.550890972958

ATGGGAGAATGTGCCAGTCCTT 0.898813040742

ATGGGAGTCTGTTATTCATGTG 0.296082103383

ATGGGATTCGCTGAGCACCCAG 1.34730332587

ATGGGCGAAAGCGTCAATCGAA 0.751248579397

ATGGGCGACAATCCTACGATAC 0.802164525649

ATGGGCTATACTCATGGCTTTC 1.32994902022

ATGGGCTCATCTGACCATAGAG 0.639688469125

ATGGGGGGATTTGACCCATAAA 0.984048935323

ATGGGGTCAAATCCTCAATTAA 0.420541882794

ATGGGTACATGTACTACCTAAA 0.436273850998

ATGGGTCTATATCATTTGGAAT 0.362375189628

ATGGGTGAACATGTCGATCCAT 1.04200362688

ATGGGTGATACAGTAGGCTCAA 0.81970283692

ATGGGTGCATGTGTAGCCCATG 1.24300442698

ATGGGTTTGCCTTCGAACCCAG 0.628928119104

ATGGTAGAATCTGATTATGGAT 0.670888649939

ATGGTAGTCATTCCTAGCTAAG 0.900809965554

ATGGTATAGCATCGTAAATAAT 0.477039712059

ATGTAACAAACTTTATAGGTAA 0.265587854424

ATGTAACAGTAATGAACATGAA 0.392101726452

ATGTAACCGACTCCTCCCGCAG 1.1583903989

ATGTAACTATCACGTAATGTAC 0.19802676005

ATGTACGAACGTCATGTGGAAG 0.659295151946

ATGTAGGAATCTGCGCTACAAA 0.374336620088

ATGTAGGATTCTGGTTGAACAG 0.214509231965

ATGTATATAATTCTTGACGCAG 0.129658039287

ATGTATCGATGTCATCGACGAC 1.30270881706

ATGTATGACGTTGATCCGAATC 0.766956869001

ATGTATGTTAAACCTATAGAAC 0.21937222942

ATGTCACACTGTGTGTCGAAAA 0.440758531543

ATGTCACATCGTCATTTCGTAG 0.844602238172

ATGTCACTCAGTGAACGGAATG 1.03125830983

ATGTCAGCATCTGCGTGTCAAA 0.753969922844

ATGTCATCGGGTGGTTACTAAG 0.630638150178

ATGTCATGTTCTGCTGTCACAA 0.797342672641

ATGTCATTATGGGGTCGCATAA 0.999554546471

ATGTCCAGGGGTCATTAGATAT 0.329940933002

ATGTCCCCACTTCTTTTGTGAA 0.557978177522

ATGTCCGTAGTTGCCACGGCAA 1.15461387367

ATGTCGCACATACATATGACAA 0.395909664863

ATGTCGGAAACTCATGTAAAAT 0.265845927496

ATGTCGGTATCTCCGTTAAAAC 0.548457703119

ATGTCGGTATCTGCTATAGAAT 0.631305709361

ATGTCGTCATCTGATTAGTCAC 0.494055887302

ATGTCTCAGTCTGGAAACACTT 0.56529261585

ATGTCTCGACCTGTTACTACAA 0.358069810619

ATGTCTCGATCTGTAACCTAAT 0.428961584896

ATGTCTGATTTCGAGAATCTAT 0.629826045728

ATGTCTGTATGTAAAACCGAAA 0.351453645257

ATGTCTGTATGTGGGCGTTATT 0.312814518812

ATGTCTTACCCAGATAAGAAAG 0.379819090337

ATGTCTTTCATTCCTTCTCAAC 0.908119344361

ATGTGACAATGTAATAAATAAA 0.309177870604

ATGTGACAATGTGATCTGGCAA 0.540394963624

ATGTGACCGAGAGACGCTAAAT 0.856216718877

ATGTGACCGTCGCTATCTTAAC 0.782983248258

ATGTGACCTTCTCAACAAATAT 0.455257148228

ATGTGACGATCTCCGGGCGAAG 0.612866322764

ATGTGACTATGTATAAAGTAAA 0.511444399926

ATGTGAGAGATTGGAACGCCAG 0.778868986605

ATGTGAGCACGTGATGCATGAG 1.15652254499

ATGTGAGCGAGTCCTGGCAAAT 0.866089897602

ATGTGAGTAAGTGATGGTTAAT 0.505081628536

ATGTGAGTATCTGATCACAATG 0.612697386616

ATGTGATAGTCTCCTCGCTTAT 0.45925802523

ATGTGATCATAGGCGATATATT 0.555225803236

ATGTGATCCCCTTGTGGATAAC 0.903793649962

ATGTGCGACTGTGAGCCCCTAT 0.595680094231

ATGTGCGCATTTGATGGTCCAC 1.16478477515

ATGTGCGCTCCTCGAGAAAAAC 3.03914185636

ATGTGCTAATCTCCAAACGAAG 0.583249352654

ATGTGGGACACGGCAGAGACAA 0.219915595323

ATGTGGGTACCTGATGGAAATC 0.582738822763

ATGTGGGTATCTCATGTACATG 0.562774667916

ATGTGTGAAGGTCATTTCAAAA 0.292648075601

ATGTGTGACTGTTATTAACAAT 0.436857864114

ATGTGTGTAACACTTGTCTCAG 1.03746856468

ATGTGTGTCTCTGCTGGCGAAC 1.3628553496

ATGTGTTGTACTGACGAGGTTG 1.13729475283

ATGTGTTTATCAGCATGGAGAG 0.328712731113

ATGTTAGCAACTCATAAACTAG 0.289800040494

ATGTTAGTGCTGCATAAAGTAT 0.61064121772

ATGTTATATGCTGCTAAATTAG 0.558611524012

ATGTTATGAGCTTGTATATGAC 0.458660195789

ATGTTTGAATATGCAAACGATG 0.283095609391

ATGTTTTTTATTCCAAACTCAT 0.740544468235

ATTAATTGTCCTTAAAAAAATG 0.326788532386

ATTAATTTAGCAGATCGAGCTA 0.400985090418

ATTACAAGATCTTTAACGCTTA 0.513571879707

ATTACACTAAGTCCACCCCATC 0.69906767361

ATTACCCGACGTGCAGGCGCAC 0.657754849105

ATTACCGGGGGTGATATTATAT 0.926870082755

ATTACGCACTCGCTATTTTAAT 0.546260222721

ATTACTCGATATGATGAAACAA 0.536780324917

ATTACTCTCGCTCGCAGGTTTC 0.555750395973

ATTACTGAATTTCGTCACTCAG 0.351678661872

ATTACTGATTATCGTCTCTTAT 0.16619730837

ATTACTGATTCTAATTCCTGAT 0.286199608321

ATTACTGCGTGTCCTTGTTTTG 0.264291016017

ATTACTGCTTTTGATCTGAGAC 0.194400010827

ATTACTGTAATTCAACCTTAAG 0.336183159825

ATTAGACAAGATCCTTGGAAAC 0.518666909844

ATTAGACCATCTGAGTACAAAG 0.596529728272

ATTAGACGACGTCAAATGAAAA 0.39246357636

ATTAGAGAACATGTTACAAAAT 0.33974575524

ATTAGAGAAGGTCATCGTATAA 0.403684544256

ATTAGAGCCCGTGATGTGTCAC 1.1299093662

ATTAGAGCGTTTCAATCAAAAT 0.498412599748

ATTAGAGGATTTGTTAAGCAAT 0.400947597744

ATTAGATTACGATTGGACCAAA 1.00497701342

ATTAGCATTCTTCGTAGCGATA 0.82636896104

ATTAGCCCGCCGCAAACGCAAG 1.22147264052

ATTAGCTACTAAGAACCGGAAA 0.459871360946

ATTAGCTCTTCTGCCGGTCGAT 0.938731363305

ATTAGGCTAAGTGATTGAAAAG 0.382509946306

ATTAGGTGATCTGACGACCAAC 1.11771378451

ATTAGTGACTGTCAGTAATTAT 0.282058688134

ATTAGTGGATGTGATTTGCAAG 0.603604738936

ATTAGTGTATATCCTTTGACAA 0.630997507177

ATTAGTTAATCGTATACAGGAT 0.43440862008

ATTAGTTACCGTCCTGAGAGAG 0.416997924823

ATTAGTTCCCGTGCCTTGCGTA 0.657782835837

ATTATAGAGGGTTCTTACAAAA 0.268629352309

ATTATGCGATCTGATCAGTCAC 0.743395140217

ATTATGGAAGCTCCTTAAAAAC 0.601819316366

ATTATGGGCGATCCAATCTAAG 0.788455935022

ATTCAGTCCCGTGTCAAGATAC 0.794898299935

ATTCATGCAGGACATCAAAGAG 0.564595996011

ATTCCAAAGCTTGGTGTTAAAA 0.473645598535

ATTCCAAATCCTGTACGCGGAT 0.362535591434

ATTCCACAAACTGCTTCCGTAC 0.3252003181

ATTCCAGACACCCCGCGGGAAC 1.50778349877

ATTCCAGGCTCTGGAGGGGGAT 0.790042650085

ATTCCAGTATATCAACCTACAC 0.282706897025

ATTCCCGCAACGGGTTTAAGAC 0.41468510166

ATTCCGGGATGTCCCGACGGAT 1.02342203593

ATTCCTAAAACTCCTTTCCGAT 0.639419139201

ATTCCTAACGCTGCTCTTCAAG 0.967227253451

ATTCCTGAAGCTCCTAAATCAG 0.454874799352

ATTCCTTTGTTCGAAAGCACAC 0.228752121594

ATTCGACAATCTGGTGCTATTT 0.23639782697

ATTCGACCCTGTCATCCATGAA 0.745892154163

ATTCGAGCATCTTATCTCCAAA 0.421247128388

ATTCGAGCCGGTGGTGATCGAG 1.05578638841

ATTCGAGGAGGACATGGCCAAG 0.49788817925

ATTCGAGGATGTCCTACGATAT 0.396245529365

ATTCGAGTTGATGATGGCGGAA 0.44341069655

ATTCGATAATATCCAAGTAAAT 0.222526650776

ATTCGATACCCTCATTATGAAT 0.572830199575

ATTCGCGGCTGTCCATTACCAC 0.378549600969

ATTCGGGCAACTGAGGTGTCAA 1.26959410395

ATTCTATAAACTCGTTTGACAA 0.347243137515

ATTCTGTTGTGTCAAAGTCGAA 0.662192024545

ATTGAACATCATTAACGTTATA 0.275144111846

ATTGATCCATAGGCTGTGATTA 0.440875743683

ATTGCACACTCACAGTCCTTTA 0.253027747394

ATTGCTCAGATTGATTGTTTAC 0.347322473833

ATTGCTCAGTATGTAAACGAAC 0.16489492612

ATTGCTCCGATTCCAAAAGAAT 0.312346561726

ATTGCTCTATCTCAAATAATAT 0.521474803623

ATTGCTGTTACTGACAGGTTAT 0.292486728336

ATTGCTTACGCTGCGAAGAAAG 0.376937116463

ATTGGAGCATTTACACTGCAAT 1.45956897359

ATTGGATACTTTGAGCTGAGAG 0.724256822932

ATTGGATAGCGTGCTGCTGGAT 2.0834769518

ATTGGCCACGCTCTGTCGAAAT 1.40818527664

ATTGTACAATGTCTAAGGTCAG 0.522689562387

ATTTAATGACCTGCTCGAAGAT 0.312854336603

ATTTACGTTGCTCCGCTCTGAG 1.66808725962

ATTTAGTAGTGTCCTAACGCAG 0.682550313083

ATTTATGAACGTGCATAGATAG 0.633627101727

ATTTATGTAGCCCCACCGATAG 1.03591388674

ATTTCACGGGCTGAATTCGTAA 0.436930326486

ATTTCACTGTGGGATTGTTAAC 0.634454441438

ATTTCAGAAAGTCATGGCTAAA 0.332861237781

ATTTCAGAATTTGAAATGATAT 0.18453787607

ATTTCAGTATCGCCCAGAATTC 0.282225262711

ATTTCATAATCTCATATATATG 0.622465445422

ATTTCCCTGAATGCTCTGAGTG 0.751261090336

ATTTCCTCGGGCGATGAAACAA 1.20053559009

ATTTCGGGTTCTGATAGCAAAC 0.875420874478

ATTTCTCTGAGTTGTCTGTAAA 0.708132541562

ATTTCTGAAACTTATGAAAATA 0.250902098676

ATTTCTGAGTGTGTTCTCCCAG 0.675212978226

ATTTCTGCCCATGATAGGGTAG 1.04866705959

ATTTCTTAATCCCGTATGCCAG 0.50304543171

ATTTCTTGCTGAGGCTGGGAAG 0.479708234073

ATTTGACAGTTCCTTCGCTAAT 0.15584718902

ATTTGAGTGTATGGTGGTTAAA 0.325286367996

ATTTGATTGTCTCCTTTGTGTT 0.777404800599

ATTTGCGTACGTGGTGAAGGAA 0.364101702276

ATTTGGCTTAATGCGAACTCAG 0.740351126404

ATTTGGTCATCTGCTTGTGCAA 0.768044953273

ATTTGTAAAGCGGCTTTAGTAC 0.329184981757

ATTTGTCAAGCTGTGTGTCATG 0.689705736484

ATTTGTCTATATGCTGAGATTT 0.281458835471

ATTTGTTATACTGCATAAAAAT 0.478262561708

ATTTGTTGAGCGCTTAATCGAA 0.59784150413

ATTTGTTGCACCCCTCATTTAA 0.847873618483

ATTTTAGAATCCCAATCTGCAG 0.915615050813

ATTTTAGTATCTCAAAGTGAAT 0.607849695695

ATTTTCTACCGTCGGAATTTAT 0.492584873582

ATTTTTGCGAGTCTGTCATAAC 0.723954479286

ATTTTTGCTTCTCGGGCACAAC 0.993449547811

CAAAAAGTATTTGCACTCATTA 0.334594879878

CAAACATAAGGTCTTAATGTAG 0.466444810131

CAAACTGTACCTGCAGAGTAAT 0.744659188598

CAAAGATCATGTGATACAGATT 0.330335674645

CAAAGTAGATGTCTATGCGAAA 0.602162340822

CAACATTACAGTTCATATGTAG 0.484484595139

CAACGATTATGTGCTAGGTGAG 0.293412763922

CAAGGACCCGTGGCTATGCTTC 1.12433770452

CAAGGTGAGTTTCTGTACGCAT 0.532680019856

CAAGGTTAGTTTCATAGAGCAA 0.41259585951

CAAGGTTTCCGTCATTGTTGAT 0.255334250383

CAATGATAATCTGGTTAGGTAT 0.427295720486

CAATGTGGTTTTCTTTATCTAA 0.51783452437

CACAAACGAACTGCATAGCAAT 1.62791918727

CACAAACGGCCTGTTTGTTAAG 0.403298209717

CACAAACGGTCACGTTGCGGAC 0.560904658347

CACAAACTTGCTTGAAGGCATG 0.138507481578

CACAAAGATGGGGATTGCATAG 0.587453051427

CACAAAGTATGTCAGCGGTCTC 1.13592440039

CACAAAGTGTGTCAGCCGACAC 0.478543763066

CACAAATAACGTGCGGTTTCAC 0.268271305342

CACAAATAATGTGTACGGAATA 0.44581752483

CACAAATGAGATGAAGCTGAAT 0.317660176571

CACAAATGTGGGGAAATTTGTT 0.962995337924

CACAACCTCGTTAATTCGGATG 0.712871668818

CACAACCTTCCTCTTTCTATAA 0.486475001319

CACAACGACGGTCGTAAGTATA 0.593259680866

CACAACGATGGTCGTGAGATAG 0.490583195958

CACAACGCAGGTCCTAGTGAAT 0.776198786944

CACAACGGACCTCGGTGCCGAA 0.477172483325

CACAACGTATGTCCTAGGACAG 0.976149580602

CACAATCAGTGTGACGATGTAA 0.727050944818

CACAATGAACCTCATTTATTAC 0.282660566816

CACAATGGAAATGCAAAGGATG 0.463443585226

CACAATGTCTTTGAATAAGCAG 0.507325178368

CACACAAATTCTTGAGGGAAAC 0.413271244239

CACACAACGAAACCTTCATATA 0.43168996167

CACACACAATGCCTCGGTCAAG 0.631606483397

CACACACCATGTCGTTGGGCAC 0.685834922289

CACACACCTTATCTTGTTCAAT 0.339519235854

CACACACGACCTCACAAAGAAA 0.929586840358

CACACACGGCCTGACTCCCAAT 0.585522615698

CACACAGAACCACAATTCAGAG 0.395143815589

CACACAGCACATTGTCATAGAG 0.366348469766

CACACAGCGATTCCTAACCGAA 0.308185506481

CACACAGCTTCTGATACCGAAG 0.998499020173

CACACAGGATCAGCTTCAACAC 0.414613201756

CACACAGTAGCTGTATCAACTG 0.789673901961

CACACAGTTGTTGTTTTGTGAC 0.640708212146

CACACATAAGCTGCTTTTGAAA 0.486670941761

CACACATACTCGCATGAATTAG 0.367162930386

CACACATACTGAGCAACAGGAA 0.204962885799

CACACATAGAGTCATGTTCAAG 0.306280006979

CACACATGAGGTCCTGGGGATG 0.675877705785

CACACATTACGGTCGTGGTTTG 1.46533821045

CACACATTAGTTGCACACGTAT 0.523773615142

CACACATTCTCTCCTTGCTAAG 0.668256064759

CACACATTCTGTGCAGGAAATT 0.567497767047

CACACCGAATCTCCACACGAAG 0.537924412422

CACACCGTGGTTGATGGCGGTA 0.646982458752

CACACCTACGTCCAATCACTAG 0.686344800239

CACACCTAGTATGCACCTCTAG 0.690977318507

CACACCTGGCGTCTTCCCGTAG 0.510381523699

CACACGAACGCTGCTATGTAAC 0.565364886188

CACACGCAAAGTCAAGAATCTT 0.289200593133

CACACGGAATGTGCTTAAGGAG 0.199819914936

CACACGGGTTGATGTCCTATAT 0.298954954019

CACACGGTAGCTTAAAGCGAAC 0.483826198232

CACACTCCAACCGTATTTCTAA 0.391685190684

CACACTGACTCTGCACCCGAAT 0.705000189544

CACACTGTAGGGGCTTCTTCTA 0.757615875409

CACACTGTATCTCCAGGCAGTT 0.420742969008

CACACTTAGCATGATGCCTAAT 0.669807682935

CACACTTCCTGTTATTACGAAT 0.342499038727

CACACTTTAAATCACAATCTAG 0.206769863058

CACACTTTAGGTGAAGTCATAA 0.207632000652

CACACTTTCTCTTATATAGAAA 0.415311782099

CACAGACAAAGTGACAAATTTC 0.131687712679

CACAGACCAATTGTTTACAAAT 0.47850201339

CACAGACCATGTCATTTCGATA 0.299660549226

CACAGACCCTCTGCTGTCTCAA 0.543283311938

CACAGACCGTGTGAACCCTTAA 0.373645856536

CACAGACCTTCTCCTGTCGAAT 1.10198411168

CACAGACGGAATCATAGGCAAC 0.211911601648

CACAGACGGACTGATCCCCAAT 0.5024363716

CACAGACTATGTGCAGTTTAAT 0.445090558839

CACAGAGAATGTGTTGCCTGTC 0.716213807916

CACAGAGAGCGATGCAGATAAT 0.292303095458

CACAGAGATAATGATGATGATA 0.511548815114

CACAGAGCATCGTAAGCGCAAT 0.502337967536

CACAGAGGACTTCATTTACATC 0.534839973954

CACAGAGGATTACAAATTTAAA 0.392117061963

CACAGAGGCTGTTTGGCCTTAG 0.820969305999

CACAGAGTAACTGATTCGATTG 0.398355813757

CACAGAGTAGCTGAGATAAGAT 0.32001208404

CACAGAGTCAATCATAACTGAG 0.875604337979

CACAGAGTGACTCAAACCTCTG 0.374266448319

CACAGAGTTTCCCCTAGCTTAA 0.622686622584

CACAGATAATTACCTGGTAAAC 0.700914807149

CACAGATACGGGTGTCTAGTAA 0.793265143012

CACAGATAGTATGCTGCCTAAT 0.480029613556

CACAGATATCGTCGTTCATCAA 0.216291311573

CACAGATTCTCACGTTAGTGAT 0.502529355015

CACAGATTCTCTGGTGAAGTAA 0.424261993576

CACAGATTGTGTGATGAAAAAC 0.489644363106

CACAGCCGCTGGGTGGCATGAT 3.24893697815

CACAGCGTCAGTGCAGCAGGTC 0.781669253066

CACAGCTTAATACATTGGTTAC 0.559500962382

CACAGGCCCCTTGATGGTTTAC 0.529798327169

CACAGGCGATCGGTAGGGACAC 1.43303359274

CACAGGGAATCTCTCAGATTAG 0.327190834686

CACAGGGCACCTCTTTTGATTG 0.247809961473

CACAGGGTGTGTGGTAAAAAAT 1.14598356912

CACAGGTCCTAGCCAATACAAG 0.338673193012

CACAGTCAAGATGAAGGTATAC 0.637162516263

CACAGTCACTTTGCGAAGGTAC 0.991552097333

CACAGTCCCTGTTTACAACTAC 0.540182639206

CACAGTCGTCCAGCAGAACTAA 0.484064167163

CACAGTCTGACTGCGTGATAAC 1.26345251398

CACAGTGAATCTCAAGAGTATG 0.629960188615

CACAGTGACAGTTCAACTTGAG 0.528178746757

CACAGTGACTCTAGTAGGCAAA 0.381292625433

CACAGTGCATATCATACTTCAA 0.492880767629

CACAGTTAATATCGTCCTTGAG 0.369945054768

CACAGTTAATCTGTAGATTAAA 0.223315045729

CACAGTTCATTTTAAGCAGTAC 0.348622186454

CACAGTTCGACTGCAAATCAAA 0.287654515833

CACATACGATCTCCAGAGGAAG 0.826322672619

CACATACGGACTTCAAGGTCAG 0.76654985898

CACATAGTAAGTGCCTGGATAA 0.41325891675

CACATCGAACCGCTAGAGGGAG 1.2118676332

CACATCGTGTCTGCTCTCACAC 0.587842889759

CACATCTAACCTGCATTAATAT 0.229785703185

CACATGCGGTATACAGAGAGAC 1.20377621301

CACATTTAAGGTGCTGTGCAAG 1.0645038994

CACATTTCATCTCGTGAGAAAA 0.36599873052

CACCAAACAACTGCAAATGGAT 0.174259289348

CACCAAGTCTGTCGACTTACAC 0.464300617313

CACCAATTAGATGCGTCTACAC 0.398750049154

CACCACCTATCAGCCGGAAAAG 0.506504393904

CACCAGGGCGGTCTTGGTAAAG 0.84469293488

CACCATCCATTTAGTGACCTAC 0.491067915357

CACCATGAGCGTACTGCAAAAC 0.272766656294

CACCATGCCGCTGATTATCGAC 1.47960795938

CACCCAATTGGTGCTTCGGTAC 0.58908422047

CACCCACAAATTCGACTATAAC 0.440380663366

CACCCAGAGCGTGCAGAGCCAG 1.08761258865

CACCCAGAGCGTGCTTCTGGAA 1.16289835046

CACCCAGTAAATGCAGGCAAAG 0.529998430874

CACCCAGTAACTGCTAGCAGTA 0.65509452632

CACCCAGTATGTCCAACAGGAA 0.949254037458

CACCCAGTCTCTCCTAAAGAAT 0.427345582908

CACCCAGTGGTTGCTTTGGGAT 0.525335895623

CACCCATGATCTGCAGGCCGAG 0.639092899904

CACCCATTATCTCATTGTCAAT 0.32706917252

CACCCTCCACGTCGTTACGTAT 0.444204424838

CACCCTCGAAGTGTTAATCTTC 0.407060210424

CACCCTCTCGCAGATGCGCTTA 1.20485616741

CACCCTGATGCTGATTGAGAAC 0.524663218771

CACCCTGGAACTCATATTAAAA 0.419332363124

CACCCTGTCTCTGAACGGTGAA 0.509679919493

CACCCTGTCTTTGAAGTAGGAG 0.460425089076

CACCCTTAACCTCTTACAGAAG 0.534945103922

CACCCTTCATGTGAAGGCGTAG 0.565674118996

CACCGAAGACATGCTATATTAG 0.616061472551

CACCGAATTAGTTCAAACAAAA 0.302714401228

CACCGACAAAATAGTTATCAAC 0.652652746439

CACCGACATAGTGGAATCCGAT 0.345526114669

CACCGACTAGGTGCAACTCCAA 0.957592901477

CACCGACTGCCTCGTTGGCGAC 0.333362665451

CACCGACTTCATCGTCACCGTG 0.805312460665

CACCGAGAAACTCAACCGGGAT 0.722364375427

CACCGAGCTACTGCTGTTGTAG 0.141719086989

CACCGAGGGGTTGGTACGAGAA 0.957421612905

CACCGATATTGTGTACGGAAAG 0.633382734513

CACCGCGGATATTTTCATTCAG 0.572245945246

CACCGCTAATGTCGAGAAGAAA 0.544884493553

CACCGCTACTCGGCTCCAAAAC 0.476399475069

CACCGGGAAGTGGGATTCTAAC 0.692829414846

CACCGTACATGAGAACGCCCTG 0.720054361998

CACCGTAGCACTGGTAGTTATG 0.590346621204

CACCGTGCTTTTGATAGGTTAT 0.595988817642

CACCTAAAGTCACTAAGCGTAA 0.405336223951

CACCTATATCCTGTTCCCCCAG 1.03130084554

CACCTATCTGGTGATTTGGTAC 0.492260130934

CACCTCCAGACTCCAGACGGAG 0.620606485891

CACCTTCAGCTTCGGAGTAAAT 0.577162148395

CACCTTGGATCTCTTTTTAAAA 0.330987112098

CACCTTTTAGTTCATAACTCAG 0.472266707918

CACGAAGGCTCTCAGAGGAATA 0.522732532683

CACGAAGTGAGTGATTAAAATG 0.774283104774

CACGAGGAACCTCCTTCGATTG 0.992828693203

CACGCACAAGCAGCTGCTTAAG 2.84951842168

CACGCACTATATGCCTACACAG 0.767417938358

CACGCAGAAGGTGCTTATAAAA 0.276669339759

CACGCAGTATCTGGTATCGAAT 0.537526967325

CACGCATAATGTGGTCTGAAAT 0.553712495404

CACGCATATCATGATATGGCTC 0.512255926828

CACGCCATGTTTTACGTTACAT 0.44939750841

CACGCCGACTTTGAATAAGGAT 0.32768678595

CACGCCTATTCTTCATGTAATA 0.405371051073

CACGCTATCACTGAAACTCTAC 0.206350642312

CACGCTCGGAGTTATGAGCTAC 0.771478979475

CACGCTGAAGGTGTTTAGATAC 0.718980936935

CACGCTGCTTGTCGCCAGCTAT 1.19384105187

CACGCTTACGGACATGCTCAAG 0.728515215009

CACGCTTTCTGTGCACTCGAAT 0.575796453015

CACGCTTTTTTTCGATCGTGTG 0.914034847484

CACGGAATATCCGATTGGGAAA 0.422958844367

CACGGAATGTATGGTCCTATTA 0.268485735112

CACGGAGACCCTCCTACCCCTT 0.695417590935

CACGGAGACTGTACAAGTAAAG 0.477856886811

CACGGAGTAGTTGGACGAGTAG 0.818145112234

CACGGAGTGCATCTTAGAAGAC 0.953336606521

CACGGATAAATCGCAGAATAAA 0.454099552408

CACGGCTATTGTCCTAATCAAA 0.464167379979

CACGGCTTTTGTGAAGGCTATA 0.886803655678

CACGGGAATAGTGATGCCAGAT 0.516498897317

CACGGGGGCTATGCATAAGAAA 0.589379591971

CACGGTATTGTTGGACAATAAC 0.564962811772

CACGGTCAAAGTCGATTAGAAA 0.673381123586

CACGGTCGAGTTCGTGAGAAAT 0.513940528778

CACGGTCTCTGTTCTTTCCTTG 0.236182026857

CACGGTGCATGGCATGGCCTAA 0.657975498374

CACGGTTAATCTCTAGGATAAA 0.416996519132

CACGGTTGAAATCGATACGGAG 0.523638086138

CACGGTTGATCCGTAGAGGAAC 0.874456315608

CACGGTTTGACTGACTCACAAT 0.785773546177

CACGTAAAAGGTCACTAAGCAC 0.571717720078

CACGTACACTCTCTGGAAGGAC 0.860170564742

CACGTACAGTGGGTCATGGAAA 0.939747434363

CACGTGGAAATTCCAACATTAG 0.242461632586

CACGTGGAAGGTCATACGCATA 0.379752139587

CACGTTCACCCTGTATACGCTG 0.193216511772

CACTAATAATACGCAGGTGAAC 0.746697861402

CACTATCGACGTGTAGTTCCAC 1.052890724

CACTATGTATGCCATCCTGTAG 0.918542482625

CACTATTGTTTTCGTTCGATAT 0.569531719424

CACTATTTATCTCTTCGTGTAA 0.537790745056

CACTCACAATGTCATAGTAGAC 0.860970329862

CACTCACCAGCTGCCTGGTTAG 0.930986452041

CACTCACCGCTTTGATCGTAAA 0.528590628549

CACTCACGTACTCAAAAGCTAA 0.42840750836

CACTCACTCCGAGATTTGCGAT 0.650828895465

CACTCAGACCCTCCTTAAATAG 0.295735893152

CACTCAGATTGTCGTCGCCCAC 0.681530238676

CACTCAGGAACTCCGGTAACTG 0.363653778588

CACTCAGGTTATCATGGAGCAG 0.383543173307

CACTCAGTACGGGGACCACGAA 0.428882588791

CACTCAGTAGTGGTAGTTGAAA 0.295515551162

CACTCAGTCTGGTTAATGTAAG 0.551373908947

CACTCAGTGTATTATACACCAC 0.902938492139

CACTCAGTTTACGAACAGTGTT 0.465216910802

CACTCATAATATGGAGGCAATG 0.728078219807

CACTCATAATGTCCTTTCCAAT 0.430061083969

CACTCATATACTCCTATCTTTA 0.503882880247

CACTCATCCTGGTAAGCTTGAG 0.935541377659

CACTCATGTCGTGCACCCAATT 1.43264665413

CACTCATTCTAGCTAGCGAGAT 0.457534345427

CACTCATTTTCGCCAGCGAATG 0.535941459056

CACTCCCAAACTCGCTTTTTAT 0.503448576745

CACTCCCAGTCTCATGGAGAAT 0.303008574127

CACTCCCCCACTGCGGGGCCAT 1.46280955364

CACTCCCCTCTTGGGGAAAGAG 0.607294477694

CACTCCCGACCTTATTTCGTTG 0.259571382014

CACTCCCTGTCTTCTGGATATG 0.822900317622

CACTCCGCATCTTGTGGAAGTT 1.00085397848

CACTCCGGCCAAGAGGCTACAT 0.577204168931

CACTCCGTAAGTCATGCGGGAG 0.208878557477

CACTCCGTCGCTCCGTACCAAG 0.654815428497

CACTCCTACGTTCATAGGATAA 0.583144308641

CACTCGGCGTTGGTTGAGCAAA 0.638628253854

CACTCGTAAAATGATGACCAAA 1.27945848846

CACTCTCAACATGAAGGATAAG 0.732160145649

CACTCTCAACTTGAAGCGTGAA 0.481026252466

CACTCTCACCATGTTGCCACAT 0.529044033601

CACTCTCACTGTGCTGCAGTAT 0.782593360549

CACTCTCGATTCCAACGTAGAT 0.563963501197

CACTCTCTAGCTCATTTCCAAG 0.429329097998

CACTCTCTATCTGCTTGCGCTG 0.829975276021

CACTCTCTCTCTGCTCCGGGAT 0.516880048534

CACTCTCTTTCTGCAAAATTAC 0.617572057712

CACTCTGAATATCTATTTTGAG 0.671622625051

CACTCTGACAGTCTGAAAGCAT 0.370466373369

CACTCTGACTTTCCTAAGAGAC 0.184064002757

CACTCTGATACTGCGAGTTAAC 0.743874187246

CACTCTGCCAATAATGACATAT 0.364938841333

CACTCTGCGGGTCCCCTTCAAC 1.21763347758

CACTCTTAGTTGTAAGCGGAAC 0.701966632066

CACTCTTGACCAGCTACGTAAA 0.332355774237

CACTCTTGCGTTGTGGATGAAG 0.127758699531

CACTCTTGCTCTGCTTTAAGAG 0.596232606137

CACTCTTGGTCTGCACTTGGTT 1.58175499407

CACTCTTTGTCTGCTGTATGAC 0.905896541751

CACTGACAAACTGGATTCCGAG 0.82413915792

CACTGACAGTATCATGCTTGAC 0.602841560512

CACTGAGAATATCATCTTATTT 0.557136470754

CACTGAGAATTTCTTGTTACAT 0.54237212848

CACTGAGCCGTCGATTGCTAAA 0.843459454381

CACTGAGGTGCTCCTATTTTTC 1.10375404677

CACTGAGTATGTGCACGTATAT 0.683518820344

CACTGATAAAAACCAGGGAGAG 0.349047734768

CACTGATAATTCGCTTCAAGAA 0.605496196511

CACTGATATTTTGACACTTTAA 0.405378759164

CACTGATCAGCTTTTCACGTAT 0.68549068448

CACTGATGGGGTGGTGAAATAG 0.639628974133

CACTGATTAGGTCATCGAGGAG 1.0967010981

CACTGATTGTCACCTGGAGAAA 0.641887385902

CACTGATTTGGGGCAGAGAAAT 0.257426171647

CACTGCGAATCTGTACTTTTAG 0.624701000987

CACTGCGACTCTGTTGGTTATC 0.409774926807

CACTGCGATGCTGCACCTTAAC 0.317510218302

CACTGCGGCTGTGATGACTCAT 1.36309157697

CACTGCGTATACGACCGGTGAC 0.605141830163

CACTGCGTGACTGATCAGGAAC 1.16851111883

CACTGCTCATCTCCTTGTCAAG 0.453515369946

CACTGGGCACTTTTTAACAAAC 0.884589130422

CACTGGTCGACTGGTAGGTGAT 0.467685480847

CACTGTCAATCTAGTGTTGTAG 0.282124967245

CACTGTCACAGTTGCAATGATG 0.655741035096

CACTGTCAGCCTGAAATCGTAT 0.509834942352

CACTGTCCTACAGATACGTGAA 0.395093505352

CACTGTCGAGCTGCAAGCGTAT 2.5194429097

CACTGTCTAGATGATGTGAGAT 0.404303756843

CACTGTCTATCTGCTTGCTTAC 0.824143551513

CACTGTCTTTCTCATGGCATAA 0.45517773883

CACTGTGCAGCTCCTTTAGCAA 0.215691067235

CACTGTGCATATGATACGTAAG 0.242971731472

CACTGTGGCTGTTAAGTCGGAC 1.08489793602

CACTGTGGTTCATATTATGTAG 1.46723476712

CACTGTGTCAAGGCAAGAGAAA 0.511177646454

CACTGTTAGGGTCATTCATAAA 0.358794240896

CACTTAGAATCCGTATAGGTAC 0.572244273921

CACTTAGGAATTGAATAAGAAT 0.345596823624

CACTTAGTGTGTACCATCGTAA 0.893210912895

CACTTATGATCTCCACGGGTAG 0.504220185852

CACTTCCTATCTGTAAGGGCAA 0.620637937037

CACTTTCAATGTGATATGCCAA 0.325849563797

CACTTTCGACCTCTTTTCGTAC 0.609356835618

CACTTTCTGTTTGTTATACGAA 0.713206513255

CACTTTGGATTTGATCCATGAC 1.26840439504

CACTTTGTATATGCTTCGCGAG 0.180373326623

CACTTTGTCTCTGCATCGACTA 0.408016790459

CACTTTTTATGTCATTCCTCAG 0.499749430894

CAGAAACAATCTGCACGATGAC 0.554379874746

CAGAAATAATTTGATCGAAAAA 0.493695413184

CAGAAATAGTTTGCTCATGTAG 0.528157865101

CAGAAATTAACTAAAGCCTTTA 0.378190243267

CAGAATGTTGCTGATGGGATTG 0.816686739942

CAGACAAAGAGTTGACCTCGAA 0.748703765358

CAGACACAATATGTAAAACGAT 0.484574287

CAGACACATGCTGGTACGAAAG 0.358717061791

CAGACACCCGCGGCATATACTG 0.446627587379

CAGACACCGTCTCCTTTCCAAC 0.453048864153

CAGACACTGAAGGCGAACGTTC 0.408401981536

CAGACAGAATCTCTACACAGAT 0.463554175907

CAGACAGACTCTGTTCAATAAC 0.350877510009

CAGACAGATTTTCCGGCGTTTA 0.911002412419

CAGACAGTATCCGTAGTTAAAT 0.232943512325

CAGACAGTATGTGAAAATCAAT 0.312509626682

CAGACAGTGTGTGCCTCACGAG 0.545354915732

CAGACATAAGCTCCTAGTGCTA 0.214758860992

CAGACATACTGTCCTAACAAAC 0.464088593589

CAGACATACTTACATATAAGTT 0.255042200753

CAGACATCACGTTCACGAAAAG 0.520593285554

CAGACATGCGGTGTTTTCTAAC 0.617523661337

CAGACATTTTGTCTCGCTAGTC 0.910480657533

CAGACCCTATGTCATCTCTAAT 0.105122583815

CAGACGCAAGCTCGTGATTGAG 0.565370622338

CAGACGGCATGTCTCTAGTCAA 0.461737419088

CAGACGGTCTCTGTCGAGAAAC 0.630110190966

CAGACTAAGAGTGTTTGACTAC 0.664130827018

CAGACTCAGACAGTATTGTAAT 0.713105367339

CAGACTCAGGGAGCTGGCTCAA 0.330476047111

CAGACTCCGTGTGCTCCAAGAC 0.742665227452

CAGACTCGTCGTCATATTTGTT 0.126632055319

CAGACTCTATCTCATGATATTA 0.649580846585

CAGACTCTATGAGATTAAGCAC 0.503502681862

CAGACTCTTCATGCACGACAAA 0.252632443217

CAGACTGAAGATCCAGCCCGAA 0.601516191174

CAGACTGAGATTCCCCGCGGAG 1.99493069486

CAGACTGGTTTTTTATAGCAAT 0.632964883938

CAGACTTCATCTGTAGTGAATA 0.357793493518

CAGACTTGCTCTGACTCGTGAA 0.700827222817

CAGACTTTCTCTCGGCGATAAC 0.477753566839

CAGAGAAAACCTTAAGCTTTAC 0.331709699623

CAGAGAAACCGGGTTACCACAC 0.889270548134

CAGAGACAGATTGTTCTATTAA 0.472177950742

CAGAGACAGTTTCGACGACGTC 0.60953369983

CAGAGACGATATTCTGTAGTAT 0.664700113898

CAGAGACTAGGTCCTTCATTAG 0.450415703262

CAGAGAGGCTCTCTCCAGAAAG 0.624156559054

CAGAGAGTAAATCAAAAATTAC 0.328254246135

CAGAGATAACCTCCATGTTATA 0.806625483518

CAGAGATCCTCGCCATTGCCAG 0.841027361908

CAGAGCCAAGATGTAGATTCAG 0.601826261129

CAGAGCCGATGTGAAAATTGAC 0.508581667587

CAGAGCCTGCGTGCACAGAGAC 1.52014719317

CAGAGCGGATCTCCTCGAACAT 0.646545073839

CAGAGGCCGTGTTCTTTACTAT 0.378190950166

CAGAGGGAATCCTATTGGCCTG 0.831884928632

CAGAGGGAATTTGAGACACGAC 1.14545518485

CAGAGGGGCTCAGCTAGATGAA 1.02993042961

CAGAGGGTGAGAGCAGCACTAA 0.454011325204

CAGAGTGAAGCTTCATCATTAC 0.349025271318

CAGAGTGAATCTCAAGCATTAC 0.357903539249

CAGAGTGACTCACCTCCCCAAG 0.731344831258

CAGAGTGTGCCTCCATCTCTAT 0.30374540363

CAGAGTGTGTGTCTACGACAAA 0.612759849478

CAGAGTTAGGCTGCTGCATAAC 0.633289009148

CAGATACAATATCAACCAGTAC 0.209155835891

CAGATAGAATGGTATTTATAAG 0.153233163291

CAGATAGTAACTCTTGACTTAT 0.889927893125

CAGATATAATCCGGTGCCAATT 0.241876072289

CAGATATACAGTTCTCACGTAG 0.354067271043

CAGATCCAGCATGTATATTGAG 0.828932802355

CAGATCGCAGATGGGGGGTGTG 1.82736358574

CAGATGGACTGTCCAAATAGTG 0.628215956406

CAGATTGAGTGCGCTGATTCAG 0.822681861513

CAGATTGGGCTGCCCGAACCAT 1.68607911175

CAGATTGTTTGACCTTTTCAAG 0.235196202686

CAGATTTGTCCTGATGGACGAT 0.46027472863

CAGATTTTCTATTCTGTTTAAC 0.701290286925

CAGCAACAATAAGATTTAAAAC 0.364662856069

CAGCAATGACATGCTCCGTAAC 0.582683858163

CAGCATCGTCGTGATCGGGAAT 1.0742817332

CAGCATGTCAATGCTGAGGCAC 0.911565040714

CAGCATGTTCCTCTATATAGAG 0.266203241446

CAGCCACTATCTGAAAGTGGAA 0.261417988935

CAGCCACTTTTAGTCCGTATAG 0.496027109414

CAGCCAGAATCTGCATGTAGAT 0.755075191796

CAGCCAGACTCTCATACCTAAG 0.733248833096

CAGCCATAACTTGATGGTCGAT 0.356291756172

CAGCCATGCTCTCGAAAGCAAA 0.927908595186

CAGCCATGTTCGGATGCCGGAG 1.03840262116

CAGCCATTTACTGCTCGTCAAC 0.491556655907

CAGCCCCAGATTGGTGAGGGTT 1.20041696047

CAGCCGTGGGCCTACTTGGAAC 2.14088680659

CAGCCTCCAATTCCACGGGCAG 0.861909063914

CAGCCTCTATATCGCTACGCAT 0.620167509921

CAGCCTGAACCATATTATGAAC 0.667395643336

CAGCCTGAATGGGCTGTTAAAG 0.670006912937

CAGCCTGGAAGTTGATCGTCAG 0.133924154707

CAGCCTGTGGTTGCGCTGTAAC 1.1337669797

CAGCCTTCGTCTGGCCGCTAAA 0.807285273848

CAGCCTTGAATTAAATACGGAG 0.22895656798

CAGCGACCCACTCCGGGCTAAC 2.03573314306

CAGCGAGAATTTCTTCTTTTAT 0.555450697984

CAGCGAGCGTGTGCTCCCTATC 0.668517974712

CAGCGAGGCTATGCACTCAAAG 0.502920722417

CAGCGAGTATATGCACCCTTTA 0.521063551441

CAGCGATAACGTTATCAGAAAG 0.207756108363

CAGCGATGTCCGGCATGGAAAT 0.556986973912

CAGCGCGTAGAGCTACGGGTAG 0.623636571815

CAGCGCGTATCGGATTTTACAA 0.417764795005

CAGCGGGAATATGTAAAATATC 0.428766581318

CAGCGGTCAGTTCCATAGGTAC 0.836429176522

CAGCGTCCTTCTGCATTTGTAG 1.04124573408

CAGCGTCTAGCTGGTGTTAAAG 0.689461575198

CAGCGTCTATCTTCTTCCTAAC 0.194598510624

CAGCGTTTCGCTCTTCGGGAAG 0.663986846894

CAGCTAGCCGCTCTTATATAAT 0.466854723988

CAGCTAGTATCAGCAGTGTTAT 0.737387650928

CAGCTCCAAACTCTGGCTCAAT 0.548139986332

CAGCTTCGGGGCGTCGTAAGAA 0.889186440268

CAGGAATAGTCTGAACATGCTC 0.488746354314

CAGGAATTAGATGCCACCCCTC 0.548268287383

CAGGAATTCCGATGATGCAAAG 0.644478676899

CAGGACGTTGATTGAACTAATA 0.624168949489

CAGGATGTCTGAGCTACATATC 0.697249530795

CAGGATTCATCCGCTCAGAAAG 0.702960886243

CAGGCACGAGCTGTTTACTCAC 0.822154671311

CAGGCACTAGGTCTTAACTATC 0.696262369066

CAGGCACTCGATGCTTGGGAAG 0.587011814894

CAGGCAGTCTATCCTGCCTAAG 0.622628493743

CAGGCATGGTCTGAAAGCTTAT 1.89785951069

CAGGCCCAATCGGTTCATATTT 0.727913162572

CAGGCCGACCGTCCTGAGCTTC 2.7532081835

CAGGCCGACCGTGAAACTAGAT 0.323523465655

CAGGCCGACTGTGCGTTGATTG 1.463712552

CAGGCGCGGATTGACAGTTCAG 1.2066859911

CAGGCGGTCCGTTACTGACCAG 1.47714648586

CAGGCTGTATGTTGTGCGCAAA 0.735362463149

CAGGGAACTCCTCGAATCATAA 0.618969382557

CAGGGACCAACTGAGTAGAGAC 1.24119898122

CAGGGAGGATCGGTATGTGCAG 0.907587933722

CAGGGAGTATGTTATGTTCCAC 0.672830238219

CAGGGATGAAATCAAGGCATAC 0.557229687159

CAGGGGCGCGGTCAATGCAAAC 0.164703126891

CAGGGGGGAACAGCTCCACCAC 0.737787769687

CAGGGGGGTTCTCTAATATATC 0.958770405091

CAGGGTAAATCTGTGAGTAAAA 0.436076069832

CAGGGTCACAGTCATGGGTAAA 0.900220600394

CAGGTACCAACTCTAAACGAAC 0.906777902585

CAGGTAGTCACTCCAGGCAAAC 0.400952057551

CAGGTGGGCGGTGCTCTATCAT 0.907979889756

CAGGTTCTGAGTCCTGCGGGAG 1.50004180118

CAGTAACTATGTCGAGCTTCAG 0.806327121767

CAGTAAGAATCTCCAGACTAAT 0.328470371657

CAGTATCATTATGCTGTATCAG 0.667738597442

CAGTATCTATGTGATTTAGGAG 0.527492602556

CAGTCAACCTTTCATTGGTTAA 0.53523476369

CAGTCAATAACATATGCAGAAT 0.229137852313

CAGTCACAATGACCAACAGGAG 0.633160533021

CAGTCACATTAACCAGCTTAAG 0.470407335472

CAGTCACGATTTGATGTCCTTC 0.855181951507

CAGTCACGCTATCTTGTGTGAG 0.696968071765

CAGTCACTGTTATAATTGTTAA 0.520297860488

CAGTCAGAATCGTTTTGGGGAT 0.532417059059

CAGTCAGAGACTGGTCACGAAG 0.480080444662

CAGTCAGAGAGACCGGACGAAG 0.514427731334

CAGTCAGGCTATCATCACACGT 0.795696612701

CAGTCAGTAGCTCGCACCTAAG 1.0588925802

CAGTCAGTATCTGCTTAGTGTG 1.12621584279

CAGTCATAATCTCGATTACAAA 0.382397307418

CAGTCATAGCCCGCTCGTTAAT 1.42626096299

CAGTCATAGTACATTGGAATAA 0.803810070833

CAGTCATATCCTGCACACTTAC 0.722171346946

CAGTCATCTGATCCTTCTGTAA 0.281236202111

CAGTCCCACTGTGAATAGGAAC 0.91562020901

CAGTCCCTGTATGCACTGTCAT 1.40022957107

CAGTCCGACTCTCATATTTGAG 0.385412121492

CAGTCGTAAGCTGAGTGCTCAT 1.01594841657

CAGTCGTCCGCCCATGCCGTAC 2.59138325133

CAGTCTCAGGTTGCACATATAC 0.674916754703

CAGTCTCCCCCTCATGAACGAG 0.283502345471

CAGTCTCTATTTCTTATTCGAC 0.801507824734

CAGTCTGAGTCGCCTAACACAA 0.879696692782

CAGTCTGGAACTCCTTCAAGAT 0.823684380971

CAGTCTGGGTCTTGTTATTGAC 0.514489997712

CAGTCTGGTTCTGAATTAGAAG 0.730531436222

CAGTCTGTACCTGATTATTAAG 0.477801400674

CAGTCTTCTTGTGCGGGCTAAT 1.28321000606

CAGTGACACTCCCAGCCTATAG 0.261404611316

CAGTGACCGGCTGCTCGGTAAA 1.2616247322

CAGTGACTATATTTGGTAAGAG 0.550326957423

CAGTGAGAAGGTCCAGGCTAAG 0.676666061164

CAGTGAGCGGGTGCTAGTAAAG 0.781421285397

CAGTGAGGACGTGCTTGTGAAG 1.12950681037

CAGTGAGTACATCAAATTTATC 0.457973290685

CAGTGAGTGACTCGACACACAT 0.96141552129

CAGTGAGTGTGTTGACGATTAG 0.276881714321

CAGTGATCCTATCTTTTGTGAG 0.442981371463

CAGTGCCCGTCTGAATCATCAC 0.393530814941

CAGTGCCGACGTTCTAGTCTAG 0.340351173001

CAGTGCCTCTCTCCATAAACAT 0.701650133003

CAGTGCTCATTTGCATAGGGAA 0.302673454137

CAGTGGACATGGGAGTTCTAAA 0.703334776933

CAGTGGCGACATGAATGATCTG 0.290588728499

CAGTGGTTTACTGCGGGTCAAC 0.877689930145

CAGTGTAGACGTCGTAATAATC 0.362679548868

CAGTGTCATCCGGATGTCGTAC 0.899515903079

CAGTGTCCTTTTGGGAGGGGAT 1.17512514383

CAGTGTCGGCCTCCTAAGAAAA 0.481951730188

CAGTGTCTCTGGGATATGTAAT 1.02879395946

CAGTGTCTCTGTGATGCCGTAG 0.937571417441

CAGTGTGGCAGTCCCTGCTGTT 1.35415383034

CAGTGTGTAGGTGCTAATCAAG 0.490936350418

CAGTGTGTATCTGCGATAGTAG 0.419664475613

CAGTGTTACAGTCGTGCCCCAG 0.502982711327

CAGTGTTAGCCGCAACCTGAAG 0.704932753209

CAGTTAGACTCCTCATGAAGAA 0.583773535617

CAGTTAGACTGTGCGGCCGCTT 1.32836519724

CAGTTAGGGTCTGCTTAGATAC 0.734601603495

CAGTTAGTATCTGGTGTGTGAG 0.769104073581

CAGTTATAATGTGGTCGGTAAC 0.752905505457

CAGTTCATCAGTCATGCATAAA 0.446835635495

CAGTTCGAGTGTCTTAACGTAA 0.518359602106

CAGTTCGGATTGTAAAAGATTA 0.434293783606

CAGTTCTAATGTCCCTACGTAC 0.514709314295

CAGTTTCAGCCTACCCCGGAAC 0.241126237572

CAGTTTGATTGTGATGGTGAAA 0.794038946865

CATAAACTCGCTCTGGCAGAAT 0.354257923544

CATAACTCATATGCAGGGGCAG 0.450891614606

CATAACTGGCGTCCCATCGAAG 1.66664039448

CATAATCGTTCTCATAATTAAT 0.416795738081

CATAATGCGCCTCAACATGTAG 0.216234949145

CATACACAAAGTGCTTGGGAAC 0.468268458867

CATACAGACTCTCTATAGTGTG 0.703180579652

CATACAGATGGTTGGTGGCTAA 0.769469136474

CATACAGGCGCTGATTTAGATT 0.528117106092

CATACAGGGTCTCATTTCAAAG 0.539067192547

CATACATGACGGGCATATTAAC 0.398341913123

CATACATGCTTTCGTTTCGTAC 0.354936514387

CATACCATCGTGGCTGGCTAAT 0.547289549463

CATACCCCCTGTCCTCGCTTAG 0.847737182354

CATACCGGTGCTCTGCGACGAT 0.64784554741

CATACGCACTCAGAAGATCATG 0.440733500589

CATACGCCACCGCAACCGGCAC 0.850267904903

CATACGGAGAAGGCTAATCTAT 0.385900118331

CATACTCACTGTCATGCCGTAC 0.379945770582

CATACTCCATATGCTGTCTGAG 0.42121087264

CATACTCTGCCTCCTGGGTCTG 0.723020261214

CATACTGCAAGCCCTAGTTAAT 0.445147604042

CATACTTGATCTCCAGCTCCAT 1.04929796266

CATACTTGATGTGAGGGCTAAC 0.528792531452

CATACTTGCCGTCACCGAACAT 0.568473786075

CATACTTTACTTGTAGCAGTAC 0.720726291211

CATACTTTATCTCAGGCTAGAA 0.641181311854

CATACTTTATGTCCTGCCGTTT 0.915092489812

CATAGACAAATAGCGTCAATTG 0.443652556568

CATAGAGGGTGTGTAAATAGAT 0.750659313656

CATAGAGTGACTCCTAGAATAG 0.717674772755

CATAGCCTAGTCCAGTATAGAC 0.423479368548

CATAGGTGCTGGGATGCGAAAC 1.08526052408

CATAGTAACTCTCTTAGTTTAC 0.379787512221

CATAGTCGAACACCAGGTGGAG 1.80459266785

CATAGTCGTTGCCGCCTAAATG 0.86402970522

CATAGTGATTCTGAATTCCAAC 0.517759286503

CATAGTGCATCTCGTAACCAAG 0.534090075345

CATAGTGGTGTTCAAGAACAAT 0.300850875925

CATAGTGTGTGACCGCGAGATT 0.88511539565

CATAGTTAACGTCTACTTCATA 0.416825737435

CATATAAACTCTGTTATCTATG 0.373085414643

CATATACGCACGGCATGCTGAC 0.771867494054

CATATACTTCCGCCACACCGAT 1.19416062277

CATATAGCCCTTGTTGATTAAT 0.806216836164

CATATAGTCAGTCATCATCATT 0.205082301445

CATATCTCATCTGGTAAACAAG 0.478221239753

CATATGCACTCTCAACACAGAT 0.962030858371

CATATTCAAACTGTGGCAAAAG 0.601246174984

CATATTCGCGCTGGGAGCTTTA 2.1097635314

CATATTCTCTCTCATCATGTAG 0.435159134947

CATATTGCCCGTCATGTGCCTT 0.703644389612

CATCACGTCTCTCCTACCGAAG 0.357731930698

CATCATGTAAATTGTGCAGAAC 0.598636000706

CATCCACAGCCTGATTCAGATG 0.276325716298

CATCCACTACGTGACAGACCAT 0.471699634415

CATCCAGAAAATGGTCTGAAAT 0.331305393554

CATCCAGTCCCTCCAAATAAAT 0.307122823278

CATCCATAAGGGCGAGTGGCTC 1.04390696383

CATCCATACTGGGTTAAACAAC 0.531715013461

CATCCATGTGGGCAATAAATAT 0.3750162783

CATCCTCAAGCTGCTATTAAAC 0.472167033572

CATCCTCTCCTGGATACCGGAA 0.73345362161

CATCCTGAGTATTGAGACTAAA 0.797001383623

CATCCTGCATCAGAATACAGAC 0.792380167901

CATCCTGCGTGTGATGCAATTG 0.488364752012

CATCCTTAAATGGCTTCTTAAA 0.20953699462

CATCGACAATGCGTCGAAGAAC 0.365545994617

CATCGACACTTTCAACGCGAAC 0.448070438075

CATCGAGCAGGAGCAGTTCAAA 0.522154356563

CATCGAGGATGCGCTACCTGAA 0.752878478206

CATCGATTGAGTGATACACAAG 0.261891019794

CATCGCTTAACGCCTATAGCAC 0.262886768168

CATCGCTTCTCGGATAGCGAAG 1.46418278108

CATCGTTCAATTTCCCGGCTTT 0.849038855652

CATCGTTTACCTGCACCTAAAA 0.707201238381

CATCTGAGGTGTGATCAAAAAT 0.684161282733

CATCTTCACGGTCAGTTAGTAG 0.421446543828

CATCTTCTAACTCATGCGGGAG 0.697545375824

CATGCACAATGTCATATGAGTC 0.491065272615

CATGCAGAATATGCTTGTTAAA 0.345292293882

CATGCTCTATCTGTATGCGCAG 0.428706349312

CATGCTCTTGGTGCTGAGGGAC 1.36560411001

CATGCTCTTTGCCTTTGTTAAA 0.467291593341

CATGCTGAGTATGAGATTGGAA 0.529673847618

CATGGAGACTGTCAAAGGATAT 0.335513257037

CATGGCGAACCTCATTCCTGAT 0.634501288863

CATGGCTAACCTTCGGTATAAG 0.641156512454

CATGGTCCAAGTGTTAGTATTA 0.568121363678

CATGGTGAGGGTCGAAAAACAA 0.522777313278

CATGGTGGCTTTTATGTGGGAT 0.772946537391

CATGGTGTAACACAAACATATA 0.385986697319

CATGGTTAAGGTCAAACTCAAT 0.236971323414

CATTAAGAGTCTGGAATTACTA 0.737086887825

CATTATGAACGTCCTTAGCTAG 0.372543674383

CATTATTACACACATTAAGGAC 0.465224063246

CATTCACTATCTCAATGAAAAC 0.397566067016

CATTCAGAATCTGACGCCACTC 0.804083612693

CATTCAGAATCTGATCGGTAAA 0.37583731497

CATTCAGAGAGTAATCCGAAAA 0.449859421608

CATTCAGAGGTTGCAAATGTAG 0.505875817683

CATTCAGCGTGTGATTGGCAAG 1.55350369635

CATTCCGAGTTTCAAACTTAAG 0.309405620295

CATTCGGCGACTGCCAGGGTAA 1.33556414906

CATTCTGGAACTGAGCTTGAAG 1.93071895818

CATTCTTAATATCCTCATCGAG 0.569324154974

CATTCTTAATCTCGTTACTAAC 0.813730871385

CATTCTTGTGCCGGTGATAAAA 0.369278102944

CATTGACAGTTTCCTGCTAAAG 0.501536958526

CATTGACTAGCTCTAACGGAAC 0.413052194578

CATTGACTCTGTGGTTCGGAAG 1.09711387499

CATTGAGAAAGTCATGGTTCAA 0.30295598206

CATTGAGAAATTGGTGAATATA 0.45160051367

CATTGAGCCGGTCATGTAGCAC 1.22647310335

CATTGAGGAGCTCCTCTCTTTG 1.00206754403

CATTGAGGTTCTCATGCCGTAG 0.542804274055

CATTGAGTCCCTGCAGCGTATT 0.820414874899

CATTGAGTCTTTGCATGATCAT 0.558450196718

CATTGATACCCTGCGCGTGGTA 0.428205060159

CATTGCCAGTCTTGAGTAATAG 0.431688092075

CATTGCTAGAGGGATACCAAAG 0.411149740174

CATTGCTTAACTCTATTATTAG 0.774487163184

CATTGCTTCTGTTCTGGTGTAG 0.363943412794

CATTGTGAATCTTACACTGAAG 0.7407518347

CATTGTGCGGCTGAATAACTAC 0.781538051059

CATTGTGGAACTGAGGGGGTAC 1.7269710962

CATTGTGTCTCTCTTTAGGGAC 1.18110411191

CATTGTGTTTCAGTATTTCAAC 0.568410191323

CATTGTTAGAGTGAAGCTTAAA 0.453249646669

CATTGTTTATATGCAATACTAG 0.826241146179

CATTTACTGGCTGGTCAACATA 0.916006985324

CATTTAGAAGCTGCTAGCAAAG 0.618591596321

CATTTAGTTTGTGCAAACTGTG 0.337868492924

CATTTTATAGTGCCGTTCCAAA 0.560235229418

CCAAAGAAAGCTCCGCCCTAAC 0.505058727489

CCAACACGTTCTGCTTTGCTAC 1.14984053456

CCAACAGACTCTCCGTTGACAA 0.783146262852

CCAACTTTATCTCTACGGCGAG 0.580413488212

CCAAGAGACACTGATTAATAAG 0.235466103202

CCAAGAGTGCCTCAAAGGCTTA 1.06323551351

CCAATTAAAGCTCAGCCGCCTG 0.959406727478

CCAATTCAATTCTCACGCTCAC 0.18875751748

CCACAACTGGCTGTAGTTAGTT 1.50079451645

CCACTTCGTCGTGCTGGGATAT 0.595067742662

CCAGCTTTATTTGCCCAATCTG 0.710094636547

CCAGGATGACCTAGTTCTTCTT 0.98933339457

CCATATCTACCTGCTATGTCAC 0.561410788006

CCATCCGTGACTGCTAATCAAA 0.64008989867

CCATCTACCTGAGATCACAAAT 0.72319017446

CCATGTCTCTCTCCAGCCCAAG 0.387626765858

CCATGTGTATGTGCAAATTGAG 0.972182589615

CCATTATCACCTGATTAAAAAC 0.218940063744

CCCAAACGCCATGGTGAGTCAG 0.496699782985

CCCAAACTATCTGGTGCCAGAT 0.737635673604

CCCAAAGACTCTGTAATGTGAG 0.617145042558

CCCAAAGGCTCTCCAACAAGAC 0.694569398077

CCCAAATAGTGTCAAATCTATG 0.434036548916

CCCAACGAACTTGAAATCTGAC 0.563687279798

CCCAATCAACGTTGTGCAAGTT 0.580716957499

CCCAATCAATGTGCTTGGCTAC 0.503724010722

CCCAATCCATGTACTACTGAAT 0.474040047171

CCCAATGATCGTTTAGACAGAT 0.254547203135

CCCAATGCTTGTTCAACAGGAC 0.55163120356

CCCAATGTATCTGAAAATAAAG 0.398180956967

CCCAATGTCTCTACAAGAAAAT 0.365434149741

CCCAATTAATCTGATGGACTAC 0.709828752389

CCCACACACTCTCCGCGTCTTA 0.635458515896

CCCACACACTGTGTACAAGTAT 0.424018750217

CCCACAGAAAGTCGTGTCCCAT 0.960188229876

CCCACAGAGTCTGCTCCTGAAA 0.700639723669

CCCACAGCATATGGATGTCCAT 0.848140087623

CCCACAGCATCTGAGTCTGATC 1.04305363152

CCCACAGTATGTGCTTGCTCTT 1.79317215913

CCCACAGTCATTGGTGCACAAT 1.47172513957

CCCACATGCTTTGTTTGAGAAC 0.164293581051

CCCACATGGTGTACACTGGCTT 0.263292415067

CCCACATTATTTCTTCAGGTAA 0.884611894624

CCCACATTCTTTCAAAGTAAAA 0.277761340727

CCCACATTTCGCGTGTCAGGAG 0.503111930408

CCCACCCTAGCTCCATTAAAAC 0.379021147692

CCCACCGAAGCTGGGAGCTATG 1.25525942414

CCCACCTTAAGTCATAGCAAAG 0.714920237952

CCCACCTTCTCAGATGCCGGAG 0.396843260808

CCCACGCAAATTCATGAGCTAA 0.618559225631

CCCACGCCGTCCGTTTTAAGAC 0.486349085713

CCCACGCTAGCAGACCACCGAA 0.613064313669

CCCACGGATTGTCATCCGCGAG 1.04307603012

CCCACGTGACCTGATTCAAGTC 0.809486933587

CCCACTCAATCTCATGCGCAAG 0.817393154877

CCCACTCACGTTCCACGTAAAA 0.549200018312

CCCACTCAGTGGGGCACAAAAT 0.710756727966

CCCACTCGAGCTCCTGTCCTAA 0.590560489804

CCCACTCGCACTCTAGCATAAC 0.486233960735

CCCACTCGCTGTGCTCAATGAA 0.199415779177

CCCACTCTGCGCGAAACAGAAG 2.03779935965

CCCACTGAATTTTTTATGGTAG 0.595577416852

CCCACTGACAGTCACTATAATT 0.769839923067

CCCACTGCGTCTCCTAGGAAAG 0.926086212332

CCCACTGGCTGTCATTTGCAAC 1.54730482014

CCCACTGTATATGCTTTCCAAA 0.744513511105

CCCACTTACGCCCTAGTATGAG 0.741053050229

CCCACTTCGTCTCCTAGCACAG 0.837920914068

CCCACTTTATTTGCGGGGCGAG 0.959882701587

CCCACTTTGCGTTAGCTACGAC 0.739185669332

CCCAGAAACTGCGCGAGACTAC 1.10217205567

CCCAGACACTCTGATATCCAAT 0.655994851101

CCCAGAGACTGTGAAATGGAAA 0.560730896216

CCCAGAGAGTTACCTTCTAAAG 1.27298944071

CCCAGAGGCGATGGCTCTTAAG 1.17052634931

CCCAGAGTAGGTGCTGCGCAAT 0.158278636126

CCCAGATAAGGTCTTACGCTAT 0.773219007527

CCCAGATATTGTCCTAAAAAAA 0.571116270527

CCCAGATCCACTCTGGTTGGAC 0.307727991492

CCCAGATGGCTTCAAAAATCAG 1.15347763752

CCCAGCCTACCGGCCTATTAAG 0.390916761438

CCCAGCCTCTGTGAAGACTATG 0.215749231719

CCCAGCCTTTCTCCTAAAAGTC 0.565070778967

CCCAGCGAAACTGCTGTTAAAT 0.467809349668

CCCAGCGCGAGTGATCATTGAA 0.749916867907

CCCAGCTCCACTCCTATCATAC 1.17838225189

CCCAGCTTCTCTGAAATGCAAC 0.253846043664

CCCAGGAAATCTGATATAATTT 0.31440250915

CCCAGGAGCAGTGATTACATTC 0.695194392814

CCCAGGGTGTGTGTTGAATATG 0.766117299839

CCCAGGTCCACTCAGTGGTTAC 0.90720693544

CCCAGGTTAACTGAAACTCCTG 0.998449619565

CCCAGTCAACGTGATAGGGAAG 0.773924822848

CCCAGTCACTGTCCAGCCTTAG 2.40676966158

CCCAGTCCGTCTCTTTACTTAC 0.635602598374

CCCAGTGAACCGTAGCGTTCAG 0.317307673993

CCCAGTGACTTTCATGAGCCAG 0.651584572478

CCCAGTGGTCCAGCTCATGTAT 0.939247633624

CCCAGTGGTGCTGAAGAGGGAA 1.15536302445

CCCAGTGTATCTCCTGGGCAAA 0.208947536956

CCCAGTTAAACACAAGGATAAA 0.800424540185

CCCAGTTAATGTCATCACCCAA 0.57308915779

CCCAGTTACGCTGAACACCCAC 1.46257418918

CCCAGTTCCGCTATTCCACGAG 0.716978427586

CCCAGTTTATGTGATAGCGTAG 0.392435138603

CCCAGTTTCGATCCGAGTCAAC 0.584487333241

CCCATATAGTATCCCGGTTGAA 0.705335774915

CCCATATATGCTCAGTCATAAC 0.134322328912

CCCATCCGTTCTCCAGAAAAAG 0.531393603635

CCCATGGTATGTGATTAACAAG 0.329104252605

CCCATTGACACTCATGTTCGTA 0.416468983507

CCCATTGTAGGTGGTCCTCAAA 1.01564166966

CCCATTTACTCTGCCCGATTAG 1.17023490744

CCCATTTTCACGCCTATCTGTC 0.815078789042

CCCCAACATTCTGCTTCTACTT 0.535685498495

CCCCAACCGTCTCAACTAGTAG 0.605364323765

CCCCAAGTAGAGATGGTATGAG 0.447532563604

CCCCAGGAAATTCTTTCCTGAG 0.758260027728

CCCCATGCATCTGATCACCTAG 0.815414097935

CCCCCACCCTCTCGGGGAGGAG 1.72067970714

CCCCCACCTAGTCAATGGCCAG 0.898241873763

CCCCCAGAATCTGAAACGTCAC 0.693508527336

CCCCCAGAGGTTGCCGCCGAAC 0.722136064222

CCCCCAGAGTGTCCTGCATTAT 1.16758369728

CCCCCAGCCTAAGCTATCAAAG 0.304176432639

CCCCCAGCGTCTTCTACACAAA 1.17908298269

CCCCCAGGATCTGATCGATCAT 0.544021650167

CCCCCAGTCACTGGTACGAAAA 0.910597905374

CCCCCAGTGTCTGCTATCGGAT 1.39301278969

CCCCCAGTGTGAGATTAGACAC 0.475868615907

CCCCCATCCTATCCTCTGTGAG 0.851106424627

CCCCCATGTAGTCATAAGTTAT 0.396703303931

CCCCCCCTATACCAAATCGAAG 0.558164732133

CCCCCCGCCTGTGGTGGTAATT 1.28661255492

CCCCCCTAATATCCAGTGGTAA 0.985367054814

CCCCCGAGGCGTCAAGTACTAG 0.955711228506

CCCCCTCCTAGTCAAAGATTAA 0.366686491657

CCCCCTCGATCTGACTGTCAAC 0.675377618733

CCCCCTCTTTATTTCAAACTAT 0.468067950368

CCCCCTGTATATTTCGGAAAAC 1.21412223411

CCCCCTTAATTTGAAAGCATAC 0.385211874335

CCCCCTTGCGTTCCTGCTCCTT 1.17352021016

CCCCCTTTATGGGATTATGATC 0.302377145892

CCCCGAACCCTTGCTGAAATTT 0.460560759864

CCCCGACGCGGTCCTGCATGTG 0.804320059325

CCCCGACTAATTCCAAAACTAT 0.50799647864

CCCCGAGAAAATGTTGAATTAC 0.218203219074

CCCCGAGAATGGGAGTCTTTAT 0.734989460006

CCCCGCGTATCACCTAAGGGAG 1.04113926165

CCCCGCTTCGGTGGTAATCAAT 1.5925861451

CCCCGGCAATGGCATTCCAAAA 0.236497225313

CCCCGGCAATGTGTTCTGCGAC 1.23657657436

CCCCGTCTATGTGCAGACACAG 1.48658785326

CCCCGTCTGCGTGGTCGGGTAC 0.448560927601

CCCCGTGTACCCGGTACCAAAT 1.00581245269

CCCCGTTTGACCGAAGGTTTAA 0.261732744162

CCCCTATCGCCTGATGAAAGAG 0.325674063086

CCCCTCCATGCTGAACGAATAC 0.226153257627

CCCCTCTTATGTTTCTGCATAG 1.26621333091

CCCCTGTAGTATCATTGTACTG 1.46507787751

CCCCTTCAAACTCCTACATAAG 1.1271182979

CCCCTTCTCTCATAAACTGTTC 0.773551544206

CCCGAACAATCTGCTACGCATG 0.234224248437

CCCGATCGATTTGTTGTACAAG 0.874593951512

CCCGATCTAGGTCGGTCGTTAT 0.665183192557

CCCGATTTTAGTCATGAACGAT 0.64064275826

CCCGCAGTATGTCACGGTCAAG 1.25033982939

CCCGCTCAAAATGGGCGAAAAA 0.576590875424

CCCGCTCGCTGCGATTCGAAAT 2.92417772067

CCCGCTCTGTGACATAAGATAT 1.87649716904

CCCGCTGTTGCTCTTACACTAT 0.824245339844

CCCGCTTAGTCTCGGTATGTAA 1.11167381524

CCCGGAACCTCTCAGGCAGTAC 0.793449399939

CCCGGACAAAGTGAATGTAAAA 0.384733813298

CCCGGACTGTCTGTAGTGAGTA 0.566767901189

CCCGGAGTGTCTCATGAGTTAC 0.858592668521

CCCGGATAATTTGCTTGACCAA 0.554246621594

CCCGGATGCGCCGATGCCTGTC 2.14545408521

CCCGGATGCTCGGATTCATAAC 1.10887665151

CCCGGGATATATCCAAGGAATA 0.542831480016

CCCGGTCAAGGTCCTTCACGAC 2.84629355212

CCCGGTCAGTCACTTTAGAAAA 0.623262802212

CCCGGTTCGTCTGAAAACCTAA 1.60776630748

CCCGGTTTGGTTGAATACTAAG 1.81609119527

CCCGGTTTTCCTCCTTATCAAA 0.778763487647

CCCGTTCGATCCCCCTCATAAA 0.478199755506

CCCGTTGACGGTCTATTTAATG 0.868586648973

CCCGTTGGTTCTGATGAGTTAG 2.09726638869

CCCGTTTCCCGTCATGTTAAAC 0.727996780941

CCCTAAACTTATGAACTGTAAA 0.331377310691

CCCTAACCAGCTGCAAGTGTAG 2.29218245894

CCCTAACCCGGTCATGCCTCAT 1.02027442506

CCCTAACTGTTTCAGTGTAAAA 0.466624605559

CCCTACCTAGGAGATTTAGAAG 0.543546576263

CCCTAGCACCCTGTCCATGCAA 0.44168782272

CCCTAGTGGTATGTTCACGTAG 1.00485990388

CCCTATAACCATCTAAAAAGAG 0.26795349405

CCCTATGACTTAGCGAGTCCAG 1.32608534463

CCCTATTTAGCTCATTGTTAAC 0.579632548378

CCCTCAATCCGTGGTAGGCGAA 0.547660012887

CCCTCACAGTATCGTTGGGTTG 0.459895809754

CCCTCACCAACTCGTTCGGTTA 0.459664048253

CCCTCACCGCTTGGAGGATAAA 0.79822628459

CCCTCACTAATTCATCGGATTC 0.294934253257

CCCTCACTGGACCCTGGACAAT 0.917936701046

CCCTCAGAATCGCCATATTGAA 0.723763018986

CCCTCAGACCATGGTTAAAGAG 0.487345352394

CCCTCAGACTCTTGATAGAAAA 0.668283349765

CCCTCAGCACGTCAATACGAAC 0.782676480166

CCCTCAGGGCATCCACGGATAT 0.973532789424

CCCTCAGTAGCTGCTTTGGATT 0.646370865584

CCCTCAGTAGGGTCTTGTCAAG 1.03346294653

CCCTCAGTATATTGCAGTTAAT 0.513213512979

CCCTCAGTCACTGTTTCCCAAG 1.10944500822

CCCTCAGTCCGTGAAGTTCGAT 0.531058124015

CCCTCAGTTTGTGTTGGGGATA 0.358591257256

CCCTCATAATCGGAACTAAAAA 0.259644651203

CCCTCATACTCTGTTTCTGCAT 0.452001933238

CCCTCATAGGGTGATGGCGTAA 0.863963708587

CCCTCATAGTTGGAAGAGATAC 0.283773887022

CCCTCATCATCTGCACGCTAAG 0.721946294002

CCCTCATGCTGAGCTCTTGAAC 1.04289927469

CCCTCATGTTGTCCGCGCGAAC 2.06001058073

CCCTCATTAAGTGGTCGGCAAA 1.00370183829

CCCTCCCCTGATCGTATTTATC 0.572497381135

CCCTCCGACCATCAAGGTTTAG 1.18202501523

CCCTCCGACCATTTTTATCAAA 0.233022461792

CCCTCGCATTGTCTTCATCGAG 0.474615879639

CCCTCGCGACCTCCTACACTAG 0.909837011816

CCCTCGCGGCTTCAAGACGAAC 0.713467514675

CCCTCGGAGGCTGATTAATGAG 0.784279937828

CCCTCGGCGTGTGCTACCTGAG 1.77040590676

CCCTCGGTATGTCTTAACTTAC 1.07332168165

CCCTCGGTCCATCGTTACACAT 0.788993379355

CCCTCGGTGGCTGCTGGCGCAA 0.308277828543

CCCTCGGTGTCTCTTCACAGTG 0.742361872568

CCCTCTAAACCTCATATTCTAA 0.712210928222

CCCTCTAGCTCTCCTTCGCGAT 1.13956933839

CCCTCTCAAAGTCGTTCCAGAG 0.814789650465

CCCTCTCAAAGTGACCCGTGAT 1.0500776129

CCCTCTCCATCATATTGAAGAG 0.629809192523

CCCTCTGCATGGCAAACATTAG 0.36203681285

CCCTCTGCATTTCAGTAACTTC 0.182611358841

CCCTCTGCCTATCGTAAATCAG 0.343395172564

CCCTCTGCGGGTGCAACCCAAA 0.270555386959

CCCTCTGCGTCTGCAGGCCCTT 0.481219118056

CCCTCTGTTCTTGATCTGCAAA 0.47379638955

CCCTCTTAGTCTGATGCCCGAC 0.956039163437

CCCTCTTATGCTCAAATTTTAC 0.575334827973

CCCTCTTCGTTTGATACAGAAG 0.639224445878

CCCTGACAGTCTGAAATGAATG 0.299537852459

CCCTGACGACAGGCTTCGTGAC 0.956130760889

CCCTGACTCAGTCATGGTTAAA 0.695978583442

CCCTGACTGACTGCCAATATAA 0.911133186074

CCCTGAGCCACTCTAACTTAAG 1.05965607309

CCCTGAGCCTTTCTACTCTTAC 0.226495066499

CCCTGAGCGTATTTTAACAAAA 0.32511023902

CCCTGAGGAGCTGGGGGCTAAT 1.4635784848

CCCTGAGTATATGCTAACCAAG 0.743539890789

CCCTGAGTATCTGCACAGTCAC 0.310040455444

CCCTGATGGAATCCTAATGGAC 0.591401076312

CCCTGATTATCTCCTCGCTGAG 0.645776073674

CCCTGATTCTATGGAGATATAT 0.745125241902

CCCTGATTGGATCCTGGTTGTC 1.27824778659

CCCTGCGACTCTCCATTGAAAA 0.575405949675

CCCTGGCAATGACTGTGTCATA 0.688837794298

CCCTGGGAACGCGATTATTAAC 0.815787858687

CCCTGGGGATGGGCAATATGTG 1.16272727212

CCCTGGGGCTATGTTCTCTAAC 1.63121165611

CCCTGGGTACATCCGGCATCAG 2.37257159447

CCCTGGTAATCAGATAGCATAA 0.562572396971

CCCTGGTGAGGTCCTTTGTTAT 0.743292371576

CCCTGTCAAGATGGTATAGAAG 0.326191856506

CCCTGTCAATGTGCTATTCTAC 0.491865221232

CCCTGTCAGTCTTCTTCCAGAA 0.653956294538

CCCTGTGACGCTCAAGTGGCAG 0.696314688851

CCCTGTGATTGTCCAACGCCAT 0.696071342106

CCCTGTGTGAATCAGAAGAAAT 0.280118087488

CCCTGTGTGCCTCCCAAGTCAG 0.330869328832

CCCTGTTAAACTCCAAGAATAA 0.35252363063

CCCTGTTAAGGTGGGTTACTTC 0.701155450182

CCCTGTTTATCACCATCTCTTC 0.641400438822

CCCTGTTTCTGTGTAACTCGAA 1.44339147637

CCCTTAGAAGGTTCCGTAAGAC 1.14454765723

CCCTTAGCGATTCTTTCATCAG 0.316047740091

CCCTTAGTATCAGACGCAATAA 0.62673650594

CCCTTATGATATCGGTGGGAAG 0.488652332802

CCCTTCGGACGCGTTTTGGTAC 0.959070410094

CCCTTTCTAGGTCCTCAAGGAG 1.21597901867

CCCTTTCTGAGTCATACGTTAC 0.891739059737

CCCTTTTAACTAGCTTGCTTAG 0.602853420559

CCCTTTTACTGTGAATATATAG 0.505714338909

CCCTTTTCGGATGATGTGAATC 0.287209488865

CCGAAATATTGTCGATATATAT 0.270524879742

CCGAAATCATTTCACAAAGAAG 0.193143810125

CCGAAATGACATGATTCCAAAA 0.324204239093

CCGAACCACTCTGTTCCAATAT 0.381363727356

CCGAATGACTATCTTAATCAAT 0.229972372842

CCGACAAGCACAGTTGTGTAAG 1.27159930598

CCGACACAATGTGCTCACAAAA 0.542326109523

CCGACACACTGTGTTTGGATAC 0.726919653711

CCGACACCAACTAACTTCCTAG 0.552532459394

CCGACACTATTGCATCCGCGAG 0.773179490357

CCGACAGAACTTTATGGGCAAG 0.891797663871

CCGACAGTCTGGGATGGTGGAT 1.77275669666

CCGACAGTGCGTGCATGACTAA 1.39302728756

CCGACATAATCTGCTTTCTGTG 0.441584730805

CCGACATCCCATCAGTGAAGAT 0.825424108351

CCGACATGATCTGGCTTATCAG 0.649036598902

CCGACATGGTTCGCTGTAGAAA 0.732913815694

CCGACATTACCTCCAGATACTG 0.47179746091

CCGACATTATCTCTAAAAAAAA 0.518818085463

CCGACCCTCTTTCCGCAGCAAT 0.90766986101

CCGACTCCACGTGGTAATATTC 0.325019211919

CCGACTCGGTTTGAAGTAAGAC 0.158306512813

CCGACTCTGCTTTCTGGATTAC 0.818568504281

CCGACTGAAAATGAAGATCTAG 0.333878517574

CCGACTGATCGACACTGCCGAG 0.895830523583

CCGACTTAAGTTCAATAAATAC 0.367111766256

CCGACTTACTCTCTTTCACAAT 1.08684115493

CCGACTTAGCCTGATTGCTAAC 0.686893319481

CCGAGACAAGCTGATATTGATC 0.491760086638

CCGAGACCCGGGGATATAGCAG 0.633401658092

CCGAGACCCTTTGTTAGCTTAG 0.857809193472

CCGAGAGAGCCTCTTTAGAGAT 0.241527608195

CCGAGAGTAACTCTATATTGTT 0.379017351399

CCGAGAGTACCTGCTGTAAGTC 0.846297200907

CCGAGATCAATGCGTCGATGAG 0.239699081049

CCGAGATCATTTGGATAATTAA 0.281307602306

CCGAGATTACTTGATCAGTCAG 0.872602879243

CCGAGATTCGGTGCTGGCATTT 1.01583556873

CCGAGCGAAAGAGCAACACGAA 0.332656007288

CCGAGCTAATGCCTTCATATAC 0.682744700941

CCGAGGCAAGGTCCTATAAGAT 0.644272371514

CCGAGGCAATATCATGTTTAAA 0.336488728103

CCGAGGTACGGTGAAATGTATG 0.485067414846

CCGAGGTCCGGTTCGAGTTAAT 1.23521560283

CCGAGTATCGCTCGTGACGATG 1.363064262

CCGAGTATGGCTGAGAGAGGAG 0.925252636194

CCGAGTCAGAATCCTTACGAAC 0.619857384697

CCGAGTCTACGTGCTCTGTATC 0.693918655766

CCGAGTGAACCTGAACAGTCAT 0.650828633011

CCGAGTTAGTCTCCTCTAGAAC 0.141253121136

CCGATACATTTTCAGTAAGAAG 0.632016585531

CCGATACGATCAGCTAGACGAT 1.21062625067

CCGATTGCAACTGATGAGGCAC 1.14674287269

CCGATTGTGTCTCCAGCACAAG 0.398504405038

CCGATTTCGCCTGCTGGTTGAA 0.198360369576

CCGCAAAAGGCTGCCGTTAATA 1.46121412516

CCGCAATCACTTGCGTGCCAAG 0.542105887669

CCGCAGGAGGTTCCTGATGTAC 0.739418197343

CCGCATAAATTTGCATTCCAAT 0.428669931929

CCGCATGTGTGTGCTCATCGAG 0.973296891463

CCGCCAAGGTATCATGGAAAAT 0.474418205508

CCGCCACCAACTCATCCATCAT 0.345190504163

CCGCCACGGCTGCCAAGACAAG 1.99255153578

CCGCCACTATTCCCAGACTAAT 0.755791271054

CCGCCAGGAGGTCCTCACGTAA 1.00195303311

CCGCCAGTATGTGCACGTTAAG 0.617142120772

CCGCCATGATCTCATGGAGAAC 0.717225833682

CCGCCATGCGCAGCAAATAGAC 1.87556966265

CCGCCCCCATGCGATGAAACAG 2.75464420343

CCGCCGCGGGTTCCTACATGTT 1.21209829356

CCGCCGTTCTGTCCTTTAAAAG 0.360957271034

CCGCCTCAATGTGGTAGACGAG 0.976199136575

CCGCCTCACTATCTAGACAAAG 0.470697221349

CCGCCTCATACTCGAAACGAAA 0.728323501706

CCGCCTTGTACTCATCGGTAAA 0.731654786822

CCGCCTTTAAGTGTTCCGCAAA 0.548089362828

CCGCGACAATGTCCTTGGTATA 0.411268594474

CCGCGACTAGGTCCTGAAGTAC 0.765209830822

CCGCGAGACCGTTAGTAATATA 0.600334773622

CCGCGAGAGTCTCATCATCCAA 0.455040167102

CCGCGAGGGGGTCTACCGTAAT 0.751723415429

CCGCGAGTAACTTAATCCTGAC 0.563399650709

CCGCGATCGTGTGCAAACTGAC 1.26303653827

CCGCGCGATTTTGAATTAATTT 0.646361204121

CCGCGCTAACATCATTAAGTAC 0.738505892244

CCGCGGGGGTGTCTTTCTCTTG 0.946704969313

CCGCGGTTAACTGGTGCACATA 1.47948334688

CCGCGTTTAACTGTTCGGTGAC 0.6360882916

CCGCGTTTAGGCGATAATTGAT 0.182615028615

CCGGAACAGCAACGTGCGGATG 0.854217422392

CCGGAACCAGGTGACTGTAAAA 1.20164874157

CCGGACGAGTCTGTAGCACCAA 0.225921280775

CCGGCACGCTTTGCAGATACAG 1.7183834083

CCGGCAGCCCTTCCAAAGAAAA 0.864292028102

CCGGCAGCGTCTGGACGCGGAG 0.315905489365

CCGGCAGTATGTCATAACCGAC 0.433758733208

CCGGCATAAACTGTTACATTAC 0.379489499139

CCGGCATACAATGAATCACGAG 0.251888774004

CCGGCATGATATACGTTCGAAG 1.37495763055

CCGGCCTCATGTCCGATATTAC 0.610046799474

CCGGCGCGTTCGTCGATGTCAC 0.88464907464

CCGGCTTACGTTCTTTCAGAAG 0.48753139513

CCGGGAGCCCTTAGAGTCTCTG 0.361180407949

CCGGGCGAGGGTGTTTTGCTTG 1.60977605132

CCGGGGCCATGTTATTTGTATT 0.194352543735

CCGGGTACATGTCAAAGAACAG 1.17789228897

CCGGGTGAATGTGCTGCACTAG 0.775757600592

CCGGGTGAGTGTGCCGCGTGAC 1.75885535024

CCGGGTGGATGTGTTTTAGGAG 1.16704532246

CCGGGTGGCACTCCTATGCAAC 1.19092714315

CCGGGTGGCGCTCATCTGGCAG 2.06043552706

CCGGTGCGCGCGGGTAGCTAAT 1.58077114529

CCGTAACCCATTGATGATAGAA 0.564006090505

CCGTAATGATCGGCTCGGTAAT 0.701586743392

CCGTAATTACCTGCGCCCGTAC 1.07164827807

CCGTAATTCTTTCAAGACTGAG 0.397573998873

CCGTACCAAACTGAATGCTAAC 0.459334319155

CCGTACCCCTTCGAGTATAGAC 0.446864490048

CCGTACGAGCGTGGTGGATCAG 0.204846317398

CCGTACGCCTGTCTAAACAAAT 0.409846266201

CCGTACGTGCGCCAAAGAGAAA 0.449837150887

CCGTACTCATGTGTAAGGTCAT 1.03835967686

CCGTATCGATCTCCACTACAAA 0.66198503917

CCGTATGCAGGTGCCGGCAGAG 1.51905104107

CCGTATGCCTCTGAGGGCGAAG 0.870966376825

CCGTATGGATCTCTTACACAAA 0.636675077458

CCGTATGGATGTTGACTGGAAT 0.997031771674

CCGTCAAGGCGTGTATTAGAAA 0.61857961246

CCGTCACAGTGGGCTCATCCAT 0.922988113442

CCGTCACCCTGTCTTACATATG 0.262003225872

CCGTCACGAGGTTATCTTATAG 0.145868112371

CCGTCACGTTGTCATTGTGCAT 0.399825581455

CCGTCACTCTAAGCTACGATTT 0.378906376751

CCGTCAGAACCTGCTACCAGAT 0.822140282767

CCGTCAGACTTTGTAATGCCAT 0.634233123175

CCGTCAGATACTCATGGCCAAC 0.956681381528

CCGTCATGAAATGAATACTAAT 0.270471756037

CCGTCATGAGTTCCGTAGGCTG 1.96230967861

CCGTCATTAGCTCCATGTTAAC 0.685742539572

CCGTCATTAGGTGCTAAGAAAG 1.31602322617

CCGTCATTGGGTCCTATATGAT 0.988420157984

CCGTCCAAGTGTTAAAATGAAT 0.359528575989

CCGTCCGATCGTTTTGGATCAC 0.541060498078

CCGTCCGGACGTCATGAGTCAT 0.421077465511

CCGTCCGTAGCTGACAATACAA 0.881308459075

CCGTCCTACAGTCGTGCTAATC 0.698031935654

CCGTCCTAGCCTGAATCCGCAA 1.3689060066

CCGTCTACCCCTGATTATCTAC 0.701923348362

CCGTCTCTTTCGGAATCCTTAT 0.621122468847

CCGTCTGAATCTTAAGCAGCAG 0.591377763459

CCGTCTGAATGGGCTGCTCCAT 1.59887101106

CCGTCTGAGGGTCGTCGCGAAT 0.386765762687

CCGTCTGATATTGCTGGGCAAT 1.15308470006

CCGTCTGCATCTGACTTTCAAT 0.469630172258

CCGTCTGCCGATCAAATGGTAT 0.354141918804

CCGTCTGTCGTTTAATGGTTAG 0.358610673696

CCGTCTTAATGTGGTTGCGATG 1.42368769178

CCGTCTTATTCTTATCTTCAAT 0.16279805558

CCGTCTTCAGGGGTACGATCAA 0.471002069122

CCGTCTTGGTCTGTGCGCGCAG 1.16533771799

CCGTCTTTGGGTCATACTCCAC 0.698661641852

CCGTGAAGCTCTGATCAGATAT 0.13188672326

CCGTGACAATAGCATCGTCTAG 1.24084340087

CCGTGACAATTAGGTTGGATAA 0.358429676323

CCGTGACAATTTGCTGCAGGAG 1.19234651147

CCGTGAGAAACGGATAAAGGAT 0.518477072112

CCGTGAGGAATACCTATATAAC 0.921349213799

CCGTGAGGAATTCTGACATGAC 0.548524029295

CCGTGAGTATATCCTCGCGAAA 0.725793640165

CCGTGATAGGCCGCATGCTGTG 0.827923252966

CCGTGATTAGGTGAGACAACAG 0.704943267668

CCGTGATTCTGTTCTAACTAAA 0.270180346469

CCGTGCAATACCAAGCATTGTG 0.572053994641

CCGTGCGAATTGGAATCCATTC 0.344302226781

CCGTGCGAGGCTGTTATGTTAG 1.55304812041

CCGTGCGTAACTGCTGAGCCAA 0.712078751407

CCGTGGGATGGGCCCGCTCAAA 0.443835538895

CCGTGGGTCTATGAACTGCCTC 0.997350036334

CCGTGGTTCTTTCCTGCCGGAT 0.790488076186

CCGTGTGAAGATCTTCATGAAG 0.281097325346

CCGTGTGAGTCTGCGTGTCGAG 2.11888126446

CCGTGTGGATGCGATTAGTCAA 1.11368589131

CCGTGTTAATCTCCTCCCATAA 0.615429493484

CCGTTACCAAGTTGAAACGTAT 0.694008952471

CCGTTATATTCTGCTCGTCTTC 0.572861852405

CCGTTGGATTCTCCCCACATAA 0.729770576183

CCGTTGTAACTTCCTTTTTAAC 1.0912809196

CCGTTTCTATGACATGTCTTTT 0.415017429364

CCTAAACTGAGTCTTACGTTAG 0.298136144858

CCTAAAGCTCCTGCAAAAATTC 0.62562761641

CCTAACTTATCTGGTTCAAAAA 0.341959658328

CCTAATGCATCTCATGATATAA 0.427495617875

CCTAATGCGCGTGTGGTCTGAG 0.957425901566

CCTACACTGCCACACTCGCCAG 1.19492665954

CCTACACTTTCTCGTGGATGAC 0.947592155939

CCTACAGAACCGGCTAGCTAAC 0.824176964469

CCTACAGCCGGGGACATCTGAT 0.632360064893

CCTACAGGATTTTAAATAGCAA 0.389946150012

CCTACAGTGACCCTTTGAGAAG 1.05554146578

CCTACATCCTTTGAAGTCGAAC 0.161799641224

CCTACATTTTGAGAACAAACTA 0.771166058539

CCTACCATGAGTGCTGTTTTAG 0.486003747078

CCTACCCTAACTGATCCATTTA 0.576567087328

CCTACTAATACTTGAGTAGGTG 0.301126027364

CCTACTCAGCCTCGTCAACATC 0.521725130389

CCTACTCGCTCTAAAATTTGTA 0.784547385724

CCTACTGCGTAGCATTGCGAAC 0.400311153882

CCTACTTCTGTTCATTACATAT 0.316695839834

CCTAGACAACATCGTTGGCTAA 0.724096715957

CCTAGACACAAGGATAAAAAAT 0.421468880233

CCTAGACGATGTGCAAGACCAA 0.673488548814

CCTAGAGAATGTCATAAGAAAC 0.232373627315

CCTAGAGATTTTTCTAGCATAG 0.609614117481

CCTAGATTCGGTCGTGGAGAAC 0.898361957496

CCTAGATTGTGTCGACAAGTAA 0.440948263222

CCTAGCGTGCCTCGATTTTGTG 1.00367946078

CCTAGGCCAGGTCAAGGAGAAT 0.771091716997

CCTAGGTCATCTGTAAGCAAAC 0.711061937061

CCTAGTGCCCGGGCTTGAGTTA 1.04672612347

CCTAGTGGAAGTGCTGAGAAAA 0.583836868559

CCTAGTTTATATGCTGTCACAT 0.161522762971

CCTATAGCCAGGGAAATAGCAT 0.620924153816

CCTATCTTCCCCTTAGCTAAAA 0.590380402255

CCTATTAACACTCAAGAATATC 0.254158512458

CCTATTAACTCATCAACAATAC 0.322685925172

CCTATTGCATTTCACCGGAAAC 0.594224653588

CCTATTGTATCTGATAAAGCAA 0.429127912516

CCTATTTGAGGTCTTCTCTTAG 0.61254103389

CCTCAGGCTTCTGTTAGGACTT 0.398724383075

CCTCATATAAGTGATACGACAA 0.280232166689

CCTCATGAAGGTGCTGTTCGAC 0.34072877268

CCTCCAGAACATGTAAGTAGAC 0.572588037729

CCTCCAGTCCGTGTGTCGCAAG 1.23945407309

CCTCCATAGTGTCAAGCTTAAT 0.464900203792

CCTCCATCTGGCGATTGATATG 0.38826339109

CCTCCATGTTATGCTAATCTAT 0.405408085926

CCTCCCGTCACCGGGCGCGCAG 1.52303070619

CCTCCGTAGCCTGGGTATTAAC 0.669835099841

CCTCCTAAATGTCATCTAGTAG 0.254839255125

CCTCCTCCAAGATTTTTAGAAG 0.389605367281

CCTCCTGATGGTGCGTAGTTAA 0.961200739916

CCTCGAGCATCGCTTGCTGAAG 1.33983942167

CCTCGCGATAGTGCTGTCGAAG 0.475226177214

CCTCGCGGATGGGATAGTTCTA 1.37109664651

CCTCGTTCATCTCATTAGTCAT 0.651775567355

CCTCTTGTCTCTGTTACTTCAG 1.24332055124

CCTGATCTAGCTTAAACAGGAG 0.884266987475

CCTGATGATCCTTCTTCTTGTG 0.332905040605

CCTGCACGGGATGCTTTTGAAG 1.6694393632

CCTGCAGAGTCTCGTAGTTAAC 0.866053357489

CCTGCATAATCTGAATATTGAA 0.429335162755

CCTGCTTCAGGTGCAGGCGTTA 1.88875382461

CCTGGAGAGGTTCAAGAACTTT 0.447395383997

CCTGGCGTAGCTGATCACAGAA 0.981345065061

CCTGGGCTTTGTGATAAGGAAT 0.499149823943

CCTGGTCAGACTGATCTGGCAC 1.21813896461

CCTGGTCAGTGTTACACTAAAC 0.660756102624

CCTGGTGAGTGTGCTTGAAAAC 1.32320892147

CCTGGTTAATGTCCTGATTCAT 0.445426856524

CCTGTAGCGTTTACAGAGGATC 0.989290810652

CCTGTGCAATGTGAAGAAGTAA 0.544078794668

CCTGTTCGTAGTTCTGGTACAA 0.596062770018

CCTGTTGGTTCTGCATTTGAAC 0.408561616286

CCTTAAGCCAATATCGCTTAAA 0.441399982572

CCTTAGCGAGTACCTGTTATAC 0.539681775215

CCTTAGGTATGTTCTACATTAT 0.437885244495

CCTTATCCATCTCTGAGGTGAT 0.971512472858

CCTTATTAGTCTCAGGGAGTAA 0.494029110438

CCTTCACCAACTCCCTGGGGAT 0.180296906432

CCTTCACTATCGCAAGAGCATG 1.08645916364

CCTTCAGTAGATCAATGGATAG 0.662366412995

CCTTCATAAGCTCCTGACTGTC 0.294400471117

CCTTCATGATGTGTTGCCAAAC 2.1264849735

CCTTCTCATTGTCCAACCTAAT 0.641143210114

CCTTCTTCATCTCAGGTGTCAG 0.966571847543

CCTTCTTGAGATGCTTTAAGTG 0.55461126665

CCTTCTTTATGTCATGGTAGAC 1.60487764466

CCTTGACAGTGTCTAGGACGAC 0.659215700875

CCTTGACTATGTGGTAAAAGGG 0.39897365754

CCTTGACTGCCTGAGTGTAATG 1.60099370687

CCTTGAGTGTGTGCGGCTCCAC 1.54270870782

CCTTGATTATCTCCTTGAAAAG 0.646048825491

CCTTGCCACCTTGGTGTCTATT 0.920980290015

CCTTGGCACTCTCGTCCGAAAT 0.441533759265

CCTTGTGGCTCTGCCTGAGAAC 1.22256299584

CCTTGTTTCTGTCAATAACAAT 0.403120340228

CCTTTAGACGTCCCGGATATTC 0.569726789264

CCTTTTTAGGGTGGGGATACTG 0.986142229406

CGAACATGGACTCATCAGTATG 0.618357001263

CGAACTCGCACTGCTTAAATTA 0.421444490823

CGAACTTTGGCTCCCGACCATT 0.283496755049

CGAAGAGCGCCTTATAACGTAA 0.338058987138

CGAAGTGACTGTCTTGACACAG 0.552354498502

CGAAGTTGAGGTTATGAATAAG 0.408375720915

CGACACTAATCTGAGTAGTGAG 0.855636629246

CGACGAGAAGCTGGTGAGTTAC 1.2393687816

CGAGATTTACATCGGTCGACAC 0.457187838242

CGAGGTTCAACTCATATGAGAC 0.501050060068

CGATCAGCGTGTCATGTTCCTG 0.817992901427

CGATCAGTTAGCGCAGCGGTAT 0.602001009715

CGATCGCTCTGTGCTGGGTTAC 0.279563300796

CGATCTGGGTCTCCTACCTGAT 0.981126678188

CGATGGTAAGGTCCAGTGCCAT 1.52529656247

CGCAAAACCGATGCCCATGTTG 0.721469787877

CGCAAAATATACTCTGGGTTAT 0.521892549042

CGCAAATTTGTTTCTAACATTT 0.533786800396

CGCAACTTATGCGCTTTTTTAG 0.457245847423

CGCAAGCTAAAGGTAAAACGAC 0.1455351591

CGCAAGTTCTCTGATATATGAG 0.325509673477

CGCACACAACGGGACATGTTAT 0.344527738236

CGCACACAAGTGGAAGCGAGAT 0.435656070084

CGCACACAATGTTATACCCAAA 0.503800143538

CGCACACACTCTGCTTGCTAAA 1.09025762338

CGCACACATAGTAAATTCGTAG 1.22539310654

CGCACACGACCTCATCAGCGAT 0.494903217885

CGCACACGCTCTGGCCAATTAG 1.01556810214

CGCACACGTACTCATAAAAATA 0.369482071621

CGCACACTCGGGGGATGCATTA 0.865985393189

CGCACACTGTCTCTTTACTTAG 1.47039862414

CGCACAGAATATTCTTTGAGAA 0.480214403235

CGCACAGGACGTCACGCCTTAG 2.33378044116

CGCACAGGAGGGGATCATGCAC 4.22379921684

CGCACAGTAGCTCCAGATCAAG 0.715251720304

CGCACAGTCTGTCAATGTGCTC 0.841946979972

CGCACAGTTCGTTATCCCTAAC 1.08175694381

CGCACATAGTTATCTTATTTAT 0.624915345813

CGCACATCATGTCCTTGGGAAG 0.786583238151

CGCACCCCATGTCTTAGTCATC 0.775653974594

CGCACCGAGTCCCTTACATATT 0.745190022387

CGCACCGCATCTCAAGGATAAT 0.666496818869

CGCACCGGCTGTGAAGGGTCTA 1.32131623699

CGCACCTAGGGTGCAAGTGATT 0.852632329671

CGCACCTCATCTGATTATCTTC 0.41555036067

CGCACCTGATCTCATGAGAAAG 0.669309396805

CGCACCTTCTGCGCATGTACAT 0.643949264497

CGCACGGTACATCCTATTTGAT 0.368524480644

CGCACTATGCCAGAAGTAAATC 0.208294641225

CGCACTCCAGCTGTAAGTACAA 2.90726342161

CGCACTCGGCGTTACTTTGCAG 1.1716478108

CGCACTCTATCTGATCACTAAC 0.828908568571

CGCACTCTGACCCACCCCGGAT 1.60833981883

CGCACTGATAGTCGTCAGGATT 1.12485705882

CGCACTGTGAGGGCACATGTAA 1.57913147512

CGCACTTGATGGCATAAAATAT 0.692949877635

CGCACTTTATGGGCAACAAAAT 0.789299548862

CGCACTTTCAGTCACTAAGAAG 0.626436783875

CGCACTTTTCCTCATCAGCAAA 1.06161441687

CGCACTTTTTGTGCACCTAGAA 0.760357527703

CGCAGACACAGTGAAGGACTAC 1.32326429756

CGCAGACCATGTGATATCAGAT 0.177102403216

CGCAGACGAGATGGGGGGCAAG 1.17502621149

CGCAGAGACTGTGCGCAATAAG 0.54942015906

CGCAGAGGACCTCTCGTATATG 0.686071255911

CGCAGAGTGCGTGTTTTAAAAT 0.420067083335

CGCAGATAAACTTTTCCCGAAA 0.446417054331

CGCAGATAGCCTCATTCAAATG 0.457062120449

CGCAGATATTATGGTTGTTTAC 0.899817376519

CGCAGATCCAATGATCAGACAC 0.251231645376

CGCAGATCGACTCCTACATATG 0.537331336623

CGCAGATGCGATCATACTAAAA 0.375434470888

CGCAGATGCTGTGAATGCCCAC 1.45892651825

CGCAGATTCAGTGATGCTTATG 0.147715315317

CGCAGATTGATTCAGAAGTCTA 0.416668286813

CGCAGATTTTGTTCTCTCCCAA 0.40917743106

CGCAGCCACAGTCCAGAGCATA 0.838641524104

CGCAGCCTACCTGATTTGACTA 1.00873086866

CGCAGCCTCACCACTACATGAT 1.59677420577

CGCAGCGAAACCCCTGGCCAAG 0.78435284886

CGCAGCGAATCTGGAACATAAG 0.474536682751

CGCAGCGGATGGCGTCCTCTAT 1.65813049833

CGCAGCTAGAGCGTTGGGCCAG 0.958734064357

CGCAGGAGCTCTGATGTCCGAA 0.507739615128

CGCAGGCTCCAGCATCAATATA 0.730159634969

CGCAGGGAGTGGCTATAGGGAC 0.906825602276

CGCAGGGGTTGTTAACGAGGAC 0.220689921763

CGCAGGTACAGTGCTGTAACAG 0.901398770752

CGCAGGTCATCTCCTCTCGGAA 0.37922723264

CGCAGTCAAACTGATGTGTATT 0.592389126815

CGCAGTCAGTATGCTGAGGAAG 1.23397280059

CGCAGTCTATGTCTCCCTGGTC 0.659587152172

CGCAGTCTCTCTTTAACGTGAC 1.09202779315

CGCAGTGAACGGCCACATAATT 0.342698407094

CGCAGTGAATATGATCACACAG 0.857501996078

CGCAGTGCACCTGAACGTATAT 0.668937034893

CGCAGTGCCGTTGATGTGTTAG 1.80425902525

CGCAGTGGATATGTACATTAAG 0.543635958704

CGCAGTGTGTGTCTTACATGAA 0.628789022198

CGCAGTTACAGTCAACCTGATT 0.657382208018

CGCAGTTACTGTGCGTTTGAAA 1.51940791894

CGCAGTTTAACTCCTGATGAAC 0.224603337309

CGCATACAGCCTCGTGAGATAA 0.203121583808

CGCATACAGTATCGTGGGCAAG 0.355643704817

CGCATACTATCTCCTGTCTAAT 0.290375285553

CGCATATATTTTGTTGCTGAAA 0.615738126252

CGCATATTAGTTCAAGTCAATA 0.306049862079

CGCATCCGTTGTTTTGAGTAAT 0.799849781641

CGCATCCTCAGTCAAAGCCAAC 0.689104095858

CGCATCGCCCCGGCAGATATAT 1.24551774537

CGCATGTAGCGTAGACGAAAAC 0.498297600978

CGCATGTCACTTTTTGAGGAAA 0.332323828934

CGCATTGCACGTTAGCATGAAA 0.903402718969

CGCATTTGATATCTAAACAGAC 0.23246545779

CGCATTTTGTAGGAGTTCCTTT 0.86573846035

CGCCAACAGTCTCAAACTGTAC 0.421195478818

CGCCAATAAACTGAATCCTAAA 1.17439663496

CGCCAATGCTATGATAAATGAG 1.10813589311

CGCCAATTGTGGGGACTGCAAC 0.622338958631

CGCCATATAACCTACCGCATAC 0.42550062924

CGCCATATGTCTCCTGGAGGAA 0.397839527732

CGCCATCGGTATGAAGACAAAT 0.868417820989

CGCCATGACGGTGATAAAGGAC 0.407068161239

CGCCATGTCTTCGCCCCTTAAT 0.559017915501

CGCCATTCCAATGCCTGCGTTC 0.52524621016

CGCCATTTCCTTTGTTGTCAAG 1.46162848929

CGCCCAAGATCTCTTCGAACAT 0.568587052867

CGCCCACTGACTCGATAGCGTG 2.98409517413

CGCCCAGCACTTCCTTATGGAA 1.27531909207

CGCCCAGCTTTCTATGTCTTAC 1.01034938266

CGCCCAGTACCTGGTCAATATG 0.800008681956

CGCCCAGTGTGTCAAGGATTAG 1.09591959748

CGCCCATACCGGTTTAAGTGTC 0.570982235325

CGCCCATACTCTGCATGCTAAT 0.53113299168

CGCCCCAAATCTCGCGAACAAA 0.913237514893

CGCCCCCAAGCACTTGTCGTAG 0.798526418531

CGCCCCGAATATGCATCGTCAG 1.61470765222

CGCCCCGCGCGTGATTACTCTA 1.6057617874

CGCCCCTACCTTTAATATTCTA 0.770644873708

CGCCCCTCATCTTACAGCCTAT 0.757433476095

CGCCCCTGTAGGGTAAGAAGAG 0.963682051268

CGCCCGGAAACTCTTAGATTAT 0.869539596598

CGCCCGGGATGTGGTTAGCCAG 1.0963051214

CGCCCGTACAGTGATATCTATC 0.329054885623

CGCCCGTAGACTCATCAATAAC 0.402028658412

CGCCCTCACACTCCACCGAGAA 0.665467227916

CGCCCTCGGCCTCATGAGAGAA 0.785192704237

CGCCCTCGGGCTGGTGGGAAAG 2.26871530591

CGCCCTCTCAGTCCGATTGTAC 0.337778529423

CGCCCTGAATCTTCTATTCTAT 0.488604524567

CGCCCTGGCTAGCTTGCTGAAG 1.59562958979

CGCCCTGGCTGTAGAAAGCATG 0.979050678393

CGCCCTGTAAATGATTCGAAAA 0.641090339887

CGCCCTTCCTTTGTAGACCAAC 1.44127570658

CGCCCTTGATACGCCGAATTAT 0.702857455975

CGCCCTTTCCAAGCTGCGGAAC 0.335890715679

CGCCCTTTCTTTCGAGACCGTC 1.25474907784

CGCCGACAACGTGTCACAACAA 0.581697295841

CGCCGACAGTTTGATTATAGTT 0.588742546761

CGCCGACGCACGGTAGCTCTAA 1.00502832995

CGCCGACTATGTCAATGGAAAC 0.320645197462

CGCCGAGAACGTATTGTGCAAT 0.558778899941

CGCCGAGAATTTGCAGCGCGAA 1.12511660149

CGCCGAGAGACTCGTAGTACAC 0.732631617361

CGCCGATAACATGCAGCCTGAT 0.799966001822

CGCCGATAATGAACCTACCCAT 1.02869299642

CGCCGATACTGTCCTCCTTAAC 0.150104496808

CGCCGATCCATGGTTTGATTTC 1.38313573926

CGCCGATTGACTGATGATTAAT 0.192167432358

CGCCGATTGCCTCAACCATGAT 0.908083244734

CGCCGCAAAACTGCACGCGCAA 0.344480909911

CGCCGCCAACGTGGTCAATAAG 0.236241136072

CGCCGCCACTGTCAGGCATCTC 0.825084213452

CGCCGCGAATTTCTTACAACAT 0.748904409244

CGCCGGCTGTGTCTTTCGTAAA 1.9582520421

CGCCGGGTAGTTCATTGGTAAA 1.51862679644

CGCCGGGTGACTGTTGACGCAG 1.38954317044

CGCCGGTGGCCTGATGAGTAAG 1.40158324911

CGCCGTATGGCTCGTTCTTTTC 0.673381653029

CGCCGTGACAGAGCTAAATTAG 0.830236969948

CGCCGTGCGCCTCCTCGCAAAA 1.0200610338

CGCCGTGCGTCTGATAGTACAA 0.38168094011

CGCCGTGGAACTGATGCTACAA 0.80297907389

CGCCGTGTACGTGCTTTGACAC 0.236019789392

CGCCGTGTGCCTGTATTGTGAC 0.442773544275

CGCCGTTAAGATCTATGTGTTG 0.902547451477

CGCCGTTACTCTCGTCCTAAAA 0.523474821243

CGCCGTTAGCGGTGGCGTTAAT 1.93815213498

CGCCGTTCACGATCTAAGAGAA 0.648034406722

CGCCGTTGATGTGATTAACAAT 1.05754821433

CGCCGTTTGTCTGAAGACCTAG 0.492444956211

CGCCGTTTTTCTCATGTTCTTA 0.703603518039

CGCCTAGAGGGGCCTTATCGTC 0.927785444367

CGCCTAGTACGTTCCTCTCATC 1.0581465699

CGCCTAGTCTCTCCTGAGAAAG 0.567955282839

CGCCTATATCCTCGTACAATAT 0.758266969063

CGCCTTGAATCTCATGAGTAAC 0.805277720345

CGCCTTTGATGGCTTACAATAG 1.15986105781

CGCCTTTTTTCTGCCGCAAAAT 0.782940878478

CGCGAATTACCTCCAATGCATG 0.621830729168

CGCGAATTCACTGATACATGTA 0.957744959147

CGCGATTGGTATGCAACACTAA 0.463671529415

CGCGCACATGCAGTTTTGCATA 0.696041499178

CGCGCACCCCGTGATGTGGCAA 1.50793785034

CGCGCAGAATGTTTTTGACAAA 1.22433857449

CGCGCAGGGTCTCACAAGTCTT 0.934276567061

CGCGCAGTGCTTGCAAAACTAT 0.314316873394

CGCGCATTACCTGATTGAGAAA 0.751382842992

CGCGCATTGAGTGATAGCTAAA 1.26572700106

CGCGCCCTGACGCCCATAATTA 0.544923359239

CGCGCGTTTCCTTTATATACTA 0.790041931139

CGCGCTACCGTACTGTAAAGTA 0.358326343169

CGCGCTACGAGTGCTCCTTTAT 0.856104266761

CGCGCTAGGCGTCCTGGTCTAT 1.41086981716

CGCGCTCGGTCTCGTACTAGAC 1.37263293785

CGCGCTTAACGTGAGAAAGGAG 1.42800050695

CGCGGAATCTGTCCTATTTAAG 0.869081466814

CGCGGAGCCTCTTAAGACAGAC 0.318590579217

CGCGGAGGTATCGCATGTGAAG 0.405554998297

CGCGGAGTGTCTTCACATACAG 1.09339330741

CGCGGAGTTTCTCGTATCTTAG 0.699948170861

CGCGGATATTGGCCAAGCAGAC 0.240879086456

CGCGGCGAATATCGTGAGTGAA 1.66755995067

CGCGGCTCCTCTGCATAACATG 0.545715704769

CGCGGGCCGGGACAAGGCGTAT 4.62090775477

CGCGGTATACCTGATCGGTAAA 0.495107865986

CGCGTACACTCTGTCAAGCTTA 0.528706129502

CGCGTATAATTCGATCCGATAA 0.680964884259

CGCGTTTCGTGTCCATAAACAC 1.24804251809

CGCTAAGAACCTTGGGAGTAAG 0.741285129432

CGCTAAGAAGGTGAATAGAAAC 0.300098149858

CGCTAAGAAGGTGCTATCACAC 0.808382830929

CGCTAAGACTCTGATAAATCAG 0.80459883854

CGCTAAGATAGTCAGCTCCGAA 0.875544559752

CGCTAAGGATCTCCTGGTATAT 0.663983207215

CGCTAAGGTTGTGCAAAAAGAC 1.05506365346

CGCTAATAGTTTTCTAAACATC 0.487415039732

CGCTAATTGAATCGTAATTATT 0.448674126745

CGCTACGGGTATCACAAATAAC 0.971266362951

CGCTACGTTGATCTCGCTGGAT 0.763121052468

CGCTATGACTTTGGTAAAAAAG 0.390847937736

CGCTATGATAGTGCTACTAAAA 0.206723595478

CGCTATGTCTATCCACTTAGAT 0.653484531478

CGCTATTAAACTGGTATTACAG 0.458816006601

CGCTCACAGAGTCGCTCCCTAG 0.289488795844

CGCTCAGAAAGTGGACACAATG 0.865423055855

CGCTCAGAATCTCTAGCAAATG 1.56426489149

CGCTCAGACTCTCTTGCAATTC 0.431030781703

CGCTCAGAGCGTGTTGGGACAA 1.34566874717

CGCTCAGAGTCCCACCACTCAG 0.769077304809

CGCTCAGATTCCCCTAGTAGAA 0.636545113328

CGCTCAGCATCTCTACGCCTAC 1.45998029558

CGCTCAGTACGCCCAGGAAAAA 0.45705748118

CGCTCAGTAGAGGACAAATAAG 0.345827032945

CGCTCAGTGGGTGTTCCGTTTA 1.35102306498

CGCTCAGTGTGTGTGCTCTTAA 1.3586219171

CGCTCATAAAATGTTGACCCTA 0.801301188351

CGCTCATAATCTCATTGAGTAA 0.161262321872

CGCTCATCTTATGCAGTCTATG 0.867598559003

CGCTCATGTGGGTCAACGTAAC 1.86486256987

CGCTCATTAGCTGATTCGTAAG 0.38498671957

CGCTCATTGGGTTGACATAGAG 0.741140660633

CGCTCCCCCACTCATGTTATAC 1.62534261341

CGCTCCGAAGGTCCTAGGTGAA 0.794143449483

CGCTCCGCAAGTGGTCGGGGAG 2.58379732119

CGCTCCGTATGTCATAGCGTAC 0.764593766488

CGCTCGCGTCGTCACGCCCTAC 1.57869706941

CGCTCGCTCCGTGCTCTTGAAA 1.45483230371

CGCTCGGACAGTGTAAAGATAA 0.673934704477

CGCTCGGATTGTCATTAGCAAC 0.935543537631

CGCTCGGGAAGAGTCGGGTGAG 2.4272954177

CGCTCGTCATCTGGGGTGACAT 1.26049702643

CGCTCGTCGTCTGTGTGCCGAG 0.453385491753

CGCTCTCCATCTCATGAGTTTC 0.965216859225

CGCTCTCGCATTCGATAGAAAC 0.846389322882

CGCTCTCGCTGACTTGCCCAAC 0.744149254843

CGCTCTCTAATTGATAGGAGAA 0.562643277051

CGCTCTGAAGTTGCTACCTAAT 0.544573658871

CGCTCTGGAACTGATTGGATAA 0.660726872176

CGCTCTGGACATGGTCGGTTAG 1.27314688757

CGCTCTGGACGTCCCTGACGAT 0.678964616407

CGCTCTGTATGCCTATCCACTT 0.480795280433

CGCTCTGTATTTAATAAGCCTA 0.464770459826

CGCTCTTACGGTGATTGTTAAC 0.506259330636

CGCTCTTCCCCTCCTTGTCCTG 0.264176997973

CGCTCTTTAGGTTATCACCAAT 0.63628139814

CGCTCTTTGTCCGATGTCAAAA 2.11141892377

CGCTGAAAAGGCCACTGATCTG 0.587552650946

CGCTGAACATCTGCGTCCTAAT 0.51782071628

CGCTGACATCGTCAAACCGTAG 0.867921861393

CGCTGACCACGTGTTAATAAAG 0.506498483426

CGCTGACCGCCTTATTTAGTAC 1.03148190491

CGCTGACCGTGTCCTGAGGTAA 1.50518938913

CGCTGACGCTCTTGATATACAT 0.290783896183

CGCTGACGCTGTGCATATGCAG 0.847926279979

CGCTGACTGATTGATGAAGTTT 0.386096204432

CGCTGAGACTATGATTCGGTAT 0.928657396801

CGCTGAGCACGGTATGTGTGAC 1.18445205167

CGCTGAGCTCGTCATCCTGCAT 1.68348475901

CGCTGAGGAAGTGATCCGGGTT 0.581165009882

CGCTGAGGAATTGCTTAGATTG 0.227267032714

CGCTGAGTATCTCATAGAAGAT 0.788785100452

CGCTGAGTGACTGCTCTGGAAC 1.41894150325

CGCTGATAAGGTGCGGGGAAAT 0.482731633904

CGCTGATGGTTTGATGAAGAAG 0.652988127718

CGCTGCATCACTCTTACTTAAC 0.331030451116

CGCTGCGTATCCGTAGCGAAAA 1.72616931028

CGCTGCGTCTCTGAATGACAAC 0.811816639957

CGCTGCTAAGTTTATTATCTAA 0.315726637423

CGCTGCTTATATGCATACTTAT 0.715787681918

CGCTGCTTCGCTGATAAGCAAA 0.369907231883

CGCTGGAAGCGTGATGAACAAA 0.152044468135

CGCTGGGAGAATGATCCAATTA 0.496048695103

CGCTGGGGCTTTCCGGGTCGAG 0.276322147754

CGCTGGGTACTTCGATAGACTC 0.81297855201

CGCTGGGTATGTGTGGCCTAAT 2.44954923529

CGCTGGGTCTCGCTTGCAATAC 1.54582361474

CGCTGGTCAAGACGTATGTGTC 0.180864237817

CGCTGGTTATCTGGGTTATAAC 0.796258564553

CGCTGTCACACTCATCGCATAA 0.526913183108

CGCTGTCTCACTGCTAAGGATT 0.977935188877

CGCTGTCTCGCTGGATCTTAAT 0.725152771633

CGCTGTGCTGGTCCTTAGTGAC 1.70691882818

CGCTGTGGCACTCAGGCATTTG 1.50489784044

CGCTGTGGCAGTCCAAGTGGAA 0.919164823577

CGCTGTGGGGTTCATAATCAAG 0.29313212889

CGCTGTGTCAATGATGTAAGAC 0.539644299255

CGCTGTTAACATGATGGCATAT 0.710243798257

CGCTGTTAAGCTGAGGTATAAT 0.145755234345

CGCTGTTCTGCTTCTTAATGAT 0.61738423941

CGCTGTTGGCCTGCATGGCGAT 1.34008957738

CGCTTACAGAGTGGTGCGTCAC 0.305572248017

CGCTTCTATTCTCAGTAGAGAC 0.221185400667

CGCTTGTTAGGTGAAGAGTGAA 1.35712983862

CGCTTTCGACCTCGTGGTGGAG 0.915553627645

CGCTTTGAGCGGCAAAACTCTC 1.19991539548

CGCTTTGCAAGTGTGGCGATAT 2.1249481924

CGCTTTGTATGTGCTCAAAGAG 0.713066939353

CGCTTTTGAACGCCTTGACAAG 0.292825410285

CGCTTTTTCTGTGAATAAAAAT 0.853153378422

CGGAAACAATCGCGTAACGGTG 1.79731993445

CGGAAACTATCTTGATGGAGAC 0.461902408141

CGGAAATTTTGTGAGACACGTG 1.1205205569

CGGAACGAAACTTCAGAATCTC 0.318730597342

CGGAACGATAATCCTACATAAA 0.128947913408

CGGAACGCGTCTCCTAGATAAC 0.777108903504

CGGAATCAACTTGAAGGCTTTC 0.978050029773

CGGAATCAATGGCGACGGTCTG 0.423516150966

CGGAATGGATATGCATAACAAT 0.21345159267

CGGAATTGGCCTGGTATGCTAG 1.04190473327

CGGAATTTGTCAGTTCGCCATC 0.444500912377

CGGACACAAACTGCTGGCTGAG 0.51051682961

CGGACACAAATTCATAGCTTAC 0.675105662369

CGGACAGAGGGTGATTCTCTAG 0.496701082911

CGGACAGATTTTGGAGGTTTAA 0.80307613824

CGGACAGGAGGACCTAACATAG 0.68448162577

CGGACAGTAGCTCCGCGAAGAT 0.466527861759

CGGACATAACATCCTGGACCAT 0.593903496586

CGGACATAGGGTGCATAATGTA 0.942528978913

CGGACATCCCGGGCGTCTTGAG 1.14949128504

CGGACATCCGCTCAATTACTTG 0.500185608156

CGGACATCGTATGCTTCCAGAC 0.416475785712

CGGACATTGAGTCCTAGCAGAC 0.986362775436

CGGACCCAACGTCTTCATTAAT 1.00148858497

CGGACCGACTTTCCTCCTACAT 0.883384333514

CGGACCGTAGATGCTTGATCAT 0.966131974026

CGGACCTGCCGTGACAATGAAC 1.01653577881

CGGACCTTAGCTGAAGGAGCAA 1.20657321369

CGGACGGAGACTGCAGCGGTAT 0.398464752452

CGGACGTAGCCTGCAAAATCAC 0.865378196586

CGGACGTTATCTGATAACTGAT 0.754591650238

CGGACTAGGTCTCATGCGGTAG 1.87513530839

CGGACTCAGGATTATCCACTTA 0.516947872699

CGGACTCAGTCGCGACGAACAC 0.705160086491

CGGACTCTGTTTGCATGCCTAG 1.32326562931

CGGACTGAATATGCATGCTCAC 1.22602089191

CGGACTGACAATCATCGTGAAG 0.861557921169

CGGACTGATTCATAATAGCATT 0.466359578993

CGGACTGATTGTGCTGTCCGAG 1.01819723517

CGGACTGTCGATGCCAAACATT 1.82688736445

CGGACTTCATGTTGTCTACGAA 0.648780606849

CGGAGACACCCTTTTGCATCAT 0.68792633075

CGGAGACTCTCTGGTACGGGTC 1.53502697394

CGGAGAGAATCTGTACACTTAG 1.10716140091

CGGAGAGACCCTCCTCGTCTAT 1.23470206311

CGGAGAGAGAGTCCAAGATAAC 0.681608590516

CGGAGAGAGCTTCATTCAAGTC 0.539304088347

CGGAGAGATCTTCAAGCTGAAT 0.885235973756

CGGAGAGTATCTCAATATGTAG 1.61268348247

CGGAGATAAGCTTAAAATCTAG 0.395220911023

CGGAGATACTAGGATACGTTAC 1.04039271455

CGGAGATGCGCTTATCACGTAG 0.752470998915

CGGAGATGTCGTGATGCCTTAT 0.425566350702

CGGAGATTAACCGAGCCCACAT 0.196007462489

CGGAGCCAAGCGGATAAGCCAC 0.757806602555

CGGAGCCTATTCGAAAGATGAT 0.434041008232

CGGAGCGCATCTCCGAAAAGAC 1.88414478853

CGGAGCGTAAAGGAACGACAAT 0.602857965007

CGGAGCGTATCGACTAGTGAAC 1.03582303063

CGGAGCGTATGTGATCCATTAG 0.839217696775

CGGAGCGTGTTTGCAACAATAC 0.849800875002

CGGAGCTGCTGTCCTAAGAAAT 0.621554693096

CGGAGGAACTTTGGAGATCGAT 1.18915397196

CGGAGGTAATCTCCCTTGTCAC 1.0802138589

CGGAGGTATTGTGAATGTCAAG 0.336452751652

CGGAGTACAACTCAATCGACAG 0.38561973102

CGGAGTCGAAGTGCTGATATTA 0.50276304445

CGGAGTCGCCCTCTAAAGTTAC 0.440495909901

CGGAGTGAATCTTATAGTAAAC 0.14097142183

CGGAGTGAATGTGCTACTTGAC 1.52964726631

CGGAGTGACGGTCATACAGCAG 0.66962364904

CGGAGTGCGGAGGATAGTCATA 0.60480882289

CGGAGTGGATGTTAACTGGCAT 0.876810106755

CGGAGTGGTTATGCTCGCCAAG 0.705407283913

CGGAGTTATGGTTCTGAGGCAC 0.753610306534

CGGAGTTGCAGTGGTGTAGTAA 0.653917792502

CGGAGTTGCTCGCTCAGGAGAG 1.14930963537

CGGAGTTGGCCTGGCATGTTTA 0.98354277267

CGGAGTTGTTGAGTATCTTTAC 0.489213028325

CGGAGTTTAAGTCCAGTAGCAC 0.789898270292

CGGATAACAAGTGGAACATGAA 0.794746315983

CGGATAGGACCTGCTAGTAATC 0.812721294823

CGGATATAGTATGCAAATCTAA 0.449995255682

CGGATGGAATTTGCTCCATATC 0.629921877336

CGGATGGTTATTGCTTATCGTT 0.943107516287

CGGATTAACTATCATAGTTGAT 1.08860726876

CGGATTGTAAAGCCATCAAAAC 1.11736060818

CGGCAACGATTCCCAAATGTAT 0.576139367954

CGGCAACGCTATGCTGGGTGTG 1.05995110527

CGGCAACTAGCTCAATAACCTC 0.617733880571

CGGCATCAAACTCTAGGTTCAG 0.864771235231

CGGCATCAAGGTTTCTAAGAAT 0.330101071303

CGGCATGAATTTGATCCTCAAA 0.660171048052

CGGCATGCTAGTTCTGAGAAAG 0.491937192815

CGGCATGTCTGTGAAACGGAAG 0.392324504331

CGGCCACTATCTCATCAGCGAA 0.693131326365

CGGCCACTCTCTTCAACTCAAC 0.296255470395

CGGCCAGCATATGGGCGTATAT 0.883088980749

CGGCCAGCCAGTTGTTAAGGAC 0.765507635663

CGGCCAGCCGGTTCTCGGACAC 2.59815421997

CGGCCAGGAAATTAAAATGAAT 0.627659738393

CGGCCAGGACGGCATTAGGAAT 0.657126165039

CGGCCAGGTTCTTCGCGCTAAA 1.28500488266

CGGCCAGTATCCTAAACATCAC 0.753666171863

CGGCCATAAAGTGCAAGTGCAC 1.1545961045

CGGCCATACGGTGAGGGAAAAA 1.61768593931

CGGCCATAGTATCGATCGCAAG 0.742471800984

CGGCCATGCTGTGAGTCCTAAC 0.988193801907

CGGCCCTTATCTCTATTTACAG 0.63366080434

CGGCCCTTGTTTGATTGGCTAG 2.17864512778

CGGCCGGACCAGGGTGCGGCAT 2.47709197657

CGGCCGTTGTCTCAGTGCGGAG 1.36910863968

CGGCCTACTTGACGAACTATAA 1.07684941509

CGGCCTAGCTATGCTAGGGATT 1.09759158257

CGGCCTCAAATTTATAAGACAC 0.288177872066

CGGCCTCAAGGTGCTTACCGAT 1.53610815822

CGGCCTCCATTAGCTCGCACAC 1.40692290693

CGGCCTCGTAATCGTAATCGAT 0.96203885699

CGGCCTCTATCTGGATGCATAA 1.37524149563

CGGCCTGACTGTGATCGGATTG 0.175679103514

CGGCCTGATGCTGCTAACTGAC 0.784698222837

CGGCCTGTGTGTGCTATATAAG 1.14788734813

CGGCCTGTTCTGGCTGGTGTAA 0.71418566236

CGGCCTTAATTGCATAAGGTAC 0.357224403417

CGGCCTTGAAATCATATGGCAT 0.357590450693

CGGCGACTCGGTGACATATTTG 1.10143332395

CGGCGAGAAACTGGAATAAAAG 0.728972227881

CGGCGAGGCTGTCCTTAGCAAC 2.20020602382

CGGCGAGGGAGTCTTCTACAGC 1.17455247945

CGGCGATGAAGTGGTTCAGTAC 0.671210106846

CGGCGATGGTGTCAAAGAAGAC 1.10424066565

CGGCGATTATGTCCAACCTTAG 1.10776231152

CGGCGCCTGTGCCCTCTAAGTG 1.12231394365

CGGCGCGAATATGAAGGAAATC 0.684261211395

CGGCGCGGAGCTGTATTATTAC 1.59283504858

CGGCGCGGATGTCAGTATTAAG 2.14959867774

CGGCGGTGAGCTCATATGGAAG 1.7464693065

CGGCGTATACCTTCCGCCTCAA 0.965216290347

CGGCGTGATTCTGTAGTATTAA 1.26830816196

CGGCGTGCAAATGTTCATTAAC 1.13811354677

CGGCGTGGACGCGCTGTGCTAA 1.13249679915

CGGCGTGTGGGTTAACCGCTAC 0.477764896254

CGGCGTTAATTTGCTACTTTTG 0.509967681117

CGGCGTTAGTATCAATCGCTTT 0.293114406113

CGGCGTTATTCTTTCTGCAGTT 0.61346767411

CGGCGTTTTCCTCTTTCGTAAT 1.05021297744

CGGCTACAGTGTCATATGTAAA 0.628809292943

CGGCTAGATGCTGGATGTTGAT 0.633045726122

CGGCTAGGCTCAGCAGTACAAT 1.12783244935

CGGCTAGTCTATGTAAAAGAAA 0.509739039198

CGGCTTGAATGTGTTACTCGAG 0.84890160543

CGGCTTGGGAGTGATGGTGGAC 2.03214062069

CGGGAAGAATGTGTTCATAAAC 0.651522653195

CGGGAAGCACCGGTTGAACGAG 0.691841703938

CGGGAAGCCTCTCTACACGGAA 0.582592094894

CGGGAATCATATGCTAAAGGTT 0.555957133384

CGGGAGTCCAGTGATTAGGATA 0.759696661624

CGGGATGTCTGTGATATCATAG 0.911508364957

CGGGATTACTCTGCACGCAGAG 0.650321262653

CGGGATTCATTTGTGTGGGTAA 0.845942320906

CGGGATTTCTCTGGTTACTTTC 1.64445300599

CGGGCACAGTTTCCAGTTAATC 0.611497038574

CGGGCAGACTATCAAGAGTCAT 1.17052003885

CGGGCAGAGGGTTGTTTGGGAC 1.79511524042

CGGGCAGCCAGCGATACATAAT 0.918076328552

CGGGCATAGACTCGGCCCGCAA 0.932093721825

CGGGCCACAAGTGCGATTTATT 1.0613449667

CGGGCCCAACGTCCAGATTAAA 0.351569574054

CGGGCCGTATTCGACTCATTTT 0.921514664862

CGGGCTCGCGGTCATTGCATAG 0.930986452041

CGGGCTGACTCTGACAGTTATA 0.292256247427

CGGGCTTGATAAGATTAGATAA 0.842322290289

CGGGCTTTTACGCCCTCGGATG 1.42267785902

CGGGGAGAAGGTGTTCATGTAT 0.912419573294

CGGGGAGTAATTTCGTAAGCAT 0.284717384428

CGGGGATGCTGTCATGTGCAAT 1.09620045874

CGGGGATTTGCCGGTTCGGAAA 0.172425196065

CGGGGCCACTTTCTTTCATTAC 0.453164983278

CGGGGCCTGGCTCATTCCATAC 0.52570116668

CGGGGGGACAGACGACGACATA 1.78638942724

CGGGGGGACTCGTCTGTGGAAG 0.291685943011

CGGGGGTCTTCTTATTACTCAG 0.98717893065

CGGGGTCTCAGACCTGGTTAAG 1.05454756561

CGGGGTGAACGTCAAGTCAATT 1.48686645349

CGGGGTGACTCTAGATCCATAG 1.90253032495

CGGGGTGGATCTGCTGTTTCAT 0.87821843838

CGGGGTGGATTTCCAAGTGAAG 0.721242681707

CGGGGTTAATGTCACCCCTAAC 0.688469012978

CGGGGTTAGTGTGAAACCTCAG 1.05006207223

CGGGTACTATCTCGCTTTATAT 0.349462483418

CGGGTAGCATCTCGTTGGTTAG 1.08301158705

CGGGTAGGTTCGCAGAAAATAC 1.35439475516

CGGTAACAATGGGCTGGACTAC 0.442527735548

CGGTAACCGTCTCCTCGCTGAG 1.51706379453

CGGTAACCTTCAACAATATTTG 0.323794884831

CGGTAAGCTACTGCCACTGAAT 0.556143084399

CGGTAATACTCTGTAAAATAAT 0.726325611518

CGGTATCCTTCTGCTCTGAGAC 0.492713448113

CGGTATCTAGCTGCAAGAGTTA 0.711938124845

CGGTATGCCGGTGCTCAGTGAT 0.434946347132

CGGTATTGGAATCACTGAAGAC 0.836039603279

CGGTATTTAGCTTTTGCTTTAC 0.552537763248

CGGTCACAATCTGCTGCCTGTA 0.641532830427

CGGTCACAATGTGGCATACTAA 0.677512622171

CGGTCACACTGTGACAGCCGTT 0.633813048197

CGGTCACGGAGTCCAGAAGATC 0.45105515903

CGGTCAGAAAGTGGTCCGCGAT 1.78045122396

CGGTCAGAATGTCGTCGCCAAT 1.51816662929

CGGTCAGAGTGTGAAATAATAG 0.327061129352

CGGTCAGCATGTGCTCTGAAAC 1.1997082226

CGGTCAGCTACTCCTCGTAGAG 0.924469806835

CGGTCAGGTGGTCCTTGCCAAA 1.41194918097

CGGTCATAACGTGTAATTGAAC 0.58266162848

CGGTCATTATTTCGACACTAAT 1.27048290991

CGGTCATTTTTTTAGTATCGAG 0.534897160058

CGGTCCACACATCTTCATACTC 0.641810977049

CGGTCCCTTTGTGCTGCTTTAA 0.983623998459

CGGTCCGAAGGTGAGGAATCAC 0.790614514154

CGGTCCGCATGTGTGCCAATAA 0.639481256009

CGGTCCGTGGATGATCGAATAA 0.534693756585

CGGTCCTACACTGGAGGCTAAC 0.755188887755

CGGTCCTATGCTGTTACTATAT 0.712252582939

CGGTCCTGGGTTGCTTCCGGAG 2.43166741093

CGGTCGCCAACTGGGTCTGTAT 2.03119186137

CGGTCGCCATGTGAACACGCTG 0.402590557273

CGGTCGTGCCATGGTTCTAAAT 0.965133917365

CGGTCTCAACCTGCTTGGATTG 0.536351144637

CGGTCTGAACTTGATCCCCCTC 1.11057233331

CGGTCTGAATGTGGTATCCATC 0.499095333037

CGGTCTGAGCTTTTAAATTTAT 0.35078414705

CGGTCTGAGTGTCCATAATATT 0.500327696548

CGGTCTGCATCTGTGAACGTAC 0.752948565846

CGGTCTGGATGTGACGCATAAC 0.580285497973

CGGTCTTTAACTGGTAATTAAG 0.657971447013

CGGTCTTTATCTGTAAGAGGTG 0.814595906271

CGGTCTTTCGATTCACCCTAAT 1.41631558963

CGGTCTTTCTGTCCCAGAAAAG 0.47362698005

CGGTCTTTGCATGAATGCTAAA 0.547480205779

CGGTGACCCCCACGTGACGCAA 1.5988779647

CGGTGACCGAGTGCCCCTTAAA 0.542266619377

CGGTGACTGCCTGCTGAGTGAG 3.5032264924

CGGTGAGGGGATGCATGGCTAA 0.767646920965

CGGTGAGTAGTTGGAGGCGAAC 0.167072589002

CGGTGATAAGCTTGTAGATGTG 0.808476416633

CGGTGATACTCTCCTTTATCAG 0.960465789435

CGGTGATCTCGTTGTGTATTAG 0.165804202694

CGGTGATGCATTGCGGATTAAG 0.72228509606

CGGTGCCGGACCGAGACAATTG 0.598204861368

CGGTGCCGGGTTTTATACCCTT 1.78305068228

CGGTGCGACTATCATTCCTCAC 0.934218702704

CGGTGCGTTTATGCCCTGAAAA 0.784527132993

CGGTGCTAATGTCGTTATGTTA 0.504795323924

CGGTGGAAATTTGAAGTGCCAA 1.06652222862

CGGTGGCAGCTTACTACACGAT 1.01777697048

CGGTGGTAACCTTATCCTTTAA 0.751557416851

CGGTGGTCGTGTTATATATTTG 1.23401206331

CGGTGTCCATGAGTAGAACTAC 1.25352357692

CGGTGTCTAAGTCACGATAAAC 0.654217102828

CGGTGTCTCTCTCCAGAGCAAG 0.620517588691

CGGTGTGACCGCGCATGAGGTG 1.48119636211

CGGTGTGAGCGTCGAAGAAATC 1.18187290432

CGGTGTGCATTGGAACACTAAC 0.845196106136

CGGTGTGGTACTCAACATCCAG 1.33261035617

CGGTGTGTACCTCTTTAAAGTG 0.488993917638

CGGTGTGTGACTGCATGAGTAC 0.662515246523

CGGTGTTAGTCTGATCGCAGAA 0.755297104073

CGGTGTTCATGTGTGCGACCAC 0.819744755677

CGGTTACAAACTGATGAAATAG 0.237204409359

CGGTTACAATTTGTCGGTTATG 1.09918341217

CGGTTACACTGTGATAGTATAT 0.147538073199

CGGTTACATTTTCTTTAGGCTG 0.255925239433

CGGTTACTCTGTCGGTACCTAG 1.01680193576

CGGTTGCAAGCCCACCCCATAA 1.53999936209

CGGTTGCCATCTGCTTGTTAAG 1.07820588518

CGGTTTGGATCGCCTGCCGGAT 0.939358635536

CGGTTTTTGTATGGTATACAAG 0.77220253156

CGTAAAGACCGTGTGCGGTAAG 0.777265418582

CGTAAATTCAATGCACGTAATG 0.442092481082

CGTAATCAATGCTATAGCAAAT 0.63989936784

CGTAATGCATTTCTATCCTTAT 0.570583018969

CGTACACCATTATGAAGAAGAG 0.919825548837

CGTACACTATATGACTACAGTT 0.280325568782

CGTACACTATGTCCCTTACTAG 1.10706440828

CGTACAGAGTGTCTTACTGTAT 0.439965977431

CGTACAGCAACTCGTTGATATA 0.233451737307

CGTACATATTGTCATGTCGAAG 0.434341828909

CGTACATCATATCTCAGGATAG 0.377449250947

CGTACCCGATCTGCTCGTATAG 0.542905169182

CGTACCTACAGACTTACACCAG 0.851270396586

CGTACGGTTAGTCATCCAGGTA 0.678887501286

CGTACTCAACAGTCACCATTTA 0.488291610634

CGTACTCAGTCTGTACTTGATT 0.96020199226

CGTACTCTATCTGCCGCACCAT 0.259437890748

CGTACTCTGTCTGATAGCGTTT 0.392252396338

CGTACTGCCTCTCCTTCTTGTG 0.929457755455

CGTACTGTGCTCCATACGTTAT 0.734776167506

CGTACTTAAGATCGGAGATATG 0.549327059665

CGTACTTATTGCCCAACTTATG 0.193138262037

CGTAGAAAATGGGGCGTGGTAC 0.809350063975

CGTAGAAACCGTCATGCTTAAT 0.378248246029

CGTAGAAAGCCTTCATGTAATG 0.437291952861

CGTAGACAATATTATGCGAGAA 0.479800153726

CGTAGACAATTTCATACTATAT 0.379975118976

CGTAGACGCCTTTGTTATAAAC 0.283108678365

CGTAGAGAATGAGGACGCACTT 0.6596010285

CGTAGAGGGAGTGCGTGGAATC 1.28012453867

CGTAGATGTCCTGCGCAAAAAT 0.882173469118

CGTAGATTATCTTCGACGGCAA 0.546152350287

CGTAGATTCTCTCGATATTTAG 0.550082426422

CGTAGGGTTTCTGCTACGGTAG 0.233823013797

CGTAGGTAAAGCGCTAACGAAC 0.748340557842

CGTAGTCACGCTCATTATGTAC 1.01468283223

CGTAGTGTATGTTCAGGATCAG 0.634494967362

CGTAGTTCATGTCATCGCTTTC 0.154498466557

CGTATATATTATGATAGATTTG 0.309524012673

CGTATATTATCTCTTCCATCAG 0.639628232738

CGTATGCTCTGTCGAGCATTAG 0.472599545193

CGTCCACACCATTCTAAGAAAA 0.388967306926

CGTCCAGTCCCTCCAGCGGGTC 1.01345666883

CGTCCATACTCTCCCCTACAAT 0.378929085791

CGTCCTGACTGTGGAGGGCTAA 0.760758853192

CGTCCTGCGTGTCCTTAATAAG 0.639134833712

CGTCCTGGGTCTCCTCTAAATG 0.940485794081

CGTCGAGAACTTGATTATCGAA 0.570777612588

CGTCGAGAATCGCTTACCCAAA 0.807550810668

CGTCGAGACACCGTATTATGAG 1.02693692474

CGTCGCGAGCTTGAATGTTGAT 0.517471121659

CGTCGTGACGGTCATCTCGCAC 1.22903481109

CGTCGTTAGGCTGATTGGCAAC 0.32629518515

CGTCGTTATGATCAGTAGAAAA 0.614380101515

CGTCGTTGGGATCATCGCTGAT 1.28459710106

CGTCTAGGATCTGCTAAGGTAC 0.219968444728

CGTGAAGTCCGTTTTTGTTCAT 0.454849486103

CGTGATGAGTATCCGACGTATT 0.59592803307

CGTGCACAGGGGGACATTATAT 1.21970261383

CGTGCACTACCTCATAGTTTAA 0.459869123446

CGTGCATAAAATCTAGCTCTTT 0.117363260119

CGTGCCCTACGTCTTATTCAAG 1.37978934343

CGTGCCTCGAGTGCTTCATAAA 2.3529167138

CGTGCGCGTACTGAGCACGAAC 1.85195333709

CGTGCTCAGTGGGAGATAGGTC 0.319495644119

CGTGCTGTGTCGGAATGAGTAG 0.753869304152

CGTGCTTCTACTGAGGAATTAC 0.469501489396

CGTGGAGACCCTCAAATTAGAA 0.558145304394

CGTGGAGACTTTCTATGAACAG 0.989440895403

CGTGGAGCGTACCCTACGGGTA 0.796712280096

CGTGGGGAGTCTGATAACATTG 1.32185781335

CGTGGGTAGTGTGATAAGCAAG 0.534665321603

CGTGGTTGTTCTCCTATACCTA 1.08362107542

CGTGGTTTCGCTCGTGTGATAA 0.932877378085

CGTGTTCTGATTTCTATACAAA 0.383005417651

CGTTAAGAGCCTCCTAACTTAG 0.946833387351

CGTTAAGCGTATGGTGGACGAA 0.465055302357

CGTTAATGCGGGGAAAGCATAC 0.840732497537

CGTTATCACCCGGATCTCAGAT 0.560244537751

CGTTCACCAGCTGAACTGCTAG 0.315905489365

CGTTCACCATCTGCAGGCAAAG 0.917659232905

CGTTCACTCACCCCATCATCAT 0.704833640262

CGTTCAGCTCCTGTTAGATATA 0.689580796499

CGTTCAGTTCCGCAGCGAATTC 0.462719699192

CGTTCAGTTTGTGTATGAACAA 0.397342588998

CGTTCATAATCTGAAAGACAAA 0.434382314867

CGTTCATGAATTCCAGGTACTA 0.980824808283

CGTTCCCAACGTCTTCAAGTAG 0.686824782157

CGTTCTCTCTCTGATGTTTAAA 0.159816958538

CGTTCTGAATATGTAAACCCAT 0.292148911749

CGTTCTGCAGTTGCAGGATAAA 0.932306902542

CGTTCTGTGTGTGTAGACACAA 0.912111754449

CGTTCTTTAAGTGAGCAGTGTA 0.708498289728

CGTTGACAATGTCCTCGATTAC 0.416489348033

CGTTGAGCCTACGAAGACATAC 0.790096438948

CGTTGATCATCACCAAACAAAT 0.534505254598

CGTTGATGATGTCCGTGTAGAC 0.662755740671

CGTTGATTATGTGATCCTAAAA 0.593223378718

CGTTGATTGGCTCCAAGTTGAG 0.36651013395

CGTTGCGCATGTGTTGAGGTAC 0.725750911774

CGTTGCTAAGGGGCAGATATAG 0.473020966397

CGTTGCTAATTTGGACTATGAG 0.243861649294

CGTTGGGAATCTGCACACCGTC 0.889297150567

CGTTGTCAAACTGATCCTCCAC 1.52232088016

CGTTGTCAATTCAAATCCACTA 0.538764283315

CGTTGTCCAGGTGACTGCATTG 0.605773632947

CGTTGTCTAAATGTTATAGTAG 0.443301975521

CGTTGTGCGTGAGCTATATTAA 0.484115082176

CGTTGTTTCTGTGGTGACGCTC 1.06099143165

CGTTTAGGTAGTGAAGAAATTA 0.226559053812

CGTTTATACTGTGCAGATGAAA 1.07359965704

CGTTTGATGAACCCTGTAGAAC 0.622009762257

CGTTTTGTTTTCCGGAAATTTT 0.765022919412

CTAACAGGAAGTGATGATTTTG 0.386164948732

CTAACATAATGTCAATCAATAA 0.0784863611445

CTAACATTCACTGCTGTATCAA 0.910245541132

CTAACCCCAGGTGTAGGTAGAG 1.22086517346

CTAAGAACCTACGCCGCGACAT 0.884845342849

CTAAGTCTGGCTGGTCTCATAC 0.326711375202

CTAATACCATCTGACAGAAAAA 0.267310936961

CTAATATCCCATCTAACAATAC 0.759438550264

CTAATTCATTCTCACGCTGCAC 0.335034245372

CTACAACAAAGTGCATACAGAA 0.29774350272

CTACCACGAGTTGCAACGGAAC 0.892580191017

CTACCAGTGCCTCAAAAACCAC 0.972166334196

CTACCATGGCATCATAAGGAAC 0.794054793936

CTACGCTAGAACCAAAGGAAAA 0.904968887544

CTACTTCCGTTTGCAATTCGAC 1.11824174046

CTAGATGTATCGTATTAAAAAC 0.529025993654

CTAGTTTAATCTGCTAACGAAT 0.511652395305

CTATCAGATTCGTCCTAATATC 0.343028891766

CTATCGGTATGTCCTCAATAAT 0.538592175418

CTATCTCACACAGAATGCAAAA 0.394563833261

CTATCTTTATCTGAAACTGCAG 0.429296776468

CTATTTTTCGCACCTCTGGCAT 0.740640231888

CTCAAACTGTGTCGAAGTTGAT 0.410476647292

CTCAAAGTATCGCTTTATTGAG 0.395984536323

CTCAACGTAATCCATCCGGAAT 0.408626352793

CTCAATGCCACTGCATACGGAG 0.573917208202

CTCAATGCCCGTATCGCGAGAG 0.475128415959

CTCAATGCTTTAGTTAATCCAA 0.377893473483

CTCAATTAAACTGGACTCCGAA 1.08694732158

CTCAATTCAAATGCTACTAAAA 0.539748997937

CTCAATTTATGTCAAAGACAAG 0.647597776939

CTCACAAACGGTGCCGATGAAT 0.579993548021

CTCACAAGCCCTCTATCCAAAC 0.28643956008

CTCACACAGTATTGTGCTGGTG 1.02947077224

CTCACACAGTCTGTACTGTAAT 0.671271577227

CTCACACGGAAAGCTATAGGTA 0.655276628729

CTCACACTATGTGATCCCTAAG 1.15832382388

CTCACACTGTCCGTAAGTGCAG 0.682601649206

CTCACAGAAATTGTACGTTCAC 0.482045059498

CTCACAGAATCTGAGTAGAAAC 0.55665039627

CTCACAGACTATCTTCATTTAT 0.225720236726

CTCACAGATACTCACAGGTTTA 0.323417727936

CTCACAGCAACTGCTATCCCTA 0.691250014978

CTCACAGCCTTTGAAGTAGGAC 0.838584908309

CTCACAGCGATTCGTGAGTAAC 0.499996907703

CTCACAGGGCATCATTAGGATG 0.864106401801

CTCACAGTAAGTCTTGAAACAG 0.368613822706

CTCACAGTCGGACGTACCCTTC 0.868584846752

CTCACAGTGTTGGCATCAATAA 0.250679796439

CTCACAGTTCGTGCTCGATAAG 0.714756249095

CTCACATACCCTCATAGCTAAA 0.354505340222

CTCACATAGTCTCCTTGTAAAC 0.555303705185

CTCACATATCATGATAGACATA 0.375331576503

CTCACATCCCGTTGTAATTGAC 0.53805225665

CTCACATTGACTGCTTATAGAG 0.869366254193

CTCACCAACGGTGCTAATGCAA 0.665795316105

CTCACCCAATTTCATAATGCAC 0.375241891958

CTCACCCAGTATCCTATAATAG 1.59327282764

CTCACCCTACCAGCACCCGAAC 1.29562169597

CTCACCCTGAGGACAGAGTAAG 0.442634597846

CTCACCGAAAGTCAGAGAACAG 0.275672781264

CTCACCTGAAGTCATAAGAGAC 1.00176709779

CTCACGCTAGTAGGTAACTGAC 0.697472428622

CTCACGGCAACTCACACGGTAC 0.833294682619

CTCACGGCAACTCATTCCCAAA 0.235638700342

CTCACGGCTTATGAATCAATAG 0.505204917385

CTCACGGTGTTTGGTTTGCCTC 0.651513294518

CTCACGTCAAGTCGTAAGTAAG 0.961205734557

CTCACGTGACGGCAACACACAG 0.835829340405

CTCACTCAAACCGCATAGTGAC 0.664302815477

CTCACTCACCATGATTTGTGAG 0.844155668045

CTCACTCAGTGCCGCGACGAAG 1.00343514899

CTCACTCCAACTCCAGCGCAAG 0.900120045507

CTCACTCCGAATCACTTCCGAA 0.458150316072

CTCACTCCGTAGGCTTAGGAAT 0.449436039012

CTCACTCGCGTTGGGTCAAAAG 1.08133899826

CTCACTGAACCTGTGAGGCAAT 0.507338660137

CTCACTGAATATCGGATTTATT 0.294572870514

CTCACTGAATATGATTTCAGAG 0.456755912261

CTCACTGACACTCATCCTGATC 0.573945143589

CTCACTGACAGTCTAAGGAGTT 0.839722460772

CTCACTGCAGGTCTTCCGAAAG 0.387086895151

CTCACTGCGTGTTCCGTGCAAG 0.375775932064

CTCACTGGCTGTGATTGCACAC 1.42021144864

CTCACTTAACGTCCTCGGATAC 0.459855841812

CTCACTTACGCTTAGACTCTAA 0.34443212606

CTCACTTACTTAGCTTCCTAAA 0.292053942435

CTCACTTCAACCGATGAACTAA 0.432758362421

CTCACTTTTGTAGATAAGTGAT 0.474978761994

CTCAGAATACCTGTAGATGTAC 0.152118159354

CTCAGACACCCTCTAGAAGAAG 0.376722985175

CTCAGACACTGTGCGAGAGTAC 0.169125173698

CTCAGACAGCTTGGTTAATTAT 0.488799499673

CTCAGACTTTGAGACGCGTTAA 0.384973392204

CTCAGAGAATCTCCTTAATAAA 0.397920604346

CTCAGAGAATGTTAAGAGCCTA 0.469918003441

CTCAGAGACTTGGCTAAAAGAT 0.656875657194

CTCAGAGAGATTGCAGTAGGTT 0.724063343995

CTCAGAGCAACTCCTTTGGTTC 0.648420813338

CTCAGAGCCTATCCATAGTAAG 0.500950327426

CTCAGAGCGTGTGTTTCTCGAG 0.961308086856

CTCAGAGTACTTGTTGAATTAC 0.187589755825

CTCAGATAAGCTGAGATGTTAG 0.484161123757

CTCAGATAATATGAGTTTGGAC 0.545360036542

CTCAGATAGACTGCTAGTCAAC 0.509392241724

CTCAGATCCACTCGTCGTCTAT 0.248682302488

CTCAGATCCTATGCACGTGAAG 0.250190185956

CTCAGATGGCTTGAAGAGCAAA 0.741415694697

CTCAGATTATGAGACACGTTTG 0.556702939851

CTCAGATTCATTCCTACTAAAT 0.348501777158

CTCAGCCCACGCCTACCAGTAC 0.423492974471

CTCAGCCCGTCGCATCGCGTAT 1.6425698359

CTCAGCGATGTTCTTCTTCTAG 0.476109010062

CTCAGCTTTTGTCTACAGTGAT 0.443337417694

CTCAGGCAACATGATATGGTAG 1.56129347049

CTCAGGCAGAGGCCAGAATTAT 0.485800946225

CTCAGGCCACATGCTCTATATC 1.25898012372

CTCAGGCGGCATCATGGACAAG 0.659880719857

CTCAGGGTATATGAATCTTTAA 0.383592886394

CTCAGGGTGGTTGAAAGCAGAC 1.42200664604

CTCAGTAAGCATGCAACGTCTG 0.571692536346

CTCAGTACGTCTGCGGGAGCAA 0.863638248635

CTCAGTCCTATTTAAAGATGAC 1.16409998414

CTCAGTCGGTCAGCCATTTGAC 1.38864297956

CTCAGTCTACGTACCTCACAAA 0.422634594772

CTCAGTCTCACTCGTGTTCAAT 0.363293845546

CTCAGTCTCTCTCGAAACTGTT 0.610493112065

CTCAGTCTCTGTCTAGTGGTAC 0.534914226058

CTCAGTCTGCCTGATAAGAAAA 0.424729924018

CTCAGTGAATATGAATCGTCAG 0.734181282754

CTCAGTGGGTGTGATTACAAAG 0.473681027882

CTCAGTGGTTCTGTTTACGAAC 1.71346182692

CTCAGTGTAAGGCGGATGTGAT 1.6560871493

CTCAGTGTGTTTTAAGGCCAAG 0.815068327623

CTCAGTGTTGATCCGGCGCGAG 0.606143612913

CTCAGTTAGATTGCTTGGATAG 0.742444743325

CTCAGTTGTGAGGTCTTTCCAC 1.09811599001

CTCAGTTTAGTTGATCCGGAAG 0.581646202278

CTCATACAGTCACCATATCCAC 0.669632829704

CTCATACATTCTGCTAAGGCTC 0.23218323359

CTCATACGATCTGTTATTTAAG 0.247260122718

CTCATACTCGGTCTTAACTATG 0.356413605356

CTCATAGAATATGCTTCGGAAG 1.04240261146

CTCATAGCATGTGGGTAATATG 0.436026364314

CTCATAGTTTGGGCACGGAAAG 0.386660795881

CTCATATTACCTGACTGTAAAT 0.529701856522

CTCATCGACTGTGATCCACTAA 1.09709129413

CTCATCGATTCTCGATATAGAG 0.569231642193

CTCATGCTCTGTGAAGCCATAG 0.837422202062

CTCATTCAAACTCCAGTTATAG 0.567727423769

CTCATTCTCCGACCTTGAAGAG 0.851424187526

CTCCAACAATCTGCGATATGAA 0.352412992738

CTCCAAGGAAGCCCTCCTCAAC 0.501479120546

CTCCAATTACCCCTAGAGAAAA 0.467161989193

CTCCAGCTACGTGATCCGGGAT 1.2269902809

CTCCAGTCCAATGTTATTACAC 0.631973954893

CTCCATCAGCATGATTTCGTAA 0.994719020285

CTCCCAATATGTCCGAAGTTTG 0.71692628567

CTCCCACACGATGCTTTAAAAG 0.454827901229

CTCCCACAGGGATCTTAATAAC 0.52556506542

CTCCCACGGCCCGACATCTATT 0.640145760704

CTCCCACTAACTGGTGCTGAAT 0.527163034772

CTCCCACTGACTCAGGGTTGAC 0.574713644782

CTCCCAGTAGCTCCAGGCGGAG 1.11429952269

CTCCCAGTCACGCATGCACAAT 0.1035542803

CTCCCAGTCTGCGGTAACCATT 1.28334465854

CTCCCATACTGTGCCGATGTAG 0.593737101931

CTCCCATCACGTCCAAAAATAC 1.12044660428

CTCCCATCCGGTCCTAAAACAT 1.12941020266

CTCCCATTAAGTGGTGATTCAA 0.169698642209

CTCCCATTACTGCAGAGTCGAG 0.551401735576

CTCCCATTAGGCGAAACTGTTG 0.776053037728

CTCCCATTGTCTCGTTCTCGAG 1.05315657629

CTCCCATTTTATCCTTCGGTTT 0.498921507063

CTCCCCCAATGTGTGTCTTCAT 0.707408653745

CTCCCCCTAGGTGATGACATTG 0.636725857675

CTCCCCTGTTATTCGCCCTGAC 0.907447099774

CTCCCCTTCTCTATAGTTCTAG 0.833466594607

CTCCCTCCACCCTCTGCATTTA 1.23243058826

CTCCCTCCATGCGAATCTAAAG 0.398175128783

CTCCCTCTAGCTCATGACCAAT 0.490809552647

CTCCCTCTATCTCGATCCAAAC 1.07752412288

CTCCCTCTGTGTCAATGGTAAT 1.18271537366

CTCCCTGAGTAGATAGCCCAAT 0.659248434692

CTCCCTGCAATTCTCCGATATA 0.732161109639

CTCCCTGCGCGTGCCAAGGATG 0.678718353825

CTCCCTGTAGGTCAGGATCCTC 1.31226279785

CTCCCTTAGTCTTAATAGAAAA 0.555308643143

CTCCCTTCACTTTATATTTGTC 0.477703609559

CTCCCTTGAATTTGTATAGTTT 0.535124691137

CTCCGAAACGATCTTAGAAAAC 0.517425854465

CTCCGACACGGTCCTCCAGTAG 1.10439529082

CTCCGACCGAGTCTTATCGAAG 0.46761250935

CTCCGACGCTATCAATCTTAAC 0.471462106802

CTCCGACTGTGACCTTGGTATA 0.612075166532

CTCCGAGAGTGTGGAGACCAAG 0.293588335449

CTCCGAGTCACTGACTACTAAC 1.37760096916

CTCCGAGTCGGACGACGTTTAA 1.31150208231

CTCCGATAATTTCGTAAAGATA 0.210003859693

CTCCGATCTTTGCTAAGCTCAA 0.965384582745

CTCCGCCCACTCCTAGGCGTAT 2.23015455501

CTCCGCCTCTGTGTTCCGCAAT 1.00721439867

CTCCGCGAATTTGATACTTAAA 1.02251486463

CTCCGCGATTCTGCTCCCAAAA 1.13788706887

CTCCGCTACAATGCACAAACAG 0.70689201894

CTCCGGGGGAGCGCTGTGGAAC 2.15626746817

CTCCGGTGCAGTGCTCGCCGAG 0.868955476445

CTCCGTCTCCATGCTTCTGAAC 0.768444455957

CTCCGTGAATGTGGTATTGATG 0.587329223436

CTCCGTGCCAGTCAAGTAGAAG 1.01815042311

CTCCGTGTCTGCGCTGGTCATC 2.15409884167

CTCCGTGTTCTTTTTAAGGGAA 0.41709835322

CTCCGTTACACTGGACCGGTTA 0.298155931332

CTCCGTTGTCGTGCCGACATAA 0.504264634644

CTCCGTTTAAGTCAGTATATAG 0.607965273035

CTCCGTTTCAATTATCTTTAAA 0.852358862092

CTCCGTTTCAGTCTACATGGAA 0.907478100906

CTCCTACAATATTGTCACGCAG 0.719890239598

CTCCTACGGACTCGCCCGTAAC 0.380659475651

CTCCTATCACCTCATATGTATC 0.492584912866

CTCCTCGGGAAACATATCTATT 0.607665716591

CTCCTGATCACTGGAGAGTAAG 0.366676097172

CTCCTTCGATGTTCGACACCAT 0.617319689451

CTCGAAGCATCTGAACGGAGAA 0.678972189239

CTCGAAGCGATTGCTCCCGAAG 1.08541551311

CTCGACCCATCTCATGGTCTAT 1.49559688769

CTCGACTTAGGTGCTACATTAC 0.188167890253

CTCGAGTTAAGTTATGTTTTAT 0.760546539534

CTCGATGCGTGTGCTGAAGATA 0.516087543086

CTCGCAACAGTTGATCATTAAC 0.507376460233

CTCGCACAAGCTTATAGGTAAG 0.509272812048

CTCGCACGTTGTCCATCTCAAC 0.741710981674

CTCGCAGAACCTTAAAATCCAG 0.315886360278

CTCGCAGAATGTCATTTGTAAC 0.782586818881

CTCGCAGAATGTGCTATCTCTG 0.82711979345

CTCGCAGAGTGAGCATAATATG 0.416390631114

CTCGCAGCCTCTGTTTCAGAAT 0.436289188561

CTCGCATACCGGGATTTCCCAA 0.771255032794

CTCGCATTTCCTCCCATTCATC 0.59711248731

CTCGCCGAGGCTGCAGATGAAT 1.39727644976

CTCGCGCCATGTCCTTAGTGAG 0.599107716519

CTCGCTAACTGTGCGGTCGCAC 1.36035669843

CTCGCTATCCATCATATATCTC 0.893622547923

CTCGCTCACTGTCTAGTATATC 0.459515057691

CTCGCTCATTGTCCAGAAAAAA 0.517137862584

CTCGCTCCGAGTACTCCGGAAT 0.822570202596

CTCGCTCTTTGTGCTTTCACTT 1.3579907057

CTCGCTGACTGTCGTATGTATT 0.307471603445

CTCGCTGAGCTTCATCTTGATA 0.430718552206

CTCGCTGGCGGTGGATTATAAG 0.660433859553

CTCGCTTTATTTCAACGAACAG 0.472880200789

CTCGCTTTGCGTTTTAGATAAG 1.32491838712

CTCGGAAATGATTCTGTGAATG 0.285328615764

CTCGGAAGATGTGCAATCATAT 0.556190536489

CTCGGATGGAGTGCGCTAGAAG 0.93137249923

CTCGGCCAGTCTCGTAATTGAT 1.64723334173

CTCGGCGCCTATGATTCCCTAA 0.603164090043

CTCGGCTAAGCTTAATCTTCTT 0.489112384612

CTCGGCTCACTTGCTCAGTCAA 0.563337614794

CTCGGGTTGTCTGATACGTGTT 0.242898305951

CTCGGTCCGCCCGATAACTTAG 1.31047523749

CTCGGTGCCGCATATAACCAAG 0.692146006248

CTCGGTTAGTTCGAGTGTAGAC 0.78988173242

CTCGGTTCAAGTCCTTCGGAAA 1.15735238873

CTCGGTTTATGTGGTTAAGAAG 0.388653342187

CTCGTAGACTATGCTACTTGAA 0.436425376062

CTCGTATAATCTGATAGGAATT 0.362092967895

CTCGTATGGTTTCCTAAAGTAA 0.802872835877

CTCGTCTTTTATCCTGATAGAC 0.314294678943

CTCGTTGACGTACAGCCATTAG 0.698405111141

CTCGTTGCAACTCTTTAGGCAC 0.38285002388

CTCGTTTTTTATGAAGGATAAC 0.696520990247

CTCTAACAATGTGGAAGGCAAC 1.47128624971

CTCTAAGAACCATATAGAGAAC 0.522002350011

CTCTAAGCACGTCCCTGAACAG 1.14632666092

CTCTAAGTAGCTGCTAATGATG 0.468200832226

CTCTAAGTATCGGCTGGGTAAC 0.741707287775

CTCTAATATTCTGCCTGCAAAT 0.607899300947

CTCTAATATTGTCGCCAATGTC 0.7662736839

CTCTACGGCTCTGCACCTCCAA 1.11123140924

CTCTACTTATCTTAGGAGTAAC 0.722679633783

CTCTAGCGATCTGGTTGTAAAG 0.67987925096

CTCTAGGACTCAGCTATACATA 0.501792281148

CTCTATCCTAATGTTGCAAAAC 0.707652549954

CTCTATCTATCTCAGTACCCAA 0.599252037442

CTCTATCTGACTTTTAAGCAAC 0.310931096047

CTCTATGACTAATGTCAGATAC 0.550510416904

CTCTATGCAAGTGGACACCAAG 0.526588891872

CTCTATTCATAGCATTTCTTTT 0.194412188409

CTCTCACAATGTGAATCGTTAA 0.431168744843

CTCTCACAATGTTATCTGATAG 0.52627403934

CTCTCACACTCCCCAGGGGGAC 0.590454709018

CTCTCACACTCTCATCATAATT 0.468331911027

CTCTCACAGGATCATCCGTAAG 0.520234998449

CTCTCACCATGTGATATCATAA 0.802775122624

CTCTCACCCTGTGCCTCTCAAT 1.29812809773

CTCTCACGCAGTGATGCGGAAT 0.747697794302

CTCTCACTAGTTGGTTGGTAAA 0.436918350456

CTCTCAGAATAAGATTTAATAG 0.179766999917

CTCTCAGAGTGTGCCCACGCTC 1.08954563402

CTCTCAGAGTTTGATAGCAAAT 0.499064388308

CTCTCAGCATCCGACTTGGAAC 0.508006520019

CTCTCAGCATGGCATGCACGAA 1.26980942536

CTCTCATTATCCGGCAAGTAAT 0.83227431769

CTCTCCCGATCGGGTCGTATAT 0.488303979749

CTCTCCCTGCGTTGATCCAAAT 0.773980229543

CTCTCCTTAAGTCCAGGGAGAG 0.492990768853

CTCTCCTTACATCATATACTTT 0.353021122229

CTCTCGCCCTGTGTTAAAGGTG 0.989400978961

CTCTCGCTATGACCATTGCTTT 0.438370726378

CTCTCGCTCCATGCTGCTGGAT 1.24038578845

CTCTCGGAATTTGATAAAATAG 0.538550290097

CTCTCGTATAATGTTTGGTAAG 0.610155228446

CTCTCGTTAGTTACTACTCGAA 0.51954286647

CTCTCTAGATGTGTAGAGCTAC 1.14510568636

CTCTCTCAAATCGTTGCCAAAA 0.731830505651

CTCTCTCACAGTCACGCTGTAT 0.802886144594

CTCTCTCTATATCGTTGGCAAG 0.227683168449

CTCTCTCTCCATGGTGAAAAAC 0.731985339718

CTCTCTGACAACGAAGAATAAG 0.443544891552

CTCTCTGAGTATGCTAGTATAT 0.563732879417

CTCTCTGCCAGTGTAACTGCAA 0.591427152578

CTCTCTGTGTGTTTTACGAGTG 1.04491455909

CTCTCTTAATAGGGCGCAAAAA 0.415522179776

CTCTCTTAATGTGGCCAGCCAG 0.275524444295

CTCTCTTGGAGGGTTGATGGTT 1.57069234252

CTCTCTTTACTTGCTCGGCAAA 1.04355115241

CTCTGAATTCGTTGCAATCAAA 0.862609520173

CTCTGACAGTATCAAGGGGAAA 0.313011524071

CTCTGACCATGCGCACCTTAAT 1.24105303038

CTCTGACTGCTTGCAAGAAAAC 0.844842792466

CTCTGAGACGGTTCTAAAGAAG 0.726027669606

CTCTGAGACTGGGATCGCTCAC 0.66437760416

CTCTGAGCGTGGGATGGAGGTC 0.999057528692

CTCTGAGTAAATGCTCAATAAT 0.476667634683

CTCTGAGTCGCTGTAGTGAGAT 0.698942206167

CTCTGAGTCGTTGCGCGTGAAC 0.528817134459

CTCTGAGTCTCGCGTACTTGTC 0.522728649529

CTCTGAGTGTCTGAACGATAAA 0.349008476473

CTCTGATACGATCATTCAAAAA 0.487396748321

CTCTGATATGCGGAACTTGTAA 0.45763417206

CTCTGATCATGGCGTGTCAGAG 0.934828561701

CTCTGATCGTCTCAAGTAGGAT 1.4652815374

CTCTGATGATCTGATGGTATTC 0.636454426456

CTCTGATGCCGTCCTGAGGGAC 0.651848457237

CTCTGATGCCGTGCGGGGACAT 1.07467298498

CTCTGCGCATATCATGTACAAC 0.629241149957

CTCTGCGCGTGTCGCTACTTTA 1.09271609396

CTCTGCGGCTAGTTCTGCGATG 1.71852076996

CTCTGCTGCAATTCTTAGTAAG 0.307779355719

CTCTGCTTATTTGCCGACTCAC 0.56195601884

CTCTGGCTACCTGCTCATGGTT 0.205704279856

CTCTGGGCAACTGCTGGTAAAC 0.764920547893

CTCTGGGCGAGTGATCCTCTAC 1.65761184057

CTCTGGTAAAGTCGTGGTAAAG 0.35806156188

CTCTGGTAATATCTCCCTCAAA 0.411455725609

CTCTGGTTCGATGCTAGGCAAA 0.212932540384

CTCTGTCACTATGAGTCGTAAG 0.492313531474

CTCTGTCAGTGGGCCAGTAGAG 0.568688029105

CTCTGTCTAATTAAATCGCGAG 0.628281495674

CTCTGTGAAAGTGAAGCGCAAT 0.430731827522

CTCTGTGATGCTGGAGAATATG 0.272687239991

CTCTGTGCATTTCAATCTGAAC 0.276636267072

CTCTGTGCTTCTCATAATACAG 0.875343335675

CTCTGTTAATATGTTTTGACTT 0.431077281429

CTCTGTTAGTCTGACAAACAAA 0.385915024827

CTCTGTTCTTGTGCAGGTTTAG 0.779683510311

CTCTGTTTATGAGCTTAAAAAG 0.772477526955

CTCTTACAACTCTATACCTCAA 0.366998260003

CTCTTAGGTTCTGTAGCCTTAA 1.22894772479

CTCTTATATTCTCGTGTGGCAG 1.36630487375

CTCTTATCGCGTGCAGCCATAG 0.884127169277

CTCTTCGAGGGTCGTATAATAT 0.222784382631

CTCTTCTGGTATGTTGAATTTT 0.831813890961

CTCTTGCAGCCGTAATAAAGAC 0.259277188734

CTCTTGGACTCTGCAAACTAAG 1.09183924313

CTCTTGGCCCATGCACGCTGAT 1.0901183322

CTCTTTGAACGTTAGTTATCAT 0.290381634779

CTGAAAACGTGTTGATATATAA 0.431398100866

CTGAAACTAGCTCCAGGGCAAT 0.713952367124

CTGAAAGAATCGTCTCAGATAA 0.343659384737

CTGAAAGTTGCTCTTAGTGAAA 0.71816576587

CTGAAATTACCTTACCCGGCAT 1.01368457011

CTGAACGACTCTCTGCGAGCAT 0.670974646538

CTGAAGCAAACGGGGAGCATAG 0.988573478249

CTGAAGGTCAGTCCTTAATGAG 0.455500455598

CTGAATCTATCTTATAGGCTTT 0.418806148956

CTGAATGCACATGGTACGTAAA 0.561940034433

CTGAATTAAGCTGGAGAAATGT 0.799675762017

CTGAATTTGGCTGCACGCGAAC 0.972508369852

CTGAATTTGGGACATACCGCAC 0.654999305619

CTGACAGACAATCCCCAAATAG 0.977122709608

CTGACAGGGTCCGAATAGGTAG 0.291062581603

CTGACAGTATGTGGTTCTTAAA 0.559213593828

CTGACAGTCTATCTTACGACTC 0.70741434924

CTGACAGTGTGTGATAGTGAAG 0.916375562833

CTGACAGTTTTAGAATAGCATT 0.207953199015

CTGACATGGTGTCATACCCTAG 0.24298106152

CTGACATTCTCTGGCACCTAAT 0.408977227986

CTGACCAAGTCCGTAATTAGTT 0.327083970302

CTGACCATCGCCGGTGTAGTAG 0.573218778984

CTGACCCCGTCCGATAGACAAT 0.807663545516

CTGACCGCGAGTCGTGACCATC 1.0638954376

CTGACCGTATTGTTAGAGTAAA 0.564897233756

CTGACCTAATTTGCTTACACAG 0.447860263633

CTGACCTCGTCTGAATGCAAAG 0.61754330198

CTGACCTGTTCTGATGCCAAAT 0.447027445628

CTGACGCGGTTTCACGCCTCAA 1.31166735876

CTGACGCTGCATCATGACTAAG 1.53372708525

CTGACTCCCTGTCCTGGTCTAT 0.931562781859

CTGACTCGCACTGTAATTAGAT 0.293403374758

CTGACTGCCGGGGATGCAGAAG 0.866161876565

CTGACTGGACCTCTTAAGAGAT 0.108899980907

CTGACTGGAGTTCATCTGGGAA 1.02710583891

CTGACTGGATCTGATGCAGAAG 0.317838556633

CTGACTGTGTCTCCAAACGAAG 1.32415880493

CTGACTGTTTGTGGCCCTGGAG 1.14312137928

CTGACTTAACAGGGGTAGTATT 1.07177103652

CTGACTTAACCTCTCTAGAGAC 0.2280785975

CTGACTTCGTCGGCTCGATCTT 0.802575855208

CTGACTTGAGCTGCACGCGAAA 0.977864754042

CTGACTTTAGGTCTTGTATTAC 0.378623862574

CTGAGACAAGGTCAGTGGAGAG 0.529903867347

CTGAGACAATCTGAACGATTAC 0.800030140805

CTGAGACGACCTGAAAATTCAT 0.468817591195

CTGAGACGCTCTCATTAACGAC 0.685734600511

CTGAGACTCGGTGATTAAGCAG 1.37220150979

CTGAGACTCTCTGCATAGTAAT 0.696773291879

CTGAGAGACGCCCCAGCATTAA 1.01876747025

CTGAGAGCACCTCATTACAGAA 0.254586208211

CTGAGAGGCACTGGCACATTTG 0.205434042238

CTGAGATAAAATGATCCGGAAC 0.606971601656

CTGAGATAACCTGGTTCCGTTG 0.928516519253

CTGAGCCAACGAGCTAATTCAC 0.868133425628

CTGAGCCACCCGGGGGAGGGAT 1.83689707344

CTGAGCGGCTTTGTTGTTAAAG 0.577281640325

CTGAGCGTATCTCAAAGATCTT 0.274316322225

CTGAGGTCCGCTCGTTGAGAAT 1.48844717728

CTGAGTAGAAGTCCTATGTATA 0.548762894398

CTGAGTATATCTGCAAGGTAAG 0.14109119958

CTGAGTCAAGATACTGAGTATG 0.400625418392

CTGAGTCAATGTGTTTTGATAA 0.427690878317

CTGAGTCCGGTCTAATATTTAA 0.72285697379

CTGAGTCGACTTGCCATGCGAG 1.48441712939

CTGAGTGAACCTTCTAAGTCAT 0.685115035456

CTGAGTGACGGCGACTAAGATC 0.729279008359

CTGAGTGCAACTGATAAGTAAA 0.340534357601

CTGAGTTGGAGTGCTTACATAG 1.12425589818

CTGAGTTTCTCTGTTTAATTAC 0.965033318415

CTGATACAGTGGGGTGACTAAA 0.704096327785

CTGATAGGGTGTGAACGACTTT 0.921205988778

CTGATTGAGGCTCATCGTACAT 0.577123057843

CTGATTGTATAGTGACCGTTAT 1.0410490017

CTGATTGTGTGTGTTGACAGAA 0.318155349336

CTGCAATAATGAGCCCCCTAAA 0.75892750739

CTGCAATAGCTTGCTGGTCTAG 1.45478928564

CTGCATCAGCCTGCATGTAAAG 0.622058403592

CTGCATCCATGTGATGTTGGTG 0.462062676812

CTGCATGTGTTTGCTAGTACAA 0.460623342967

CTGCATGTTGCCCCATAAGTTA 0.649444963612

CTGCCAAATTCTGCTGGCGAAT 0.803250801034

CTGCCACCGAGGCCTGCACAAA 0.809467346937

CTGCCACTGCCTGAGTTGTATC 1.97697050416

CTGCCACTGTATCATCGGCGTT 1.53403544585

CTGCCACTTTTTCCTGATCAAT 0.484097993732

CTGCCAGAAGCGGATAGCGGAA 0.951771895665

CTGCCAGAATTTCTTAGACCAA 0.548157149133

CTGCCAGCTTCTCCTTTAATAG 0.41254543208

CTGCCAGGATCTTTTATACTAC 0.368761283441

CTGCCAGTTTGCGTAACTCTAC 2.4383132794

CTGCCATAACGTGATCAATATA 0.6141296284

CTGCCATCATGTCAAAGATAAC 0.312303947111

CTGCCATGATATGAGCAATTAT 0.517248408749

CTGCCCTAAGTAGAAGTCGTAT 0.489482789493

CTGCCGCTGAATGTAACTGTAG 0.987878217947

CTGCCGGCATCCCATTAAAGTG 0.580772214599

CTGCCGTCCGTTGATGGTACAA 0.344225400188

CTGCCTCACTATGAAGGCTGAG 1.2848650065

CTGCCTCCGGGTCCCCCAGCAA 0.860816272573

CTGCCTCGCTCTGCATGTTGTG 1.81449105021

CTGCCTGACAGTGCTCGGTTAC 1.18555691422

CTGCCTGGGTGTCCGGTATTAC 1.5460243274

CTGCCTGTCCTTGCACAGATAA 0.353762367084

CTGCCTTTCTGTCCACCTGAAG 1.66247748034

CTGCCTTTGAGTCCCATAATAC 0.589373973499

CTGCCTTTGTGTGCAAAACAAG 0.50526041307

CTGCGACTGAGTGATGACTAAA 0.419067916297

CTGCGAGAGTCTGATGCATATT 0.504450372957

CTGCGAGTAAGTGCAAGTCGAA 0.571232916538

CTGCGAGTGTATCATTCTCCAT 1.83435422245

CTGCGATAATGTCAATGGGAAC 0.386612411389

CTGCGCTGGTCTGCTCTTGCAT 1.97507951244

CTGCGCTTCACCGACTAATTAG 0.392918893622

CTGCGGCAATTTCCTTATGAAT 0.482853039726

CTGCGGCCCTATTCTTGCAAAT 0.851596729503

CTGCGGGGATATCCGGTTATAG 0.163641325279

CTGCGTCAAAGTCCATTCAAAG 0.692989183616

CTGCGTCAATCCCAAACATAAA 0.524692865518

CTGCGTCACGAATCACTTCTAA 0.587523081158

CTGCGTGAATCTCCAATATTAT 0.43655386465

CTGCGTGATAGTGGTGGCCCAG 0.245265066688

CTGCGTGCATATGATACGGTAG 0.725297547465

CTGCGTGCCGATGACATTGGAA 0.783163526613

CTGCGTGTGTGTGTGGGCTTTG 1.0794254511

CTGCGTTAATCTCTCGACATAT 0.442546007127

CTGCGTTGAGGTGTTGACTGTA 1.17423002618

CTGCGTTTGATTGCAGATACAC 0.35186802279

CTGCTACGTTCTGGACGATTAA 0.822723255195

CTGCTCGCATGTTCTTCATAAT 0.808095167615

CTGCTTTAATGAACTGTTACAA 0.579394815837

CTGGAAATGGGGGAATATTTAA 0.34964629721

CTGGAACGGTTAGATTGTATAC 1.07776677526

CTGGAAGAAGTTGAAAGTTAAC 0.767689493766

CTGGACCCGGATCATCAATATC 0.21526823143

CTGGATCCCACTTCACCAGTTA 0.170192732543

CTGGATCGGTATGAACAGTCAT 1.03051894724

CTGGCACACCATCATAATAAAG 0.721525698309

CTGGCACACTAGCTAGCGGTAT 0.726699469686

CTGGCACAGAGGGGTGAAGCAC 0.867121862692

CTGGCACTATCACGTAGGAAAA 0.438384658355

CTGGCACTCACTCATCCTGTAC 0.271610689826

CTGGCAGGACATGCTTAGAGAC 0.570851318573

CTGGCTCAGAGTGCACTTGTAT 0.346433818761

CTGGCTTCATCTCATTTGGTTC 1.06906290932

CTGGCTTGGTGTGATAAGAGAT 1.05394923231

CTGGGACTGAATCCAAGAATAG 0.608731049735

CTGGGAGTCGTTTGTAGCCAAT 0.773655088926

CTGGGAGTCTCTGCAAACCAAT 1.07401534552

CTGGGATTCATTGGGAATAAAG 0.336204697416

CTGGGCGTACCGGGTGTCAAAC 0.13753385629

CTGGGCGTATTTGATAATGCTG 0.305143723403

CTGGGGGTATCTGTCTTCGTAG 1.28377598303

CTGGGGGTCTCTCTTTCTAATA 0.404351198357

CTGGGGGTTGGTCGTTTCATAG 1.30364911164

CTGGGTACACGTCGAGTCGGTC 1.35360234374

CTGGGTCAATCTCCTTCGACAT 0.997224235091

CTGGGTCAGTGTTCAGTACATT 0.756643675235

CTGGGTGTATGTGTTAAACCAA 0.902192827859

CTGGTAAGATCTCGTTGGGAAG 1.24634468711

CTGGTACTATATCCATAGGGAT 1.06117794972

CTGGTACTTTCTTAAAAGTAAG 0.520398838388

CTGGTTGAGGGTGCTCCCTGAC 1.53743678338

CTGTAAGAGTCTGTTCGTCATG 0.768794007376

CTGTAATTGTGTGACGTTGTTC 0.781514102403

CTGTACGGAATTCAAGTAAGAA 0.308135181107

CTGTACTGCCTTGAAGGGCCTC 1.22038440536

CTGTAGTCTAATGTTGGAATAC 0.45144725512

CTGTATCGAAGTGCTTATACAG 0.209235906511

CTGTATGAAGCTGCTTAAAAAA 0.62095382613

CTGTATGCGAGTGAGACTACAT 0.211760243896

CTGTATGTTTTTGCTAAGTTAA 0.313534899808

CTGTATTAAATAGATAGACTAA 0.0799115314458

CTGTATTCATCTGCATACATAT 0.719937413753

CTGTATTCCAGTGACAATAGAG 0.542751295025

CTGTCACAACTTCGCACTGTAT 1.26494193628

CTGTCACACCGTCCTTTTGTAT 1.01845413619

CTGTCACCCAGTTGTCATAAAT 0.607255678901

CTGTCACCGCGTCCGATTCTAT 1.34718084835

CTGTCACTACTTTAATCTGAAC 0.311888255759

CTGTCACTAGTTGCAAACTAAG 0.613350290925

CTGTCACTCTATGCCTCCTAGA 0.410880078541

CTGTCACTCTGTGCTTGCTGAA 0.794755782774

CTGTCACTTTGTGCCCGCTGAG 0.978419858519

CTGTCAGAAAGTTACTAAACAC 0.769447598185

CTGTCAGAAGGTGCATACTAAA 0.384949815187

CTGTCAGCATGTTGCCATCAAT 2.03616253805

CTGTCAGTATCTCTCTAGGCAG 0.647607792695

CTGTCATAATAGCGTGGGGGAC 0.692449665117

CTGTCATCCTGTCGAGTGAGAG 0.670701363985

CTGTCATCGTCCTTCGGTTGTG 1.50197531115

CTGTCATCGTCTCCTCAATTAC 0.863330723219

CTGTCATTGGGTCCGACAGGAT 1.47026726737

CTGTCCCGAGCAGACTGGTGTA 1.64789601682

CTGTCCGTCTGGCCAGGTCAAT 1.13040794007

CTGTCTCAAAGTTGGCATAAAA 0.38833468785

CTGTCTCCATCTCAGGTATGAG 0.26069321658

CTGTCTCGAGTTGATGATCGAT 1.67891664458

CTGTCTCTAGTTGCTGAGGCAC 1.32181675971

CTGTCTCTTACTGGTACTGAAA 0.73945406023

CTGTCTGTGGAATCGGTAGCTG 0.378466473954

CTGTCTTCCGCTCCATCCTAAT 0.933256453121

CTGTGACAACATGACGTGAAAC 0.389335454099

CTGTGACCAGCTCATCCTCGAG 1.3662055699

CTGTGACGCTCTCTGCCGGAAT 0.699989229818

CTGTGACTCGATCCAAACCAAT 0.621478515466

CTGTGACTGACCCATGTTAAAT 0.804954504473

CTGTGACTGTCTCCTTGTGCTC 0.86271502416

CTGTGAGACCCTGGACGTAATT 0.625130283502

CTGTGAGAGGTCGGCTACGTAA 0.969301673456

CTGTGAGAGTGTTCTTGCGGAG 0.378358391935

CTGTGAGATGCTGCTTACGTAG 0.668176315928

CTGTGAGCCTGTGCTTTGCTTC 0.951330366559

CTGTGAGTTTCTGCTAGTCAAT 0.401184426079

CTGTGATACGGTCCTGGGCGAA 0.673104985792

CTGTGATTACGTCATTGACCAG 0.415991203289

CTGTGCCATATGCATAAAAAAG 0.232576429636

CTGTGCGAGATTGGATGCGTAC 0.820685818824

CTGTGCGTTTGTCAAAGCAGAG 0.727364655425

CTGTGCTGTCCTGGTTAAAAAG 0.86449872817

CTGTGGGTCCACGGTGAGGAAG 1.46468882705

CTGTGGTAACGTCTTGTCCGAA 1.19755393351

CTGTGGTGCGCTCATGTGTGAG 1.2364839662

CTGTGGTTGTGGCTGTAAATAA 0.77307848582

CTGTGTAAATCTCCAACATTAG 0.688983860229

CTGTGTCAGTGTGGTTTGAGAT 0.3543869793

CTGTGTCCCACTGTTTAGAAAT 0.191094385527

CTGTGTCTAAGTCATGAGGAAG 1.18210910337

CTGTGTGCATTTGCGATCGCTG 0.824586434943

CTGTGTGCTTATGCACAACAAC 0.337617663561

CTGTGTTAATCTTATTGTTAAT 0.434389144725

CTGTGTTAGTCTGGTGTATTTT 0.250810754989

CTGTGTTCTCCTGGACAGCGAT 0.589193710999

CTGTGTTTCCATTCTAGCTTAG 0.794804808526

CTGTTAGAATGAGCATGGGATG 1.00371116571

CTGTTAGTATACGAATTGTAAC 0.480102984278

CTGTTATTGACTCAAGACCAAC 0.638309970275

CTGTTATTGAGTGCAGTATAAC 0.450405098456

CTGTTCGAGAGTGGAGCTTAAT 0.183454881049

CTGTTGGCGCCTGATGTCGTAG 0.47514001082

CTGTTTCACCGCGTTAAGGTAA 0.742824134092

CTGTTTCTGTTTCACGATGTAA 1.2130582521

CTGTTTGCATCTCCTGGTTAAG 1.5975098453

CTGTTTTTGTGTGTATGGTCAC 1.03178825998

CTTAAACCATGAGTCTTGTTAG 0.470515736403

CTTAAATCACCGGAAATAAAAC 0.322595378204

CTTAAATTCTGTGATACCTAAG 0.476998123967

CTTAATCACGGTGGAGGTTCAA 0.101083408509

CTTACACACTGCGCACGTAAAA 0.833249764893

CTTACACTAAGTCACTTGAGAG 0.295640957167

CTTACAGACGCTTCAATCACAA 0.379477212406

CTTACAGCAACTGGATTTGAAG 0.747457531104

CTTACATAGGCTCCTGATGAAA 0.901185138661

CTTACATTAACTCATGGCCCAG 0.494318240576

CTTACCCTCCCTGCTACAAAAA 0.2241029695

CTTACCTCCACTGTTTACCCAG 0.340172346327

CTTACTCCCTAACGCATCGTAT 0.58279455324

CTTACTGAATGATATGCGTGTG 0.739282695183

CTTACTGTTTCTGATTGCCAAA 0.614487278424

CTTAGACCGTATGTTAGGGATA 0.855429454276

CTTAGACCTTGTGCTGTGCAAC 0.868412624933

CTTAGAGCGTGTCGCCCTTAAG 1.18302935338

CTTAGATTAAGTGTTACAGTTA 0.199692773481

CTTAGCCTTAGTCCACAAATAC 0.562461441128

CTTAGCGCAATTTTAAATTCAG 0.339302800827

CTTAGGCTGTCGGATAGATAAA 0.631946380664

CTTAGGTTCTCACTTGGTCCAT 1.14500185686

CTTAGTCCCGATCATCACAGAG 0.267636657658

CTTAGTGACCTTGATAGCGTAC 0.616307485306

CTTAGTTAAGCTGCCCTATTAT 0.388580263713

CTTAGTTACACGGACATACTAG 0.322778061416

CTTAGTTTCTTGCGGTCCTATC 0.948925183523

CTTATGTGACCTCAACGTAATC 0.55941532753

CTTATTTACGCTCTTCAGACAA 0.355829968017

CTTCATGGATATCTAACAGAAG 0.403145329095

CTTCCACAACGGCATCGGGCAG 0.500629807666

CTTCCATCTTGTGGTCTTTAAT 0.556450072664

CTTCCCCTATGGGGTTCCGGTC 0.960090372405

CTTCCTGGAGCTGTAATTTCAT 0.343299041937

CTTCGACTAACTCTTTGCCGAG 0.838157285558

CTTCGAGCCTCAGATTTTGGAG 0.48077139094

CTTCGAGCGTTTGCTTATACAG 1.13718177296

CTTCGAGTCTCTCATTCCATAT 0.560920436148

CTTCGATTGCCACAATGCCGAC 0.972327092991

CTTCGCGACTATGTGACGGCTG 0.264665126044

CTTCGCTAGAATGCCGATATTA 0.506873609079

CTTCGTCCAAGTAGTAGAGGAA 0.617493803616

CTTCGTTATGGTGCTTCAGCAC 1.14485519163

CTTCGTTCCTGGGTTAGCGAAC 1.06203952543

CTTCGTTTCGTTGACAGGGAAC 1.06405773348

CTTCTACAACTTCCTTCAGTAC 0.749888977365

CTTGATTAACTTTGTACAGAAC 0.280940579047

CTTGCCGCATGTATTGGCTGTC 2.51249974936

CTTGCGCTAGATGCGGACAGAT 0.984377328389

CTTGCTCGCTCTGACCGATCAC 0.292161592687

CTTGCTTACAGTGATCAAAAAG 0.554845768153

CTTGCTTGATCTGATTGTGGAG 0.901493990132

CTTGGACAGACTCGTGAGCTAT 0.59203775519

CTTGGACAGAGTGAATACAAAC 0.377249923349

CTTGGAGAATTTCATCATCGAA 0.262772164361

CTTGGAGATAATGTTAGTAAAA 0.589881352906

CTTGGATTGGGTGACCACATTT 1.05793877122

CTTGGCCTACATCCATTGGAAG 0.723731841553

CTTGGTCTGTCGCAAGAGAGAA 0.329555044531

CTTGGTGCCAGTAATCGAAAAA 0.624542810112

CTTGGTGCTTATGGTGGTAAAC 0.333304830067

CTTGGTTGATTTGATGACCAAT 0.640455471266

CTTGTCGCAACTTTTTAAGCTC 0.909344621414

CTTTAATAGGGCCATCAGATTG 0.601995664221

CTTTAATTTTTTGATGCTCAAG 0.294113920705

CTTTACTAGACTGACTATGTTT 0.353227323875

CTTTATTTGTGTTCATTCAAAG 0.479157891169

CTTTCAAAATATGGTCTTATAC 0.291872031636

CTTTCACAAGCTTGTTACTAAC 0.492317643578

CTTTCAGTCTCTGTATATGTAG 0.236230807368

CTTTCATTACGCGCTCGTAGAG 0.742723744927

CTTTCATTCAGTTTATGCTAAG 0.886095633126

CTTTCCGACTTGGATAAAGATT 0.390790091464

CTTTCCGCACATGAGCAAAGAT 0.707428962821

CTTTCCGTATCTGACACCGGAC 0.672478507868

CTTTCCTTGTGTGTTCCAAAAG 0.314413207257

CTTTCGGACTATCAAGGATGAG 0.683552891741

CTTTCTCGCTCTGATCGATATA 0.335218466826

CTTTCTCGGCGCGCTCCCTTTA 0.986462357128

CTTTCTGCCCCAGCTGCAAAAG 2.80968937217

CTTTCTTTCTCTCCTTAGGTAC 0.557058826307

CTTTGAAACGATCCACCCAGTC 1.32932085869

CTTTGAACATAACTAGCGAAAC 0.235420643224

CTTTGACGGATCGTTGATCCAA 1.0230099828

CTTTGACTGCATCATAATCAAT 0.178812599502

CTTTGAGAACATTAGACTGAAA 0.368553044077

CTTTGAGCAAGTGCTCGGGCAA 2.08521934218

CTTTGAGGAGTACTTGATACAT 0.679593396613

CTTTGATCGTTTGGGTTGTTAA 0.714507117269

CTTTGATCTATTGTTGGCGAAC 0.480925939069

CTTTGATGCGCTCAAGAAGGAG 0.538263354197

CTTTGCCTGCCTCAGAGAGTAT 0.647928896829

CTTTGCTCCTAAGTTGACCAAC 0.873930155001

CTTTGGCCTTCTGCAAAAATTA 0.834263394574

CTTTGTCCATCTCTTCAAGTAA 0.782351376904

CTTTGTCCGACTGTTTCATAAT 0.26399572546

CTTTGTGAATCTGGTCACGGAT 0.357301547033

CTTTGTGATAAAGATTTAACAA 0.514170226896

CTTTGTGCATGTGATTGTCCAG 1.0040162777

CTTTGTGTGGGTCAACGATTAT 0.501093329907

CTTTGTTCGGTTTTAAAACTAT 0.256742475741

CTTTTACACTCTCTTGATTGGG 0.617933403452

CTTTTGTTCGCTGCAGGCTAAC 0.812176422204

GAAACAGAAGGTTACCGCTAAG 0.745265151243

GAAACTATCTGACATGACGTAT 0.494504709038

GAAAGAGAGGGTGCATACTAAC 0.521207391831

GAAATATTCTGGCCACGCTTTC 1.02834646337

GAACCAATCTCTCATGTAAAAA 0.114349497382

GAACGAGACATTGACTTATAAA 0.261716323645

GAACGAGTATGTCCTGTGCGAG 0.543558262246

GAACGTGTATCCCTTCAATTAT 0.452054813342

GAAGCCGAAACTGATCGATAAG 0.58708970373

GAAGCTGCCAAGTGAACCCAAA 0.686195076943

GAAGGGGGTTGTTAAGGTATAG 1.35486995458

GAAGTCTTACATTGAAATAAAT 0.51615659744

GAATCACGAGGTCCAGGCACAA 0.464425883286

GAATCATAGAATCAGCGTTCAC 0.536023427043

GAATCGTTAGTTGATGAAAGTT 0.520773297291

GAATCTTAAAGTCATTAGGTAA 0.480626581613

GAATGATAGAGTCCTGCGTTTC 0.488521146155

GAATTACAGAATGTTGTCTTAT 0.32435730183

GAATTAGAACCCGGATTGGAAA 0.385170184027

GACAAACAAGGGGTTATTTAAG 0.302924141363

GACAAACGAAATGATCGATGTC 0.603910026705

GACAAAGAGCCTGTTTACTGTA 0.983347969315

GACAAAGGTATTGCATCCGCTA 0.553608531918

GACAAAGTGACTTTTGTCCTTT 0.298646683899

GACAAATGGACTCCTGAAGGAC 0.637088259516

GACAACGCATCTGATTAACTAC 0.310531207368

GACAAGGCAGTGGATGGGGAAA 1.42232954352

GACAATCAACGTGCACGGCTAA 0.765196555135

GACAATCCGAGTCCCAGCTATC 1.18001440674

GACAATTAATGTGGTGCGTGAT 0.330895070554

GACAATTACTCTTAAGCGAAAG 0.127851669838

GACAATTGCTATCCTTGAAAAC 0.698230383578

GACAATTTGGGACAAACGGTTA 0.219804424134

GACACACACCGGGCTGCGAGAC 0.608977737917

GACACACAGTCTCCTGGCTGAA 0.26861577221

GACACACCCACTGATCGGAGAG 0.342510637642

GACACACGAACTGCATGGAATT 0.773464147222

GACACACGATATGATGATATAA 0.44836018416

GACACACTAAGTCCGTCCTATC 0.654600135314

GACACAGACGCTCCTATCGCTC 0.565394593808

GACACAGCCCCTGCAGCCCAAC 0.356972133359

GACACAGTCACTCAAACACAAG 0.125036447177

GACACAGTCTCTGCATTGAGAT 0.37924401007

GACACAGTGCCGGCGCAACGTC 0.665280741048

GACACATAATCTGGTTGGATAG 0.0881015954699

GACACATAGTGACAACCTATAA 0.386084739916

GACACATTAACTCCGTGGGAAG 0.136244792809

GACACATTAGGTCATCATATTC 0.549575220727

GACACATTATATGAGTACGAAG 0.686741927736

GACACATTGGTTCCTGTAGTAA 0.298420612829

GACACCCGATGTCATTCCTTAC 0.713239873483

GACACCGAGGAGGATCAACCTA 0.626757207682

GACACCTTCGGTTAAACTGGAC 0.614986770297

GACACCTTTAGCCGATCCGAAA 0.670283195012

GACACGCTGAGTTCTAGGGAAC 0.483464865422

GACACGGCCCCTGCTTCAAGAC 0.396126868465

GACACGTCGACTTATCAGTCAG 0.249688912883

GACACGTTCCCTCTATGTCAAG 0.954109596899

GACACTCTAACTCATAAGGTAA 0.449467002796

GACACTCTAGCTGATATGGAAG 0.178167061391

GACACTGAAGCTGATGTACTAA 0.492923069473

GACACTGACCCTCAGGTCTTTG 0.511661384776

GACACTGACGTTGCAAACTATG 0.326748044409

GACACTGACTGTGCAATCGGAT 1.07005308207

GACACTGTCGGTCGTGTATTTG 0.567552673898

GACACTTAACGTCTCTTAAAAT 0.271313299171

GACACTTCGCCTGAAGTTTCAA 0.236184280744

GACACTTGACGTGTTATTAAAC 0.417242472326

GACACTTTCTATGTTGCATTAC 0.55638159833

GACAGAAGACGTAATCTCGTAT 0.236297672524

GACAGAATCTTTCAAAGCCAAC 0.275664762088

GACAGACAAAGTCATTTGGTAG 0.462175881127

GACAGACAGACTTCCTACGTAG 0.810339102339

GACAGACAGTGCCGTCGATAAT 0.933484612723

GACAGACTAGGGCTATTAGAAA 0.713824446579

GACAGACTATCTCGATCGAAAC 0.368680841542

GACAGACTATTTGGTTACCAAC 0.663914667498

GACAGACTTTCTGAAACAAGAT 0.24172959201

GACAGAGAATATCTCTGTACAA 0.420963029078

GACAGAGAATCTGACAAGAAAG 0.526305798368

GACAGAGCACCTCCTCAACCAG 0.319538824654

GACAGAGTACTTCTTTACGATT 0.521359589378

GACAGAGTTGACGCAGCGCAAT 0.79011516425

GACAGATAATCTCATTAACTAG 0.198208179058

GACAGATAATCTGCTATGTTTA 0.389221004281

GACAGATCACCTGTCTGGAAAT 0.531305956898

GACAGATGGCGTTATACGTCAA 0.833709892635

GACAGATTATGGGATCACCGAC 0.83446549428

GACAGATTCTATGCAAAGGTAC 0.576020454787

GACAGCCCCGTTCCCCCTCAAC 1.29146045996

GACAGCGTATCTGACGGACAAT 1.09460528259

GACAGCTTACCTTCTGGCTAAA 0.478685092693

GACAGGCATTATGTTCCAACAT 0.149737826997

GACAGGGATTGTCATTACAAAA 0.43754849715

GACAGGGTGTCAGCTACGGAAG 1.37692112894

GACAGGTGTTGTCGTCCCAAAG 1.03309987265

GACAGGTTGTGTCCAAACTAAA 0.425300961146

GACAGTATTATTCACAGTAGAC 0.386274674253

GACAGTCAATCTGCTCAATCTA 0.675794993259

GACAGTCCAGATCATTATTGAG 0.475994144957

GACAGTCCTGGTTAGAACGGAG 0.421042066206

GACAGTCTCGCACCCCGACTAC 0.784601145128

GACAGTCTGATTTGCGACTAAA 1.03617199231

GACAGTGAACGACAGAACGAAC 0.587635967395

GACAGTGAATCTCGATGCTTTT 0.65735699608

GACAGTGAATGTGGTAGCCCAT 0.436035235465

GACAGTGCCCGTGATTACTAAT 0.720301531184

GACAGTGCGGTCCAGTTCTAAA 0.682450847534

GACAGTGGAACTCATGGGCAAG 0.553971729726

GACAGTGGCCATCTAATGGTAT 0.562112623562

GACAGTGGGAGTGCTGACAAAC 0.750949798769

GACAGTTAAGCTTATTCGTTAC 0.255484933058

GACAGTTAATGTGGTTGCATAT 0.560756798504

GACAGTTAGCGTCACCACGAAG 0.963186661511

GACAGTTGAAGAGCGTGCTGAT 0.552205870033

GACAGTTTATGTGCCGATCAAA 0.825259278703

GACAGTTTCGCTCCTTTTACAT 0.40267763812

GACATACAATCTGGTATTCCAG 0.742352562034

GACATACGTGCTGGTCGTTAAC 0.471972377547

GACATAGTATCTGGAGCACATG 0.901421203909

GACATAGTGCTTGATGCGTAAG 0.442400297619

GACATAGTTCTTTTATATTTAG 0.346454056668

GACATATACTTTGATGGACATC 0.396660011015

GACATATTATCTGAGACAAAAC 0.344119729561

GACATCCAGTCGTATGGAGATA 0.682050710497

GACATCCGAATTGTTATCAATA 0.580007738355

GACATCGCATGTCTGTTAGAAC 0.784432781102

GACATCTTGTCTGGGAGGAAAG 0.314264238908

GACATGCTAGCGCAATTAAAAA 1.02065403583

GACATGGATTCTGGAATACGAA 0.718232614177

GACATGTACTTTTTAACAGTAC 0.761232597887

GACATTCAGTGTGATTTAGTAT 0.327128234643

GACATTCATTGTCGTAAACATC 0.237373783894

GACATTGAAGGCCGAGGGAAAG 1.40874299361

GACCAACAGTGTGGATGTGCAG 0.760252110318

GACCAACCATCATATAAGAGAG 0.493245673927

GACCAACTAGCTCAAATAGTAC 0.43299399998

GACCACTAACGAGGAACCGCAA 0.916311412145

GACCAGCGAAGTGATGTGAAAC 0.920035874566

GACCAGGAAAGTCCATGGCAAT 0.606211800305

GACCAGGCACTTGCCTAAATAG 0.831075083264

GACCCAAGATCTGATTAACAAC 0.755292070258

GACCCACAATGAGTAAGATGTA 0.319196883749

GACCCACAGTCTGCTAGTACAA 0.436960973931

GACCCACGGAGTCATTCCTATT 0.572072802011

GACCCACTAGGTCAATAATATA 0.643374793376

GACCCAGTTTCTCATCAAACTG 0.420035400971

GACCCATCGAGTCTTAACTCAG 0.39134677622

GACCCATGATGTGCAAGGAGAC 0.60670987961

GACCCATGCCGTGTTGGGTCAC 1.36362778877

GACCCATTCTATTAAGGCTCAA 0.559507615751

GACCCATTGCCTGTATTTGATG 0.455500125444

GACCCCGATTCTCCTGTGCCTA 0.641249855306

GACCCCTCTGCTCTTGGGACAG 0.376555711349

GACCCCTTCTTGCGCCATAGAG 1.26268335203

GACCCGGCCTCGCTTGCGAGTT 2.05201819359

GACCCGGTCGGTCCATGTATAT 0.702366334981

GACCCGTTATCAGCTATCACAG 0.689330088296

GACCCTCAACGTGCTATAGAAA 0.374652706706

GACCCTCACACTGTTTTTCATG 0.97215277657

GACCCTCATTCTCCTACACATG 0.503908667308

GACCCTCTAGGTTAAGTTTAAA 0.480917037682

GACCCTGAATCTCACGACTTTG 0.497646642113

GACCCTGGCCACGTTTGGGTTT 0.63691225523

GACCCTGTCGATGACAAGAAAA 0.191664262459

GACCCTGTCTATCACATGAGAC 0.368676578632

GACCCTTAAACCCATTGCGAAC 0.993155661987

GACCCTTACGGTGGGGTGCATA 1.24141471329

GACCCTTTAGCTTTTCGACTAA 0.528679198704

GACCCTTTATCTGAAAACCTAC 0.604733289871

GACCGACTATCCGTTGGCGAAA 1.15043288257

GACCGACTATCTTCAAGTGAAT 0.302828311021

GACCGACTGTTTGGTTCTGGTG 0.898658821625

GACCGAGACTCTCACCCACTAC 0.189215430169

GACCGAGAGTCGCAAAATGGAA 1.01661618817

GACCGAGATGGTTTAAAGATAG 0.548684232153

GACCGAGCCTCCCAATCCAAAT 0.639177314622

GACCGAGGGGTTCCATACCCAG 0.730028525701

GACCGATAGCGTGAAACGCGAG 0.951041681959

GACCGATATTCTGAAATGAGAC 0.666966863605

GACCGATTAACTCCTCGGTAAT 0.644561945954

GACCGATTATCTCGAATTAAAA 0.147631193123

GACCGATTGTTTGCTATCGCAT 0.30410711176

GACCGCCATTTTTCTTCACAAC 0.472048782001

GACCGGGAAGACCAGGAGTTAA 0.302126481585

GACCGGGGATCCCTTTAATAAG 0.354296491255

GACCGGTAAACAGCGTAGTGAC 1.10543666844

GACCGGTGAACAGCTGCGTAAT 0.315905489365

GACCGTCCCAAGGAATCTGTAC 0.44942366063

GACCGTCCCTCTGCAAAGTGAA 1.00122011612

GACCGTGAGAGTCACGTCAGAG 0.802031562077

GACCGTGCCAGTCCTGAAATAT 0.875656541809

GACCGTGCCTGTGGTTTTGTAT 0.784775632316

GACCGTGTCGCTGGAGACCTTC 0.67718329416

GACCGTTAATCTCCCCGTGTAC 0.231342318785

GACCGTTCACCACATGTTTATT 0.409594136531

GACCTAGTATGTGAGTAGGCTA 1.64930858207

GACCTCTCGACTCTAATAGCAG 0.436254939584

GACCTTGAGTCCGTTTACGTAC 0.177194105794

GACCTTGATTCGGAACGAAGTC 0.886386223914

GACGATGCTTGTGATCATCGTA 1.11262535491

GACGATTGATGTCCTACTGAAT 0.594266878007

GACGCACACACACCCTCTTTAC 0.529099312685

GACGCACAGGCTGGTCTTCAAT 0.47017845669

GACGCACCAATTGCCTAATAAT 0.320950005659

GACGCAGAAAGGCATGAGAATA 0.499777562906

GACGCAGACAGTGCTGTGCCAT 1.21545120933

GACGCAGGGATTCTATATTTAG 0.280346799278

GACGCAGTAACTGAATTCTTAG 0.636999104919

GACGCAGTAGTGGTGTAATAAA 0.608178133355

GACGCATTATCTGTGCAAGAAG 0.272326169011

GACGCTCAGATTTTTAAGGTAA 0.300319581286

GACGCTGAATCGCTTTTGTAAT 0.546333492803

GACGCTGCAGGTGCAAGAATAT 1.96997837356

GACGCTGTAACTTCATACAGAG 0.65140940319

GACGCTGTCCCGTCAAATTAAC 0.561460979904

GACGCTGTCTCCGGTAGTTCAA 0.136318078377

GACGCTTCGTTTTTAACGCGAG 0.61874766293

GACGGACAACTTTCGAACCAAA 0.662802511406

GACGGACATCTTGTAGAGATAA 0.465174330121

GACGGACTAACATATGGTTATT 0.320654510373

GACGGAGACACTGGTCGGTAAC 0.643986960375

GACGGCTTACCTCATGACCAAC 0.496995192555

GACGGTGAAGCTGTTGGGAATG 0.858251146629

GACGGTGGAAGTGTTCAATTAA 0.321784068362

GACGGTGGCTGTGATGCGCAAT 1.95221924534

GACGGTTTAGCTCGTCTCTAAG 0.812711506195

GACGTACTCTGTCAGCAATATC 0.504982062058

GACGTACTGAGTCATAGGAATA 0.619997341582

GACGTCGTCAGTGGAAACCTAG 0.417464721251

GACGTTCACGGTCATACCAAAG 0.37976764339

GACGTTGAACATGCTATTCTAT 0.554809793163

GACTAACCGGTTCATTGCATAT 0.438174727696

GACTAACGTGTTGCTTGATAAC 0.328451915815

GACTAAGACTATGCCGTACTAT 0.112614058998

GACTAAGGATCTGATAATGAAA 0.367259070727

GACTAAGGTGTACAAGTGCATA 0.650765158297

GACTAAGTATCTGGTTCGTGAG 0.392179065421

GACTAATAACGTCCTATTTCAC 0.32752250962

GACTAATACGCTGAATCGTAAG 0.532275008382

GACTAATAGTGGGTTCATTTAA 0.326401603902

GACTAATTGAATTCACCTAGAT 1.51767383759

GACTAATTGGTTGATTCTTGAC 0.568615961084

GACTACTTATCTGAAGGAAAAA 0.575889615203

GACTAGCCCTGTGAAAAATGAC 0.71058376219

GACTATGCTCCTGTTGGGAAAG 0.614702400495

GACTATGTGACTTCGCATTCAC 0.791207510975

GACTCAACGTTTCCTTGAAATC 0.914420772395

GACTCACAGTGTCATAAGATTC 0.202440753453

GACTCACCAGGTGATAATGAAG 0.533821490495

GACTCACCCTCTCGTGGCTGAG 0.644606294166

GACTCACCGCCTCTTTATTGTC 0.618968248361

GACTCACTAATTGATTTTGTAG 0.442333604419

GACTCACTGACTGTACCAGTAG 0.268264028926

GACTCACTTTATGCATTATTTC 0.558534162069

GACTCAGAACATACTAACAAAC 0.315392288274

GACTCAGTATCAGCAAATCTAG 0.451906801669

GACTCAGTCTGTCTAGTAAAAT 0.369979089455

GACTCAGTGCGACAACAAGGAG 0.64166956591

GACTCATAAACTTCATTAGAAG 0.159431235924

GACTCATCCCTTGGAAGCAAAG 0.588216244774

GACTCATTACAAGACTCTCAAC 0.548591274258

GACTCATTATCTGTACGTTGAC 0.666790300108

GACTCATTATGTAATCACTTTT 0.253312216112

GACTCCCACCCGGAAGCCCAAC 0.771573866231

GACTCCCCTTATGCTCACACAA 0.677582606197

GACTCCGAATGTGGCGATGGAG 0.942275902988

GACTCCGATGATCCATGGTAAG 0.467473772666

GACTCCGCACTTCCTGTTTAAA 0.665999264526

GACTCCGCCGCTGCAGGTCAAG 3.13527265906

GACTCCGGGGATGTTCAGATTT 1.3843909556

GACTCCGTTTTTCCTGAGTAAA 0.930507947916

GACTCCTTAACTGCCAACCTTT 0.478606680727

GACTCGGAGCTTGTGGACCAAG 1.2540028936

GACTCGGCGACTCAGTAGTTAG 1.39181518131

GACTCGGTAGTTCCTACTTGTA 0.282590527123

GACTCGGTATATGTACAAGTTT 0.536759043913

GACTCTCAACTTGCAAGCTTAG 0.604667698042

GACTCTCCATCTCATTTCAATC 0.463014566141

GACTCTGAATGTCCTATGGATG 0.38025991486

GACTCTGAGAGTGAGTTTTTAG 0.862672137505

GACTCTGAGCCTCAATACTCTG 0.437037918349

GACTCTGATTTTCTCTGGTTAA 0.237375844067

GACTCTGCAGGTCATGTTTGTT 0.350808513371

GACTCTGCCAGTCGAGTGTAAT 0.753538472346

GACTCTGGATGTCAATGGCATG 1.02413699723

GACTCTGTAGGTGCTGTGGAAG 0.341402237658

GACTCTGTGTGCGATGAAGAAC 0.171799811151

GACTCTTCGACGCCTAACTAAC 0.474350647341

GACTCTTCGTGTCAAAGCATAG 0.916378819419

GACTCTTTGAGACCTAGTGAAG 0.373042241706

GACTGACATTTAGCTGGACTAG 0.289606516231

GACTGACCATCTGCTACAAAAG 0.244550361379

GACTGACTCGTTGCAGAAGAAA 0.660189543678

GACTGAGAATAGCCTCACTAAA 0.363662784409

GACTGAGCAGTTGTTCTCATTT 0.441413217887

GACTGAGCTACTCCTCTCGAAC 0.222653724593

GACTGAGGCTCTCCAAGGGGTT 1.22722894533

GACTGAGTATACGATTGTAATC 0.503240387959

GACTGATACTGTCGTGGTCAAA 0.399562379438

GACTGATACTGTGTTTATTAAG 0.276693618531

GACTGATCATGTCCTACACTAC 0.268843636732

GACTGATGGATTTTTTCTTAAG 0.463505466788

GACTGATTGGGGGAAGCCCAAT 1.11480346448

GACTGCTAGTTTCCCAAAGAAG 0.645057903713

GACTGGCACATGGAATGTAAAG 0.369008578695

GACTGGGTAAGTGCATCTATAC 1.24826433254

GACTGTACCGGTCGTGAGCGTG 0.675627080559

GACTGTCCACATGCTGGAGTTT 1.15862378452

GACTGTCGGTGTCACGGGGAAG 0.964286273166

GACTGTCTACTTCGGTGCACTG 0.91795256779

GACTGTCTCGCTGTTTCTTTAT 0.850210133584

GACTGTGACGGTCGGTAGCGAA 2.31456393534

GACTGTGAGAATTCTAGGAAAC 1.2565717325

GACTGTGTATTTCGACAGTCTC 1.36607623062

GACTGTTACGATGAACCCCGAT 0.645813768205

GACTGTTATCCAGCGATTTTAC 1.2813851326

GACTGTTCAACCGATTGACAAC 0.266610054211

GACTGTTTTGTAGATATATCAG 0.264065826394

GACTTACAATGTCGTGAAAATA 0.294787336379

GACTTACTTTCTGATATGTGTC 0.314704530368

GACTTGCACGGTCCATGCCTTT 0.807563986962

GACTTGGGTTGTGGTCATAGAT 1.09224543806

GACTTTCGGGCGGTTGAATCTC 1.6878514206

GACTTTGACACTGCAACTTAAC 0.536924606193

GAGAAACAATGTTCTAACGGTA 0.4539242578

GAGAAACGAAATCCATGTACAA 0.229827017363

GAGAAACTGTTTCCTACAACAG 0.308327419022

GAGAAAGAATTTGGGGCGCTAC 0.871695899039

GAGAAATACTGTCATAGTTAAC 0.652700718937

GAGAACGCATCTGTACACAAAT 0.571664475325

GAGAAGTTACCTCCTAGCCGAT 0.738907895591

GAGAATCTATCTGCTACCTAAC 0.799748344338

GAGACAAGCTTTCATACATTAT 0.336122444236

GAGACACAAGCTCTAACCCAAA 0.530344454068

GAGACACAGCCTCACGACCCAG 0.429359540445

GAGACACCACGCGATCCAAGAC 1.48885215015

GAGACACTGACTGATGAACTAG 0.381588388444

GAGACAGACTGTGATTATTGTA 0.413242869447

GAGACAGATTGACGTACCAAAC 0.410085490322

GAGACAGGCTGCGGAGCAAGTG 1.28834697686

GAGACAGTACTTTAATGTGATC 1.02170979406

GAGACATACAGTGGGTTGATAG 0.512377056931

GAGACATAGAGGCCTACCCATT 0.494604295672

GAGACATAGGCTGAAAACATAT 0.18755656698

GAGACATTAACTCATGCCGTAG 0.636287241159

GAGACATTGATTGCCGCTGAAA 0.577091503524

GAGACCCAAACACAGAGGGATG 1.32551582795

GAGACCCGATGTCAAATATAAG 0.177295840287

GAGACCGACGGTCAGACCAGTA 1.29028599398

GAGACGCGCTGTGCTCCAACTT 1.09741697434

GAGACGCTCTCCCATAGCCAAG 0.982599865606

GAGACGGACTACGTGGAGGCAG 1.82325642119

GAGACGTGATGTCAGAGAAAAC 0.621028674027

GAGACGTTTTTCGGGCGATTAG 0.263356678324

GAGACTATATCTGCAAGTCCAG 0.453416462907

GAGACTCAAAGTGGTGTTGGAG 0.675979545951

GAGACTCCATGTCAGGAAGATC 0.511743954625

GAGACTGACAGTTAAATGGGAG 0.7146695048

GAGACTGTAGAAGATCGCCTAA 0.441313881984

GAGACTTAATGAGATTGTATAT 0.483051104752

GAGAGACACATGCCGAACATAT 0.317973330542

GAGAGACATTGTGTACGGAATT 0.632180797857

GAGAGAGAATAGCATTCTTAAC 0.518714197599

GAGAGAGTACATGCTGTTATAG 0.52325711651

GAGAGATAAGGTGAAAATATAT 0.230791814985

GAGAGATTATGTGCCGGCACTC 0.873262660605

GAGAGCCCATCTTCTAAACAAT 0.552693605383

GAGAGCGCATATGATCCGGAAG 1.12277493419

GAGAGCGCTTGTGAAGTGCAAA 0.549790197971

GAGAGCGTGTATCTAGGATGAG 1.02385225714

GAGAGCTTCTATCTTCGCGAAT 0.785369004007

GAGAGCTTTTTCTGTTGCAGAG 1.0326484943

GAGAGGCACGATCAATACTGAA 0.354300534185

GAGAGGTTAGCTCCAAAATAAG 0.778144296392

GAGAGTCCACCTCATTTGCAAG 0.769811587638

GAGAGTGAAGATTCTAAGCTAC 0.390082479601

GAGAGTGATTGTGGCGGGCCAG 2.20838792889

GAGAGTGCGGAGCATTGAGATG 0.592557823269

GAGAGTGCTTGTCCTTCCCATA 0.554595900102

GAGAGTGGAAAACAAACAAGAA 0.433969107357

GAGAGTGTAACTCCAAATCAAG 0.696913675023

GAGAGTTAAATTACTAGAGATT 0.368951903321

GAGAGTTAATGAGAAAACGTAC 0.62044809192

GAGAGTTAGGGCCTAATCTTAT 0.793226973473

GAGAGTTATGCTCAGTCGAATT 0.804493937365

GAGAGTTCATGTGAAAACCAAA 0.288933140908

GAGAGTTCTCGTTGATATATAG 0.232139035941

GAGAGTTTATCCCATTATAAAA 0.366801722728

GAGAGTTTTGGTGTAGACCAAC 0.198590638408

GAGATAGCGTCTCAAATCGATA 0.20035194313

GAGATAGGTACGGCTAGTCAAT 0.68942353607

GAGATATGACATGCTACGCGAT 0.445318436052

GAGATATGAGCAGGTGGTGTAT 1.14847089253

GAGATATTGACTGGTAAGACTA 0.497373207845

GAGATCTCCTGTCGTGTGGCAG 0.569577087549

GAGATGGGCAGTCCTTGGTAAG 0.832251787335

GAGATGTATGCTTATGGCTAAA 0.546414369494

GAGATTCACTGTGCTGATGATT 0.884424299826

GAGATTTATTGTCTAAAAGAAC 0.228697876779

GAGCAATCACCTGTTCTGGCAT 0.448983822536

GAGCAATCATGTTAAACGGGAC 0.174058917402

GAGCAATTAAGTCCAGGATCAC 0.444104921394

GAGCAATTATATCCTGAGACAT 0.369421336149

GAGCACCGATCGGGAGTTATTT 1.42428946568

GAGCATCCCCGTCCACGCGTAT 1.16852054306

GAGCATTCAAGTATTAGCCCTA 0.390205832416

GAGCCAAAAGCTGGTAGATTAG 0.803520866842

GAGCCAAAGGCTCAGTGGGGAG 0.898245874238

GAGCCACAACGTCAATAATAAG 0.282956499299

GAGCCACAATACCCTTAGGCAT 0.933514277158

GAGCCACAATTTGATTCATAAC 0.412759558339

GAGCCACCCAGTCAGCGCACAT 2.54481599278

GAGCCAGTCTCTCCTATAGCAG 0.822230163927

GAGCCATAAGGTGCAGAGGAAC 0.320099679473

GAGCCATATTCACCGGGGCATA 0.20002439117

GAGCCATTAAGTTATCGCTATG 0.889856991347

GAGCCATTTCCGGCTTGGTCAA 0.521628212676

GAGCCCCGATGTTATTGTGAAG 1.09124196829

GAGCCCCTGTGCGTGGTGCAAG 1.47892748116

GAGCCCGGACTGCCTGGTTAAC 0.972904986771

GAGCCCTAATGTCATTTATCAT 0.23681324819

GAGCCGGGAAGTGCTGTGTGAT 0.871282406856

GAGCCGTAATGCGCTGAGTAAA 0.549878369859

GAGCCTAACTTTTATAACCCTC 0.846060753137

GAGCCTGAAATTGCTCCGCTTC 0.324034954882

GAGCCTGTATGTCATGTTGGAC 0.788667780477

GAGCCTTATATTACTACCCAAT 0.703747675161

GAGCCTTTCAGTCTTACATTAC 0.286381140608

GAGCGACATGCTCGTTAGTAAA 0.665269350133

GAGCGAGCATCTCCCTGATTAC 0.589947831843

GAGCGAGTGAGTGCTCCGCTAC 0.469848751103

GAGCGATAGTAAGCACCGCTTG 0.384995469874

GAGCGCCAGTTTGCATACAGAG 1.02658942761

GAGCGCCGGCGAGATATGTTAG 1.70540646843

GAGCGCCTAAGAGACAGTTGAA 0.46148604955

GAGCGCCTGCATCCAATATTAT 0.619589023918

GAGCGCGACTGCCCAGAACAAT 1.33862200707

GAGCGCGAGTGTGTCGGAGTAC 1.75917115811

GAGCGGCAACCGGATTTTTAAC 1.14613827987

GAGCGGGCAGGTCCGGGTAGAA 1.71996311784

GAGCGGTCATCTCCTCCGCATG 0.965759004425

GAGCGTGCGTCTACAGAAAAAC 0.987469604599

GAGCGTGCTCCTCCTACTCAAG 0.980362864333

GAGCGTGGAGGTCCCGTGCAAG 1.19915601947

GAGCGTGTGGCGGATGTGGGTG 2.6884761444

GAGCGTGTGTCTCCGGGGTAAC 0.754502832486

GAGCGTTGCACTCATGACGTAG 0.702312523135

GAGCTACCGTCTGAGGCCTCAA 0.898157625885

GAGCTAGAATGAGATGCCCAAA 0.657946014393

GAGCTATATTCTTCCGCTAGAT 0.40183287641

GAGCTTCTCTTGGTTGCCTAAC 0.708690917148

GAGCTTCTGCATGGTTAAGTAT 0.254057831663

GAGCTTGATACTCTAAGTCAAT 0.456753137787

GAGGAAGAAGCTGAATGTGCAA 0.536103624356

GAGGAAGAATGTGGTACAGAAG 0.791653256367

GAGGAATACTCTGACCGCTAAT 0.663439785561

GAGGATCGAAGCGAAAAAAGTA 0.239637375671

GAGGATTACAGTGCACGCAAAA 0.576713678246

GAGGCAGAAACTGAGGAGAGAG 0.531932342273

GAGGCAGTTTGTCCAATTTTAG 1.06538823453

GAGGCATCTTCTTCACCCTGAG 0.500859511429

GAGGCATTCGCTCCTAATTCAC 0.809697036378

GAGGCATTTGGGACTCGTAGTT 0.434572886197

GAGGCTAAATATGAATAGGTAT 0.457594517953

GAGGCTCAGACTGGTACAGTAA 0.415756008494

GAGGCTGAAAATCCGGTTAATG 0.645819202071

GAGGCTGAGATTGAATGTTCAA 0.362345838397

GAGGCTTTACCTCATACGTAAG 0.432933816904

GAGGGAAACGATTAATCGCAAC 0.293448448877

GAGGGACCGAGTGGTCCTGTAC 1.03751343623

GAGGGAGATGCTGATGAACAAG 0.329108409206

GAGGGATGATATACGCTTGTTG 2.02017499932

GAGGGCCTATTTGCTCAAGGAC 1.11523914086

GAGGGCGAATATCATTTGTGAA 0.699137103534

GAGGGGCTTGCTCTCTGGCAAG 0.639587229284

GAGGGGGGATGTCCAGTCAAAA 1.07783925512

GAGGGTCACTCTGGAGCTGATC 1.15426809004

GAGGGTGGACATCAATACAATC 0.427826014353

GAGGGTGTGTATGCTCTGGTAT 0.515639500676

GAGGGTTATCTTCAACACGATC 0.403710059559

GAGGTATATACTCGCACGGCAG 0.74729492286

GAGGTCGAGTCTCCTAGGGGTG 0.324283038435

GAGTAAATATCTCATGTAGGAT 0.746147314082

GAGTAACGCGGTCCTGAAAGAT 0.737918028003

GAGTAACTATACCCGTAATAAT 0.441759977353

GAGTAATTATGTCAAGGTGAAA 0.335823745814

GAGTAATTTTCTTGATAGTTAG 0.482666273389

GAGTACCACGCATCCAGGCATT 0.610815887428

GAGTACGGAACTGAAGAAGTAA 0.315464692041

GAGTATACTCGCGAAGTTTAAG 0.377939173505

GAGTATTGATGTTGCTGTAGAC 0.312832565275

GAGTATTTCGTTCTTACTCAAG 0.365325548721

GAGTCAATTAATCCCCCGTTTG 0.369249480567

GAGTCACAATATCAGTCAGCAG 0.340075499727

GAGTCACAATTTGGCCGATAAG 0.793434630976

GAGTCACACGGTCTTGCTCGAG 1.29143991042

GAGTCAGAACCTGTTCACAGAG 0.394893046049

GAGTCAGACATATTTCTGGCAC 0.506433166694

GAGTCAGTACGTGAACGGACAG 0.251132304991

GAGTCATCAACTGGTGGGTCAA 0.556423812051

GAGTCATCTAATCTATCATCTC 0.529996497453

GAGTCATCTTCTCATTCATTTA 0.496523277023

GAGTCATTGAGATAAGTGAGAA 0.421080795083

GAGTCATTGGGCCCTATTATAG 0.757242103429

GAGTCCCGACTTGCCGCTCCAT 1.35792363952

GAGTCCGAAGATGCTAGTGCAC 0.799645672262

GAGTCCGTAAATGATCAAACAG 0.489108980995

GAGTCGGCCCATCCAGGACAAC 1.73532935223

GAGTCTCAGTTTAATCCTGGTC 0.373381245755

GAGTCTCCAGCTGGTGGTTCAT 1.56279743076

GAGTCTCGATGCCCAGAAATAG 0.496867273347

GAGTCTCTCTTTCTTCCACAAA 0.49177821154

GAGTCTGAAGGTCCAATCGAAT 0.697130650547

GAGTCTGGTAGTTCCTAGCAAC 0.894719539134

GAGTCTGTAACTCTTTTGCTAC 0.499140806848

GAGTCTGTCTTTGTACGCTGTT 0.833381219129

GAGTCTTTACGAGATCGATTAA 1.24566100849

GAGTGACACAGCGCAACCCCTC 1.06531025477

GAGTGACGATGTGATTGGAAAC 0.733152765165

GAGTGACTTCCGGTAGTAAGAC 0.808796801885

GAGTGAGAATGTGGATAAAAAA 0.664750129503

GAGTGAGCATATCCTCACAGAC 0.755904012769

GAGTGAGTACGTGCTGGCGAAA 0.837884694035

GAGTGAGTAGGCTGTGACAAAA 0.781192978116

GAGTGATAGCCTCAAACCTGAG 0.838657316821

GAGTGATATGGTCTATTCGTAC 0.744736207726

GAGTGATCCTGTCTTAATCTTC 0.616869192575

GAGTGATGCTGTGTTTTATGTG 0.549178488047

GAGTGATTAACTGCCAGTACAT 0.309198844489

GAGTGATTGTATCTAGTTCCTG 1.8532462123

GAGTGATTTTGTGCTTATGAAA 0.418601021865

GAGTGCCAGTGTCCTAACCGTG 1.43681910735

GAGTGCCTGCGTGCGGGGTGAT 3.30166482568

GAGTGCTTGTGTCCAATCTAAC 1.07251148258

GAGTGGGTAAGACATTATCTAG 0.462217376069

GAGTGGGTGGTTCTTAAGATTA 0.500808073495

GAGTGGTGATCGCATACATAAA 0.527106699616

GAGTGGTTAATTGAATTTGATC 0.756472772444

GAGTGTCAATGTGAGGCGTGAA 0.772694660446

GAGTGTCTGTATGGTTTTTCAC 1.31784484684

GAGTGTGGATGGCGAGAGTCAG 0.970445642667

GAGTGTGTCTTTGATTAATCAG 0.327752158179

GAGTGTGTGTGCGTAAAAAAAC 1.05939615618

GAGTGTTCATATCGAACCTAAG 0.902485419262

GAGTTATAGTCTCCATAGTCTG 0.690492844453

GAGTTCTAATGTTGTCAGCCAC 0.635746321884

GAGTTCTGTTTTCCAGTTTGAC 0.277234102804

GAGTTTGGGGCTGGTCGCTGTG 1.26211685726

GATAAAGCGGAAGATGTGGTAA 0.73033444187

GATAAAGCGTGTGGTATCAAAC 0.588214083711

GATAACTTAGCTGTTTAGCTAC 0.156631876096

GATAATCCCTGTCATGCGTAAC 0.282294631366

GATAATCGGACTGTTGAAGGAA 0.561134601532

GATAATTACTCTGGATTCACAG 0.570553372685

GATACACAATTTGATTGTTGAA 0.406651312323

GATACACGATCTGTTAAATAAG 0.405165373371

GATACACTATGTGCCGTGTGAA 0.850935047899

GATACACTGTCTGGGGTTCTAG 0.872794081456

GATACAGAATGGGGGACGTGAA 0.691880074211

GATACAGATGGCCATACAAGAT 0.578577495666

GATACAGGGGGTGATGTTTAAG 1.20742114377

GATACAGGTTGTCAAAGAGATC 0.417561405096

GATACAGTCATTGCTCGCTTTC 0.36674934744

GATACATAGCCATATAGTGAAT 0.346441154234

GATACGTGATTTGTTCGGGCAA 0.492937190165

GATACTACCAGTTCATGGAAAG 0.641492435015

GATACTCAGGCTGATCGACAAC 0.869045198546

GATACTCTAACTGGTACGTAAT 0.18560837929

GATACTGACACTGAAAACATAG 0.205245199039

GATACTGCAGGTGAAATGTAAC 0.841422456515

GATACTGGTATGCATAATATAC 0.352644885519

GATACTGTGAATGAACAAGGAC 0.743577696994

GATACTGTGAGTTGAGCATAAG 0.312520737138

GATACTTACGGCGATTGTTTAC 0.153504873299

GATACTTCATGGCCGCGATAAG 0.648211377327

GATACTTCCCGTGTCTGGACAT 0.371343888191

GATACTTTATGTCCGGTTTGAC 0.331369447041

GATAGACAAGGTGTTAACGCTT 0.291067963276

GATAGACAATCTGTTAAGTAAC 0.30320288254

GATAGACGATGTGAATAACTAG 0.908022290175

GATAGACTACGTGGTTCTGTTG 0.593174648158

GATAGACTTGATCCACTCACAT 0.222332358892

GATAGAGGACGTGCTTCCTTAT 0.886785319741

GATAGATGATGTTCAGTTTAAG 0.633053405525

GATAGATTCAGTTATGACCGAT 0.420535726538

GATAGCCTCTCTGCTTGGTCTC 0.769602993477

GATAGCTACTCAGTTCACGAAC 0.871804022857

GATAGTACGTCTGCTACCGAAA 0.531788042829

GATAGTATCAAGGGCCTTATAG 0.835307985613

GATAGTCAATGAGAGTACCGAC 0.277327285518

GATAGTGAATTGCCCGGTGGAT 1.75728654246

GATAGTGCTGGACCAAAACGAC 0.422263652896

GATAGTGTAACTGGTCGGCTAT 0.157511630083

GATAGTGTAGCTTGTGAGAAAG 0.794845724751

GATAGTGTCCCTCTTTGTTAAT 0.384216646919

GATAGTTATGATTGCTACTAAG 0.214294754798

GATAGTTATTGCGCAGGAAAAG 0.547234122382

GATAGTTTAACTGCAGGGTAAG 0.717899599777

GATATAGTTAGTCATTGCAAAG 0.353778228178

GATATTCCTTCTCATGAACAAG 0.969492040035

GATCAATTGTCTGACACAATAG 0.358943232729

GATCATTATTGTCCCAATGAAA 0.37479609156

GATCCACCGTCTGGTAGGCGAC 0.67549349321

GATCCCCATCGTCATGAGAAAA 0.298031403387

GATCCCCCTTGTGATTGGCCAG 0.933765910278

GATCCTCAATGTCACGAATGAA 0.518508580369

GATCCTGTAGCGCCCACAAAAT 0.65969859448

GATCCTTAGCCTCCAAGCGTAA 0.734909798503

GATCCTTTAGCTGAAGTTTCAG 0.338752968524

GATCCTTTCAGTTTATGGCATA 0.366908824445

GATCGAGATGGTCATTCACCTC 0.76002221016

GATCGAGTTTACTGTAAGTGAT 0.462857215204

GATCGCCAGCCTCCAAAGGGAG 1.11162675811

GATCGCCGATTCTCTGGAGTAA 1.56737409119

GATCGCGAGCATCAACCAGAAA 0.489407053539

GATCGCGTCTAACCTCTAAAAG 0.349745795833

GATCGTCTCGCTTTTTCTGAAA 0.446832445261

GATCGTCTTACACAACTCCAAC 0.381744658863

GATCGTGAAATTGATGCCGGAT 0.57492194148

GATCGTGCATGTGGAGTAAATG 0.497768857131

GATCGTGTTTCTGTCAACAAAT 0.420826425361

GATCTAGTCGGTCGACGAGAAG 1.14905046106

GATCTTAAACATGGTTTCATAC 0.39853641625

GATCTTTATTCTGGAGACCAAC 0.818756063171

GATGCAAGAGGTCCTTATTAAA 0.339132987255

GATGCATAGTGTCATGGTACAT 0.202159749887

GATGCGGGAGTTTTTACATTAT 0.331833204992

GATGCTCAAAGTGATGTGCCTC 0.571264256801

GATGCTGAGTGTCAGGGGTCAC 0.833830261412

GATGCTTGTAGTGAATTGGCAA 0.355430347234

GATGGACACTTGTCTATAGGAG 0.568124937267

GATGGACCACCTTAAATTGCAG 0.460468790635

GATGGACCTACTGATAACCGAC 0.301698898278

GATGGAGTATCGCATTGCACAC 0.837794692475

GATGGTGACACTGCACCAATAG 0.658111676153

GATGTTCGGCCTGCTGTTATAG 1.36678713439

GATGTTGAGATTCCTAGAGAAG 0.334728557106

GATTAATCGTCTTCTTCTTAAG 0.144505360011

GATTAGCCCACTGCAGAATATC 0.335063970068

GATTATCTACGTCTATGTGGAA 0.435707032881

GATTCACATTATGCTCGTGGAT 0.207925483234

GATTCAGTAACTCCTACAGATT 0.554370372102

GATTCAGTATTTTCTACGGAAG 0.512338188886

GATTCATAAACTCGCTGGGTAA 0.811938037084

GATTCATAAATTTAAATTGGTT 0.425896919712

GATTCCGTGTATCATTCCGTAG 1.58069458041

GATTCGGACTGTCTTTCTCGAC 0.719985207332

GATTCTATAGCTGGTACAGGAA 0.313901023265

GATTCTCAGTTTGCTTGCTCAC 0.493446719505

GATTCTCCATTTGAATAAGCAG 0.380573526301

GATTCTCGAGATGAAGCTCAAA 1.99840096125

GATTCTGAAACTTGTAACAGTG 0.401902562398

GATTCTGGCGGTGCTTGGTATA 0.535225993288

GATTCTTAAGTTCGTAGGTAAG 0.371140925939

GATTCTTGGGACGGATACCAAG 0.941557768177

GATTGACGGTTTGGCCCAATAA 0.2783175961

GATTGACTGTATGTGCCGCCAT 0.830054643905

GATTGAGTACGTGTTCCAAGAG 0.815918788181

GATTGAGTCTTTTTAATTAAAC 0.617953423406

GATTGAGTGAGTCCTCATCGAT 0.582032127803

GATTGATAATGGACGCTATCTT 0.496240837778

GATTGATAATGTCCTAAAGTAC 0.518916765449

GATTGATAGTATTCCGATGGTT 0.474270990619

GATTGATGTCCTGCTCATCTAG 0.332948563545

GATTGCGTCTTACGAGGTTAAC 0.718440139091

GATTGTCAATCTCCAACTCAAT 0.44623446237

GATTGTGATTCGGCAGCTAAAG 0.574037065882

GATTGTGCTTGTGCTCCTGTAC 0.757149217857

GATTGTTCCTTGTATAAATTAA 0.241294705468

GATTTGTAATCTCAAATCAATG 0.520678820277

GATTTGTGAAGTAACCGTTGAA 0.301194793108

GATTTTATCTGGGTATATTGAG 0.631600857078

GATTTTCGTTGACCAGGTACAA 0.480870859427

GCAAACGTGGGGCCAACTTCAA 0.927298066307

GCAAAGTCATCTGGTGCATATG 0.370224399569

GCAAATTTGCTTGCATGGTATC 0.666159858611

GCAACAGACTGGGCACCAGCAT 0.902048037845

GCAACAGTTTCACATTCTAATG 0.32804589975

GCAACTCGAGGTCATTAAGTAA 0.984102347702

GCAAGTCATAGTCTAAAGTAAA 0.721377597589

GCAATTTCGTCTGGAAACGAAA 0.558068004156

GCACCGCACTCTGCTAAATAAC 0.395307887313

GCACGATACTCTTGTACCTGAA 0.313684326913

GCACGATCGGTTGACACGAATA 0.945461167208

GCAGGGGTAGGTCATAGGTAAT 0.810655242471

GCATAATTTAGTCGTATAGTAA 0.302217711998

GCATCAATATGTTGAGGTGATG 0.522239527652

GCATCGGCGTATGCTTACGTAG 1.15129214533

GCATGACTTTCTCCAACTTCAG 0.634978380074

GCATGCTATTCTCAATATGGAT 0.716551386772

GCATGTTAATCTCAAAGAGTAG 0.422922885728

GCCAAAAACAGTTATAAGTAAG 0.324581092097

GCCAAAACATGTGATCGTCAAC 0.526220733735

GCCAAACTAGGGCATATTCGAG 0.557608066445

GCCAAAGCCGTTCACGTATCAA 0.150846799047

GCCAAAGCGTCCGATACAGGAG 0.980500260092

GCCAAATAGTCTGAATAGATAG 0.677840957962

GCCAAATGATCTGACAGGCTAT 1.49404646567

GCCAACGGATCTCAGCTTGAAC 0.927143506516

GCCAACTTCGGTGCGACGTGAA 1.51970390707

GCCAAGGAATGGGCTCGATAAG 0.768984510205

GCCAATGCGGCCGCTCTACAAG 0.864163905952

GCCACACCATTGGATCCACGAA 0.494385063033

GCCACACGAAATGCAAAAGATC 0.409907053143

GCCACACGGGTTGAATATAGAC 0.455844375275

GCCACACGGTGTTTTGATTTTC 0.723872398116

GCCACACTAACTGCAGGACTAC 0.234012655533

GCCACACTAATTCAACGTGTAG 0.185586074952

GCCACAGCAGCTCTTACCGGAC 1.33518223549

GCCACAGCATCAGAGTATTGAA 0.618510517168

GCCACAGCATCTGTTACGAATC 0.429829903097

GCCACAGGAATTGTTAAGGTAT 0.563853422896

GCCACAGGATATGACAACCTAA 1.09467137121

GCCACAGGGACTGCTCTCACAC 1.1947048854

GCCACAGTAAGTCCTTTCAAAC 0.404631001618

GCCACATATTGGGTAGGCAAAG 1.39209121887

GCCACATCTCCTCCTTAGGAAA 0.227313809712

GCCACATTCTCTGCTATGGCAG 0.878924779379

GCCACCCATTCTTGAGGGTAAG 0.841344342393

GCCACCCCAACTTATTACAGAC 0.359915206644

GCCACCCCCCGTCCATCTCGTG 0.635196429066

GCCACCGCCTCTCAATACTGAA 0.698673236102

GCCACCGGATGACTATCATGAG 0.834760137413

GCCACCTACGCTGCATCCTCAC 1.39651391571

GCCACCTATAGTGGTATGTTAT 0.581405705996

GCCACGGTCTATCTTCTATAAT 0.872249503303

GCCACGTACGCTGCTGGACAAC 0.58097130384

GCCACGTGCCTTGAAACACAAA 0.817400782964

GCCACTACATATGCTACCGATT 0.941163257254

GCCACTACATTTCCTGCTATAC 0.852260469568

GCCACTCAGATTCCAGCTACTG 0.148567763042

GCCACTCTATCTCCTTGCTAAC 0.656165773966

GCCACTCTGTCTCTTTTTAAAC 0.666090061081

GCCACTGCCGCTCAAAGGTTAC 0.576020585102

GCCACTGGCTACGGTAAATAAA 0.465271360652

GCCACTGTAACTGCTGCTCAAC 0.246845315642

GCCACTTAAGGAGCTAGGCTAA 0.24427537295

GCCACTTAATGTCGAGCTTGTA 0.863004378098

GCCACTTTATAACGTCGAAGAG 0.59449609769

GCCAGAACCTCTGTTAGCCTAG 0.600440340462

GCCAGACAAACTCATGCAATAG 0.388532347969

GCCAGACAAAGCGTTGCTGAAA 0.914879141742

GCCAGACTGCATGAAGTTGATA 0.700835388465

GCCAGACTTACTGCACTATGAC 0.619085604112

GCCAGAGAAGACGCCGTTTGAC 0.490904890442

GCCAGAGAAGGTGATACCGAAT 0.720349760384

GCCAGAGACTGTTCCGACGAAA 0.462750806613

GCCAGAGTCGATCCGCTGTTAC 0.456934824245

GCCAGATACTTGCCTAGTAAAG 0.588516061925

GCCAGATTGCTTTAATCCCGAG 1.27783656368

GCCAGATTGGGTGATTTGGCAC 0.651160583607

GCCAGCACAGGTCATTTCCAAT 0.747616821102

GCCAGCCTTACTGATAGCTTTT 0.672779507294

GCCAGCGCGTGTGATATGAAAC 0.876072674469

GCCAGCTAGATTGCAATACTAT 0.148727746497

GCCAGTAAAGGTGATGACGATC 0.992754152149

GCCAGTAACTATCATCCAGAAG 0.523529576145

GCCAGTACCACCGCAATATATC 0.453187399005

GCCAGTCAGTCTGGTACCTAAT 0.750169079841

GCCAGTCGCATTCTAGTATATG 0.746600320872

GCCAGTCTAATTGGACCTCAAA 0.630816962196

GCCAGTGAATCGGTAACAGTAC 0.922116155248

GCCAGTGCCGGTGCTGCTCTAA 0.992172992205

GCCAGTGTATCTCGACCCCGAC 0.726935156494

GCCAGTTAAACTTCTTTGCAAG 0.557637579536

GCCAGTTACTGATAAGGCTGAT 0.658889943909

GCCAGTTCCTGTCAATCGTAAC 0.729438494617

GCCAGTTTCGGCGAATCGCGAG 0.570535188198

GCCATAGACTGCCAAGCTCGTG 1.62261822559

GCCATATTCTGTGCATAGTCTT 0.448626910842

GCCATGCAATAGGCCGCTGATT 1.28882728839

GCCATGGTGACGTATAGAAAAC 0.744635454521

GCCATTCGGTCTGCTACTAAAT 0.531805316354

GCCATTGACCCGGAAAACGCAC 0.658332769635

GCCATTGAGAGGCATATCCAAC 0.411019711366

GCCATTGGCCGTGGTACCATTG 1.06413602524

GCCATTTTTCCTTCTGGCTATG 0.740358279973

GCCCAAGAATATGGTGCTTGAT 0.639556682012

GCCCAAGAGCTTCATGGTAAAG 1.14821161613

GCCCAATGGGGTCCTCCCTCTT 0.910767446405

GCCCAATTGTCTCGTCGAAAAT 0.946603805038

GCCCACGTTTGTGGTGCGATAG 0.34835800727

GCCCAGCGATCTGATGTCCGAG 0.958339168154

GCCCAGGTAGCCGCATGAATAG 0.319961123488

GCCCATTTAGGTCTTTTAAAAG 0.783944364556

GCCCCACAGGGTCTAGGTGGTT 0.857214860723

GCCCCACAGTATTCTTTTGATA 0.496401276358

GCCCCACCAGCTCGTGGTTGAT 1.29298700365

GCCCCACGCTCAGCTACAGGTC 0.184536558165

GCCCCACTCTATGCAGAATGAA 0.301807267555

GCCCCAGAGTATCACTGCAAAG 0.248212760393

GCCCCAGGACCTGTTTTGTTAG 0.895758234795

GCCCCAGTACCTTATTGTCAAG 1.13243210115

GCCCCAGTTTCTGCTAGGTATT 1.3554457442

GCCCCATAACTTCGTCTTTGAG 0.396550711751

GCCCCATAAGATGTTTACGAAC 0.465558118708

GCCCCATCCCATCCTAAATCAA 0.382874881472

GCCCCATTATCTGCGAAGGAAC 1.17074286977

GCCCCCCACGCCGTAACTTAAG 0.820076412079

GCCCCCCCATCTCAAGCCATAT 0.59293048891

GCCCCTCTCTGTGAGTGCTAAC 0.638391917388

GCCCCTTGATGTGATAAGACAC 0.810524720802

GCCCGACAATAGCCACATTGAC 1.45713182187

GCCCGAGAAAGTGAACGACTAA 0.385460365317

GCCCGAGACTCTGGTCTCGTTA 0.516371974105

GCCCGAGTCTCTGAACCGCAAG 1.34614356833

GCCCGAGTGCATAAACTAGAAT 1.11259308496

GCCCGATATTCTGATTTTCTAG 0.282709782616

GCCCGCGACGGCGTTGGTACTA 3.29197088409

GCCCGCGGAACTGAGTAGTGAT 1.77083309241

GCCCGCGGCACGGAAACGTAAC 0.783014766863

GCCCGCGTCCGTGATCTAGAAT 1.18125687224

GCCCGCTAATGTCCTCGCTAAA 0.473321496793

GCCCGCTGGACTGGAAATGAAT 1.13512001319

GCCCGGCGAGCTCATGCGTGAC 1.36646320005

GCCCGGCTATCTGCATTTAGAG 0.716144722162

GCCCGGTACTGTCAGGTGTAAC 1.47913276958

GCCCGGTTAGGCGTTGCTCGAT 1.44361870194

GCCCGTCAATGTGATTTTGGAC 0.314673836857

GCCCGTGTGGCTGAATAGACAG 0.452207907693

GCCCGTGTTTCTGATTCATAAG 0.223349737167

GCCCGTTAATCCCCGAGCTAAG 1.25361717947

GCCCTAGTATTTGAATTGATAT 0.409868099957

GCCCTAGTCTCTTTGTTTAGAC 0.453914705197

GCCCTTGTTACTCCTCAAGAAG 0.320880271866

GCCGAACGGTTTCCTAATAAAA 0.555047797721

GCCGAACTGACTGCTTGCGCAG 0.985762122595

GCCGAATACTGTCTTGTGAAAC 0.188976855513

GCCGAATATCGGCCTTGTTTAG 0.85029679013

GCCGAATATTTACATAAAGGAC 0.295446867868

GCCGAATTACCTGTTCACGCAA 0.448857962517

GCCGATGACTCTGGAGGAGAAC 1.3093561038

GCCGATGAGGTTGATAAGTCAT 0.199049077473

GCCGCAAAATGTCTATACTAAG 0.855993296733

GCCGCAGTGAGGGTCGGATCAA 1.27788720861

GCCGCATGATCTGTAAGTTAAC 0.282428046955

GCCGCCGCATATTATAAATTAG 0.969653224501

GCCGCTCATTCTGCGACGTCAC 1.98436658833

GCCGCTCTATGTTTATGCCGAT 0.485553656903

GCCGCTGACTTAGAGTCAAAAA 0.940210967209

GCCGGACAGTTGCATACGTAAA 1.17800618871

GCCGGACCGGCTCATTAAGATC 0.705015016343

GCCGGACGATTTCCAGATGCTT 1.47286874364

GCCGGAGGACATTAAAAATATC 0.770028696996

GCCGGATCGTCTTAACCCAAAG 0.387650050113

GCCGGCGAGTCCCAGTGATAAT 1.17774911632

GCCGGCGTCTGTGCCCGTAAAC 0.70667618811

GCCGGCTAGTGTGATTCCGTAG 1.66028457858

GCCGGTTCACGGCCTACACAAC 0.947033856279

GCCGGTTGCTATCAGGAAATTA 0.330052571673

GCCGTATAAGCTCATCTCTAAA 0.288983621649

GCCGTCTGAAGTTGTCTGCCAG 1.45932007556

GCCGTTCACTTTCATTCATAAT 0.280752694438

GCCGTTTTAAGGCCTATGTAAG 0.65596778568

GCCTAAGCTACTCAACGTGATC 0.539193652811

GCCTAATACTCTGCACAAATAT 0.261614143289

GCCTACCATTCTTATCGATATT 0.336776291484

GCCTACTCAACTGATGTTAAAT 0.560070811144

GCCTAGGCCATTGATTATATAC 0.460080857647

GCCTATCAGCCTGGTGGCTCAG 1.02889708222

GCCTATGATAGAGCTGCTAAAC 0.435177892977

GCCTCAAGGACTCAAACCCCAA 0.237638777413

GCCTCAATCCCTCCAATTGAAG 1.18763055708

GCCTCACCACCTGCAAATCTTG 0.582429206884

GCCTCACTATGTCGACCTTAAT 1.04563471512

GCCTCAGAATGTCCGCGTACAC 0.575399514353

GCCTCAGAGTATGCTGCCTGAT 1.47790773248

GCCTCAGATTGTTGTAAGAATC 0.765411884651

GCCTCAGCATCTGTGTCGGAAA 1.17755456893

GCCTCAGGATTTGCTCTGCTAG 0.820996981278

GCCTCAGTATGTGGACGAGAAT 0.368178691819

GCCTCAGTATGTGGTAATTTAT 0.420677932996

GCCTCAGTCGATGGTTAATAAC 0.303442610582

GCCTCAGTGACTCCCAATAAAT 0.601185094028

GCCTCATAATTTGAGCAGTATC 0.929682529154

GCCTCATACCGTCCTGGTATAT 0.218919719492

GCCTCATGTCGTGATGGCCCAG 0.932104833143

GCCTCATTAGCTCAGAGGGCAG 1.82322664288

GCCTCATTCTGTCCACCGAAAG 0.340636958065

GCCTCATTGTCTGACTGGACTG 0.724423124519

GCCTCCCAGGCTCATCACGAAT 0.106734282241

GCCTCCCGCGCACGTGTGCGTA 0.732965561255

GCCTCCTTATGTCCTGTTTGAA 1.205804149

GCCTCGTAATCTGGACTACGAA 0.715146610348

GCCTCGTTGTTGGATGTCTATT 1.02877792648

GCCTCTCAATTTGAGACAGTAG 0.29350123849

GCCTCTCTTACTGCTGATGAAT 0.655646575994

GCCTCTGACGTTCAAAGGTTAA 0.520763077471

GCCTCTGGGTCTCCTCTTCTAT 0.361118790989

GCCTCTGTTAGTCCAAAATAAG 0.680475905979

GCCTGACAGTTTTAAATCAAAA 0.495118379707

GCCTGACCAGGTTATCATATAA 0.882795898003

GCCTGACCATCTCAAGTCGGAT 1.1499252177

GCCTGACCGTATGACTGTACAA 0.396393853417

GCCTGACGATGTCCTCGTGGAC 1.11362065826

GCCTGACGATGTGATTGTTCAC 0.88645764895

GCCTGACTAGCTCAACTGTCAG 1.07731649916

GCCTGACTCTGTGTACAGGGAC 1.38350110992

GCCTGACTCTTTGAATATGTAC 0.428380477535

GCCTGAGACACTTCGAGTCAAG 1.04442370441

GCCTGAGAGTGTCCCGATTGAG 0.932938190795

GCCTGAGCATCTCAATAGGGAA 0.602974564945

GCCTGAGGCAGTGACTTTTTAG 0.802669541249

GCCTGAGGGTTTGGCCTGTAAT 1.06638401209

GCCTGATAGACTCGAGCCTCAA 0.568122471309

GCCTGATTCACTTCAGTCGAAG 0.814396031613

GCCTGCAGTAACTTATGACGAC 0.736215429376

GCCTGCCACAATCTTCATGAAG 0.524974455892

GCCTGCCACGTTGTTTCATAAT 0.666637837347

GCCTGCTAGAGTCAACAGTAAG 0.751542608132

GCCTGCTATCGTGCTGAGAAAT 0.238633039869

GCCTGCTTATACGCTGCTTTAA 0.1310578511

GCCTGGTTCTTTCGACATAAAA 0.279031449296

GCCTGTAGCACTGATTGGTGAC 1.42296038319

GCCTGTCAAAATGAGAACACAG 0.453270830004

GCCTGTCGAAGTGCTTCGATAG 0.68217379947

GCCTGTCTCGGTCATGCCGTTG 1.02349183217

GCCTGTCTGAGTGACGAACAAA 0.60478115752

GCCTGTGAATCTCCACGCGTTG 0.777361724357

GCCTGTGCTTTTGCTGCTGTAG 1.49286249289

GCCTGTTAGTGTGATATAGAAT 0.600087814051

GCCTGTTGCTCTGAAACTAGAC 0.624540389471

GCCTGTTTGTATGCAGCTCAAC 0.845215843751

GCCTGTTTTGGTTCTATAGTTC 0.319404545391

GCCTTACAATCTCATACTAAAA 0.511714719707

GCCTTACCATCGGCCACCCAAT 0.972246476269

GCCTTCCATTTGTTAATGGAAA 0.959866307273

GCCTTTCGACGTGCTCGATAAC 0.932379702521

GCCTTTGAAGTTTTCTATCCAG 0.774207426465

GCCTTTGGGTGTTTTTCTGAAT 0.656070611285

GCCTTTTTTTCTGATATTTTAA 0.661016072647

GCGAAACTACACCCTGCATCAC 0.735179053894

GCGAAAGTAGTGCATGCTAAAT 0.525726691194

GCGAAATACTGTCCTTAAGTAA 0.696521546449

GCGAAATCTATTTAATCAATAG 0.456746393937

GCGAACCTGTTTGGTGCAGGAA 0.765333561089

GCGAACTAGTGAGAAGAATGAT 0.251715958156

GCGAAGGAGTCTGATCCCAATA 1.00880389754

GCGAATAAGTATCAAAACTTAA 0.284807753151

GCGAATGAGCGTGATTTTATAG 0.619788438742

GCGACACTAGCTTCTATGACAA 0.328262182027

GCGACACTTGGCGCCGACTGAA 1.03768273288

GCGACAGACGCTGGTAGTTGAT 0.608255901206

GCGACAGCACGTGAATAGATAT 0.305517880438

GCGACAGCGTCTCGTTGGATAT 0.191822737319

GCGACAGCGTCTTATAAGCAAA 0.725376888204

GCGACAGTAACTGCACATCAAC 0.733168091968

GCGACATAGACTCCTTAGAAAC 0.320707443904

GCGACATGCGATCGTAACAATG 0.950382931504

GCGACATTAGGTGACCGGTAAC 0.890793415711

GCGACCGAATTTGCCTATTCAC 0.425652970233

GCGACCGAGTTTCTAAAGGAAC 0.277984295781

GCGACCTCATATGTTGAGAAAC 0.290042169375

GCGACGGATTATCGATGGAATT 0.163799202787

GCGACGGTGTCTTCTTCTGAAC 0.751464183462

GCGACGTTGTCTGCTGATTTAG 0.70302255743

GCGACTCAACCGGCTGCCGTAA 1.62018259408

GCGACTCCACGGCATACTCCAT 0.346094406043

GCGACTCCGTATCATGAAAGAG 0.908801510469

GCGACTGAATCAGCATACTTAT 0.779319706672

GCGACTGGCTATTGTAGGATAT 1.13149113429

GCGACTGGGCCTCATTAGTAAG 0.288634041369

GCGACTTAATGTTCGATGTCAG 0.415470504

GCGACTTAATTTGCTCTTTGAG 0.651675239859

GCGAGAAAGTCTCCCGTTAGAA 0.519771734951

GCGAGAAGTGGTGATAAGCCAG 0.354833386154

GCGAGAGAACGTGAAGCGGAAA 0.575527204526

GCGAGAGCATCCCAAAAAACAG 0.747392642827

GCGAGAGGCTTTGCAGTGAATG 0.326959204049

GCGAGAGGGGCTTTGACTCTTC 1.19870461455

GCGAGAGGTTGTGCAAAATAAG 0.654547162276

GCGAGAGTCCTCCATGAGTATC 0.240999857824

GCGAGATCAGTTGAAAACGTAC 0.70858262156

GCGAGATCATGTCATCACGGAG 1.26844158889

GCGAGATCATGTGATAAGAATT 0.64241732473

GCGAGATTAAGTGATAATTTAT 0.295050434684

GCGAGATTACCTGCTTTGGATT 0.719882507716

GCGAGATTCATTCTTTCAGGAC 0.654447005174

GCGAGATTCTGTGTTAAGGAAG 0.277781089859

GCGAGCCGATGTGGTAAAGGTG 1.0524137776

GCGAGCGAACATTACGCTTCAC 1.35597990807

GCGAGCGAGGGTGATGTTCTAG 0.729372555194

GCGAGCGTGTCTGATATTGCAA 1.0620007096

GCGAGCGTTAGTGAATCTTCAA 0.772809170183

GCGAGCTAATCACAAATAGTAC 0.645234800169

GCGAGCTCGCCTCGTGCCGCAA 2.49162275498

GCGAGGATCTGTGCCGTCAAAA 1.27125298295

GCGAGGGAGGCACAAGTAAAAT 0.419704608889

GCGAGGTCCTTTCCAACGAATC 0.706119910156

GCGAGTACTTATCAAGATTAAA 0.544366318049

GCGAGTCAAGCTGCGTCGGGTG 1.0887885698

GCGAGTCAATATCCTCGCGAAT 0.493279294846

GCGAGTCCGTGTGATGTTGCAG 2.12780895196

GCGAGTCTACGTCCAAGGTCAT 0.378578437506

GCGAGTGAATGTGATGATAAAG 0.321688089181

GCGAGTGACTCTGTACTCGTTG 0.849427077731

GCGAGTGCTGCTGATGCGCAAT 0.719202598737

GCGAGTTCGTATGCTATTCAAC 1.04956932981

GCGAGTTTACCTGATTTCGAAG 0.375813435611

GCGAGTTTATCTGTTTTAGTTC 0.855609632519

GCGAGTTTCAGTGTTGTTAAAC 0.310053545605

GCGAGTTTGAATCATTCAAGTA 0.265883623424

GCGATACTCGATGTAAAATAAA 0.45072658077

GCGATACTGTCACAAGGTTAAC 0.566603317765

GCGATACTGTCTCATTGACTAC 0.382244651564

GCGATCCTCACGTCTCGTTTAT 0.274802249962

GCGATGTCCGTGGCTTCGATAC 1.12883047659

GCGATGTCGCAGCATGAAATAA 1.08999476248

GCGATTCACTCTTTTGTATGAG 0.568921923703

GCGATTCGATCTGTTCAAAGAT 0.804334576487

GCGATTTCATGTGGTAAATAAG 0.55059229589

GCGATTTCTTCAGCTGCATTAT 1.63545759142

GCGCAATTGAGCGCTAAGCATG 0.428009772528

GCGCATCGATCTGATAGGAAAA 0.74696491056

GCGCATGAGTGTGTATTTTTTG 0.611386948685

GCGCATGCAGCCGAAGAAGGAC 0.525126285181

GCGCATTAATGTTCACCAATTA 0.62379512178

GCGCCACAGGATGTGGGATTAC 0.907840744495

GCGCCAGTACCCGAGTCTCGTG 0.993750936347

GCGCCAGTATTTCGTCATATAC 0.782304116266

GCGCCATGCTGTGAACGTCAAG 1.03871682448

GCGCCCGAATTTCAATCTAAAA 0.749060491845

GCGCCTCCCTCTACACCGAGAC 0.645680779681

GCGCCTCGGGGTGATGACCAAG 0.859697337773

GCGCCTGGATCTCATTTCGGTG 1.48961178995

GCGCCTTAATCGCATGTTGTTG 1.44454967785

GCGCGACAAGCTGCTAGCAGAA 1.02309758529

GCGCGACACTCACAACTCGTAA 0.78148905986

GCGCGACCATGTGAACTTAAAC 0.42686424772

GCGCGAGAATTTTCACTGCTAC 1.37058320672

GCGCGATCAACTGCAATACAAG 0.570144649897

GCGCGATCATCTGCTTGAGTAC 0.466967012434

GCGCGCTAAATTCAACGGGAAA 0.267549230721

GCGCGCTCATGTCCAGTCTGAC 1.901604305

GCGCGGCAAACTCATGTATAAG 1.71102619851

GCGCGGTAACGTGATGTTTAAG 0.822255635116

GCGCGTCAGATTGGCAAGTCAA 1.18256979889

GCGCGTGACTGTCCTAGTGATG 1.22185886775

GCGCGTGCCTCTTCTTGATCAG 1.36268306327

GCGCGTGTCGCTGGATTGTAAA 1.49781124256

GCGCGTTAATATCATGATGAAT 0.626092045109

GCGCGTTTGCCTATTTATTAAA 0.510164028934

GCGCGTTTGTATCTTTATCAAG 0.659823498132

GCGCTACCGTCGCACAATGAAG 1.254554792

GCGCTAGAGAGTGAATGGTCAA 0.48089220899

GCGCTGGTATTTTTTACGCTAT 0.386665537252

GCGCTTCACGATAATTAGGATG 1.36808281588

GCGGAAGTCTCTCGTTCGTCAC 0.428745041769

GCGGATCAAATTGGTACGATTG 0.652682350332

GCGGATGGATCTCATTCTTGTT 1.21961027988

GCGGCACCTACTCAATGTAAAA 0.490945634864

GCGGCACTGGCTGGTTCCAAAT 1.04533817408

GCGGCAGTATATCTTACTTTAG 0.455081381122

GCGGCATAACCTGCTAGGCGAA 0.216224658459

GCGGCCGCGTATGCACCTGTAT 0.828737577829

GCGGCGGCTGGTGTATCAGTAC 1.59804234933

GCGGCGTGCCATCAAATTCAAG 1.60799456241

GCGGCGTTAGCTTCTTGAATAG 0.371361848083

GCGGCTGACGCTCCTGCAGGTA 1.02013622583

GCGGCTTAAAGTGACAGATAAA 0.92723106283

GCGGCTTCAAGTTTTCCACGAT 0.469403932035

GCGGGACTCTCAGCACTAATTA 1.10859653638

GCGGGAGACACTCCGTAAACAC 0.960514578633

GCGGGAGTATAGGGTCTAACTA 0.497920036224

GCGGGATCATGTTAACGGCAAG 0.344151664543

GCGGGCTCGACTCTTATTTGAC 0.908780587557

GCGGGGTAGTATTAAGCTCAAT 0.52983011938

GCGGGTCATTGTCATCAGACAT 1.36849018484

GCGGGTCTACCTCGCCTGCAAT 0.505552762472

GCGGGTTAACTTCTTTTCATAA 0.523906313536

GCGGTACGATGCGGAGTACATG 0.203879568365

GCGGTCTAGCGTGCTAACATAC 0.899395279999

GCGTAAATTAATGAGGAATTAG 0.355207780025

GCGTAACAGTATCATTATAGAG 0.389860889388

GCGTAAGCATGAGCACAGCTAC 0.835929699837

GCGTATGAACGTGGTCAGTAAG 0.593013564609

GCGTATGCACCTGCAGTGAAAA 0.222851030835

GCGTATGGGCTTCCTTAGGGAT 0.78362509396

GCGTCACAATCTCAAAAGATAG 0.426299768058

GCGTCACGGGGTGACAAAATAG 0.654858992895

GCGTCAGACAAACATTATCAAC 0.197322447524

GCGTCAGGAGTTGAAGCGAGTA 0.659485641372

GCGTCAGGCTCTGCTGAACGAG 0.701628716991

GCGTCAGTGTCTGTACTGAGAC 1.43566059729

GCGTCATAAGCTTTATTCAAAT 0.410106582272

GCGTCATAATGTGTTTGGTGAT 0.717335677429

GCGTCATACCGTCATTCATAAC 0.688982005456

GCGTCATACTATCTTGGTGCAT 0.503752083741

GCGTCATCCCGCGTAAGGAAAC 0.618367090775

GCGTCATCGTCTCCTTCGTAAC 0.879164188819

GCGTCATGATCTCAGCTATATA 0.479178947736

GCGTCCCTCTGTGCTACTTTAC 0.429569552863

GCGTCCGTAGGTCCTAGACATT 0.280734834624

GCGTCCGTATGTGATTCTTAAC 0.665792278806

GCGTCCTTCCCTGCACTCATTT 0.866680702272

GCGTCGCAGTATGGACAAGAAC 1.27245654402

GCGTCGGTAGCTCATAATGGAA 0.800751598623

GCGTCGTTATCTCCTGCATTAA 0.917770344616

GCGTCTGGCACTGATGGTGATC 0.65039573479

GCGTCTTCGTGTCGGTGCTGAG 1.36292183618

GCGTCTTGAAACGCGACGCCAA 0.915554265406

GCGTCTTTCTGGAATAAACTTA 0.595584620164

GCGTGAAAAGATCTTTAGAGAG 0.525990543655

GCGTGAAAAGCTACGATATATG 0.895056786079

GCGTGACGAACTCTTCGTTAAC 0.578410667898

GCGTGAGAAACCGATAATCATT 0.731650757704

GCGTGAGACAGTCCCGCCGGAT 2.17753660535

GCGTGAGAGTCTGAAGCCGTAA 2.50613339604

GCGTGAGAGTCTGAATAGATAC 0.461599845014

GCGTGAGATACTTCTTTGTGAG 0.562418059983

GCGTGAGTACTACATGCTTCAC 0.500177063472

GCGTGAGTGTTTGATGGAGATT 0.730204009204

GCGTGATAATGAGCTGGAGGAG 0.708735566885

GCGTGATACAGTCCTAAGGAAC 0.535841518546

GCGTGATCGTCGCATTTAGTAC 0.761360101367

GCGTGGCACACTGTCTATCAAA 0.824765636996

GCGTGGGACGGTGGTAACTGAG 1.12516206175

GCGTGTACATGTGCTCCGTGTC 1.33136714968

GCGTGTCAGTCTCCTGCTATTC 0.926306925049

GCGTGTCGGGCACATGTTCAAG 0.687104964605

GCGTGTGACCGAGATTTGTAAT 0.485090355318

GCGTGTGAGAATGAAGCCGTAG 1.4926956984

GCGTGTGTATCTCCAGTAGGAG 0.928857023882

GCGTGTGTCTGTGAAGATGGTA 1.73080478336

GCGTGTTAAGTTCAAATTCTAG 0.581369385687

GCGTGTTAGTCGGCATAGAATG 0.693149937948

GCGTGTTCACATGAAGCTCTTT 0.575569088838

GCGTGTTGATGTGGTGGAGAAG 2.15831820785

GCGTTACAATGTGTGGTAAGAT 1.34383293941

GCGTTAGCATATGGTCTTCCAA 0.330156747564

GCGTTGCAGTCTCGTCTCTATG 0.924481304684

GCGTTTCACTCTGCAGATTCAA 0.507890890282

GCGTTTGAATGTGAGTCTTTAC 0.689333274812

GCGTTTGCCTATGCGCCCAAAG 0.594822633415

GCGTTTGGTTCTGCCTAGGGTA 1.87086375023

GCTAAAGACGCTGTTGCGATAG 0.56641185404

GCTAAAGTCTCTCTTGGGTAAT 0.682950404422

GCTAAATTCTGTGAATCGAAAG 0.632780823408

GCTACACACTTAGCAACGGAAG 0.336219248594

GCTACACAGCGTCGTATTACAC 0.525235213801

GCTACAGACATTCATAGTCTAA 0.356483716658

GCTACAGCCTTTCCAACTATAG 0.481792036194

GCTACATCGACTGCCCTTACAT 0.914519313152

GCTACATTGTATCCTAGTTAAA 0.596652062902

GCTACCTAAGCTCATGGTTATA 0.466801053274

GCTACGCATTCTGCTTTGTTAG 0.275205167199

GCTACGGAATCTGTAGAAGAAG 0.570490130535

GCTACGTCGGTTCCTGAGTAAA 0.904959095013

GCTACTCGGTGTGAATACTGAG 0.780267313607

GCTAGACAAACTCCAAAGCCAG 0.301238736589

GCTAGACAAACTGCAATAACAC 0.329138387504

GCTAGACGATTTTGTCTCAAAC 0.307329748869

GCTAGAGGGTCTCATCGATCAG 0.995291223519

GCTAGATTGTGTGCATGGAAAG 0.450522847062

GCTAGCCTCTGTGATAAGCAAC 0.468033364708

GCTAGGCTCCTGCCACAACAAT 0.886343457402

GCTAGGGAAGGTCGAAAACTAG 0.349437282901

GCTAGGGTTTTTGGCTGGGGTG 1.54616351602

GCTAGGTACAGCGGCGAAGAAA 0.854453387306

GCTAGTCCCTCGGGATATCTAG 0.838404774605

GCTAGTGCGAGTCTGCACGGTA 1.44409505105

GCTAGTGTCTCTGATTTATTAC 0.337992068637

GCTATAGAATGTGACCCCGGAA 0.753701212107

GCTATTCTCTGTGATCCCTTAC 0.591340848882

GCTATTGTCGTTGGTGTACATC 0.734334445276

GCTCAGGTGAGTTACCTAGTAT 1.46961924119

GCTCCACACTGTGAAGCAACAC 0.567360136894

GCTCCAGGATCTGCAAATCGAC 0.679285042507

GCTCCATCATGACCTCCGATAC 0.986994087059

GCTCCATGGTATGGTATCTCAT 1.08363695538

GCTCCGTGTGGTGATTGCTGAG 1.59478681513

GCTCCTCAGACTTTTTGTACAT 0.755000043535

GCTCCTCAGCACCTTAGTTCAA 0.315821136642

GCTCGAATATCTCTGAAAATAA 0.0281210123719

GCTCGACAAGTTGACACCTTAT 0.887975988089

GCTCGACCAGGTTATATTTTAC 0.576265129406

GCTCGGCAGTCTCATAGAAGAA 0.972708079041

GCTCGTCTTTTTGCACCAAGAG 0.742905485788

GCTCTACACAATCCAACAGAAG 0.478604323376

GCTCTCTAACTCGGTGATTAAG 0.861144309421

GCTCTTGTGTGTGCGTGAGAAT 1.49656930513

GCTGAATACTGTCATCAAAAAG 3.40281827938

GCTGACCATTATGAAGAGAATG 1.69012269835

GCTGATCAAGCTGCGGCCCAAT 0.787868582128

GCTGATTGATGTGTAGCCGATT 3.53907440034

GCTGCATACGCGTGAGCGACAC 1.71039906471

GCTGCCCAGGTTGCTACCTGAT 0.315905489365

GCTGCGTAACATGCAAGTTTAA 0.0450779785984

GCTGCTGTAGGTGCTCCGGAAA 3.08332023263

GCTGCTTAATTGGCAGAGGAAA 2.80968937217

GCTGCTTTATGCCGTGATTAAT 2.41492226621

GCTGGAATCCGTGAATCCGATT 3.1456434435

GCTGGAGACCCAGCGTACAAAA 0.485147812831

GCTGGTGCATGTCCTTTCGGAG 1.88552555814

GCTGGTGTAGGTGATGTTCCTC 2.57928794067

GCTGGTTTCGGTGCTATGACAG 1.56279743076

GCTTAAGAAAGCCGTCTGGAAG 0.446805920936

GCTTAATCCGGCCATTATAAAA 0.456818991445

GCTTATTTACGTCCTAGAAGAG 0.306868473201

GCTTCACTTTCTAATAGTAAAT 0.429052224085

GCTTCAGGACCTGCATGTTCTG 0.443979019179

GCTTCATCCTATGATGGTTGAG 0.275594577279

GCTTCCTCGGATGTGTGAAAAC 0.367971408785

GCTTCGCGAACTTATATCCATT 0.393233488698

GCTTCTGTGCTTGATTGATAAA 0.818042352749

GCTTCTGTGTGTGCCAAGTAAG 0.399135327762

GCTTCTTGGATTGGTTAGCCAT 1.15465605675

GCTTCTTTATATCAAGCAGCAG 0.187243470752

GCTTGACACTGCCCCTCTAAAA 1.4197738612

GCTTGACAGAGTCTGGATATTT 0.630399402611

GCTTGACCCTCTCATAGATAAG 0.528522291956

GCTTGAGACGCTCCGTCTAATA 0.665017865098

GCTTGAGCACCTCCACTTTGAG 0.447758774275

GCTTGGAGATCTGCCTGCTAAA 0.773628540768

GCTTGGGTATGTGATTCTTAAA 0.97607842805

GCTTGGTGGTCGGTGGTAAATC 1.17629028007

GCTTGTCCAAGTTCTGGCGAAA 0.52522900479

GCTTGTCCGGGTTGTGTGGCAA 1.31306915519

GCTTGTCTATCTGATAGTCAAG 0.524817629054

GCTTGTGACTGTGCTCAGGTAA 1.09901087908

GCTTGTGTGTAGCCTTTATTAA 0.659118799946

GCTTGTTAATCGCTAGTACAAT 0.553482578854

GCTTGTTAATCTCTGCGCTGAG 0.47216750875

GCTTGTTAATGTGCAAATCCAG 0.446198070581

GCTTGTTCATAAGCTAGCAAAG 0.239753235709

GCTTTACAACGTGTTCACGTTA 1.52271311679

GCTTTTGGATCTCGTGAGAAAG 0.556532732376

GGAACACAGTCTTAGTCCTCAC 0.540596674568

GGAACAGAATCTCTTAATCAAA 0.379390529243

GGAACAGATTTTCATTGCCCTA 0.521645812378

GGAACAGCATCTCCCAGTCTAA 0.380328481245

GGAACAGTGTCTGGTGATTAAA 1.10212868198

GGAAGATCAAGTCAAGGGGGAT 0.541560997806

GGAAGTTAATCGGGATAGATAG 0.721666498674

GGAAGTTATTGTGATAGATGAT 0.260907319032

GGAATAACATCTCCTGGAAGAA 0.6159869018

GGACCGGTTACTGGCACAGGTC 1.57815110255

GGACCTTTGGATGATCGGGATA 0.470910606407

GGACTTCCATCTGACTAGTTAA 0.510498236499

GGAGCCCGCTCTGATAACACAA 0.222818924924

GGAGCCGTATCCTGATTATAAG 1.87505390304

GGAGGAGGTCGTCTTTGGAATG 1.37992394722

GGATCTTGCCCTGCATGAGCAC 0.971166376812

GGCAAAAAGTTCCCAGGATGAC 0.420953671742

GGCAAAATGTCTGAAAAGTCTT 0.47209697663

GGCAAACGATGTCCAAAATCAG 0.997186577333

GGCAAACGCTGGCTAGGGCAAC 1.65870507802

GGCAAAGGTGCTCCGGTTAGAA 0.43122332151

GGCAAATTATGTCTCAACTTAT 0.2413345137

GGCAACCTACCTCAAGTTAAAG 0.78869091354

GGCAACGTATGGGCTAACAAAT 1.07271483664

GGCAAGATGCCTCATACTATAT 0.536379144862

GGCAATCATTCTGTCAGTGTAA 0.435036762071

GGCAATGAATCTGAATCAATAA 0.668353358208

GGCAATGTCCCTGATGTTTCAG 0.853652139375

GGCAATTGAACTCCTAGCTAAC 0.605287669051

GGCAATTTAAGTGGGACTCGAG 1.30937707913

GGCAATTTATATCACGTTTAAA 0.524337328297

GGCACAAATTTTCATGAGAATA 0.458761181909

GGCACACAAAATTGTTATTAAA 0.499446240246

GGCACACAACCTTGACGCTAAG 0.252578041789

GGCACACACTTTCCAATATAAC 0.293724988196

GGCACACAGTGTCGAAAGGATG 0.642086415139

GGCACACCAACGGTTTGGAAAA 1.66301590812

GGCACACCAACTCATTCTAGTC 0.147520424678

GGCACACCATGTGCTATTTAAC 0.598450754338

GGCACACCCGATGCTTGTCAAA 0.828899575884

GGCACACGCAGTTTTTGGGAAG 0.818897122943

GGCACAGAATCTGATTTAGGAC 0.596019303917

GGCACAGATAAGCGTTGGATAA 0.49293882552

GGCACAGGAGGTCCTACTGAAA 0.536333119377

GGCACAGGATGTTCTCATACAA 0.918738450743

GGCACAGGGTGACACTTGAGTA 1.89388052057

GGCACATAATCGGCTCGCCCAA 1.17272376595

GGCACATACGATTATCACCTTA 0.462090559625

GGCACATAGCCTCGATGTGTAT 1.02392764041

GGCACATTGAGTGGACTCCGTA 1.08419924578

GGCACCCACGCCCTATTCCGAG 2.38010032281

GGCACCCACTATCCTACTGTTC 0.639154951671

GGCACCTAACCTGGTAGCATAA 0.452216079755

GGCACCTTTCCTGCTGTTTAAC 0.582493129228

GGCACGCCTCCGCATGAGATAA 1.49061206807

GGCACGCTCTCACACCGTAAAA 0.616240465581

GGCACGGTATGTGACAGAAGAT 1.17457861818

GGCACGGTGTATCCGTGCTTAT 0.65020215919

GGCACGTTTGCTCGCGGCGGAT 1.63920721002

GGCACTCACTGTCCTAGTTATG 0.885097416111

GGCACTCGATTTGAATAGAAAC 0.840322764811

GGCACTCTATTGTTTTATCGAA 0.680587360814

GGCACTCTCCCTCGGGGGGTAC 1.39955823485

GGCACTGAACGTTGATCAGCAA 0.391560575703

GGCACTGACCATGATTATATTT 0.594923477927

GGCACTGACTCTGTTAGGACAA 0.310613542781

GGCACTGAGTTAGCCGTATCAG 0.86889447558

GGCACTGGACATGCTCGAAGAT 0.68699433871

GGCACTGTATTTGCGCCTACTC 0.438250553268

GGCACTTCAACGGATCCGAAAT 0.997460479076

GGCACTTTAACTGCTGCTAAAG 0.696568042289

GGCACTTTCTTTGCTACAGCAA 0.491120092937

GGCACTTTGATTGCGCGGGCAG 2.15405204147

GGCACTTTGCGTTCCCGGGAAT 1.23681593748

GGCAGACAGTATCCAGACACAT 0.801236854998

GGCAGACCAGCTCATTTATCAG 0.30306697814

GGCAGACCCGCGCTCAACGTAG 1.98979100094

GGCAGACGAATTGTTAAGGTTT 0.771669647385

GGCAGACGTTGGGCTTGATAAG 0.458051831206

GGCAGACTCAATGTTAGAGTAA 0.530973510878

GGCAGACTCGGTCTTATGTCTT 0.597992402801

GGCAGACTGGTACCCCGCTTTG 0.341073879994

GGCAGAGAATATCTGCACCTAG 0.637961859724

GGCAGAGACTCTCAAAAGCCAC 0.965140028332

GGCAGAGCAAGTCAGTGTGAAT 0.515280985743

GGCAGAGCGAGCGTAAACGGTG 1.22263397377

GGCAGAGCGTGGCGTGCAAGAG 1.07554246902

GGCAGAGGGGCTGAGGAGGAAT 1.47952237442

GGCAGAGTAGGTCATAAATGTC 1.18819184937

GGCAGAGTCCCTGATTAGCGTG 0.280804431366

GGCAGATAATCATATGGAGAAA 0.505083664363

GGCAGATGGTCACTAGCGGGAC 0.0945077440596

GGCAGCATGTGCCCGGTGGGAG 3.04089632895

GGCAGCCATTGATATAGCGAAT 0.586697480762

GGCAGCCCGATTTTTATGTATG 0.381991236004

GGCAGCGAATGTGCATCATGAG 0.225733856124

GGCAGCGACAGTGCTAGGCGAG 1.60422497743

GGCAGCGCGAAACATTCGTTAG 1.3376640223

GGCAGGAGAGATGAAAGATAAG 1.15958520653

GGCAGGCAAGCTCTATAAACAC 1.73603659163

GGCAGGGAGCATGCTCTGAGAG 0.315905489365

GGCAGTAGCTGTGCTTCATCAA 0.407030498827

GGCAGTCACTCTTATGATAATA 0.506446347815

GGCAGTCCACGTCGTGCATCTG 0.282366253431

GGCAGTCCATTTGATGTGCAAT 1.00779912017

GGCAGTCTATATTAAACTGTAC 0.561621654099

GGCAGTGACTATCATTAGACAC 1.2134264046

GGCAGTGACTCTCGTCCTTTAC 0.328505522985

GGCAGTGAGCGTGCATACCAAG 1.05920426279

GGCAGTGATACTCCTCGTTAAG 0.414406525095

GGCAGTGATGGTGAAACATGAA 0.884311021268

GGCAGTGATTCTCTATTAACAT 0.343976980602

GGCAGTGGGTTTCTATTAAAAA 0.839623044831

GGCAGTTTATCGCCTAACTAAA 0.345865455647

GGCAGTTTATGTGATGGGTAAA 1.00620273187

GGCATAGAAATTGACCCGGTAC 0.214609370501

GGCATAGAATCTCAGTACCAAA 0.317970337333

GGCATAGACTGTCATGGAACAA 0.555096279912

GGCATCCAATGTGTTTCATTAT 0.268922889362

GGCATCCCCACTCATCCGACAG 0.70599092435

GGCATTAGCGTTCCTGCACTAG 0.817740409748

GGCATTCGTAGTCATACCGAAA 0.39335420088

GGCATTCTAAGTGAAAGTGCAT 0.341402407359

GGCCAAGCATCCCAAACGAGAT 0.350667297769

GGCCAATGTCCTCGTACATCAA 0.42237223901

GGCCAGTAAGCTGGAAAGATAG 0.762120452893

GGCCATCCCTCTGAATTGTCTC 0.542161375654

GGCCATGAGTGTCAAAAAACAT 0.639792008238

GGCCATTAACGTACATCTGGAC 0.693989245719

GGCCCACCAGGTGATAGCAAAA 0.545716932631

GGCCCAGCCCCTGCTGCCTTAA 3.33651147488

GGCCCAGCGTATGCGCCTTCAA 0.677684545712

GGCCCAGCTAATGCTTACAAAC 0.705651634627

GGCCCAGGTTATGTTGAGTAAC 1.06277173286

GGCCCATAAGCATCTGCAAGAG 0.896467917675

GGCCCATAATCTGCAACAAATG 0.356349243506

GGCCCATAATGTGCGTAAAAAA 0.239795288956

GGCCCATGATCTGCCTCACGAA 1.02487257918

GGCCCCCAATCTGGATTTAGAG 0.8674094234

GGCCCCCCAGATGTAGCCCCAG 1.41131428028

GGCCCCTGCTGTGAAGATATTG 0.852898893081

GGCCCGGGATCTCATGAGAGAT 0.700409862893

GGCCCGTGCTCTCAGGTCGGTG 0.84185138836

GGCCCTGATTGTGAAAAGTATG 0.733514577291

GGCCCTGCAACTGTTAATAGAG 0.656872462117

GGCCCTGGATATAGTAAGCCAG 0.532944194351

GGCCCTGTAGATCAAATTCTAG 0.91139282951

GGCCCTTACAATGCATATATAC 0.386368336251

GGCCCTTTCAATCCAAAAGTAG 0.188614733058

GGCCGAGAATCTTTGAAAAAAG 0.691079517251

GGCCGAGACTGCCATATAGGTC 0.841774047586

GGCCGAGGATTTCCCATCGGTT 0.893141980641

GGCCGATACTGTCCTTATTATC 0.770395997553

GGCCGATCATCTGCTCCGGAAT 0.2755028825

GGCCGCGCTTGTGATGTAATAT 1.84688430706

GGCCGCTTGATCCTGATAATAA 0.448267155954

GGCCGGCACTCTGATTGACAAG 1.21889121047

GGCCGGGGACGTTCACTAAGAT 0.485012462081

GGCCGGTGATGAGGTCATAAAG 0.653943460062

GGCCGTAAATCTGTATGCCGAA 0.651403112384

GGCCGTCGCTCAGCTTCCTAAT 1.04126727806

GGCCGTGAGTGCGCTACATATG 1.16070789128

GGCCGTGGGAGTCCAGCTAGAT 2.21864338899

GGCCGTGTGTATGCCCCAATAA 1.04504649681

GGCCGTGTTTCTGCAAGTCATC 2.12839249089

GGCCGTTAGAGTCACTGTGAAA 0.835217939462

GGCCGTTTATCTGTTAGGTAAC 0.278037153276

GGCCGTTTCTGTGCTACTCGAT 1.03608711

GGCCGTTTGAGGCAATGCGGTA 1.70439697184

GGCCTACAATCTGCTATAGAAA 0.405679407742

GGCCTAGACCATGCTGCCACAC 1.29458051777

GGCCTAGTACCTCTTAATTAAT 1.13199220714

GGCCTAGTCGGTGACGGTAATT 0.605463415467

GGCCTAGTTTGCGTACAATCTC 0.460633804397

GGCCTCCGTTGTGTAAACAATA 0.590334444685

GGCCTTCAATCGCTACTACGAT 0.586561767391

GGCCTTCAGTCTCATGCAGCAT 1.16010699799

GGCCTTCTTTGTCTTCATAAAT 0.658929471374

GGCGAACACTGTGTAAGGACAG 0.49938214223

GGCGAACGACTTGATGTGAAAA 0.343625611111

GGCGAAGAAAATGGGCGTTTAG 1.78628141065

GGCGAATTCACTGCTGCTGTAG 1.1759994595

GGCGACCAGGCTCATTAATCAT 1.22828045379

GGCGCACTGAGTTCTTGACGAA 0.271771827954

GGCGCCCCTAGTTCTAAGGGAG 1.28241152633

GGCGCCCGTCGTGCTGTTGGAT 0.75911747758

GGCGCCGCCAGTGCACCGTTAC 1.41037525751

GGCGCCGCGTCAGACTGGATAA 0.658051196505

GGCGCCTAATCTCCGAAAGAAT 0.477678478715

GGCGCCTCACCTTCTACAATAT 1.2999024991

GGCGCGGATTCACCAGAAAGAT 0.431013836919

GGCGCTAAATATCCTAAGTGTT 0.32327013638

GGCGCTAAATTTGTTTGTCTAC 0.577392574684

GGCGCTCCATGTCCATCAGAAC 1.07788342845

GGCGCTGAATCTTGATCCGTAC 0.936842718243

GGCGGACTCTCTCTAGAACAAA 0.716702337823

GGCGGAGCAATTGCCGGCTAAA 0.943188662458

GGCGGAGCGGTTCCATGAGAAT 0.854256238175

GGCGGAGTCTCTCTCGATTAAA 0.758242029553

GGCGGATGATTTCTTAGAAAAG 0.753623935764

GGCGGATGTCCTGATATGATAA 0.47276939006

GGCGGCCTATCTCAAGTGTATC 2.20449944568

GGCGGCGCAAGTGCCCAATAAT 3.60533949966

GGCGGCGGAACTGCTAGTCCAG 5.26560059676

GGCGGTCGGTCGCTTTAACAAT 0.95789526124

GGCGGTCGGTCTGAGACTGCAA 1.79195756765

GGCGGTGAGTCTACACGCGAAG 0.664750821607

GGCGGTGTCACTTCTCAGGCAC 1.54438651736

GGCGGTGTGCCTGATAGCCTAG 1.54983826995

GGCGTACATTGCGGTCCTCAAC 0.314935366092

GGCGTACCCTGTCGATTTGTTC 1.34946882183

GGCGTATAGTCTGCTCTCTGAA 1.18247571015

GGCGTTCACACTCACGGATAAC 0.985483187376

GGCGTTCCATCTCCCAGGAAAG 1.62670703206

GGCGTTCTCTCTGATATTATTG 0.641168743187

GGCTAACGAACTGAAAGTGGAA 0.909266903448

GGCTAACTATGTTCTTACGGAA 0.455993132899

GGCTAAGAAGGTCCTGGCAAAC 0.54623721852

GGCTACGAGTCTGCTTGACTAG 0.440556851795

GGCTAGTCGTCGGTTCCGAAAG 1.57797750966

GGCTATCGATGTCTTTTAGGAT 1.28463295145

GGCTATCGTTGTGGAAATTGAG 0.23436573601

GGCTATGTGTCTCCTCTCAAAT 0.642737169139

GGCTATTAGTGTGATATTGTAA 0.392113191764

GGCTATTGTTCATCGAGTTATT 0.4316618253

GGCTCACCGGCTCCAATCAGAT 1.26508366314

GGCTCACGACGTTCAAATTTAG 0.985018918143

GGCTCACGCGGTGTATGTATAA 1.08913087658

GGCTCAGAATGGGTTGGTCTAA 1.27271973157

GGCTCAGATGCGGAAGATTTAG 0.345646444977

GGCTCAGCATCTTATCTCCGCT 0.610637390443

GGCTCAGCTACTGATAACAAAG 0.446995618452

GGCTCAGGAGCTCCTGAGCCAT 1.57407767772

GGCTCAGGCTCTGAAAGGAAAA 0.415099162226

GGCTCATAATGTGACCGTGAAA 0.321019105721

GGCTCATCACCTGATCCTAAAC 0.331555475274

GGCTCATTTATTGCCTCGCGAA 0.930649682436

GGCTCCCCATCGTCGAAGTTAT 0.652736393425

GGCTCCTTATGTGCTGCACAAT 1.5323160663

GGCTCGCAATCCTGAACTAGAG 1.31854625932

GGCTCGGAAATTTACGGGGCAC 1.81707384009

GGCTCGGAATTTCGTAATCCAC 0.771970082513

GGCTCGGCTTATGGTAACTTAT 1.98781873389

GGCTCGGTGTGTCCATGGAAAG 3.12452401504

GGCTCGTACCGTGCACAATGAT 1.64080045665

GGCTCGTACTCTGCAAATGATA 0.457098665444

GGCTCTCGATAGTCGTATAATG 0.443588148662

GGCTCTCTCTGTGTTTCTTAAG 0.301642280356

GGCTCTGAGAATCTACGAATAC 0.30027684643

GGCTCTGATCCTCTAAGGTAAG 1.01331742026

GGCTCTGCGTCTGATGTAAGAG 1.16607935253

GGCTCTGGATTGCGGTTGCGAG 1.35385059891

GGCTCTGGGTGTGAGTTGCCAC 1.7071308258

GGCTCTGTCTCTCAAGGAAAAT 0.258105005704

GGCTGAACCCCCCGTGGCGGAG 0.444659388312

GGCTGAATGAGTAGTGATTGAA 0.547333244358

GGCTGACAAGTTGCATGTTGAG 0.488179770177

GGCTGACGATCTCCTCGCTTAA 0.863363220469

GGCTGACGATGTGGGGACCCTC 2.18733791036

GGCTGACTCCCTGAGCTCTAAG 0.783777476322

GGCTGACTGTCTGCTTCCTATA 1.44031327412

GGCTGACTTAGTTTAGAAGATG 0.587636645151

GGCTGAGAACACCATACTGCAT 0.665006881058

GGCTGAGAATCTCAGGCTAAAT 0.644717927594

GGCTGAGAGGCTGCTAAGTGAG 1.27563212248

GGCTGAGCGTCTCCCGTCAGAA 1.59720842708

GGCTGAGGATCGCGCTAATGTG 2.33482544165

GGCTGAGTTAGGGAAGGGCAAT 1.02651557282

GGCTGATAACGTGTTGGACGTG 1.31592030179

GGCTGATCATATCATGTGTCAC 0.740922787027

GGCTGATCATGTCAAGACTAAC 0.411940873685

GGCTGATTAACGCAATTGATAA 0.79878881015

GGCTGATTAGCTCCATGCGGAA 1.00867960537

GGCTGCCAATCTCATATCTAAA 0.579436458097

GGCTGCCCTTGTGTTGAAAAAT 0.503370000735

GGCTGCGCTCCTGAGTTGATAG 2.0307531159

GGCTGCTAAGTTTCAAGAGAAT 0.350376318483

GGCTGGTAGCATGCAGAATGAT 1.17223922637

GGCTGGTCACATGATGAGTAAC 0.802025968922

GGCTGGTTAGATCATCAGGTAA 1.43345413956

GGCTGGTTGTGTGCAGTATCAA 1.94422396364

GGCTGTCAGTGGCTTCACAAAT 1.14634714915

GGCTGTCTCAATTAACGTTGAC 1.37871075705

GGCTGTCTGTTTCAAGCCCGAG 1.30703476722

GGCTGTGAAGCTGATTAGTTAC 0.448164261418

GGCTGTGACGCTCGCTTACTAA 1.62922367508

GGCTGTGTTTGACCACAGATAC 0.429773540145

GGCTGTTGCACTATGCTTACAG 0.899067144675

GGCTGTTTCTATGACACTAGAG 0.476069320001

GGCTGTTTCTGTGCATTATAAT 0.927451925123

GGCTTATTGTGTGCTACCTTAA 0.50281149239

GGCTTTTTAAATCAAGTCCAAG 0.27207932273

GGGAAATCAATTGTTCTGGTAG 0.699976983309

GGGAAGCACTATGCTGTTGAAG 1.39633498118

GGGAAGCGGCGTTACCGGGAAG 1.07465121721

GGGAAGGTAACTCCATAATAAA 0.527645582084

GGGAATCAAGGTGAATGTAAAC 0.924667083971

GGGAATCAATCTGGAATATGAC 0.546215151102

GGGAATGAATAAGATTAATTAT 0.582988548234

GGGACACAACTTCTTTACTAAG 0.40171638195

GGGACACACCCTCGTATGCGAG 0.282035102505

GGGACACCTTCACCTACAATAA 0.937626356118

GGGACACTGGGTGATTAAATAG 0.596231672401

GGGACAGGCCCTCCTACATCTC 0.800261859913

GGGACAGGGGATGGACGTCGAT 1.45165718275

GGGACAGTCAGTGCTGAATAAA 0.455947007367

GGGACAGTTTGACCAGGTTAAG 1.11376868398

GGGACATAATATCCCTATCTAG 0.458441038246

GGGACATAGTTTCATGGATCAC 0.579769066093

GGGACATATTCCGCTTGTATAG 0.317991406694

GGGACATCGAGTGCTTTCCATA 0.326962803167

GGGACATTCTGTCGTTCTGGAC 1.32475732767

GGGACATTCTGTTAAGAAGCAG 0.85588572376

GGGACATTGAATGCTCAATTAT 0.526495388027

GGGACATTGCCTGCTGATTGTA 0.544768529883

GGGACATTTACTGAATACTAAG 0.540462912774

GGGACCCATGTTGCTGCAGAAT 0.995288858216

GGGACCCGCTTTGCTCCAGAAT 0.75075626774

GGGACCTAATGTCCTAATTAAT 0.63287252929

GGGACCTATACTCAACCTCTAA 0.388473904752

GGGACCTTGCCTGTAAACGAAC 0.449553111365

GGGACGCTTGCCCCTGCGATAT 0.404002230681

GGGACGTACTCGGCTCCGTGAG 0.309469881806

GGGACTAGATCTCTGACATCTG 0.715865251865

GGGACTATGGGTCATAACTCAG 0.748327117627

GGGACTCGTAAGGCAATAGAAA 0.440128992065

GGGACTCTATATTCGAGAGAAA 0.889103090848

GGGACTCTATCTCCTACTGAAC 0.333358703247

GGGACTCTCGCTGCTCCACTAG 0.659024041739

GGGACTCTGGGTCATTAGCGAC 0.618580224133

GGGACTGCATATACCAGTATTG 0.583403062816

GGGACTGCGTCAGGCTGAACAG 1.12246648215

GGGACTGGCTGTCTCCGTGCAG 0.526685729039

GGGACTGTATCTCATATATTAT 0.816799070203

GGGACTGTATCTGCATAATTAA 0.462754189044

GGGACTTTTTCTGATTATTATA 0.669950199932

GGGAGAACATCTCCTGGATAAG 0.819554353429

GGGAGACTGTCTTCTTGTTAAG 0.567337089589

GGGAGAGCATCTGTTTAGATAC 0.628458374992

GGGAGATAACCTCCAGCTTAAC 0.459585233091

GGGAGATATACTGGAGAGCAAT 0.391096083339

GGGAGATCTGGTCGTACGCCAG 0.582876216221

GGGAGATTCGGTCATGAGTCAC 1.09668926851

GGGAGATTGAGTTATGGGTCAT 0.597794698872

GGGAGATTGTCCCTTTAAGAAT 0.361760052948

GGGAGATTTGGAGTAGGCGCTG 1.30214223989

GGGAGCCCATCTGATGCACATG 1.81757093573

GGGAGCGAGCCGAACCCAATAG 0.592381994092

GGGAGCGCATTTGCGGCAAAAC 2.04968290623

GGGAGCGCTAGTGAATCTCAAC 0.680939053812

GGGAGCTAATTTGGAATGATAA 0.278428386087

GGGAGCTCATGTGCCCAGCTAG 1.67051245361

GGGAGGAGAAGTGCAAGCGGAG 1.13247260942

GGGAGGGGATCTCCTGAGTAAT 0.965363779699

GGGAGGGTAAGTGCTCGGAAAA 1.38497837326

GGGAGGGTTAGTTGTGCAATAC 0.989684178654

GGGAGGTTACCTCATGTTAAAT 0.633322224642

GGGAGTACGTTGGGTCCCTTTG 0.45478846511

GGGAGTCAGCGTCAATCGATAC 0.669346825674

GGGAGTCCGTGTGATGGAAAAG 1.70159078342

GGGAGTCTCTCTGATGTTGAAG 1.24502571138

GGGAGTGACTCTGCACAAGCAT 1.07497269028

GGGAGTTAATCTCAATGAAAAG 0.34417565181

GGGAGTTATGGTCCTGCAGATC 1.4313070492

GGGAGTTGAAGCCCTCCATCAG 0.987353968061

GGGAGTTGAAGTCGAATTGCAC 0.393025981761

GGGAGTTGATTTCCTTTATTTT 1.52661196952

GGGAGTTGGCGTGGTAACCGAC 0.750873302347

GGGAGTTTATGTCATCTGCCAG 1.08054135034

GGGAGTTTGGTGCCTGATACAC 0.20499668562

GGGATACGCTGTGCGGCCTAAG 1.31414797533

GGGATAGAGCGGACTCGCTAAA 0.831942630994

GGGATAGCAGCTCCTCGGCAAA 1.4901588479

GGGATCGACCATTCTTACTTAG 0.399666330367

GGGATCGCATATGGAGGTTTTT 0.896719072978

GGGATGGCAGTTCCTAGGTGAT 1.12016728131

GGGATTACAATTGCACGAATAC 0.434607664217

GGGATTTCTTGAGCGAACTCAG 1.7799507259

GGGCACCGAACTGCCCTAGAAC 1.21520682942

GGGCATGTCAGTGTTAGATTAG 1.16783608081

GGGCCACATTCATCCAGGAAAG 0.661655537997

GGGCCAGAATCTGGAAGTTCAA 0.729664063841

GGGCCAGCAGAGCTTAGGGAAG 1.4811872966

GGGCCATAATCTCGTACCAAAA 0.66527069781

GGGCCATCACCTCAAATATCAC 0.274842119918

GGGCCATTAAATGATTGAACAG 0.606959754263

GGGCCATTAATTCTTCACCATG 0.355703619494

GGGCCCCTTGCACCTGACCAAG 1.33395474088

GGGCCCGATTTTGCTGGAGCTG 1.44732304646

GGGCCCGGCATTCTGGCTAAAG 1.58749067382

GGGCCGTTGGCGGATCGCACAT 2.26757583812

GGGCCTCGATCATTTTAAGGTC 1.18150054937

GGGCCTGAAGCAGCTTAGAAAA 0.409608140226

GGGCCTGACCACGCCATACAAA 1.4145623091

GGGCCTGTCGGGCCAAATTGAT 1.30842894754

GGGCCTTTAAGCCTCCTCACAA 0.628654695623

GGGCGACAGCGGCCTATTACTC 1.47278231764

GGGCGACTCTTTGTGGCCTTTT 1.70437124066

GGGCGACTTTGTGCACCGCGAA 1.39189060066

GGGCGAGCCACTCATATTTCAC 0.446299670815

GGGCGAGGGGGTCAAGTATAAT 0.913792343925

GGGCGAGGGTCTCCACCTACAA 0.457689747645

GGGCGATAAAGTCTCACACAAG 0.755498526903

GGGCGATACGTTGATAACCCAA 0.808471849614

GGGCGATACTCTGCTTGGTAAG 0.845274270323

GGGCGATCAAGTGATTACGCAG 0.183387587694

GGGCGATGAACTCCAATCAAAG 0.867965273453

GGGCGCGTTACGGATAACCTTG 1.17735669114

GGGCGTATTTCTTAAGATAGAT 0.433934445328

GGGCGTCACAGTGCTAAGTAAC 1.418581035

GGGCGTCTATGTTCGGCACCAA 0.272172498763

GGGCGTGTCGACGCTAAAGATA 1.07761580348

GGGCGTTCTTAGGCTGAAGAAC 0.863470756484

GGGCGTTGACGTCCAGGATATG 1.58842496576

GGGCTACTACGAGCTTGAGCAG 0.720310657872

GGGCTTGAACCATCGTAGTAAC 0.93737955868

GGGCTTGACTCTCGCAACGAAG 0.947387243297

GGGGAACATCCAGCAGACTATC 1.10395109386

GGGGAAGACGGCCCTGGTACTG 0.559042057385

GGGGAATAGTCTGCTCGGCTAG 0.842133188628

GGGGACTGATCTGGAAGAAAAT 0.686229408622

GGGGATTTGATTGTTGCGGGAA 0.868717595386

GGGGATTTGCGTGATTCCATAC 1.05066206235

GGGGCACAACGTCCTGAAGGAC 1.17376821312

GGGGCACAATGTTCACCTCCAA 0.572097936499

GGGGCACTATCTCCTAATGCTA 0.342993163521

GGGGCAGAGTGTGATATGATAC 0.848771293624

GGGGCAGGATCTCCTAATGGAT 1.27276010396

GGGGCAGTCACTGATACCACAG 0.451405656921

GGGGCATCGCCACGCGATCTTC 1.77504055325

GGGGCATGAACTCACAGTAAAC 0.306683925668

GGGGCATTATGTGAAGCAGTAG 1.0590384337

GGGGCCGGATGTGTTCATCTTG 2.06854588362

GGGGCGTTAGGTAAAGACGATA 0.510438900631

GGGGCTCCGGATGCATAGGTAG 2.2077969833

GGGGCTGCAAGTGCGTCCACAT 0.46709149558

GGGGCTTACATTCCAGTGCAAC 0.920857633404

GGGGCTTACCCTCCAACTCAAT 0.792982416883

GGGGCTTACTCTGCTATCAAAT 0.494151826522

GGGGGAGAGTATCCTACCTTAA 1.43063916785

GGGGGAGAGTCACAACGGTGAG 0.65983422134

GGGGGAGGTCGCCAGATACAAC 1.0754196052

GGGGGATCTTGTTCTCTATGAG 1.54221545142

GGGGGATGATCTTCCCTTAAAG 0.755832932227

GGGGGCGAATACGAAAATTTAG 0.247159063936

GGGGGTCATTCTGTACTCTCAT 1.33222932627

GGGGGTGTGTGTCCGTGACAAA 0.554497734361

GGGGTAGTAGACGATTGAAAAG 0.477026901504

GGGGTATGAGGTGCTACACTTA 0.772458834068

GGGGTCGCACAAGTATTATTAA 0.628894066337

GGGTAACACCCTCATGTGGCTA 1.62588339019

GGGTAACTATTAGCTACCTCAC 0.778776814084

GGGTAAGAGGCAGCGAGACTAA 0.845710699706

GGGTAATACTCTGATGCTGCAC 0.761944780628

GGGTAATCATCTCCGTCTTGTA 1.14037300741

GGGTAATTAAGTCATTCAAATC 0.659789644686

GGGTACCACGCAGATATGTAAA 1.27555363031

GGGTACCTATGTGCCTAGCAAG 1.73733941832

GGGTACGTCTGCTCAGGTGTAC 1.01185253666

GGGTACTCTTTGGATGGAGTAC 0.533356049901

GGGTATCACAGTGATTGGTAAG 0.729387698642

GGGTCACAAACTCACGTATAAG 0.328819345615

GGGTCACACAGCCATCGTGCAA 0.949411014926

GGGTCACAGCCTGTTACCGTAG 1.43096765487

GGGTCACATTCTGCATACGAAT 0.737796751494

GGGTCACTCTGAGCTGCGTTAG 0.396131697068

GGGTCACTGCTTGGGGAATCAC 0.635388627226

GGGTCAGAATCTGTTCAAGAAA 0.813795945025

GGGTCAGAGTGTCATTATACAA 0.905160731451

GGGTCAGGTTCTCGACGCTTAG 1.66434833011

GGGTCAGTACGTGATTTAGTAG 0.828246479526

GGGTCATACTCTGCGAAACTAG 0.852312250458

GGGTCATCGACCGCGTGTAATG 1.50150188335

GGGTCATTATTTGATATTAAAC 0.34938061912

GGGTCCTTTCCTCGTCCTAAAT 0.657434899837

GGGTCGAGCGTTCGTGCACAAG 1.43928577605

GGGTCGCGCCCTGAGAGTCGAT 1.19142177051

GGGTCTCTATCTGATTATAAAC 0.580411749079

GGGTCTGAATTTTATGTACAAC 0.634051724936

GGGTCTGAGTCTCAGACCGTAC 1.33557610777

GGGTCTGCACTTGCAAAGCCTG 1.43537613876

GGGTCTGCCTTTCGCATTAATC 0.241938959306

GGGTCTTAAACTCAGTGGCATT 0.670559890698

GGGTCTTCATGTTTATGTGAAT 0.61329174425

GGGTCTTGAGTTCTCAGGTCAG 0.911338428856

GGGTCTTGCTTTTCCACAAAAA 0.604458024588

GGGTCTTTATGTACATAAAAAA 0.208323039766

GGGTCTTTCTCAGGCAAGTCAA 0.783898600326

GGGTGACAGTCTGGTTAGTGTA 0.890092356548

GGGTGACGAGATGGACCTTAAT 1.10548121776

GGGTGACGTGATGATCAAGTAA 1.25469478439

GGGTGAGAATCTTACTTAAAAA 0.20953395506

GGGTGAGAGGGTCACGTCAAAG 1.78899815921

GGGTGAGCACCTGGATGGGAAG 1.73837223737

GGGTGAGCCAGTGAAAAGTAAG 1.1986206223

GGGTGAGCGTCTGTCAGGTAAT 1.61087406107

GGGTGATACTCTCATCTATAAG 0.318178950943

GGGTGATAGACTGCCTAGTGAA 1.24493553104

GGGTGCGCCTCTGTTCCTCGAT 1.35615092744

GGGTGCTAGTGTGCGGATAAAG 1.01721718167

GGGTGGCCATGAGTTCTAGTAC 1.31122155016

GGGTGGCTCTGTGCGGATTCAA 0.862492256856

GGGTGTCCGGCTCATTTTGGAG 0.733915894031

GGGTGTGAGCATTGTTGTACAT 0.4152287074

GGGTGTGAGTCCGCATACTATA 0.665193499701

GGGTGTGCATCACGATCACAAG 1.29981927587

GGGTGTGTGACTTATGAGCTAT 0.341263377661

GGGTGTTAACGTGGTAGATATC 0.775774507142

GGGTGTTCAGCCGCTAACAGAG 0.806304991771

GGGTGTTCATAATCTCAAGAAG 0.433352146546

GGGTTAGAGAGTCCTACTTATG 0.84461911402

GGGTTAGTAAATCCTTAAGAAA 0.475895798056

GGGTTAGTCGGTTATCGAGAAT 0.994211767983

GGGTTTCCATGTCCTGATAAAA 0.840195319256

GGGTTTGAATCTGGAAGCTGAA 0.670398174123

GGGTTTGAATGGGGTTACTAAG 1.11647533251

GGGTTTGCCATTCATCGAAAAA 0.943091136072

GGGTTTGGCCCTGCGGCGCGTT 1.05394923231

GGTAAACTAAATGTTCAAGGTC 0.38745375818

GGTAAATGCTCTTTTGCTTAAA 0.641988799745

GGTAACGGTTGTGCTTTCCTAT 1.30914768153

GGTAAGCGATTTCAGGAGGATC 0.976370829869

GGTAAGTGGTCTCCTGAAAAAG 0.496641496816

GGTACACCCAATGATCACACAC 0.869215028755

GGTACACGCGGTGCCGGAAAAT 1.79975870649

GGTACACTTAGTCGAAAGTAAA 0.482552674934

GGTACAGAGGCCGATAGTACAT 0.236152070262

GGTACAGAGTCTCCTGACATTG 0.767580363254

GGTACAGCCTGCCACTGGGGAT 0.989226844988

GGTACAGCGTCTTTCTGGTAAT 0.719528149011

GGTACAGCTCAACCTCGAAAAT 0.905949234891

GGTACAGGACGTGCCACTACAG 1.40302246436

GGTACATCTTCTGAACGCTATT 0.446414261437

GGTACATGATCTGTGCGCACTT 2.10821562168

GGTACCCCATCTGATTTTACAC 0.836180383249

GGTACGCGAGGTCTTTTATAAC 1.02196718479

GGTACGGCACTTTGGGCATGAG 0.353524171976

GGTACTCAAGGTCGATGTTAAT 0.379490396662

GGTACTGACTGTCACTCCGCAT 0.249533178413

GGTACTGTCTTCCCTGACTAAA 0.685991477753

GGTACTTATGATGGTAGGTATC 1.12777876897

GGTAGAATATGTCCGGACTAAT 0.650513881162

GGTAGACACGGAGAAGGACGAA 0.199465222567

GGTAGAGAATGAGAAGCGTAAT 0.564225761413

GGTAGAGGAATTACTAATCAAT 0.594836065948

GGTAGCCTTTGTGTTCCGGTTG 0.938971513839

GGTAGCTATTTTGTTTATTAAG 0.344150015949

GGTAGCTGCTGTGCTGGACATA 0.725720350474

GGTAGTCTTCTTGGAGCATGAG 0.700322045244

GGTAGTGAAACGGATGACTCAT 1.02287371133

GGTAGTGGGTCTCGAAATGATG 0.747298231418

GGTAGTGTTTCCCTTGAGAAAT 0.780086734498

GGTAGTTAGTGTCCAAGGACAG 1.15408500662

GGTAGTTGATGTGGACGATAAG 1.3453818965

GGTATAGAATTGGGAAGAAAAC 0.512105157112

GGTATATACCCTTCATTATAAT 0.374924933443

GGTATCTTCCCTGGTCGAGTAA 0.774207113983

GGTCAACTTGTTCTGATGCCAA 1.13369223348

GGTCAAGTTCGTCCTTGGAGAG 0.7428428416

GGTCAATCACCTGCCTCAAAAT 0.747698489706

GGTCCACCATGTCCCCAAGTAG 0.825308252134

GGTCCATCACATCCTTCATTAA 0.498422553887

GGTCCATTGAATTCTCAATCAG 0.573771125706

GGTCCCTAGTCTGAACCCTGTA 0.722727379914

GGTCCTGAAAACGATTAAGAAT 0.500579042397

GGTCCTGAATCTTGTGCGTAAG 0.838761507224

GGTCCTGACTTTTGGATCAAAC 0.692595516094

GGTCCTTAAGTAGTAAAGTTAC 0.274275903696

GGTCCTTCGACTGAGCGCAAAA 1.84765791429

GGTCGACTGACTGGTGAGTAAG 1.12370645227

GGTCGATCCTCTCATATGTAAC 0.371456801653

GGTCGCCGGTGTCATAAGGAAA 1.1848226534

GGTCGCTACTCTGAGCTAATAC 0.841266294835

GGTCGCTCTAGTCAATGGTAAC 0.933017348085

GGTCGGGTCTCTCCTAGTGGAA 0.891248457083

GGTCGTCAATTTGAAAGGTTAG 0.462652662517

GGTCGTGCGGGTGGAGAGCAAA 0.612456282142

GGTCGTGTATCTGCTGTTGGAT 0.870866466485

GGTCGTTACTCTCTAACTTGAC 0.97890531717

GGTCTTGAACCTTCCCTGGCAA 0.89689154429

GGTGACGTCGAGGGTACGACAG 1.20447262721

GGTGCATCAGGCCCATCGAGAG 0.989588637727

GGTGCTTACATTCCTCGGATAT 0.338877156151

GGTGGAGACGCCCAAAACAGAC 1.19624257107

GGTGGAGGGCCTGATGGCGAAC 2.55362148742

GGTGGAGTGAGTGTTATCAAAC 1.31882144144

GGTGGATCTGATGTTACAGCAG 0.918887245193

GGTGGGAGATATTGTTTGCGAC 1.22485756698

GGTGGGGAGTATCGTAAAAAAC 1.37447755321

GGTGGTGGATGACCTCAATAAA 0.910596654894

GGTGTTCAAATTCATGAGTATC 0.236259209836

GGTGTTGTCGCTCGATAATTAT 0.224627302227

GGTTAACCCTGTGCAGGCTAAT 0.504763124491

GGTTAAGCATCTGATTTAATAG 0.547899375509

GGTTCAACCTGGCCTCTTTAAG 0.530721586279

GGTTCACCAGCTCATGGTTGTT 0.491164391824

GGTTCACGCGCTCATTGGGAAA 1.44611742664

GGTTCAGACTCCGATAGTTGAG 0.405448121985

GGTTCAGGAGAAGGTAATCAAC 1.01337107489

GGTTCATGACACGATTAAGTTG 0.809342169121

GGTTCCGAACCTCTAATGTTAA 0.384885019692

GGTTCCGATGGTGAAGCCGGAG 0.999545150973

GGTTCCTTATGTGATATATAAC 0.444074332337

GGTTCGGTTTCTGCTAAAAATG 0.772002612996

GGTTCTAACTTTGAAACACTTT 0.222062787949

GGTTCTGCTTTTGAAGGTGCAT 1.5709140864

GGTTCTGGGTGTGTCAAGGGTG 1.71840573853

GGTTCTGTCTGGGTATAACGAC 0.685581397105

GGTTGACCATGTGCTCTAGAAT 0.223934954845

GGTTGAGCCCCTGTTGAGTATG 1.6936048534

GGTTGAGCTCCTCGATACTTAA 1.18500011036

GGTTGAGGGTGTCCAACGTCAT 0.498173670525

GGTTGAGTATAAGCTACTGCAA 0.437318173777

GGTTGATACTGAGGCCAGTAAC 0.431830219892

GGTTGATTCTTTGCATCTCTAT 0.31322881173

GGTTGCCCGAGTTATAGTTAAG 0.847617520402

GGTTGCCTTCAGCAAAATTAAG 0.575027290203

GGTTGTCAAGTTGCAATAGAAA 0.435454283339

GGTTGTTACGCTCGTTATATAC 1.53011336584

GGTTGTTCGGGGGATGAGATTA 0.926443404361

GGTTTACACCCTGCAAGACGAA 1.14497901657

GGTTTAGACTTTGTAGACCTAA 0.213100044206

GGTTTAGAGTGTGCTGAGGAAC 1.2484811858

GGTTTAGGGTCCGATTGGAAAG 1.17968647834

GGTTTATAAGGTCATCCGTAAC 0.579296187945

GGTTTATTGTTTGATAAGTCAG 0.441317013381

GGTTTCCAGGTTGATCACAAAA 0.615151221026

GGTTTGTAGTCGGTTGCCTCAC 2.84457721569

GGTTTTTCATCCGCAACCCAAA 0.199152752769

GGTTTTTTGTGTGCTTCTGCAG 0.719526126978

GTAACAGTGGCTGAAATCGTAC 0.653918557482

GTAACCGCGACTGAAACTTGAT 0.498085064565

GTAACTTCGTTTCCTAATTAAA 0.406253561659

GTAAGTGAAAGTGAAAAGACAG 0.367036960215

GTAATAGTGACTACTCAGATTC 0.603874338983

GTAATATATTCCGAGTAGGTAT 0.478737195257

GTACGACGCTCTGAAGTCCGAC 1.23622970502

GTACGAGTATTGGCGCAGCAAG 0.311005366336

GTACGATAAACGGCAAAGGAAG 0.574024046295

GTAGAATGATGTCCTGCCTGAG 0.989876203801

GTAGGAGGTTCGCCTCATAATC 0.339498495877

GTATCCGGCGTTCCTAGGAGAC 1.04264087811

GTATTTTTACGTGGAAAAAAAG 0.254181711473

GTCAAACGATCTTTATATAAAA 0.905619631538

GTCAAACTCGGTGGGTTAATCG 0.843696801973

GTCAAATAGTGTTCGCCTGATG 0.406075596816

GTCAAATGCCCTGCAAATGGAG 0.908019921007

GTCAACGTGGATTTTGCTAAAG 0.623304301803

GTCAACTTTTGTCATAGGGAAG 0.443653934284

GTCAAGGGGTGTCCTGCCTTAA 0.729649778193

GTCAATCAATCTCTATGGTTAT 0.141211905917

GTCAATCAATTACATGAGAAAG 0.202552014233

GTCAATCGGAGTCATCGGTCAC 0.472459612973

GTCAATGAAAGTGTTACTCCAG 0.566379287783

GTCAATGAGTTTGATGGGGATG 0.540787682512

GTCAATGCGCGCGATTGGAGAT 0.777075222398

GTCAATGGGAAGCTTAGAAAAA 0.531973321624

GTCAATGTAGGTGAATAGTTAG 0.248601450812

GTCAATTCCAGTGCATGGTGAT 0.327317271363

GTCACAATATGTTCTAAATAAT 0.434474287938

GTCACACAAACTAACGGGAGAG 0.539112450398

GTCACACAATCGGAATCTAAAA 0.34417557605

GTCACACAATCTCTCTGACAAT 0.936293465726

GTCACACAATCTGAAAACGAAC 0.145174973373

GTCACACCAGCTGAAGTCGAAC 1.66037148021

GTCACACTCGTTGCAGTCATTG 0.779256926861

GTCACAGAATCTCATGTCTAAT 0.54317080535

GTCACAGAATCTTATGGATTAT 0.335105448212

GTCACAGAATGTGCAAAGAAAC 0.401352891473

GTCACAGATTATGTTACGTTTA 0.573012398285

GTCACAGCAAGTGATGTGTCAC 0.556909315276

GTCACAGCATATCTTTCGGTAC 0.380519874611

GTCACAGGATCTCCTATCATAA 0.396208096118

GTCACAGTATTTGATCATGAAG 0.485702333137

GTCACATGAATTCAATAGGTAT 0.598471111678

GTCACATGGAGTCATGATACAC 0.598637091264

GTCACATGGTCGCCTTAGATAG 0.540602129681

GTCACATTATATGGGAGTGGTC 0.670198606736

GTCACCCACTGTGGAAACGTAG 0.883723366644

GTCACCGAGTTTCCTTAAGAAA 0.403175110657

GTCACCGATTCTCTCATCCGAT 0.557770640731

GTCACCTACTATCGTACAACAA 0.973273344742

GTCACCTATCGTTATTCCGCAT 0.559711948324

GTCACCTCAACTGGTGGAGCAT 0.522750228759

GTCACGCAATCTGTTAATCCAG 0.501009968366

GTCACGCAATGTCCGTGTATAG 0.877521542781

GTCACGGACGCTCAAGACCTAA 1.01118866002

GTCACGTTGGGTCGGTAACGAA 0.868027207015

GTCACTCCCGGGGACCTGAAAC 1.27718390895

GTCACTCGCTCTCCTAAGCCAT 0.462763369149

GTCACTCTTTATGTGTAGATAA 0.552194865322

GTCACTGAATGGCTAGCCAAAT 0.670761068876

GTCACTGCATGTGTAGGTTAAG 0.913221685677

GTCACTGGGAATGCTGTGTGAG 1.0441471896

GTCACTTCAACTCATGATAAAC 0.456452500698

GTCACTTGAACGCCTTTTAAAA 0.49101487824

GTCAGAAATCGTGAGTGTATAG 0.393221501274

GTCAGAACCCCTCAACGTTAAA 0.361509403181

GTCAGACAATTTGTATGATGAT 0.700723403656

GTCAGACAGTGCCCATATGATC 0.659207366283

GTCAGACGGTGTGGATTTACTT 0.272956923989

GTCAGACTATTTCAGTAGTATT 0.369874697252

GTCAGACTGTCTCATGCCTAAG 0.247451225491

GTCAGACTGTGTCCAGCCCAAT 0.407281997788

GTCAGAGAAGCTGAGAAGTAAC 0.273626937218

GTCAGAGAATTCGCGCGATTAT 0.856634512513

GTCAGAGAGTCTCTAGACGAAT 0.587497357863

GTCAGAGTAGCTGCAGCTTTAA 0.68466758294

GTCAGAGTAGGTCAGAAATAAA 1.14435052932

GTCAGAGTCCGTCCTGCGGTAT 0.870790727902

GTCAGAGTTTCTCCTTTTAAAC 0.703166535103

GTCAGAGTTTTTGATACCCAAT 1.1051888236

GTCAGATACAGACAAACGTCAC 0.950580332105

GTCAGATCTAGTGATTCGAATA 0.279980810368

GTCAGATTAACTGCTATTTTTT 0.489588598351

GTCAGATTATATGCTAACGAAA 0.236771000768

GTCAGATTATCTGTTAAAACAG 0.15034292561

GTCAGATTGTTTGAGACCCAAT 0.643306233907

GTCAGATTTTCTCTTGCCTGAA 0.977881142045

GTCAGCAAGGCTTCTGCCGCAG 1.35526407914

GTCAGCGCAGCCGATGTCTGAA 0.164657287962

GTCAGCTACACTCCAGAGAAAG 0.632092948568

GTCAGCTCAGGGGCTTACGTAC 0.480964446145

GTCAGCTTATCTGGTGGCCTAT 0.697045829805

GTCAGGGCTCCTGCTGGTATAC 1.25182330912

GTCAGGGTACCTCTTGAGGCAC 0.895679031727

GTCAGGTGATGTGATACGAGAC 0.546387979111

GTCAGTCAATATCCTCGTCGAC 0.2862799349

GTCAGTCAATCACCTAGCCATT 0.761494030727

GTCAGTCACGGTGATCGGTTTT 0.490445645869

GTCAGTCGATGTCATCAAAGAC 0.214090157253

GTCAGTGAATGTGCTTAAGAAA 0.511253306378

GTCAGTGTCACTGAAAAATTAG 0.523951551432

GTCAGTGTGTGTACTTTAACAG 0.207945687219

GTCAGTTACTCTGCTAGCTCTT 0.554303456031

GTCAGTTCCTTCGTGCGTCGAA 1.37275064276

GTCAGTTTAACTTGTCATTTAA 0.386373488228

GTCAGTTTAATTTTTGCGAAAT 0.444740794576

GTCAGTTTCGGTGGATACCGAA 0.583513980162

GTCAGTTTGTCTGGTGCTACAA 0.467904024916

GTCATATAATCGTTCAGATCAG 0.77327232969

GTCATCGTCCCTCATATGTTTA 0.361160267229

GTCATGCCCTGTCAGGAGTTTG 1.37206327158

GTCATTCTAACAGCGCAAAAAT 0.566751526961

GTCATTCTTAATCAAAGGACAG 0.540109296578

GTCATTCTTTCTCCTAACGGAC 0.647502819634

GTCATTGACCATCGTTTCTTAT 0.669045648622

GTCATTGCGGGTCGTGACAGAC 0.239524011477

GTCATTGGACGTGCAACGAAAG 0.19221931443

GTCATTTTAAGTGAAGGGAGAC 0.501632904163

GTCATTTTGCGACCTCAACGAG 0.831025519711

GTCCAAATCTCTGGTAGGTATG 0.467271982267

GTCCAAGAATGTCAGATGTAAG 0.711159845126

GTCCAAGTCGTGCCTACGACAG 0.59835506685

GTCCAATAAACTGGAAGAACTG 0.33869389938

GTCCACCAGTTTGCAAATTTAC 0.282831881782

GTCCACTTCAGTCATGGTTGAC 1.11836584952

GTCCATACAACTCATCAGCATA 0.219222274318

GTCCATCACTGCCCTGTTGGAA 0.884344166661

GTCCATGATGATGAGGGTATAC 0.530559042276

GTCCATGTCCGTGCTATCGGTC 0.652136592322

GTCCATTATTGTCAAGGCATAC 0.417288697694

GTCCATTGAGGTGCTAAAGATA 0.385285752316

GTCCCAATTGCTCCCACACTAT 0.460483466684

GTCCCACAATTGGCTAAAAGAT 0.82352414922

GTCCCACAGGCTGAAACGGGTT 0.406217204103

GTCCCACATTCTCAAGCATAAC 0.125844181459

GTCCCACCTTCTTTAGGGCAAG 0.635276470655

GTCCCACGATCTGAACGAGTAG 0.694890246739

GTCCCACGTGCCCGTACTTTAA 0.774058225452

GTCCCACTTGCTTACTCGAATC 0.474070539245

GTCCCAGAATTTCATCCCGAAG 0.929397576997

GTCCCAGCTCGTCATCGCAAAA 0.857130543476

GTCCCAGTTTCTTCGTAGGAAT 0.609766816993

GTCCCATAAGCTCCAGTCTAAC 0.502636535498

GTCCCATGATCTGGTAATTGAT 0.481888073695

GTCCCATGCTCTCAAACGTAAT 0.385103039242

GTCCCATTGTATGCAGGATTAG 0.278050867226

GTCCCCCACTTTCCTTTGGGAC 0.932925238086

GTCCCGCACTTTCATATATTAA 0.337086671654

GTCCCGGAGTGTCATGCCGATG 1.37834349116

GTCCCGGATAAGCAGTTGTAAA 0.370235194706

GTCCCGTCAGATCAGCAAGTAC 0.542911497201

GTCCCGTTGTTTCATTATCAAC 0.336555265533

GTCCCTACGTGGCAAACACCAT 0.384745080657

GTCCCTATATCTGCAAGGATAA 0.536166535438

GTCCCTTAAGCTGGTCATCAAA 0.635069922133

GTCCCTTTACGGCCAATCGGAC 0.73194782217

GTCCCTTTGAGTGCTGGATTAG 1.67800837795

GTCCCTTTGATTTATCTAGTTA 0.551699147115

GTCCGAAACTGTGAATGAACAA 0.26932237239

GTCCGAGGAAGTCCTAATATAG 0.817737846204

GTCCGAGTACGTCCTAAGGCAA 0.584861269

GTCCGAGTCTAGCATCGATAAC 0.704557869474

GTCCGATGATTACAACCCAGAG 0.656643221693

GTCCGATGCTCTCATTATTAAG 0.703694315448

GTCCGATGGCATTCATAAACAG 0.594483165714

GTCCGATTCGGTTATGTCAAAT 0.902956412029

GTCCGATTGTTTGCTAATAAAA 0.270704581877

GTCCGCGTAAGTTATGCCTATC 0.558144788815

GTCCGCTAGCGTCATTCTGGAA 0.529903038139

GTCCGGGTAGCTGATCACATAA 0.313313476851

GTCCGTCCCAGGTTGGAATAAC 0.621948349045

GTCCGTGCACGCGATTGGATAG 0.701959494715

GTCCGTGTGGCTCATTGTGGAA 0.888698594964

GTCCGTTACAGTGATAGGCCAG 0.62335536273

GTCCGTTGGTGTGTTGAAAATT 0.59771288271

GTCCTAAAAGCTGATTCAGGAG 0.571832960378

GTCCTAGACACTCAAATGTTAC 0.465125722899

GTCCTATAGTCACCATACCAAG 0.334520318949

GTCCTATATCCTGCTAAAAGTG 0.523251972154

GTCCTATGCAGTGTTCAAAGAC 0.649997604373

GTCCTCCTGTTTTATTGATTAC 1.0194210344

GTCCTCTGGTTCGGTCGACAAC 0.524324321246

GTCCTGCTACTTGGAACCTTAG 1.89606375635

GTCCTTCCTCCTTCTGGTAAAA 0.394221025073

GTCGAAGCGAATTATTGTGGAA 0.411941955949

GTCGAATATTCTGATTACAAAT 0.630695647023

GTCGATCAATATGTTTCGTGTT 0.239977561768

GTCGATGTACAGCCAACACCAT 0.592153486052

GTCGATGTCTGTGCGCCTTAAT 0.912717005692

GTCGATTGCTCACATCTCTATC 0.374701936086

GTCGCACAGGGTGCACATTAAC 0.213153667363

GTCGCACCGATTCCAGCGAAAT 0.219818020815

GTCGCACGCTCACAAGGGCAAG 0.743996182711

GTCGCACGGTGCCGGGCGTTAG 0.881965639471

GTCGCAGTCACTGGAAACGTAT 0.85411151517

GTCGCATAGTCTGTTTACTAAT 0.490734637493

GTCGCATGCGGTCAATCTCAAT 0.555488106434

GTCGCCCAAACTGAGGATGATA 0.558167329547

GTCGCCCCGGTTGCTCTCGAAC 1.39145684546

GTCGCCTCGTCTCATACGGAAA 0.651196810143

GTCGCGCATGATTATAGATATG 1.07696091715

GTCGCGGTAGCGCCCATTCAAT 0.710738006738

GTCGCTGGAAGTCCAGCCGCAC 0.980648660098

GTCGCTGTCGATGTGCACGGAG 0.96527490314

GTCGGAAGTGCTGATAAGTTAG 0.470603434397

GTCGGACGAACTCTTATTATAG 0.302831064065

GTCGGACGACATCGTGCTCAAG 0.484084063164

GTCGGACGATGTGCTTTTGAAA 0.293708204782

GTCGGAGAACTAGTTTTCTAAC 0.381272861705

GTCGGAGAGGATCTTAAGTATC 0.494203727349

GTCGGAGAGTGTGGTTACTAAC 0.613323699276

GTCGGATCATCCTGTGGCACAC 1.69664595479

GTCGGCCTCTCGGCACGCTTAC 0.389302681297

GTCGGCGAAAGTCTTGCCAGAA 1.05961069798

GTCGGCGAGCCTGGCAGCACAC 1.78776043107

GTCGGCGATGCAGCGGTGCATC 1.38696135046

GTCGGGCTATGGCCTGTCTTAC 1.35909311711

GTCGGTATGCGTCCATGATAAG 0.864069096472

GTCGGTCCCCCTCGTTAATTAG 1.86940293042

GTCGGTCTTTCGCAAAAAAGAG 0.297028006387

GTCGGTGCTCGTGCTACTGGTG 0.523649417579

GTCGTAATAGGTTATAACAAAA 0.493741479855

GTCGTAGATATTGCTGACTATG 0.894599193908

GTCGTAGTCTACGACGGCATAG 1.34379279355

GTCGTAGTGACGCATAGCCTAA 0.678306956326

GTCGTATACAGTGGCGCAATAT 0.740727154097

GTCGTATTACGTGGTGCTGAAT 0.715279191593

GTCTAACCGGCTGAATTTGAAG 0.498687047274

GTCTAAGAAATACCATCCTAAT 0.274919786018

GTCTAAGAATCTCAAAACGTAC 0.166928664135

GTCTAAGAGTCTCATAGCGGAA 0.381575286833

GTCTAATCAGCAGAAGATATAG 0.400934880674

GTCTAATTGGCTCACAATGCAC 0.903794080529

GTCTAATTTTGTGCATAATAAA 0.473636245265

GTCTAATTTTTTCATTAGAGAA 0.593467101066

GTCTAGGAATGTCTTTGAAGTT 0.632301409308

GTCTATCCATGTGTTGCACTTT 0.880360970728

GTCTATCTGAATGCTCTACGAC 0.565485861846

GTCTATGCATCTCAGTCGTAAA 0.584349674148

GTCTATGCTTATGTTAAGAAAA 0.335086862369

GTCTATTAAGGTGCATTGTAAC 0.432459858742

GTCTATTACTCTCAAGCGGGTC 0.152793715161

GTCTATTCTGGTTTTTGATCTA 0.333972576368

GTCTATTGAGGTCGCTACATAG 0.267239160797

GTCTATTTGTCTGCTGCCGCAA 0.779179020747

GTCTCAAAATTTGGGATACGAC 0.591235497261

GTCTCAACATCTCCAGGGTAAA 0.447509607737

GTCTCACAAAGTCTTTTATGAT 0.262741855801

GTCTCACAAGCTGATGAAATAT 0.310465958411

GTCTCACAGTCTCCATTCAAAG 0.42719472987

GTCTCACAGTTTGAAACAGCTC 0.465554554298

GTCTCACAGTTTTGTAGAGAAG 0.287509353409

GTCTCACCATATGGACTTGAAT 0.703906402695

GTCTCACCGTATGAATGCCAAG 1.60718606005

GTCTCACGATCGCCTCATAAAA 0.287426203743

GTCTCACGATCTGCTTGTGAAC 0.618666696204

GTCTCACGGGGTCTATAGCTAG 0.423478438086

GTCTCACTAAGTGCTCAGGGAC 1.30480974904

GTCTCACTCATTAATATAGTAC 0.443525824247

GTCTCAGAAACTCTAAAAGGTG 0.725167444597

GTCTCAGAAAGTGGTCATGGAG 0.449049056278

GTCTCAGATCGTGTACCCATTA 0.401637609367

GTCTCAGATTCTGCTAGTAAAA 0.260301758822

GTCTCAGCATTTCATCTTTTAC 0.423271557831

GTCTCAGGATATGTTGAAAAAC 0.624091851607

GTCTCAGTATCTGCGTTTGAAT 0.44776683828

GTCTCATAAACTGAACTAAGAT 0.455365115782

GTCTCATACACTAATTCGCAAG 0.647497537056

GTCTCATACTTTGGATATCCAA 0.307074566264

GTCTCATAGGGTCTCCTTATAG 0.337880752587

GTCTCATAGGGTGTAGCTCAAG 1.07655074169

GTCTCATATTCTCTTGTAATAA 0.593527803178

GTCTCATCAAGGGATGAGAGTA 0.335767053518

GTCTCATTACCTGTTAGCAAAG 0.240423319154

GTCTCATTTCATGGTTGGAGTA 0.327230162919

GTCTCCCACTCTGGTCTCCAAG 0.667345420805

GTCTCCCGCGCTGGTGGAAAAT 0.815064294459

GTCTCCGCGGGTACCCCGTCAG 1.14285098006

GTCTCCGGACCTGTACCCTGAT 1.48373447638

GTCTCCTTCCTTTCTATTGATC 0.624693847484

GTCTCGCCCGTTCCTTGTACAG 1.17212767903

GTCTCGGTGAGTGGTTTGATAG 0.438783518933

GTCTCTAGAAGTCGTGAAGGAG 0.722782039546

GTCTCTCAGTGTCAACTTCGAT 0.638839484035

GTCTCTCCACCAGTAGGCTGAA 1.61984848467

GTCTCTCTCCGTGTAGCTATAC 0.697354851405

GTCTCTGACTGTGTTGTCTCAG 0.732237305195

GTCTCTGAGTTTGCTTAGCGTT 1.34144499638

GTCTCTGCCCGTCAGACGGTAT 0.479993221063

GTCTCTGTTTCGGCTGGCATTA 0.880373812232

GTCTCTTAAGCGGCTATATAAG 0.260521844344

GTCTCTTGATGTCATCCATATT 0.681770179031

GTCTCTTTCTCTGAATGAGTTC 0.287226138996

GTCTGACACCCGCATGAGTATG 0.972409137507

GTCTGACATATGCGTACCCAAG 1.0758121858

GTCTGACTAGTTTCTATACGAA 0.41465899036

GTCTGACTATCTGTAGTTTTAG 0.659880776465

GTCTGACTCAAGGAATCGTAAA 0.324178070703

GTCTGAGAAAATGAAGAGGTTC 0.83158572824

GTCTGAGATACGTCTCTTAGAG 0.324030913795

GTCTGAGGCGTTGTTGGCAATC 1.42273122626

GTCTGAGTAAGTGCATTGTGAT 0.787217596201

GTCTGAGTATCTGCGTCAAGAC 0.605518953317

GTCTGAGTGGATTGAGTCCCAG 1.80305704003

GTCTGATCTAATGCTTAATAAA 0.173387463952

GTCTGATTATCTCCTTTAACAG 0.441875647028

GTCTGCACATATCTCAAATGAT 0.441331414785

GTCTGCCAATCTGGTGGCCGAA 1.41315178175

GTCTGCCACAATCAACACGGAA 0.400928445903

GTCTGCCACTTTGTAACTTTAA 0.454514370857

GTCTGCCAGCTCCCTTTAGCAG 0.621915080721

GTCTGCGCATCTGCTTTACAAT 0.552070415775

GTCTGCTCATGTCAGTCCTAAG 0.968835338825

GTCTGGCGATTACCTTCCCGAG 0.957390446337

GTCTGGCTACGTGCCCTGGAAC 0.593504473528

GTCTGGTACACTGTTCGTAAAT 0.846618514889

GTCTGGTTCCTGGCATAACCAA 1.07920834977

GTCTGTCCGTTTCGTGCCTCAC 2.56403342629

GTCTGTCGATCTCCAGAGGAAT 0.642252298282

GTCTGTCTACCTGATTACCATT 0.322765232791

GTCTGTGAACCTCCAGCTTAAG 0.999109964348

GTCTGTGAATGTGCTGCTGAAA 0.993225678445

GTCTGTGGCCTTCGACGTAAAA 0.823664669054

GTCTGTGTTGGACCGAGACAAT 1.02826283373

GTCTGTTAGTATCCTGCTGAAC 0.933619820373

GTCTGTTTATGTGATTCCTAAC 0.659022156305

GTCTTACAGTCTGCTCCACGTT 0.181407249551

GTCTTACGACGTGTTCGGCATT 0.257953145774

GTCTTCCCACCGTAGCCTTATC 0.964096474964

GTCTTCGCACCTCCATGATCAG 0.347887828471

GTCTTCGGGACTGGTAGACCAC 1.72803392919

GTCTTTATAGCTCTTACATTAC 0.482441781181

GTCTTTCCTGCTGGACGGAAAC 0.869721151394

GTCTTTCGAGCAGCTTAGGCAC 1.09984890422

GTGAAACCGTAAGCTCAAAAAT 0.207212367215

GTGAAAGCTGCTGCGTTAATAA 0.641762418026

GTGAAAGTTTCCCTTACGGATG 0.764726904824

GTGAAATACGATCTGCAGAAAA 0.0693666056406

GTGAACAAATTTGAGGCCCAAC 0.793912812921

GTGAACCGTACGCCTCCTTAAA 0.567211616752

GTGAACGAAGGGCCTTCATTAG 0.318510522872

GTGAATGAGAGTTACTGGTAAG 0.65597977202

GTGAATTACGCTGGTTAAGAAA 0.567064838667

GTGAATTCCTGACTTGCAACAC 0.75256224173

GTGAATTGCTCTCCTTCGGCAT 0.790334707249

GTGACAACAACTGCAGGGGAAG 0.966610081461

GTGACACAATGCCTAAAGAAAC 0.443179143564

GTGACACCAACTTGAGTAACAA 0.500905197524

GTGACACTATGTCCTAGGGGAC 0.982188236066

GTGACACTCGGTGATGTGGAAG 0.445474374797

GTGACACTCTCTCGTGCCGCAC 0.675681457105

GTGACAGAAACCGGTTCGTGAT 0.261292015268

GTGACAGAATTTTCATACTAAT 0.375023413476

GTGACAGAGAGTCCCAACTAAC 1.14612365985

GTGACAGATAATGAAGCCCTAT 0.29192674002

GTGACAGTCACTCCACATAAAC 0.876992018572

GTGACATACTGTGCATCTTGAG 0.995208527996

GTGACATAGTATCCTCGTGCAA 0.343688793517

GTGACATCAAATGCTCCAGTAC 0.590886518845

GTGACATTGGGTGCAGATAAAA 0.244851403464

GTGACATTTATTGGTGTTGATA 0.544883423497

GTGACATTTTGTGCTCGATTAG 0.384372335739

GTGACCCGACTTTCGGACGTAA 0.852407996752

GTGACCGACGCACATAGCAGAG 0.506113250552

GTGACCGACTCTGTTTTCAAAG 0.438727238898

GTGACCGAGTCGGCAAGAAGAG 0.469888118072

GTGACCGTAGGTCCTCCGCTAT 0.539647929238

GTGACCTTATGGGGAAGCTGAG 0.448207378195

GTGACGGTGTTTGCTATATTAA 0.263400941593

GTGACTCACTGTTGTGGTTAAC 1.39358823063

GTGACTCCTTTGGGTATGCGAA 0.205091706104

GTGACTCGCTATGGTTGAACAA 0.800379968713

GTGACTCTTTGTTCTGCGCAAT 1.08632773143

GTGACTGAGGCTGATCAGTCAG 1.15524166477

GTGACTGTTGGTCCACCACTTA 0.657189578269

GTGACTTGTGGTGTACACGGTC 0.804644805496

GTGAGACTAACGCGAGGCCTAA 1.63559990241

GTGAGACTCCGTGAAACTTGTT 0.449791979201

GTGAGACTTTCTGAAGCAGTAC 0.892830833454

GTGAGAGAATCTGTGACATGAG 1.3175939821

GTGAGAGAATGTCCACAGATAA 0.444833516959

GTGAGAGCATCTCCTGTGAGAC 0.139963207824

GTGAGAGCATCTGATGGAAAAA 0.927572880907

GTGAGAGTCTGTGAAAATCGAC 0.650355337889

GTGAGAGTGTCAGAATGACAAT 0.600336044597

GTGAGATACTCTGAGCGCCGTA 0.271161143718

GTGAGATAGTATGGAGGGATAA 0.295114479412

GTGAGATCAACTGTAGGCAAAA 0.415723354001

GTGAGATCCCATACTCAGCAAT 0.326566899047

GTGAGATCTATTTCAGACCGAC 0.436191603218

GTGAGATGAACTGCTCGGCTAT 0.515960782809

GTGAGATGCCATGATACTGTTC 0.750185751686

GTGAGCCAGGTTCATGCCTTAG 1.0581842197

GTGAGCCTGGATCTATCTAATG 0.393698645255

GTGAGCGACTCTGGTTCAAAAG 0.490836047518

GTGAGGTAAAGTGGTGCCGAAT 0.603722751899

GTGAGTACACCTGAACAAAGAA 0.521221242511

GTGAGTAGGAGGCAATCTGGAT 0.631565309419

GTGAGTATAAGAACTTTTCTAG 0.355994868986

GTGAGTCAGCCTCATCAACTAT 0.637091974223

GTGAGTGACACTGATGCCGGCA 0.830349677664

GTGAGTGACCGTGAATCAATAG 0.585118739504

GTGAGTGATGGTCCCCCTCAAG 1.68719284186

GTGAGTGGGGGTATTCTAGCAT 0.864333158406

GTGAGTGTGGCTCCTAAGCAAC 0.898380854923

GTGAGTTACACCGCACTTACAA 0.684135904856

GTGAGTTATTATGCTGTATAAC 0.524373109926

GTGAGTTCCTCTGGTTGACATA 0.795996331483

GTGATACAATATGATGTGTAAT 0.568930018005

GTGATACCATGTCAAGGCGAAT 0.907539098989

GTGATAGAATCTCCTGATAAAC 0.644521572918

GTGATAGACACTGATCCAGGTG 1.34795825213

GTGATAGTGTGTGCAACTTTAT 0.816089327584

GTGATATCATATGATCGGAAAT 0.327758830357

GTGATGCCGTCTTAGTAAAAAT 0.848406994864

GTGATTTCCACTGATATCGAAG 0.232056434876

GTGATTTTAGGCGATCACCTAG 2.82781049878

GTGATTTTATCTCCTACTTTAG 0.29089404454

GTGCAAGTAATTGATGGATATG 0.326689374267

GTGCAATAGCGGCATAAGGTAC 1.16486378442

GTGCAATCATCCGTAAAAGAAC 0.764777596384

GTGCAATGACGTGCAGAATAAC 0.425853488528

GTGCACTTCTGTCCTGCCGAAC 0.619707122261

GTGCATGAAACTCGTTACTCAC 0.46851885256

GTGCATGAATGTCAAGCGATAT 0.720671758798

GTGCCAAAGCCTGCTAAGGTTT 2.60501138188

GTGCCAAGATCACCATCTGCAA 0.515827779083

GTGCCACAAAGTCGTTAGGGAA 0.478946696777

GTGCCACAGTCTGCGGGTGAAA 0.915561085862

GTGCCACAGTTTGTATAAGCAG 0.931363859066

GTGCCAGAATGTCAGTGATTAA 0.436726166249

GTGCCAGATCCTCTAAATACAC 0.393994944956

GTGCCAGCATCTGAAATCAAAC 0.26393030697

GTGCCAGTCATTGCACGTTTAC 0.3813131443

GTGCCATAGTATGCCAAGTAAA 0.389671145617

GTGCCATCCACTGAAGATTCAG 0.42057552996

GTGCCATTCTCAGGTGATGTTG 0.747209135729

GTGCCATTTTTTCCAGAACGAA 0.240382529533

GTGCCCGTAGGTGCTTAATAAC 0.712167696223

GTGCCCTGGGGTGAAGAGCCAC 1.62749826976

GTGCCCTGTCGTGCTTATGTAT 0.720601622074

GTGCCGGACACTCAATCCCATA 0.550481774416

GTGCCGGCGTATGATTATGAAT 1.21221870515

GTGCCTCAGTGTGCTAGCATAC 0.869957322666

GTGCCTGAATCTACTAGGTAAC 1.2490494107

GTGCCTGGACTTCAGTATTTAC 0.166052294952

GTGCCTGTTTGTGATCAGATAA 0.628610464664

GTGCCTTAATATGCTGAGCAAA 0.652392934214

GTGCCTTAATCCCATGGAAAAA 1.33671440577

GTGCCTTGATGGCCACGCATAC 1.12551714457

GTGCGACAATTTGGTGGATAAA 0.539975054711

GTGCGACTGCGTGAAACTCGAG 0.882783098522

GTGCGAGAATCTTCTATTGAAG 0.457007801721

GTGCGAGCAGGTCCAAAGGTAG 1.45608135967

GTGCGAGGATATGCTAATGAAA 1.04382109476

GTGCGAGTCTTTCAGTGACCAT 0.559907522081

GTGCGATAGTATCATTCTGAAG 1.10973378297

GTGCGATCATCTGATATGTCAG 0.587286444637

GTGCGATCATCTGCAAACTAAA 0.715960383525

GTGCGATGTCCTCCATACAAAG 0.577977534187

GTGCGCCCAGGTTATCATGTAG 0.904212359207

GTGCGCTATTCTGTTGCATAAG 1.16157237608

GTGCGCTCAACGCATTGGTAAG 0.87070288405

GTGCGCTCCAGTCGTGACTATC 1.1172315144

GTGCGCTGAACTCTTGGGTCAT 0.90644385316

GTGCGGCACATCCAATATGAAA 0.48768104484

GTGCGGGCCCCTTTTTGTTGTG 0.78763618837

GTGCGTACAATTTAAAAGACTG 0.419411525078

GTGCGTCACTATCCTAGCCTAG 0.574201787681

GTGCGTCATCCTGATAACAGTG 1.90432589473

GTGCGTGAACACCGTGTGTCAG 0.579808155639

GTGCGTGTACGTGGCCAATCAT 0.226312136341

GTGCGTTAGTATCGTAGAGATT 0.828912996095

GTGCGTTTGTCTCTTAAGAAAG 0.192208202254

GTGCTACTATTTGCCTTATGAG 0.80732969326

GTGCTACTGCGTGCTAAGTGAG 0.689347469982

GTGCTAGACTAAGTGGGTATAC 2.94627750939

GTGCTATCGAGTCACGATAAAT 0.496099857187

GTGCTGAACTCTTTAGACAAAC 0.464847560705

GTGCTGTAGTCTGCTGAGTTAT 0.212114774765

GTGCTTCGCTCGTAATCGGGAT 0.376578850441

GTGCTTGACTGGGCTCACACAC 0.581273773146

GTGCTTTAATGTGACGAGCTAG 0.539475343211

GTGCTTTGCTCTCATCAGAGAA 0.383347884908

GTGGACTGGGCTGTATATTTTT 0.515755826779

GTGGACTTCTACCAAAGCGTTG 0.451196018465

GTGGATGTTTGTCATCTGCGTC 1.38507653971

GTGGATTCCTCTGATCTCCCTC 1.21988937251

GTGGATTGATCTCAGAACACTG 1.03210137988

GTGGATTTGTATCATCACAGTC 1.44930187652

GTGGCACTAGCTTAACTTAAAA 0.401400897669

GTGGCATTAACGCCTGTCTCAT 0.605761531501

GTGGCATTATTTCTTAGGAATA 0.567941106405

GTGGCATTCAGGGATGGATCAC 0.912777848886

GTGGCCGTTTCTGATACATAAG 0.903386930279

GTGGCCTTATCTGATAGATGAC 0.623533584443

GTGGCGTATCGGCGTCTATAAG 0.857103479544

GTGGCTCGCTCACATCACTAAA 1.08149608022

GTGGCTGAATATGGGGTCAAAG 1.1448960186

GTGGCTGAATGTCCTTAAACAT 0.404751972203

GTGGGACAGGGACCAGACATAC 1.3924524301

GTGGGACTATGTGCTGCGCGAG 1.80928802294

GTGGGATAATGTCTACGGAGAG 0.430860717764

GTGGGATTCTGGGTACACTAAG 0.590117696085

GTGGGCCAGAGTCCAAGTAGAC 1.2696833639

GTGGGTAAATCTGATGAACTAG 0.470201547864

GTGGGTGCGGGTGATAGAATAT 1.17662595715

GTGGGTGTGTCCGCTTCCATTA 1.57225111701

GTGGGTGTGTCTCACTGTGTTA 0.335324036269

GTGGGTTCCTCTCAAAAGGGAC 0.85006446412

GTGGGTTGCTCACTAGACCGTG 1.05836670775

GTGGGTTTAGCGCGTGTGATAC 0.236869307292

GTGGTCGGATCTGCAGCGAGAC 0.593790989627

GTGTAATAATCTGCATACGGTG 0.911948849194

GTGTAATTGTGTCCTTAATGAA 0.249500408107

GTGTACCTACCTGGTAGGAGAA 0.792038114676

GTGTATCTGTATGATGACTCAC 0.640719618989

GTGTCAAACACTGAATACAGAG 0.596966272607

GTGTCAATCTGTTGTGATAAAG 0.397829525547

GTGTCACACTCTCCTGTAAAAG 0.605747362385

GTGTCAGTGGTTGCCAGGTTAG 0.60587376963

GTGTCATCAACTGTATTGAGAT 0.776784655456

GTGTCATCGTTTCATACCTGAT 0.900847096947

GTGTCATGTTGTCTTCGAGGTA 0.756682123423

GTGTCCGCTACTTCGTTACAAT 0.596328618496

GTGTCGGTCTCTCATACGGAAA 0.725783934233

GTGTCGTTATAAGATGATGTAA 0.475080096254

GTGTCTTCCAGTTGTAAACAAG 0.553239208641

GTGTGACCTTGTCTAACCAAAA 0.796736955039

GTGTGAGTATCTTCAAATTGAA 0.311638245949

GTGTGCCGGCTTGAATGGACAC 2.46628763293

GTGTGCCGTAGAGCTGACACAC 0.304304963346

GTGTGCGAAAATGATATGAGAC 0.316890292827

GTGTGCGCATGAGTTTGAGGAC 0.722975077874

GTGTGCTTGCGTGACAGAATTA 1.13443939697

GTGTGCTTTAGTTGAAGCGGAA 0.86661422964

GTGTGGCTGTATGTTACGATAG 0.839397754673

GTGTGGGTATGGGCAATCTATT 0.811009835015

GTGTGTACATGTCCTACCGTAG 0.446128953352

GTGTGTCTCTGGTCCAAGATAC 0.378294811115

GTGTGTCTGAGTGCTGTTCTAT 0.275076963829

GTGTGTGCAAGTGATGGAGCAA 0.688601343168

GTGTGTTACAATGCGCCTTGAT 1.14392317362

GTGTGTTTATTTCGAGATGTAG 0.475384975398

GTGTGTTTGTATGATTTGGATT 0.329648978867

GTGTTAGAGTTTTCTCAATGTT 0.231580756717

GTGTTAGTACTTGTAAGCATAG 0.510106438527

GTGTTAGTAGATCGGTTTGAAG 0.649113742933

GTGTTATTTGCGCTACGAATAA 0.560156563447

GTGTTTGTACCTTAACAGTTAG 0.569175719602

GTTAAACGTGGTGCTAGAGCAG 0.623254918794

GTTAAACTTATTGCCCAATAAA 0.649702256789

GTTAAAGACTCTCTACAATTTC 0.438679824734

GTTAAATCATCTGCTGTAAAAA 0.127515616233

GTTAACATGAGTGTTTCTCCAG 0.516460562607

GTTAACTAAACTGGATTAATAC 0.366323293068

GTTAATCGCAGTGATCGTGCAT 0.574247745702

GTTAATCGTTCTCCAGCAGCAT 0.433611354886

GTTAATTTAAGACCATATAAAG 0.509397756903

GTTACACCGACTCCTAGCCGAG 0.630831056774

GTTACACGAAATCCTGCGGAAT 0.570047758603

GTTACACTAGGCGGTGGGTTTT 0.479650862537

GTTACACTGTTTGTTGACAGAG 0.20340441944

GTTACAGAGTGTGATACCAATG 0.649637160031

GTTACAGTAACTTCTAAACGAT 0.624177483028

GTTACATAAGATGAAAGTTAAC 0.229755976168

GTTACATAGTGTGCCCCATAAG 0.498621024079

GTTACATATTGACTTCTCAGTG 0.492805103733

GTTACGTGCACTCTACTATCAA 0.486241240342

GTTACTCACGCTGCTTTATCAT 0.512962882883

GTTACTCCGCGTGATGGTAAAA 0.69267175129

GTTACTCGCGGTCCCTTTGAAA 0.955928864228

GTTACTGAAACTCATTCACCAG 0.362400432234

GTTACTGAATGGCCTACCTCAA 0.608076183784

GTTACTGACTGCCTTGGATGAT 0.644022829837

GTTACTGCACCACCGTAGCTTA 1.03553562876

GTTACTGCATATCGTAACTGAA 0.69528116753

GTTACTGTCTCTTCTAACTAAC 0.450736384887

GTTACTTAATCTCCATGAAGAT 0.548098372925

GTTACTTGGTCTGATACAGCAA 0.733882273336

GTTACTTTTGCTCCTTGACCAA 0.436442090064

GTTAGAATACATCCTAACTTAG 0.265517077303

GTTAGAATGTGCCCACCAGAAT 0.654054192808

GTTAGACCATCTACAAGTATAC 0.428271277531

GTTAGACGAGCTGATCTCGAAT 1.03297869214

GTTAGAGACAGTGTTTTCTTAG 0.555401357877

GTTAGAGGCCGCGACGCATGAG 1.16673364285

GTTAGATAAGGATCTGCTCCTT 0.304662749891

GTTAGATCTGTTGAAATTACAC 0.280532450222

GTTAGCCAATATGTTCACACAT 0.648330639939

GTTAGCGCATCTCCTCGAAGAC 0.34888343842

GTTAGGCCTGCTCAATGTGCAC 1.13957301713

GTTAGGTAAGCTCCGTTAGAAC 0.457832486815

GTTAGGTGATGTGGTGTCATAC 0.29263624203

GTTAGTGCAACTGCTGCGATTT 0.471207936833

GTTAGTGCACCTCCTTTGAAAT 0.447304970572

GTTAGTGGATCTGGTGAACAAC 1.19503363785

GTTAGTTAATCTGATGTAAAAC 0.208849996252

GTTAGTTATGATCATAACGTAC 0.337941033343

GTTAGTTTTTGTGAGTGAATAA 0.210930833006

GTTATAGCAGGCGATGTACGAG 0.413231714212

GTTATAGCATGTGTATATCTAC 0.270842085173

GTTATAGGAGGTGCATAACTAA 0.375625374522

GTTATCGTTTTCTGTCAACAAG 0.81057640833

GTTATTTGGTCTCCAGCGAGTG 1.51646464397

GTTCAACTACTTGAGGAGCGAA 0.615547066766

GTTCAATGATGTCCTCGTGTAG 0.30106309726

GTTCATCAAAGTTATGAAACTG 0.591229836135

GTTCATTTGTGTCATGCTATAG 0.547183730912

GTTCCAAAAAGTGGAAGCGTTG 0.642960481695

GTTCCACACTCTGGTGAACAAA 0.324405169094

GTTCCACTATATGCGAGCAATG 1.1724804139

GTTCCATGATCTCCGAGCACTC 0.775767395126

GTTCCCGGAAGTGGTGGCTAAG 1.32390595703

GTTCCTCAGTGTCATTCTGTAG 0.594568106763

GTTCCTCTCGGGGCATACAAAT 1.42299558357

GTTCCTCTCTCTGCAACATCAG 0.378992486203

GTTCGAATAAATGAAGGAGTAC 0.284566781199

GTTCGACAACCGGATTTGCTAC 0.686466784585

GTTCGACTATTAGAATAGTCAC 0.419284492544

GTTCGACTCTTTGAAAAAAAAG 0.44604485013

GTTCGGCTGTCTGTTAGAGGAG 0.161890933443

GTTCGTCAGCATGGTAATATAG 0.565071117578

GTTCGTCTCGCTAAGGAGTAAG 0.850110491825

GTTCGTGAGTGTCCATAAATAT 0.611365287043

GTTCGTTGTCATGCTTAGTTAC 0.553060110286

GTTCTAGAAGGTCATACAAAAG 1.33170048115

GTTCTTCTAAAGGTAGAATAAC 0.285802001537

GTTGCACCATCTGATAACTGAG 0.177461793245

GTTGCACGTACTCAGACATTAG 0.397372212385

GTTGCAGCCACAGAGTGTCTAG 0.287462401015

GTTGCAGGAACTGCTGCATGAG 0.80628062115

GTTGCCGATCCAGCTCTGAAAG 0.590114547226

GTTGCTACCTATCCTGGAATAC 0.781174340192

GTTGCTCAATCTGGTACCTAAT 0.636246857066

GTTGGCGCGACCTCTCCGCCAA 1.87548774092

GTTGGCTTAGTTTAGCGCTGAC 1.45080295993

GTTGGTGTCATTGTCTGGCAAC 0.892387217362

GTTGGTTTTTCGGCATGAGAAG 0.947008401695

GTTTAAAAGTCTCAATTACCAT 0.439922284602

GTTTAAGTACATCATAATATAC 0.457898348277

GTTTAAGTAGCTGTAGTCCGAA 0.331588117635

GTTTAATCATTAGCTCGGGTAC 0.743408585823

GTTTAGCGCGCGAGAGGTAAAC 0.294815712601

GTTTATGAGCGTGTACACAATC 0.475875783753

GTTTATTAAACTTACTTAAACG 0.309307894173

GTTTCCCGCGCTGCTGCACATG 0.305847783349

GTTTCCCTACTTCACTAGGAAC 0.35273700018

GTTTCCGACTCTGATGGCTTAC 0.79781264846

GTTTCCTTACCTGCATCCATAC 0.447595571528

GTTTCCTTCGCTGAAGAAAATC 0.459997557192

GTTTCTCAGTCGGAAATTCAAT 0.594804489404

GTTTCTCCACCTCCTATGAGAT 0.513051814468

GTTTCTCCCTGTCCGAACCTAT 0.630989962212

GTTTCTCTACCTCAGTCAGAAA 0.530132248267

GTTTCTGTAATGCCTATTCATC 0.271204866307

GTTTCTTAGGCTTCAGGTGTAG 0.854728013472

GTTTCTTAGTAAGGTGAGAGTT 0.422926051548

GTTTGACAAGGGTTGCGTTCAC 0.44199660977

GTTTGACAATCTCTTGACTAAT 0.218355940017

GTTTGACTTAATCATGGGTAAT 0.376000361004

GTTTGAGAAACTGAAAATGAAC 0.54834414348

GTTTGAGAAGCTCTTTGAAAAA 0.519004804165

GTTTGAGCCGTCGCGACAACAG 1.1753605435

GTTTGATAGTGTTGTTTTAAAA 0.495369625514

GTTTGATTTACTCGTCAATCAT 0.375616727068

GTTTGATTTACTGTTAAGAAAT 0.28129711381

GTTTGATTTTCAGGTTACATAC 0.582647074115

GTTTGCCAGTCCGAGGACATAC 0.469074288929

GTTTGTAATCCTGGCTCCGTAG 0.782253647949

GTTTGTGTTAGGCCAATCTGTC 0.498558205548

GTTTGTTCTGTTGAATGGTTAT 0.223892419796

GTTTGTTCTTTTCACGTCGCAC 0.879401634942

GTTTGTTTATCTCCCCTTTAAG 0.330516384957

GTTTTAAGCGTAGATCGTCAAA 0.508060870014

GTTTTAGAACGTGTTGTTAGAA 0.641974320253

GTTTTATAATCAGCTAGTGTAC 0.837523008652

GTTTTGCACTCTGATGCTAAAA 0.452705020627

GTTTTGGACAGTGATCAGAAAA 0.760331994318

GTTTTGTACTCTGTTTGCTTAC 0.59663969487

GTTTTTGGGAGTCATAAAATAT 0.311210340605

TAAAAAGTTAGTTCTTCCAAAG 0.370067570186

TAAAATTTGGCAGTCCGAGCAG 0.345976929317

TAAACACCATGTGGAACTCAAG 1.1476650675

TAAACAGGCGACGCAAGTACAC 1.38412343443

TAAAGTCTGTGTTCCGAAGCAT 0.809751171001

TAAAGTGAAAGTCGTCCCGCAT 0.550927939375

TAAATTTCATGGGACGCCAAAC 0.480565697416

TAACCTGCATGCCGAAAACCTA 0.829586947308

TAACTTGGATCTGACGTGTATC 0.335739627804

TAATCATTACGTTCTCTAACAC 0.135326685663

TAATGCGCTACTACTAACGGAG 0.630711139658

TACAAAGAAAGTCAATCGAAAT 0.343816239852

TACAAAGAATTTCGTTCCGGAC 0.80820740637

TACAAAGACAGTGTGCTTCTAG 0.334174406103

TACAAAGATTCTCCCTATGATG 0.454652807966

TACAAAGGAGCACCTGGGGTAG 0.625829582954

TACAAAGGCGGAGATTTAAGAA 0.170226184939

TACAAAGTGCCTGCTATGATAA 0.491488846608

TACAAATAAGGTCCCGGGATTC 0.429481831055

TACAAATCACGTGTTCACCCAG 0.374505183284

TACAAATTTTCTGACACTCCAT 0.602536531134

TACAAATTTTGTGATTTAGAAT 0.391809413428

TACAACGATTGTGCCAATTAAC 0.935726603553

TACAACGTATACCAATCGCCTA 0.469339008217

TACAACGTCTCTGGTCACGTAG 1.187648452

TACAACTTGTTTCCCGGGTCAC 1.05774518175

TACAAGAAAACTGTTAAGGATA 0.332537852017

TACAAGCATGGTCGCGCGGGAG 0.333440894035

TACAAGGCAGGTGGTCGGTAAT 0.938557345991

TACAATCCGAGTGCTGTTCCAC 0.901844838207

TACAATGACTGGGAAAATTAAC 0.483606274073

TACAATGGCTGTCTAGGAAAAC 0.954498702918

TACAATTGATTTCAAGTCATAT 0.349390521243

TACAATTTCCATCAAGACTCAC 0.594270028855

TACACACAAGCTGATGTCGTAT 0.516535206562

TACACACAATTGTACTAAATAC 0.209817779351

TACACACACTGTGATTCGTAAA 0.591394853824

TACACACGTCGCGAAGTATTAA 0.902173853156

TACACACTGTCTCATGTTTAAT 0.511363143815

TACACAGAGAATTCCCCACTAC 1.67814382173

TACACAGCCTCCGGATGTACAA 0.54872054947

TACACAGGCTCAGACCGTGTAC 0.368089696164

TACACAGTACGGGCTAAAAAAC 0.854130885182

TACACAGTAGATCATTAGAGAG 0.504750900985

TACACAGTATCTCATTGGTGAC 0.886607149494

TACACAGTGTCTGAATGATCAG 0.865206895474

TACACATAACTTGGAAAAACAC 0.609729524157

TACACATATACTGGACTGTAAA 0.189371175683

TACACATCCAGTGATGGAGTAG 0.534305575663

TACACATCGTCGGCTCAAAAAT 0.227101928759

TACACATGATGTGTATGTGATT 0.289446695539

TACACATGGCGTGATAGCATAT 0.548112385894

TACACATTAGTTCAATAGCCTA 0.410066722461

TACACATTCGATCCTGATTCAA 0.314139127525

TACACATTCTATCCTACGAAAG 0.198681322555

TACACATTGACTCAATACCCAG 0.546665039566

TACACATTTTTTGTTGTCTTAC 0.499169391495

TACACCCACGGACAAACTCATC 0.53771891193

TACACCCTAGGTGAGACCTGAG 0.811696054383

TACACCCTGTTTTCCAGTGAAT 0.597580351269

TACACCGACTCTGATGCACTAT 0.95343534106

TACACCGAGTCTGCATCGCTTA 0.526863499741

TACACCGGTGATGTTTCGAAAA 0.603487790846

TACACGCGGACTGCAACGATTC 0.873447257797

TACACGGAGACTGCTGGTGAAG 0.968359345294

TACACGTAAAGTGTAGGGGGAG 0.662250754258

TACACGTGATGAGACAGATTTA 0.856996587697

TACACGTGTCATCAATGGATAT 0.676717800821

TACACTCAATGTCCTGTCAAAT 0.718537499001

TACACTCATTCTTTTTTAGAAG 0.397400848852

TACACTCGATATGAAATTGTAG 0.626037003595

TACACTCTAGCTGAACCATGAT 0.564122263922

TACACTCTGCCTGCTATGGAAG 1.00697270856

TACACTCTGGGTCCTAACCTAG 0.792391922723

TACACTGAATATGATCCAGGAG 0.476363709839

TACACTGGAACTGAAATATGAG 0.495813556259

TACACTTACACTGCTAGATTAG 0.350650144454

TACACTTACTATCTTAGTGCAA 0.720057805171

TACACTTATTCTTAAACGCTAA 0.347332254361

TACACTTCAACGCATCGTAAAG 0.417712193221

TACACTTGGTATCAAGTAGTTC 0.292885872923

TACACTTTATCTTCTTGCAGAT 0.302340086837

TACAGACAACTTGTGGCTTATG 0.202009592191

TACAGACAATCTTATGGGAGAG 0.383031726072

TACAGACACCTTGTAGTAAGAG 0.394605647525

TACAGACATTTTCATCGGGAAG 0.505928996793

TACAGACTATTTCATTCAATAG 0.526986956932

TACAGACTCTCCCCAAGTTGAG 1.10833792656

TACAGAGAAATTGATACACGAG 0.251072544986

TACAGAGACTGTGATCATGCAC 1.12114464027

TACAGAGAGCGAACTTCATTAG 0.413545321718

TACAGAGATACTCAGAGATGAA 0.684835462226

TACAGAGCAACTGGATGTAAAT 0.372352772554

TACAGAGCGTGTGATCTTGTAC 0.660205773689

TACAGAGGCTCTATTACCCTAG 0.683779808792

TACAGAGGGAATTATTAGACAG 0.392056518446

TACAGAGGGCGCCCAGGTGTAG 1.80091845209

TACAGAGTATCTGGACAGCTAC 0.761196936698

TACAGAGTATTTGATTTTCGAG 0.866628406029

TACAGAGTGTATGCCTATTATG 0.299597208256

TACAGATAATCTCTGTCTGTAA 0.550710255951

TACAGATATTCTCCAGAGATAG 0.228862468279

TACAGATCGAGTCCTCAAGAAG 0.607894785441

TACAGATTTTGTCCATCAATAG 0.175856653859

TACAGCATGCGTGCAGGGGGAA 1.22487525575

TACAGCCGGAGTGAATCGGGTG 0.68727962666

TACAGCCTACTTCCGAACCTTG 1.05806346843

TACAGCGAGTGTGCATCCTATG 0.239441737584

TACAGCGTGTGGGGAGCATTAG 0.923702737123

TACAGCTAATGTGACTGTAGAG 0.992467230239

TACAGCTTACCTCTAATTCGAA 0.664196021455

TACAGCTTATATCGATCAGAAC 0.45533342224

TACAGGTAATCTCACTGGTTAA 0.144810169148

TACAGGTAATCTGCTTGCTAAT 0.589920769002

TACAGGTCGGGTTATGTCTGAT 1.13024801894

TACAGGTTCAGCCGTAACACAA 0.292835303805

TACAGGTTGTGACGCATCTAAC 0.384826313273

TACAGGTTTAATGCAAAAAGAT 0.405069013879

TACAGTCAAGATGACGCCGGTG 0.54656949049

TACAGTCAGTCAGATTCTAATG 0.440047441288

TACAGTCATTCTCGTGTATGAT 0.649932053021

TACAGTCCATATTACAAAATAC 0.62906575232

TACAGTGATTGTGATCACGTAG 0.447708646181

TACAGTGCCTGTTATATGCATC 0.664530622062

TACAGTGCGTCTGCAACAGCAT 0.791971497865

TACAGTGGCTGTGATAGGAGAC 0.623964500352

TACAGTGTACCGCGACCAGCAT 1.29767218735

TACAGTGTACTTCGTCTATAAG 0.516229234742

TACAGTGTAGCTTGAACGGTAA 0.317054194289

TACAGTGTCCCTCATCCGTCAC 0.49318785353

TACAGTGTTCGTCATGTTGTAA 0.617472075918

TACAGTTAAAGTGATATTCTAT 0.591193823655

TACAGTTACTCGCCTTGGTCAG 0.153603329588

TACAGTTATTGGCATCTTAAAC 0.635627979894

TACAGTTCATGTGAGGTGAAAC 0.369675537364

TACATACGAACTTCAGCATGAG 0.698274940363

TACATACTAAGTCTAGGATCAG 0.19392929549

TACATAGAAACTGATTTAGGAC 0.417440598273

TACATAGACACGCATCCGAATC 0.465206064064

TACATAGCCCTTTTACGACAAC 0.452541784775

TACATATGATATGATGGATTAT 0.206547350541

TACATCGACTTTGCAATAACAG 0.482834137982

TACATCTAAGATCAGGAGCAAG 0.371279769675

TACATTGCAGGTGCACAGTGAA 0.48752480147

TACATTGTCTATGCTATGGCAC 1.24810138967

TACATTTAAAATCCGCCCCAAT 0.842863761937

TACATTTTCAATGAGCATTGAT 0.258717000293

TACCAACGGAATGTGCTGTGTA 0.262815069402

TACCAAGAAGCTGAAGGGACAT 0.34564305959

TACCAAGCGCCTTATTGACCAG 1.04503748587

TACCAATTATTTCATTCTTCAT 0.379353957119

TACCAGCTTCATCATTAAAAAC 0.426460574618

TACCAGGGCGATTTTATACCAC 0.982848453644

TACCATCAATTAGGATCAAAAC 0.318946866802

TACCATGCTCGTGATGAACCAC 0.557052442562

TACCATGGATCTGCTCGACCAC 0.548152869202

TACCATGTATTTGATAGCTAAT 0.614000693935

TACCATTAATGTGCGAGCATAA 0.219492066522

TACCCAGAATGTGAACTGCAAG 0.932325890885

TACCCAGAATTTGCATAGAAAT 0.503921999067

TACCCAGACTGTGTGAAGGTAT 1.07040354658

TACCCAGCGTATCAATTACAAG 0.29353959853

TACCCAGGTTATGATGCGCATA 2.44804917274

TACCCAGTACCTCCCAGCTTAA 1.03800928978

TACCCAGTCTCTCAGATCTTAG 0.618356749319

TACCCAGTTGGGCCGGATTTAA 0.827716789118

TACCCATAAATTCGTGCCAGTC 0.493411856117

TACCCATAAGTTGTTATACATA 0.588466352926

TACCCCCACTGTGCTCCGCAAA 0.328745249015

TACCCCCGACGTCATATTGAAT 0.664070758778

TACCCGCAAGCTGTCCCCACAC 0.848391422558

TACCCTAGTATTCAGGGCAAAG 0.582110125442

TACCCTCAATGGCCTACCTCAG 0.597901140485

TACCCTCAATTTGCTTTAGGTG 1.06725189135

TACCCTCCTTGTGTATATTTAG 0.63830379359

TACCCTCGAGCTCAGAACTAAA 1.08023222429

TACCCTGGCCGTCGGGAGTAAC 1.04549487976

TACCCTGGGTATCTCTAACTAG 0.378504331057

TACCCTGTAGCACCAGTCTTTT 0.543654075801

TACCCTTCGTCTGCTAACCGAC 0.614385470807

TACCCTTCTGTTCATCAACCAT 1.00954209059

TACCCTTTCATTTAACGATTTA 0.772402562166

TACCGACAATCCCTTTATCCTC 0.28133776291

TACCGACAGATTGCAGTCATAC 0.234528177978

TACCGACTCTCGGTAGTTGGAA 0.483489500687

TACCGAGGAGTGCATTACGCTA 0.183378016629

TACCGAGGCGGTGCGAAATTAG 0.555451303025

TACCGAGTAGTTCCTTGGTAAT 0.642059975688

TACCGATAAACTGAACAAGGAA 0.342057594076

TACCGATAATTTGTGCCATCAG 0.570505892935

TACCGATACTTGGCATCAATAT 0.22358899855

TACCGATCACATCCTTATAAAG 0.647176880539

TACCGATCACGTGCGAAAATAA 0.644025218785

TACCGATTATGTCACAGTCAAG 0.725644186799

TACCGATTATGTGTATCTCAAT 0.207536656889

TACCGATTGTGTGCTTCTCGAC 0.531352823922

TACCGATTTGCTGATCTCTAAT 0.888149057271

TACCGCCACTCGGTTTAGTTAG 1.01353566144

TACCGCGCGTCTCCCAGTCAAG 1.3210523753

TACCGGATCTCTGGATGGCGTC 1.06155607464

TACCGGCGGTGTGCTAAGGTGC 0.840222075887

TACCGGGACTCTGATCATGGTT 0.932575630544

TACCGTATGAGTCGAGAGAAAA 0.501440919269

TACCGTCCCACTCTTGGACCAT 0.438696565607

TACCGTCCTGATCCTTCGAATA 0.817792226168

TACCGTGCTTGTGATAGGCCAA 1.23957095848

TACCGTGTGAGTCCTCAGTGAA 0.720229634335

TACCGTTTTATTTCATAAGAAA 0.410202253037

TACCTAGGACGTGCATCTAAAA 0.257249366627

TACCTATGATGTCAACTATAAA 0.54934670124

TACCTCGAACCGGCTGTGCAAT 0.833649864707

TACCTCGAGTTTCAACCTAATT 1.15865578008

TACCTGGCCTCTGCAGCAAATT 0.778733046645

TACCTGTTGTGCGGACGATAAC 0.7846732762

TACCTTGATTCTTAGCCGTTAA 0.591518392081

TACGAACAGTGTGTAAAAAAAG 0.436503800663

TACGAAGAACTTTATACTAGAG 1.66009302862

TACGACGGGGCTCATAATCATA 0.730144186005

TACGACTTATTTGCTAAATGAA 0.526358082275

TACGATCCAAGCCCGGATTAAA 0.537051372576

TACGATGGAAGTCGTCGCAAAC 0.704732969878

TACGATTTTTCTGGTGTATAAC 0.599270755431

TACGCACACACCGAAGTGCAAG 0.2481919356

TACGCAGACTGTCATCCAATAG 0.600106140481

TACGCAGTACCTCCGTTCGTAC 1.02589490326

TACGCATAATCAGCAGTTGAAA 0.11994915181

TACGCATAATTTGCTTGGTTAG 0.770565480968

TACGCATCATGTGCATCTTTAT 0.581827380174

TACGCCCACTTTGTTCCGGTTG 1.25541712863

TACGCCCAGACGGAAGGGTATC 0.85351569322

TACGCCGAATATGCAGACGGAT 1.27354792172

TACGCTCACGGTTCCCTGGGAA 1.31316160091

TACGCTCCAACTTATGTACCAG 0.496450766334

TACGCTGAAATTGAGTAAGTAA 1.01870695117

TACGCTGAATTTGAACAACGAG 0.340301378212

TACGCTTACGCTGCCTTGCGTG 1.16296719132

TACGCTTCTGTTGTAAGCCAAT 0.417669055492

TACGGAAGCTGTCGACGCTGAA 0.749219564913

TACGGACACTGTCCAAGGACAG 1.24465904755

TACGGACGCATTCATTCACGAA 0.529269240873

TACGGATAGGTTCCTCGATGAT 1.12387052754

TACGGATCGCCTCGTAAATAAA 0.77967093784

TACGGATCTGCAGGTCGGGAAT 0.763274367563

TACGGATGGTCTTTTGACAGTA 0.709890638032

TACGGCGAATCTGTTCGTAATG 0.656381678149

TACGGCGTCTGGGTGTATTGAC 1.36984652775

TACGGTATTAATGGTTACAAAT 0.283987284121

TACGGTCCATATCCTAGTGAAA 0.768821012513

TACGGTGAAACTGATAGCTCAG 0.509909273424

TACGGTGAAATTGATTGATAAG 0.330162150162

TACGGTGCAAGGGATACCTTAG 0.615846401926

TACGGTGGCTATGTTTGGAAAT 0.804105863728

TACGGTGTGTTTGCGGTCGATT 1.01772072783

TACGGTTGTCTTGGTGTAAAAC 0.42487277456

TACGGTTTAAGTGAAGCGCCAG 1.18723086789

TACGTACGTCGTGCTTTAATGG 0.668246072346

TACGTCTAAGCTTAAAGAAAAG 0.556320286332

TACGTGCAAGATTATGCTCTAC 0.565842441516

TACGTTTTAGGTGCTAATCGAC 0.8836860518

TACTAACACCCTGATTCGCGTT 0.334839531608

TACTAACCATGATCTCCCCAAA 0.224438729448

TACTAACCCTGTTGAGCGACAC 0.812106662359

TACTAAGAATGTGATAGGCATC 0.738424458955

TACTAAGACGTTGTTAGGTATC 0.335805889167

TACTAAGAGCCTCCCCAGTGAG 0.981246852051

TACTAAGGCTGTGCTACTTGTG 1.10819053725

TACTACAAATCCGCAGATGCAA 0.545107396586

TACTAGGAGGGTTGATAAGGTG 1.17639912966

TACTAGTCATGTCATTGTACTC 0.373047136661

TACTATCATATTCGACGTCTAG 0.823002715672

TACTATGAAGCACATCCTAGTC 0.400984652789

TACTATGGGGGTGTAAGGTTAA 0.459498342331

TACTATTGATGTGCCGGTGTAT 0.946138572018

TACTATTTAACTCAACTGGAAA 0.383258260435

TACTCACACGCTCCTCATTGAC 0.183420517047

TACTCACAGCGTTAATTCTTAG 0.804253329283

TACTCACCCTGTGACAAGCGAC 0.670958872918

TACTCACGTTGTGCTAACAGAC 0.499586972298

TACTCACGTTGTTGTGTGTAAA 0.536804359112

TACTCAGAATCTGGTACTTGAC 0.537762621618

TACTCAGACAGTCGATCTGTAA 0.514664092698

TACTCAGACTCTGTTAAGCTAT 0.262930531742

TACTCAGAGGCTGTTCAAGAAC 0.791787859672

TACTCAGATTGTGAAAGTTGAA 0.945399132879

TACTCAGCATGTCCTATGGTAA 0.421947143022

TACTCAGCTTGTGAAGAGGAAC 0.445827009633

TACTCAGGGCGTCATTCCACAT 0.270699216537

TACTCAGGGTGTTTTCGGAAAG 1.0940015942

TACTCAGGTACTCTAGCATAAG 0.545609718932

TACTCAGTATCGGCTGCGGAAG 1.58406046579

TACTCATCCAATCGTTGGCCAT 0.801740967039

TACTCATCCAGTGAAGCTACAC 0.745068928429

TACTCATCTTGTCAAGTACGAG 0.254863942819

TACTCATTATCTGCAACATGAG 0.743599216959

TACTCATTATTTCGCGCTAGTC 0.411416580003

TACTCATTTACTCCTGGGAAAA 0.176348197837

TACTCCCTGTCTCATCCTCTAG 0.676719842347

TACTCCGTGCGTGTACATTGTC 0.825598729414

TACTCCTAAGGTCGTAAGTGTA 0.860060238443

TACTCCTGATATCGATAGTGTC 0.660226738664

TACTCCTTATCTGGTGCAAAAT 0.202878107623

TACTCGCTGCATCCGCCATAAT 0.527614998982

TACTCGGCCTCTGATGATTAAT 0.362622845221

TACTCGGCGCGTGCCGGTGCAA 0.993360914178

TACTCGTTTCCTGTATATTTAG 0.27888726145

TACTCTCACCGCGTACTTTAAA 0.600759895803

TACTCTCCATTTCTCGAGACTG 3.00932329928

TACTCTCCCTCTCCTAGGATTT 0.511588267058

TACTCTGACGGTGGTCTCAGAT 1.66310683608

TACTCTGACGTTGATCAGCAAT 0.827838475733

TACTCTGCATGGTGTATTAGAG 0.6116925172

TACTCTGCCACTCCTTGACCAG 0.471290383855

TACTCTGTGCCTGCCGGGAAAA 1.24401057719

TACTCTTACTCTGCAAGTAAAC 0.532423819092

TACTCTTATGGTGCATCTCGTG 1.49956325846

TACTCTTCATGTGTTTACTTAA 0.364982613936

TACTCTTCGGATTCCAGTGATT 0.185238340254

TACTCTTCGTTGGATTGAGAAA 0.451636019184

TACTCTTTATCTCGTGGTAATA 0.455097081169

TACTGAAGGAGAGATAATGCAC 0.262368433736

TACTGAATACCTCATGCGGAAC 0.46259408687

TACTGAATATATCCTTAGTCAA 0.57659065402

TACTGACACGGTGAACTTTTTC 0.52544437881

TACTGACAGCTTGAGTCTGATT 0.478424029775

TACTGACCATATCATTCCAGAG 0.18947306702

TACTGACCGTGTTGTGGGCTAG 2.00492834436

TACTGACTCTCTGCTTAGGAAG 0.458312534205

TACTGAGATGGTCCTGTTCATT 0.614704085902

TACTGAGGATGTCGTGTGCTAA 1.27840933364

TACTGAGGTTCTCAATAGTATG 0.630895390308

TACTGAGTCTCTGCTCAACCTG 0.690214140451

TACTGAGTGAGTTTAGCAAAAT 0.53274625346

TACTGAGTGCGTCCCCGGCGAG 1.23040082782

TACTGAGTGTAGCGTGCTAATA 0.653143901346

TACTGATATAGTCTTGACTAAC 0.48714096796

TACTGATGAATAGAGGTTATAC 0.654769678996

TACTGCCAATATTCCGGAGTAG 0.799414762293

TACTGCCAATCTCTAGACCTAG 0.558880644663

TACTGCCATTTTGCTGATCTAA 0.667016595807

TACTGCCTCCCTCATGAGTGAA 0.731536206423

TACTGCGAATCAGAGTCTGTAG 0.614046747315

TACTGCGCCCCTAATCGACATG 0.98236010085

TACTGCGTAAGTTGCAATGGAG 0.858153901442

TACTGCGTACTTCATTCGCTAG 0.295611551715

TACTGCTAAAGAGGTATCTTAT 0.549055456378

TACTGCTAAGCTCCATTAAAAT 0.48616545075

TACTGCTACCGTCGTAAGACAT 0.38671738826

TACTGCTGGCTTCATGCGTAAG 1.21521094382

TACTGGCATGCTGCTGCAAATT 1.35665874835

TACTGGGGAGGTCCATCATTTG 0.734475618067

TACTGGTAAGCCCATCTTTGAC 0.47861879307

TACTGTCACTCTTCGCGGCTAT 0.565505358318

TACTGTCACTTTGTTGATTCAG 0.937845321678

TACTGTCAGGCAGCTAAGTAAG 0.574237183669

TACTGTCCGTGTGGTGAGAATC 0.579857209637

TACTGTCCTACTATACAGCATT 0.57857000515

TACTGTCGGCCTCTAACTACAC 1.30007599475

TACTGTCTATAGGATGAGTCAT 0.93666499005

TACTGTGATTATGGAAGTGAAT 0.304159909391

TACTGTGGTGGTTATAAGGCAG 0.670696886969

TACTGTGTCCTTGCTATACTAA 0.174949335724

TACTGTTAATGTGGTCACAGTC 0.923858677444

TACTGTTATGCTGGCACGGCTT 0.186199388241

TACTGTTGACATGGTGAAGAAG 0.446680882244

TACTGTTGCTGGTATTTTCCTG 0.914354979059

TACTGTTGCTGTAGACCTGGAC 0.588465286554

TACTGTTGGACTTCCTTCACAA 0.672780819892

TACTGTTTCACTCGAAACTTAG 0.237523902818

TACTTAGTTTCTCATGAAGAAT 0.404403323745

TACTTATAGATTCGTGAGTTAG 0.222690729113

TACTTATTATGTGATGGCTGTG 0.563720052187

TACTTATTCTTTGCCAGTGGAC 0.901761016086

TACTTCGAACGTGTTACCCTTA 0.62715473671

TACTTGGTATGTTAAGGTTTTT 0.457133524412

TACTTTCTCCGTGATCACAGAT 0.43010509555

TACTTTGCCGATGATAGAGTTC 0.499286862434

TACTTTGCCTCTGATAACAGTC 0.373907631981

TACTTTGGAGCTCCAGTCGCAA 0.4251107445

TACTTTTACCCTTGTATTAGAC 0.61752265837

TACTTTTATTCTCCAGATGGAT 0.549966692697

TAGAAACACGGTCTCCACTAAT 0.493386478688

TAGAAACTGTTTGCTTCTTCAG 0.577110190046

TAGAAAGCATATGACGCCGTAG 1.0616994706

TAGAAATGAACCGCAAAGGTAG 0.821853594188

TAGAATCGTCATGCTCCCCAAT 0.527667056471

TAGAATGTGGTTGCCACACAAT 0.875293266771

TAGAATTACCAGGTCTAGGGAG 0.974940757706

TAGAATTAGAGTCCTCAGATAC 0.387979320452

TAGACAAACTGTCATACGTAAG 0.313692253724

TAGACACAATGTGAAGATTGAT 0.497296585855

TAGACACTCCCTCCTACATCTG 0.777832825869

TAGACACTCTGTCCTTGTTCAA 0.735394123709

TAGACAGAATTCGTAAGGTTAG 0.696149023726

TAGACAGATGCTGTGATTTAAA 0.400979851899

TAGACAGCAGCTCATGTTCTTA 0.341919930883

TAGACAGCATCTGCTCGTTAAC 0.475366990228

TAGACAGCCTGTGAAGTGGAAG 1.10503666009

TAGACAGGACCTCATCATTAAA 0.219415457151

TAGACAGGTGCTCCATGTTCAC 0.541451822512

TAGACAGTAGCTGAGGCTGAAC 2.00070113385

TAGACATAAGCTGCTTAGTCAG 0.448257651375

TAGACATATTGTCTACCTTAAT 0.441292842682

TAGACATCAGGTCGTGGCGTAT 0.801985686427

TAGACATCCTGTGTTGCCTTAT 1.11815058703

TAGACCATAGCTGGTAGCTCAG 0.470327924128

TAGACCCTCGGTGATTAATAAC 0.407313483095

TAGACCGAGACTGGTATTGGTA 0.692006863056

TAGACCGATGCTGGTTGTGAAA 0.616142226505

TAGACCGCGGCTCTTGAGCTAA 0.739197005463

TAGACGCGATCTCCTTACTATT 0.68099799522

TAGACGCTGGGTGATAGTCAAC 2.18792362842

TAGACGGAATGTCAATCGGGAT 0.792504294968

TAGACGTGGCCTGATTACCGAA 2.07706903736

TAGACTCACCAGCATTTATCAT 0.540024316449

TAGACTCACTTTGACTAGTTAG 0.284388670563

TAGACTCAGTCTGCTGGGATAA 0.517681452032

TAGACTCATCCTGTTACGAGAA 0.323385393982

TAGACTCCAACTCGAGGTTAAC 1.36760940401

TAGACTCGTTCTGCCAACGTAT 0.840258200667

TAGACTGCTGGTGCAAGTGAAC 0.980549763395

TAGACTTCATCTGTGCTAGAAT 0.589745129392

TAGACTTTACTTGATTAATTAC 0.18174153728

TAGAGAAGAAATGATCTGCGAG 0.27435237611

TAGAGACAATGCGCCTGCTCAA 0.301406312958

TAGAGACACGCTGATTGTAAAT 0.339824610347

TAGAGACCATATCCTGTTTTAA 0.630485800436

TAGAGACCATGTCGATGTATAG 0.254938110576

TAGAGAGATTGTCAAGATGGAC 0.226051496486

TAGAGAGCACCTGTCAAGGAAG 0.992448012583

TAGAGAGCCTATCTAAAGTGAA 0.37898690439

TAGAGAGTGTACCTTCTGGAAT 0.381941262823

TAGAGATAGAGTCTAGCCACAG 0.3272409809

TAGAGATATGCTCCATGATAAC 0.394166910925

TAGAGATCATGTGATAACGTAT 0.698467582163

TAGAGATGAACTGCAGCAGCAC 0.448538109709

TAGAGATGCGCTTCTCAATAAA 0.73675591622

TAGAGATTACGTGATCACTTAC 0.550521955856

TAGAGATTCAATCAAAAAGTTA 0.483899708579

TAGAGCCAACATGCAAATGAAG 0.444326151978

TAGAGCGAAACGTAAGATCGAC 0.748921558667

TAGAGCTAGTATTTCGTCGGAT 0.39244918458

TAGAGGCTACGTCCTTAGACAT 0.192675893959

TAGAGGTAGTGTTATTTGTATG 0.392339365096

TAGAGTCCACGTCCGTATTTAG 0.313866828527

TAGAGTCCGGCTGCAGAGGTAG 1.65989945904

TAGAGTCTAAGTCATCCTACAT 0.382434125878

TAGAGTCTATGTGAAAGATAAT 0.365214024735

TAGAGTGAAACTGGTTGATAAG 0.547144073052

TAGAGTGAATCGTAAGAAAAAG 0.678169055705

TAGAGTGAGCGTCATCTGTCTA 0.468679273115

TAGAGTGATACTCTATGTCAAC 0.453091666104

TAGAGTGCGTCTGCTAAGGTAG 1.17270983457

TAGAGTGTCGCGGAAAGTTTAA 0.275418798125

TAGAGTGTTGCTGACCAGCTAC 0.851401499988

TAGAGTTGATCGGGCTTCTCAC 0.725209315273

TAGAGTTGATCTGGTCATAAAC 0.717958360999

TAGATACAATGCCATTCGTATG 0.584480440196

TAGATACTATCCGATAAACAAC 0.263093862534

TAGATAGAAGGTCGATTCTATG 0.805162052499

TAGATAGCTACTGACCCTTAAA 0.937422418281

TAGATCAGATTCGGGAGGCGAC 1.22756805772

TAGATGGGTACTGCTAGATAAT 0.477538608976

TAGATTGTGAATGTTGGTTAAG 0.561782281662

TAGATTTCATCTGCTACTTTTG 0.511164753647

TAGCAAAACCCTTATCGTCATC 0.371687000877

TAGCAACCATTTCTTAGCAAAC 0.430358548887

TAGCAACTACGTCTATCGCAAG 0.553644220234

TAGCAAGAGGCTGGAAATACAG 0.822782751094

TAGCAATAAGCGTGTTCACAAT 0.552547830992

TAGCAGTCTCGTTCTAAATCAT 0.396009016799

TAGCATCTATGTCGATTGTGAT 0.692937978102

TAGCATTCATCTTCAACCGTAC 0.701141654448

TAGCCACACACTCCGACTGAAA 0.196498915666

TAGCCACCCGCTCCAGAACAAC 0.612419234093

TAGCCAGAAGGTGTTTACGGAT 0.469637995786

TAGCCAGAATCTCGAGCAGAAT 1.38127874151

TAGCCAGAGTATGCAACATAAG 0.35618006591

TAGCCAGTATTTGGTATACGAC 0.444777363871

TAGCCAGTTTACCCTTAACGAG 0.267470206961

TAGCCATCCTGTGGGCGCCTTG 2.62790328801

TAGCCATCGTGTCAATCAGGTT 0.476667583956

TAGCCATTACTTTCTTGGCCAC 0.685848750469

TAGCCATTTTCTCGGCATACAG 0.318966418451

TAGCCCAGTGGTGAAAATTAAA 0.306161470102

TAGCCCCTATCTGCTTTAATAA 0.13912276247

TAGCCCTTGGGATCTGGAAAAT 0.731433019744

TAGCCGTTAAGTCAAATCATTA 0.455604717282

TAGCCTCCATTTCCTAATCCAT 0.676073413635

TAGCCTCCCTCTTCGACTAAAT 0.95814739861

TAGCCTCTATGTTATAACTGTG 0.313518148059

TAGCCTCTCCCTCATGAAGAAT 0.162692678006

TAGCCTGTACTTTAACGTCAAT 0.378756375739

TAGCCTTCAAGATCTATGGAAA 0.542964750798

TAGCCTTCCGGACGTCGGGGAC 1.7682564868

TAGCCTTGAGATCGGAAAACAA 0.588374539317

TAGCCTTTAACTGAATCAAGAG 0.840778077692

TAGCGACAATGTCCTAAACTTT 0.513636880105

TAGCGACCGTTTCACGCCGAAC 1.65959288619

TAGCGACGACCTCCGGTGAGAT 0.884239825274

TAGCGACGTTGTGATATGAAAC 0.36358592674

TAGCGACTATATGCACTCAGAC 0.795421152714

TAGCGAGACGTTCCTGATATAA 0.955035311283

TAGCGAGCGCGTCTATCGCTTT 0.684586673128

TAGCGAGGGCCACCTTCCATAG 0.991478814852

TAGCGATACCGTCCAATGTAAA 0.498231482533

TAGCGATCCTATGTTCCAGCTG 2.29218245894

TAGCGCGTAGATGCGCCGAAAT 0.528802087828

TAGCGTCACATTCCTGGAATTA 0.713483534969

TAGCGTCGCTGTGATATTTTAG 0.248241300035

TAGCGTCGGTGTGAATACCTAG 1.79198291614

TAGCGTGTAAATGAAGCATTAT 0.525169054275

TAGCGTGTTTTGCGTGAGATAT 0.601699437812

TAGCGTTAGGGTGATGAAGATT 0.427516102332

TAGCGTTGTTCTGATTGGTCAG 1.27566147215

TAGCTAAACTATTCTTACCTTC 0.373207758518

TAGCTATCAACCTGTCGGATTT 0.813056177142

TAGCTATTATCCGCAGTAAAAG 1.0131504332

TAGCTATTGGCAGGTGTTATAT 0.881070453777

TAGCTGGAATCTGTTGACGGAG 2.51015136803

TAGGAAGTGTGTCGTTTACCAG 1.08619633917

TAGGAATAACCAGATATTGAAT 0.552973444316

TAGGATTGGGGCGTTGCGGAAA 2.57050926259

TAGGCACAATTTCACCGAGGAG 0.613462559218

TAGGCACAGTGTCCTTGGAGTT 0.740149186566

TAGGCACAGTGTGACTTATATA 0.496206036997

TAGGCACGGTATGCAATCTAAA 0.504119413329

TAGGCACTACTTGTTATCCCAG 0.379335327647

TAGGCAGAGGGGTAATCGTGAA 0.790905510136

TAGGCAGGACATCAAGCTCTAT 0.364625889311

TAGGCATAACCAGGTGCGTATC 1.09476069773

TAGGCCGACTCTGCTTATCTAA 0.67769627652

TAGGCCGAGTGTGCGAGCATAT 1.13867921826

TAGGCCTCATGTGCGAGTATTT 1.44280281951

TAGGCGTTATGTGGCCTGTCAA 0.817453204745

TAGGCTCAATGTTTTCAGGAAA 0.917348857967

TAGGCTCGACATCCTCAATAAT 0.294512571274

TAGGCTGACGGTCAATAGGGAG 0.577452453353

TAGGCTTTATGTGTTTCCCCAG 1.29811776256

TAGGGACAAATTGAGGAATAAA 0.914725150454

TAGGGACTCTGTGGATCCTAAG 1.15390526455

TAGGGAGTGTCTTATGGGAAAT 0.736141468327

TAGGGATAAATTGTTCAGAGAC 0.629891076984

TAGGGATCATGTCATGCAGATT 1.03436868254

TAGGGATGGTACGCCGTCTAAC 1.31977471887

TAGGGCCGGGGTCATAACTGAT 0.250251195862

TAGGGCCTCCGTCATATGTGAT 0.566157858581

TAGGGCGGATCAGCTTCATCAC 0.797235755645

TAGGGCTAATGTGCAGGGATAA 0.823297917011

TAGGGCTCATCTGCTGATCCAA 1.22826850954

TAGGGGATTGCTCCACGATGAG 0.711210100892

TAGGGTGGCCTTCACGACAAAC 1.14908099505

TAGGGTTTATCTGATGTTCATC 1.01477469683

TAGGTTCTTTGTGAATACTAAA 0.469897934866

TAGTAAACACCCGGTCGTTGAA 1.07929569364

TAGTAACAAAGACTTGTAATAA 0.314998818877

TAGTAACCAGGTACTTGATAAG 0.503652511667

TAGTAAGATTGTCGTGCGGAAG 0.212540995646

TAGTAAGTGCGGTTTGCGCAAG 0.773513319239

TAGTACGGTTCTGAGTACCCAG 0.395647829483

TAGTAGTTACGAGTTAGGGAAC 0.277499115139

TAGTATATCTGTCCTACTTGAC 0.75256550987

TAGTATCTATTCCAATATTTAG 0.31512619912

TAGTATTCATAGGCATTGCAAT 0.459906342683

TAGTATTGGGGTCATATTGTAC 0.495736461671

TAGTCACAAGTTCCAGGTCAAC 0.624725767505

TAGTCACGCTATCCTGGGATAA 0.790477657032

TAGTCACTAGGTGATACGTGAG 0.289459110691

TAGTCAGGAGGTCCACGGGTAC 2.05012705843

TAGTCAGGATGTCTTAGGTAAG 1.03854013239

TAGTCAGTGCGGGACCCGTATG 1.97832798526

TAGTCATAAATCCCGCGTCTAG 0.47487349331

TAGTCATAAGTTCCTGATAAAT 0.224632516769

TAGTCATACTCTCCTGCGTTAA 0.816122159053

TAGTCATTGTGTCCAGTATAAT 0.712140447209

TAGTCCCAATAAGAAGTAGAAG 0.375524702364

TAGTCGAACTGTTCTCATTAAT 0.946551700513

TAGTCGCTGTGTGTGCACACAT 0.991274674943

TAGTCGGTGGGTCATCGTTAAC 1.06589374932

TAGTCGTACAGGGCACAGGTAG 1.09600460841

TAGTCGTGTCATCATCACAAAA 0.595055929021

TAGTCTCAGTCTGCTGGAACAT 0.713906992673

TAGTCTCCGAATTAGGATATAC 0.311428477052

TAGTCTCTTTTTGAAAAAAGAG 0.580473312687

TAGTCTGACTTTTCTACTGCAC 0.385743353006

TAGTCTGAGGGTGGAGTTGTAA 0.838881346109

TAGTCTGCCGCTGCCCGGGGAC 3.26392323183

TAGTCTTAAGGTGATAATTCAT 0.520532616802

TAGTCTTATGCTGCATTGTAAT 0.385948552506

TAGTCTTCTATTGGATGCTTAG 0.353017689557

TAGTCTTTACGTCCTGGATAAT 0.445332419694

TAGTCTTTCTTTGCGGCGTAAG 0.701256823184

TAGTCTTTTGGTCATACTAATG 1.03703143428

TAGTGACCATGTGCTCTAGAAG 0.939178118826

TAGTGACGCACTGCTAATATAC 0.666442485421

TAGTGACTAGGTGTTTACGAAA 0.610513738593

TAGTGAGGGGGTCAATAGCAAA 1.46605023958

TAGTGAGTAGGTTCAACTCCAG 0.894782194319

TAGTGAGTCTCTGATTTAGATG 1.21867686538

TAGTGAGTGTTTCATCACCAGC 0.520821169208

TAGTGATGAGCTGATCAGGAAG 0.512054329182

TAGTGATGATGTGGTCCGTTTG 0.815862287371

TAGTGATTATCTCGATGGCCTG 0.77933314146

TAGTGATTGCGGCATACTTAAA 0.704053922612

TAGTGCCAATCTGAAAGTATAT 0.685209980077

TAGTGCCGAATTGTAAATGGTG 0.820978572916

TAGTGCCTAGCTTGTGCCTAAA 0.975227263559

TAGTGCTCGTAGGGATCGGCAT 1.25005839151

TAGTGGGACACTTCCGAAAAAT 0.795439144947

TAGTGGGGATATCTATACGTAC 1.0318078421

TAGTGGTAAGCTCATCAGTTAG 0.752613109935

TAGTGGTAATGTGCGAATAAAG 0.641375395789

TAGTGTAGGTAACAGGAACTAG 0.341658302024

TAGTGTCAGAGTCACAGAAAAC 0.922718674043

TAGTGTGCGGGTTAAGAAGAAT 0.412919268742

TAGTGTGTGTGACATAATGCAC 0.949502423914

TAGTGTTACCTTCCAAGACGAA 0.999260406632

TAGTGTTCAAATGATACCTAAC 0.259142216606

TAGTGTTCGGCTCCTATCAAAG 0.405145400749

TAGTGTTGCGGGGATGGATCTT 1.00548441374

TAGTGTTGGAATGCTAGTTCAG 0.634927733967

TAGTGTTTATGTCTAGAATGAT 0.618592412306

TAGTTAGAGCGTGCAAGGAGTT 0.380705757872

TAGTTATTAATCCCCTGGATAC 0.684273796212

TAGTTCTAACATTATAGTGGAG 0.559031177478

TAGTTTGCAAGGTCTGAACAAT 0.3600817419

TATAAACATCACTATTACAAAG 0.437382210696

TATAAATATCCTACGTTATGAT 0.687607099185

TATAAATTCATTTAAGCTTGTC 0.605324616637

TATAACCTACCTCTTTGTATAG 0.431907839938

TATAAGGGGTCTGTTCTACTAA 1.37601049469

TATAATCTCATTCCTTGTTGAA 0.206681181652

TATAATGATCTCCCAGTAATTA 0.192355396044

TATAATGTGAGTTAGGCACCTG 0.964055888283

TATACACCCATTGATGGTCGTC 0.922558053049

TATACACCGCGTGGTACATCAG 0.426583834238

TATACACCGTGGCGACCATATA 0.770185103477

TATACACTATCTGCATCGACTG 0.404459637677

TATACAGACAATGTCGCGGCAC 0.95951741381

TATACAGAGTCTCATTAAGTAG 0.317347298519

TATACAGCGTATTCCTCATGAG 0.489368854892

TATACAGGACCAGAATCACAAG 1.47083649567

TATACAGTAAGTTAGAATTGAG 0.331582505272

TATACATAATCTCGTGAATCAC 0.355959931875

TATACATTAGCTCTTCTACGAG 0.61758095528

TATACCCAAAGTGATTGGTAAA 0.219413622879

TATACCGAATGTGCAACATATT 0.348201985401

TATACCGATCCCTGTAAAAGAA 0.365466051257

TATACCGTGTCGCCTAGCCAAG 1.99622053446

TATACCTAAGATGCTCACATAC 0.606677486614

TATACGATCTCACTTTGACGAT 0.50169173741

TATACGCCAACTTTAAGGCTTC 0.727492758681

TATACGGAGAATGAATTCTATG 1.12279587653

TATACTATATGTCAATTGTGAG 0.275345363285

TATACTGAAATTTAGCTGCGAC 0.515986387817

TATACTGACTATCAAGTAAAAC 0.289752559619

TATAGACGCTCAGTTGACTTTT 0.4597496221

TATAGACGCTTTGAACATCCAT 0.347903401236

TATAGACTCTGGCCCGCTAGTG 1.41765393749

TATAGAGCAATTCATCAAGCTG 0.599737885488

TATAGAGGGAGTCTTTTCTAAC 0.459027673081

TATAGAGGGGCTCGTGACAAAC 1.9485960546

TATAGAGTATCGGTAAAAAAAC 0.343658651581

TATAGAGTGACTGCTGGTATTG 0.640727420551

TATAGATTACGTCTCAAGCGAG 0.22064241042

TATAGATTCTTTTAGGAGCGAT 0.711849001578

TATAGCGTAAGTGATTAAGTTA 0.269926037727

TATAGCTCCTGTGAAACTCGAC 1.2243806394

TATAGTCCTTCTCATATCTAAA 0.308412294666

TATAGTCGAGCGTCTGTGTTAG 1.2734222172

TATAGTTACTCTCATGGGATTC 0.758928708889

TATATGTTTTGTGTCGGCAATA 0.886984003089

TATATTCCGAATCTGTCTGGAC 0.511088804039

TATCAGGGCCCTGCCGAGTAAC 1.70478085475

TATCCACAGAGGCCTCTTTGAC 1.06433122691

TATCCAGAAACTTCTGTAATTG 0.215928841685

TATCCAGAAGTTGCTCTGTTAG 0.573414467173

TATCCCGCACGGGGGCTAAAAT 2.07677679495

TATCCGGTACGTCATATTAAAT 1.00218679672

TATCCTATACCTGAAACAAGTC 0.300297002524

TATCCTCACTGTCCGAGATTAA 0.801225870279

TATCCTCGTTGACAGAGTAATG 0.376752986077

TATCCTCTATCGTCTCGAAATC 0.744208894285

TATCCTCTTTCTTAAAGGCGAA 0.691405165627

TATCCTGAGTTTCCTCCGTGAC 0.503803735861

TATCCTGGCTGTCAAGGTGCAA 0.980600852519

TATCCTGTCTGTGCTTCACTAA 0.598673492691

TATCCTTAAACTGGGATTGTAG 0.546433690136

TATCGACACACTCATAGCAAAC 0.63726356605

TATCGACCATTTCCTGTGGTTA 0.302456751518

TATCGACTATCTGCCGGCTGAT 0.280383436042

TATCGAGCCTGTGGTTGTGAAG 1.31884433159

TATCGAGTATCTCAGGCAATAT 0.440717555382

TATCGCCTCTTTGGTTTCACAG 1.24822832985

TATCGTGTGTCTTCATGCCAAA 0.780146785955

TATCGTTTCATAGCTCGATTAA 0.252176842225

TATCTACACGCGCGCTCCCAAC 1.40543490943

TATGAAGGAGGTGAATGACAAA 0.748972050752

TATGCACTTTATGGCCCTGTAT 0.558942277898

TATGCAGCCGTTGAAAGGGAAT 0.949868316394

TATGCCGTCTCTCAAAATTTAG 0.382197228288

TATGCGTACTCGGGTCCTGATA 0.919690931267

TATGCTCAATTTCAACAGGGAC 0.806774747319

TATGGACACACTCCTCCTGGAG 1.08216685791

TATGGAGAAGTTCAAATTTAAA 0.200748991103

TATGGAGGATCTCTTGAGCTAT 1.29817447256

TATGGTGGATCTCGTTACCAAC 0.499945566253

TATGGTTGATGAGCTGGTACAA 0.939518515067

TATGTATAGCTTGGGTCCATAA 0.388117512639

TATTACCACTTTGCAGATCCAC 0.428223897412

TATTAGGTCCCTCTTCGTTGTA 0.588352076127

TATTATGAAATTGAGACAAGAT 0.257880881041

TATTATGTATTTGCTGGGTTAG 0.693103889029

TATTCACAGCCTGGAGTCCAAT 0.319036559186

TATTCACGCCGTCATCTATGAA 0.552171210625

TATTCAGTGGGTGATACTTATT 0.2505918081

TATTCCTCGTTTTGTCTGACTG 0.325562086918

TATTCGCTAGCTCGTAGCAAAC 0.666782995198

TATTCGTACATACCAGAGGTTG 0.652081228883

TATTCGTCCACTCAATCCCGAG 1.20850805502

TATTCTGTAACTCAACGTACAC 0.478827884837

TATTCTGTCCGTTCAATTTAAA 0.689433429127

TATTCTTAGTGTGATAGCAGAC 1.07728537531

TATTGACGATCTCCCAGATCAG 0.802814678329

TATTGAGATACTGAAAATGCAC 0.367818627797

TATTGAGGGGGTCCTTTAGAAT 0.780212309422

TATTGCAAATGTCAATTGATAG 0.544009577283

TATTGCCGATAACCTACTTAAA 0.330047728525

TATTGCTAAAGCGCAGGGGGAC 0.893293193289

TATTGGAGATCTCAGGCACCAA 0.36651504369

TATTGGGGCTCTCATAATTCAC 1.15312177875

TATTGGGTATCTGGGCTTTGAC 2.33229260245

TATTGGTAGGCTCTTTCTATAA 0.886381873859

TATTGGTATTGTCGTTACAAAG 0.323358439256

TATTGTGTCGTTCCCCACCTAC 1.02060355035

TATTGTTCATTTGCTTGTCAAC 0.516355310938

TATTGTTCGCGTTCCCGGTAAT 0.947874041281

TATTGTTGATAAGCATACGTTC 0.557162283702

TATTTATCCACTGATTATGGAG 0.514713874655

TATTTATTATTTGAAACATCAA 0.442883260911

TATTTTGTATTTCTAAAGGGAC 0.679759995568

TCAAATGTAGGTCATTGAAAAT 0.547520013717

TCAATAGATTCCCATCCCGTAA 0.420693217631

TCACAAGCATCTGTATGCCAAG 1.18338312145

TCACCAGAACCTGATACACAAA 0.77467633222

TCACGGCAAGCCCATTCATTAG 1.12829150514

TCACGTCCATCTTAACTTGCAG 1.25617886886

TCACTTCAGTGGGAATGTGAAG 1.04701597335

TCAGTATATTCTGTAATTGAAT 0.580013146641

TCAGTCTAATCTGTGGCCGAAG 0.541802609697

TCATATGAACCTCCTGCATGAG 0.281205121858

TCATCATACAGTCCTTTTCTAG 0.673323568543

TCATCATCCTCTCCGTCTTAAT 0.609482411224

TCATCATTTTGTGAATTTCTAA 0.272291190235

TCATCCGTCTCGCATAATAAAA 0.0900459887775

TCATCTCTCACTTCAGCTAATT 0.738273560669

TCATTACATGATCATTGTGAAT 0.411430288844

TCCAAACACTACCATACTGCTT 0.417899076353

TCCAAAGGAGCCGTGCCGACTA 0.793792371952

TCCAAATCTACTCCTCAACAAT 0.598125165428

TCCAACGTCACTGATAGGGTAT 0.453657278146

TCCAACGTGGCTTATATGAGAG 0.454250136114

TCCAATCTCTAAGCTATCCAAA 0.220078284595

TCCAATGCCTATGGCAGGAAAT 0.302652221279

TCCAATGGAACTTAACTCGTAT 0.448055375928

TCCAATGTACGGCATCCTTTAA 1.38060541202

TCCAATTAGTGATCTGACCTAG 0.58833474549

TCCAATTTAGCTCCTTGTATAA 0.370899623143

TCCACACAACATGGTCAATATT 0.445840858217

TCCACACAACGTGTCATACTAA 0.655077392783

TCCACACAGACTACAGCATTAG 0.560860497635

TCCACACATTCTCAATGTAAAT 0.274697087586

TCCACACTATGTTCTGACTAAC 0.549758656086

TCCACAGATTCTGCTTAGGAAT 1.00722814244

TCCACAGCATCTCCTGTGCGAC 0.308222973556

TCCACAGTCTCTGGTTAATCAA 0.422077390751

TCCACATAAGAGCCATGTTTAG 0.891613201767

TCCACATGGTCTGTTATTGAAA 0.411499069615

TCCACATGTTTTGCTTGCGTAA 0.50610825656

TCCACCGGTGCTGAAAGATTAT 0.292858461535

TCCACCGTGCCTGTTGGGTAAC 1.14226802901

TCCACCTAGCGTTTTGTTGGAG 0.856385105302

TCCACCTTAACTCTTACATGAA 0.319957549482

TCCACGCTGAGTGCTCCCTGAA 0.468034336863

TCCACGTAAATTGGTATGACTT 0.521711403313

TCCACGTCCTGTCAAGTGCAAG 0.231258689747

TCCACTCAAAGTCATAAATTTT 0.323617850467

TCCACTCAAGCTCCAAAATGTC 0.701584499153

TCCACTGATCCTCCGAGCTGAA 1.12199836292

TCCACTGCAACTCTTTACGCAA 0.528850971443

TCCACTGCCTATGATACTATTG 0.851791754472

TCCACTGTAACTGATAGTTCAT 0.338954187077

TCCACTGTAGGTCTACTTGAAG 0.657848538237

TCCACTGTATATGCTTAAGGAC 1.13454662342

TCCACTGTTGGATATCGCCTAC 2.01867997479

TCCACTGTTTGTCCAAGGTAAG 0.59262843566

TCCACTTGCTCTCTTCTGAAAG 1.34161588501

TCCAGACAAAATCGGCTCTATG 0.952256647665

TCCAGACAACGTCGGAAGAAAA 0.503057754031

TCCAGACACACTGTTGACTTAA 0.578442096677

TCCAGACTAGGGGCAGCCATAT 0.631542792752

TCCAGAGGCTTTCTTTCCCGAG 1.5871425133

TCCAGAGTACCTTTTACCTAAG 0.443429344027

TCCAGAGTTTCTCGTATTATAA 0.326717853987

TCCAGATAAACTGAAGGTTTTT 0.71372581673

TCCAGATGATCTCCTCAATGAG 0.239342677201

TCCAGCATTTTTCAACCTAATC 1.12116584177

TCCAGCCCAGGTGAGTAAGAAC 1.8135549084

TCCAGCGTACTTGCACTACTAG 0.646151467731

TCCAGCTTAGGGGAAGTCGGAG 0.250677442402

TCCAGGCGAGGTCGGTAGACTT 0.880472988729

TCCAGGGAATCTGATGCCCAAC 0.845996413366

TCCAGGGAGGCTACATCGAAAC 0.69382865611

TCCAGGGATCGTGGTAAGGAAT 0.586956462781

TCCAGGTGGATTGATGGCATTC 0.585871476713

TCCAGTCACTCTGGATACTTAT 0.96057590422

TCCAGTCATTATCCGGCTTCAA 0.785287310955

TCCAGTCTATGTCAACTGGAAC 1.5184161997

TCCAGTCTCTCTCCGTCCGCAC 0.702806882107

TCCAGTCTCTTTGTAGTCTCAA 0.127180867956

TCCAGTGAACATCCTATATAAA 0.40411548085

TCCAGTGAGCCTGTTAGATTAT 0.729865658794

TCCAGTGCGAATGCGATAGTAG 0.523919203578

TCCAGTGGTTGTCATCAGTATC 0.52029005734

TCCAGTTAGTGACCTCGGCGAA 1.09439177848

TCCAGTTGATGTCTTGCCGAAC 1.02408937152

TCCAGTTTCTCTGGTTAGGGAA 1.010932388

TCCAGTTTCTCTGTTTCGTTAT 0.652403704221

TCCATACCCTATGAATACTAAG 0.646931789268

TCCATAGAATCGGCTTGACGAC 1.86162678061

TCCATAGTGACTGCTGTGTTAG 1.21026332129

TCCATATAGGCGGATGTACTAG 0.954420806947

TCCATCATTTGTGCTGGGTAAA 0.651108722462

TCCATCCGCTGGGCTGTCCTAG 0.882669630455

TCCATCGTATCTCCTTCGTAAA 0.784444247723

TCCATGGTATATGAGATCGCAT 1.21121686681

TCCATTGGACATGTATCAGTAG 0.513948932166

TCCCAATACGCTCGGTGCAGAA 0.927017812074

TCCCAATGCCCGTATCGCTTAG 0.823563956606

TCCCATCGATCTGCGATCAGAG 1.53467817367

TCCCATTTAGTTCAAACCACAC 0.383653093324

TCCCCACGCAGTCATTGCGGAG 0.529342202218

TCCCCAGTGTGTCCTTGCTTTC 0.808626312895

TCCCCATAATATGGTGGGTCAC 0.600067426062

TCCCCCCTACCTGAAGGGGTAC 1.62171872988

TCCCCCCTATGTTAGAAGCAAG 0.237407245183

TCCCCCCTCTAGGCATGGTAAT 0.0602944124209

TCCCCCGATAGTGCTAAGACAG 0.532288040208

TCCCCCTAGCGTGCAGGCACTC 1.47858946668

TCCCCCTGACATGATTACACAG 0.969479948111

TCCCCCTTATCTCGACCCGTAT 0.779057726451

TCCCCGTAAGGTTGTCATGCAA 1.60906667669

TCCCCGTAATGTCGTAGCGGAA 0.699429213895

TCCCCGTCGTGTAGAAACACAC 0.316736916998

TCCCCTATGTGTCTAAGGAAAG 0.673443277049

TCCCCTGGCAGATGTATCATAC 0.962239931136

TCCCCTTAAAGGCATGACCAAA 0.829250068462

TCCCGACGCTACAATTGCTCAT 1.36615842099

TCCCGAGAGCTTGCCCTGTTAT 0.364761821462

TCCCGAGCGTCCGAGAGGGAAC 1.12132571147

TCCCGAGGAGTTGTATACGGAC 0.724376210821

TCCCGAGTATGTGGTGGCTGAG 1.44224576146

TCCCGAGTCGGGGAAGTGTTAT 1.63821776238

TCCCGATCGTCTCCTGCAGAAT 0.376536268227

TCCCGATTAGGTGCCCTAAAAA 0.607177951349

TCCCGCGATTATCTCTATTGAG 1.18386412388

TCCCGCGTGATTGCTGTGTGAG 0.247992202834

TCCCGCTAGTATTTTGGCAAAC 0.421533820094

TCCCGCTGGGCTTAATCAGAAA 1.22651428933

TCCCGGGGATGTGTGTTGAAAC 2.05150573246

TCCCGGTTAAGTTCTTGCATAT 1.01123176271

TCCCGTGAGCCGCATGAGTAAC 1.19185804106

TCCCGTGTGTGTGCAGAAATAT 0.952082913976

TCCCGTTCGTGTGAAGCCGCAA 1.17505356547

TCCCGTTTCACTCCAGTCGAAA 1.0283098807

TCCCGTTTCTCGGGCCGGCTAC 0.482230950015

TCCCTAGACTCTCCAACAGTAC 0.4891987869

TCCCTAGAGTGTGCATCGAATG 0.784761595253

TCCCTAGTGCATCCTAGGTTTG 0.565693471101

TCCCTATATAGTGATTCAACAC 0.517695380089

TCCCTGCCATCTTCGTTCCATG 0.986225715834

TCCGAAGGACCTCTTTCTGGAT 0.38471853144

TCCGAAGTGTGTCCATGTAAAC 0.228607770192

TCCGACGACTTTGGCGTTTTAG 1.02317841043

TCCGATTAATCTCCTTAACGTT 0.852000562327

TCCGCAACAATCGATTGGTAAG 0.839037450124

TCCGCACAGGTGTGATGTTCTG 0.843642244069

TCCGCACATACTGCTATGTCTT 0.94258957729

TCCGCACTACCTGGTCCCTGAC 0.876544867358

TCCGCAGTGACCCTGGATGAAT 1.55458652

TCCGCATGGTGGCAAGGCCGTT 1.78632675589

TCCGCCAGGTTTCCTCGGCAAG 1.06475297651

TCCGCCGAGGCATCAGCGTTAA 1.00777930847

TCCGCCTTCTGTCGGAGCGAAA 1.59019904063

TCCGCTCTGCTTCCCAGTTAAC 0.193545194545

TCCGCTGCATGTGCAACATCAG 0.368615401476

TCCGCTGTGTCTCATTAATGTA 0.774785761329

TCCGCTTATTATGCTTGAACAA 0.564243353102

TCCGGAAAACCTCTTGAGAAAT 0.275690355217

TCCGGACACTGTTATTTGGAAC 0.284532176419

TCCGGACAGTATGTTCACGGAG 0.874374828188

TCCGGACAGTGTGCTGAGAGAA 1.27936211541

TCCGGAGCAGGTGGTGACTTAC 0.747967178192

TCCGGCGACTATCAACGTAAAC 0.740566573652

TCCGGCTAATGTGCTAAGAAAA 0.222327038052

TCCGGTCGTACTGCTAACCCAT 1.67759168333

TCCGGTCTACCTGATGCTGTAT 1.00839678211

TCCGGTGAGCCACCTAATAGAG 0.476304146964

TCCGGTGCGGGTCTTCCTTAAA 0.490418852602

TCCGGTTCCAGTTAACATTCTC 0.580755576282

TCCGTATCGGTTGTTGAAGGAT 0.845395870873

TCCGTGGCCAGTGGGAATAAAC 2.2646342988

TCCGTGGCGTATCTATATAATT 0.709443877458

TCCGTTGACTCTCATAAACGAG 0.394434880978

TCCGTTGTAGCTGTTGCTATAG 0.923079919478

TCCTAAAAATTTGATGGGTTAC 1.05069200225

TCCTAACAAACGGAAATCATAG 0.469208775042

TCCTAACAAACTGCGAGCAGAC 0.379633483559

TCCTAAGGTACTGTCTGGACAG 0.758619152743

TCCTAATGACCTGATGGTAGTT 0.77990749102

TCCTAGCACAGAGAGATTCGAC 1.00566767349

TCCTAGTCTTGTGGTGGGACAT 0.63854012381

TCCTATCCATTTCTCATAGCAG 0.249311604668

TCCTATGTAGGTGAAAACTCAC 0.515114827539

TCCTATTTCGCTCCTGGGTGTT 3.03158678245

TCCTCAACGTCAATATGGGTAG 0.250414668905

TCCTCACAATCTGTTCAAGATG 0.695706481888

TCCTCACATACTGCCTGTGAAA 0.625642726648

TCCTCACTATCTGCTCTCCATG 1.27154439195

TCCTCAGAATCTCATGTCTTAC 0.162039952948

TCCTCAGACTCTCTACACACAT 0.346441561471

TCCTCAGCATCTGCAGTTTATT 1.21443866694

TCCTCAGGGGATCAACCCTGAT 0.596757713283

TCCTCATAGGGTGGACGATAAA 0.819363006993

TCCTCATAGTCCGCTAAATAAT 0.552659961812

TCCTCATCCTGTGCTGGTCAAA 0.675051706087

TCCTCATTATCTGATTTCGAAG 0.656307748206

TCCTCCCTCCGGTGACCCCCAG 1.07431856954

TCCTCCGAGACTGTCAATCAAG 0.573367605857

TCCTCCGATCCTGAAGGTCGTG 1.1340177548

TCCTCCGGCTGACATGCACCAG 1.34861920509

TCCTCCGGGGGTCAGAGCCCAA 0.700126638314

TCCTCCGTACTTGCAAATGAAC 0.217820283711

TCCTCCTTATGTGATGGTACAG 1.09091497985

TCCTCGTAGAATGTTCCAATGA 0.801811973932

TCCTCGTAGTCTGCTGAGGAAG 0.823018679476

TCCTCTAAACCTCTAATCCGAA 0.985318340198

TCCTCTATACTATCAAATTTAT 0.641743012394

TCCTCTATCTCTCCTAAAGTAC 0.84031534326

TCCTCTCCAGACGTAAGTTGAA 0.549841428437

TCCTCTCTATCTCTTGACAAAT 0.750311960981

TCCTCTGTCCTTCATTCTTAAT 0.690247164456

TCCTCTTAGTGTGATGCGGGAT 0.160771544025

TCCTCTTGCAATGCTTCGGTAG 0.682306101873

TCCTGAAATCCTGATCCATCTC 0.287833450892

TCCTGAACCGTTTAAGTAAAAT 0.495731764196

TCCTGACACGCGTCTAGTGGAC 1.69029204217

TCCTGAGACTCACATTCACAAA 0.469052015658

TCCTGAGAGCCTGCGGGGGTAG 0.600360754651

TCCTGAGCACCTCCTAATGGAA 0.671930717937

TCCTGAGCGTCCCAATGCAAAG 1.62395804423

TCCTGAGTAACTGAATAAGAAT 0.592951762336

TCCTGAGTAGCCTTATTGGTTG 0.710184193542

TCCTGAGTCTAGTCTCCTCTTA 0.165843233337

TCCTGATACCGTCAAACGTATT 0.675856346043

TCCTGATACTGCGTTTAGTGAG 0.173189654192

TCCTGATGATCTGGAGCGATTG 1.55402325057

TCCTGCGTTGATCCATATGATG 0.360329704217

TCCTGCTAGTCCGCTGCCGGAG 0.24318322267

TCCTGCTTATCGCCAGACTGTA 0.735324676901

TCCTGGACCACTGCTAGTCAAC 1.26818958141

TCCTGGATGACTCTTCATAAAT 0.509503861853

TCCTGGCAGTGTGTATCCGAAC 0.877966617203

TCCTGGCATCCATTGCGCCAAG 0.757083835643

TCCTGGCCACGTCCGGTCAAAT 1.1097320336

TCCTGGGTAACTGATGGAGAAA 1.00233855185

TCCTGGGTTTATGGTAGCGCAT 2.09010940796

TCCTGGTCCCGTGGATTCCAAG 0.704753645897

TCCTGGTTATTTCTACGTAGAG 0.439976647508

TCCTGTAAACGTCATTGAAGAT 1.54416674662

TCCTGTAGAGCTCGACGGCTAA 0.354196987328

TCCTGTCAATCTGATTCCAAAG 0.356121537389

TCCTGTCCGTCTGGTCGTCAAC 1.02677682138

TCCTGTCCTTCAGCTAAACAAG 0.582356334726

TCCTGTCTATATGATAGGCTAG 1.17205181295

TCCTGTCTTTCTGGTCGTTAAG 0.935249484615

TCCTGTGAATGTGCGTCTAAAA 0.968977705195

TCCTGTGACGTTTTAGTGAATC 0.977046744059

TCCTGTGGAACTCATTTTTCAC 0.982913669695

TCCTGTGGATCTGTTATGGGAC 1.01431244135

TCCTGTGTAGCTCCCCGCGTAG 0.629525913286

TCCTGTGTAGTGGGTCCGACAG 0.867107554088

TCCTGTGTGGATGCTAAAGAAT 0.777330800418

TCCTGTTAGCCACCAGCGTCTT 0.866495847372

TCCTTACACAATGATATCCAAC 0.316988152762

TCCTTACATTACCTTGCCTAAC 0.739298412181

TCCTTAGAAGCTGAAGAGGATG 0.57649032553

TCCTTATAGTCTCGTGCCTCTG 0.675080510567

TCCTTTCCAGCAGAAAGAAGAT 0.899785755072

TCCTTTCCATGTGCGATCGAAG 1.23060580829

TCCTTTCCGTATCAACATTTTG 0.261748509856

TCCTTTCGAGGAGGGTTACAAT 0.276040880191

TCCTTTCGATTTCCTTGTAAAT 0.508928115882

TCCTTTCTATGTCATGGTGGAC 1.24710517482

TCCTTTGCATGTCCTTAGAGAC 0.662528124901

TCCTTTGTCGCTCAATCCCAAT 1.18554227639

TCCTTTGTTGCGGAATAAGAAT 0.453420942218

TCGAAAAGCAGTCCCACCTGAC 0.494840099512

TCGAAACAATCTGTTTGGAAAC 0.331244256196

TCGAAACAGTCTGATCTTACAA 0.703624826022

TCGAAACGCAGTGATCGAAGAT 0.605214953624

TCGAAACGCCCGTGTACTAGTG 0.830920073127

TCGAAACTATGTGTCGTTAAAC 0.56261637373

TCGAAATAGTCTCATTGCTGAG 1.0219242335

TCGAAATGAAGTCCTTTCGGTG 0.329947672851

TCGAAATTATGTGTTGCTAAAG 0.644354993915

TCGAAATTGTCTTAATTATCTC 0.337113460697

TCGAACAACTGTGTTATAAGAG 0.864050322858

TCGAACCCCGGTGAAACCACAG 0.924494595986

TCGAACCTGCCCGCTGCGATTT 1.18556636861

TCGAAGTAATGTCGAGCAACAA 0.452878737942

TCGAATCACTCTGGTATGGTAT 0.645509228015

TCGAATGACTCGGATTAGGAAA 0.565679620788

TCGAATGGACGTGTTCCGTGAA 0.504346950926

TCGAATTAAAGTGCTGGAAAAA 0.746218257059

TCGAATTATTCTCTTTATTGAT 0.346881655999

TCGACACGACCTGCTAAACCAG 0.562478871019

TCGACACGGTCTCATGATCCAA 0.61149418262

TCGACAGATTCTCGTAAGTTAC 0.316219514345

TCGACAGATTCTGATTCATAAA 0.35411958933

TCGACAGCACCTGATTTATATC 0.248799975992

TCGACAGTAGCTGATTCACCTT 1.00231060442

TCGACATAGCCTTCAGAACATG 0.775145049203

TCGACATAGGGTGGTAAATCTT 0.302648200248

TCGACATGATGTGAAGTTCGAG 0.492683390586

TCGACATTACTTTAAGAGCGAA 0.622054082052

TCGACATTGTGTTATCCAAGAC 0.248134902758

TCGACCACACGTGATAGTTGAA 0.799072742767

TCGACCCCACCTCGAGTAGATA 3.40063726211

TCGACCTTCTTGCAATCAAATG 0.456314786296

TCGACGCGGGGTGCATGAAGAG 1.74965872393

TCGACGTCTTGTCTTTAAAGAG 0.592130265346

TCGACGTGCTCTGAGGCAGCAG 0.751676602569

TCGACTCAACGTCGGCAGTCAT 0.887484298523

TCGACTCAAGCTCTTGCATATC 0.514505705584

TCGACTGATTGTCCTTTATTAA 1.294518105

TCGACTGCATGTGTCTCTGGAC 0.307836832769

TCGACTGTCTCTTGAAAGAAAT 0.643968233218

TCGACTGTTAATGCACGATAAC 1.04653057372

TCGACTGTTTCTGATGTAACAA 0.444893743902

TCGAGAAGATATCGTGTTTAAT 0.738941855781

TCGAGACAACATGACGCATCTG 0.856897396616

TCGAGACAATGCGTCTAGATAA 0.483162234812

TCGAGACACGTGCCGACCGTAG 0.989067623045

TCGAGACAGACTATCCAATAAT 0.665226688842

TCGAGACTCTTTCCTGGAAATG 0.873349065315

TCGAGAGGGTGGGATCCCAGAA 0.227804064035

TCGAGAGTATTTCGAGCGAATG 0.398437123095

TCGAGATCACGTGGCTGATAAT 0.687543927316

TCGAGATTCTTTGTGATACCTC 0.440878922292

TCGAGATTTTGTCAATTGAATC 0.575049583395

TCGAGCATCCCTTTTGACAGAG 0.54170194068

TCGAGCGCAACTGAAGGGAATG 3.08460602128

TCGAGCGGGCTTGCCTGATGAG 1.44077439575

TCGAGCTTCGATCATTTGGGAT 0.922904125646

TCGAGGCCCTTTTATTAGGAAC 0.989901767449

TCGAGGGGTTTTTTATCTTAAA 1.20729406523

TCGAGGGTCTGTGACCGGGTAA 2.87380299734

TCGAGTCCCGTTGTTGGTGTAA 0.729258349064

TCGAGTCCTCCTGATTTCTTAT 0.592153457876

TCGAGTCGGGGAGCTAATGTAG 1.17098936874

TCGAGTCTTTCTCATCCTGATA 0.901244326107

TCGAGTGATGGTTACTTAGAAG 0.335302120281

TCGAGTGGATCTGATTCTTCTT 0.974195406555

TCGAGTGGTTGTGGTGGGCAAG 0.818417266906

TCGAGTGTACGGTATGAGGAAA 1.07301004168

TCGAGTGTCATTGCTAGGCTAT 0.395710393195

TCGAGTGTGGATCCTACGAAAG 0.698171355658

TCGAGTTAAGCTGATTAAGCTA 0.470544767157

TCGAGTTAATATTAGGATCGAT 0.499067398594

TCGATACTGTTTCCTGCCCAAT 0.875228927756

TCGATATAATGTTCTCGATAAC 0.539625999354

TCGATATTAGCTGCTAAACAAA 0.0889357116719

TCGATGGGAGGTGGTGTAAGAC 0.880114973242

TCGATTGTAAACGCTTGGAAAA 0.947260367813

TCGATTGTATCTCGTCTATAAG 0.881770446655

TCGATTTAAATTGCTAAGGTAT 0.28139727858

TCGCAACAGATTGCGCTGCCTC 1.88886921084

TCGCAATTACCTCCTTTAGGAC 0.653051382653

TCGCATCTTCGTGTAACGGGAG 0.747032833026

TCGCCACAAGATGTGCATCAAG 0.819186379578

TCGCCACAGGCTGATAAAATAC 0.683854840202

TCGCCACCATCTCCCACGGAAA 1.28099362978

TCGCCACCTCGTGCTGTCTAAA 1.37805529838

TCGCCATAAACTCCCAAGAAAA 0.744914031387

TCGCCATACGCTGTAGTTCTAG 0.741510244197

TCGCCATAGTTTCAAATGGGAC 0.987962754197

TCGCCATGGGTTGAAAAGTAAG 0.28907376829

TCGCCATTAGCGCTTAATTAAA 0.425774003027

TCGCCCACCCGTGATTGAATTA 0.958900635992

TCGCCCCGGAATCCTCCCCATA 1.58696976482

TCGCCCGCCGACGATGGGTATA 0.848225981025

TCGCCCGTAACTGCTGTGGGAC 0.512299849708

TCGCCCTGTCCTTGTAATAAAG 0.496829430671

TCGCCCTTCACTCAATAGAGTC 0.424431671908

TCGCCTGCATCGTATTGCTAAG 1.47079047043

TCGCCTTAAGATCATTATCCAG 0.14964108381

TCGCCTTCCGCTGATACAATAA 0.577913054107

TCGCCTTTAACTGGTTGGCGAC 0.950402663127

TCGCCTTTGGGGCCTGCGCGAC 0.77780319939

TCGCGAACTGGTGTTTAGTGAC 0.46160694103

TCGCGACGAAGTGGATTCAGAG 0.689399100048

TCGCGAGCTTCTCCCTGGGAAC 1.68257099608

TCGCGAGTCAGTCCTGAAAAAA 0.502730455723

TCGCGATCGTCACATAATTAAA 0.477183675877

TCGCGCCATCGAGCGTAGGTAG 1.16488337211

TCGCGCGCATCTTGACGATTAG 1.77432204174

TCGCGCGCCTCTGCATGATGTA 1.55773491327

TCGCGCGGTACTGGGAAGCAAG 2.10281147726

TCGCGCTAAATTGATAGAGCAC 0.468804069349

TCGCGGGCGCGAGATACCCCAG 0.550229656611

TCGCGGTAATCTCAAAGAGCAC 0.962601117193

TCGCGGTACGCTCGTCTGTTTG 1.02635441428

TCGCGTCAGTCTGCTGTAACAA 1.20356788752

TCGCGTCTCTGGCCTGGGGAAC 1.11198401497

TCGCGTCTGACTCTCCGATTAT 0.921688337526

TCGCGTGCATGCCTTAAATAAC 0.21405845363

TCGCGTGCGACTCGTTGGGAAA 2.10105151977

TCGCGTGTTTCAGGCTTGCCAC 1.33303948954

TCGCGTTGTTGCGCATCGTCAT 0.916675530326

TCGCTAGAGTCTACTGCCAAAT 0.986429996387

TCGCTCGAAAGTGCGGAGTGTG 1.07776495166

TCGCTGCGATGTTAAGGGATAT 1.50979880355

TCGCTTCCGTATGAAGCTAAAG 0.833443739003

TCGCTTGAATGATCAGAAGTAA 0.150801387492

TCGCTTGAGTGTCTACGCTGAA 0.382033338899

TCGCTTGGGTGTGATTATAAAT 0.233071466029

TCGGATGTATTGCAACATTAAT 0.689769255957

TCGGCACTACACCAACATCAAA 0.740516470793

TCGGCAGCACATCTTATATTTC 0.643864897918

TCGGCAGCATATGATGCTCAAG 1.97237701533

TCGGCAGTACGTGTGTCAGCAA 1.77056075277

TCGGCATAATGTCATATATCAG 0.652609567439

TCGGCATGCTATCATGGGATAA 0.927994288524

TCGGCCGAATGTCATTGGACAC 1.22891816156

TCGGCTCCGTCTCATTATAAAG 0.957405224365

TCGGCTGTATGTCATAGAGTTT 1.90538136369

TCGGCTGTCTCTCGTACCTGAG 1.71079037859

TCGGCTTCTTGTTAGTTCTAAC 1.58168381524

TCGGGAAACGGTGTAAGAATAC 0.461024210626

TCGGGTTGAGATGATGTTGAAT 0.559041462263

TCGGTACTAGTTGCTCCTAAAG 0.942162765694

TCGGTAGACAGCGATGGATAAC 0.622116260147

TCGTAACCCATTCAGGGCGAAT 1.70554716878

TCGTAACCGTCTCATCAATAAC 0.673115051965

TCGTAACTAGATGCTCCCTAAG 0.517265643315

TCGTAACTGTATGCCCGGTGAG 0.272653990075

TCGTAAGTATGAGATGTTGAAA 0.649113614851

TCGTAAGTCACAGAACCTAATG 0.789427151993

TCGTAAGTCCATCCTCCTCATA 0.263087434776

TCGTACCTCTCTCGTATTACAC 0.499156462206

TCGTCAAAATGTTCTGTTCAAA 0.629221607724

TCGTCAAGAGTTGCAGCACAAT 0.898081174163

TCGTCACAACCTGATCAAACAA 0.304616695714

TCGTCACAACGTCGTACGTTAC 0.885189955456

TCGTCACAATCAGGTTGCTAAT 0.43694319469

TCGTCACACCGTCCATTTGCTC 1.51796981217

TCGTCAGATTCTGCTAACGCAT 0.281154955475

TCGTCAGGCGTTGTCAATGTAA 0.955506775641

TCGTCAGGGAGTTCTCTCTTAA 0.326450239618

TCGTCAGGTCATCGTTAGTGTG 1.28910071474

TCGTCATCATAAGATTATGTAA 0.194394191213

TCGTCATGACCTGCAGCGCGAG 1.08366309141

TCGTCATTCGCTGATAGATTAG 0.965257278626

TCGTCCCTACAAGCTAAACTAT 0.316577623885

TCGTCCTAATCTGCGTCCCAAT 0.786333504175

TCGTCGCTGACTCGTGACTAAG 0.563870551896

TCGTCGGAACGCGTAGCGATAC 0.898252886181

TCGTCGGAGTGTGAAAATACTG 1.07837281529

TCGTCGGCTGGTGAACCAGCAC 0.580084599608

TCGTCGGGCTCCGGTATAGAAC 2.42769103854

TCGTCGTAGTGTCCCGAGAATT 0.668257773818

TCGTCGTGCTCTCAAGGTAATA 0.7850376963

TCGTCTCAACCTGTGCCTTGAG 0.972840099191

TCGTCTCCTTCTATTCGACCAA 0.568180996694

TCGTCTGAATGTCAAGAATCAC 0.23048692103

TCGTCTGCCTCTGAACCTCTAA 0.392782534837

TCGTCTGCGTGTGACGAGTAAC 1.04707502717

TCGTCTGTATATGCGCTACGTG 1.10802758667

TCGTCTGTCTCGGCTCCATTAT 1.4483332915

TCGTCTTCGAAACTTTGTTAAT 0.397596393012

TCGTCTTGTACCCCTTCATGAG 0.601610087584

TCGTCTTTATTTGATACGTTAA 0.0955373578976

TCGTGACAAAGTGGTGATTAAG 0.358084248686

TCGTGACATACTTGTGATGGAA 0.395522148315

TCGTGACCAGTTGCTTTTTAAC 0.897646339062

TCGTGACCCTGTCTAGAAAGAA 0.203304724518

TCGTGACGAACTCATTGAACAT 0.622234034898

TCGTGAGAAAAGTATTTCGCAC 0.758117877959

TCGTGAGAGGCAGTCCAGGGAG 1.24049366979

TCGTGAGGGTCTCAGTAGCTAC 0.375019945916

TCGTGAGGTGGGTCTTAAAGAG 0.63520900294

TCGTGATTAACTACACCGGTAG 1.35919855999

TCGTGATTCTTTGTGCGAAAAT 0.641037712315

TCGTGCGCGTCTGATTCTAGAG 1.2390431513

TCGTGCGGATATCGCGATCAAG 1.13998136368

TCGTGGGGGGATGAACGAGTAT 1.09080611044

TCGTGGTACGCTGGTGGACCAG 2.46141651183

TCGTGTACAACTGCACGAACAG 0.417370737207

TCGTGTCAAAAGCACGGTTAAG 0.689199063172

TCGTGTTTGCGTGCAACCTAAC 1.31721578156

TCGTTATTATGTCATTTTGCTG 0.617041600632

TCGTTTAGCGGACCCTCTCGTG 1.73671798334

TCGTTTCTAAGTTGTACCTCTT 0.580859369317

TCTAAACCAACGCCTCCACCAG 0.762706388521

TCTAAACGGGCTGTAATTCAAA 0.660282263401

TCTAAAGTTTTGTCAGCCACAC 0.536743880245

TCTAACCACAATCTAGTGTTAC 0.455704577438

TCTAACCTGGCTGAAGGGGGAA 0.895497254933

TCTAATCAGCCAGATCTTTAAC 0.855741190775

TCTAATCTGCATGGAAACGTAT 0.454281909686

TCTACACACTCTCGTACGGTTG 0.950832167897

TCTACACATACTCCATAACTAC 0.34430454156

TCTACACCCACTCCTTGGGGAA 1.19470186975

TCTACAGCTCCTCTAAGAGAAA 0.753588532732

TCTACATAAGATGCTGCTGATG 1.01934148771

TCTACATAATGCGACCATAGAT 0.638704773955

TCTACATATTCTGCTTCCTAAA 0.917073076854

TCTACATCAGCTGCATTCTAAG 2.57928794067

TCTACGGATTTTCCTAAGCTTG 0.691121474083

TCTACTAAGTGTGGTGGATATC 0.442675217049

TCTACTACGTCCCGTCACTTAG 0.194692983271

TCTACTGGGGCTGAAAAATTAA 0.988207021865

TCTACTTTAACACACACCCTAC 0.72926264193

TCTACTTTGCCTCGTGTTTGAC 0.786364260431

TCTAGAACGTGGCCTGGATATC 0.608295903565

TCTAGACTTTGTGTGCGGTCAT 0.958503638025

TCTAGAGACAGTGATATAAAAA 0.427057115243

TCTAGAGCGTGTGTTGCTTATT 0.877720172777

TCTAGATGGGGTGGGCCAATAA 0.156045542269

TCTAGCGACTGAGAAATGGAAC 0.657171215147

TCTAGGCTGTTTGGACGAGGTG 1.23525959611

TCTAGGGTCTTACGAACAACAC 0.522859546611

TCTAGTCACAGAGTTCTTCGAC 1.06773274896

TCTAGTCCTTGTCCTTTTGTAC 0.678817656528

TCTAGTGCAGATGCTAATAAAG 0.800514013804

TCTAGTTACGATGGTTAACTAG 0.793653774802

TCTAGTTACTGTGATTGCTAAG 0.601866069109

TCTAGTTGCACTCCAACTTCAG 0.780931469495

TCTAGTTTATTTCGAGGTTCTC 0.882452584124

TCTAGTTTGTGAGCTGCGCAAG 0.706501610303

TCTATCGGCAGTTCTAGAACAA 1.37011679343

TCTCAAGAAGTACCTAGAGAAG 0.575792620069

TCTCATGGAGCTCTTTGCAGTC 0.627033272155

TCTCATTACCATGATGATTTAC 0.26612852545

TCTCCACCCTCTGATTAAGATG 1.19304137137

TCTCCATATTCTCGGGACCTAA 0.862675969978

TCTCCATTTCGTCGAATTAAAG 0.402444240591

TCTCCCGTATCTCTGTTACGAC 0.500555206714

TCTCCCGTATGTGCTGCGGATG 1.65068820336

TCTCCGTCAGCTCAAAGGTTAA 0.576441510228

TCTCCTCAGAGTCGTGAAGGAC 0.67308388607

TCTCCTCCATCGGAGTAAATAC 0.518833487021

TCTCCTTACATTCACAGCCCTG 0.665776090592

TCTCCTTAGTGGGTTTAACTAT 0.430820539661

TCTCGAGAAACGGAAAGGAAAT 0.482952929072

TCTCGAGATTCACATAGCACAG 0.709832182038

TCTCGAGCGTCTGGTAAATATT 2.02032688118

TCTCGAGCTGTCGCAAAGAATT 0.888554402153

TCTCGATAATCTGATAGCTCAT 0.213288501116

TCTCGGCTAAGCGATTTTAATA 0.949188340753

TCTCGTTAAAATCCCCAGCAAT 0.959362762326

TCTCGTTCGTGTCTATGAGGAA 0.61521986649

TCTCGTTGCGGTGATAAAACAC 0.431420907304

TCTCTGTTAGGTCGTGATTTTG 0.909908572508

TCTGCAAACTACTTTGTCAGAT 0.544865583893

TCTGCAGAACCCGAACGGCAAC 0.955317749493

TCTGCAGAAGCACATGCTTATT 0.485111484623

TCTGCATATTGTCTACGGCCAT 0.669080644145

TCTGCCGGATGGGATTTTCGAA 1.40472918083

TCTGCGCATTGTCCTGGGTGAT 2.14545650316

TCTGCGGGGCGTGGACGCTGAA 5.23402404748

TCTGCTCTCCGGGCTGAGTGAA 0.644283212092

TCTGCTGACGGTGCTCATTATG 0.196760402836

TCTGGCCTGGGTAATGGGGAAG 0.951845397765

TCTGGGGAAACTGAAAAACAAT 0.732545783959

TCTGGGTAAGATCCTTATTTTA 0.436988938553

TCTGTACCTCCTGAGAATCAAG 0.633378923387

TCTGTACTATCTGAGTATATAT 0.507555100332

TCTTAACAACATCTTTCAGAAT 0.510810280185

TCTTATGAATCACGAATTAAAG 0.364386885029

TCTTCACCATTTGCATCTGCTC 0.945800900494

TCTTCAGAATCTGAATCAAAAA 0.40827276255

TCTTCAGAATGAGATTGGAAAG 0.589039141181

TCTTCATAACCTGCCACACTAC 0.64576402699

TCTTCATTCTTTTCTGTTCGAT 0.324408019673

TCTTCGCTACCTGGTTAATAAA 1.03528134195

TCTTCTGCATATCGGCTGCAAG 1.44286130819

TCTTCTGGCTGTGACCACAATC 0.869459228755

TCTTCTGTGCCCGGTTCGTAAG 0.355891007734

TCTTCTTATTCTGCTCCTGATA 0.613767151784

TCTTCTTCTAGTGATGGTCCAA 0.438211382857

TCTTCTTTATATGCATCGCCAC 0.581748292554

TCTTGAAATTCTTCAGGAATAT 0.763274258939

TCTTGACCCACTGAAGATGCAA 0.621715375929

TCTTGAGAACCTGATTTAGAAC 0.462392614399

TCTTGATAAAATCAGTGCAAAG 0.415819131042

TCTTGATTGTTTCGTGGTACTC 0.708829830514

TCTTGGATATGTGCACAGCCAA 1.38629362276

TCTTGGGATCTTGTTTGCGAAG 1.48131755424

TCTTGGGATTATCCATAAGAAC 0.490092702899

TCTTGTGTAGCTCCTGCTCAAG 1.26306208346

TCTTGTGTCAGTGCTAAGGCAC 0.717625342868

TCTTTTGCGTTCCCTTGTGAAG 0.992784162111

TCTTTTTTATCGGATTCACGAG 0.564632444449

TGAACAGAATGTTCTAGGGCAA 0.426094961329

TGAAGACTAAGTCGGAACAGAA 0.44425781215

TGAAGATAATATCATGACTAAT 0.327100347564

TGAAGCGTATTTGCAGAGTTAT 1.01048738801

TGAATACAGAGTGGTAAATGAA 0.499758494943

TGAATTCACTCTCGAAACCATA 0.492794234889

TGAATTTTAGGTTCTCGAGAAT 1.71499365374

TGACCGTATGCGTCAAGAACAG 0.557125541321

TGACGTTTGGTTCAGAAGTCTT 1.13762544959

TGAGCAGTCCCTCGTGTCTTAG 0.919176479283

TGAGCATAACGTGCTGCTTAAT 0.739375581632

TGAGGTACGCGGGATCTAAAAG 1.65473698735

TGATATGGGTCGGGCATCCAAC 1.11324222464

TGATCACTCGTCTATAGGGAAG 0.465587667694

TGATGACACATTGAAGGAATTA 0.291585347154

TGATTCCGGCCTGGTTAACTAT 1.19586993759

TGATTTGCGCGTGATCGAGCAC 0.820162158359

TGCAAACTAGGTCGTACAGTAA 0.577574889278

TGCAAAGAAGCTTACAAGATAA 0.590030408387

TGCAAAGCCTCTGCGCAGATAG 0.492118282504

TGCAAAGCGGATCCTATCGCAG 0.196704541986

TGCAAAGTACCTGCCTCTTTAA 0.342517796334

TGCAAATGGACTGAATATTTAG 0.70374940417

TGCAAGGTCCCTCCCTATCCAT 0.899616504365

TGCAAGTGAGCTTTAACCCAAG 0.708451365269

TGCAATATATCTCCTATAATAA 0.649867618714

TGCAATATCAGTGCCAAAGCAT 0.691639795583

TGCAATCTGACTCTTTGCGAAA 0.577493967592

TGCAATGGATCTACTTCGTAAC 0.848881458091

TGCACACAGCCTAGGTTTAAAT 0.770670986762

TGCACACAGTCTGATCCGAAAT 0.701268559726

TGCACACGGTGTTATCTAAGAC 0.463783508698

TGCACAGAAACAGTTAAGAAAG 0.510779489242

TGCACAGAACGTGATGGCTATG 0.710018551328

TGCACAGAATCGGATGGGATTT 0.492820072205

TGCACAGATAGACGTAGAATTA 0.271525188361

TGCACAGCAGGTCTTCTTTGTA 0.23299195834

TGCACAGCGGGTGATGTTCTAC 0.830163544214

TGCACAGGATGTCAACTGAAAG 1.14607186924

TGCACAGGCTCTGCATGTCTAT 1.42156889506

TGCACAGGCTGTGTCAGTAAAG 1.52925058117

TGCACAGGGTCTCAGCAGAGTA 0.678603816958

TGCACAGGTGGTGTAGTGAAAA 1.12235882775

TGCACAGTATTTGCTCGACAAG 0.471496737998

TGCACATACTCTGGACTATCAT 0.48410231556

TGCACATCCAAGCATTCCGGAG 0.652615370312

TGCACCCTCGATCTCACGTCAC 1.0705623247

TGCACCGTGTGTGTGAGCAAAA 0.422409148343

TGCACCTAAGCCCAATAATATT 1.03169803389

TGCACCTCATCTGATTGAGGAA 0.945006184609

TGCACCTTCGCCGCTCTGTTAC 0.428494225619

TGCACCTTGGATCGAATATGAC 0.74043817212

TGCACGCCGAGTGATGCTCGAA 0.336468878666

TGCACGCGCTCTCCTGTAGGAG 1.34756471159

TGCACGGTATCTCCGAGCGAAA 0.829273668635

TGCACGGTGACTCGGGATATAC 0.642414409141

TGCACGGTTTATCACGGCGATC 0.522323891117

TGCACGTCAGGTGATGAAGCAT 1.46234593072

TGCACTCAGTTTGAATGTTTAG 0.761653540159

TGCACTCGAACTGTTTACCCAG 0.530774906383

TGCACTCTAGCTCTTTAGGAAC 0.654411809029

TGCACTCTCTTTCCGACTACAA 0.848624878994

TGCACTGAAAGTCGTTGGTAAC 0.681153486406

TGCACTGACTGTGATATGAAAG 1.0977800544

TGCACTGAGGGTGCACGAGAAC 0.931052765153

TGCACTGCATCTCCACTACCAA 0.394030597017

TGCACTGCCTTTTCAAGAAGAA 0.530424587723

TGCACTGGATGTCGATGCCGAA 0.33024912479

TGCACTGGGTAGGAAAAAAAAC 0.903806368417

TGCACTGTATATGAGCACTAAT 0.359557555281

TGCACTGTGTTCCGAAGTTATC 0.629466073005

TGCACTTAACGTTCTGATAAAA 0.686743397611

TGCACTTATTCTGCTAACCAAG 0.474235828189

TGCACTTCAACACAATGGACTA 0.341245675156

TGCACTTCAATTCAACGATCTC 0.929091714635

TGCACTTCCTAACATGCGTGAG 0.676834969874

TGCACTTGATGTCATGCTGCTT 0.481109373786

TGCAGAACGACGGAGATATTAA 0.653329070275

TGCAGAATTGGTGAAAATACTT 0.478730653227

TGCAGACACTGTGGTGGCTATT 1.21704743392

TGCAGACGGACAGCTTAGTCAA 2.42625565963

TGCAGAGAAAGTGAGCTCACAT 0.746437457762

TGCAGAGAACATCAAAAATAAG 0.66090317772

TGCAGAGATGTACATACGCTAT 0.281887407704

TGCAGAGCGGCTGCAAGTAGAT 1.6105980968

TGCAGAGGTTCTGCTTCCCAAA 0.76975767014

TGCAGAGTATGTGCTATTTATT 0.595789730044

TGCAGATAATGTGAACAAGGAG 0.270636385586

TGCAGATACAATGCATCGGTAA 0.741029237217

TGCAGATCACGTCAGTTACGAG 0.455461858815

TGCAGATGCGTCGCAAGCCATG 1.61964823454

TGCAGATGTCGTTGAGGTCAAT 0.691255581968

TGCAGATTATCCAGTAACACAA 0.349273970475

TGCAGATTATGTCAATGGGCAC 0.281371967641

TGCAGCCGCCCTCGAAAGGAAA 0.768411039529

TGCAGGCAAACTCAATGGCTTA 0.510436635491

TGCAGGTCACATGACCCCGCAT 1.01246589988

TGCAGGTTAACTGCTTGGATTG 0.309445030587

TGCAGTCAAACTGCGGTTTAAG 1.24748199711

TGCAGTCAATGTATTCCCAAAC 0.449128668223

TGCAGTCAGTGTGAAAAATTAG 0.516836176104

TGCAGTGACGCGGATACGGGAA 0.797110260936

TGCAGTGGGTCTGATAACCAAG 0.795317253417

TGCAGTGTAAGTCTAAGGAAAC 0.224836606186

TGCAGTGTAGATCATGTTTCAG 1.0444451618

TGCAGTTAGGATGGTTGTCTAA 0.958860410959

TGCAGTTGAAGATAGGCGATAT 1.82278795162

TGCAGTTGATCTTTATATGAAA 0.128428106564

TGCAGTTTTAGAGGAACTTCTC 0.320346190878

TGCATACATTCTCATGTTATTA 0.395655878295

TGCATACTAGGTCACTTAGAAG 0.540663407196

TGCATAGAATATTCGATAGGAA 0.783800054103

TGCATATCCTGTCAAAGGGAAC 0.640090993498

TGCATCCCGCATGTACGCTGAT 1.4157600878

TGCATCCGTGCTGATACAGCAA 0.718224698325

TGCATCGGGTGTTCTAACTGAT 0.79885944335

TGCATGATGTGTGATGCCGTTG 0.877702598102

TGCATGGACGCAGATTATCAAT 0.453009188554

TGCATGGTTCCTGTCTCACAAG 1.09717511054

TGCATGTCGAGTCCTGTCATAC 0.192709398582

TGCATTCCATGTCATGCCCATG 1.02043511108

TGCATTGAGATTCGACGATCAG 0.896629145686

TGCATTTGGGTTGCTCACCAAA 0.204352606417

TGCATTTTTCCTCAGGATTTAG 0.458235699784

TGCCAAGCAACTCCTGCTAAAG 0.800349662124

TGCCAGGGATGTGGTAATTGAG 1.20030633781

TGCCAGTAATCTCAGCCGATAA 0.618705717022

TGCCATGCAGCTGCTGCCGAAT 3.57261999561

TGCCATTCGGCTCAACACACTC 0.273415355087

TGCCCACAAAGTCCTTTAACAA 0.709978051382

TGCCCACTGTGTCCACCTAAAG 1.74527853228

TGCCCAGAGACTGATGAATCAG 0.326450061733

TGCCCAGCACCTGCATAGTTTA 0.687766389857

TGCCCATACTGTGATGGCTGAG 1.43712546818

TGCCCATCAGGTCAATTAGTTA 0.683069563487

TGCCCATGCCATTAATGAGTAC 1.07419064693

TGCCCATTCCTACGAGTTTAAG 0.549077249517

TGCCCCCACAATGAAGGCTTTC 1.15464791271

TGCCCCGGACCTGCAGTTGGAA 1.89767642449

TGCCCGCTAGCGGGTGGGACAT 1.69110814708

TGCCCTGGATCTCAGGGGCAAG 1.1535828092

TGCCCTGTTATGGGTAGTGCAA 1.05423462339

TGCCGAATGGGTGAAGGGTAAT 0.286772579375

TGCCGACAATCTCATTCAGTAG 0.561003827173

TGCCGACAATGAGCATCGTTAA 0.414205736513

TGCCGACGACCGCCTTAATAAG 0.541125878827

TGCCGACTCTGTGACTATTTAA 0.640232823204

TGCCGAGAAGGTGTTTACGAAG 0.834021302158

TGCCGAGCATCTCGAGGAAAAA 1.92864088101

TGCCGAGCCAGTCGAGTTAATG 0.172750006337

TGCCGAGTAACTTCTAAGATAT 0.344131349565

TGCCGAGTAGCAGCTGATACTC 0.187910438625

TGCCGATAAGCTGCTGTAGATC 0.483930738985

TGCCGATGCTATGTACGATAAG 0.709239889907

TGCCGATGCTCTCCACACGCTA 0.817964452471

TGCCGATGGAGTTCTCGACTAC 1.80512431903

TGCCGATTATATCCTTGACCTG 1.03282006372

TGCCGATTGGAATACTCATGAC 1.26447998245

TGCCGCATAGCTCTCTCGGAAA 0.555773876737

TGCCGCCAAGTTTCATTTTAAA 0.917736683554

TGCCGCGTCAATGATGCACGTA 0.202159648359

TGCCGGCAAGCTCGTCGATATG 1.11479415015

TGCCGGCCCGGTCATATAACAT 0.754652316758

TGCCGGGAGTCTTAGCTGAATC 0.548800803916

TGCCGTCTATGTGCATTCATAG 1.29990016116

TGCCGTCTGTTTCAATCTTTAC 1.39561329198

TGCCGTGAGCGATTTCGGTCAA 0.722550239994

TGCCGTGAGTCTCAATGCGAAC 0.156883582952

TGCCGTTTACCTCATGGGAGAC 1.02128666401

TGCCTAGACTCTTGTGCGTAAG 0.715121300831

TGCCTAGAGTGTTCTCGGGGAG 0.934108685845

TGCCTATGATATCACCCGAATA 0.813147710029

TGCCTATTCAGTGATCAGCAAC 1.05421468567

TGCCTCGACTCTGATGCGTCTC 1.15744231983

TGCCTTTACAGTGGATGGAAAC 0.474676690748

TGCCTTTCAAATGAATTGGTAA 0.658186122092

TGCCTTTTAACTTATGCCGGTT 0.672919463122

TGCCTTTTTTGTCGTCCTAAAT 0.45052724317

TGCGAATAGTCTGATAGTGAAT 0.243994166811

TGCGAATGGTCTGCTAGGTTAC 0.661496414002

TGCGAATTCACAGATTATTATG 0.230204302966

TGCGATCTATCTGCACCGCCAA 1.25553976275

TGCGATTAGTCTGATCGAGGAT 0.826762629065

TGCGATTATAGTCACGATTAAC 0.475642078403

TGCGATTGGTTAGATCCTCAAT 0.480059522532

TGCGCACAGTGTGCTAGAACAT 0.640355267876

TGCGCACTACCTGTTCGCGAAA 0.829640479341

TGCGCACTATGTCAGAGAGTTA 0.949762068327

TGCGCAGTGTGTCAGCATTTTT 0.681726979152

TGCGCATAAACTGCCTGTAAAC 0.296613690942

TGCGCATAAGTGGAAATGTAAG 0.1876602179

TGCGCATAGTATCCTCTCTGAG 0.664473072143

TGCGCCGAGTCGGGTGCCTCAG 4.9953479194

TGCGCCGTCACTGATAGGCGAC 0.726522909418

TGCGCGAGATGTTAGAAGGGAC 0.994322817076

TGCGCGTATGCTGCGCCGAATG 1.6634623401

TGCGCTCCGTGTGGACAGTAAC 1.04424241498

TGCGCTGTCGCTGACGGTTGAT 0.747699695113

TGCGGAACACATCTTAGATATT 1.11208050501

TGCGGACCATCTGATGCTCAAG 0.41035760554

TGCGGAGAGTTTGAACCCCAAT 2.11686712453

TGCGGAGGACCTGGACAGAAAT 0.991305855951

TGCGGAGTATGTCATCATCAAG 1.54031929137

TGCGGAGTCTGTCGAGTTCGAT 1.487047848

TGCGGATCAGGCCATAAACAAG 1.0833145962

TGCGGCACGTCTCTTTATTAAA 0.55776596647

TGCGGCGAGTCAGAGGCACCAA 1.7771307778

TGCGGCTCGTATCAATACACAA 3.24797876519

TGCGGCTTGTGTGGTCGCGTAC 2.81347107125

TGCGGGTATTATGGTGTCAAAT 0.919566342251

TGCGGTATACTTCGATGATCAT 0.683543853007

TGCGGTCACTATGGTAGACGTG 0.93628615077

TGCGGTCCATGATATACTATAC 1.88823619121

TGCGGTGAAAGTCGAGATCCAG 0.709299707125

TGCGGTGAACGTCCAAGCGAAA 1.21021817341

TGCGGTGAAGATGTGAACTAAT 0.408597783549

TGCGGTGGCGCTGCATATAGAA 1.71359596971

TGCGGTTTACATGATGACCATC 0.619826200871

TGCGGTTTCAGTCCTGTTACAC 0.566668002733

TGCGTCCAATCTTCACGTCCAT 0.652588827768

TGCGTCTTGTGTGAGGCGCAAC 1.66108364042

TGCGTGGTATCACATTAGTAAG 0.695605675176

TGCGTTGAGTCACACAGTTGAG 1.00800525975

TGCTAACAAGGGCACAGGCAAA 1.27418214509

TGCTAACCATCTATGTGCGTAC 0.214937456904

TGCTAACGGACTGCACAATCTG 0.167142005245

TGCTAAGATCCTGGGTCATAAA 0.907711196571

TGCTAAGTCCGTTGTCCATCAC 0.810165226558

TGCTATGACTGTGGTGCTGAAG 0.96553761036

TGCTATGGCCGAGGTATTGGAT 1.25593132471

TGCTATTAGTGTGGAGACTATT 0.557247792379

TGCTATTGTGGTCATCTCTGAC 0.471835888916

TGCTCAAAGAGTCATTCCCCAA 0.458588931138

TGCTCACAATATCAAAAACCAC 0.599202062087

TGCTCACAGATTGCGGCATTTA 0.63735442366

TGCTCACCTTGGCAGATCGATA 0.973369159484

TGCTCACGGTCTGATTTATTTC 0.702073766608

TGCTCACTAGTTCAGCACAATA 0.495207494815

TGCTCACTGTCTCAAGGGTATC 1.10266430383

TGCTCACTTTCGCTTCTTAAAG 0.818577084262

TGCTCAGAATGTGCTAGATTAC 1.15814611945

TGCTCAGATTGTGAAGAGAAAC 0.794089158003

TGCTCAGCGTCTGATGTCAGAG 0.577603023463

TGCTCAGGGAATGGAGCCTCAC 0.793493196524

TGCTCAGTACGTGCGGCTATAC 0.275925136347

TGCTCATAAACTGCTTTCGAAG 0.670371934385

TGCTCATACTATGATCGTAGAG 0.340590353081

TGCTCATAGTCTCCTCTGTTAC 0.206408946643

TGCTCATCATTTGCTTCTCGAC 0.382833054082

TGCTCATGGTGTTTTCGAGTAT 0.78201809811

TGCTCCGCCCCTCGAAATAATC 1.0459283331

TGCTCCGCCGGAGCATACAAAG 0.211687386168

TGCTCCGTACCCCACGAAGAAT 1.23555954246

TGCTCCTAAGGTGGGGAGCGAG 1.1138317209

TGCTCCTGACGTCTTGCACAAC 1.25842309081

TGCTCCTTACATGCTACTTAAG 0.402994047761

TGCTCCTTATCTTAGGAGAAAC 0.272757281263

TGCTCGGCAAGTCGTATCTAAT 0.657402864909

TGCTCGGGTAATGTGTCTATTA 1.13491065306

TGCTCGTTACGTGATTCACCAA 0.135225431385

TGCTCGTTATCGCGATGCCAAC 1.33382532565

TGCTCTCAAACGCATCTGAATA 0.513849870485

TGCTCTCACTCTGCTGTGCAAT 0.769379277593

TGCTCTCATTCACTTACGGTAC 0.889753626205

TGCTCTCGGTCTCGTTTAAAAA 0.821607168103

TGCTCTGTATCTCATGTTCAAT 0.33099440141

TGCTCTGTATTTGATAAACAAG 0.676501217488

TGCTCTGTTTTTCTAGACCAAC 0.401767191247

TGCTCTTATCATGAACGTAAAA 1.05916486003

TGCTGACAAGTCCTAGGATCAT 0.910500464501

TGCTGACAATATCATTACGTTG 0.413703352023

TGCTGACCCTGTCATCCTAGAG 1.00630643344

TGCTGACGACATGTGGGCGGAA 1.45100591512

TGCTGACGTTATCAACACATAG 0.274847643445

TGCTGAGAACGTGCATTCGATG 0.274079374277

TGCTGAGGCACTGCAAAGCTAG 1.10867488092

TGCTGAGTATGTGCTAGCTTAG 0.9752867224

TGCTGAGTGACTAGTGTAATAG 0.14698246726

TGCTGATAATGTCGAATCATAC 0.425590359573

TGCTGATACTCTCCTTAATGAT 0.344719716126

TGCTGATAGACGCGGCATATAT 0.750844523809

TGCTGATCGGAGCGTCACCAAT 0.589603168815

TGCTGATGGAGATTAGCGTTAG 1.0517034385

TGCTGATGGTGTCGCGATTAAC 1.29917791854

TGCTGATTATAAGTTATTAGAC 0.659903759839

TGCTGCCACAGTCCTTTTCAAG 0.379862395209

TGCTGCCTACCTGGGATTGTAC 1.33333908281

TGCTGCGTCTGTCAACTTAATA 0.602410597012

TGCTGCTACTCTGAAATGGGAC 0.528763359286

TGCTGCTCAGATTATTACCTAC 0.802723305994

TGCTGGATTACTCTTCGTTTAT 0.286492073723

TGCTGGCACTGGGGTGTGTGAT 1.21797539276

TGCTGGCTCCCGGCTACGTGAC 1.54339947707

TGCTGTCAATGGGCAACTCGAC 0.725328046474

TGCTGTCAATGTTCATCTTAAA 0.976263681974

TGCTGTCCATTCGTGTAGTTTA 1.47631039618

TGCTGTGGAGGCGCATGAAGAC 0.725234968867

TGCTGTGTCCCTCCAAAAATAC 1.01034410152

TGCTGTTAATCTTTACCAACAC 0.722004881673

TGCTGTTCACCTGTATAACATT 0.65829539947

TGCTGTTCTACTTTATTGGTTT 0.4247175315

TGCTGTTGATCTCCCGGCCGAA 3.54865220966

TGCTGTTTCGCTCAAATTACAA 0.744892836825

TGCTGTTTGCGTGCTTTGGGTT 0.7879973016

TGCTTTCGAAGTGTTAGTATTA 0.803043425156

TGCTTTGTGGACCTATATGAAA 0.645782544073

TGGAAACTCAGTGCTCGGTAAC 1.57368518754

TGGAAACTGTGAGCTTCCCAAG 0.896839308401

TGGAAATTGTGTGGTCCTCAAA 0.475459288019

TGGAACGGGAGTCTGTACCAAG 0.676412743108

TGGAACTTGATTCTGTGTGCAA 0.252851633882

TGGAATAACTATGTAATCACTC 0.142780893701

TGGAATGAAGGTGCTTTTAATT 0.666675144452

TGGAATGCCTCTCATGCCGTTT 0.722293600047

TGGAATGCTTGAGGAGCCAAAG 0.891268055176

TGGAATGGATTACCCTAAGCAA 0.787229376852

TGGACACACCATGCTGCTGATG 1.96934761948

TGGACACGGATTGCTTTTGGTA 0.962754010499

TGGACACTGGTCGCTTAAAGAA 0.409564885781

TGGACACTGTGTCATCAGCAAC 0.520910369607

TGGACAGAATGTGCTTCATAAA 1.05961559911

TGGACAGAGCAAGACGTCTAAT 0.651724780569

TGGACAGAGGAGGTTAAAGGAT 0.703400532202

TGGACAGATTGTGCTTTGATAT 0.670306009482

TGGACAGGATCTCAAACCTCAA 0.488577890975

TGGACATAATCTCCCGAAGAAT 0.377088575244

TGGACATACAGGGCAAGAGTAG 1.16538296114

TGGACATATACTGCTACGAGAG 0.274163402592

TGGACATATGTTTGTGAGGCTC 0.677025100204

TGGACATCCTTTGTTACGGAAT 0.613969967553

TGGACATGATATTTTGTACAAA 0.566332233059

TGGACATTACTTGATCATTTAG 0.555605231938

TGGACATTCTCTCAAGACAGTT 0.303649816823

TGGACATTGTATCGTTACTAAA 0.790033540022

TGGACCCGAGACCCTTCGGTAT 1.42322266096

TGGACCGAACGTGCTGGCATAA 1.35030299871

TGGACGGAGTATGATCGGTAAT 1.10745675249

TGGACGGGAAATCAAGCTTAAG 0.255446386526

TGGACGTCATCTCAGGCATTTA 1.49804848915

TGGACTATAAGTCTTATATGAC 0.280693711101

TGGACTCAAGGGTAAAGAGTTC 0.730903518828

TGGACTCAATGTCAAGTTGTAT 0.301927671043

TGGACTCAATGTTTAGTCCTTT 0.343287196864

TGGACTCATCTTCATAAATGAC 0.271023027755

TGGACTCCATCTGCTCTACAAA 0.782267127746

TGGACTCCCGGGGCGCGTCAAG 0.532389751816

TGGACTCTAGCTGATTCCTGTG 1.48302296154

TGGACTCTATTTGACTAATGAA 0.834638897665

TGGACTGAAAATCTACGAAAAA 0.407374651354

TGGACTGAGTATGCTTCGGATG 0.101254921193

TGGACTGAGTGACTCCGAAAAT 0.874869417656

TGGACTTTAAGCACTAAGCAAC 0.310603083093

TGGACTTTACATCGTTCTGCAA 0.504698920797

TGGACTTTAGATGTGATTCCAT 0.987026052345

TGGACTTTTTCTGAAAGAGGAG 1.06031929547

TGGAGACGCGTTCATACGGTAC 0.516316662264

TGGAGACTACGCGATAATTTAA 0.447430529362

TGGAGACTTGGTGGACTAAATA 0.50093343637

TGGAGAGAAGGTCACGAGGGAT 0.574392226623

TGGAGAGAATCTGCTACTAATT 0.452939003765

TGGAGAGACACTGATTAACTAA 0.625646182741

TGGAGAGACCGTCCTACAACTC 0.47833219601

TGGAGAGATCGGCATTTTAATC 0.640372493084

TGGAGAGTAGGTGCGGACTTAG 1.47849460757

TGGAGAGTATGTCCAAACGAAG 1.10163585488

TGGAGATAATATCCAGAATTAC 0.548480622872

TGGAGATATCCTGTAGTGGTTT 1.11509251634

TGGAGATCAGGTGCAATGAGAG 0.604837989044

TGGAGATCTTGTGGTAAGTCAA 0.268114049709

TGGAGATGAGGTTATACCTAAG 0.402188954298

TGGAGATTTCATCATGCAGATC 0.645787935492

TGGAGCCGGTGTGTTCGTCGAC 1.54069638783

TGGAGCCTCTCTGATTAGTAAT 0.797927237234

TGGAGCCTGAATGCTAACGCAC 0.983827986121

TGGAGCGAATATCCTAAACTTG 0.309817338722

TGGAGCTATTGTCAAACCGGAA 0.37444989218

TGGAGCTCAAGAGTTATCAGAG 0.471376471119

TGGAGGGATGGTGCGCCAAGAA 1.52012862693

TGGAGGTCATGAGCTTAGTTAG 0.357754820118

TGGAGTAGGCCCACTATTTCTA 0.734752080751

TGGAGTCCGAACGATAATTGAT 0.546245624987

TGGAGTCTGAGTGAAGGCAAAG 0.319113641711

TGGAGTGGACGTGTTGTGAAAT 0.577706241417

TGGAGTGTCGCTAGGAGGGGAG 0.219469525562

TGGAGTTAAGCAGTTAAGCCAT 0.143463949599

TGGAGTTATGCTGCCAATGCAT 0.372770305192

TGGAGTTCGTCTAGAAGAGCAT 0.65458766783

TGGAGTTCTCATCCACTCTAAT 0.827684011333

TGGAGTTGCACTCTTTACTGAT 0.692669675254

TGGAGTTGGCCTCCTGGCTGAG 1.18573782909

TGGATAGTCTGTCATGGGAAAT 0.791990284074

TGGATCCAATTTAGGTACACTC 1.04077638937

TGGATCGAATGTGATAACGGTA 0.838496725041

TGGATCGTCAGTGGTTCTGTAC 1.48061065204

TGGATTCAAGCTGCTGATCAAT 0.543439602762

TGGATTGACGGCGCATAGTTTA 0.487088227791

TGGATTTACTTTGATGCACGAG 0.442387909913

TGGCAAGACTGTGCTGCGGCAA 0.987504130931

TGGCAATAATGTGATAGTAAAT 0.530712172737

TGGCAATGCTGTCCTGGTCTAC 0.856982589801

TGGCATCCGCGTTATTTGTATG 1.02414732495

TGGCATCGTTGTCATTGAGCAC 0.588407989672

TGGCATGTATAGCGACGTATAT 0.821572944347

TGGCCAGATTCTCCGGGTGTAG 1.71021270332

TGGCCAGCCGATCGTTGAGAAA 1.21578953648

TGGCCAGTCAATTATAGGTTAG 0.403748455214

TGGCCATAATCTCACGACGCAA 0.521747772464

TGGCCATCATCTGCATTTCCAG 1.13847309316

TGGCCATCCTCTCTTACCTGAC 0.868930784362

TGGCCATTATGTCGTAGTGGTA 1.07176725961

TGGCCATTGGGAGTACATGATG 0.288290899685

TGGCCCCAACGTGTAAACTCAC 0.948679679646

TGGCCCGATACTCACAAAGATG 0.649514655324

TGGCCGCACGATGTAAGATTAC 0.583516638989

TGGCCGCGGTTTCAAAGTAAAG 0.704832816439

TGGCCGCTTCTTCTTAAATTAA 0.59558791999

TGGCCTGAATGTGCAGAGGGAC 1.17335963779

TGGCCTGCGCCGCTTTTATAAA 0.903329183257

TGGCCTTGGACTGAACCCATAA 1.0924440315

TGGCGACACGCTGCTCAGCTAT 1.61978180914

TGGCGACCATTTCTTGTTAAAC 0.931283513139

TGGCGACGCCGGCCTACTCTTG 1.64824979926

TGGCGACTGTGTGTTGGATTAC 1.22829970561

TGGCGAGACTGTCCATGATTAT 1.60647775258

TGGCGAGCGATTAATAGGAAAT 0.859858689188

TGGCGAGTGTCTCCAGGCAATG 1.42072458401

TGGCGATACCGCGTTGGTTTAT 0.692042215231

TGGCGATCGCATGATACAATAA 0.685843665578

TGGCGCTACTCTGAATCATTAG 1.0700786145

TGGCGCTGAAGTCCGCGAAATC 1.21242876758

TGGCGGCGGTGTCATACAGATG 1.4077637204

TGGCGGTTATATGCGATGAATT 0.992415477285

TGGCGTCAGTCTTATTAGTCAT 0.779765734679

TGGCGTGGGACTGCTTGGGGAG 2.99164496046

TGGCGTGTGGCGGTACGATATG 1.16706184828

TGGCGTGTGTTTGTCCGCATAG 1.87698516728

TGGCGTTACTGTGGTACATAAC 1.00145519328

TGGCGTTGACCTGATGCCTCAG 0.572541373906

TGGCGTTGATGGGCTGGCAGAG 2.6086933215

TGGCTATGACCGGTACTGTAAA 0.775310848078

TGGCTATTGTGTTGATCAAGAT 0.937776927198

TGGCTCTTCGGTCGATCATGAG 1.08215891035

TGGGAATAGACTGCATAAGGAG 0.670585883478

TGGGATGCATCTGCATTGAGAT 1.04410173175

TGGGATGTAACTTATGCCTAAA 0.557124024009

TGGGATTTAGGTGTATGCTAAT 0.256664486474

TGGGCAATGTCAGTTCACGGAA 1.02846545375

TGGGCACAATTTGTTACATCTC 0.750972068513

TGGGCACATTGTCTAGGCGTAT 1.42578241914

TGGGCACCCTCTGTATGAAGAT 0.989009272233

TGGGCAGACTTTACTAAGGATA 0.49332170335

TGGGCAGTACCTCAACACACTA 0.61093617001

TGGGCAGTGTCTGGACGATGTC 1.67147088472

TGGGCGCACTTTGGTAGTCATA 1.12644583853

TGGGCTCTAGGGGCTGGGTAAG 1.03153855436

TGGGCTGAATGAGTATGTGTAA 0.98011022874

TGGGCTGAGGTTCAGTGGTAAT 1.05758101544

TGGGCTTCGTTTGGTTTGCGAG 0.594531947566

TGGGCTTGATGCGATAGAACAA 1.0532991199

TGGGCTTTGGTGTGTGGTTTAG 1.24246486676

TGGGGAGGAGGACCTTGAATAT 1.352275192

TGGGGATAATGTGGTTGAACAA 0.92675415114

TGGGGATGGTGTAAAATTAGAG 0.518316423809

TGGGGCTCAGCTCTTGGGGATA 1.96616935773

TGGGGGGGCAGTGAAAAATCAT 2.27421939421

TGGGGGGTACGTCACGATGAAT 1.35991675514

TGGGGTCATACCCCCGACATAC 2.6359248458

TGGGGTCCATCTCCGCATAGAA 1.13133316656

TGGGGTGAGCGAGTGTCGAAAA 1.17310901778

TGGGGTGGATGTCATTCATCAA 0.515255322199

TGGGGTGTCTGACGGCGTCGAC 3.46570967031

TGGGTACAGTGTCAACCTGAAC 1.14249079901

TGGGTAGAATATCCAAAATGAT 0.524595801082

TGGGTAGGACGTGAAATGCAAA 0.99503041438

TGGGTATGATAGTATGGCTATT 0.550420260526

TGGGTGCCCTCTTCAAAGCGAA 1.4517329711

TGGGTTTTTTGCGCTAATAAAC 0.36225255861

TGGTAACTCTCTGGGGCGCTAT 0.936116028738

TGGTAATACTCTGGCAAAGAAT 0.43804615429

TGGTACGAAGCTCATTAGTAAG 1.47175252714

TGGTACGCGTGCGTTTCCCTAT 0.965934779638

TGGTATGTAAATAGAAAGCCAT 0.53006848614

TGGTATTACGGTGATCGTGCAG 0.4138453843

TGGTCACACTTTTCTAGCAGTT 0.484088084242

TGGTCACCCGCTCATCGACTTC 0.942385382752

TGGTCACCTCGTGTTCAAGTTC 0.484111704122

TGGTCACGAACGCTGTTCTTAG 0.545649582603

TGGTCACGCGTTGAAAATGTAT 0.615500251971

TGGTCACTATGTTCTTTCTGAA 0.374793754723

TGGTCAGAACTTCGTCAATTAA 0.471313103952

TGGTCAGCCGTTCCATACTTAG 0.519606075564

TGGTCAGCCTATCTTTCCAAAG 0.659198211742

TGGTCAGCGTCTGGACTCCGTA 1.04394888709

TGGTCAGTACCTGATTAATCAG 1.12348601441

TGGTCAGTAGTTCAACGCGAAC 0.893925152929

TGGTCATAATCTTTTAGTAAAG 0.61796709598

TGGTCATACCTTGTTGTCGATG 0.904332378346

TGGTCATCTTCTGATTACTAAC 0.312871055029

TGGTCATTAAGTGCTTAAAAAT 0.388845036037

TGGTCATTCGATGCACCTCTAT 0.64394528148

TGGTCATTGTGTCAATTTTCAT 0.432525724148

TGGTCATTGTGTGCTCATGTAT 0.117594896959

TGGTCCGTAATTCCTGTGGTAA 0.675970182865

TGGTCCGTACGTGTCGAATTAA 0.828137172249

TGGTCCGTATGCGATCTTAGAT 0.934690174514

TGGTCCTAACCGGAACATGAAA 0.587428183186

TGGTCGCTGTCTGTAGTTGAAG 0.236587110219

TGGTCTCGACATCCTAGATATG 0.607911029421

TGGTCTCGTAGGGTTAGAGTAG 0.352367772987

TGGTCTGACTGTTAGGATACAC 0.686055049299

TGGTCTGAGCGAGACGTAAGAT 1.34320370714

TGGTCTGAGTTGGAGCTACTAC 1.33056035264

TGGTCTGGAAATGGTAAAATAA 0.854395533641

TGGTCTGGAGATGAGTTTCATA 0.57130219921

TGGTCTGGCTATGCTGAGGTTG 1.54861552675

TGGTCTGTATCTGCGGCGGATA 1.20276287155

TGGTCTGTATGTGCTCGGCAAT 1.4829893292

TGGTCTGTCAATGATAGTCCAG 0.541608697897

TGGTCTGTGGGACCTACACCAG 1.6080670578

TGGTCTTAATCTCCTTTAGCTA 0.999069553818

TGGTCTTTACCACGGGTCAAAC 1.38210934646

TGGTCTTTGTATGAAAACACTA 0.379148242803

TGGTGACGAGCTGTTAGTTAAG 0.517200057466

TGGTGACTAAATCTCCATCAAA 0.169133893071

TGGTGAGAAACTTCAGTCCAAG 0.513215242902

TGGTGAGAATCCCCGAAAGAAA 0.36140475223

TGGTGAGTCAGTGGTACTTTAC 0.229459032243

TGGTGAGTGTCTCGATGGGGAT 0.821360481549

TGGTGATACACTCGTGGGTTAC 0.57947366202

TGGTGCCTAAATCACTACACAG 0.11401380365

TGGTGCGACAGTGCTCGATTTA 0.210781858226

TGGTGCGGATCTGCTGTTTTAG 1.60743872864

TGGTGCGGTGCTACGGACCGAT 1.22332336125

TGGTGCTTAAGTGTTTAGTTAA 0.670400813508

TGGTGGCAATGTGTGGATAAAT 0.978878844265

TGGTGGGTACGATTTTCAAGAT 0.790208845937

TGGTGGGTCCGTGCAAAGGAAG 0.584624774219

TGGTGTACAAATGGTTGCTATA 0.789125832538

TGGTGTAGCTGTGGACTCAAAG 0.910092693425

TGGTGTGAGTATCGTTACGCAT 1.32343058486

TGGTGTGATCACTTTTAGTAAA 0.348430905416

TGGTGTTTATGTCCATTTAATC 0.75840933136

TGGTGTTTGTATGCAGGATCAT 0.967453680991

TGGTTACGTGATGGTATCACAG 0.883199482767

TGGTTATAGGGTGCTGTTTGAA 1.42978473089

TGGTTCCTATGTGCACACGGAA 2.15532735447

TGGTTTCAAAGTCAAACTGGAG 0.640514168909

TGTAAATAGTCTGAATTGCGAT 0.563741591735

TGTAAATGGAGCTCCTTAGATA 0.490483334648

TGTAATGGACTCCTTCCAGGTG 0.621183326673

TGTAATTGCCAGGCTCAAAAAA 0.514819581616

TGTAATTTCTGTCCTTCTGATA 0.622706811227

TGTACACAATCTGTCGTAAAAG 0.476629218165

TGTACACACTCTGTTGATAAAG 0.602172918994

TGTACACAGTAGTCACAGTCAC 0.885502902758

TGTACACCATCAGCTACGAAAA 0.432890934057

TGTACAGAATCTCGTTGAGATA 0.504983447696

TGTACAGATGGTGATCCGAAAT 0.499087822468

TGTACAGCAATTGTCGTTTAAC 0.472053580552

TGTACAGCGTATGATCCCGAAA 0.52019552867

TGTACATCTACTCATTCAAAAG 0.317412868144

TGTACCCTATCACTAAATAAAT 0.341313222737

TGTACCGAAAGTGCTAGGAGAG 0.599650297918

TGTACCTAAGTTGGGTGATATT 1.04925741394

TGTACCTATTTTTAAAGCCAAA 0.281894003132

TGTACCTTAAGTGTATTACTTC 0.756554419657

TGTACTCGCTGTTTATCGACAA 0.861971942924

TGTACTCTGTGTCCAGCGACAT 1.04981937787

TGTACTGTGAGTGATGAAGTAC 0.91077836325

TGTACTGTGTCTGTTGAGAAAC 1.09510986119

TGTACTTGGTGTGATTGCGTAA 1.16485506674

TGTAGAAGGTTTCCTCCAGTAC 0.829734501972

TGTAGACGCCTTGGTAGGTTAC 1.09600758391

TGTAGAGAAACTGTGCCCTGTT 0.64431372407

TGTAGAGACAGTGCGGGAGAAT 0.626268664441

TGTAGAGGGGATGCTTCCTAAT 0.282988900455

TGTAGATCACGTGGTAACCGTT 0.527582497566

TGTAGATTACCTGAATATCTAT 0.492079423564

TGTAGATTGTGTCAAGGATAAG 0.608790446252

TGTAGCCAAAGGCATGAACAAC 0.707218531013

TGTAGCGAGGGTGTTTGTTAAT 0.537025616563

TGTAGCTACTGTGATAAGAGAT 0.871184779446

TGTAGGGGGAGACAGGAGAATG 0.4289872831

TGTAGGTAGCCTGCTAGATTTA 1.09285150333

TGTAGTCTAGCTCAGAAAGAAC 0.581451414853

TGTAGTGAATGGGCTAAGTATG 0.834178958398

TGTAGTGACTGTCATCGTTCTA 0.44139244389

TGTAGTGTATACGATTATGGTC 0.985728163462

TGTAGTGTATCATTTGGAGCAC 0.994206194089

TGTAGTTAGGATCATCAGCCAA 0.986360728322

TGTAGTTGCCCTGCAGGGTGTA 0.629703331308

TGTAGTTTGAATGATTTGAGTG 0.943169747796

TGTAGTTTGTATGCTGATTTAG 0.576906782362

TGTATACAGCGTGCATTCTAAT 0.547233186918

TGTATACCCCCTGTGTAGTCAT 0.552466219835

TGTATCGAGTCTCCTGATTTAA 0.623425163449

TGTATTCTATCGGCTTATTTAG 1.10830422102

TGTCAGGCCTATGATAAAAGAA 0.89327161247

TGTCCAGTGATCGCTCGCCGAT 0.981455077562

TGTCCAGTGCCTGATGTCGCTC 1.01332653563

TGTCCATGGGTTCATGTTAGAT 0.610897176034

TGTCCGGGCTCGCCTAATGAAG 0.777904135508

TGTCCGTCACGTTCACTGTTAC 0.726961922244

TGTCCTCAGTGTCTTGGCGAAA 0.717378548957

TGTCCTCTAATTCTAAAGTTAT 0.29104399495

TGTCCTTAGACTTATGGTCAAT 0.670063715037

TGTCCTTTACATTTGAGACTTT 0.458214644144

TGTCCTTTATGTGAGGGACTAC 0.747500082718

TGTCGAAACTCGGAATCTTAAT 0.727878963134

TGTCGAGGTGCATAGTCCGAAG 1.23457514199

TGTCGAGTAAATCTTATGAAAT 0.2780261494

TGTCGATATTATCCAGCCTTAT 0.701546168957

TGTCGCCAACTTCTATCACATG 0.653952878407

TGTCGCGGTTGCCGTGGGTGAG 0.803774774473

TGTCGTCAGAATGCTCACGAAA 0.60544520598

TGTCGTCCCCCAGTTTTATAAG 0.803807001971

TGTCGTTCGTGTGTACGCCAAC 1.12196451841

TGTCTATCATGTTTTCACATAG 0.715192726384

TGTGAGGGCATTCGCTCAATAT 0.704024781409

TGTGCACAGACTCCAGCAAGAA 2.00858333316

TGTGCAGACCCTGCTCTGGTAT 1.92917420571

TGTGCAGTCTCTTCGCGTTCAC 1.21837279103

TGTGCAGTGGCTGCGGGTGAAA 0.576238807676

TGTGGACTTTCTGAATCACATC 0.706477638312

TGTGGTCCATGGCCTTCGAAAG 0.835770805724

TGTGGTGGATCTGTCGGATCAT 0.463527687301

TGTGGTTTATTTGCTAACTAAA 0.695805540435

TGTGTTGTGGCAGTCCTTTAAG 0.605467178842

TGTTAACGCCGTGTTTGCTGAG 1.00680439419

TGTTAACTATCTCTTATGGTAA 0.502475428541

TGTTAGGGGGGTTACGGCTTAC 0.551626834417

TGTTATCTTACTGTAATCTTAT 0.473340440849

TGTTATGAATGTGCTTGCCGAG 0.713272521345

TGTTATGGTGGTGATAACTAAG 0.525992110691

TGTTCAAGACGTCTTTATCCTA 1.06462790648

TGTTCAGTGTGTGATCGGTTAG 0.518815307063

TGTTCATAATCTGCCTAAGGTA 1.1173543848

TGTTCATGTCCCGCAGACTTTT 0.49319001141

TGTTCCGGACGTGTTGCTTAAA 1.6956726609

TGTTCGGCCGCTGCGAGTGAAC 1.18992708047

TGTTCTAAAGATTATGCTATTC 0.364318300809

TGTTCTCAAAATCCGTGTTGAG 0.459688553944

TGTTCTGAAAGTTCTGAATCAG 0.380400583224

TGTTCTGGAACTGGACCTATAG 1.17906269391

TGTTCTTTCAATCTATTGCAAG 0.23820207275

TGTTCTTTGGGTGATACGTGAT 0.477721396944

TGTTGAGGGCCTCACCATGTAG 0.55065827622

TGTTGAGGTGGTGCCCAGCGAG 0.898179790173

TGTTGATCAGGTGTAGAGTGAA 0.870743694126

TGTTGATCCTCTCATGGCGAAA 0.743973239383

TGTTGATGCAGTGTGGCCTAAC 0.980180129036

TGTTGATTGTGTCGATAAGGAA 0.658620523306

TGTTGATTTCCTCCACGACCAC 1.2361937747

TGTTGCGTAACTTCTAGGATAG 0.48631624728

TGTTGGTCCTATGTAGGATAAC 0.512980454866

TGTTGTACCAAAGATTGAACAA 0.309990283929

TGTTGTGAACTTCATCAATTAT 0.505640269648

TGTTGTGAGACTGGTGATGCAC 0.658305945778

TGTTGTGAGTCTCCCAGTAGAC 1.0089083046

TGTTGTGCCATTGATGCAGCAA 0.964846926884

TGTTGTTACAATGATGCTCTAA 0.534968051906

TGTTTACCCATTGATTCTACTG 0.930478754936

TGTTTACTATTTGCGACAGAAA 0.695939662791

TGTTTATCATCAGCAAGGAAAC 0.478713882582

TGTTTCGTAGAGGAAAGTGCAT 0.373568859094

TGTTTTGCGAATGCTTAGCTAT 0.534174568081

TTAAATGCAAGTCAAGGTGAAC 0.238433478023

TTAACAGAAACTGATTATAATA 0.398498840472

TTAACATCAATACAAATTAGAG 0.213412109136

TTAACTCCCTCCCCGTGGATTC 0.959309353314

TTAACTGTAGATGTTTAGTGAG 0.24093594259

TTAAGATGGTCTTAGCGCATAG 0.678676165082

TTAAGCTTCGTTCCTTAGGTAG 0.624874993584

TTACCAGAAGCGGATCCCAGAG 0.564749146396

TTACCCGTGTCTCGTAGAGAAT 1.40312553645

TTACGCTCCCCTGCCCATGGAA 0.709680858676

TTATCCGGCTGTAAACCACAAA 0.422272375237

TTATGAAGTTGTGCTAAGTGTT 0.450216819167

TTATGTGTACGTGTTGCGGAAG 0.645612142456

TTATTAGTGCTTGATAACTATC 0.393825178124

TTATTATAGTTTGATCAGTTAT 0.310312899621

TTCAAAGACAGTCATGCTTCAG 0.194741648469

TTCAAAGGGTGTGATGTACTAT 0.559066175592

TTCAAATAGTCTCCAGACAATG 0.428412698816

TTCAAATTCTCTGAAAGCCGTA 0.90621410171

TTCAAGGACTCAGCAGTCGATC 0.808111364715

TTCAAGGTATAGCAAGGGATTC 0.427640238144

TTCAATCAAACTTCTTCGCGAG 0.462350526849

TTCAATCTAACTGCTGGCAATA 0.326594026708

TTCAATCTAGGTCTTGCTAAAC 0.70094727453

TTCAATGAAATTGACTACCTAA 0.324010508134

TTCAATGAAGGTGAATACTATG 0.351674084465

TTCAATGAATCTGCGGACAGAA 0.542875264644

TTCAATGTATTGGGTTGATAAG 0.751112269059

TTCAATGTTTTCGCTGAGAAAG 0.962491308987

TTCAATTAAACTCCTAAACGAA 0.389795747406

TTCAATTAATGTCACCCGAGAA 0.445321330808

TTCAATTGAGCTCCTAATTGAC 0.623371176707

TTCAATTTATCTGGAATCCTTA 0.862435519982

TTCAATTTCCGTGCTTACCATC 0.418486320013

TTCACAAGGAATTTTTATTTTG 0.609539754595

TTCACACAACGTCGCTCGGTAC 0.854052307695

TTCACACAACTTCCATGGTTAT 0.605092960863

TTCACACAAGGTAATTGGTAAA 0.340185107435

TTCACACACAGTGAGAAGGGAC 1.15950569538

TTCACACCTCGCCAAACCAAAG 0.614624590534

TTCACACGATCGTCTATCTTAG 0.433745369918

TTCACACTATATCTAAAAGAAG 0.294853193363

TTCACACTATTTGTTTGAAATC 0.637979558526

TTCACACTTGATGCTGCCACAG 0.778665313083

TTCACAGACGCTGGTCATATTA 0.740533773252

TTCACAGATAGCGCGGAGATAA 0.677830830632

TTCACAGCACCTGAGGGGCGAG 1.83416487604

TTCACAGTAACTCATCATTTAC 0.264083394526

TTCACAGTAGGTCTACTTATAG 0.395901414301

TTCACAGTCTCTCGTCCGTAAG 0.579984097214

TTCACAGTGGGAGGTGGAATAG 0.897096178464

TTCACAGTTTATCCATCACCAA 0.563318416609

TTCACATAAACTGCACTTGATC 0.382347554105

TTCACATCAATAGCTTAATTAC 0.478045507889

TTCACATCATGTGATTACCAAC 0.559856045886

TTCACATGAAAGGCTTTGCTAC 0.694824023216

TTCACATGAGCTGCCTGGGTAA 0.647038741768

TTCACATGATGTGGACGTTTAG 1.09415889451

TTCACATTAAACGGTGAGACAT 0.689684563079

TTCACCCTCTTTCACATGGATT 0.263421152362

TTCACCGGTTCTGCACGATATG 0.961070667047

TTCACCTAAAGTGGTCGGTATA 0.535008006186

TTCACCTAGTCACGCAGGAATG 0.884275755836

TTCACGAGAGATCATTAGGAAT 0.283310855617

TTCACGCGGTTTGTTTAACAAT 0.900163241286

TTCACTATTTGTCAGGGGAAAG 0.272981080727

TTCACTCAATTAGTTAGGGAAG 0.378196331695

TTCACTGAATAGGAATAGATAT 0.588379111653

TTCACTGAATCTTATTCCTTAA 0.276793890663

TTCACTGACTGTGATCAGGAAG 0.884270544279

TTCACTGAGTCTGGTGGATTAT 0.0979842478178

TTCACTGAGTGTGATACCGTTG 1.00163374019

TTCACTGGAAGTGGAACTATAT 0.510771355248

TTCACTGTCGCTCGAGCGCGAT 3.61415625705

TTCACTGTGTCTCACGAGACAA 0.879733774289

TTCACTGTTGCTCGATCAATTG 0.307443046625

TTCACTTACTTTTCAAAATTAC 0.483974550497

TTCACTTATACTCCTGCATTAA 0.501512105537

TTCACTTATTCTCGGTCGAATA 0.535506337736

TTCACTTCAGTTGAGAGGGAAG 1.69975995586

TTCACTTCCTGGGGTCCATGAC 1.1005062272

TTCACTTGCAGTTCTGGACTTT 0.966687114689

TTCACTTTGTGTGTTTCCAATT 0.481382690287

TTCAGAATGCATGCAATGTTAA 0.458968621167

TTCAGACAATGTGCTTTAAATA 0.375669387003

TTCAGACACTCTCATATTAGTC 0.748214156204

TTCAGACCGCGTGATCTTTTAT 0.751175410471

TTCAGACGAGGTGCTGGGTTAG 0.738542161648

TTCAGACGATGTCGTCCTCAAT 0.190029706226

TTCAGACGCTCTGGATCGTGTA 0.898801098152

TTCAGACTGCATGATCTCGCAT 0.743494286387

TTCAGAGCCTGGTAATAGCAAT 0.378076105127

TTCAGAGGATCTTATCTGGTAT 0.596689688943

TTCAGAGGGAGTGCTCAATATT 0.643083983102

TTCAGAGTGGCGCCTGAAACAG 0.646555943034

TTCAGAGTTTTTCGAATATCTT 1.01511996156

TTCAGATAACCTCGTTAGGTTG 0.571440656782

TTCAGATAAGGTCAATGTAAAA 0.519954787078

TTCAGATCACTTCGTGACATAG 0.379935546323

TTCAGATTCATTCATCAGGAAG 0.44326746207

TTCAGCCAATTTGACCCCAAAT 1.18360821689

TTCAGCCCCCTTCTAGTGTCAC 1.85615356857

TTCAGCCCTGCTCTAGTCTAAG 0.425178432386

TTCAGCGAACCGGGTTATAAAG 0.6830897098

TTCAGCGCCTATTATTCAGGAT 0.702063020891

TTCAGCTCATCTCGTATATGAC 0.242079580622

TTCAGGCATTGTGCTTTAAAAG 0.649934763701

TTCAGGCCAAATGCAGCGAATC 0.279734680879

TTCAGGGAAGGTCACTGCTTAG 0.469302073752

TTCAGGGCTGCTCAAGGTATAA 0.500405592663

TTCAGGGTACGTGGAGCCAATT 0.646696616375

TTCAGGTAAACAGGTAGCTCAT 0.64488294882

TTCAGGTACGGTCGATACATAG 0.439006372246

TTCAGGTCTTTTGAAGCGATAA 0.622768060606

TTCAGGTTATATGCTATGGAAG 0.728759972801

TTCAGTCAATACCCCCTGTAAA 0.486708671169

TTCAGTCACGACCTTAGAATAT 0.426533638798

TTCAGTCCCGCTGCTAACATAA 0.366378951155

TTCAGTCCGTCTCTCCATTATC 0.472611856057

TTCAGTCCTCCTGATACAGGAT 0.653493997635

TTCAGTCGCGATCCTTCAGGAT 1.25868324864

TTCAGTCGCTCTTCAAGCTATG 1.25333299488

TTCAGTCGGAGTAATAAGTGAT 0.800511322189

TTCAGTCTAAAAATTTACGGAG 0.723711923415

TTCAGTGAAAGTCTTACTCTAG 0.151659726039

TTCAGTGAAAGTGAACGTCTAG 1.4248045832

TTCAGTGAACGCCTGATAGATG 0.542669228591

TTCAGTGACAGTGCTCCCCGAA 1.18122600239

TTCAGTGGTTCTGGTACGAATG 0.577541763643

TTCAGTGTCCGTCATATTGAAA 0.637588565282

TTCAGTTACTATGAATAGATTC 0.22590067487

TTCAGTTACTATGCACGAGATC 1.16507231221

TTCAGTTAGTCTGCTAGATATT 0.222967550945

TTCAGTTCCACTTAATTTATAG 0.229553881591

TTCAGTTTAGCTTAGGCTGAAC 0.583645571492

TTCAGTTTCTGTCAGAACTTAA 0.44844483922

TTCATACTATCGGACGGGAAAC 0.542642491845

TTCATAGATAGTTAAGGGCAAC 0.490808560809

TTCATAGCAGGTCGTACAAAAC 0.450357827497

TTCATAGGAGCTGCTTAACTAG 0.48245890623

TTCATATAACCTGCTTATGAAC 0.606355626866

TTCATATATGTGTCTAAGATAA 0.639692892685

TTCATATCTAATGATGTAAGTG 0.479352502671

TTCATATTCTATCCCGCTAAAT 0.515994908678

TTCATGTTTTGTGTTTATGTAT 0.387026260705

TTCATTCACTCTGAAGATTCAG 0.250767200905

TTCATTGCAACTCTTTGAGCAG 0.179617300545

TTCATTGCATCTGTTTGGAAAT 0.258783923989

TTCATTGTGTTTGTATCAACAC 0.76409937562

TTCCACGAAGGGGATGGCACAG 0.400941633003

TTCCACTCATCTCGTCTTATAC 0.806149786717

TTCCATGTGAGGCCCGCCGAAA 2.04913308404

TTCCATTTAACAGAACTAACAC 0.465358228908

TTCCCACACGCTGATAGCCATC 0.725285194551

TTCCCACAGATTCCTAACAGAA 0.674768869023

TTCCCACATACTGTCTCACAAC 0.419225483725

TTCCCACTGTGTTGAGGTGTAG 0.996016172508

TTCCCAGAGTGACTTATCTATC 0.378883006527

TTCCCAGCGTCTGCTCAGAAAG 0.846114698008

TTCCCAGCTGCTGAAGTCAGAT 2.80968937217

TTCCCAGGAAGTGCTCTACAAT 0.481033046411

TTCCCAGTAGTGGGTGGTCTAT 1.47443958203

TTCCCATAAATTCCTTCAATAA 1.24433715813

TTCCCATAAGGTTACGGGCAAG 0.821165060371

TTCCCATGATCCGGTGACGCAC 0.513332459913

TTCCCATGTAATGTTCGGCTTC 0.354525699557

TTCCCATTTTCACTTGAGGAAT 0.788415042163

TTCCCCCTGAGTGATAAGTTAA 0.873224901101

TTCCCCGAGACTGGGTCTTAAC 1.22662145791

TTCCCCGAGGCTTATTACAAAG 0.39631170846

TTCCCCTAACGTTTTATTTAAA 0.305805120957

TTCCCGAACTTTTTTAAGCAAC 0.651763808509

TTCCCGACACCTCTAGACCGAC 0.832704898577

TTCCCGTACTTGGTTCAGTAAC 1.06698116497

TTCCCGTGGGATCTTATCATAT 0.218359497694

TTCCCTACCGCCCGTGTAGTAG 0.67178482908

TTCCCTCACCCTGCTGGCTTAA 0.843241120407

TTCCCTCAGCAAGACGCAGTAC 0.572705108456

TTCCCTCTGACTCATTTAGAAT 0.372286127044

TTCCCTGAGGGTTACTCACAAT 0.858672018063

TTCCCTGATTTTTATAATGAAG 0.620026550816

TTCCCTGCACCTGATGAACAAG 0.397840049603

TTCCCTGCCTCTCATCGCCTAT 2.06100571823

TTCCCTGGATCACCTCCCGATT 0.800608729613

TTCCCTGTCTTTGCTCGCCAAG 0.422894932748

TTCCCTGTGTGTCAGGCAAAAG 1.42297443757

TTCCCTTAATATCGGAGTTGAG 0.421100296247

TTCCCTTAATCTGATGACGCAA 0.535700276904

TTCCCTTTACCTGTGACCCAAC 1.38090261521

TTCCCTTTATCTTCATGAATAC 0.367335493488

TTCCGACAACCTCGAAGAGAAG 0.802925082275

TTCCGAGACACTGCTAATACAG 0.180863192921

TTCCGAGTATTCGATGGATAAC 0.308595018147

TTCCGAGTCTCTTTCGGCCGAG 0.750345063133

TTCCGATAGTGACCTATAAAAA 0.378011091445

TTCCGATCATTTCATTTCTTTG 0.525938464915

TTCCGATTATGTCCGCAGATAC 0.482201570543

TTCCGATTGTCTCATGGTAAAT 0.461288907462

TTCCGATTGTTTGGTTGAGAAC 0.793629163318

TTCCGCCAAGCTGCTATTGTAC 0.333713092623

TTCCGCGGCTCGTTTAGGGCAG 1.83076206656

TTCCGCGGCTGTGTCACACAAG 1.32555740458

TTCCGCGGGCTTCAGAGAATTG 1.81213306385

TTCCGCGTATTTGAAAGGATAT 0.32092058048

TTCCGCTTCTGTGGAACAAGAG 0.847616829416

TTCCGGGGGTCGCCATGCGTAC 2.14278812817

TTCCGTCAAGCTGATTCGTTAG 1.31089564278

TTCCGTGCAACTCGTCTTTTAC 0.316889595973

TTCCGTGGAGTCGAATGCTAAA 0.932136835549

TTCCGTGTAATTGCGTGTAAAT 0.730116732333

TTCCGTGTCGATCACGTCTAAT 0.792756480001

TTCCGTGTCTACCATGCCGAAG 0.469800552591

TTCCGTTAATGTGGCGCTGAAT 1.23676488386

TTCCGTTGAGCTTAATCGTAAT 0.276080820576

TTCCGTTGATGTGTACTATTAA 0.182743051354

TTCCTACCCACGTGTAAAGGAA 0.61126355941

TTCCTAGAGACTTGTACACTAA 0.428436403597

TTCCTAGATTCTCATGTCCATG 0.47913003382

TTCCTAGTCTCCGCTGATTATG 0.928164978224

TTCCTCGTATGTCTAACCTCAT 0.591961390741

TTCCTGCTTACTTAAGCTGGAT 0.340914095514

TTCCTTCTGCTTCAAAGATGAA 0.301812004453

TTCCTTGCAACTCTTGTTGAAG 0.232477641249

TTCCTTGTATGTGTAGCAGTAT 0.845728689944

TTCCTTGTCAGTCCTGATGTAC 0.320645587711

TTCCTTTCGTGTCACCATCATG 0.827547997634

TTCGAACATTATGTAGGTGTAC 0.448266056059

TTCGAACGAAGTGGAAGCTATC 0.879622807704

TTCGAAGACCCTGTACGCGTAA 1.15538901812

TTCGATCTTGGTGTCGGGCGAA 0.2470169234

TTCGATGAGAGTCGATGGCGAC 1.11271150396

TTCGCACTTAGGCGGGTTTAAT 0.68383895687

TTCGCAGAATGTCACGTGCAAC 0.734082780613

TTCGCAGACTGTCCTGGTGTAA 0.274152109002

TTCGCATACTTCCAACACCCAG 0.677509403829

TTCGCATATTGTTAAGAGGGAA 0.754148707139

TTCGCATTAATTGATAGACAAC 0.253246407189

TTCGCCCAATCTGTATCCCATT 1.16396996184

TTCGCCGAAGGTGATAAGTGAT 0.654696786068

TTCGCCGACGCTGCTCTGTTAC 1.5955892659

TTCGCCTCGTCTAGATATATTT 0.908309581246

TTCGCCTTTGCTGCAAATGCAT 0.538091160706

TTCGCTGCTTGATATGTCTAAG 0.672262954641

TTCGCTGTCGCTGTAACAGAAT 0.912624571838

TTCGCTGTTCCTGCTTAGGTAG 1.00456843925

TTCGCTTTAACTCATGCGTTAG 0.678044777523

TTCGGACATTGACCTGAGTTAG 0.188897667329

TTCGGACCATGCCCAAGGAGAG 1.36999586579

TTCGGACTATGTGAACTTTGAT 0.604395485397

TTCGGACTTTCTCCCTAGTTTA 0.561413617222

TTCGGAGAAGCTGGAAAAAGTC 1.0325652711

TTCGGAGACATTCTAGGATAAA 0.262952990951

TTCGGAGAGAGTGATAGTCGAA 0.737046743262

TTCGGAGCATCTCAAATTTAAG 0.49242454605

TTCGGATAGTGCCAAAACGGAG 1.11660953384

TTCGGATAGTTCGATTTATATG 0.393052518686

TTCGGATCGCGTCATCGATAAT 0.26683036913

TTCGGGACAGGTCATCCCAAAC 1.3792449822

TTCGGGGTATGGTCTCAGTAAA 1.46920418239

TTCGGGGTCCGGGCTCAGTCAA 0.675782588977

TTCGGTATAAGGCCTATGAGAC 0.69177358872

TTCGGTCTGTTTCGTCTGGTAC 0.160945762446

TTCGGTGGAGCTGCTACGAAAT 1.21334148129

TTCGGTTAGGCTGGTGACTTAA 0.64352265135

TTCGGTTGAGGTGTTGCGGTAC 0.276320024533

TTCGGTTGTCCGCAAGCAGCAG 3.96282735178

TTCGGTTTACGACGATATAAAA 0.465948849685

TTCGTAAGACCTCATGTCATTT 0.867968236628

TTCGTTCTGACTGCTATAGAAA 0.407247158771

TTCTAACAAGATTTGTAATATG 0.388051287155

TTCTAACAGTGTGTAGCGCAAC 0.822022935414

TTCTAACATTCTCCCGAGCGAG 0.448493552417

TTCTAACGATGTGAAAAGAGAA 0.491835709725

TTCTAACTAGTTGTTTTTAATA 0.354858228232

TTCTAAGAGTCTCCTTGGGCAA 0.632019375325

TTCTAAGTGTGTGCACAATAAG 0.638057088825

TTCTAATAAGGTTCTAACTAAT 0.423810625372

TTCTAATCCTGTCATTGACAAT 0.614666064784

TTCTATCAAGCTGAAGGTAAAG 0.749586214472

TTCTATCACCTTGACTGAAATC 0.537245327532

TTCTATCATCGTCATCAATGAT 0.444755378779

TTCTATGACACTCACACTATAC 0.452862950892

TTCTATGACTCTCACCTGTCAT 1.1778914296

TTCTATGTCTGAGTACTGATTG 0.512720880226

TTCTATTCGTGTCCAGCAACAC 1.31476278227

TTCTCACACTCTGGAATACTAT 0.19602966998

TTCTCACACTGACTAGCTTTAA 0.537233013289

TTCTCACTAGGTTTGAATAGAA 0.338226290972

TTCTCACTGTCTCATGTAATAG 1.12038354323

TTCTCAGAACATCGTCTAGGAT 0.425690230016

TTCTCAGAATGTCTTGACCAAA 0.590365930528

TTCTCAGACTGTCCTCCTGTAG 0.227165493275

TTCTCAGATGACGCTAGCGTTG 1.02990041514

TTCTCAGGAAGTGATGGATTTC 0.864984072576

TTCTCAGGCACTCAACCTTGAG 1.16056659633

TTCTCAGTAACTCAAAAGGCAG 0.700007869056

TTCTCAGTACGTGAAATCTAAG 1.09116631809

TTCTCAGTATTTGCCGGAACAG 0.665890830923

TTCTCATACCCAGCAAACAAAG 0.705552882571

TTCTCATACGGTCTCAACGAAG 0.894884085263

TTCTCATACTGTGTTGTATAAG 0.520008283633

TTCTCATAGATTGAATTCGCAG 0.812306080557

TTCTCATCCGCTGGAATTGAAT 0.555998831523

TTCTCATCTGACCCAATTGTAC 1.15178057437

TTCTCATTAATTGCAGTGTGAG 0.770065932834

TTCTCATTACGGGAACGGTAAT 0.443076631521

TTCTCATTAGTTGATGGTCGTG 0.848836989973

TTCTCATTTTGTCACAGTGATG 0.7271923633

TTCTCCCACATGGCTGTAGAAG 0.7402245286

TTCTCCCTACCTCATGAAGGAT 0.670462425137

TTCTCCCTGTGGCATCTAGTAG 0.448813253644

TTCTCCGACAATCTGGACTTAC 0.729183373896

TTCTCCGGAGATTAAGCCTCAC 0.941422799053

TTCTCCGGATGTCCTGTTTTAT 0.681722722624

TTCTCCGTATCTCTACACATAT 0.420721117188

TTCTCCTAGTGTCATATTGATG 0.701658686795

TTCTCCTTATTTTAATCAGAAC 0.284412250493

TTCTCGCACTCTGCGAGGATAA 0.321471712134

TTCTCGCAGTCTCCTCGGTGTT 0.965520481091

TTCTCGGCATCTCATCGATCAA 0.321207072009

TTCTCGGGATGTCCCATTTAAA 0.416372541007

TTCTCTCCGTCTCGATCTATAA 0.605137286456

TTCTCTCTAACTGATTATTTAG 0.401882200461

TTCTCTGCTGACGCTGTTTTAA 0.97161339477

TTCTCTGGATGAGTTACAACTT 0.658366869246

TTCTCTGTAACAGTAGGTTCTA 0.796382975862

TTCTCTGTACTTCCGCGGTAAA 1.21224110721

TTCTCTGTAGCTTCAGATAGAG 0.591916365344

TTCTCTTACCCTTTTCGTTTAG 0.586371612537

TTCTCTTACGGCCCGCGTATAG 2.23453598872

TTCTCTTACTCTCCCGGCTCAC 0.416420370792

TTCTCTTTATCTCGAAGAAAAC 0.304152889574

TTCTCTTTCTGTCCGTGAGGAA 0.708010753262

TTCTCTTTGATTGCGTGGCATG 1.12603923879

TTCTGAAATCATCCTACGGTAC 0.525066468365

TTCTGAATAGAGGCTTATTAAT 0.455818889804

TTCTGACAAACTGAAATTTAAG 0.207324096103

TTCTGACAACTTCACTAGAAAG 0.437037572968

TTCTGACAATGTCCAGCTTCAA 0.252417153075

TTCTGACATCCTGCCCCTTGAG 2.32593169544

TTCTGACCCTCTTGATGACGAG 1.33330800668

TTCTGACTCTGTCCATGTACAG 1.03359224809

TTCTGACTGTCTGATGGGGTAG 0.745655104835

TTCTGAGACCATCCATCCTAAT 0.381306064829

TTCTGAGATAGTGTTGATAGAA 0.22692593796

TTCTGAGATTCTCGTATCAGAC 0.297460608901

TTCTGAGCGTCTCCAAAAGAAG 0.784983217964

TTCTGAGGGTGCGCTAGGAAAT 0.915981052437

TTCTGATCACCTGCGTGTTTAC 1.06535243885

TTCTGATCGCCTCGTGTCGGAA 0.741108498517

TTCTGATCTTGTCGAACGCAAC 1.08418371321

TTCTGATGGCCTTACAATCAAG 0.546303889885

TTCTGATGGTGTGATCGTATTT 0.462933719627

TTCTGATTATCCTGAAGATTAA 0.360280320782

TTCTGATTTTCTGCGTTCAATT 0.48506966533

TTCTGCCCTATCGCTAAATTAC 0.339454311721

TTCTGCGACTGTCCTACCTAAA 1.1277443904

TTCTGCGCAGGACAATACGTAG 0.959897226594

TTCTGCTACTGTGCAGGATTAA 0.290771790049

TTCTGCTACTGTGCTGGCCAAA 0.975682027981

TTCTGCTAGTCTCCATGCTGAG 0.408646120961

TTCTGCTTATCTCTAAGATAAT 0.256263524013

TTCTGGCATGGTCAGTTCCTAG 1.09363633051

TTCTGGGTGTCTCTAGTCTGAT 0.367750477676

TTCTGTAAGGGTGGCCGATTAT 0.516667860371

TTCTGTCTATATTGTATAGTAA 0.27467351507

TTCTGTGATACTGCTCGTTTAG 0.73798782082

TTCTGTGTGAGTGTGAAACTAC 0.757057547732

TTCTGTTAATTTTGAGGCCAAA 0.704469292084

TTCTGTTACCGTCGTCCACATT 0.345008457071

TTCTGTTACTCACATATGGTAA 0.284313908691

TTCTGTTCAGATGCAACCCTAA 0.367027652496

TTCTGTTGTGGTGCTATTGAAA 0.601279303335

TTCTTAAGATGTGAAATGGAAG 0.301286138906

TTCTTAGCAGTTCATGTCTTAA 0.513150873353

TTCTTAGTCTCTCCTTCAGAAC 0.365650790917

TTCTTAGTTGGTGAGCGAAAAC 0.586470243705

TTCTTATATGCTCATGCTATAA 0.266101930968

TTCTTATGCAGTGGATCTTTAG 0.922457792427

TTCTTCAGTTGTGCTTGCTAAC 1.37538090673

TTCTTCTAGGCTGCGATTTATG 0.675675660515

TTCTTCTATCGTGATGATTAAA 0.442874773233

TTCTTCTCCCCTGCTCTTCTAC 0.787321188796

TTCTTTCAAAATCGTGAATAAG 0.380500691022

TTCTTTGAATATCCATTCGTTC 0.975047273678

TTGAAAAACCGTGATGAATAAT 0.621445845231

TTGAAACCGTTTGCTAGTGAAC 0.567858378797

TTGAAAGCGTTTGGTGCCTCAC 1.54825486756

TTGAAAGGGTCTGTTCAGCGAG 1.45091576371

TTGAAATCATAGGTTGGGATAA 0.442508925241

TTGAAATCCGCTCGTCGAAGTG 0.709245426239

TTGAAATCGACTCCGACGACAG 0.469763028261

TTGAACGAATGACATTGCTTAC 0.402979957724

TTGAACGCAGCTGCAGTTCCAA 0.315905489365

TTGAATATATTTGTTGATCTAT 0.198100191278

TTGAATTCAATTCATTTATAAT 0.447384013067

TTGAATTCATCTCATTAGTCAA 1.00807696388

TTGACAACATATTTTGGAAGAA 0.292134484321

TTGACAATCTTTGCAGTAATAG 0.607074795607

TTGACACAAATTCATTATCGTC 0.638968009167

TTGACACAGACTGCCCCTACAC 0.799005284071

TTGACACAGTCGCCTTTCTGAT 0.556121854138

TTGACACCGGCGCATGGCTATA 1.29485730553

TTGACACCTTTAGTTAGGACAA 0.381991315741

TTGACACTATGAGAATCCGAAA 0.608872994653

TTGACACTATGTGCTGGCCTAG 1.88813497973

TTGACAGAGTATGAAACATGAG 0.37584297517

TTGACAGAGTCTGAACCATGAG 1.01470384482

TTGACAGATTGTCTAGAACTAG 0.457333106845

TTGACAGTAGGTGCATAACAAT 0.494489375161

TTGACAGTATCTGGAGTATAAC 0.50366678625

TTGACAGTTTGTCATTCCAGTA 0.174471273271

TTGACATAAACTGGATTAAAAT 0.120311151571

TTGACATACAGGGCTCCGAAAT 0.440120641446

TTGACATCAGCTCATAGGCTAG 0.976707136208

TTGACATCATGTTGTACAAGTA 0.49500003501

TTGACATTTGCTGCTATACAAG 0.583232807897

TTGACCGAAGGTGTTACCGAAA 1.18847549426

TTGACCGAAGTTGAGACGTAAC 0.957076627917

TTGACGCCATTTCATGTATATT 0.584632174246

TTGACGCGTCCTCATAACCAAC 1.06427439724

TTGACGGTATCTCTTCAGGTAA 0.998084846118

TTGACGGTCGTTCAATATCAAC 0.584544130084

TTGACGTATACTTATGGCAAAC 0.883122541113

TTGACGTCCGGGTCTAGGTTAG 0.800318426716

TTGACTGAACCTCATGCACCTG 0.515550853935

TTGACTGAACCTGAATTAGCAG 0.472634919976

TTGACTGACAATGCACACGCAC 0.856705355827

TTGACTGGATCTGCAAGTAAAT 0.421738073438

TTGACTGTCTGGTATCTATTAG 0.18553887785

TTGACTGTTATTTTCTAACGAT 0.53496166078

TTGACTTATCGTTCTGTGTCAG 0.643601293539

TTGACTTATTTCCATAATGAAG 0.412753419904

TTGACTTCAGTGCATCGCAGAT 0.837892999787

TTGAGACAACGTCCCAAAAGAC 0.535070262652

TTGAGACAATGTCCTGATTGAG 0.589011192535

TTGAGACAATTTTGAATCTGAT 0.646181462588

TTGAGACAGCCTCGTCTCGGTG 1.40279857736

TTGAGACCCTGAGAATCAGTAG 1.07629610451

TTGAGACTATGCCCTGTTCCAG 0.330557022654

TTGAGACTTGCAGTACCCTTAA 0.863181023377

TTGAGACTTTTTGATGGGTGAT 0.68826728945

TTGAGAGAAGCCGCACTTGAAG 1.09335159976

TTGAGAGAAGTTGCCACAGTAG 0.628734837424
[truncated: 13,156 more chars]
