# Supplementary material for: Weak base pairing in both seed and 3′ regions reduces RNAi off-targets and enhances si/shRNA designs
Source: Nucleic Acids Res. 2014 Sep 30;42(19):12169–76. doi: 10.1093/nar/gku854 (PMC4231738; doi:10.1093/nar/gku854)
Supplement: SUPPLEMENTARY DATA [file supp_gku854_nar-00955-y-2014-File009.docx]

| **Active shRNAs (potent on-target efficacy)** | **Inactive shRNA** |
| --- | --- |
|  |  |
| TGGGAGGAAAGTGATATCAAAT | GAGACTCAAAGTTAATATTCTT |
|  |  |
| ACCAGTGAATTTCTCAATCTTG | AAGGAGGAGTCTGAAGAGTCAG |
|  |  |
| CGGTGGTACATGAACCGATTT | GATAGTGTACATTCTTCTTAAA |
|  |  |
| CACATGTAAATTCACCAAATAA | GTGCAGATTGCTTGTATGTAA |
|  |  |
| ATCTTTGGAAATGAACAGATAA | ATGGCTACATGTGCTTAAGGAA |
|  |  |
| CTGTGCCAGAAACCCTTAAGAA | GTGATCATGTCCCAGATCTTG |
|  |  |
| AGGCAGCACATTGCTTTATAAA | CAAGGAGGAAGCTTATCTATG |
|  |  |
| ACGGCTGAACATCATCTCTAAC | TATGTCATGGCAGATAGTTTA |
|  |  |
| AAAGCACTATCACAAGGAATAT | CGCAGGCTATTTGTTCAGAGAG |
|  |  |
| ACCAGTCGGTCTTTAAATGTAC | GGGCCAAACTGTGCAAAGTGAG |
|  |  |
| ACTGGATCCTGCTGCTGTAAT | CACAGAGGAAGTGAAATTTGAG |
|  |  |
| CTGTGCTTATCGCCTGGATCTA | TGCTGTTAGTGACAGTTATTAC |
|  |  |
| CACCTACTGTCTGGTAGCGATC | CGTCGCTTCAGTGAAGAAAGAG |
|  |  |
| TGGAGAGTGTCTTCAAGCTGAA | AATTGTTGTAGTGGATTGGAAC |
|  |  |
| CAGTGACTGTTTGCTGGATAAC | TGTTGTGTCCATTAATGTTTAT |
|  |  |
| AAGTCAGCAACTAATCATTAAA | CTTATTGACTCTGGTAAACTTA |
|  |  |
| GGCACAGGAAGTTCTCATTGAT | ACTCCTTTCTCTCATCTCAATT |
|  |  |
| TGGTGTGATTGTCCTGAGCGTC | CCCACCACATTTACCCATATT |
|  |  |
| CTGACTGAGCCTGCTCATTGTT | CTCAACTAGCCTGGAAAGTTAG |
|  |  |
| GATGCTGCAATTTGTCAGTAAT | GCAGACCAATCACATTATTAT |
|  |  |
| TGGCTGGTAAATGGTGTGATT | GTTGGCCAAAGCCATTGCTAAT |
|  |  |
| GGTGCTGAAGCTGGCAAATCAA | TTTGTTGTATCTTCTATTACAT |
|  |  |
| AACCCATAATATCATTGAGAAA | TCTTGGACAATTGCAAATCAAA |
|  |  |
| AGGAGCTCTTCTGCTGAGGAAA | AGCAGTTGACCCAGGAATTAC |
|  |  |
| TTGTGGTTAACTCATTTAAGAA | CACGTGCACTCTCTTCCTGTAC |
|  |  |
| ATCTGAGAATCACATTTGGTTT | ACTTGCTAAGCTCTCCGAGAAA |
|  |  |
| TACCCTCTGTGTACTCAGAAAT | AACCCAGAAGCTGCAGCAGGAA |
|  |  |
| CACAGTGACAGAGATTGGCAAA | TCCACTACATGGTGCACACATC |
|  |  |
| GGCCCTGAATGCCCACACCTTC | CGGAGAACGAGCTGAAGTTCC |
|  |  |
| GTGTCTTCATTTGTCGAGGAAA | GCCCTTCGAGACAACGAAATT |
|  |  |
| GGTGGATGTGATCTTTGCTGAT | CTCTGAATATGTGGAAACTATT |
|  |  |
| GGGCATGTGGGTGCTGACTTAG | TGCCTATCATCTGCAGCTCCAG |
|  |  |
| GCGCCGTTCTGTCAGTGACAAT | GCAATTCTTCAGAAAGGTTTA |
|  |  |
| CACAAGCAGTTTCTAAACCAAA | TTCAGTTGTTGTTCTGTATCAG |
|  |  |
| AGAGGTGTATGAGCAGCTTTA | CATGATTCAGTTCAAACAGTAT |
|  |  |
| GGGACAGAAAGATGACCTCAAC | TTGAGGCCCTAACAAATTTCAG |
|  |  |
| TGAGGGTGAGCAGCATGAGAA | TCCTGCCCAGCGGCACCCAAAG |
|  |  |
| AGAGGTGTATGAGCAGCTTTAT | AGCCCTGCGTTCTGTGCTTCT |
|  |  |
| CATAGTTGATCTTCGGCATAAA | CTGGGTGATTTTGACCGCTTC |
|  |  |
| ATTGCTCGATCTCTTGGTAAAT | AGTTAAGACTCTCCAAAGCAAA |
|  |  |
| AGTCCTGTATATGTATGTAGTA | TGAACCAGTGGTGGATCTTTA |
|  |  |
| GGGAGATGGTGTGGGAGCAGAA | GAATAGCCCTTCCATTATAAA |
|  |  |
| TCCCTGTTATCTGCTAGATCTA | TGCAGGAGCTGGAGTGCATTT |
|  |  |
| TGCAGAGGATCACATTAATTTC | CAGGAGGACTGGGAATACATAA |
|  |  |
| TAGGGCTCATATTATAAGTGAT | GCCTCAGGATCACAAATGTTAC |
|  |  |
| GTACTACGACACTGAACGCAT | ATCCCAGGAAATCATAGAATTA |
|  |  |
| GAATATGTATGAACTGCATAA | ATGGAATTATTTCCCAAGTTAC |
|  |  |
| CACAATGAGTCAGAAGATGAA | TAGAATGCATGCCAGTTCTGAT |
|  |  |
| TCATCTCTACGTGTATATCGAA | TTCCAAATATCCGATGCCCTAG |
|  |  |
| ACTCCTTCATTCCCAAATTCAT | ATGATATCACCAGCCGAGAAAC |
|  |  |
| AACACTGAAGCTGGAGCTGTTA | CGGAGATGCTGCCTTGAGACAG |
|  |  |
| GCAGTGCAACACAGAATTAAA | CTCCGTTTACTACATTCTACAT |
|  |  |
| GGCAGTCAGTCTCAAGATGAAG | AACTCAGAGGGTCAAGATGATT |
|  |  |
| TTCAGCTTAACTGACAGACGTT | TTCTAAGTTCATGTTTGTAAAT |
|  |  |
| TTCTGTGGATTCATTTGATAT | AAGAAACACTCTTATTCCCAAA |
|  |  |
| GAGGCGTATAGAGACAAATTG | TGTAATTAATATCTTGCTGGAT |
|  |  |
| ACCTCTTATTCTCTACATGCAA | GTCCCAGACAATTGTTGTAGAC |
|  |  |
| TAGTCCCAACGCGTTTGCAAAT | GGGAGAAGACACTTGAATATT |
|  |  |
| TTGCCATCAACGTATATTTCAA | AAGAGCTAAGTTCTAAGTTCAT |
|  |  |
| CTTTCTGCATAATGTTGATTAT | TGGTTCCAAACCAGTTTATTC |
|  |  |
| GACTAAGTACCTGCAATCCAAC | GATTCTTCAGATCCATACCAAG |
|  |  |
| CCCTGGGAAATGCATAATGTAA | AGGAGAAGCTCTGGTACTACT |
|  |  |
| AAGATGGAAGCTTGTTTCTAAT | ACGCCAGCCTCTCCAACGACAG |
|  |  |
| GTGACTTAACCTGAATTGTAAA | GACTATGAGATGCACAAGATG |
|  |  |
| TGCAACCTTAATCCTTTACAAT | TTGAGGAGGACAGCTATCTTG |
|  |  |
| TGAAGCGTATGTTGAATTTCAA | GCGTGTGTGTGCGAGTGCGAAT |
|  |  |
| AGCAGTGAAACTAGACAAGAAA | CCCCCGATCAGTGTCAAGTTC |
|  |  |
| CACTGGGACTAGCAATGATAAT | CAGCCAACAACGTGGAGATCC |
|  |  |
| TCCAGTGTATCTCTTTGTATTT | CAGGTGTTTTCCGAGTACTTC |
|  |  |
| TGGAGCTGATGTTCAAGCAATT | GACAGTGGCCGACCAGGTGCTA |
|  |  |
| TTCAATGCACCTTCCCACATT | CTCTCGCAAGTGCCTCCGTGAG |
|  |  |
| AGTAGTCCAGGTTTACAGGAAG | GGGCCATTAAGAGAGTAAATAC |
|  |  |
| TTCCAAAGAGCTGAGACAGAAG | CTTCCCTTTACTCATGCCGAAA |
|  |  |
| GATGCATGTCTGGTTGCTAAT | GTCTATCTGTAGTATTACATAT |
|  |  |
| ACCTCTTTGTCTACTACAAGAA | GGCACATTCACTTCTCCCACTC |
|  |  |
| ATGGCATTGTTTCCAAGTGTTT | ACCAAACACCTTGGTCATAATA |
|  |  |
| ACCCAGCAATGTTCTACGAGAT | TCCACCTTGGGTGAGAACACTT |
|  |  |
| ATGCATGGATGTCTAAACAATT | TGGTCCTTCTTTGAAGCTCTTG |
|  |  |
| CCCTAATCTTGAGTTGAGGAAA | GTGGAAGTTGGAGAAGAATTAA |
|  |  |
| AGGAGTCAAAGAGATGTCTTTA | AGATTTCACAACTGCACAATT |
|  |  |
| GTCTAGGCTGCTGATCAGTAAG | GTCAGGCACTGTGGATGTGAAC |
|  |  |
| AGGCATGATGGGATTCAATAA | AGGTGAAAGAGTCCTACATCAC |
|  |  |
| CTGACTCAGTTGAATACCTAAT | AGTCCAACTTCACGGCTTATG |
|  |  |
| TGGAGCTGAAGTCACTACTGAT | ATTTGGTATCCACGTTGATTC |
|  |  |
| GCATGAAGCTACTCCCAGTTT | CCTGGGCAGCCTTCATATTTG |
|  |  |
| TGGTGGCTCTGTCTACAAATAT | ACCACATGAGCTCCTACAGAAA |
|  |  |
| AGCAGAGGAGAAGAAAGATGAG | AGGACATGGTGCCAAAGGAGAC |
|  |  |
| CGCCAAGGACATCAAGAAGATC | CAAGTTATCTGTACAGCATTA |
|  |  |
| GTCAGATGATGACATGGGATTT | CATCTTCTCCTATTATGATAA |
|  |  |
| TGGCTTCAAAGTCCTGGTTTT | TGCAGTGAATGGGATTGGAAAG |
|  |  |
| CACACAGGCACTGATCTCCCAA | GACACCCGCGAAGACTATATC |
|  |  |
| TGCCTACTTTATGTTGTATAAT | TGTCAGCCCTCTCATACTATG |
|  |  |
| AGGTTTGCAGATGATACATATA | ATGGCTTCCTGGCCTTCCAAAC |
|  |  |
| ATCCTGACTTTGAGCGACTAT | TCCTTGGTGTTTCATGAGAAAG |
|  |  |
| TTGTCATATTATGGAAGGCATA | CATGTATAAAGTGATTGACTTG |
|  |  |
| TGCAGATATCATCTTTGGAAAT | GTCAGCGTTTGGCTTAACAGAT |
|  |  |
| TTCTGATGCTTTGAATATAGTT | ATCTGGCTTCATGATTCCTTTG |
|  |  |
| TGGTTTGAGTCTTACAGAGAAA | TTCATGTATCCTGGTAAAGCAT |
|  |  |
| CCCTCAGCTTCTGAGAATTGTA | CTCAGTTGAATACCTAATTTG |
|  |  |
| TAGGCATATTCTAATTAATGAA | TACCGCTTTGTTGGAAGCAGAT |
|  |  |
| ATGAGAGATCCTAATAATCAAC | AATAGAGAAAGTCATGAAGCAT |
|  |  |
| AGGAGGCAATATGCTAGTCTTT | AGTGGAGAAGATGGAGGTGAAG |
|  |  |
| GAGAAACTATATGCAAGTTATG | TCCTGTGACCCTCATCATTAAA |
|  |  |
| TGGGATCAAAGTTGTCATGAAT |  |
|  |  |
| AACAAAGAAAGTGTTCAGTGAT |  |
|  |  |
| AGGCATTTATGACACTCTAAA |  |
|  |  |
| CTCTGTGTATGCGGATGATTTA |  |
|  |  |
| CAGCCACAGAATATTATGTAAA |  |
|  |  |
| GCGCGGAGCTGTGTCCAATAAA |  |
|  |  |
| AGGCCATGACTTCCTCAGAGAT |  |
|  |  |
| TACTGACTGACTGCAATATTTG |  |
|  |  |
| TCACCTGAATGTTCTATGTAAT |  |
|  |  |
| CAGAGGCTCAGTGAAGTATATA |  |
|  |  |
| TTGGGAGGATGTCTTTATTCTA |  |
|  |  |
| AGGATGTAATGTCCTTCTCATA |  |
|  |  |
| AGGTGATGTAACCATTACAAAT |  |
|  |  |
| GGCTCATATTGGGAATGCTTAT |  |
|  |  |
| AAGGTTTCTACTGCTGTATTAT |  |
|  |  |
| ACAGAAGACAAGTACGAAATA |  |
|  |  |
| GCCATACAAGACACAAACAAT |  |
|  |  |
| GCCATGTGATGGCAAAGCATT |  |
|  |  |
| CAGACAGCAACGGGAAGATAT |  |
|  |  |
| GACCATGGCTGCTGAAATCAAA |  |
|  |  |
| TTGCCTCTAGGTGACTTTCCAA |  |
|  |  |
| TGCCCTCCAGCTTGTATTTAAG |  |
|  |  |
| GAAGCTCTTCTGTCGTTATAT |  |
|  |  |
| GTGACTGGATTTGGCATTTAA |  |
|  |  |
| TCCTCCCGATGTGCCAGTATT |  |
|  |  |
| GAGAGTTACACAGTAAATTAAT |  |
|  |  |
| GACACTCTACCAGTTAATGGAA |  |
|  |  |
| AGCTGTTTATGGCAATTTAATA |  |
|  |  |
| TAGACACCATGTTGTATCAGAA |  |
|  |  |
| AGCCGTCAGATTCCACAGCTAA |  |
|  |  |
| GCCAGTCAAAGTGGCACAGCAA |  |
|  |  |
| TGGTCGGAAAGAGATTGACATA |  |
|  |  |
| GTCTCCATATCTGTATTACTTT |  |
|  |  |
| TACCCGTAATTTGTAACATAAA |  |
|  |  |
| AGTCAAGTTTCTAATACAGAAT |  |
|  |  |
| CACCAGCAATCTGAATGAGAAA |  |
|  |  |
| AACACTGAAAGTTCTAGCTTA |  |
|  |  |
| GCCAACCATGACTGATCAAAC |  |
|  |  |
| TTGGCATGATATTGAGAAAGAT |  |
|  |  |
| TGGAGATGAAGTGTTGTGCAAA |  |
|  |  |
| TATACAGTTTGTGACCAGAATT |  |
|  |  |
| AAGGATTTAACTGCAAAGCAAA |  |
|  |  |
| TAGCTTCATTATGATACGTAAT |  |
|  |  |
| CTGGGAAAGAGTGACTGATAAT |  |
|  |  |
| ACACGACAAATTCGCAGAATA |  |
|  |  |
| CGAAGAAGACATACACGACAA |  |
|  |  |
| ACCGTATATGCGTAGTATTTG |  |
|  |  |
| GGCAAGTCATATTGCTTCTAAT |  |
|  |  |
| GTTAGAGAATGAGGTTGACTAA |  |
|  |  |
| TGGGACCCAAGTCCATGATGAA |  |
|  |  |
| TGGGACCACATCAGTAATTAT |  |
|  |  |
| TCCGCACCAACTCCCTTGTCAT |  |
|  |  |
| ACCGGATAGTTTACTTGTGAAT |  |
|  |  |
| ATGTAGCTATGTGGAATGATAT |  |
|  |  |
| CTCTTTCAATTTCCTTGTGAAA |  |
|  |  |
| AGGTAGCAATCACAAACTAAAG |  |
|  |  |
| ACATCTTACTGGGTTTAATTAA |  |
|  |  |
| ACCAGAGTATTGTATAGTCAAT |  |
|  |  |
| AGGCTAGGAATAGGAACTAAA |  |
|  |  |
| CTCACAGAGATGTGTATTAGAA |  |
|  |  |
| TAGAAATGTTCTCAGACTTTAA |  |
|  |  |
| ACGAGTTCAAGTCTGTATCAAA |  |
|  |  |
| GGGCGGGACACTCACTCAATAT |  |
|  |  |
| GGGCTAGTTTCTTACAATGAAA |  |
|  |  |
| TCGTCATACTCAGAAGTCCAAA |  |
|  |  |
| AACTGGCAGAGTTAGAACTGAA |  |
|  |  |
| GGGTCTCTGTGTATTAAGCTAA |  |
|  |  |
| AAGGTACAATGAGCTCTCTTAC |  |
|  |  |
| AGCATCTCAAATGATAACCGAA |  |
|  |  |
| CAGCCATAATCCTCTGAATGAT |  |
|  |  |
| GTCCAAGAGTAGCCTGTGGAAT |  |
|  |  |
| TTCATATGTTATACTCAGATAA |  |
|  |  |
| GAGATTGGCATTGATGCTCAAT |  |
|  |  |
| GTGAGATAAGGTTGTTCAATAA |  |
|  |  |
| CGCCAGTTAGCTCAATATCATG |  |
|  |  |
| TTGGCAGTTCTTCAACAGTTTA |  |
|  |  |
| GCCCAGGCAAGGCGTAGGTTAA |  |
|  |  |
| TGCACATAGTGATTTAGATGAA |  |
|  |  |
| CTCTGAGAGACTGAAGATTCGA |  |
|  |  |
| TCCTTAGTAAAGGACTTATCAA |  |
|  |  |
| CGGGACAAGCTCAATAACTTA |  |
|  |  |
| CACAAAGAAAGTAGTAGACTAC |  |
|  |  |
| TGCTGAGAAGTCCAATGTTAAA |  |
|  |  |
| TTCCCGTGGTTGGTTAGAATAT |  |
|  |  |
| GTCAGAGATTGTAAATGGTCAA |  |
|  |  |
| AGGAGTCTTTGACATTAATAA |  |
|  |  |
| ACGCCTATCTCTTCAAGTACAT |  |
|  |  |
| AATCCGTCAAATTCTTGTATAA |  |
|  |  |
| GCAATGAAAGTAAACGTGTAT |  |
|  |  |
| AAGCCAGCACCTGATGAAACTT |  |
|  |  |
| TGCTGTTGAAATGTTGTGAAAT |  |
|  |  |
| AGAACTCCATTTGGATATCAAA |  |
|  |  |
| GAGAGAGGATTTCTGTAACTAG |  |
|  |  |
| AATGGATGATGTCACGACTTAA |  |
|  |  |
| GGCCAAGAATCTCTTCGTTCTT |  |
|  |  |
| TTGGCAGCAACTGTATTTGAAT |  |
|  |  |
| AAGCGCTGCAAGTACAAAATT |  |
|  |  |
| GGCTACAAATGTGCAGAGGTAA |  |
|  |  |
| CAGGATTGGTTTCTTCAATAAA |  |
|  |  |
| AGGAGTTAAGGGAACAATTAAA |  |
|  |  |
| AGTGCTTAAACAGTTAACTGAA |  |
|  |  |
| TTCGCAATTCGTGCTGATCATG |  |
|  |  |
| AGCACCTGAACTCCAGTGATAT |  |
|  |  |
| TCCACAGTTTATGTTTCATTTA |  |
|  |  |
| AGGCCCGTTTCCTCCTTTCAA |  |
|  |  |
| AACTGGCCATGTTATTGATATC |  |
|  |  |
| GCGCAACTCTCATGCAGATTT |  |
|  |  |
| GGGCCTGGATGTAGTTTATAAT |  |
|  |  |
| TGCGGTGTGACTAGCTTTAAA |  |
|  |  |
| GCCAAACAAATCCAAGATCAA |  |
|  |  |
| CCACAACAGCAAACCAGTTTA |  |
|  |  |
| CCGAAACTCTTTATAGACTAT |  |
|  |  |
| ACTGGAGAGAGTGCTAGTGAAT |  |
|  |  |
| TAGAGGTAACTTGTTTAATGTA |  |
|  |  |
| GTCATGGCATCTGTTTACATTT |  |
|  |  |
| AACCGTTTATTGTTTGATGAAA |  |
|  |  |
| AAGCCACAATATTGAATCTAAA |  |
|  |  |
| AGGAATGAATATGAACGTTCTT |  |
|  |  |
| TCCATTCCATTTATTCACACT |  |
|  |  |
| CAGTGATGACATGCTTATTAAG |  |
|  |  |
| GAGAGAGCAACAGCATTGTAAA |  |
|  |  |
| GCCAGTCTGAAGCAGCGTTATA |  |
|  |  |
| TGGCAAGAGGATTATGGCTAAC |  |
|  |  |
| TGGCAAGAGGATTATGGCTAA |  |
|  |  |
| CAGACGTTCTCGTAAGGACAAA |  |
|  |  |
| CCAGGATTATGTTGTGACTGAT |  |
|  |  |
| CCTACGAGATGAACTCTTCAA |  |
|  |  |
| TGGGAAGGATGACGTTGATAAC |  |
|  |  |
| ATCTCATCATCTCTCTATGCAA |  |
|  |  |
| GAGTCCTTCAATAATGTTAAAC |  |
|  |  |
| TTGCTAGTTCTTCCTTGTAGAG |  |
|  |  |
| GTCTGTCACTTTCCATGCATAA |  |
|  |  |
| AGCAGTGTCTGTCACTTTCCAT |  |
|  |  |
| AAACCTTTGTGTGCTGGTTTAT |  |
|  |  |
| TGGCATCATAGTTGTGTATGAT |  |
|  |  |
| AATCCCGAATATGATTATTTAT |  |
|  |  |
| ATTACACTATCTGATTAATAGT |  |
|  |  |
| CACAATTGGTGTGGATTTCAAA |  |
|  |  |
| AGCAATGAATTTGCAATCTGAA |  |
|  |  |
| AACAGTTACTCAGATAATTCAA |  |
|  |  |
| TGCTCTGTTACTACTTAAAGAA |  |
|  |  |
| AGCTAACTTTGTAGAACATAAA |  |
|  |  |
| AGGTGTGGACAGGAATATTAAG |  |
|  |  |
| AGGAGTTCATGACAAAGGTTTC |  |
|  |  |
| ACCACACCATATCAGGGATCAT |  |
|  |  |
| TTGCCATGAAATGAATGAATTA |  |
|  |  |
| GGTATCTAATCAGATTCCTAAT |  |
|  |  |
| ACCACCTCATGTTGTTAAAGTA |  |
|  |  |
| TGCTGCGAGAAGCACAGAATAA |  |
|  |  |
| TTGGTGTTAATTGAAAGTATAA |  |
|  |  |
| CTCTGGGACGTTGGATCGCTAC |  |
|  |  |
| ACTGCAGAAGCTGTTCAGTATT |  |
|  |  |
| ACTACATAATGACCTGTTCAAA |  |
|  |  |
| TCTGGGATCAGTTATAATATAT |  |
|  |  |
| TAGTGAAGCACTTCTATCCAAA |  |
|  |  |
| TGGAAAGTAGTTGTTTAACTTT |  |
|  |  |
| AAGCAAGTATCTAATACTAGAG |  |
|  |  |
| AAGAAAGCATCTCCTTAATGAA |  |
|  |  |
| ATCGCACCGTGCTGTAAATGTG |  |
|  |  |
| GCCACAGGAACTGGTACATTCA |  |
|  |  |
| TGGGAGCTATCAAGAACCTCA |  |
|  |  |
| TGCAGACGATTGCCTACAGCAT |  |
|  |  |
| CTGGCTCTTCATCCTCTTCAAA |  |
|  |  |
| ACCCATCGGACTGACAGTGAAA |  |
|  |  |
| TGTAGCTACACTTCAGATTAAA |  |
|  |  |
| CAGTATAGATCTCTATTTCTAT |  |
|  |  |
| CTCAGAGCAGAAGTTATGATTT |  |
|  |  |
| ACTCCAGTATGTGAAAGTTAAT |  |
|  |  |
| GTGAGTTAGAAACAAACTTAAA |  |
|  |  |
| TGGCGAGAATGTGAAAGCCATA |  |
|  |  |
| TGCACAATCAGTGTTGCTCAAA |  |
|  |  |
| GGCAGAGGAGGTGAACAAATTA |  |
|  |  |
| CACATGTTATCTGCTAAACTAG |  |
|  |  |
| TTGTCTGAATGTTCACCTTTAA |  |
|  |  |
| CAGTCCCTTTCTCACATGTTAT |  |
|  |  |
| CCGACCCTATTCCTTTATTGAA |  |
|  |  |
| GTCCTACAATAAACTTCCGTA |  |
|  |  |
| GTTGCTCACTATTACGTATAAT |  |
|  |  |
| GAGAGCTAGTCTGTATTGCTAC |  |
|  |  |
| AAGTGTACATATGAAGATTGAA |  |
|  |  |
| AGATCAGAACCTTCACAGAAAT |  |
|  |  |
| AAGGCAAATTCTTTAATCAAAT |  |
|  |  |
| ACCAGTTACACTGAGATTGTAA |  |
|  |  |
| TTGGAGCTGATTAGGCGTTTA |  |
|  |  |
| TGGCCACCTATGGGAAGGAAAC |  |
|  |  |
| GGAAGAGAGATTTAAGACTTAT |  |
|  |  |
| CGGATATATTTCCCTGCTGTT |  |
|  |  |
| AAGGGTTCACCTCACACTTGAA |  |
|  |  |
| AGGATAGAAATCCCATTCAAAT |  |
|  |  |
| CTGACAGCACCAGGAAACCAAA |  |
|  |  |
| TACTGGAGAACTTTAAACTAAA |  |
|  |  |
| AGGTTACAAACCTACCATTAAA |  |
|  |  |
| CCAGCACCAAGAGATTATTTAA |  |
|  |  |
| AGCTGCGCAGTTTCATTGCTAA |  |
|  |  |
| TGGAGTGAAGATGATCTTAATT |  |
|  |  |
| GAGACCTGTTGTGCTGTGTATT |  |
|  |  |
| CCCACTCTATACCAAGGATATT |  |
|  |  |
| GTGTCTGATCTTGGTGTTCAAA |  |
|  |  |
| GGCAGAACTTGCCAAGATTATG |  |
|  |  |
| CATAGACGAAGTCGTGAACAAA |  |
|  |  |
| AACCGAGCAATCTCTGGCGAAC |  |
|  |  |
| ATCCCAGGATCAAACAGGAAAT |  |
|  |  |
| CCTGGAGCACTTCCACAAGTAT |  |
|  |  |
| CACCCGTGATATTAAGCAATTA |  |
|  |  |
| ACCTCTTCAAGTGCAAGCCAAT |  |
|  |  |
| GCGAAGGAAATTGAAGTGAAAT |  |
|  |  |
| ACGAATCAATCTGACTGGAGAG |  |
|  |  |
| TAGTGGTAATTTGTATAATCAA |  |
|  |  |
| GACCAAGGAATCTGGATTTAA |  |
|  |  |
| GTGACAGAGAAACTTTGTTAAA |  |
|  |  |
| TGGTACTGAACCCGAATTTAA |  |
|  |  |
| AAGGAAGAATTTCCAAATGAAA |  |
|  |  |
| CTACGTGGTGGAAATAGTAAA |  |
|  |  |
| ACCAGACTATCAGTGCTTTGAT |  |
|  |  |
| TACTGCTCAACTTTGCAGAAAT |  |
|  |  |
| AGCACTTATCTTGGCTACTAAT |  |
|  |  |
| CTGTAAGGCTGTGCACAAACAT |  |
|  |  |
| GGCCGTAGTAAGCATTAATAAT |  |
|  |  |
| ACTGAACTCTAGGATATTTAAT |  |
|  |  |
| GCGGGACTATGGGAAACGGGAT |  |
|  |  |
| AGCCGAGCAAGTGAGAGAAGAA |  |
|  |  |
| TGGAATGGTAGCTATGATTAT |  |
|  |  |
| CTTCAACCATGTTGACATCAAA |  |
|  |  |
| TACAGAGAAAGCTGAAGACAAA |  |
|  |  |
| ACTAGCTGGTATGTTTCATAAA |  |
|  |  |
| GCAGTCGTATTCAGAGATAAA |  |
|  |  |
| GACGGTGGGTGTGATTGCAATA |  |
|  |  |
| AACCGTTAATTCCTTGGTTTAA |  |
|  |  |
| TTTCTTCTATGTGAAACTTGAA |  |
|  |  |
| ACCAACTCATTTCTTATATCAT |  |
|  |  |
| GTGGATTGATGTTTAAAGAAAC |  |
|  |  |
| GTGTTATTATCTACTTCTGAAT |  |
|  |  |
| ACTGCAAGTTGTTGGACTAAA |  |
|  |  |
| ATCACAGTTTGCCATTCTTAAA |  |
|  |  |
| CGGTCAGCTTATCAAAGTGATT |  |
|  |  |
| TACATTGCAAATTGTTGAATAA |  |

**Supplementary Table 5** Target sequences (complementary to the Guide strands) of active and inactive shRNAs in ricin resistant screen study.
